# *In Silico* evaluation and identification of fungi capable of producing endo-inulinase enzyme

**DOI:** 10.1371/journal.pone.0200607

**Published:** 2018-07-12

**Authors:** Jayaram Chikkerur, Ashis Kumar Samanta, Arindam Dhali, Atul Purushottam Kolte, Sohini Roy, Pratheepa Maria

**Affiliations:** 1 Animal Nutrition Division, ICAR-National Institute of Animal Nutrition and Physiology, Bengaluru, Karnataka, India; 2 Department of Microbiology, School of Sciences, Jain University, Bengaluru, Karnataka, India; 3 Bioenergetics and Environmental Sciences Division, ICAR-National Institute of Animal Nutrition and Physiology, Bengaluru, Karnataka, India; 4 Division of Genomic Resources, ICAR-National Bureau of Agricultural Insect Resources, Bengaluru, Karnataka, India; Saint Louis University, UNITED STATES

## Abstract

The enzyme endo-inulinase hydrolyzes inulin to short chain fructooligosaccharides (FOS) that are potential prebiotics with many health promoting benefits. Although the raw materials for inulin production are inexpensive and readily available, commercial production of FOS from inulin is limited due to inadequate availability of the enzyme source. This study aimed to identify the fungi capable of producing endo-inulinase based on the *in silico* analysis of proteins retrieved from non-redundant protein sequence database. The endo-inulinase of *Aspergillus ficuum* was used as reference sequence. The amino acid sequences with >90% sequence coverage, belonging to different fungi were retrieved from the database and used for constructing three-dimensional (3D) protein models using SWISS-MODEL and Bagheerath H. The 3D models of comparable quality as that of the reference endo-inulinase were selected based on QMEAN Z score. The selected models were evaluated and validated for different structural and functional qualities using Pro-Q, ProSA, PSN-QA, VERIFY-3D, PROCHECK, PROTSAV metaserver, STRAP, molecular docking, and molecular dynamic simulation analyses. A total of 230 proteins belonging to 53 fungal species exhibited sequence coverage >90%. Sixty one protein sequences with >60% sequence identity were modeled as endo-inulinase with higher QMEAN Z Score. The evaluations and validations of these 61 selected models for different structural and functional qualities revealed that 60 models belonging to 22 fungal species exhibited native like structure and unique motifs and residues as that of the reference endo-inulinase. Further, these models also exhibited similar kind of interaction between the active site around the conserved glutamate residue and substrate as that of the reference endo-inulinase. In conclusion, based on the current study, 22 fungal species could be identified as endo-inulinase producer. Nevertheless, further biological assessment of their capability for producing endo-inulinase is imminent if they are to be used for commercial endo-inulinase production for application in FOS industry.

## Introduction

Fructooligosaccharides (FOS) are the linear short chain oligomers with repeating fructose molecules linked together by β (2–1) glycosidic bond with a terminal glucose molecule. FOS is one of the potential prebiotics [1, 2]. It stimulates the growth of bifidobacteria and lactobacilli in the gut [3,4] and leads to the production of short chain fatty acids which further stimulate the growth of colorectal mucosal cells, slow down atrophy of the mucosa and reduce the risk of harmful changes in the colon [5]. FOS is the first approved prebiotic food supplement for health benefits [6]. The global market of FOS was found to be 134.0 kilo tons in 2015 and is expected to grow significantly in next few years [5]. Therefore, large scale commercial production of high quality FOS will be required to fulfill the increasing demand.

Although, fructooligosaccharides can be produced by acid or enzymatic hydrolysis of inulin, the enzymatic method is mostly preferred as it produces high quality FOS economically [7]. Fructosyltransferase and endo-inulinase are the primary enzymes that are capable of producing FOS by hydrolyzing sucrose and inulin respectively [8]. Nevertheless, FOS production through fructosyltransferase hydrolysis has disadvantages. In this method, only limited amount of sucrose can be converted to oilgofructose due to the inhibition of enzymatic activity by released glucose molecules. Moreover, along with FOS, the higher amount of sucrose and glucose also remains in the enzyme hydrolysate that is undesirable and requires further purification [9]. In contrast, production of FOS by endo-inulinase is a single step process that yields high quality FOS and excludes any further purification steps [10, 11].

Endo-inulinases are found in plants and microbes. Microbial endo-inulinases are preferred over plants as they are easy to cultivate on large scale. Moreover, enzyme yield is higher in microbes than plants. Fungi are the major source of microbial endo-inulinase as they are capable to produce the enzyme in high quantity as compared to bacteria [10]. Although, endo-inulinase activity has been demonstrated in a few fungal species previously [12–20] their potential for producing FOS suitable for nutraceutical applications is yet to be established. Therefore, it is necessary to explore the diverse group of fungi capable of producing endo-inulinase that would help in identifying potential species for large scale production and commercial application of this enzyme.

Intensive laboratory screening of a large number of fungi for endo-inulinase would be an extremely tedious and expensive process. On the other hand, recent progress in the field of computational biology has made it possible to screen unique microorganisms from a population for a particular biological function. Further, the identified organisms can be evaluated at the laboratory to validate the predicted biological function. Several theories and methodologies have been developed previously based on computational methods to characterize microbial enzyme [21] and to study the protein-ligand interactions and drug design through *in silico* approaches [22–27]. In recent years application of Molecular dynamic (MD) simulations in studying the chemical and biological systems is expanded significantly [28]. The huge application potential has led to implementations of MD in many software packages. GROMACS is one of the widely used application package for MD simulations [28]. The objective of the current study was to identify different fungi capable of producing endo-inulinase enzyme based on the *in silico* analysis of non-redundant protein sequence database.

## Results

### Retrieval of protein sequences and homology modeling

A total of 230 protein sequences were retrieved from the NCBI non-redundant protein sequence database that exhibited >90% sequence coverage with the reference endo-inulinase protein sequence of *Aspergillus ficuum*. The list of the retrieved proteins is provided in the S1 Table that included hypothetical proteins (119), invertase (5), levanase (12), glycosyl hydrolase family 32 proteins (16), β-fructofuranosidase (10), inulinase (25), fructosyltransferase (6), exo-inulinase (18), endo-inulinase (10) and other proteins (9). The retrieved proteins sequences were found to be from 53 fungal species belonging to the genera *Aspergillus*, *Fusarium*, *Penicillium*, *Talaromyces*, *Pseudogymnoascus*, *Stachybotrys*, *Phytophthora*, *Pyrenochaeta*, *Rhizopus*, *Mucor*, *Macrophomina*, and *Oidiodendron*.

**Fig 1:**
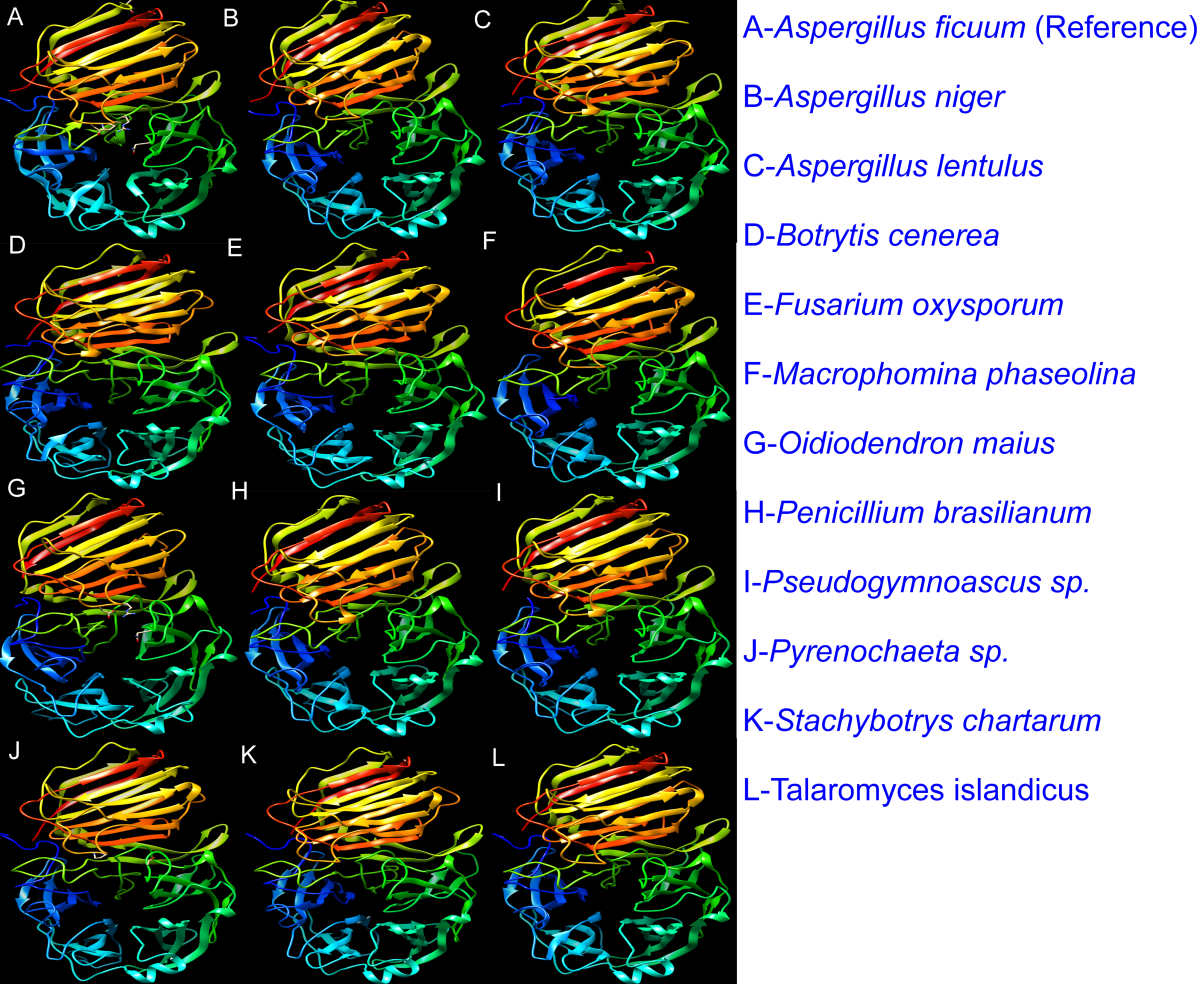
Three-dimensional (3D) models of representative fungal proteins that resembled as endo-inulinase. 3D models were generated from sequences retrieved from the non-redundant protein sequence database using SWISS-MODEL web server (https://swissmodel.expasy.org/interactive). The model images were created using USCF-Chimera.

The 230 selected proteins were subjected to Swiss homology modeling [29–32] to generate 3D protein models. The models were constructed based on the experimentally characterized template structure. The reference based homology modeling is more appropriate as the predicted models were constructed based on the native template structure. The analysis revealed that out of the 230 selected protein sequences, 100 were modeled as endo-inulinase. Further, it was observed that out of 100, the amino acid sequences of proteins which showed sequence identity >60% with reference endo-inulinase, were modeled as endo-inulinase with good quality QMEAN Z scores [33] of >-3 (Fig 1 & S1 Dataset) The details of sequence identity and QMEAN Z scores of 61 predicted models were presented in S2 Table).

**Fig 2:**
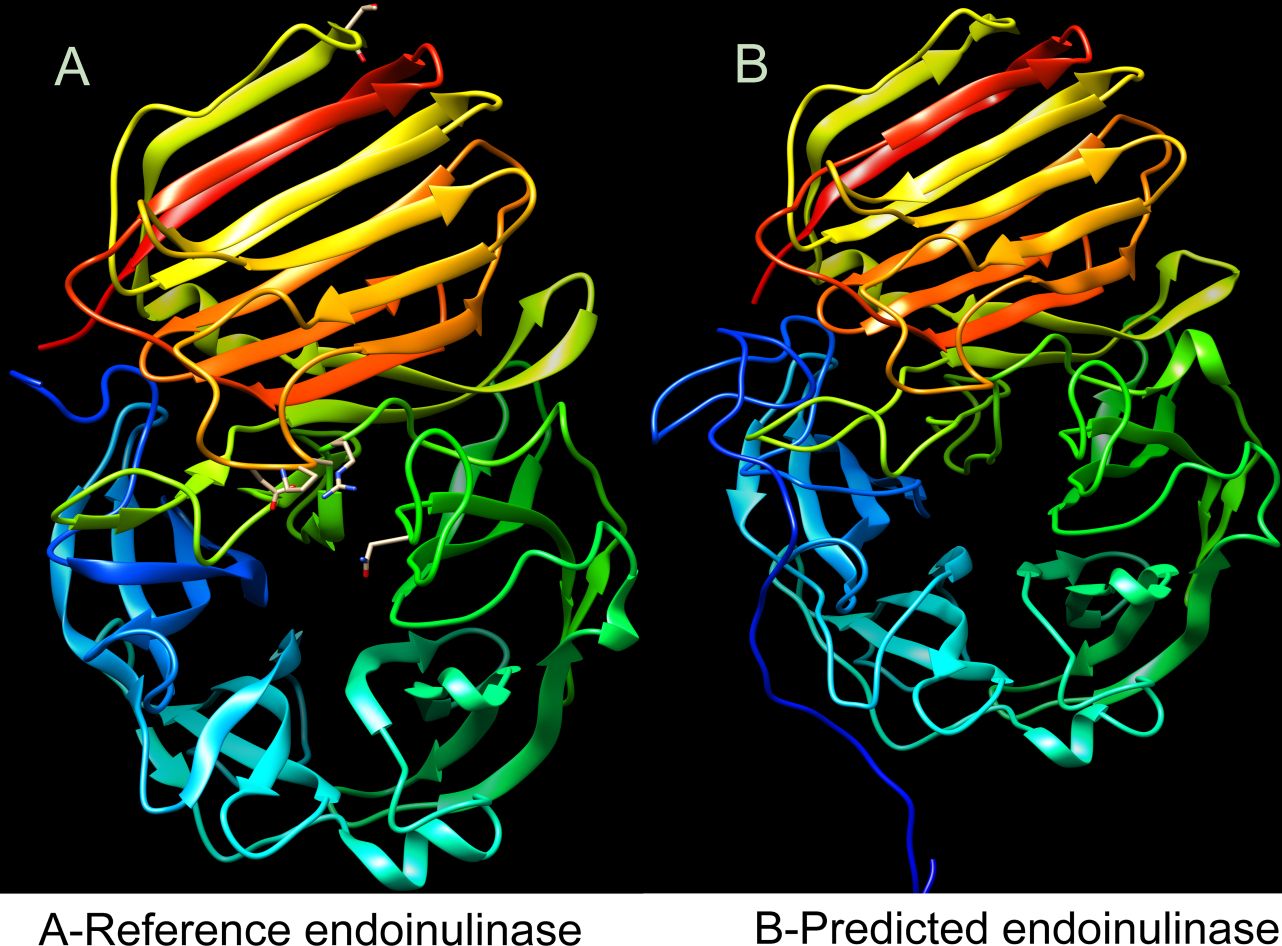
Three-dimensional (3D) model of representative fungal protein, constructed based on denovo/ab-initio method of protein modeling using Bhageerath H web server (http://www.scfbio-iitd.res.in/bhageerath/bhageerath_h.jsp). A- Reference endo-inulinase (3SC7); B- Denovo 3D model of predicted protein from *Aspergillus fumigatus* resembling native endo-inulinase like structure. The model images were created using USCF-Chimera.

The native endo-inulinase like model of these 61 protein models were further corroborated by denovo/abinitio method of protein modeling. The denovo/abinitio protein models were constructed using Bhageerath H (a webserver for homology/ab-initio mode of protein tertiary structure prediction) [34]. The denovo protein modeling also revealed that the predicted models resembles native endo-inulinase like structure (Fig 2. & S2 Dataset). Hence these 61 endo-inulinase like predicted protein models were selected for analyzing and validating different protein qualities.

### Quality evaluation of predicted 3D protein models

The structural stability and correctness of the 61 predicted protein models selected from homology modeling was evaluated using different protein stability validation modules like Pro-Q, PROSA, PSN-QA, Verify 3D and PROCHECK.

Pro-Q [35] evaluates the model structure by checking the residue wise local quality of a model structure. Pro-Q employs neural network approach which integrates contacts among atoms and residues, solvent accessible surfaces, and secondary structure statistics. Based on the structural features, Pro-Q generates LG-Score and MaxSub score. The quality of the predicted models can be explained based on these scores. Predicted protein model with LG score > 3 and MaxSub score > 0.1 was considered as good quality structures. Pro-Q analysis revealed that all the 61 models exhibited LG score of >4.24 and MaxSub score of >0.34 that indicated their good structural quality (S3 Table).

**Fig 3:**
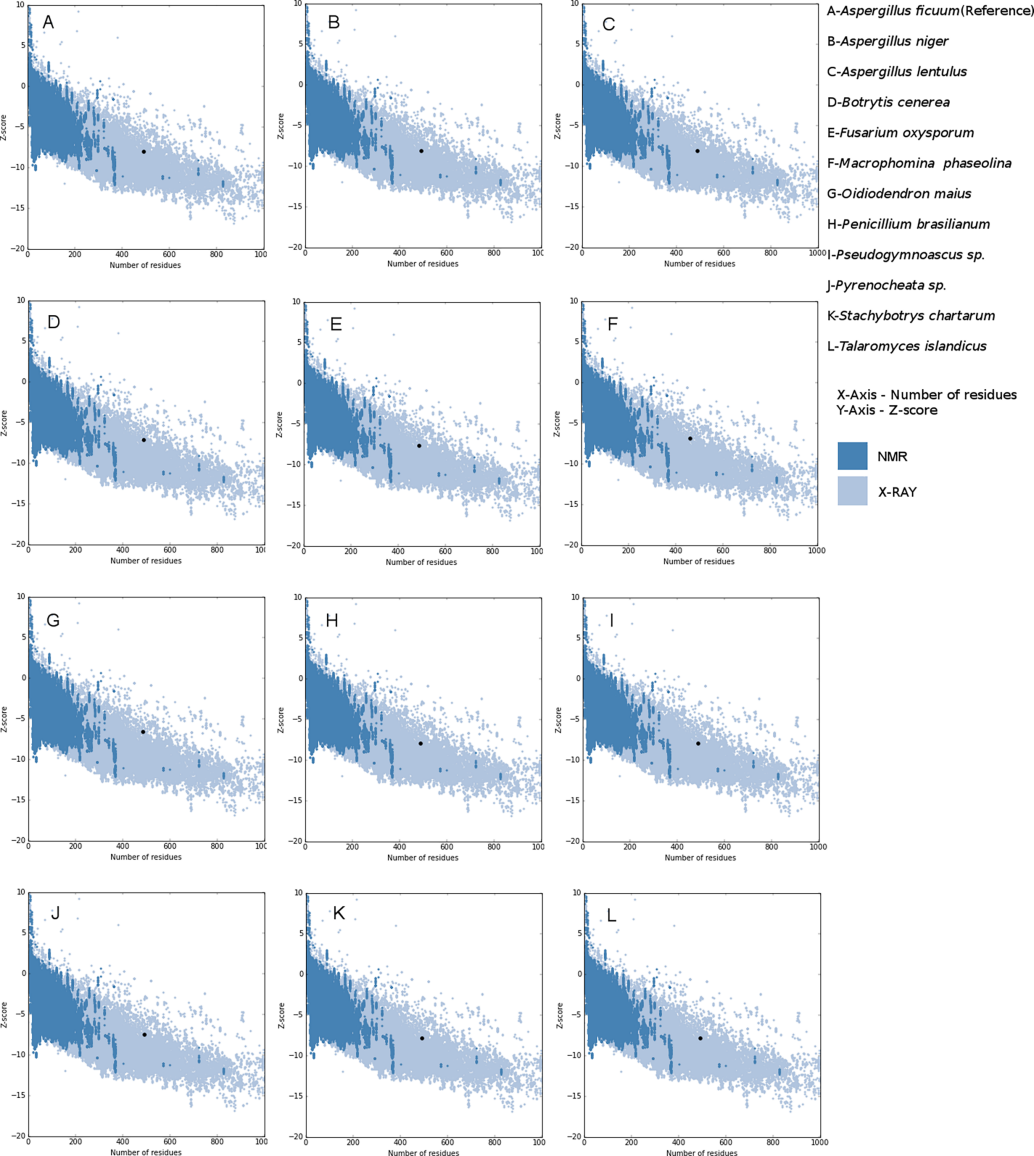
Plot of residue energies with Z-scores of representative 3D protein models, generated using PROSA web (https://prosa.services.came.sbg.ac.at/prosa.php). The blue color shaded region in the plot corresponds to Z-scores of experimentally determined protein structures characterized by NMR analysis; grey color shaded region corresponds to Z-scores of experimentally determined protein structures characterized by X-Ray diffraction studies. The Z-scores of predicted 3D models falls within the range of experimentally determined native like protein structures.

PROSA [36, 37] diagnoses the protein tertiary structure by matching with the statistics of available experimentally determined structures. This module applies statistics of Cα potentials of mean force to evaluate the quality of predicted protein structures. This module plots Z-scores with its residue energies. Z-scores falling within the range of experimentally determined structures distinguishes native like protein structures from erroneous structures. The ProSA Z-score plots of 61 predicted protein models revealed that the predicted models were within the range of experimentally determined native like protein structures (Fig 3 & S3 Dataset and S3 Table).

**Fig 4:**
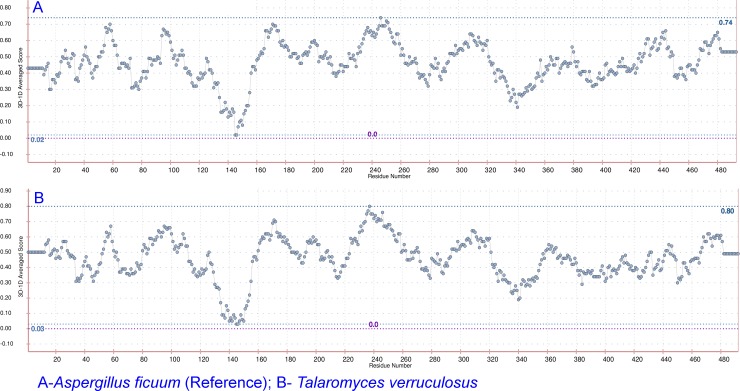
Plot showing the average 3D-ID score for each residue of A. *Aspergillus ficuum* reference endo-inulinase and B. Predicted endo-inulinase from *Talaromyces verruculosus*. The plot and scores are generated using VERIFY 3D webserver (http://servicesn.mbi.ucla.edu/Verify3d/).

PSN-QA [38] is a network based approach for the quality analysis of predicted protein models. These networks are constructed using non-covalent interactions between the side chains of the polypeptides. This module assigns a rank to the predicted models based on its closeness to its native like protein structure. PSN-QA rank of predicted models beyond 16 represents native-like conformation and a rank below 10 represents non-native like conformation. PSN-QA rank was found >16 for all the models indicating their native-like conformation except for the hypothetical protein V496_07217 (gb|KFY54637.1) from *Pseudogymnoascus sp*. *w*here the rank was 15.9 (S3 Table). Verify 3D [39, 40] module evaluates the quality of tertiary structure of the predicted protein by checking the residue wise compatibility of amino acid to the whole protein. This module measures 3D-ID profile scores for each residue. The protein structures are evaluated based on this residue level score for checking the suitability of each residue to its structural environment, defined by the secondary structure, burial position and polarity of positions in a structure. 3D-ID profile score of ≥ 0.2 for a residue, makes it suitable to structural environment. If 80% of amino acid residues in a protein are with 3D-ID profile score ≥ 0.2, then the protein is more likely stable. The analysis of protein tertiary structure of the models using VERIFY-3D tool revealed that 86.9 to 100% of the residues of the models exhibited 3D-1D profile score of ≥0.2 indicating the overall good structural quality of all 61 models (Fig 4 & S4 Dataset and S3 Table).

**Fig 5:**
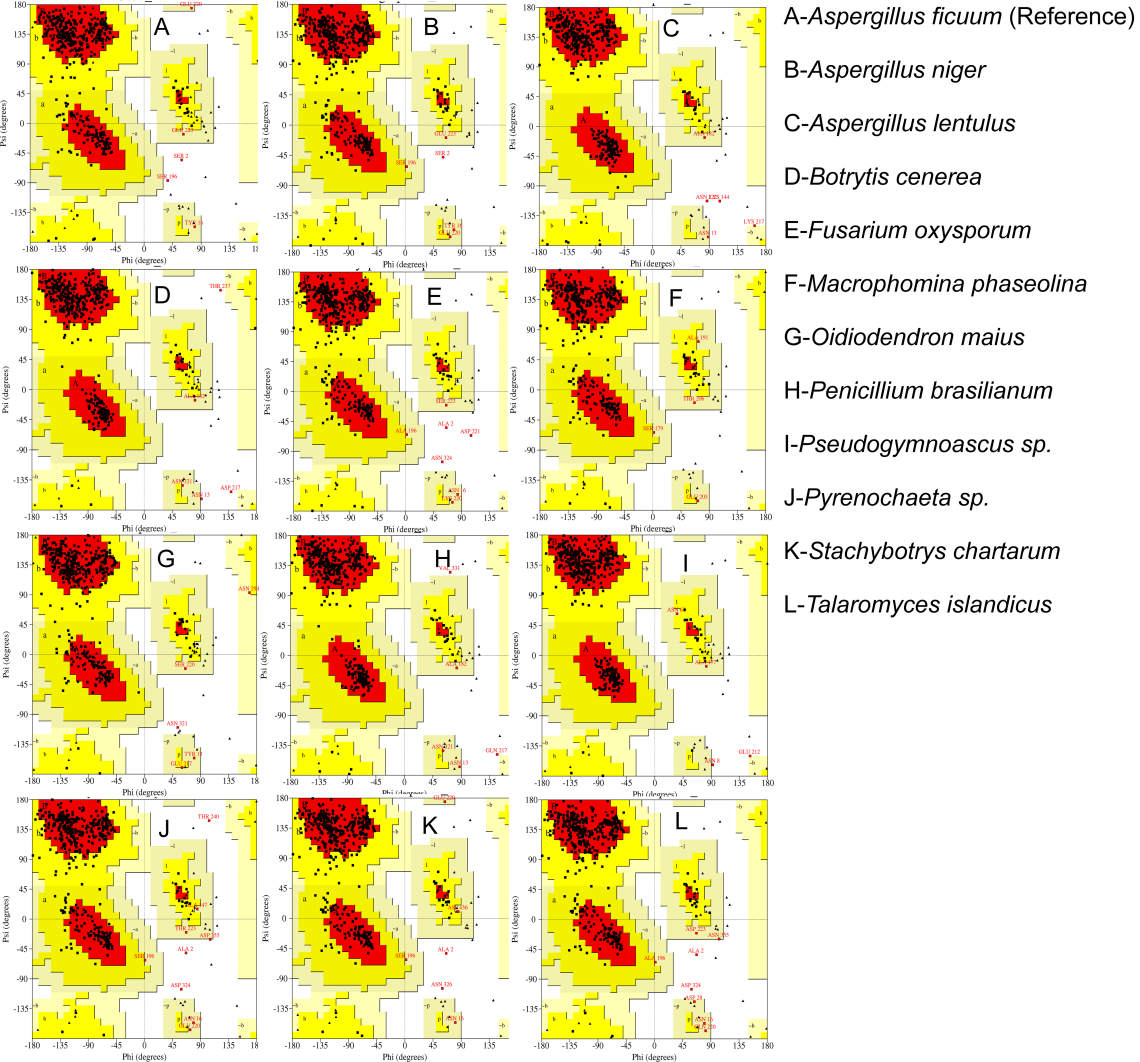
Ramachandran plots of representative proteins that resembled as endo-inulinase. The plots were generated using PROCHECK module of PROTSAV webserver (http://www.scfbio-iitd.res.in/software/proteomics/protsav.jsp).

The secondary structure of predicted protein models and experimentally characterized endo-inulinase were evaluated by Ramachandran Plot generated through procheck module [41] of ProtSAV server [42]. The Ramachandran Plot depicts that 81.6 to 85.6% of the total residues of the models were found within the most favored, additionally allowed and allowed regions and the respective value was found to be 84.8% for reference native endo-inulinase (Fig 5 & S5 Dataset and S4 Table).

The above protein validation modules emphasizes that the predicted models are good and reflect native like protein structures.

### *In silico* validation of 3D protein models as endo-inulinase

**Fig 6:**
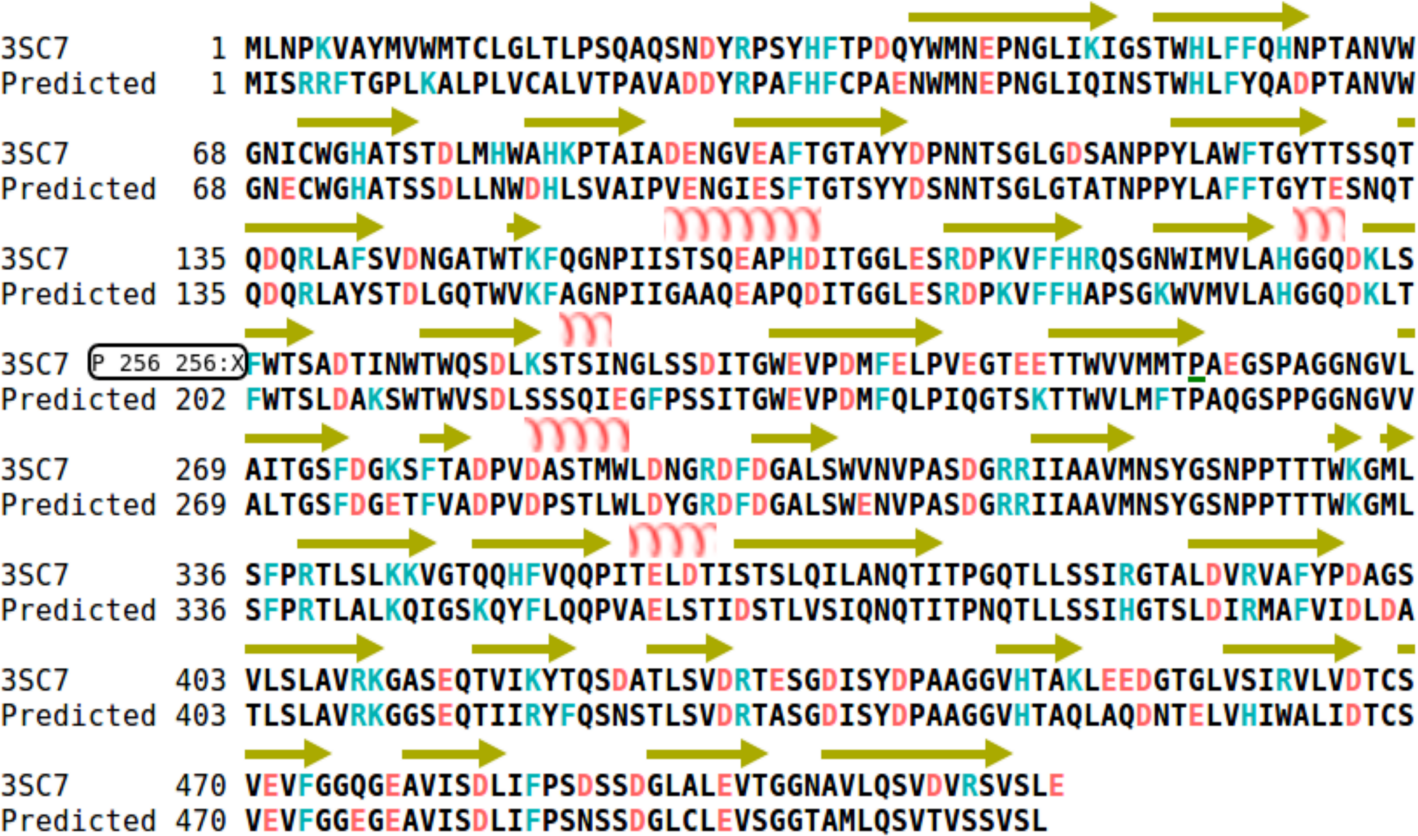
Structure based sequence alignment of reference endo-inulinase from *Aspergillus ficuum* and the predicted protein endo-inulinase model from *Talaromyces sp*. for identifying unique motifs and residues required for endo-activity. The Sequences are aligned using webserver (http://bioinformatics.org/strap/aa/).

The structure based sequence alignment of the 3D protein models with the reference native endo-inulinase revealed that the conserved (W-M-N-D(E)-P-N-G), loop1 (P-T(A)-A-N-V-W-G-N) and loop4 (A-V-M-N-S-Y-G-S-N-P) motifs [43] were existed in the respective positions in all the models with minor modifications in the loop1 and loop4 motifs except for the inulinase (dbj|GAO81637.1) from *Aspergillus udagawae*, where the catalytic glutamate (E43) in the W-M-N-D(E)-P-N-G motif was replaced with aspartate (D) and loop1 motif was not conserved hence excluded from the docking analysis (S6 Dataset; S5 Table). However, the specific amino acid residues T-100, G-196, V-234 and D-298 that are unique to endo-inulinase [43] and critical for its biological activity were found conserved in the positions in all the models (Fig 6 & S6 Dataset and S5 Table).

**Fig 7:**
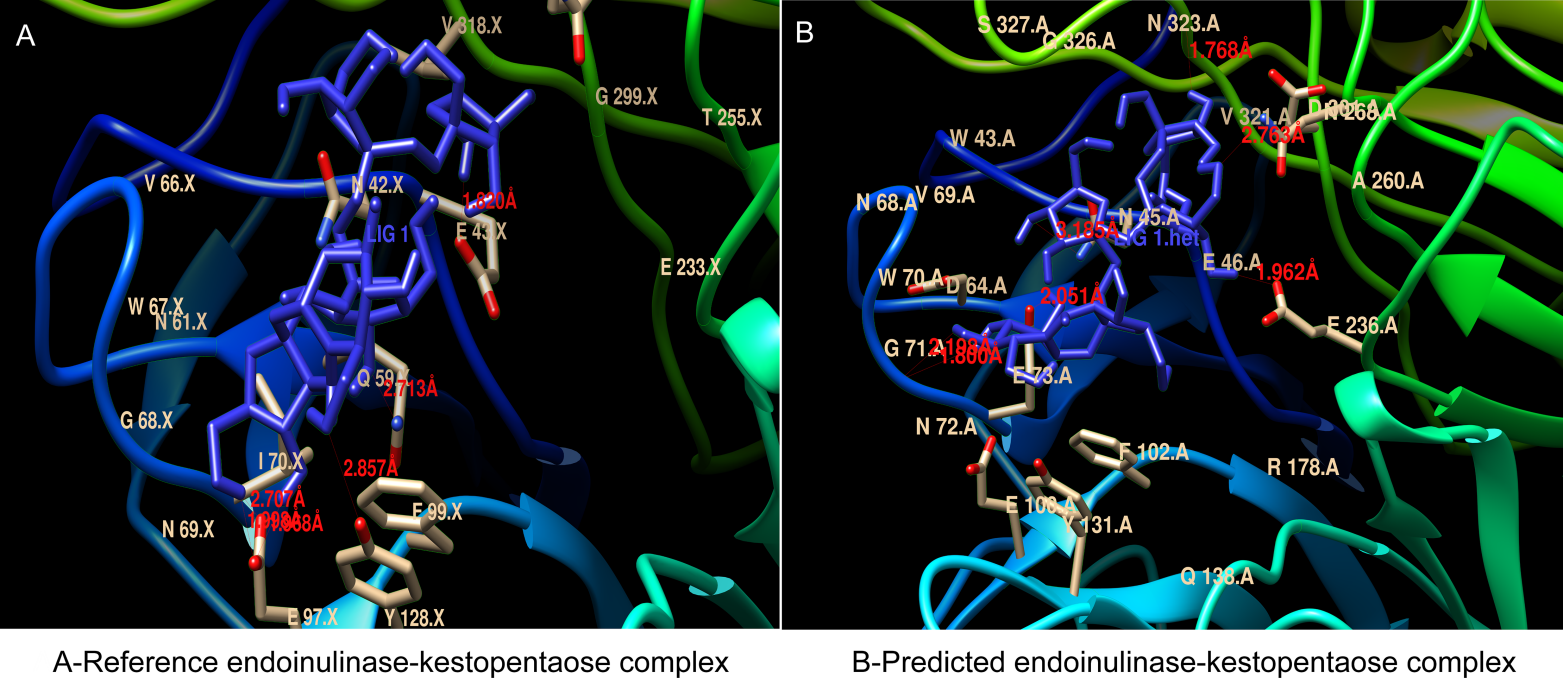
The Interaction between the active site around the conserved glutamate residue and substrate (kestopentaose) in the three-dimensional protein models of reference endo-inulinase from *Aspergillus ficuum* and predicted endo-inulinase from *Talaromyces sp*. The docking was performed using MGL python tool and autodock 4. The interactions and hydrogen bonds formed between the substrate and the active site of enzyme was visualized using UCSF Chimera. The hydrogen bonds formed and their size were indicated in red color.

Docking of kestopentaose (substrate) with the active site around the catalytic residue GLU43, revealed that certain residues play an important role in substrate binding and endo activity of the standard endo-inulinase enzyme from *Aspergillus ficuum* [43]. In the present study, docking was performed between all the predicted protein models including endo-inulinase from *A*. *ficcum* and substrate kestopentaose. The docking results indicated similar interaction between kestopentaose and the active site around the conserved glutamate residue of all the models as described earlier [43]. The information of interacting amino acids and the number of hydrogen bonds formed between the amino acid residues in the active site and the substrate, the binding energy of enzyme-substrate complex are provided in the S6 Table and Fig 7 & S7 Dataset.

### Molecular Dynamic (MD) simulations and MMPBSA analysis

**Fig 8:**
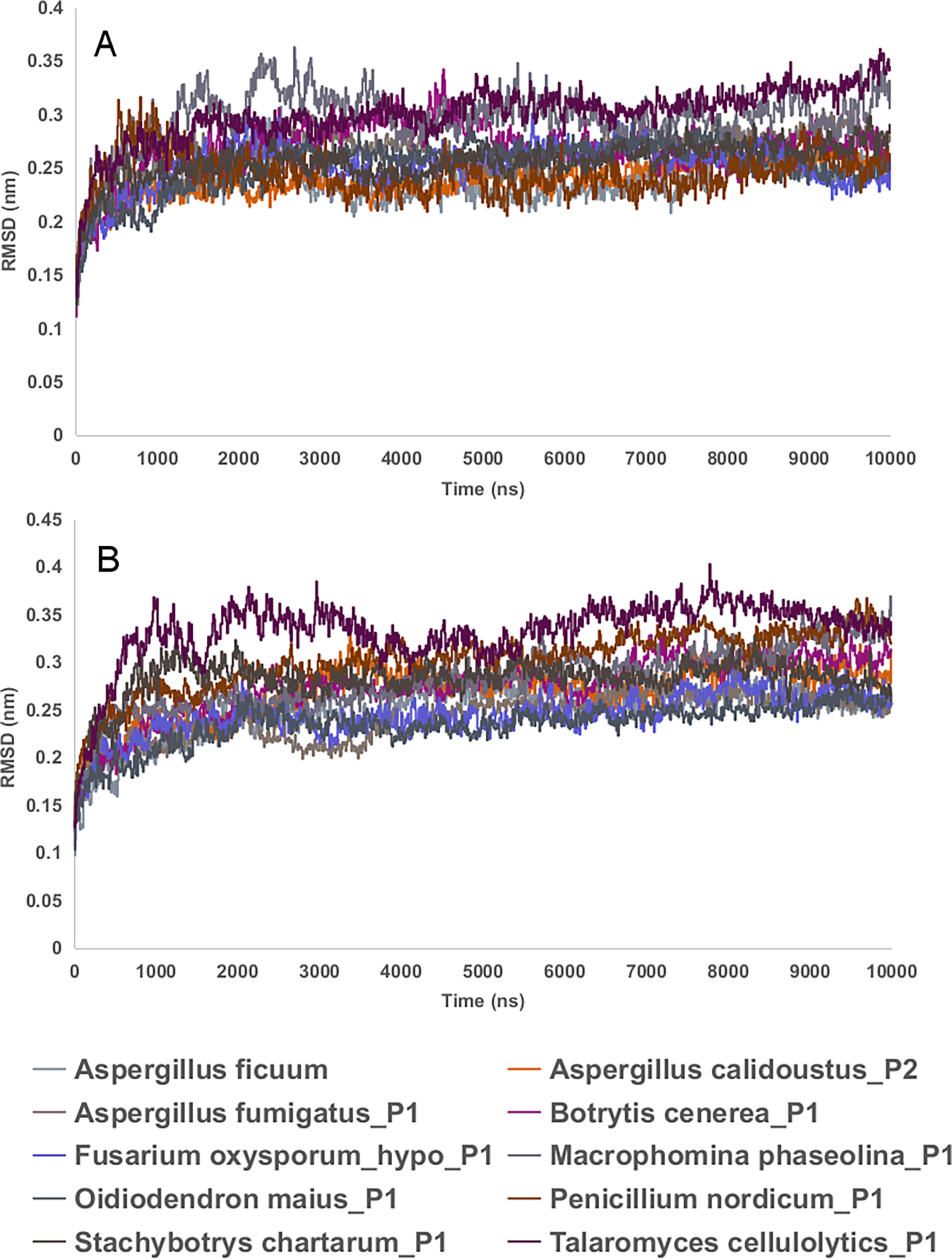
RMSD plot of protein backbone of 9 predicted endo-inulinase models and a reference endo-inulinase (3SC7) after 10ns of MD simulations. The data was generated using gromacs rms function. A. Protein back bone of enzyme-substrate complex; B. Protein backbone of enzyme without substrate. The plots were made in the work sheet.

Molecular dynamic simulations were performed for 9 predicted endo-inulinase models and a reference endo-inulinase (3SC7), docked with kestopentaose substrate, using Groningen Machine for Chemical Simulations (GROMACS) [44, 45] in order to check their stability. The MD simulation revealed that the root mean square distance (RMSD) of protein backbone of enzyme substrate complex and enzyme without substrate, was converged after 4ns of simulation and it is stable for the complete simulation run (Fig 8).

Further, it was noticed that there is no considerable variation in the RMSD of protein backbone of enzyme substrate complex and enzyme without substrate. (Fig 8).

**Fig 9:**
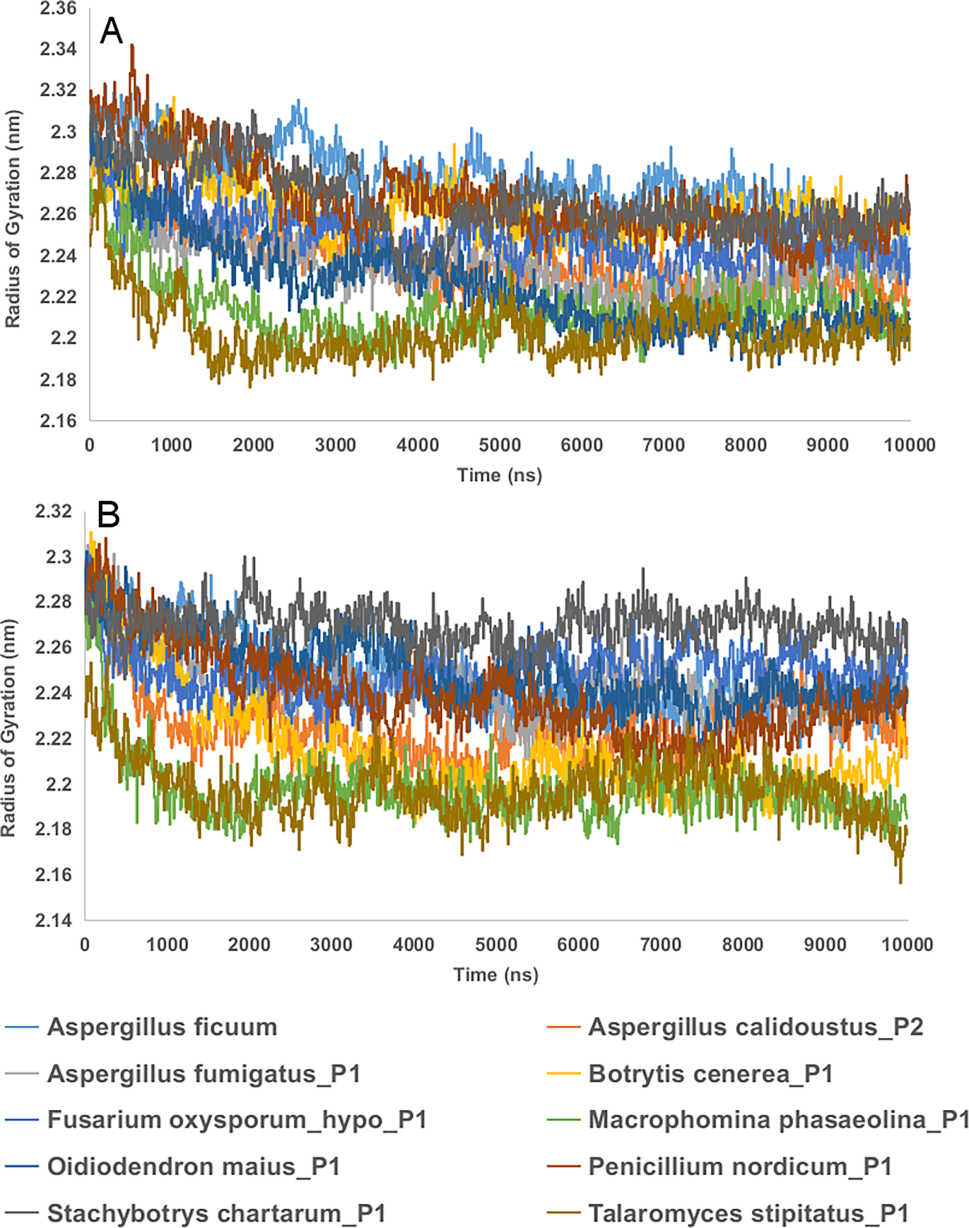
Radius of gyration (Rg) plot of protein backbone of 9 predicted endo-inulinase models and a reference endo-inulinase (3SC7) after 10ns of MD simulations. The data was generated using gromacs gyrate function. A. Protein back bone of enzyme-substrate complex; B. Protein backbone of enzyme without substrate. The plots were made in the work sheet.

Radius of gyration (Rg) explains the compactness of the protein. If a protein is stably folded, it will likely maintain a relatively steady value of Rg. If a protein unfolds, its Rg will change over time. The MD simulation reveals that Rg of protein backbone of enzyme substrate complex and enzyme without substrate are relatively stable and shows no significant variation (Fig 9).

The last 1ns simulation results revealed that the hydrogen bonding observed between the kestopentaose and amino acid residues of the catalytic subsites of the predicted endo-inulinase models (S7 Table), was similar to those interactions observed with *A*. *ficuum* endo-inulinase and kestopentaose, reported earlier [43]. The H-bond information of the enzyme substrate complex of 9 predicted endo-inulinase models and a reference endo-inulinase (3SC7), for first 1ns, last 1ns and for the entire 10ns MD simulation was presented in S8 Dataset.

The trajectories of last 1ns MD simulation were used to calculate the binding free energy of the enzyme-substrate complex using g_mmpbsa package [46,47]. The MMPBSA analysis revealed negative binding energy (S7 Table) of the enzyme substrate complex that indicted the reaction is spontaneous, favorable and requires less energy (S9 Dataset).

## Discussion

Endo-inulinase is the enzyme that degrades inulin into short chain FOS that are established as potential prebiotics with health promoting benefits. Although the raw materials for inulin production are inexpensive and readily available from different plant sources, commercial production of FOS from inulin is limited due to inadequate availability of the enzyme [48, 49]. In the current study, attempt was made to identify fungi capable of producing endo-inulinase enzyme based on the *in silico* analysis of non-redundant protein sequence database. The results indicated the potential of 22 fungal species that can be explored for commercial production of endo-inulinase.

Until now, only the native endo-inulinase of *Aspergillus ficuum* has been studied and characterized extensively [43]. Hence, the corresponding sequence was used as a reference for retrieving the endo-inulinase like sequences from the non-redundant protein sequence database. A total 230 such sequences belonging to 53 different fungal species could be retrieved and 3D protein models were generated using SWISS-MODEL. The homology models were further confirmed by abinitio/denovo modeling. The structural quality of the generated 3D models were evaluated on the basis of QMEAN Z score, which is a composite scoring function based on different geometrical properties and it provides both global and local quality estimates [33]. The score provides an estimate of the ‘degree of nativeness’ of the structural features observed in a model and indicates whether the model is of comparable quality to the reference template structure. The SWISS-MODEL analysis of the retrieved sequences reveled that only 61 3D models belonging to 23 fungal species conformed to be of good structural quality and resembled as the endo-inulinase like structure. Therefore, these selected 61 models were further analyzed and validated for different protein qualities using Pro-Q, ProSA, PSN-QA, VERIFY-3D and PROCHECK tools.

The quality analyses of the selected 61 3D models using the above tools revealed that all the models were structurally stable and resembled as native like endo-inulinase.

It is established that the conserved W-M-N-D(E)-P-N-G motif, loop1 motif P-T(A)-A-N-V-W-G-N, and Loop4 motif A-V-M-N-S-Y-G-S-N-P form an enlarged cavity, which is critical for endo-activity of endo-inulinase [43]. In addition to these motifs, the residues glutamate (E) in the conserved WMNEPNG motif, threonine (T-100), glycine (G-196), valine (V-234) and aspartate (D-298) are found play a critical role in its activity [43]. Therefore, the selected models were further analyzed using the structure based sequence alignment tool STRAP [50] for the presence of these unique motifs and residues in the selected 3D protein models. The unique residues were found to be conserved in all the models. Similarly, the motifs were found to be conserved in all the models except the inulinase from *Aspergillus udagawae*. Therefore, it was not included in the further investigation for assessing the likely interaction of the models with the substrate.

Based on the STRAP analysis, 60 models belonging to 22 fungal species were selected to assess the interaction between kestopentaose and the endo-inulinase specific active site through molecular docking. It is reported previously that kestopentaose is a suitable substrate for assessing the enzyme-substrate interaction of endo-inulinase [43]. The docking study revealed a similar kind of interaction between the substrate and subsites of the active site around the conserved glutamate residue for the native endo-inulinase and all the selected protein models, as described earlier for *Aspergillus ficuum* native endo-inulinase [43]. The stability of the docked complex, the enzyme-substrate interaction and binding energy calculations of 9 predicted endo-inulinase models and a reference model was verified by performing the MD simulation using gromacs [44,45] and MMPBSA analysis using g_mmpbsa package [46,47]. The MD simulation results substantiated the enzyme-substrate interactions observed between the subsites of the catalytic site and the kestopentaose of native endo-inulinase described in the earlier study [43]; while the negative binding energy obtained for enzyme substrate complex using MMPBSA analysis revealed that the complex formation is spontaneous and most favorable. The results verified the selected 60 proteins as endo-inulinase from 22 fungal species. Among 22 species identified in this study, endo-inulinase was experimentally studied and characterized in four species (Table 1).

**Table 1:** Experimentally characterized endo-inulinases from species identified in this study.

Species Name	Sequence/GenbankID	Enzyme	Reference
*Talaromyces cellulolyticus (Penicillium sp*. *strain TN-88)*	dbj|GAM42287.1	Inulinase	[51,52]
*Talaromyces purpureogenus (Penicillium purpureogenum)*	dbj|BAA12320.1	endo-inulinase precursor	[14, 53]
Penicillium subrubescencs	OKP12957.1, OKP07876.1, OKP07872.1	Extracellular endo-inulinase	[18, 54]
Aspergillus niger	UniProtKB/Swiss-Prot: O74641.1	Inulinase	[55]
*Aspergillus niger CBS 513*.*88`*	ref|XP_001394322.1	Inulinase	[56]
*Aspergillus fumigatus Z5*	gb|KMK58827.1	Inulinase	[17, 57, 58]

Although, inulinase from *Rhizoctonia sp* [19], *Rhizomucor pusilis* [20], *Thermomyces lanuginous* [59] and endo-type inulinase from *Chrysosporum pannorum* [15], *Rhizopus sp*. Strain TN-96 [16] were identified, their amino acid sequences are not available in the non-redundant protein database, hence not included in the current study.

In conclusion, based on the results of the current *in silico study*, 22 different fungal species belonging to 10 genera could be identified as endo-inulinase producer. Nevertheless, further biological assessment of their capability for producing endo-inulinase at the laboratory is imminent if they are to be used for large scale endo-inulinase production for application in FOS industry.

## Materials and methods

### Retrieval of protein sequences and homology modeling

The amino acid sequence of endo-inulinase protein of *Aspergillus ficuum* (PDB ID 3SC7) was used as reference for retrieving the highly similar (>90% sequence coverage) protein sequences belonging to different fungi from the NCBI non-redundant protein sequence database using pBLAST tool (http://blast.ncbi.nlm.nih.gov/Blast.cgi) [60]

Three-dimensional (3D) protein models of the retrieved sequences were generated using SWISS-MODEL [29–32]. The proteins modeled as endo-inulinase with QMEAN Z score of >-3 were selected for further validation [33]. The models were also generated by denovo/abinitio method using online webserver called Bhageerath H [34] and visualized using UCSF chimera [61].

### Protein quality evaluation

The selected 3D models were analyzed and validated for different protein qualities using the tools Pro-Q (residue-wise local quality) [35], ProSA (tertiary structure) [36, 37], PSN-QA (native like structure) [38], VERIFY-3D (amino acid compatibility) [39, 40] and PROCHECK (secondary structure) [41] on PROTSAV server [42].

### Structure based sequence alignment and Autodocking analysis

Endo-inulinase contains unique residues and motifs, which confer endo-hydrolysis activity to the enzyme [43]. Therefore, the selected 3D models were validated for the presence or absence of those consensus motifs and residues by using the structure based sequence alignment tool STRAP [50]. Further, the selected endo-inulinase 3D models were docked with kestopentaose (substrate) in the active site around catalytic E-43 residue to assess the likely interaction of the models with the substrate with minimum binding energy. The docking was performed using the methods of AutoDock4 [62, 63].

### Molecular dynamic simulations

The docked complexes were subjected to molecular dynamics simulations using the GROningen Machine for Chemical Simulations V4.5.4 (GROMACS) [44,45]. The GROMACS MD simulations was performed based on the methodlogy adopted from Bevans Lab [64] GROMOS96 43a1 force field was applied on 10 docked enzyme-substrate complexes and 10 enzymes without substrate, were placed in the centre of the dodecahedron box solvated in water. Topology files and other force field parameter files for the ligands were created using PRODRG2 server [65]. The docked complex and enzyme were immersed in dodecahedron water box of SPC216 water model. Total negative charges on the docked and enzyme structures were balanced by suitable number of Na+ ions to make the whole system neutral using genion program of GROMACS. The system was initially energy minimized by steepest descent minimization for 50,000 steps. After adding ions the system was again energy minimized by steepest descent minimization retaining the same parameters. The V-rescale, a modified Berendsen thermostat, temperature coupling [66] and Parrinello-Rahman pressure coupling [67] methods were used to keep the system stable at 323 K temperature and pressure of 1 bar. The Particle Mesh Ewald (PME) method [68] was selected to compute long range electrostatic interactions. A cut off distance of 14 Å was set for both Coulombic and van der Waals interactions. Rotational constraint was applied to bonds by LINCS algorithm [69].

### MMPBSA anlysis and binding energy calculation

MMPBSA analysis of last 1ns trajectories was performed using g_mmpbsa package (46, 47). The method involves calculation of three energy components: viz., Calculation of potential energy in vacuum, Calculation of polar solvation energy and Calculation of non-polar solvation energy. After calculating the three energy components, the binding energy of the complex can be calculated using a python function.

## Supporting information

S1 Dataset Three-dimensional (3D) models of the selected 61 fungal proteins that resembled as endo-inulinase. 3D models were generated from sequences retrieved from the non-redundant protein sequence database using SWISS-MODEL.
(ZIP)

S2 Dataset 61 Three-Dimensional protein models constructed based on denovo/abinitio method using Bhageerath H webserver. (ZIP)

S3 Dataset Protein structure analysis of the selected 61 three-dimensional (3D) protein models using ProSA tool explaining the predicted endo-inulinase 3D models fall within the range of experimentally determined structure. (ZIP)

S4 Dataset VERIFY-3D scores of the selected 61 three-dimensional (3D) protein models explaining the protein structure stability of the predicted endo-inulinase 3D models. (ZIP)

S5 Dataset Ramachandran plots of the selected 61 proteins that resembled as endo-inulinase. The plots were generated through PROCHECK analysis.
(ZIP)

S6 Dataset Structure based sequence alignment of the 60 selected proteins that resembled as endo-inulinase with the reference endo-inulinase from *Aspergillus ficuum* for identifying unique motifs and residues. (ZIP)

S7 Dataset Interaction between the active site around the conserved glutamate residue and substrate (kestopentaose) of the selected 60 three-dimensional protein models as well as reference endo-inulinase from *Aspergillus ficuum*. (ZIP)

S8 Dataset Results of molecular dynamic simulations: Hydrogen bond information of enzyme substrate complex of 9 predicted and reference endo-inulinase models generated using GROMACS hbond function. (ZIP)

S9 Dataset Results of g_mmpbsa: Binding energy calculation of enzyme substrate complex of 9 predicted and reference endo-inulinase models generated using g_mmpbsa package. (ZIP)

S1 Table Details of the retrieved protein sequences exhibiting >90% sequence coverage as compared to that of the reference endo-inulinase from *Aspergillus ficuum*. The sequences were retrieved from the non-redundant protein sequence database through pBLAST analysis.
(XLSX)

S2 Table List of the protein sequences that resembled as endo-inulinase along with QMEAN Z scores. (XLSX)

S3 Table List of the selected 61 protein sequences along with different quality scores obtained through Pro-Q, ProSA, PSN-QA and VERIFY-3D analyses. (XLSX)

S4 Table Results of the Ramachandran plot analysis of the selected 61 protein sequences that resembled as endo-inulinase. (XLSX)

S5 Table Results of the structure based sequence alignment of the 61 selected proteins that resembled as endo-inulinase with the reference endo-inulinase from *Aspergillus ficuum* for identifying unique motifs and residues. (XLSX)

S6 Table Results of the molecular docking study: The hydrogen bond interaction between the active site around the conserved glutamate residue and substrate (kestopentaose) of the selected 60 three-dimensional protein models as well as reference endo-inulinase from *Aspergillus ficuum*. (XLSX)

S7 Table Results of last 1ns molecular dynamic simulations and g_mmpbsa calculation of 9 predicted endo-inulinase models and reference endo-inulinase with kestopentaose: Hydrogen bonds formed between the substrate and catalytic subsites of the predicted endo-inulinase and binding energy of the enzyme substrate complex. (XLSX)
