# Supplementary figures and images for: In Silico evaluation and identification of fungi capable of producing endo-inulinase enzyme (part 1 of 4)
Source: PLoS One. 2018 Jul 12;13(7):e0200607. doi: 10.1371/journal.pone.0200607 (PMC6042768; doi:10.1371/journal.pone.0200607)

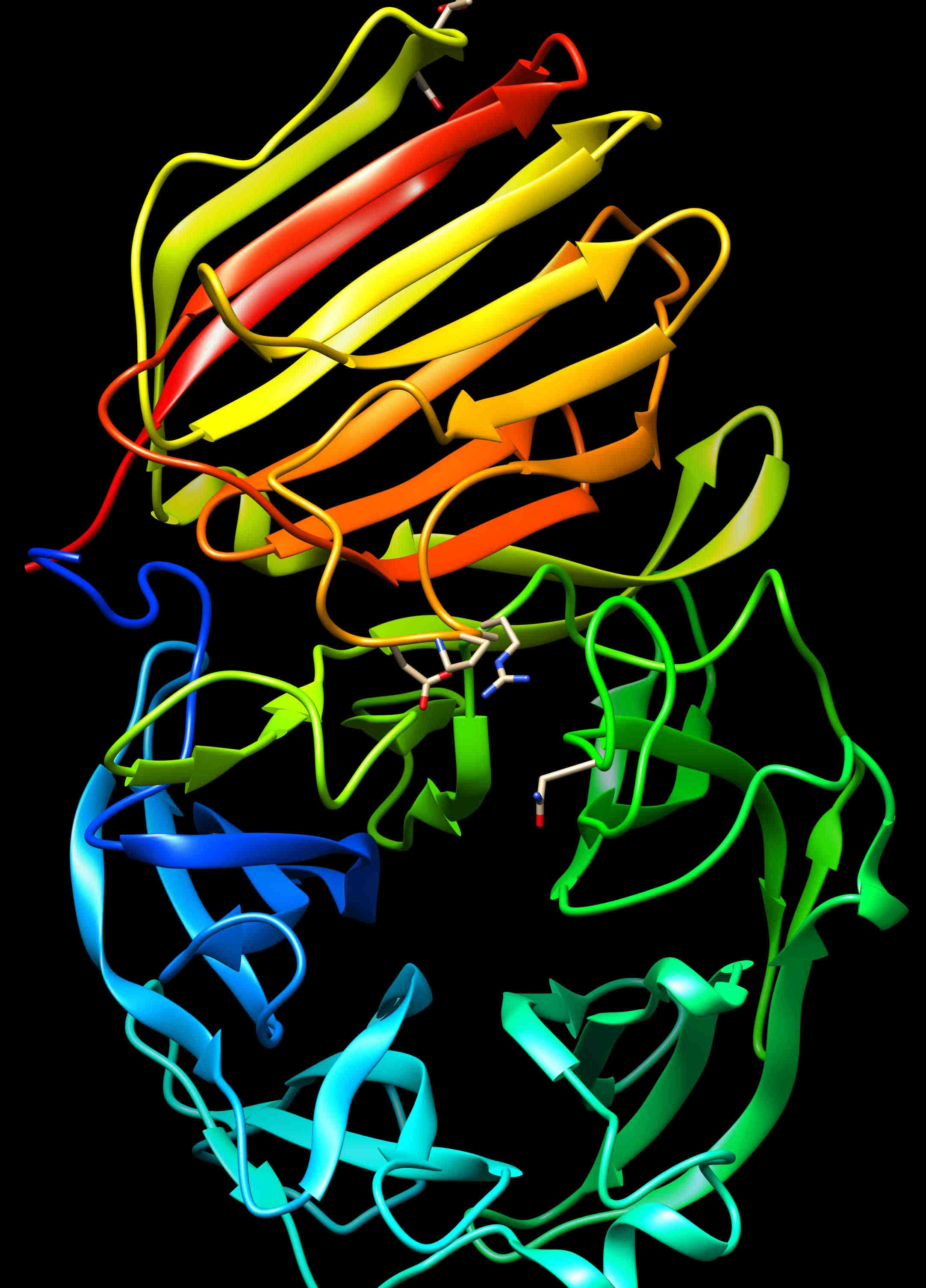

Supplement: S1 Dataset — 3D models were generated from sequences retrieved from the non-redundant protein sequence database using SWISS-MODEL. (ZIP) [file pone.0200607.s001.zip › Homology_Models/3SC7.jpg]

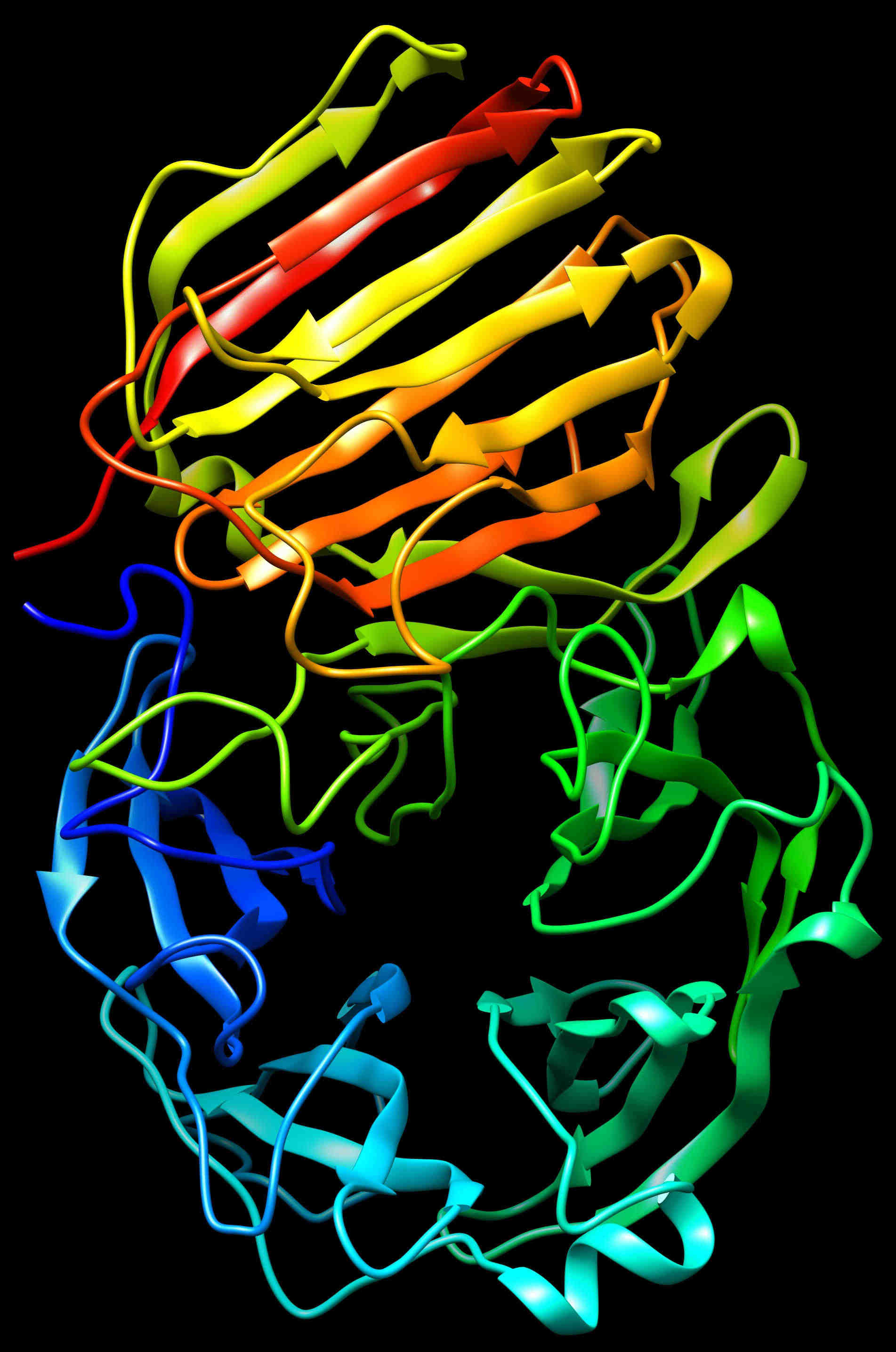

Supplement: S1 Dataset — 3D models were generated from sequences retrieved from the non-redundant protein sequence database using SWISS-MODEL. (ZIP) [file pone.0200607.s001.zip › Homology_Models/Acalidoustusp1m1.jpg]

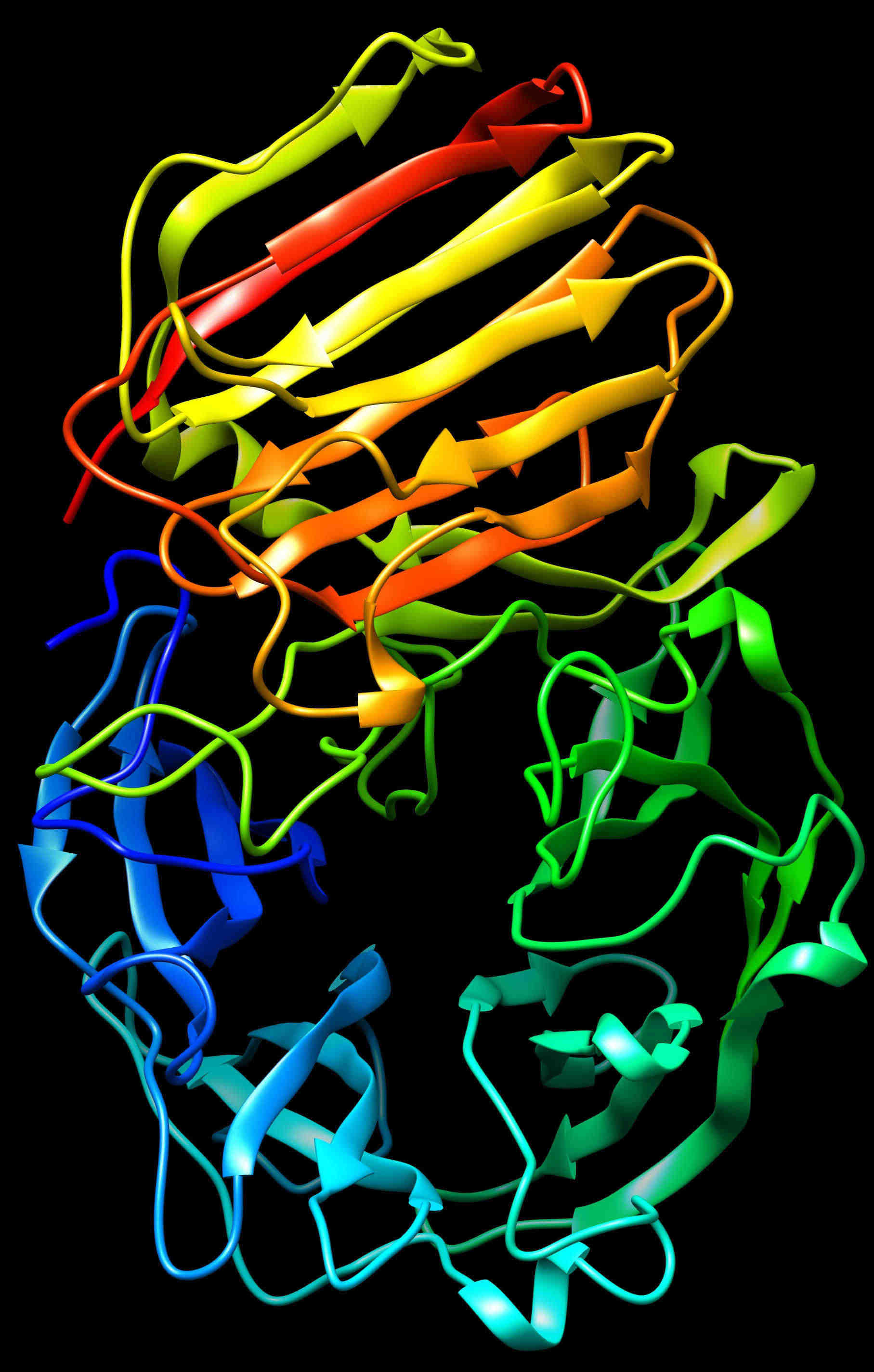

Supplement: S1 Dataset — 3D models were generated from sequences retrieved from the non-redundant protein sequence database using SWISS-MODEL. (ZIP) [file pone.0200607.s001.zip › Homology_Models/Acalidoustusp2m2.jpg]

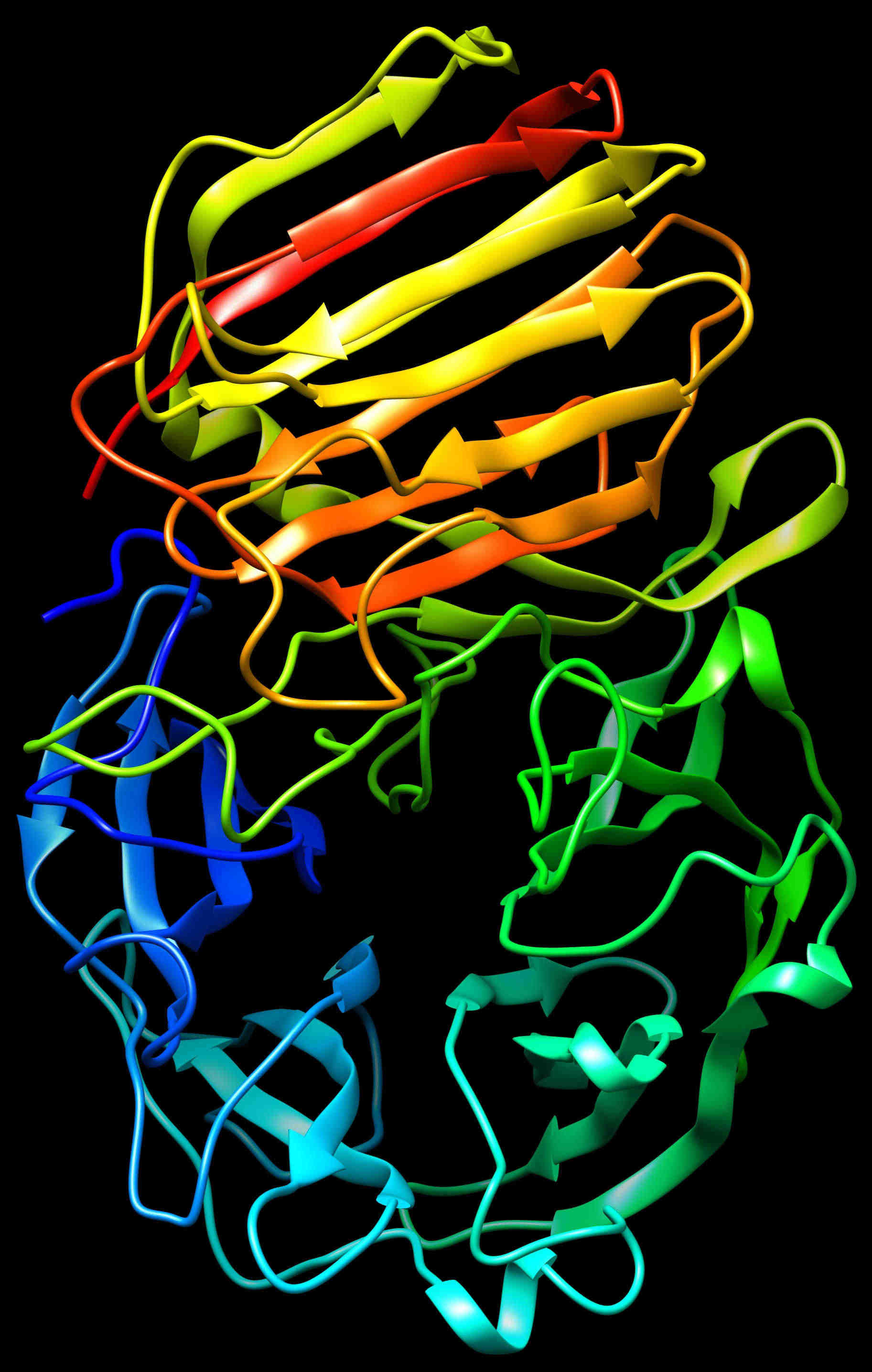

Supplement: S1 Dataset — 3D models were generated from sequences retrieved from the non-redundant protein sequence database using SWISS-MODEL. (ZIP) [file pone.0200607.s001.zip › Homology_Models/Afischerip1m2.jpg]

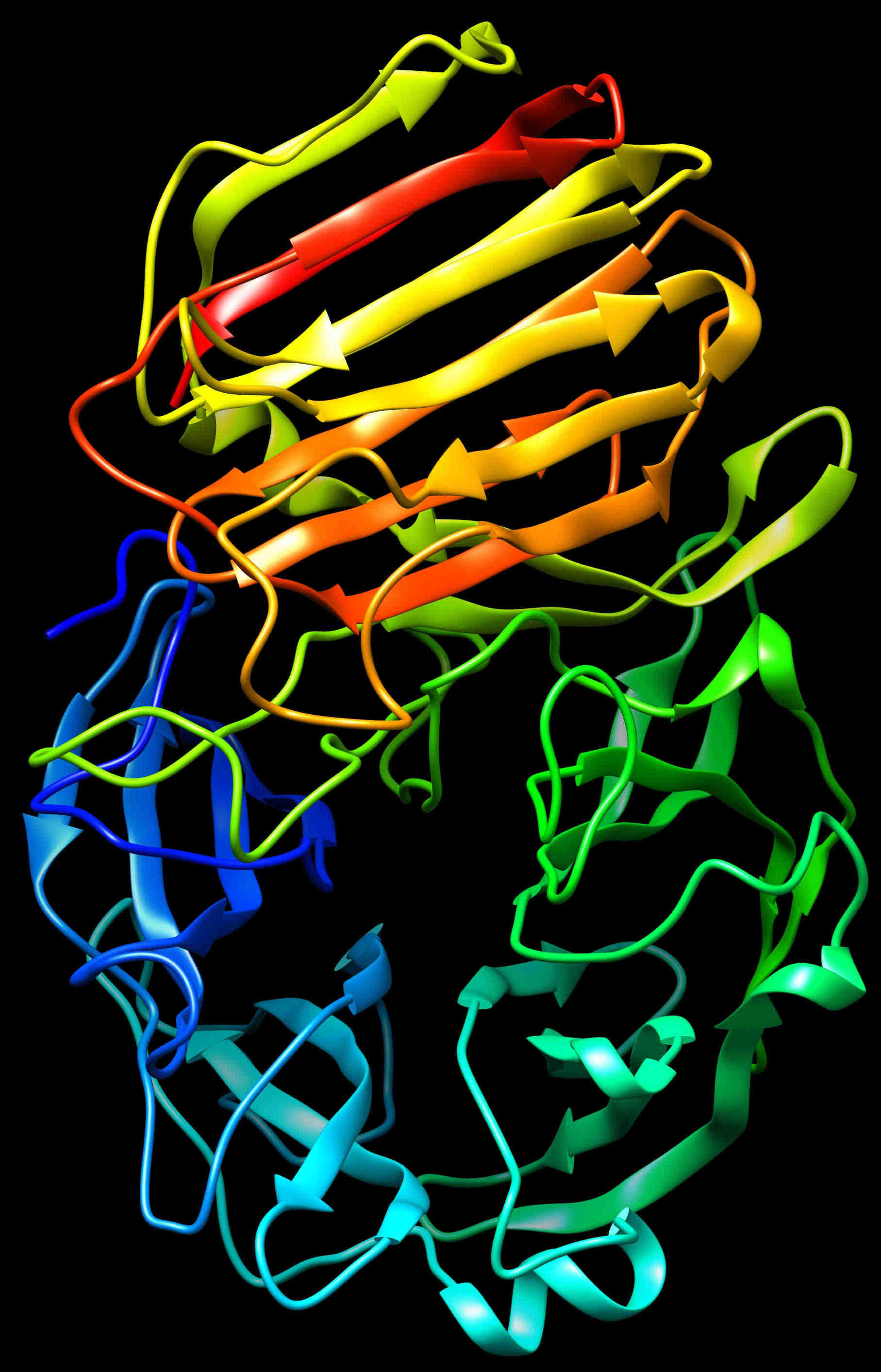

Supplement: S1 Dataset — 3D models were generated from sequences retrieved from the non-redundant protein sequence database using SWISS-MODEL. (ZIP) [file pone.0200607.s001.zip › Homology_Models/Afumigatusp1m1.jpg]

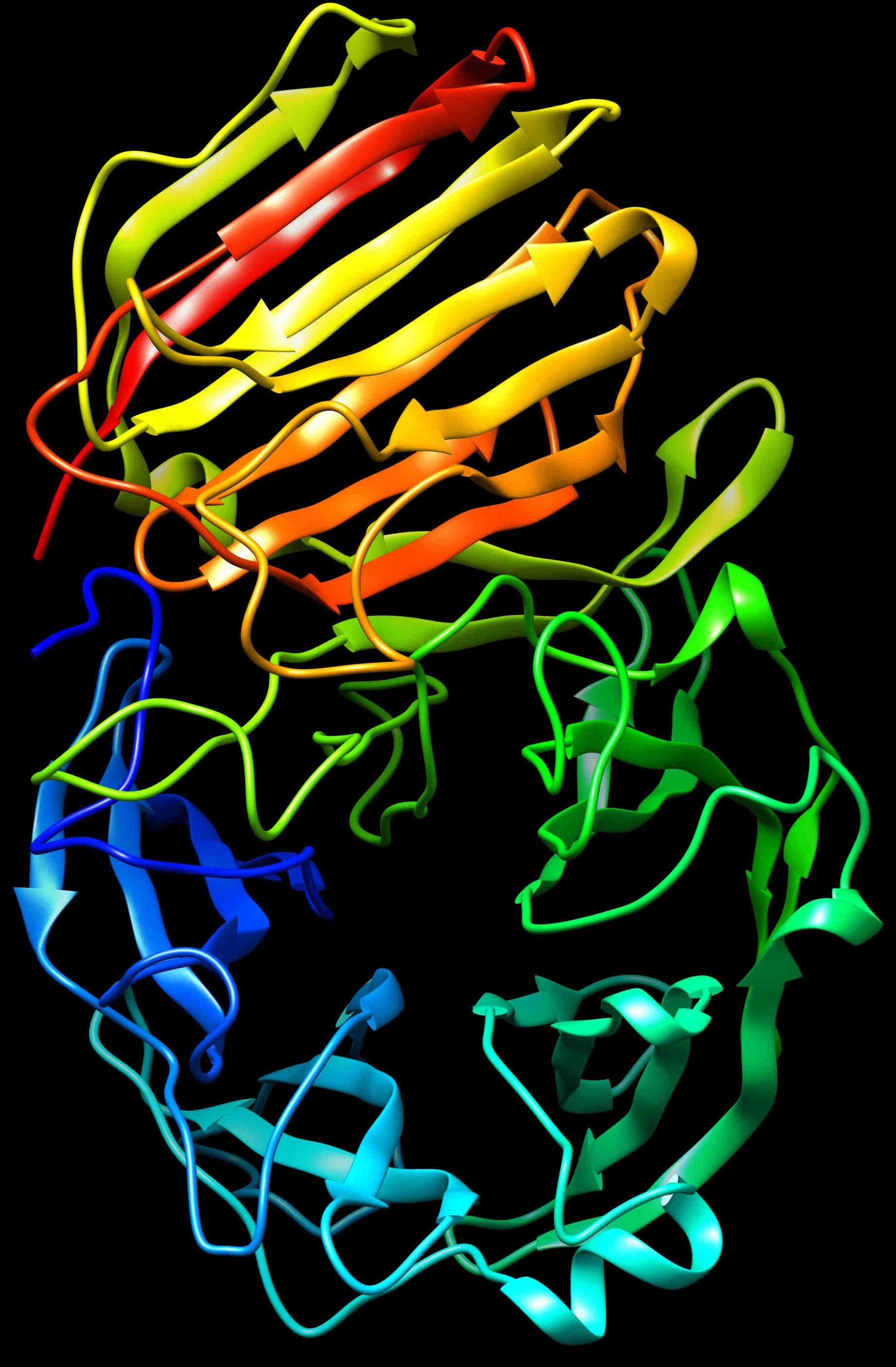

Supplement: S1 Dataset — 3D models were generated from sequences retrieved from the non-redundant protein sequence database using SWISS-MODEL. (ZIP) [file pone.0200607.s001.zip › Homology_Models/Afumigatusp2m1.jpg]

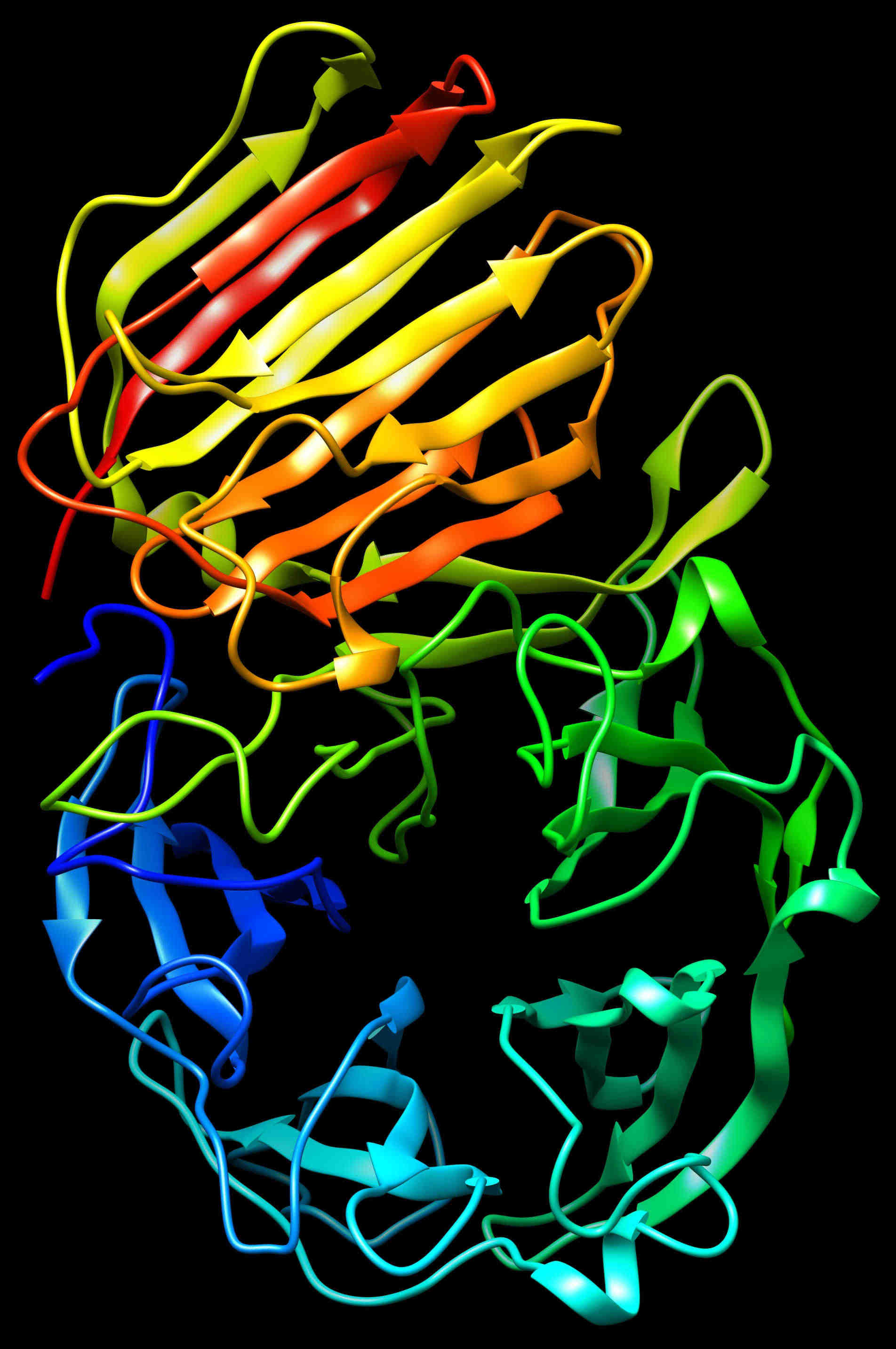

Supplement: S1 Dataset — 3D models were generated from sequences retrieved from the non-redundant protein sequence database using SWISS-MODEL. (ZIP) [file pone.0200607.s001.zip › Homology_Models/Alentulusp1m2.jpg]

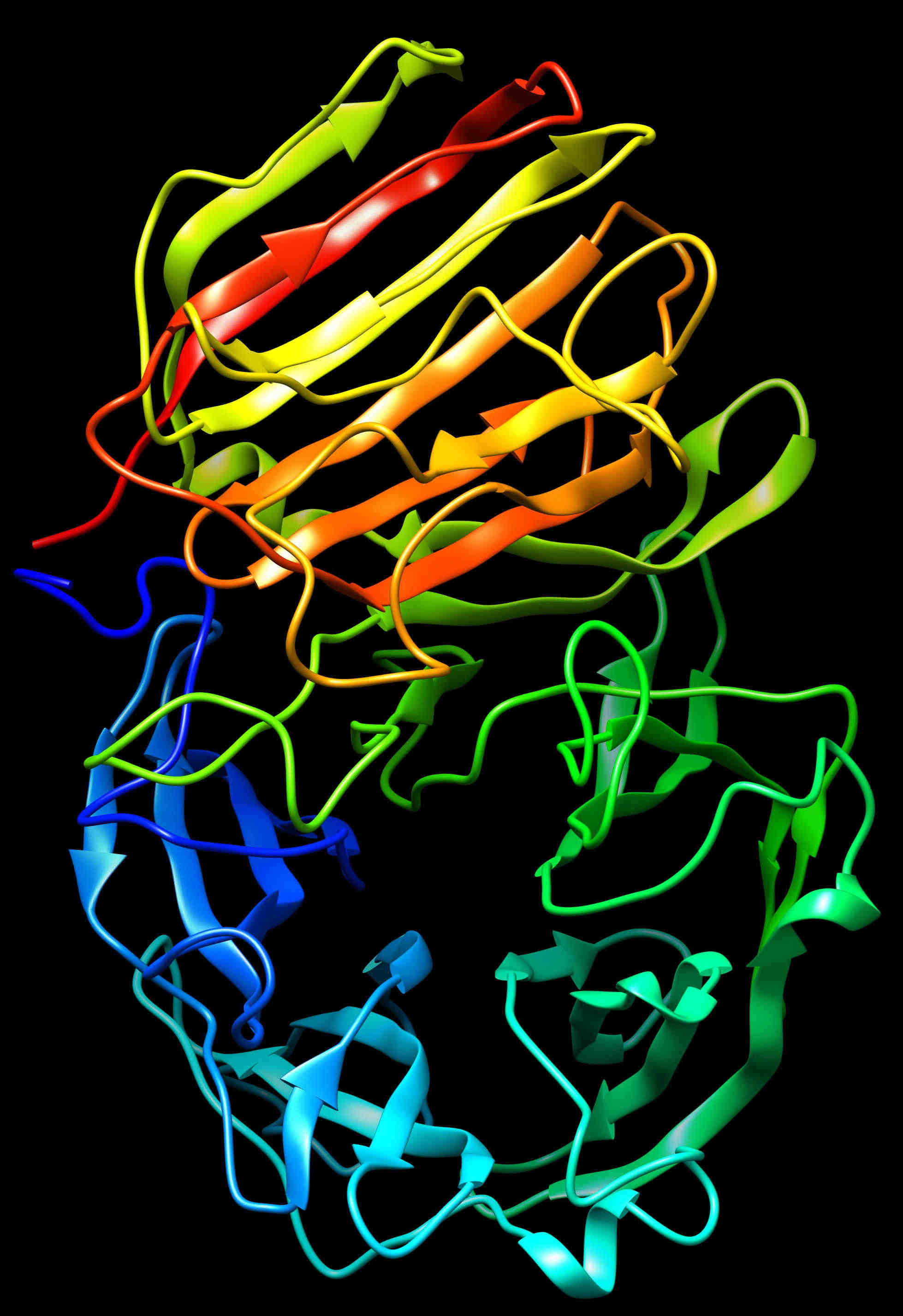

Supplement: S1 Dataset — 3D models were generated from sequences retrieved from the non-redundant protein sequence database using SWISS-MODEL. (ZIP) [file pone.0200607.s001.zip › Homology_Models/Anigerp10m1.jpg]

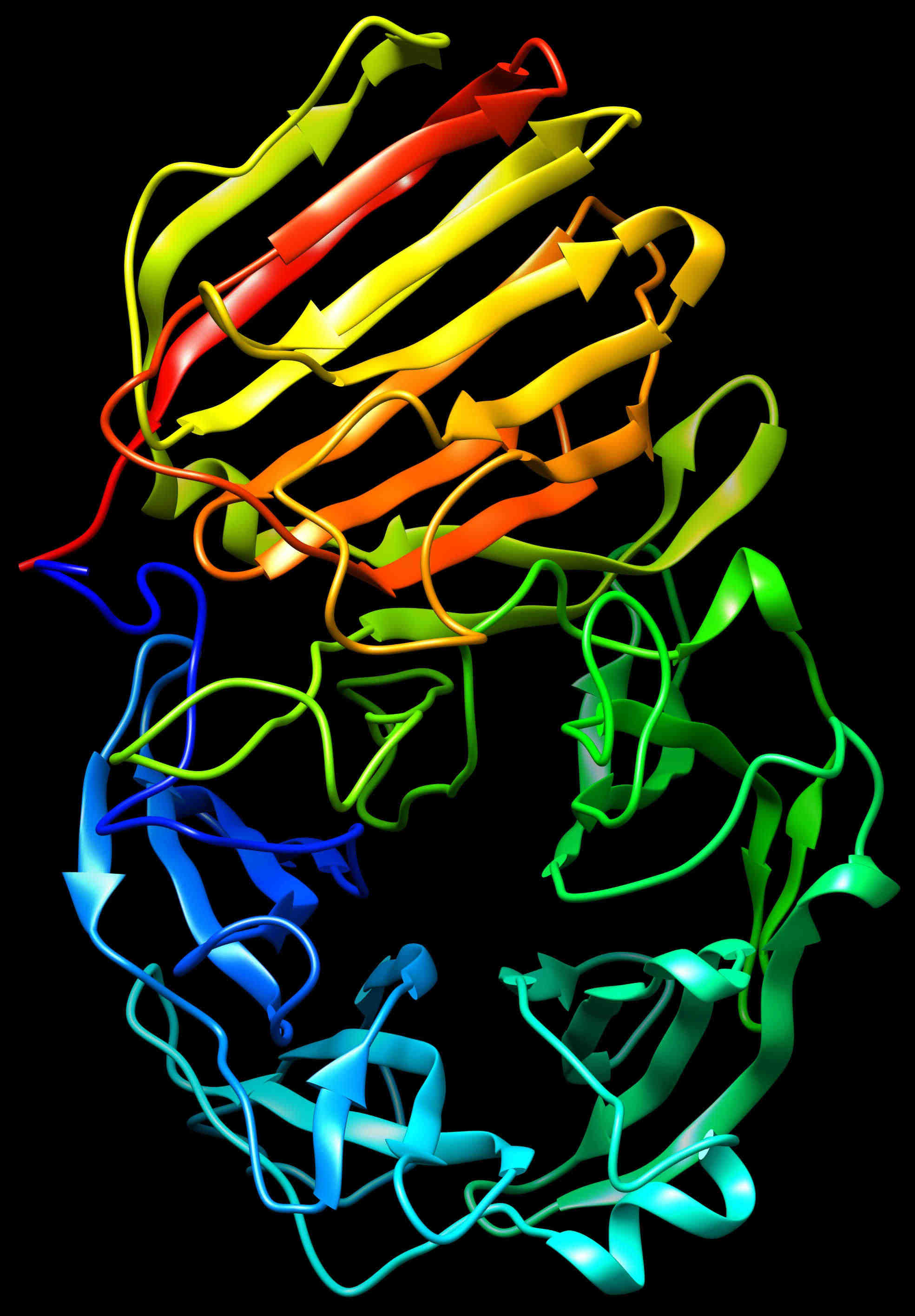

Supplement: S1 Dataset — 3D models were generated from sequences retrieved from the non-redundant protein sequence database using SWISS-MODEL. (ZIP) [file pone.0200607.s001.zip › Homology_Models/Anigerp1m1.jpg]

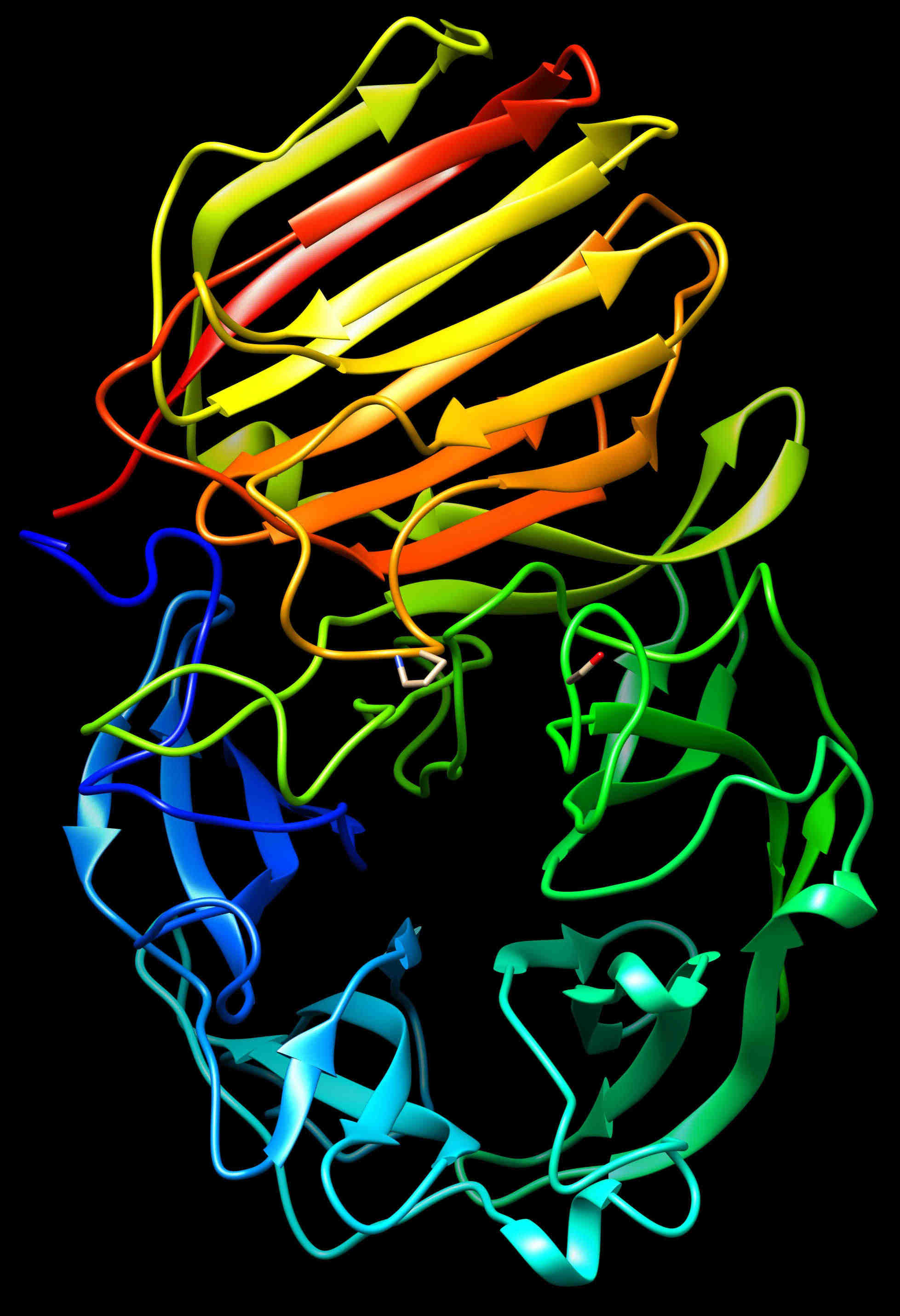

Supplement: S1 Dataset — 3D models were generated from sequences retrieved from the non-redundant protein sequence database using SWISS-MODEL. (ZIP) [file pone.0200607.s001.zip › Homology_Models/Anigerp2m1.jpg]

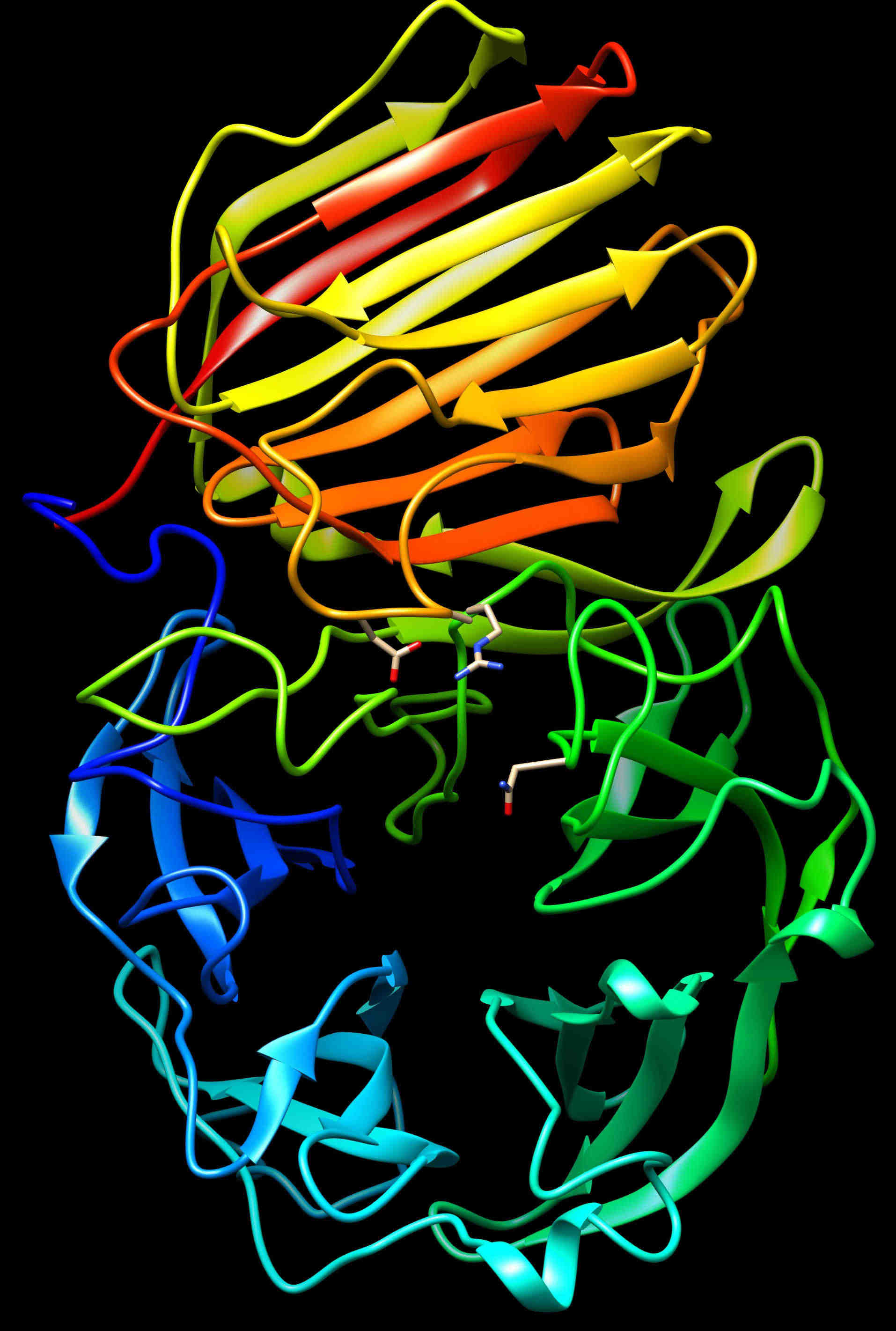

Supplement: S1 Dataset — 3D models were generated from sequences retrieved from the non-redundant protein sequence database using SWISS-MODEL. (ZIP) [file pone.0200607.s001.zip › Homology_Models/Anigerp3m1.jpg]

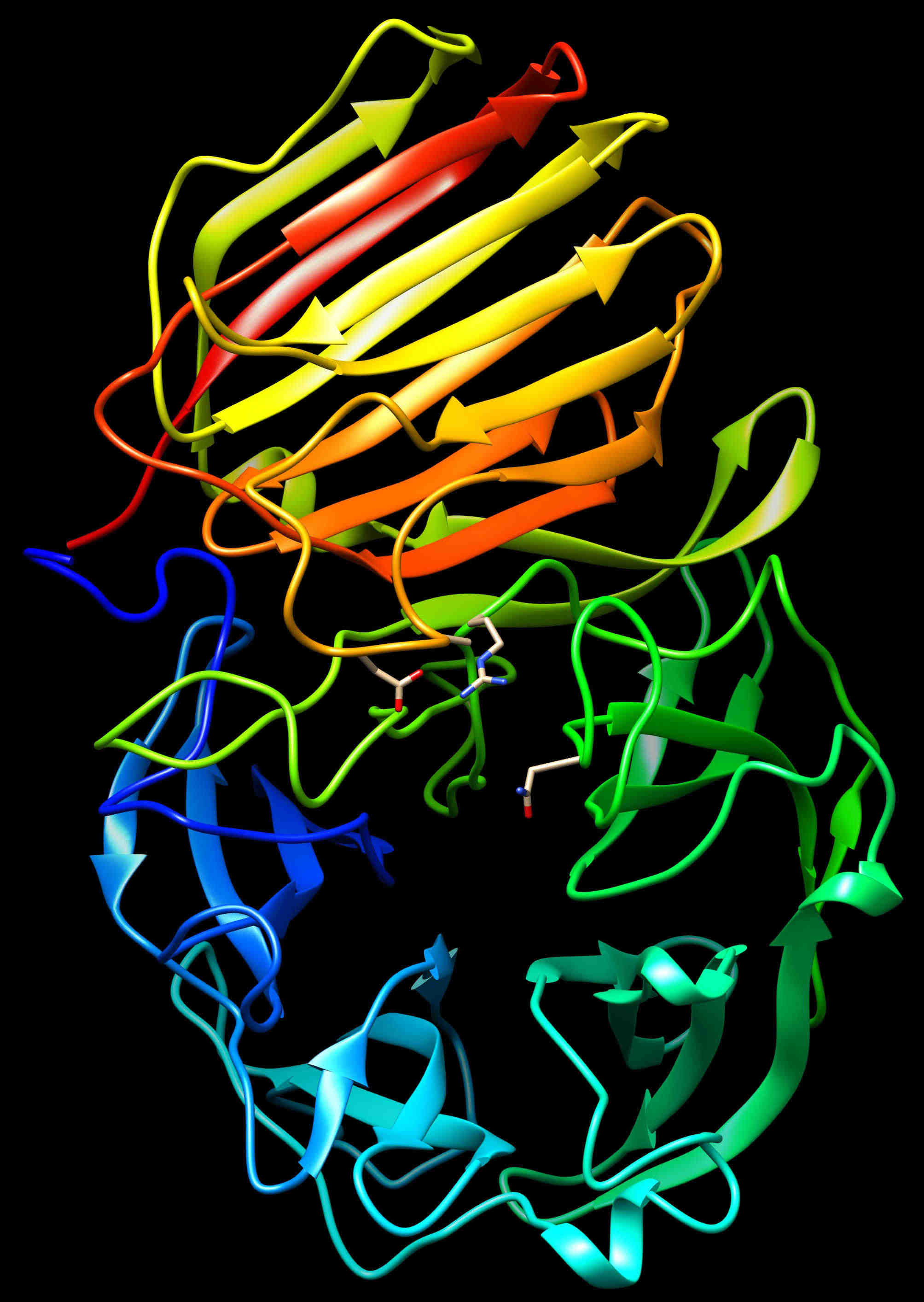

Supplement: S1 Dataset — 3D models were generated from sequences retrieved from the non-redundant protein sequence database using SWISS-MODEL. (ZIP) [file pone.0200607.s001.zip › Homology_Models/Anigerp4m1.jpg]

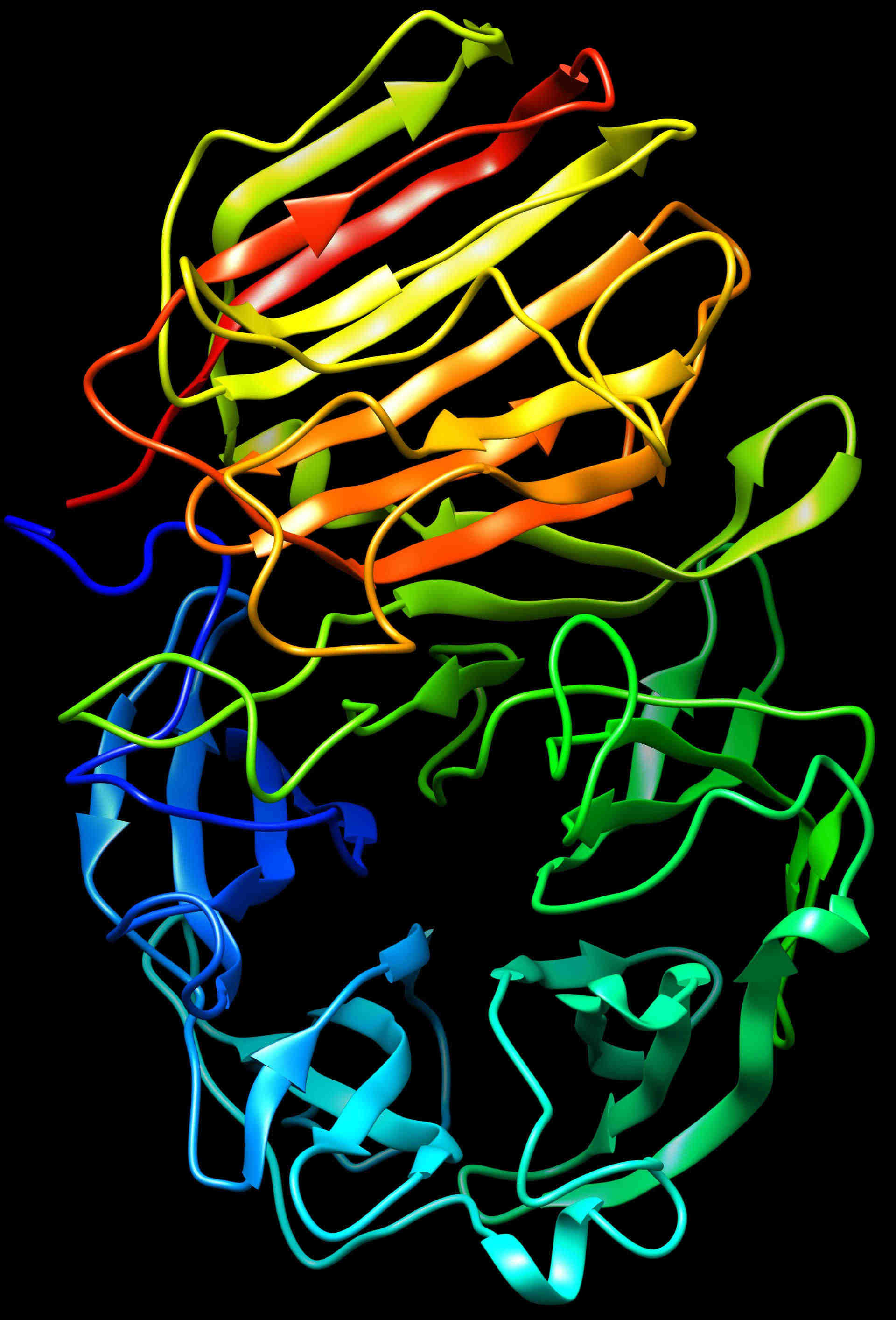

Supplement: S1 Dataset — 3D models were generated from sequences retrieved from the non-redundant protein sequence database using SWISS-MODEL. (ZIP) [file pone.0200607.s001.zip › Homology_Models/Anigerp5m1.jpg]

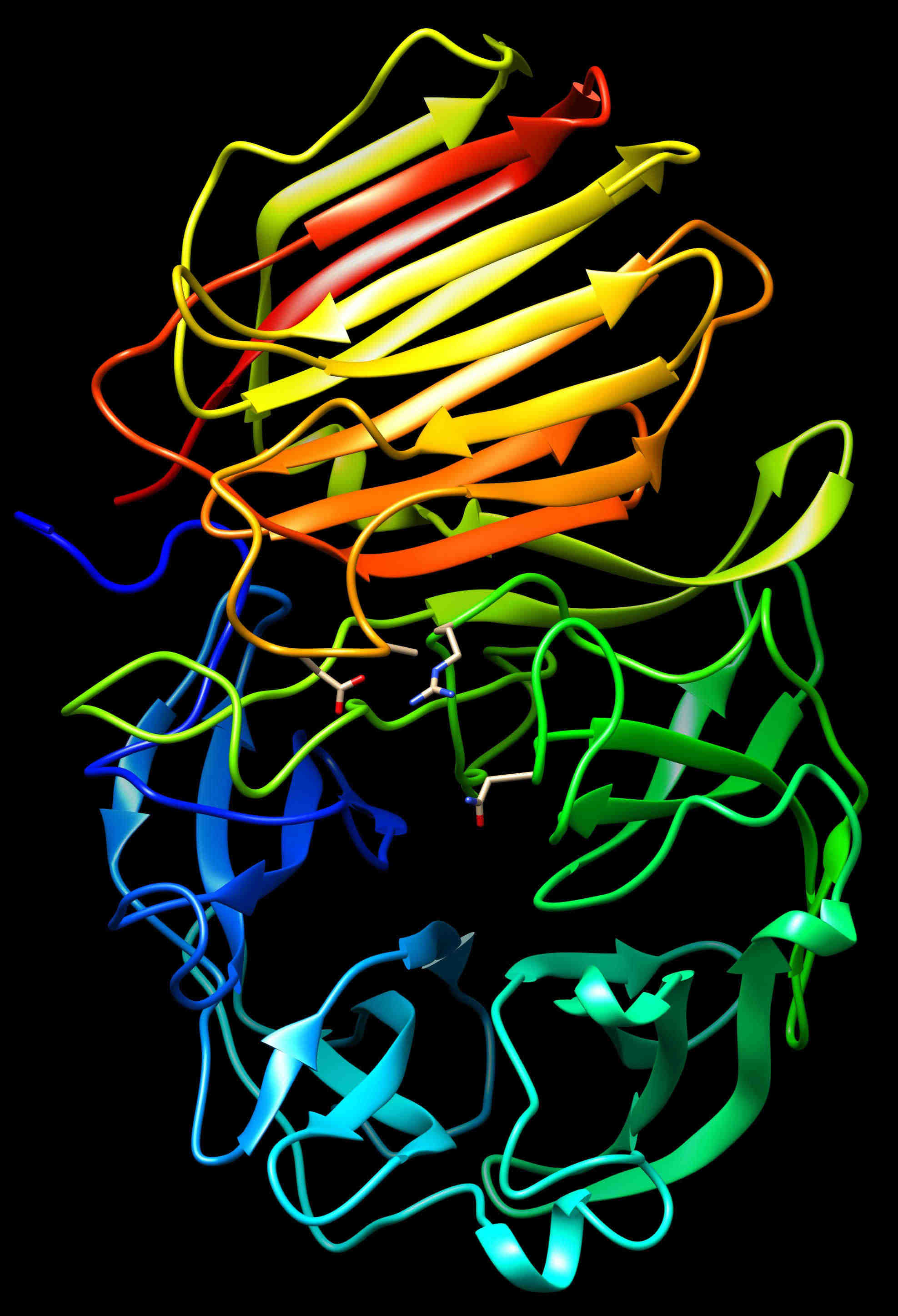

Supplement: S1 Dataset — 3D models were generated from sequences retrieved from the non-redundant protein sequence database using SWISS-MODEL. (ZIP) [file pone.0200607.s001.zip › Homology_Models/Anigerp7m1.jpg]

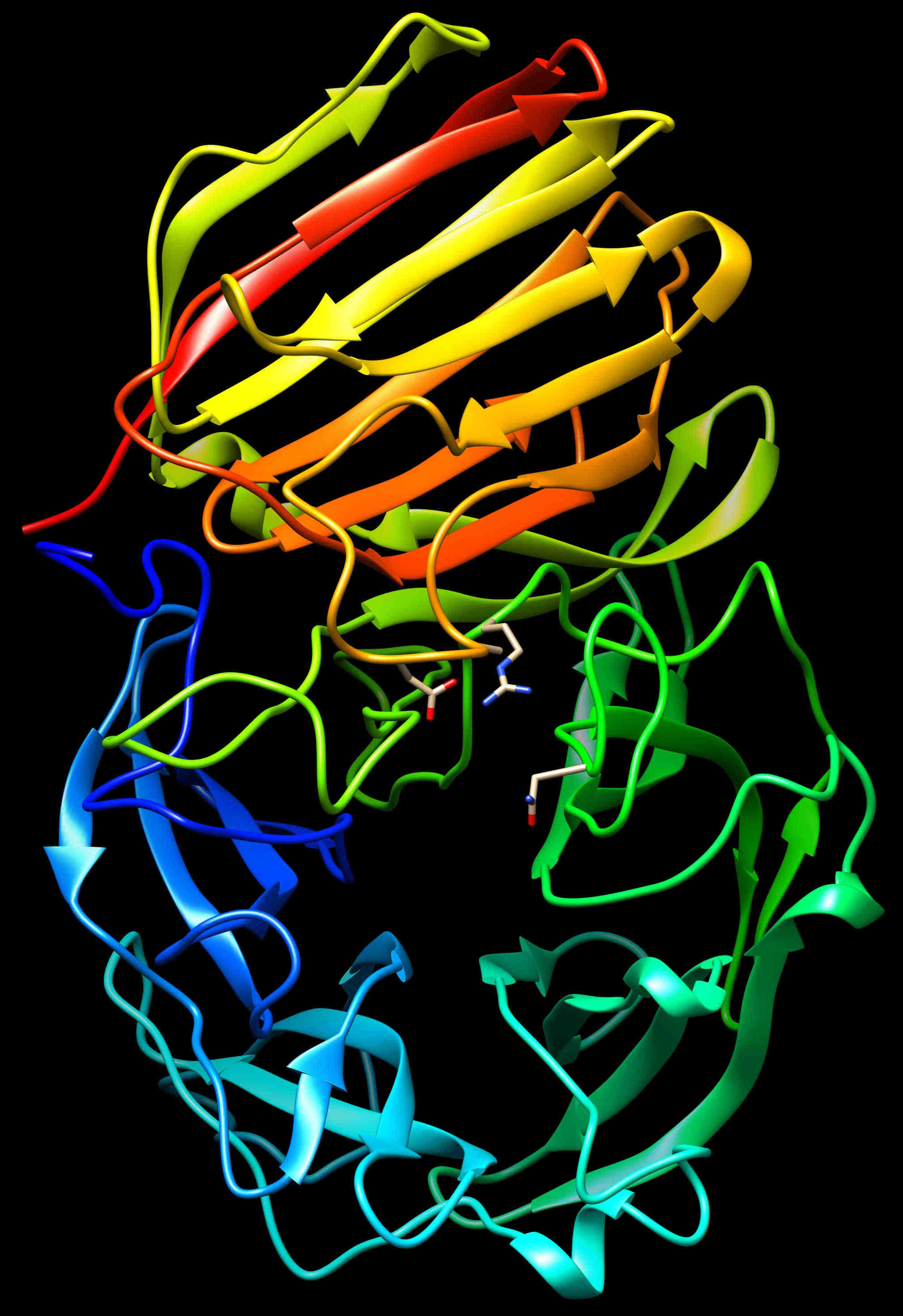

Supplement: S1 Dataset — 3D models were generated from sequences retrieved from the non-redundant protein sequence database using SWISS-MODEL. (ZIP) [file pone.0200607.s001.zip › Homology_Models/Anigerp9m1.jpg]

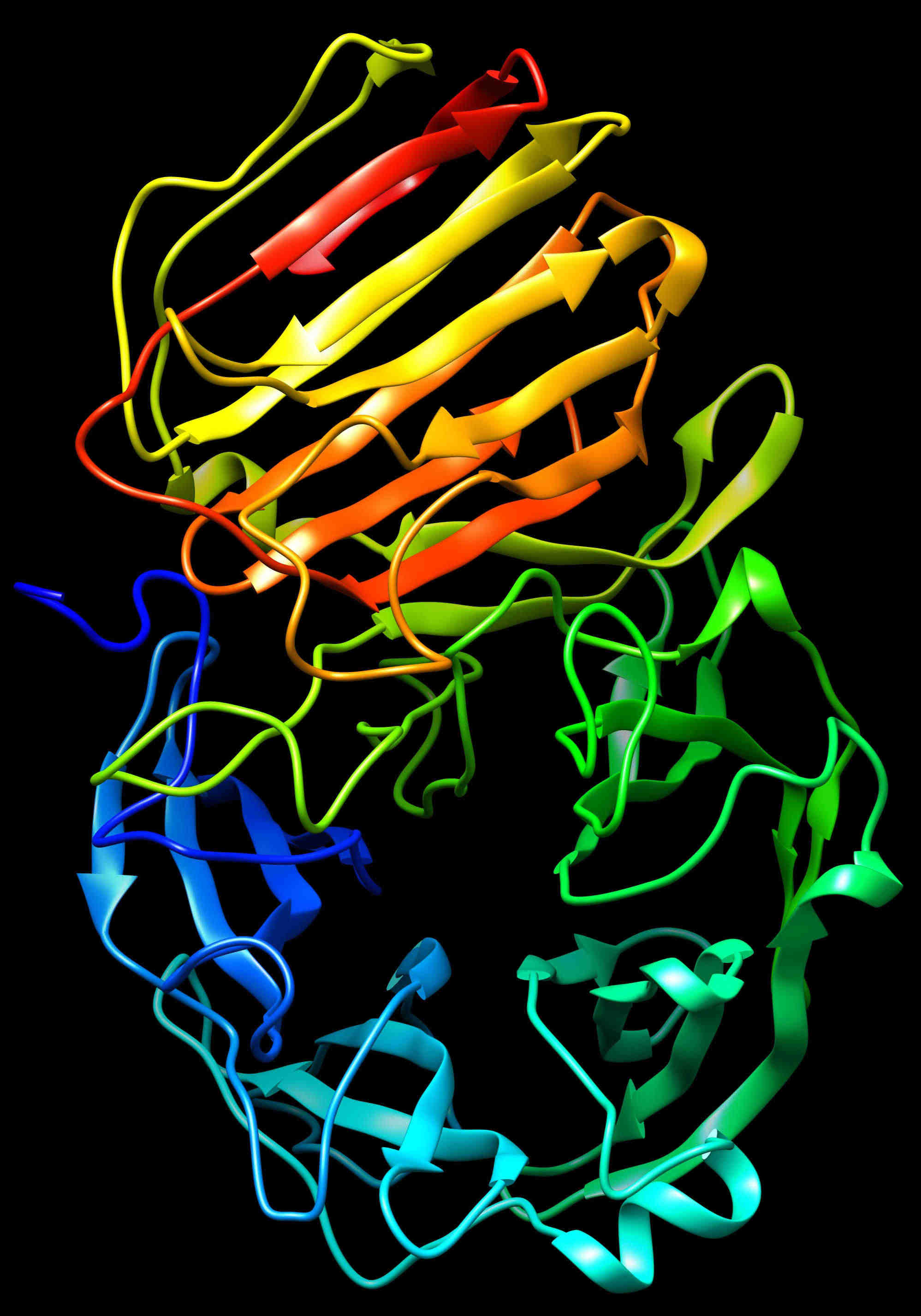

Supplement: S1 Dataset — 3D models were generated from sequences retrieved from the non-redundant protein sequence database using SWISS-MODEL. (ZIP) [file pone.0200607.s001.zip › Homology_Models/Arugulosump1m2.jpg]

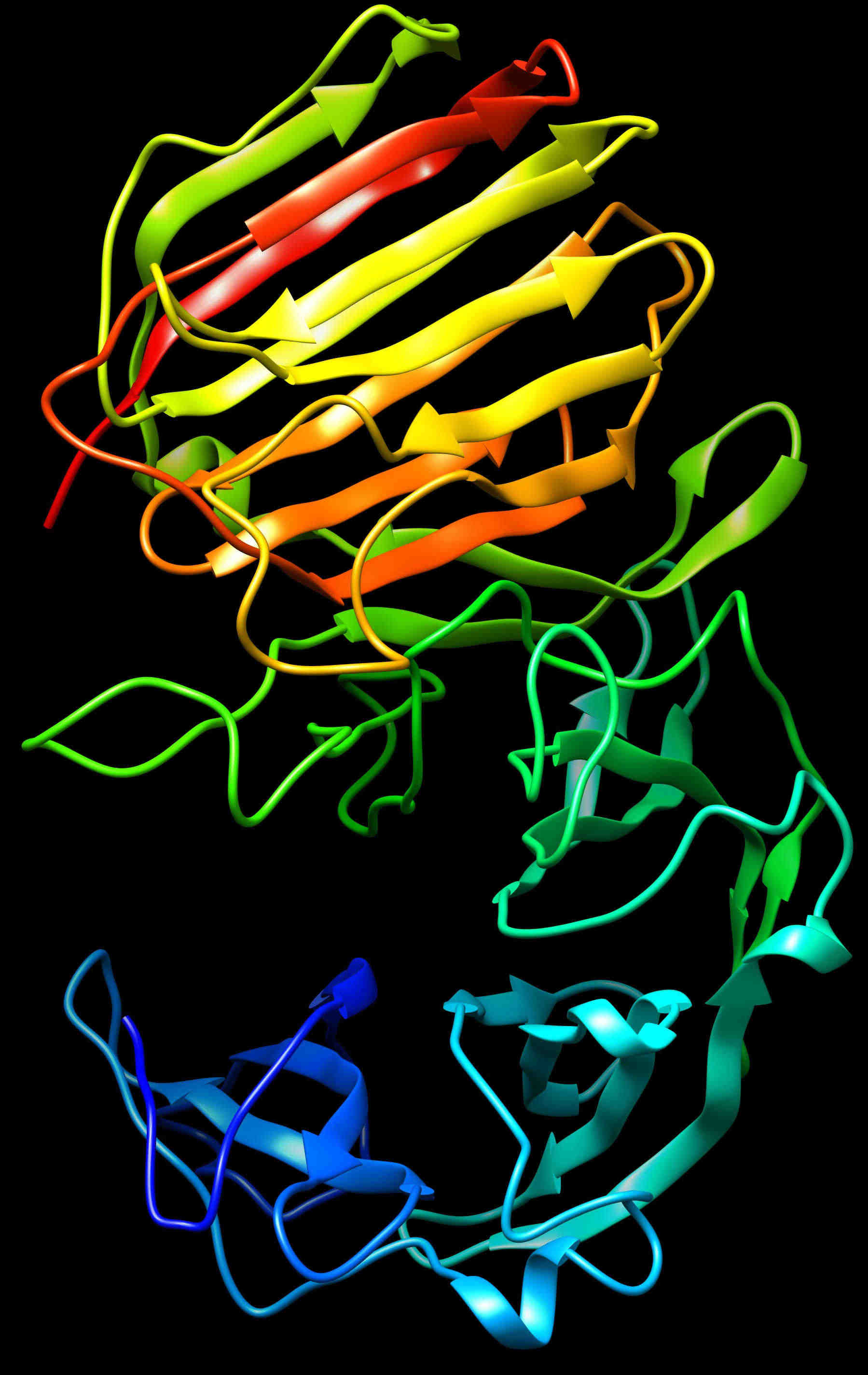

Supplement: S1 Dataset — 3D models were generated from sequences retrieved from the non-redundant protein sequence database using SWISS-MODEL. (ZIP) [file pone.0200607.s001.zip › Homology_Models/Audagawaep1m2.jpg]

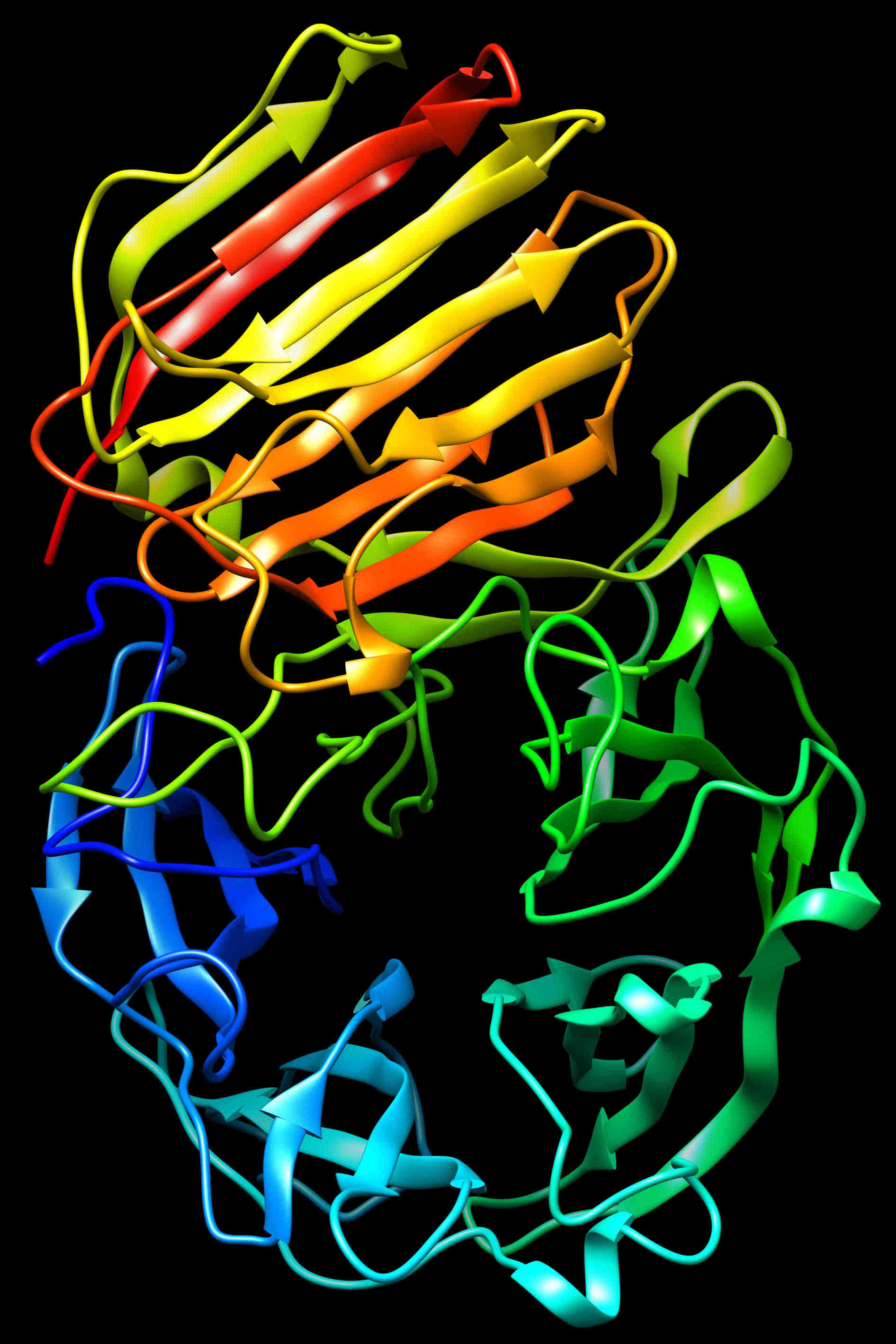

Supplement: S1 Dataset — 3D models were generated from sequences retrieved from the non-redundant protein sequence database using SWISS-MODEL. (ZIP) [file pone.0200607.s001.zip › Homology_Models/Bcenereap1m1.jpg]

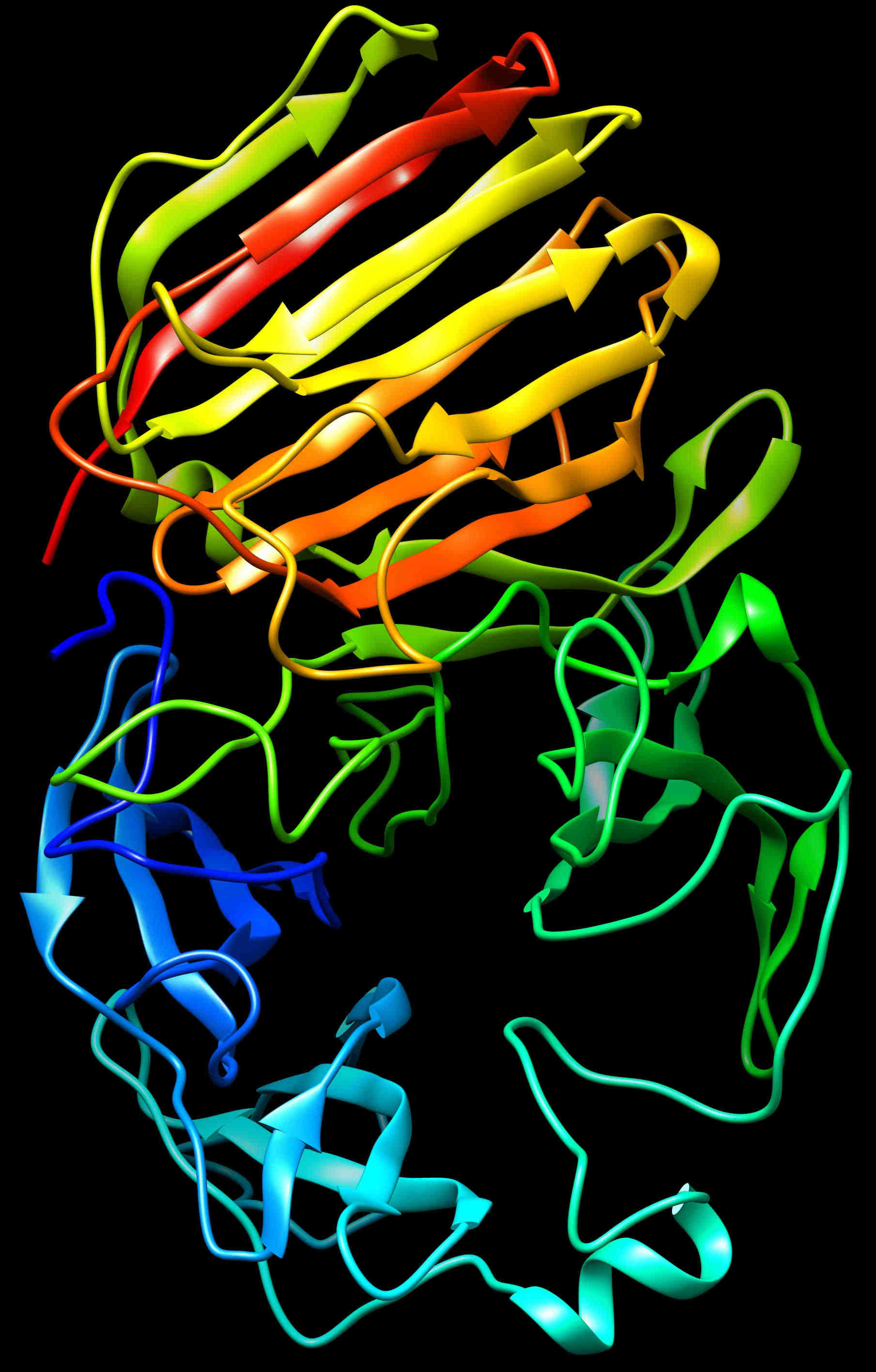

Supplement: S1 Dataset — 3D models were generated from sequences retrieved from the non-redundant protein sequence database using SWISS-MODEL. (ZIP) [file pone.0200607.s001.zip › Homology_Models/Bcenereap2m1.jpg]

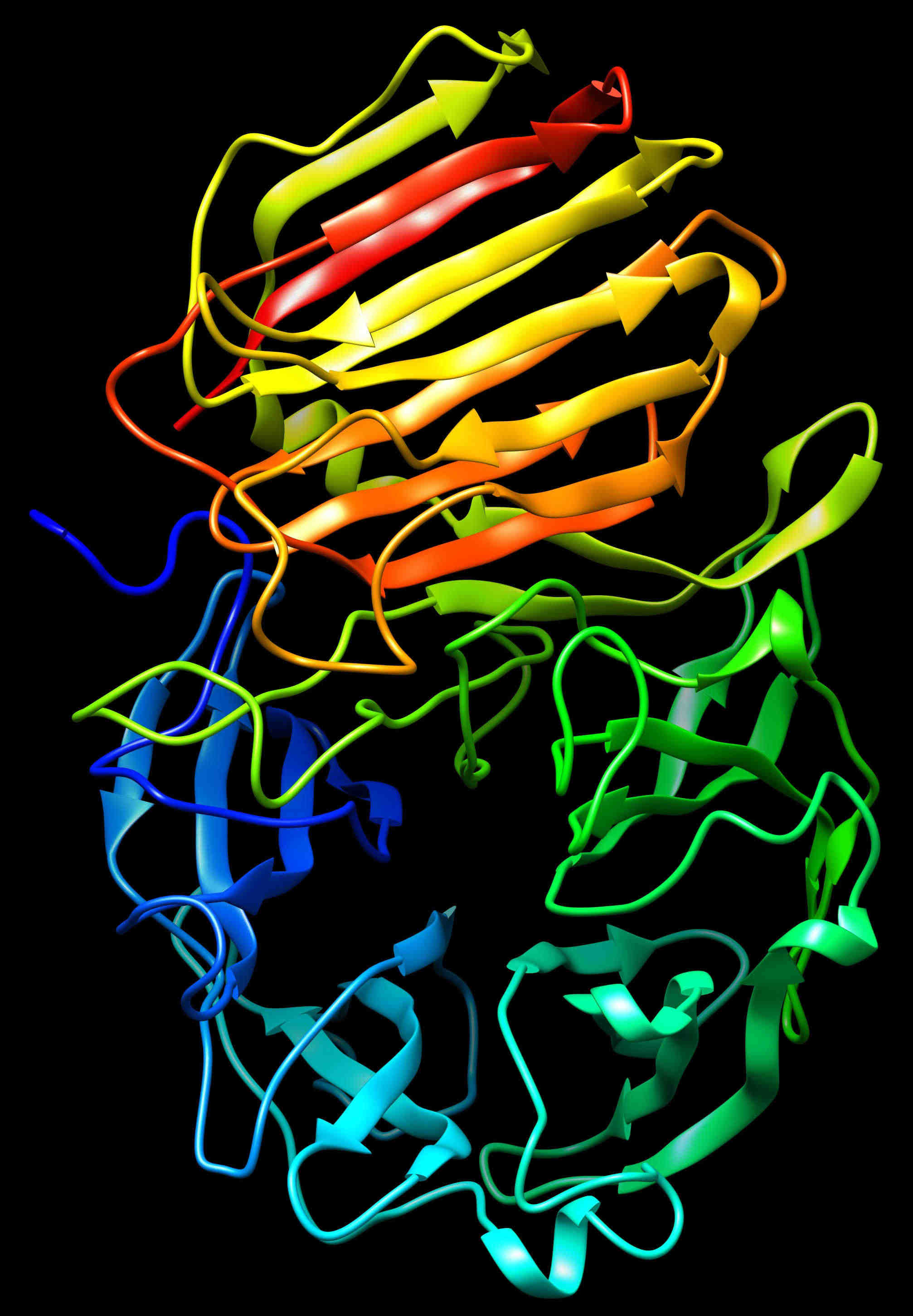

Supplement: S1 Dataset — 3D models were generated from sequences retrieved from the non-redundant protein sequence database using SWISS-MODEL. (ZIP) [file pone.0200607.s001.zip › Homology_Models/Foxysporumhypop1m1.jpg]

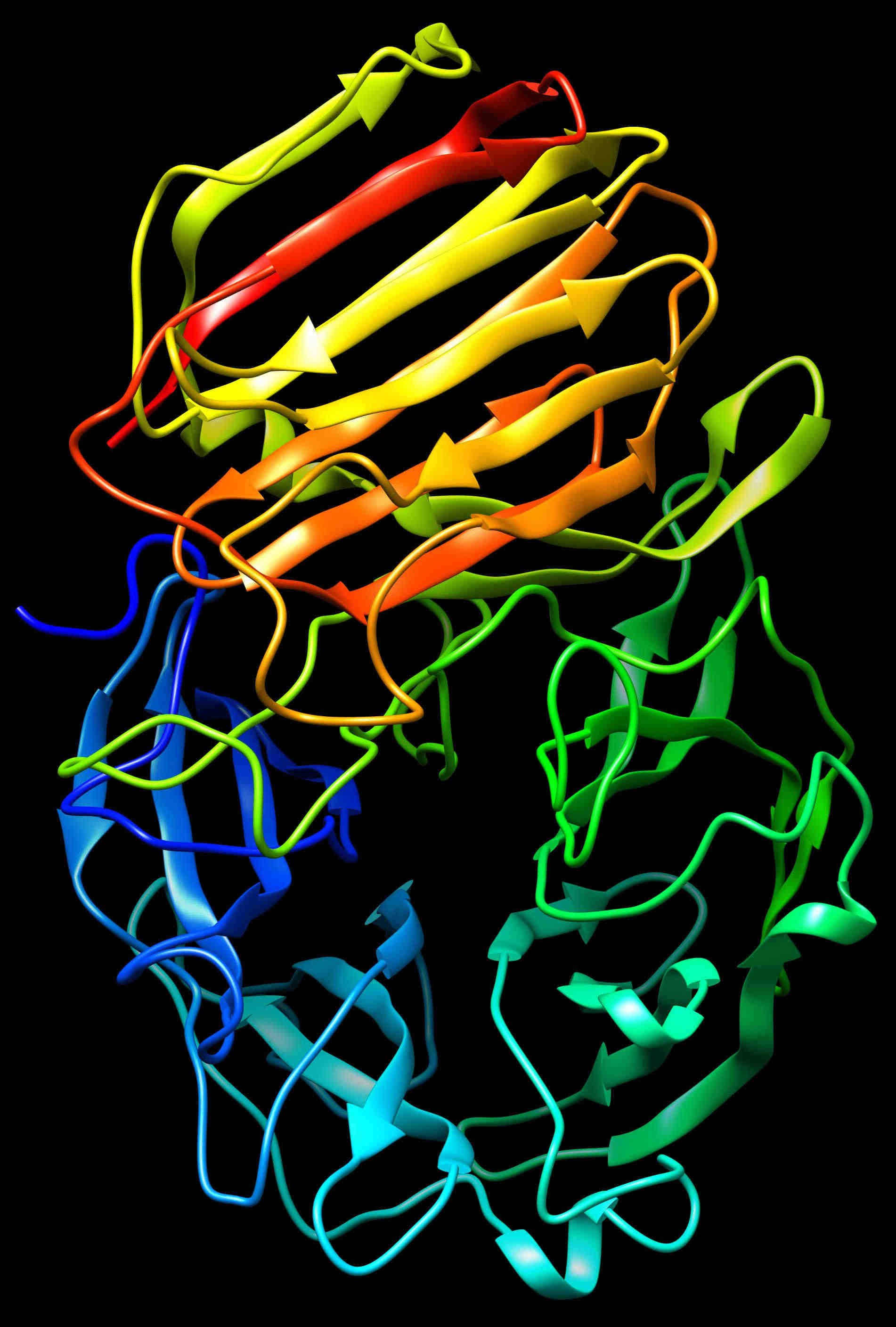

Supplement: S1 Dataset — 3D models were generated from sequences retrieved from the non-redundant protein sequence database using SWISS-MODEL. (ZIP) [file pone.0200607.s001.zip › Homology_Models/Foxysporumhypop7m1.jpg]

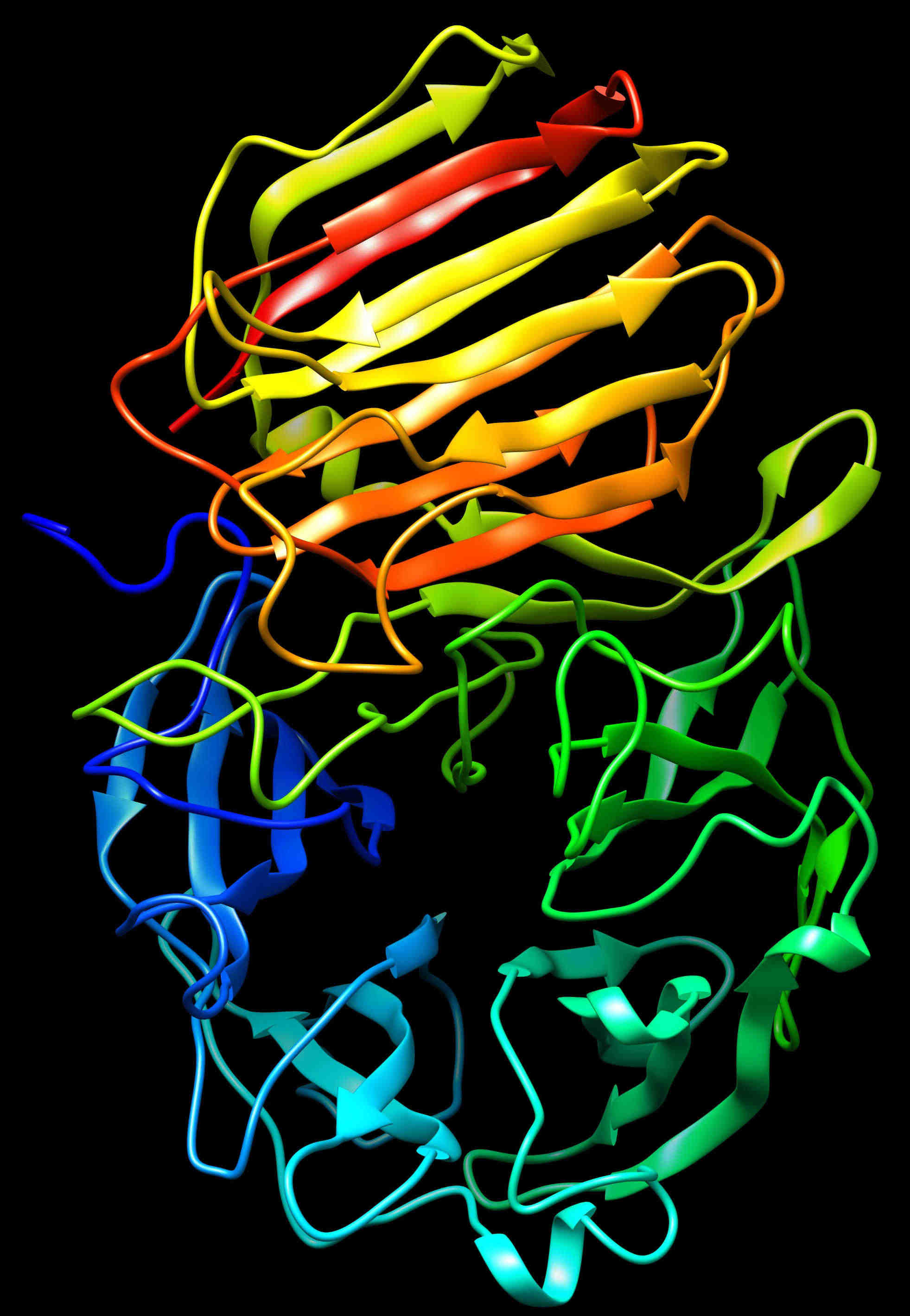

Supplement: S1 Dataset — 3D models were generated from sequences retrieved from the non-redundant protein sequence database using SWISS-MODEL. (ZIP) [file pone.0200607.s001.zip › Homology_Models/Foxysporuminup10m1.jpg]

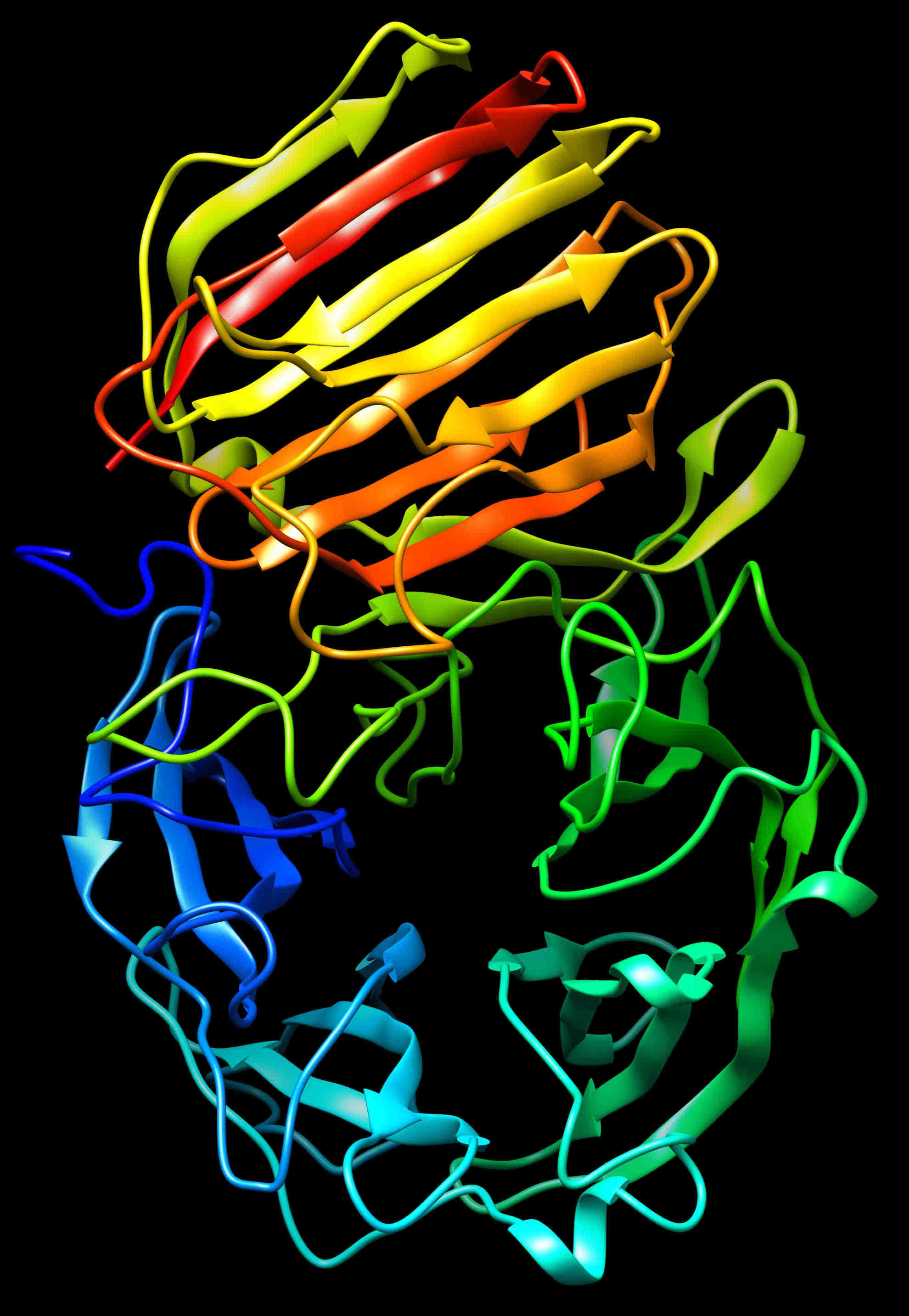

Supplement: S1 Dataset — 3D models were generated from sequences retrieved from the non-redundant protein sequence database using SWISS-MODEL. (ZIP) [file pone.0200607.s001.zip › Homology_Models/Foxysporuminup11m1.jpg]

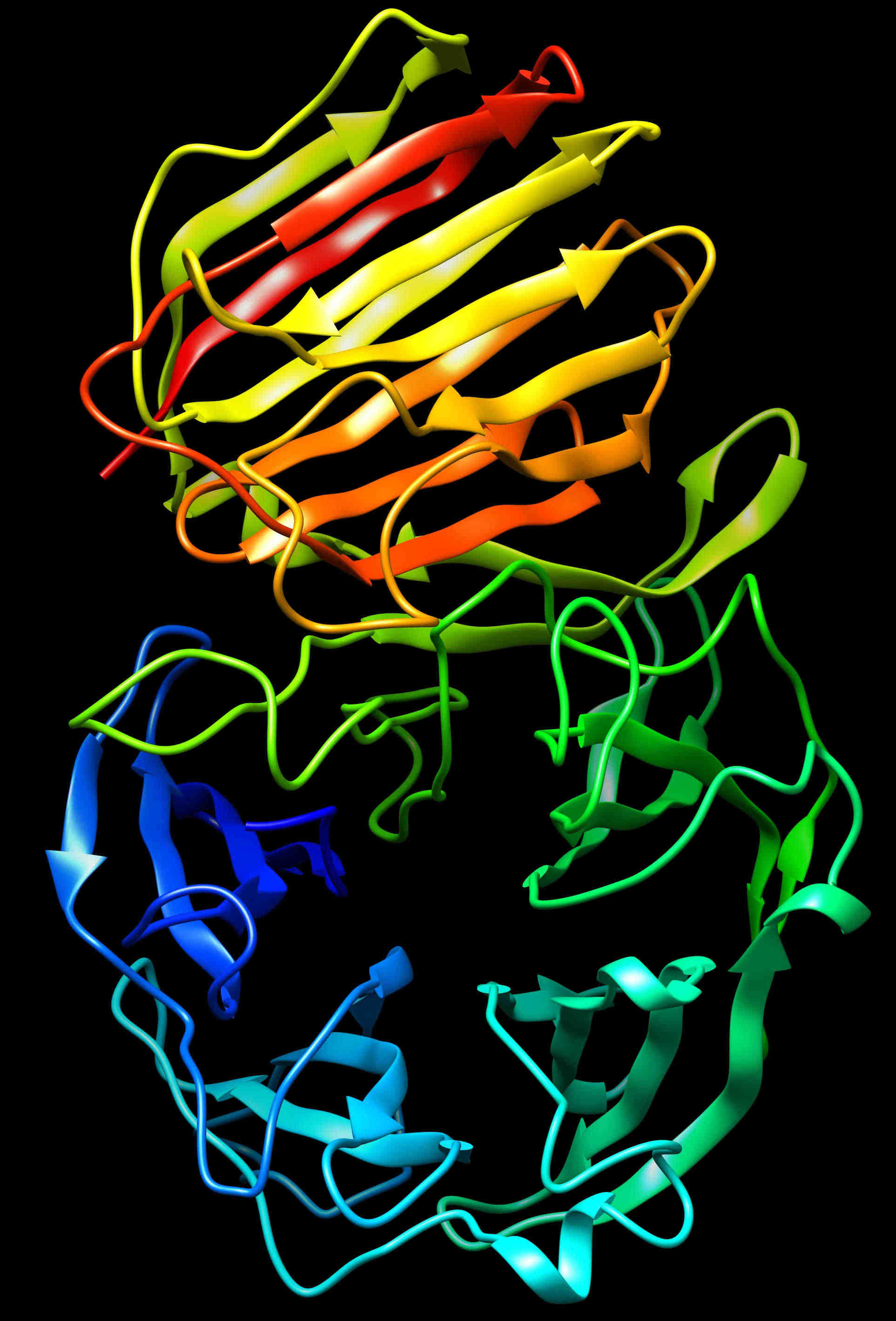

Supplement: S1 Dataset — 3D models were generated from sequences retrieved from the non-redundant protein sequence database using SWISS-MODEL. (ZIP) [file pone.0200607.s001.zip › Homology_Models/Foxysporuminup12m1.jpg]

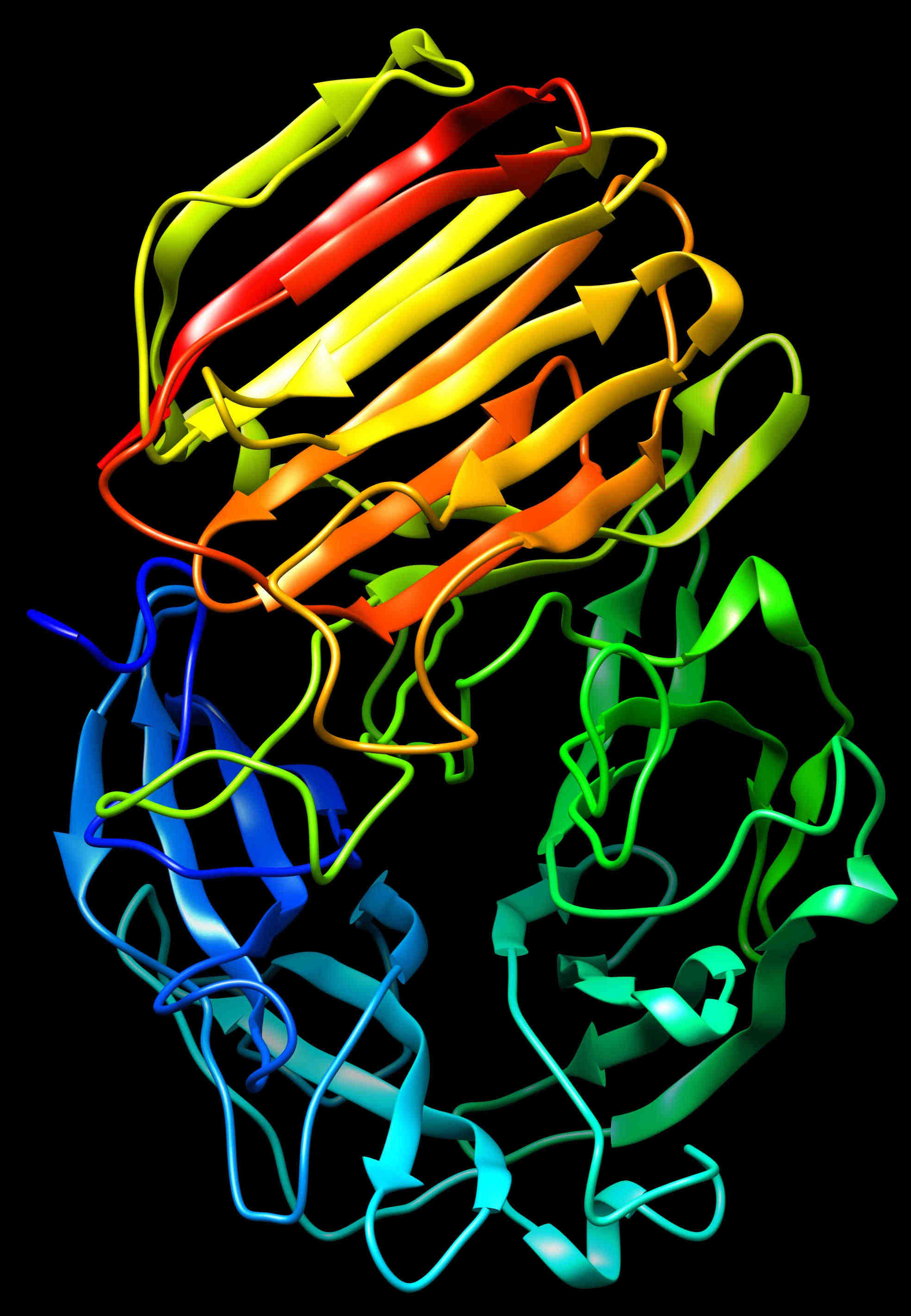

Supplement: S1 Dataset — 3D models were generated from sequences retrieved from the non-redundant protein sequence database using SWISS-MODEL. (ZIP) [file pone.0200607.s001.zip › Homology_Models/Foxysporuminup1m1.jpg]

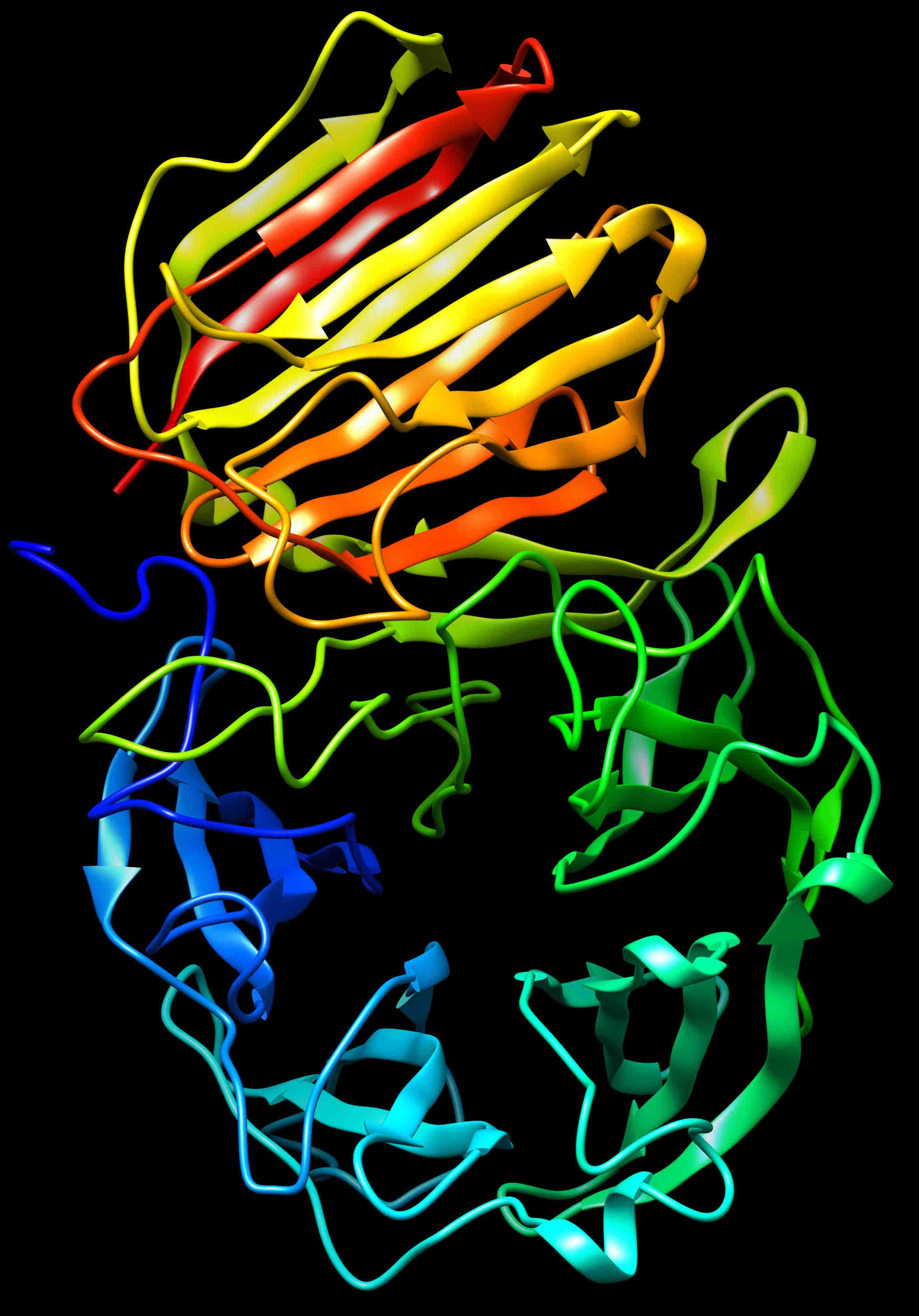

Supplement: S1 Dataset — 3D models were generated from sequences retrieved from the non-redundant protein sequence database using SWISS-MODEL. (ZIP) [file pone.0200607.s001.zip › Homology_Models/Foxysporuminup2m1.jpg]

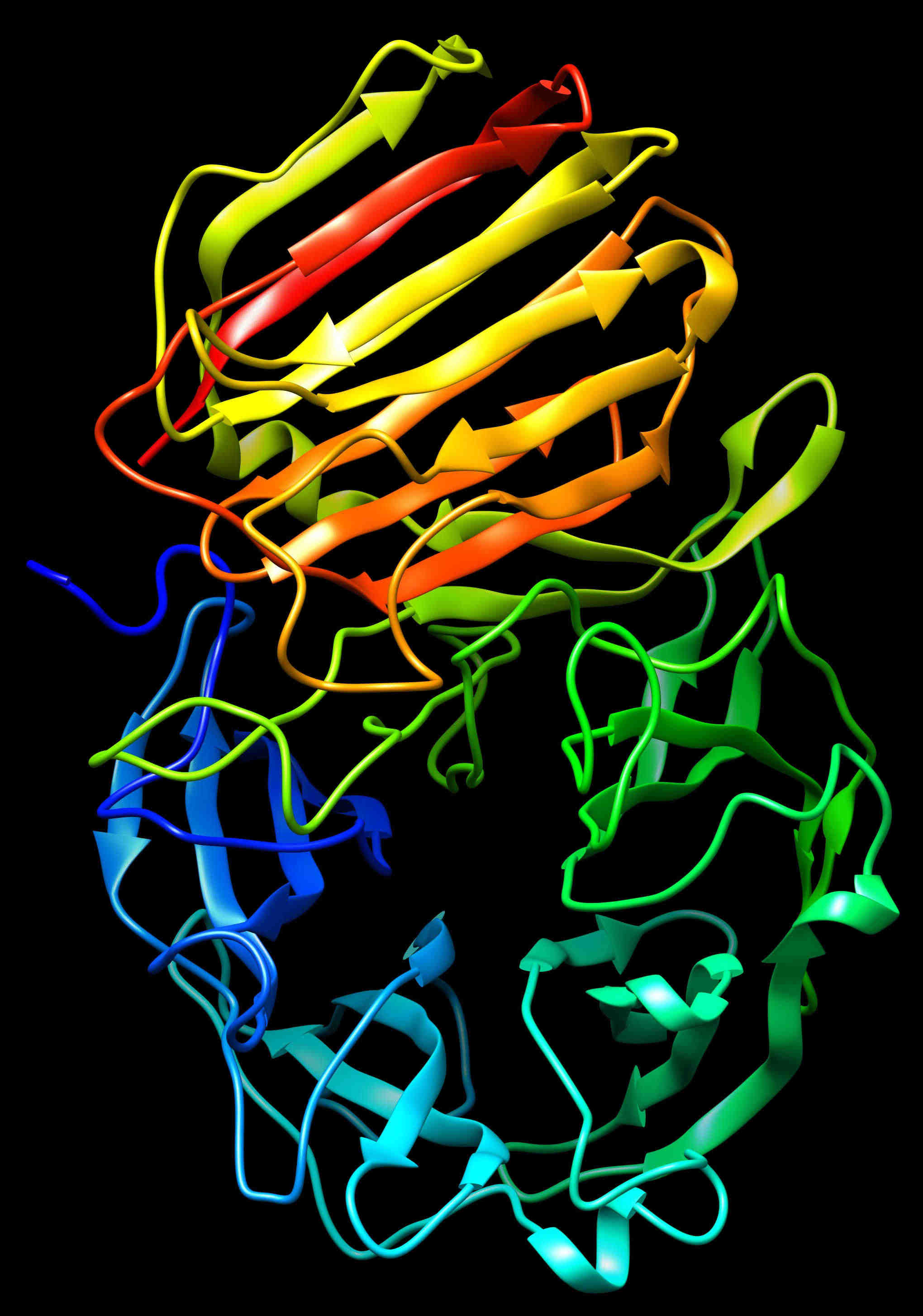

Supplement: S1 Dataset — 3D models were generated from sequences retrieved from the non-redundant protein sequence database using SWISS-MODEL. (ZIP) [file pone.0200607.s001.zip › Homology_Models/Foxysporuminup3m1.jpg]

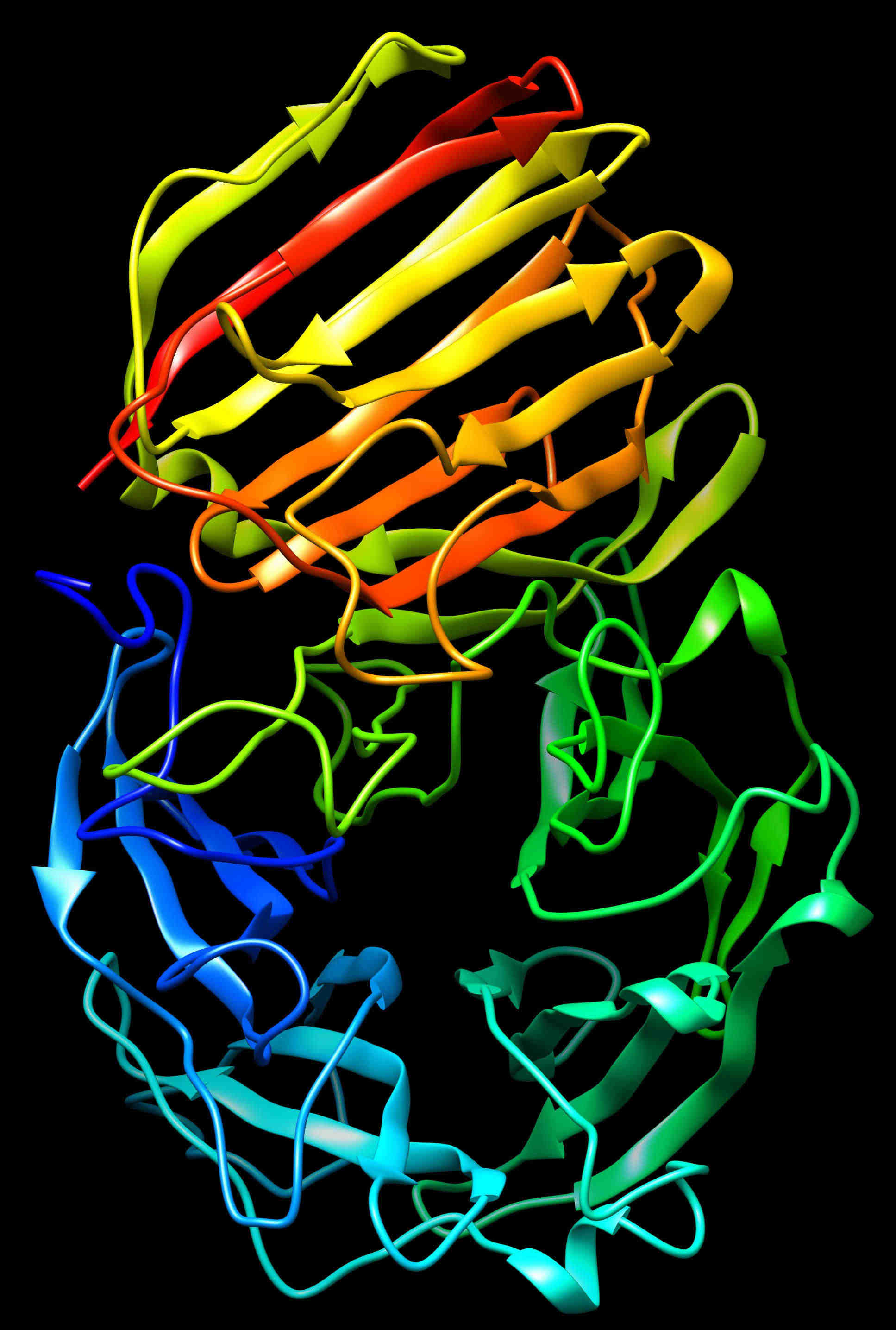

Supplement: S1 Dataset — 3D models were generated from sequences retrieved from the non-redundant protein sequence database using SWISS-MODEL. (ZIP) [file pone.0200607.s001.zip › Homology_Models/Foxysporuminup4m1.jpg]

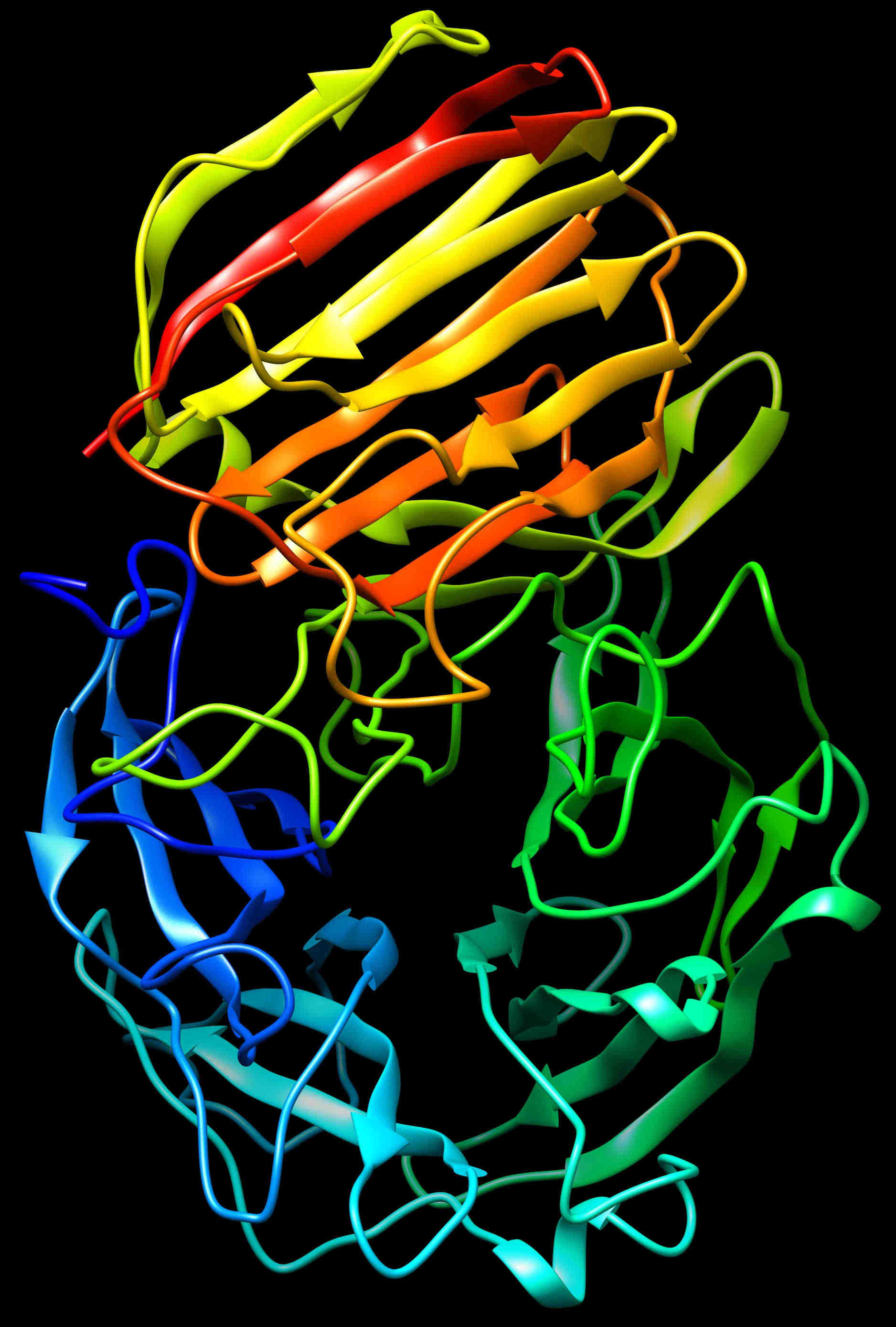

Supplement: S1 Dataset — 3D models were generated from sequences retrieved from the non-redundant protein sequence database using SWISS-MODEL. (ZIP) [file pone.0200607.s001.zip › Homology_Models/Foxysporuminup8m1.jpg]

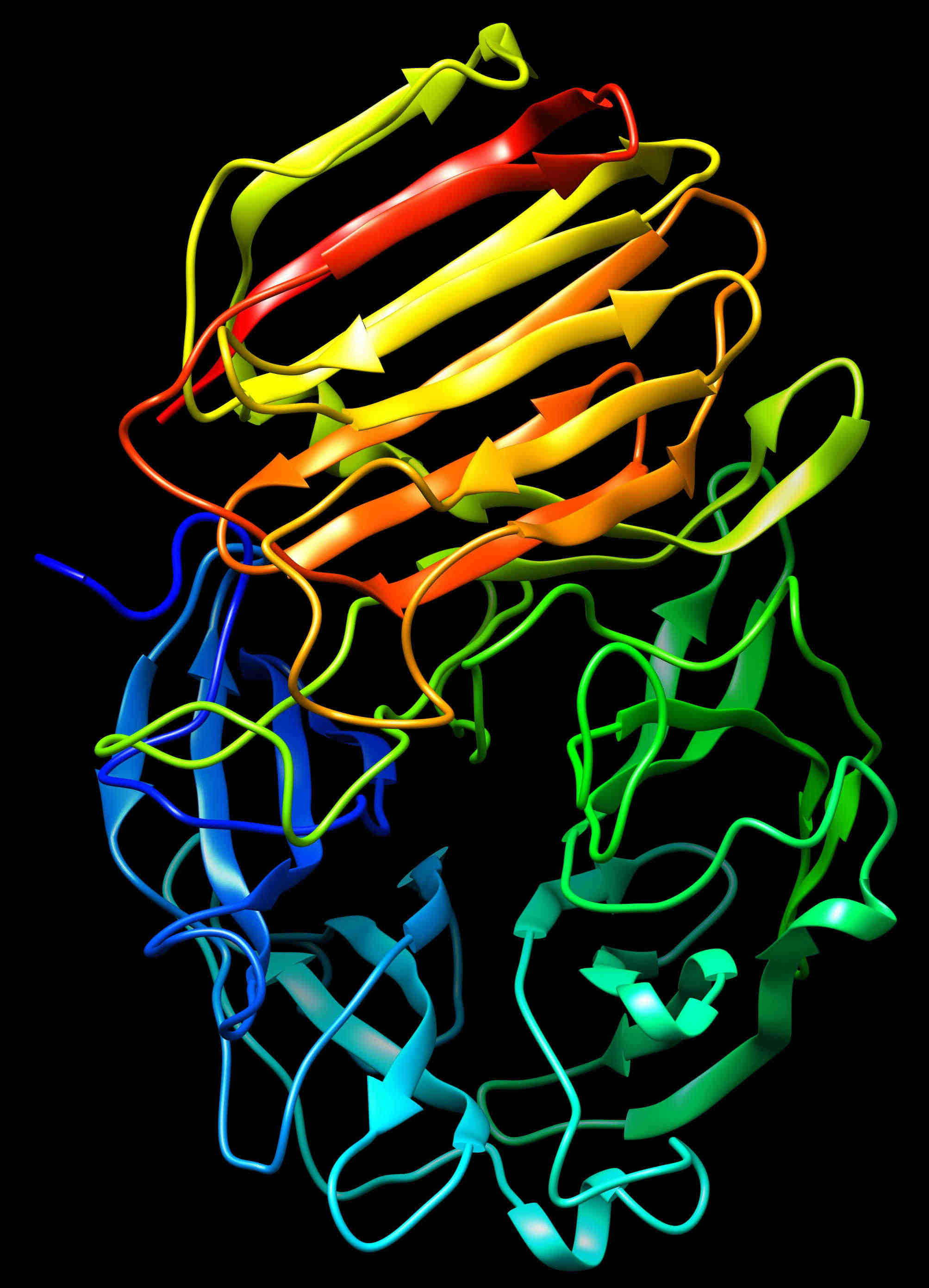

Supplement: S1 Dataset — 3D models were generated from sequences retrieved from the non-redundant protein sequence database using SWISS-MODEL. (ZIP) [file pone.0200607.s001.zip › Homology_Models/Foxysporuminup9m1.jpg]

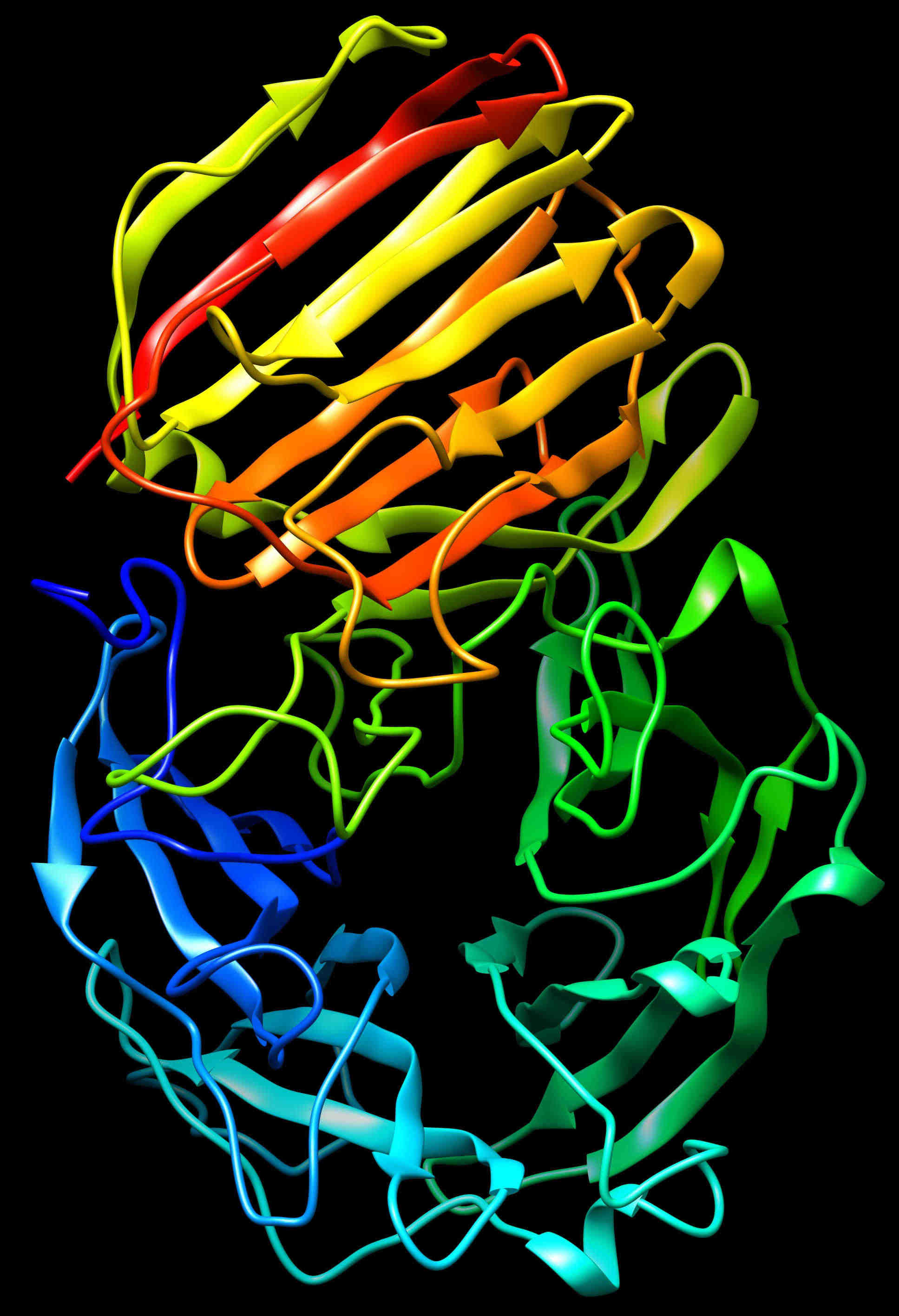

Supplement: S1 Dataset — 3D models were generated from sequences retrieved from the non-redundant protein sequence database using SWISS-MODEL. (ZIP) [file pone.0200607.s001.zip › Homology_Models/Foxyspruminup5m1.jpg]

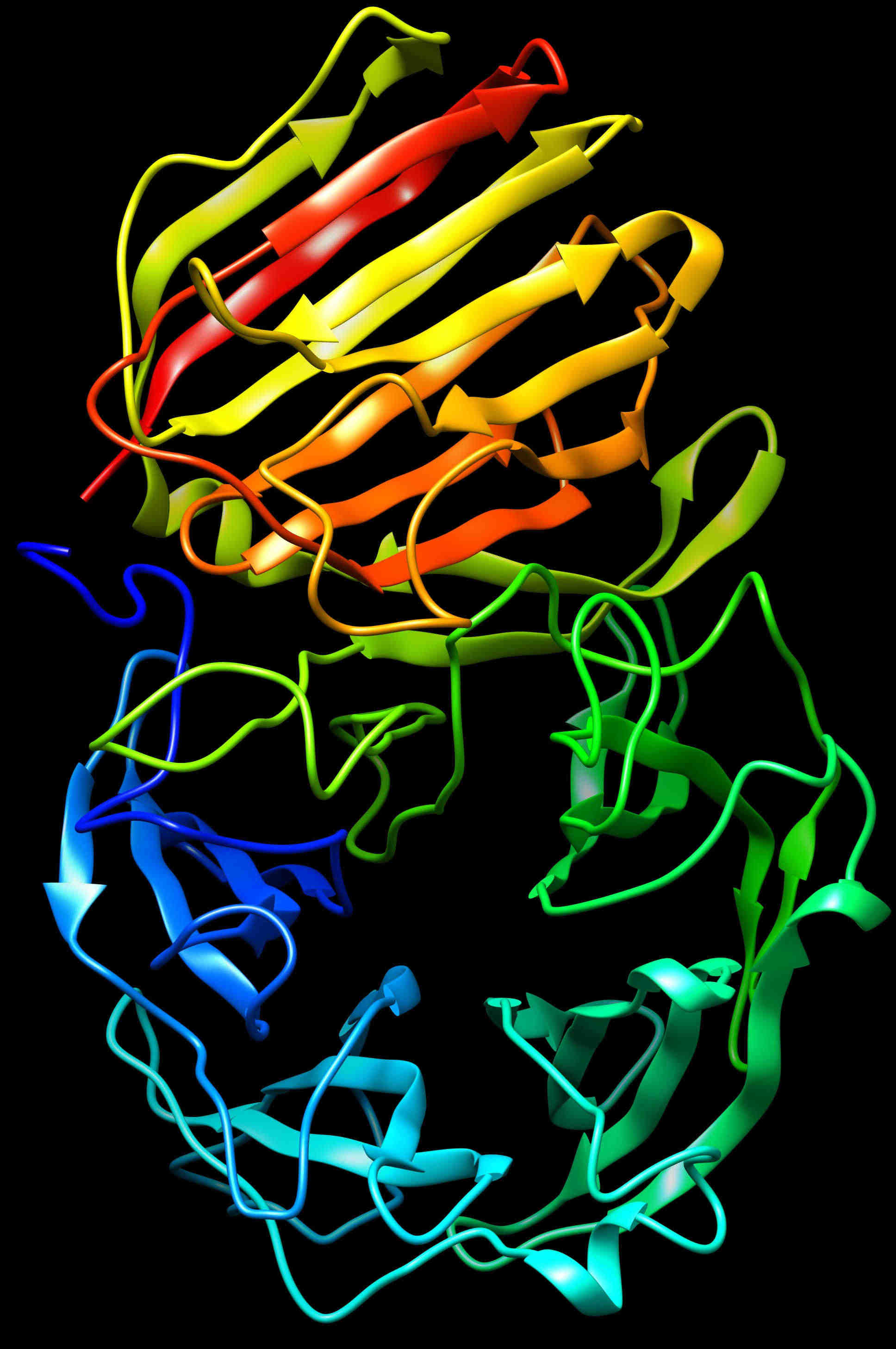

Supplement: S1 Dataset — 3D models were generated from sequences retrieved from the non-redundant protein sequence database using SWISS-MODEL. (ZIP) [file pone.0200607.s001.zip › Homology_Models/Foxyspruminup6m1.jpg]

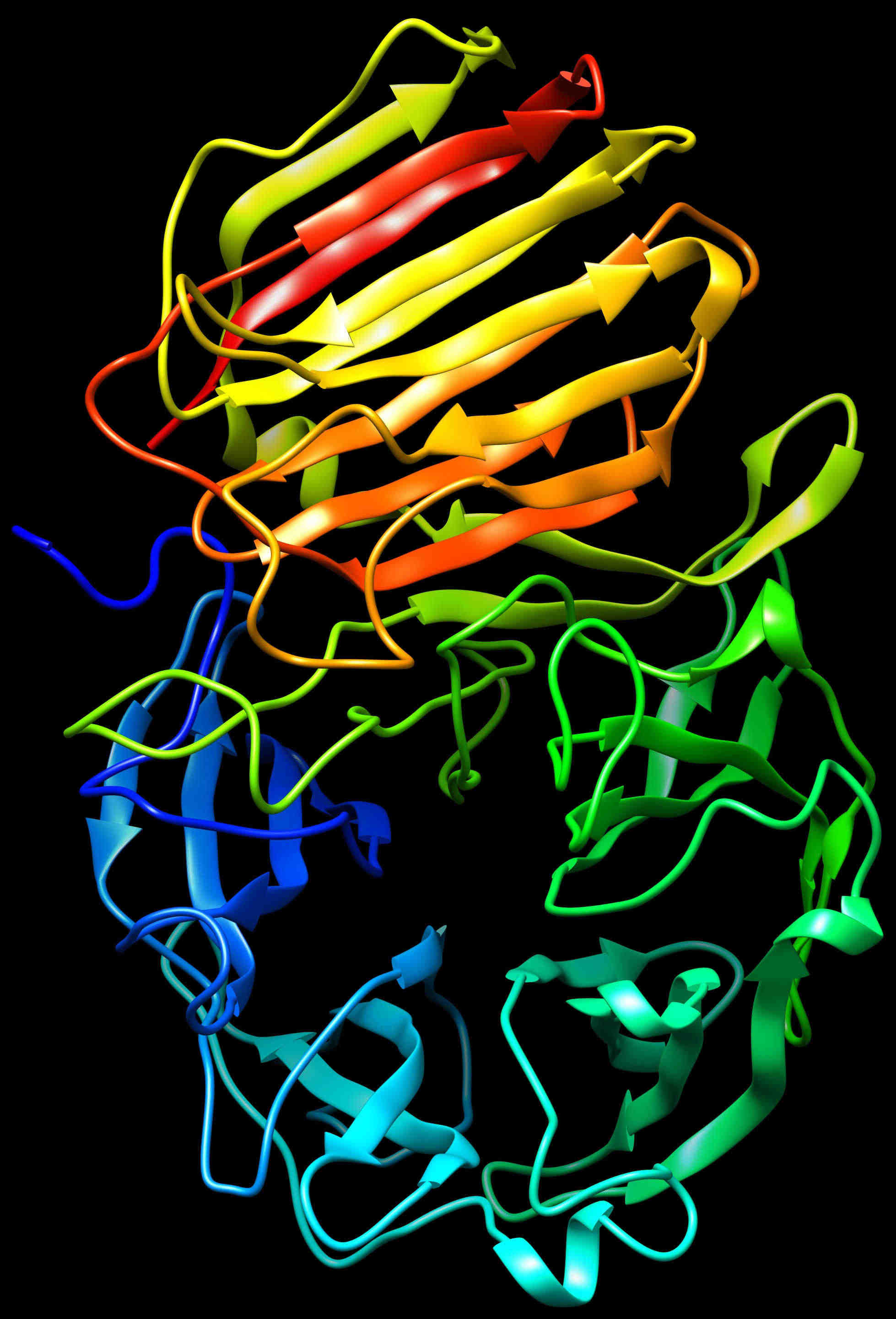

Supplement: S1 Dataset — 3D models were generated from sequences retrieved from the non-redundant protein sequence database using SWISS-MODEL. (ZIP) [file pone.0200607.s001.zip › Homology_Models/Foxyspruminup7m1.jpg]

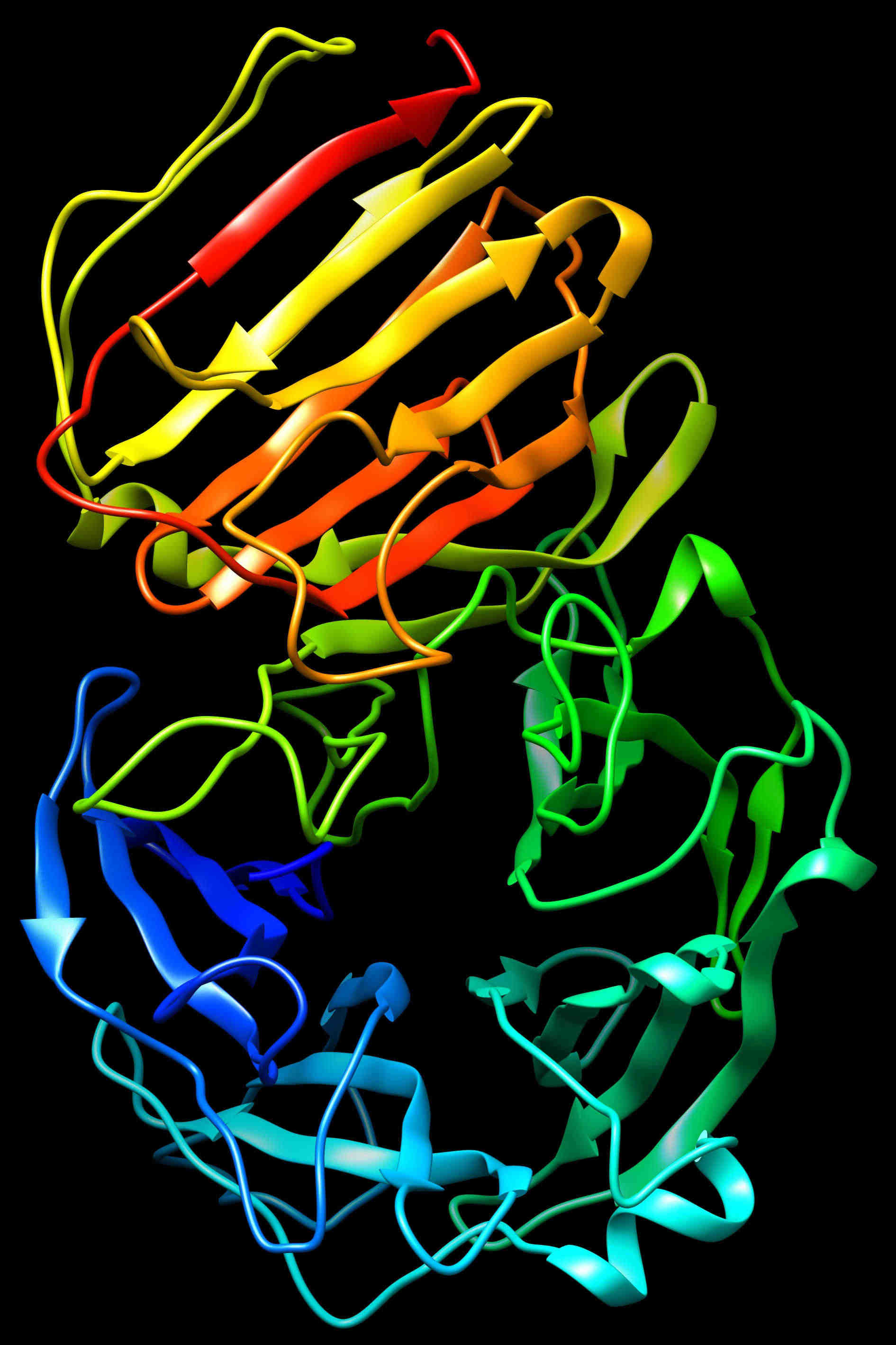

Supplement: S1 Dataset — 3D models were generated from sequences retrieved from the non-redundant protein sequence database using SWISS-MODEL. (ZIP) [file pone.0200607.s001.zip › Homology_Models/Mphaseolinap1m1.jpg]

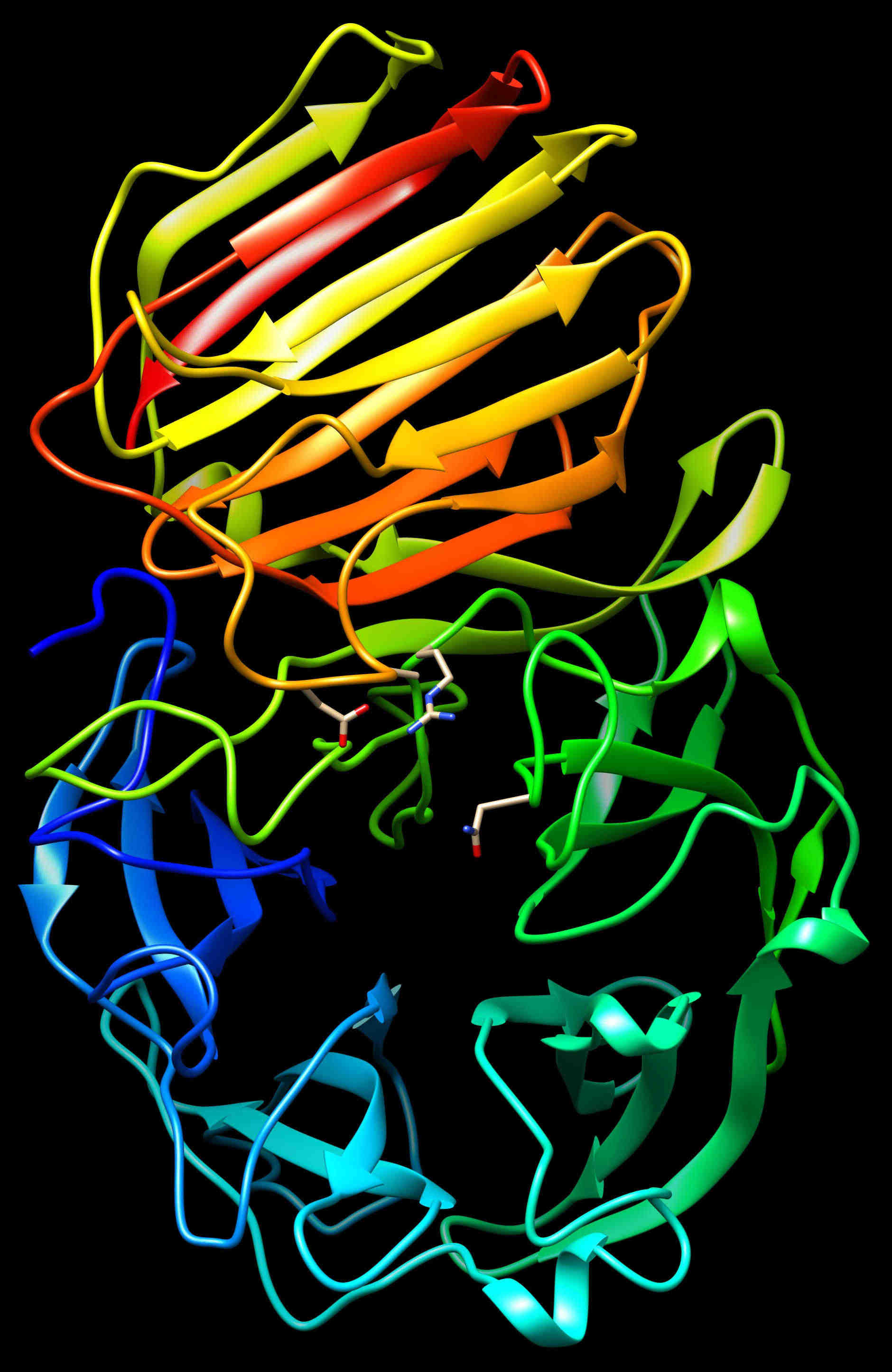

Supplement: S1 Dataset — 3D models were generated from sequences retrieved from the non-redundant protein sequence database using SWISS-MODEL. (ZIP) [file pone.0200607.s001.zip › Homology_Models/Omaiusp1m1.jpg]

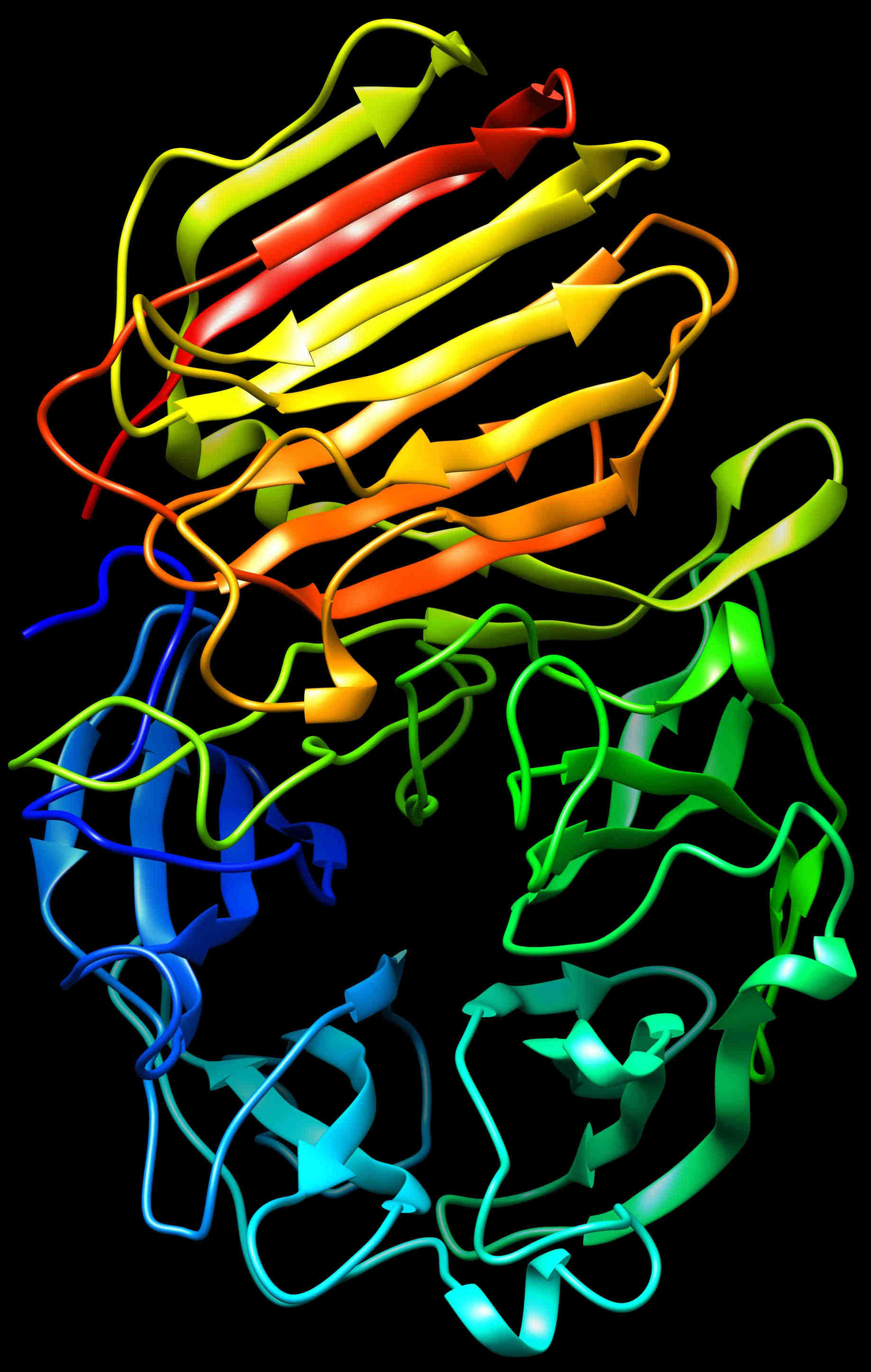

Supplement: S1 Dataset — 3D models were generated from sequences retrieved from the non-redundant protein sequence database using SWISS-MODEL. (ZIP) [file pone.0200607.s001.zip › Homology_Models/Pbrasilianp1m2.jpg]

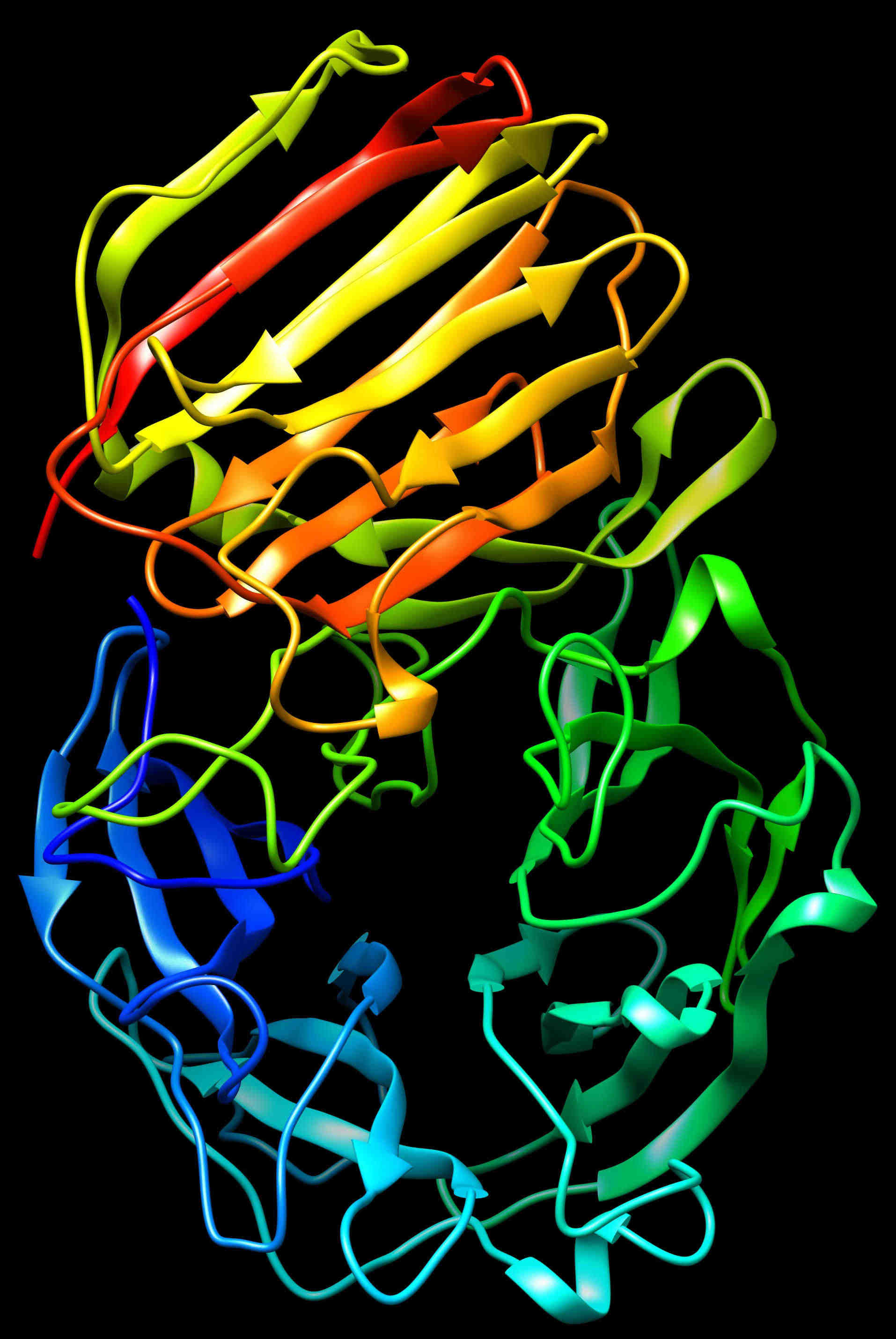

Supplement: S1 Dataset — 3D models were generated from sequences retrieved from the non-redundant protein sequence database using SWISS-MODEL. (ZIP) [file pone.0200607.s001.zip › Homology_Models/Pseudogymnoascusp1m2.jpg]

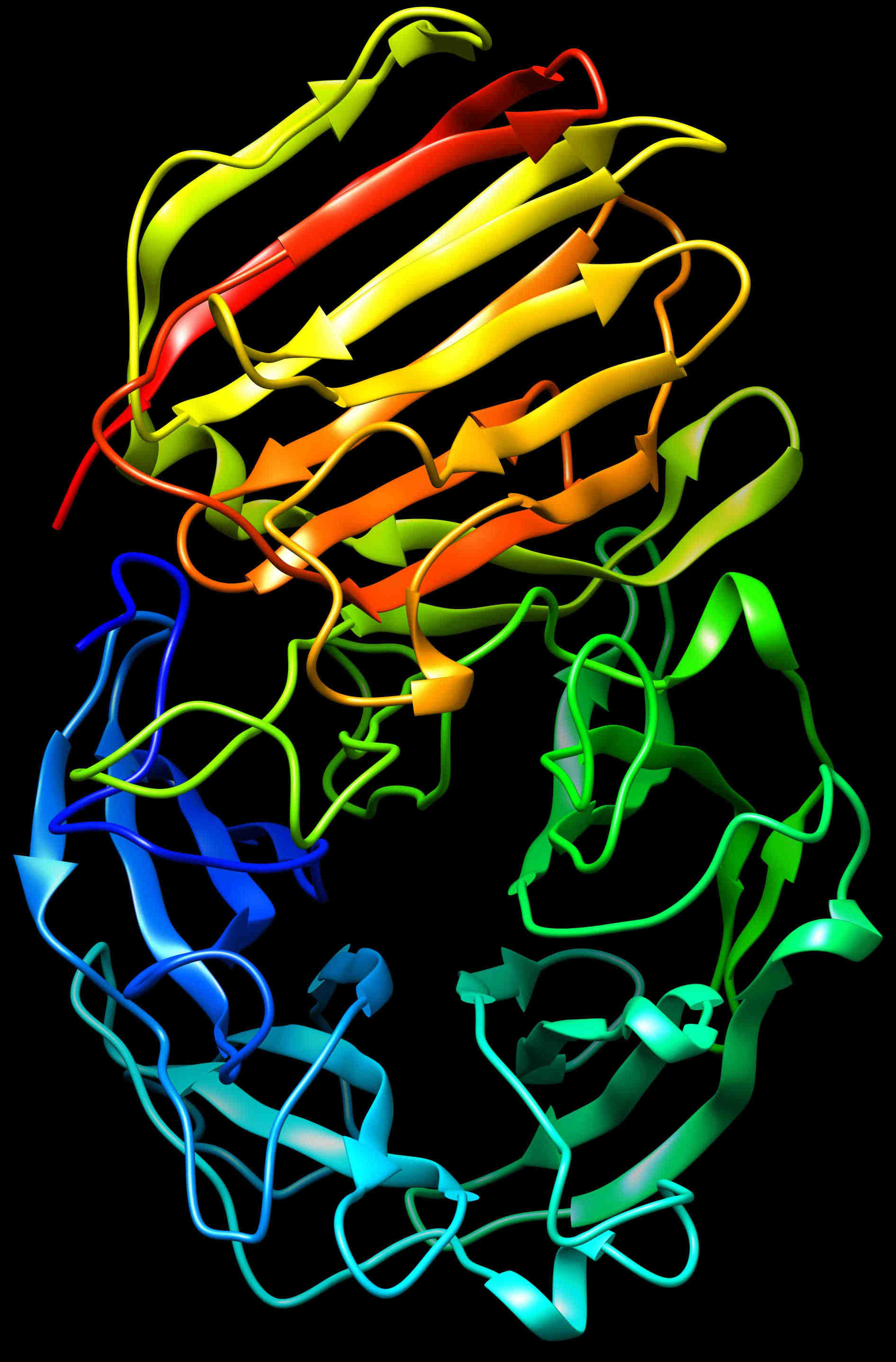

Supplement: S1 Dataset — 3D models were generated from sequences retrieved from the non-redundant protein sequence database using SWISS-MODEL. (ZIP) [file pone.0200607.s001.zip › Homology_Models/Pseudogymnoascusp2m1.jpg]

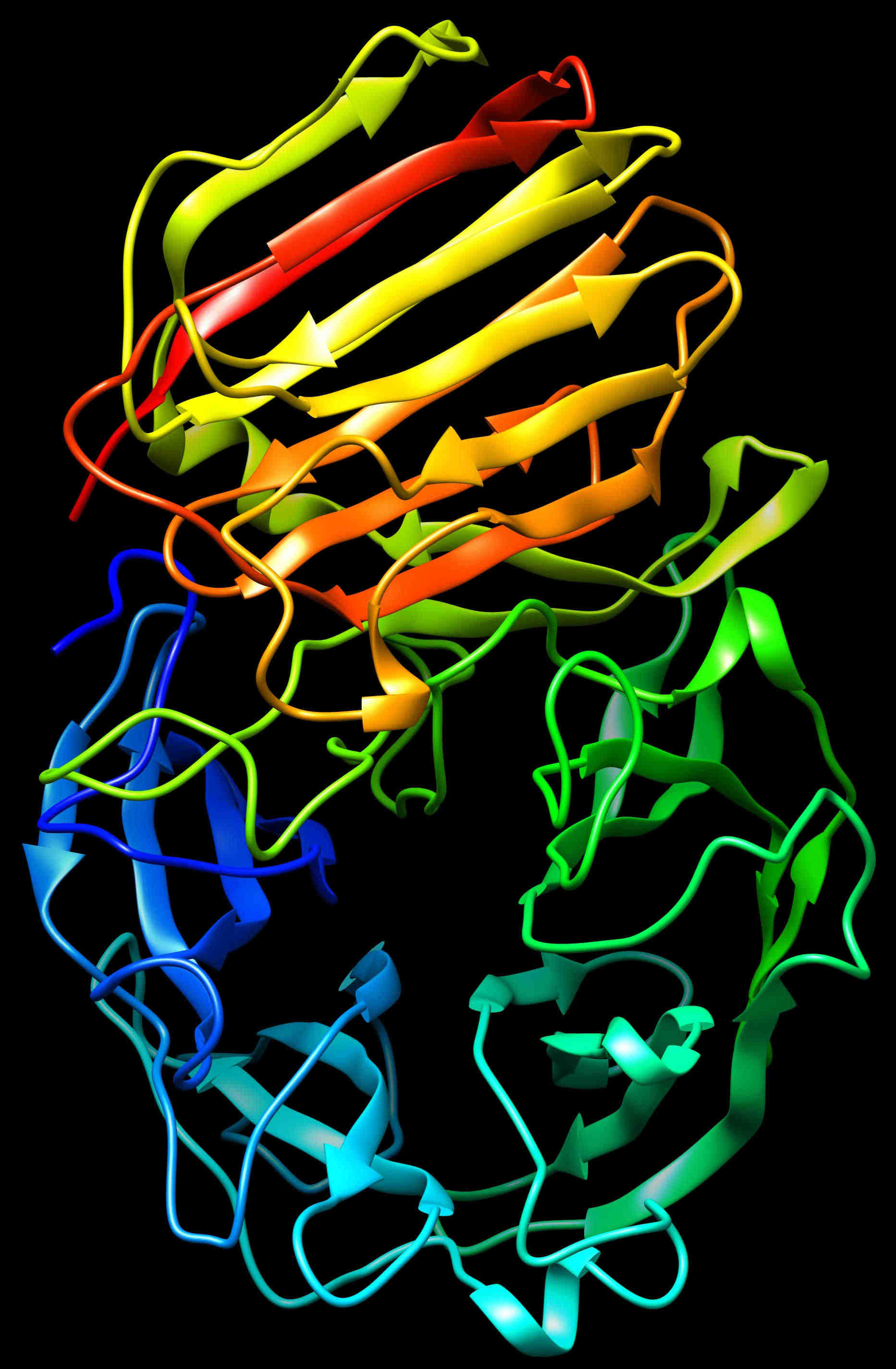

Supplement: S1 Dataset — 3D models were generated from sequences retrieved from the non-redundant protein sequence database using SWISS-MODEL. (ZIP) [file pone.0200607.s001.zip › Homology_Models/Pseudogymnoascusp3m2.jpg]

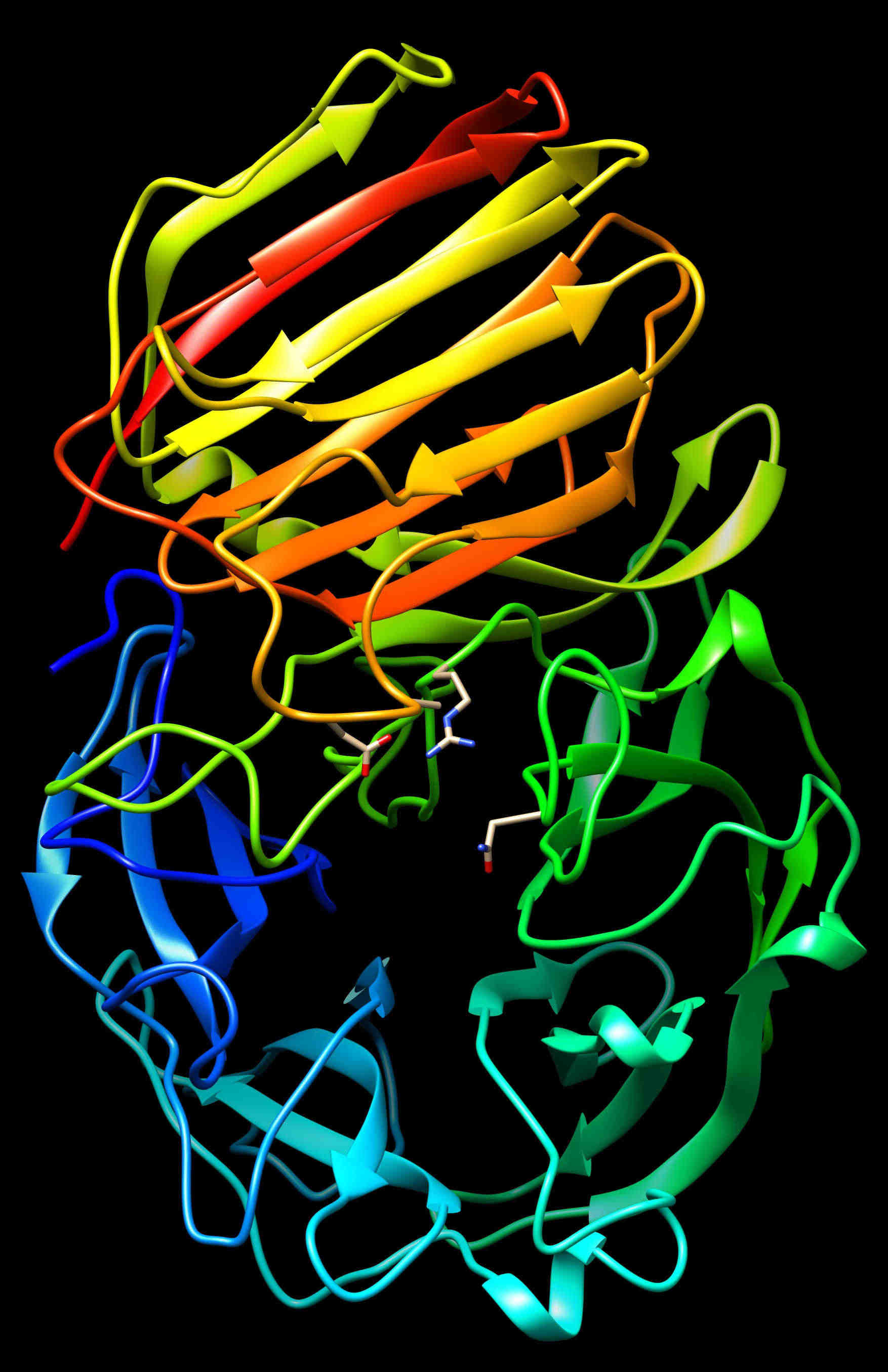

Supplement: S1 Dataset — 3D models were generated from sequences retrieved from the non-redundant protein sequence database using SWISS-MODEL. (ZIP) [file pone.0200607.s001.zip › Homology_Models/Pseudogymnoascusp4m2.jpg]

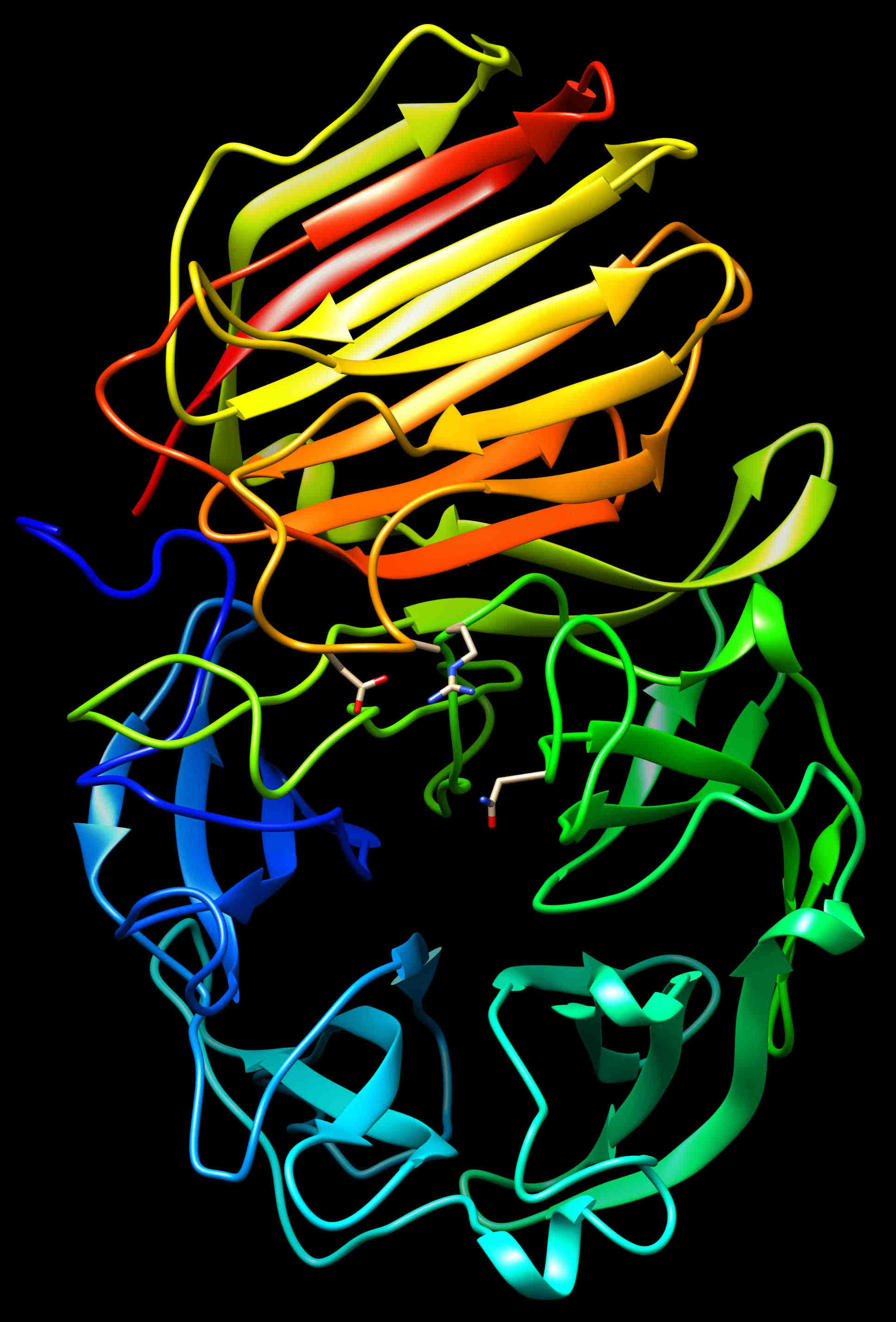

Supplement: S1 Dataset — 3D models were generated from sequences retrieved from the non-redundant protein sequence database using SWISS-MODEL. (ZIP) [file pone.0200607.s001.zip › Homology_Models/Psubrubescens.p1m1.jpg]

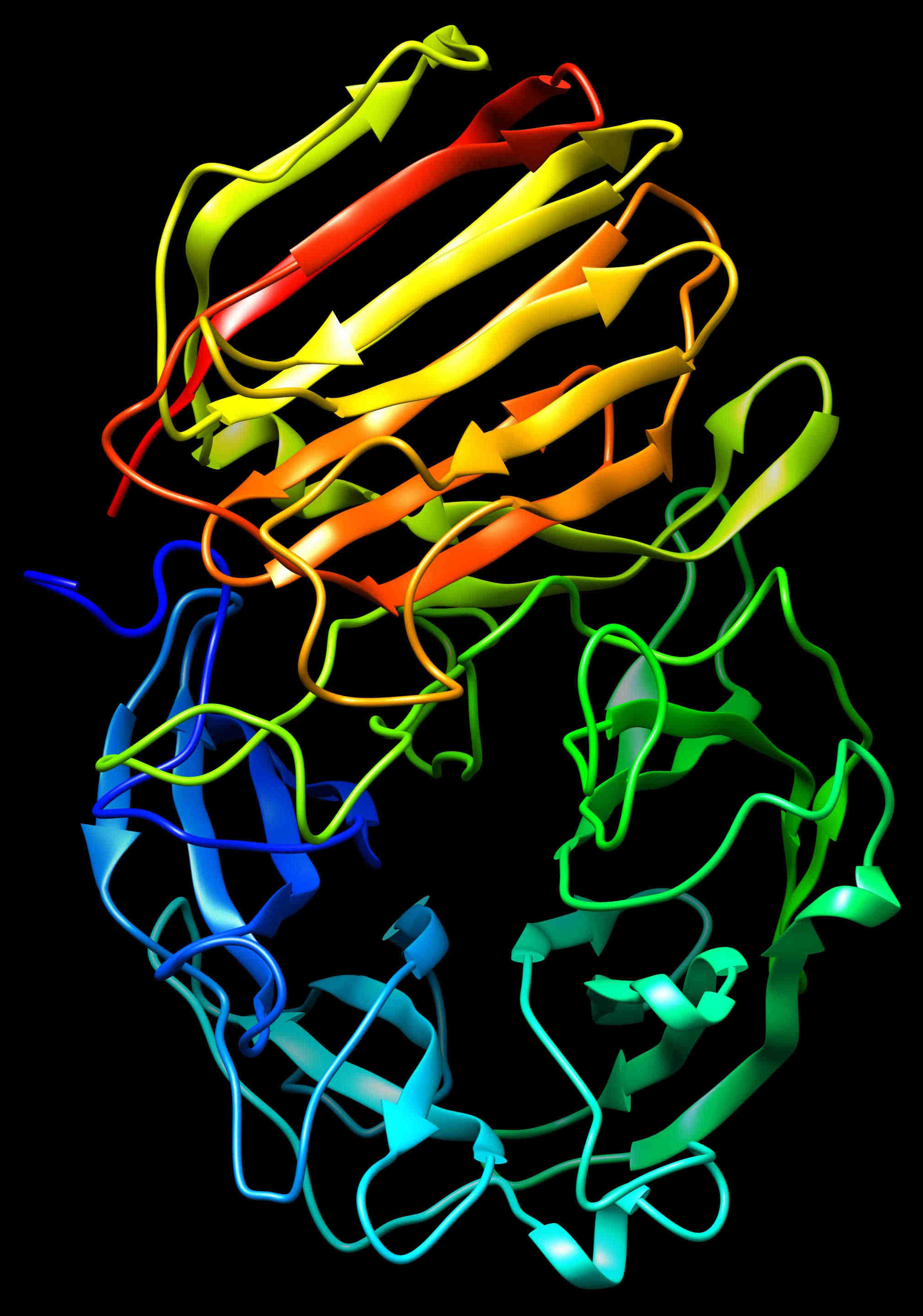

Supplement: S1 Dataset — 3D models were generated from sequences retrieved from the non-redundant protein sequence database using SWISS-MODEL. (ZIP) [file pone.0200607.s001.zip › Homology_Models/Psubrubescensp2.jpg]

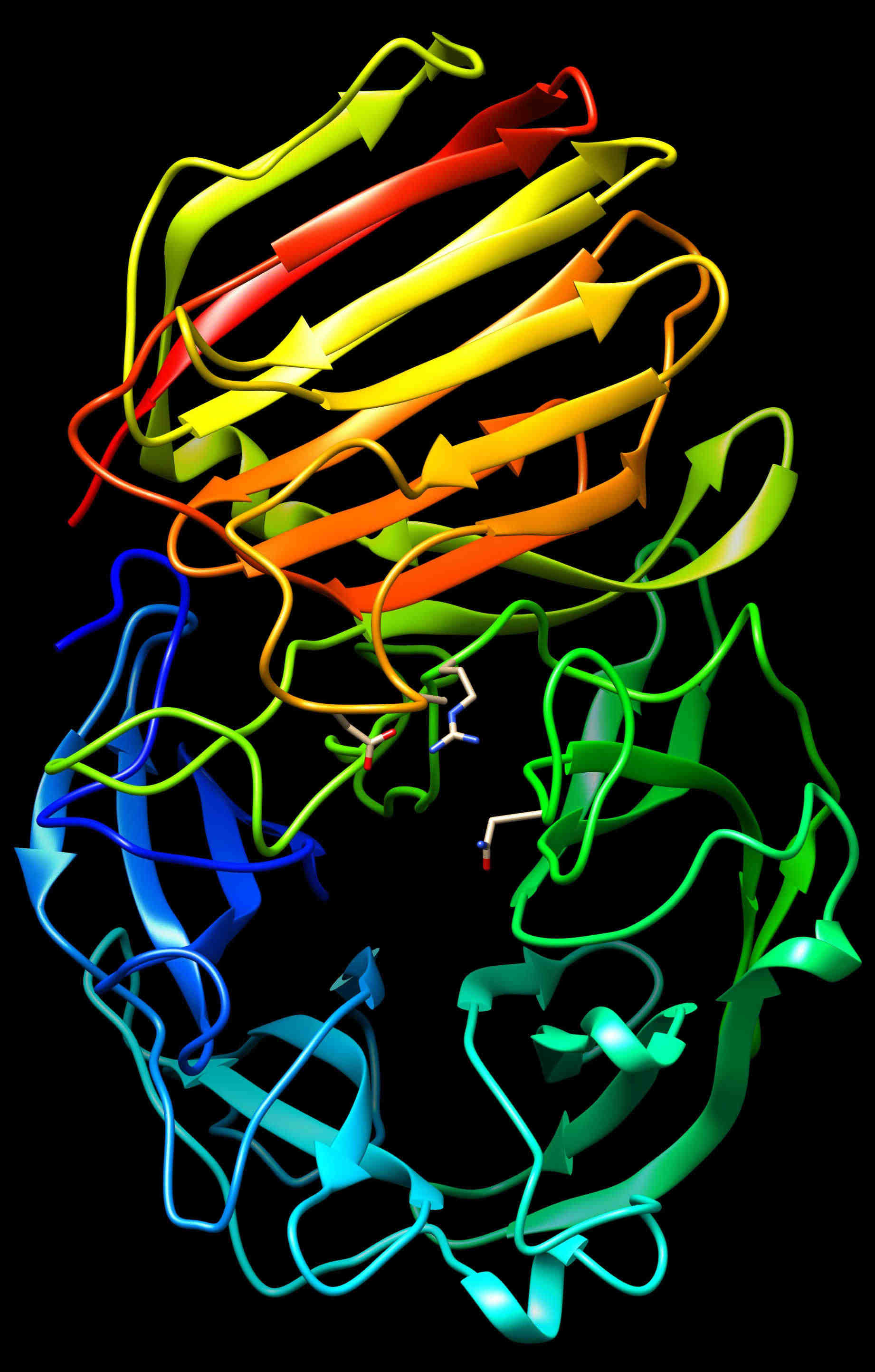

Supplement: S1 Dataset — 3D models were generated from sequences retrieved from the non-redundant protein sequence database using SWISS-MODEL. (ZIP) [file pone.0200607.s001.zip › Homology_Models/Psubrubescensp3.jpg]

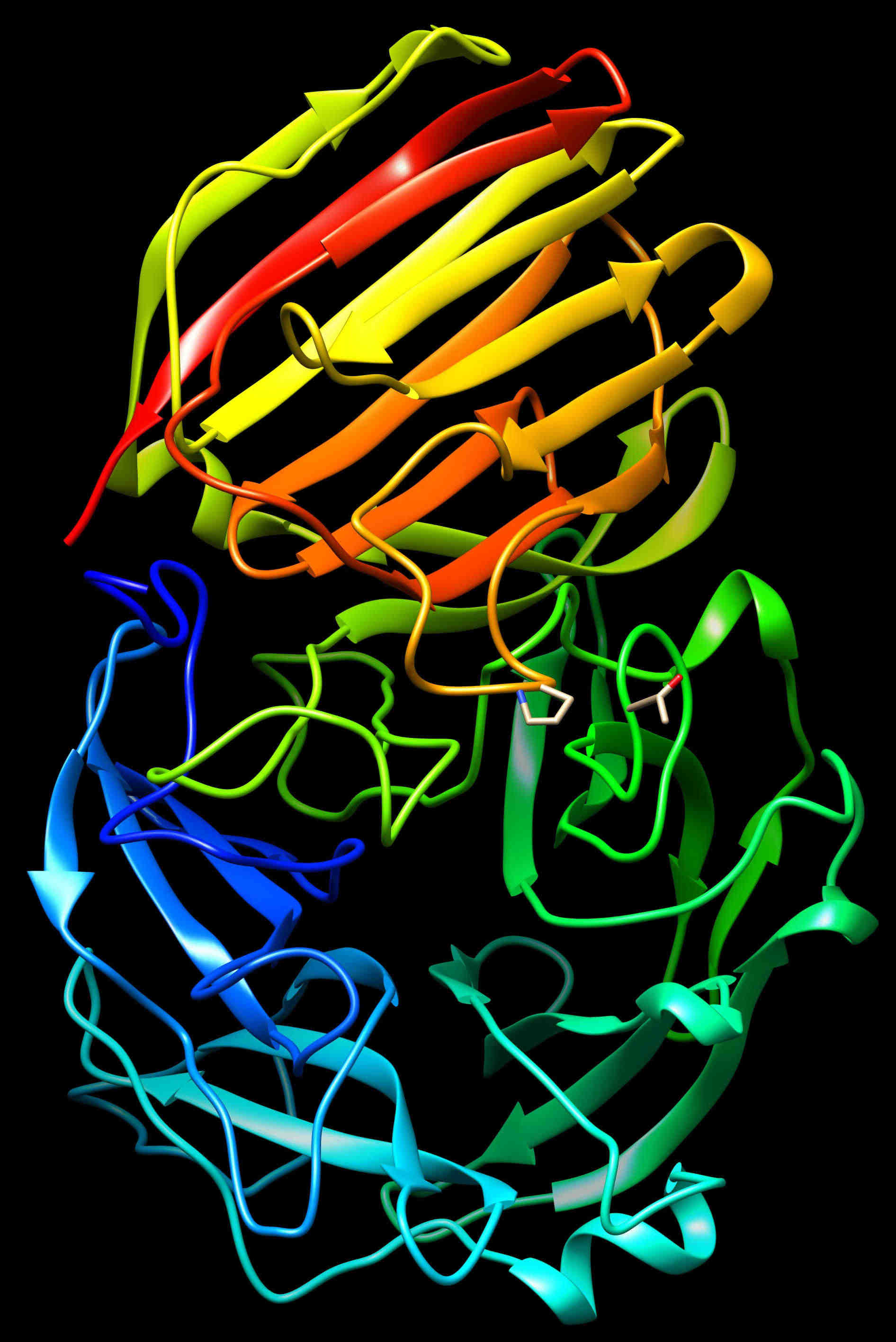

Supplement: S1 Dataset — 3D models were generated from sequences retrieved from the non-redundant protein sequence database using SWISS-MODEL. (ZIP) [file pone.0200607.s001.zip › Homology_Models/Pyrenochaetap1m1.jpg]

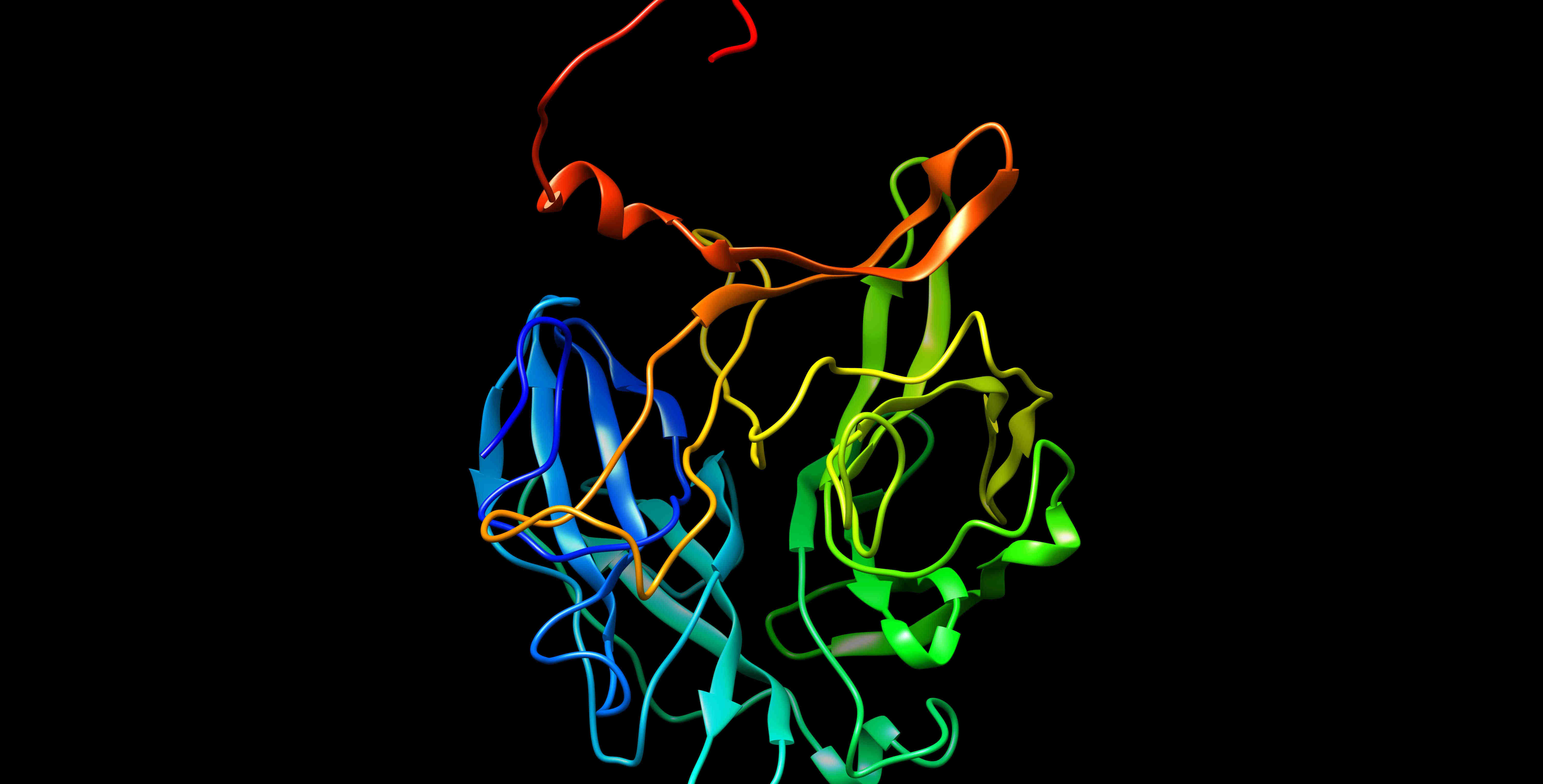

Supplement: S1 Dataset — 3D models were generated from sequences retrieved from the non-redundant protein sequence database using SWISS-MODEL. (ZIP) [file pone.0200607.s001.zip › Homology_Models/Schartarump18m2.jpg]

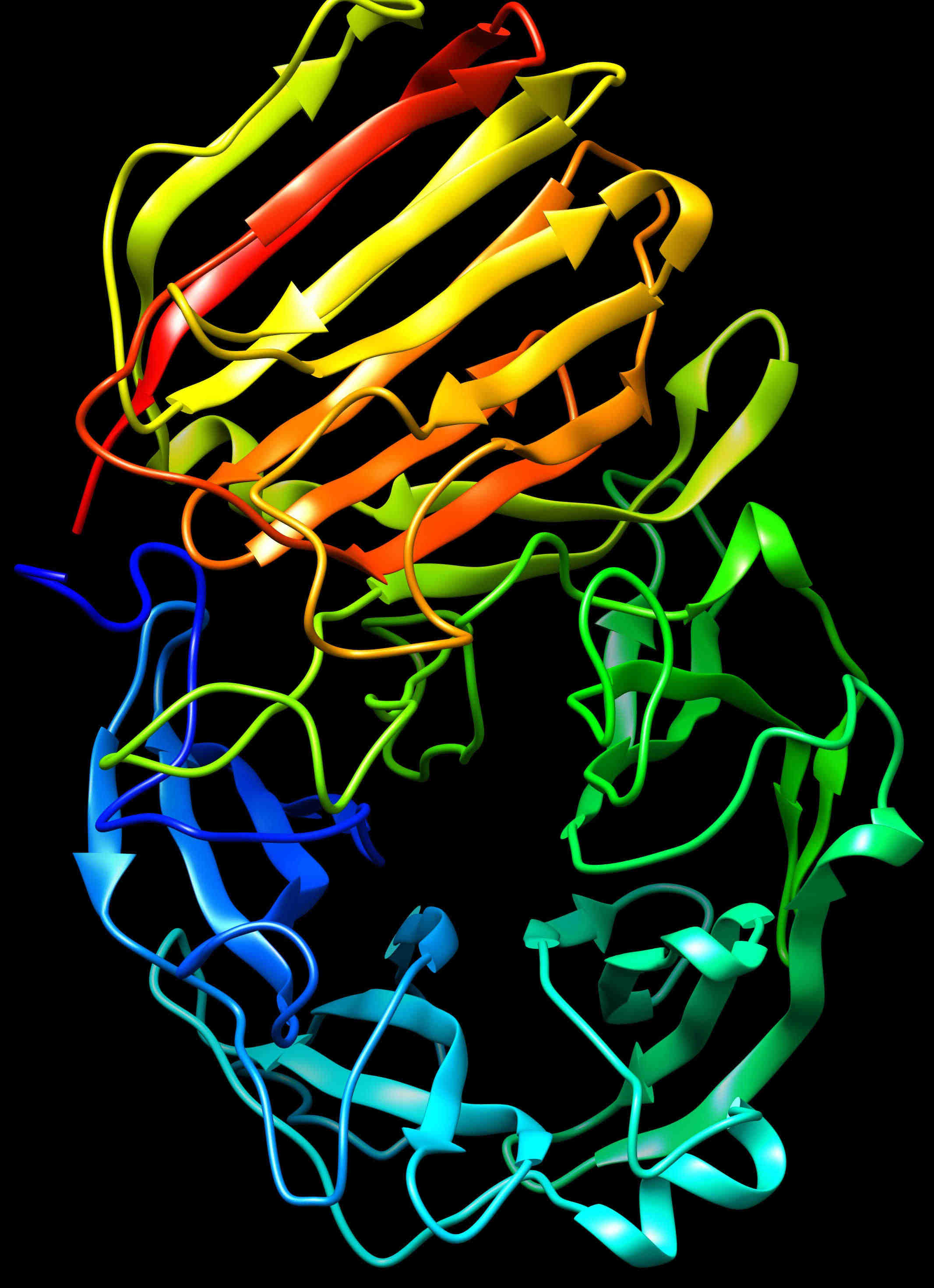

Supplement: S1 Dataset — 3D models were generated from sequences retrieved from the non-redundant protein sequence database using SWISS-MODEL. (ZIP) [file pone.0200607.s001.zip › Homology_Models/Schartarump1m1.jpg]

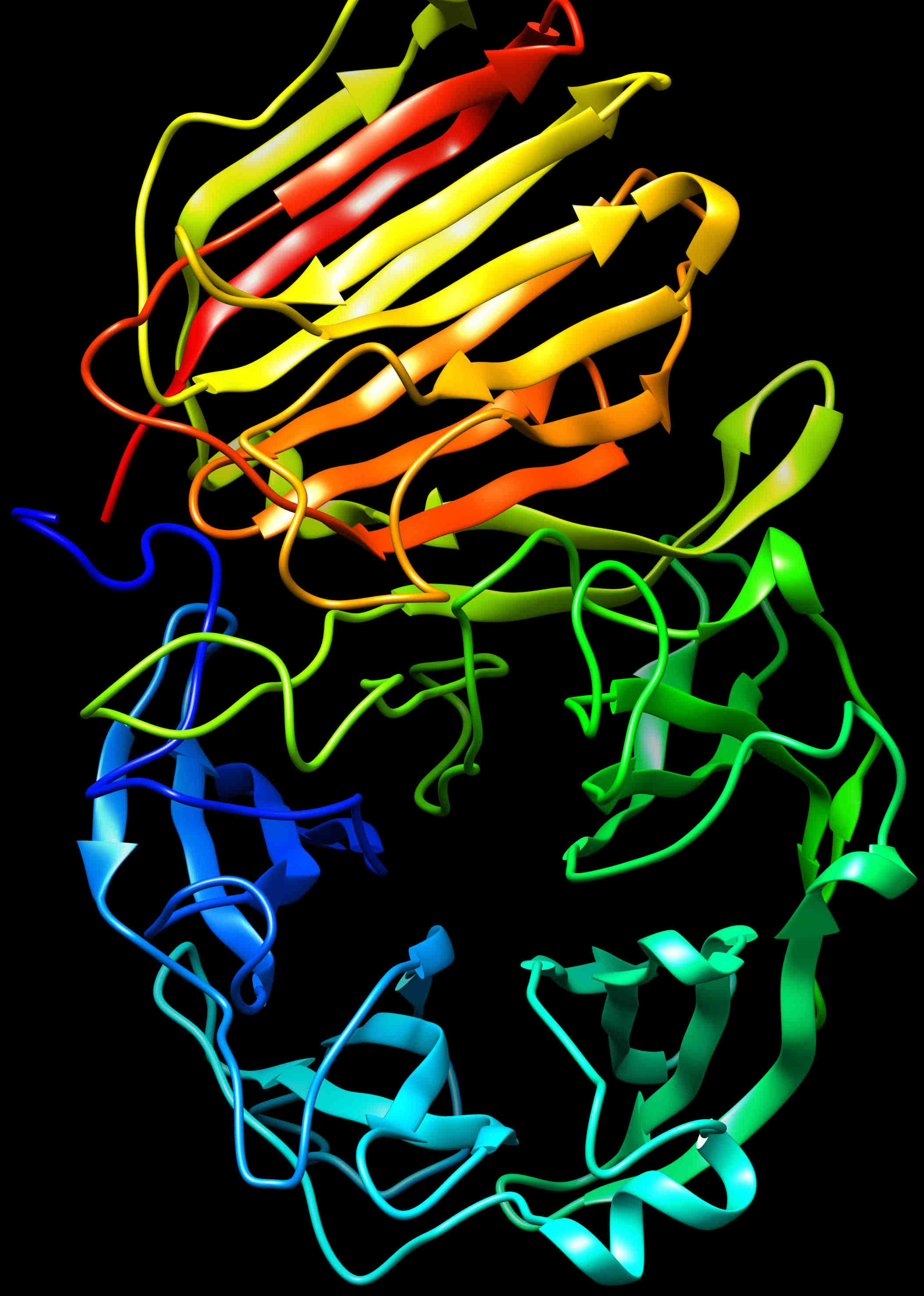

Supplement: S1 Dataset — 3D models were generated from sequences retrieved from the non-redundant protein sequence database using SWISS-MODEL. (ZIP) [file pone.0200607.s001.zip › Homology_Models/Schartarump2m1.jpg]

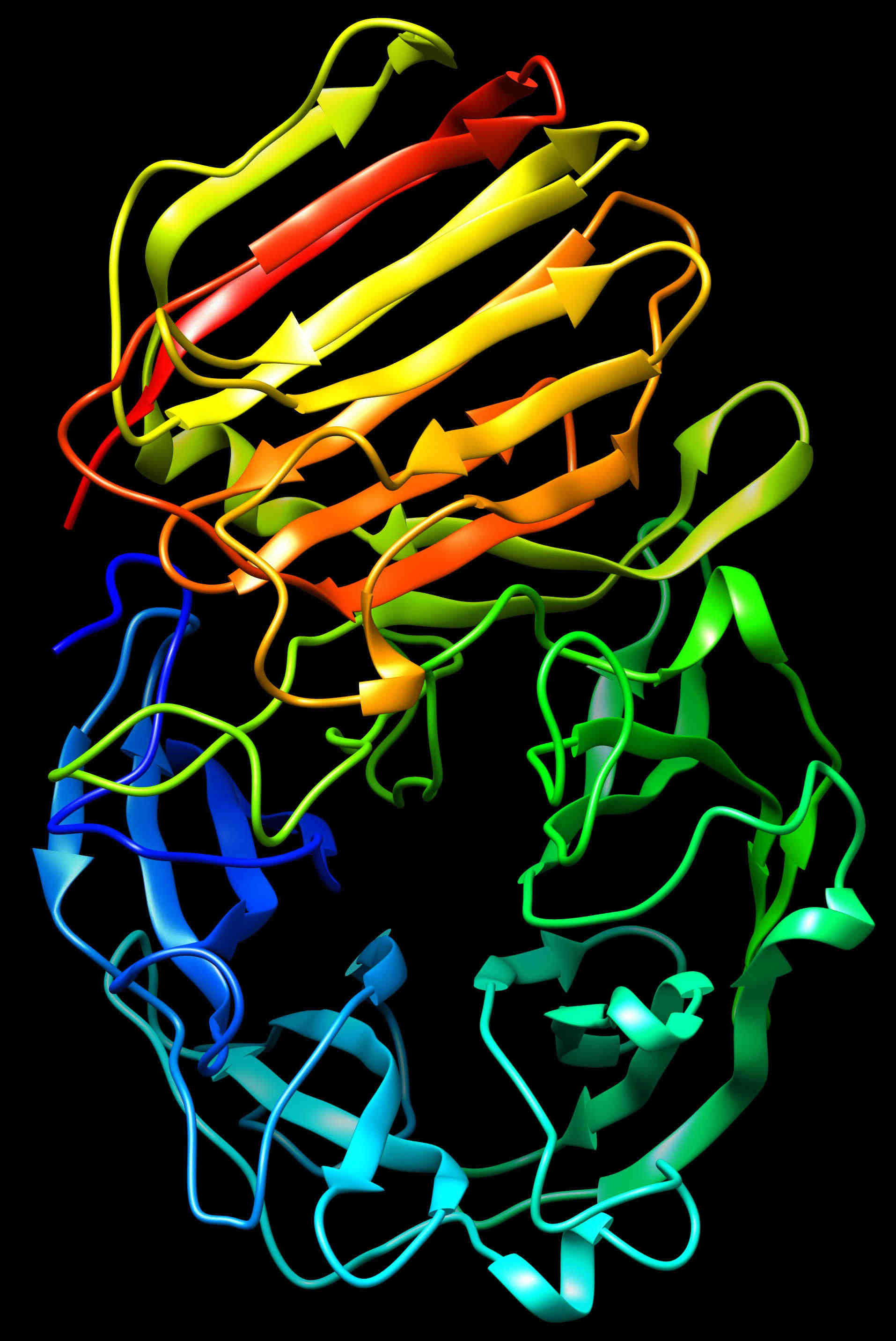

Supplement: S1 Dataset — 3D models were generated from sequences retrieved from the non-redundant protein sequence database using SWISS-MODEL. (ZIP) [file pone.0200607.s001.zip › Homology_Models/Schartarump3m2.jpg]

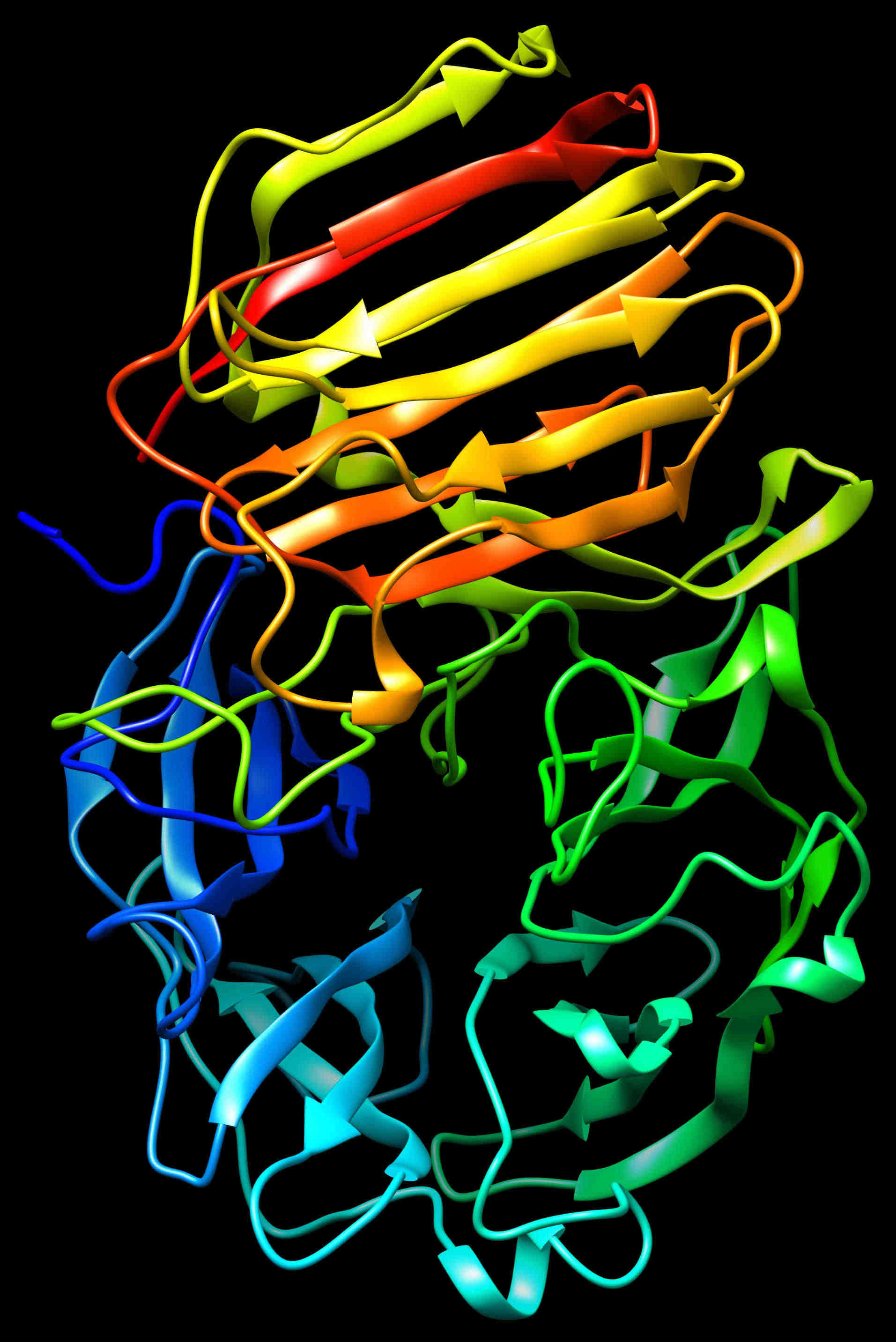

Supplement: S1 Dataset — 3D models were generated from sequences retrieved from the non-redundant protein sequence database using SWISS-MODEL. (ZIP) [file pone.0200607.s001.zip › Homology_Models/Schartarump4m2.jpg]

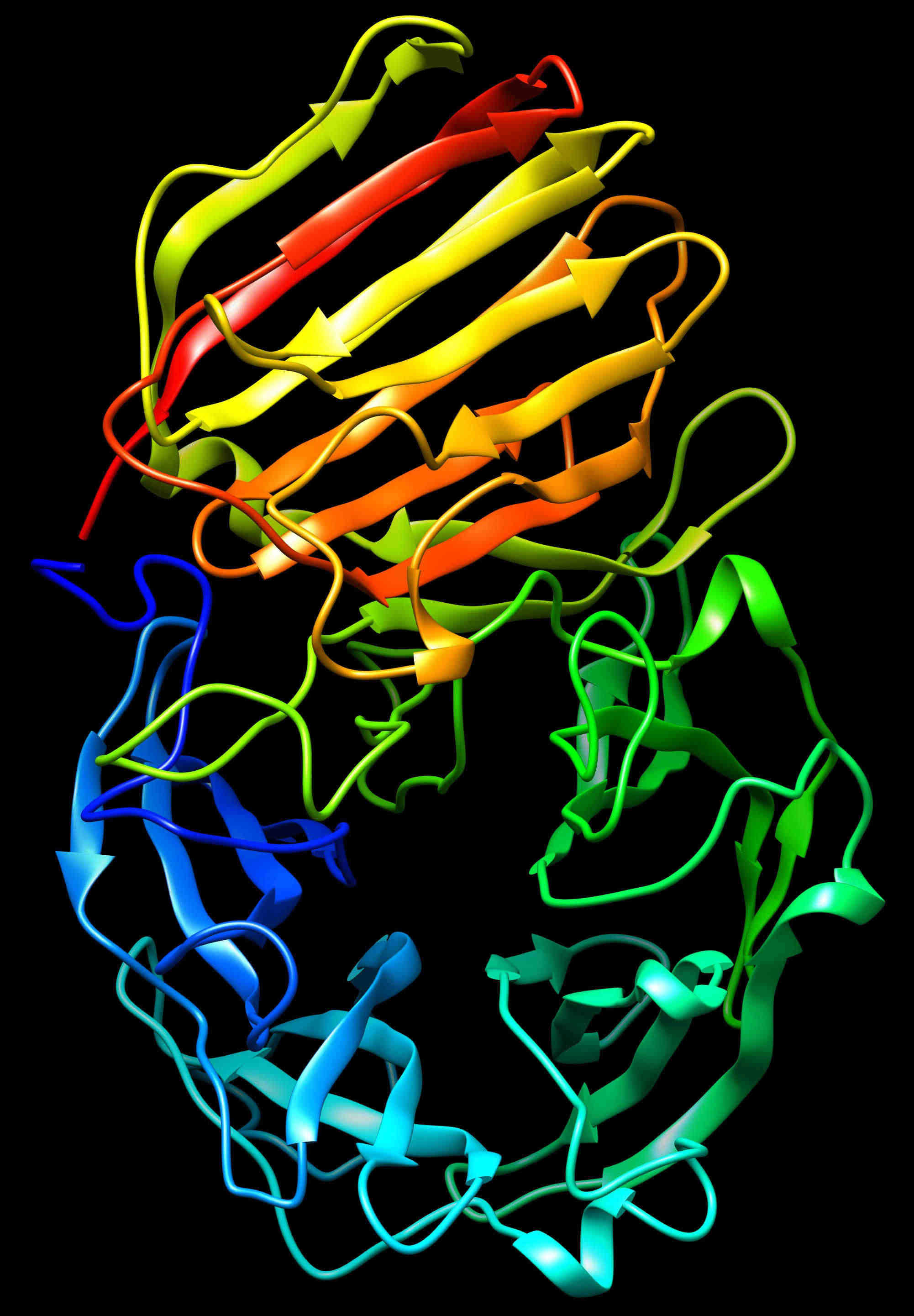

Supplement: S1 Dataset — 3D models were generated from sequences retrieved from the non-redundant protein sequence database using SWISS-MODEL. (ZIP) [file pone.0200607.s001.zip › Homology_Models/Schartarump5m2.jpg]

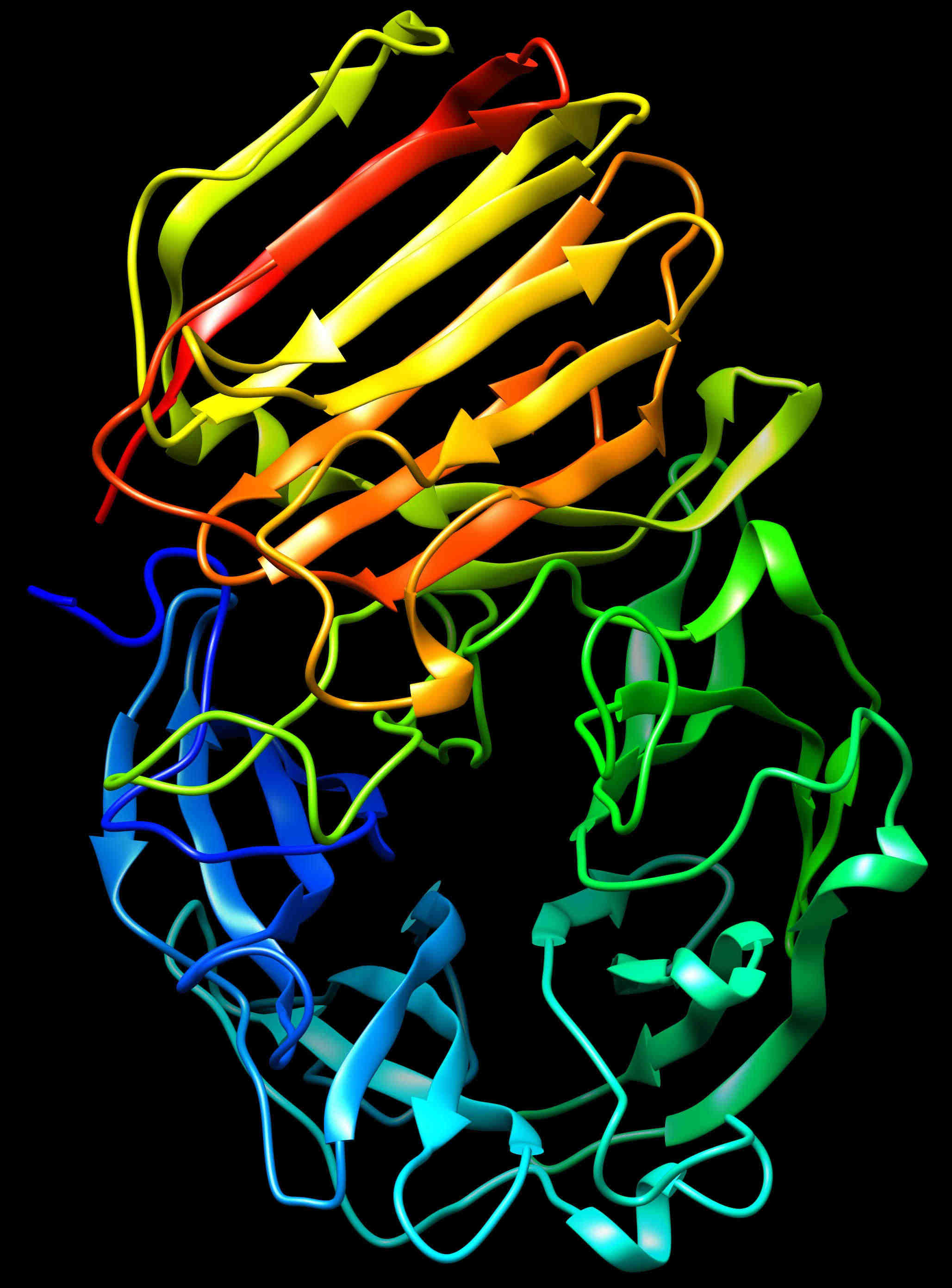

Supplement: S1 Dataset — 3D models were generated from sequences retrieved from the non-redundant protein sequence database using SWISS-MODEL. (ZIP) [file pone.0200607.s001.zip › Homology_Models/Schartarump6m2.jpg]

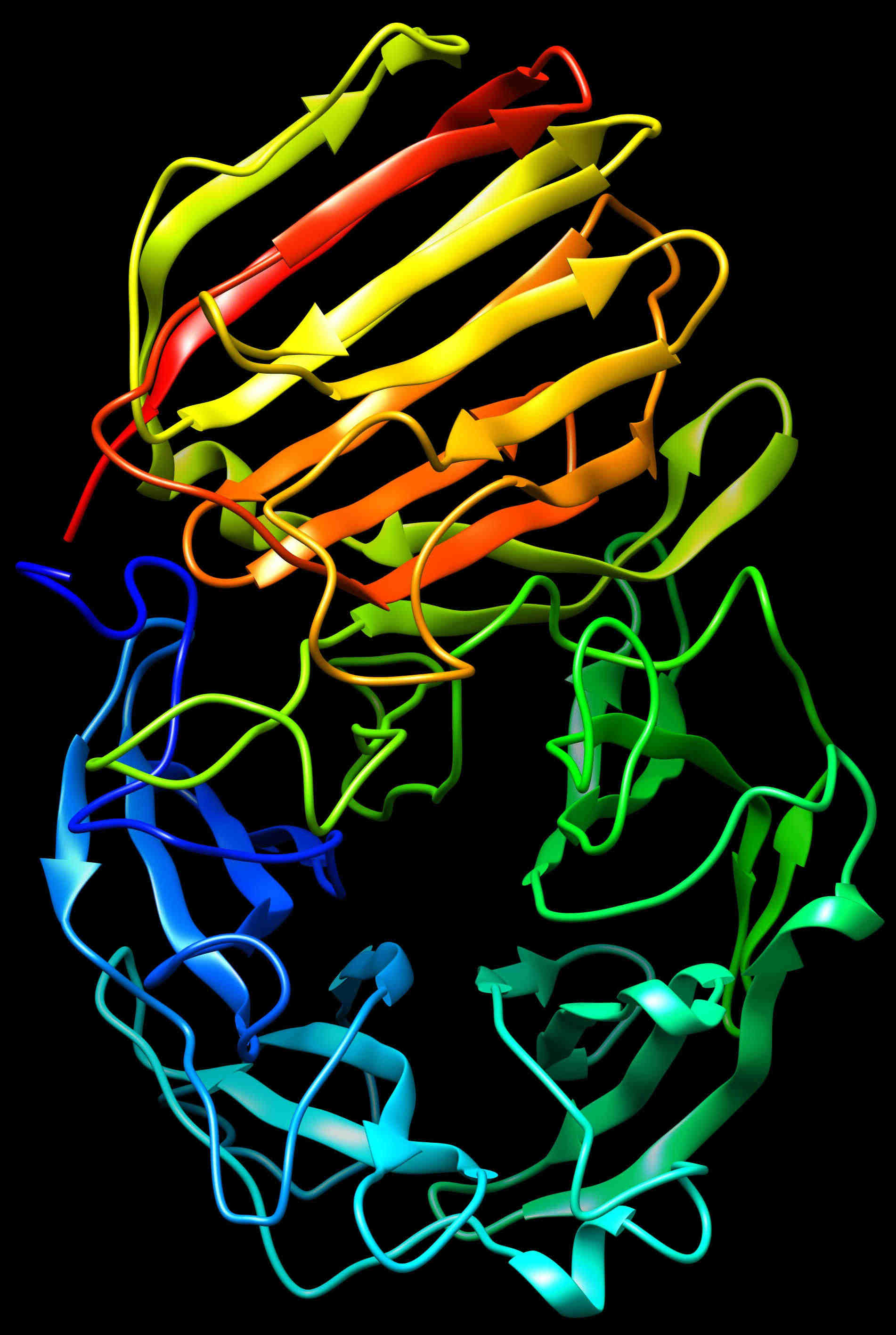

Supplement: S1 Dataset — 3D models were generated from sequences retrieved from the non-redundant protein sequence database using SWISS-MODEL. (ZIP) [file pone.0200607.s001.zip › Homology_Models/Schartarump7m1.jpg]

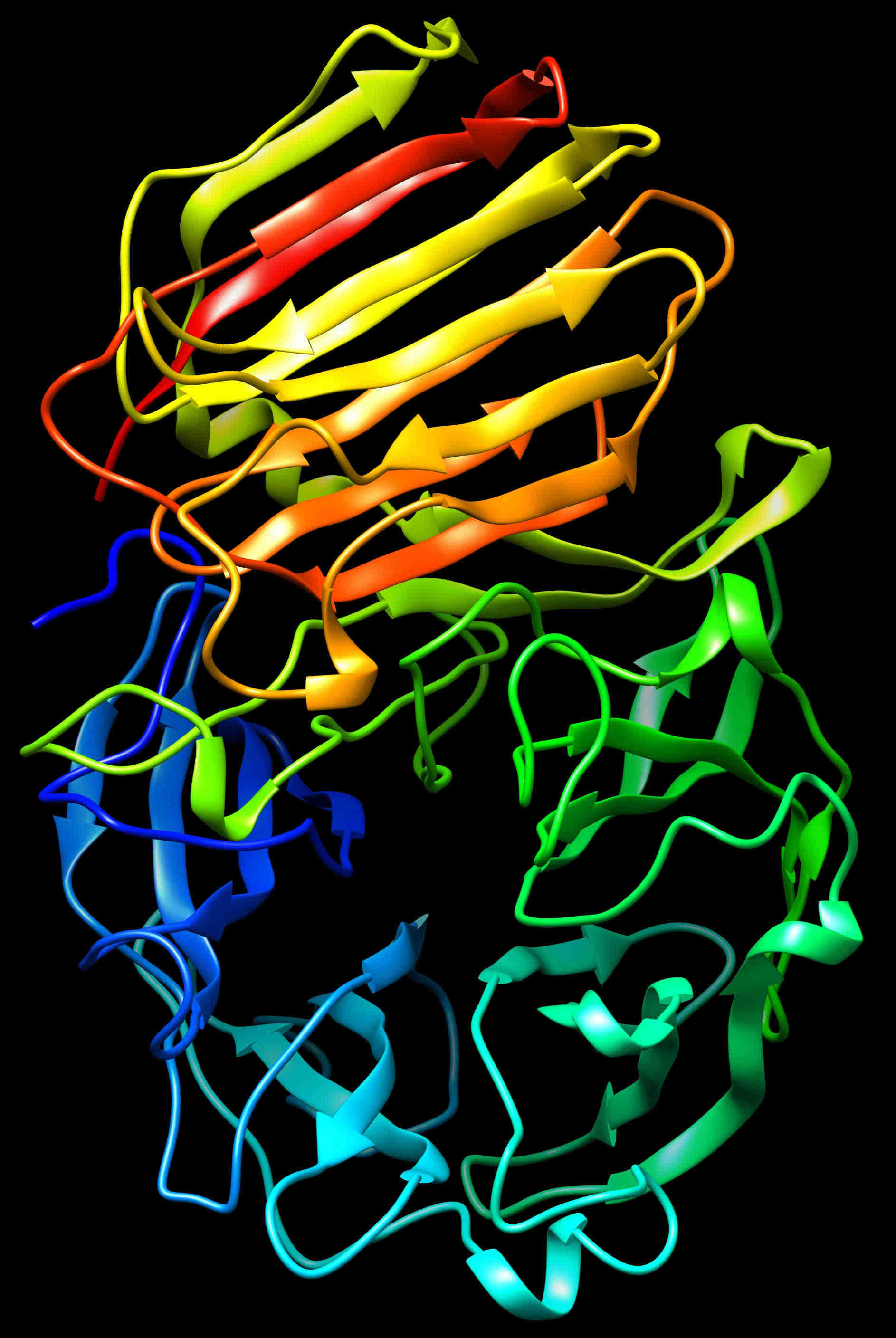

Supplement: S1 Dataset — 3D models were generated from sequences retrieved from the non-redundant protein sequence database using SWISS-MODEL. (ZIP) [file pone.0200607.s001.zip › Homology_Models/Schlorohalonatap1m1.jpg]

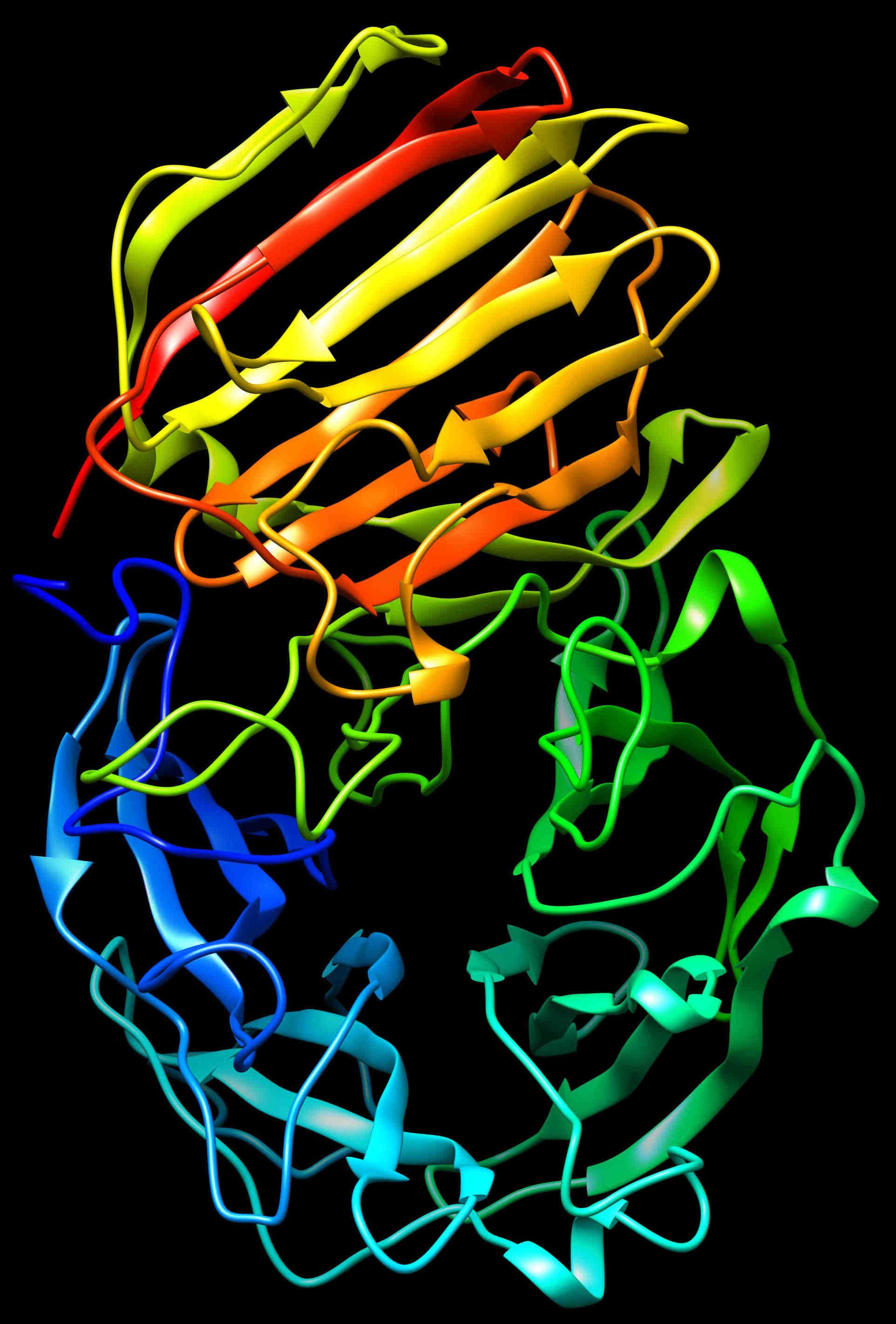

Supplement: S1 Dataset — 3D models were generated from sequences retrieved from the non-redundant protein sequence database using SWISS-MODEL. (ZIP) [file pone.0200607.s001.zip › Homology_Models/Schlorohalonatap2m2.jpg]

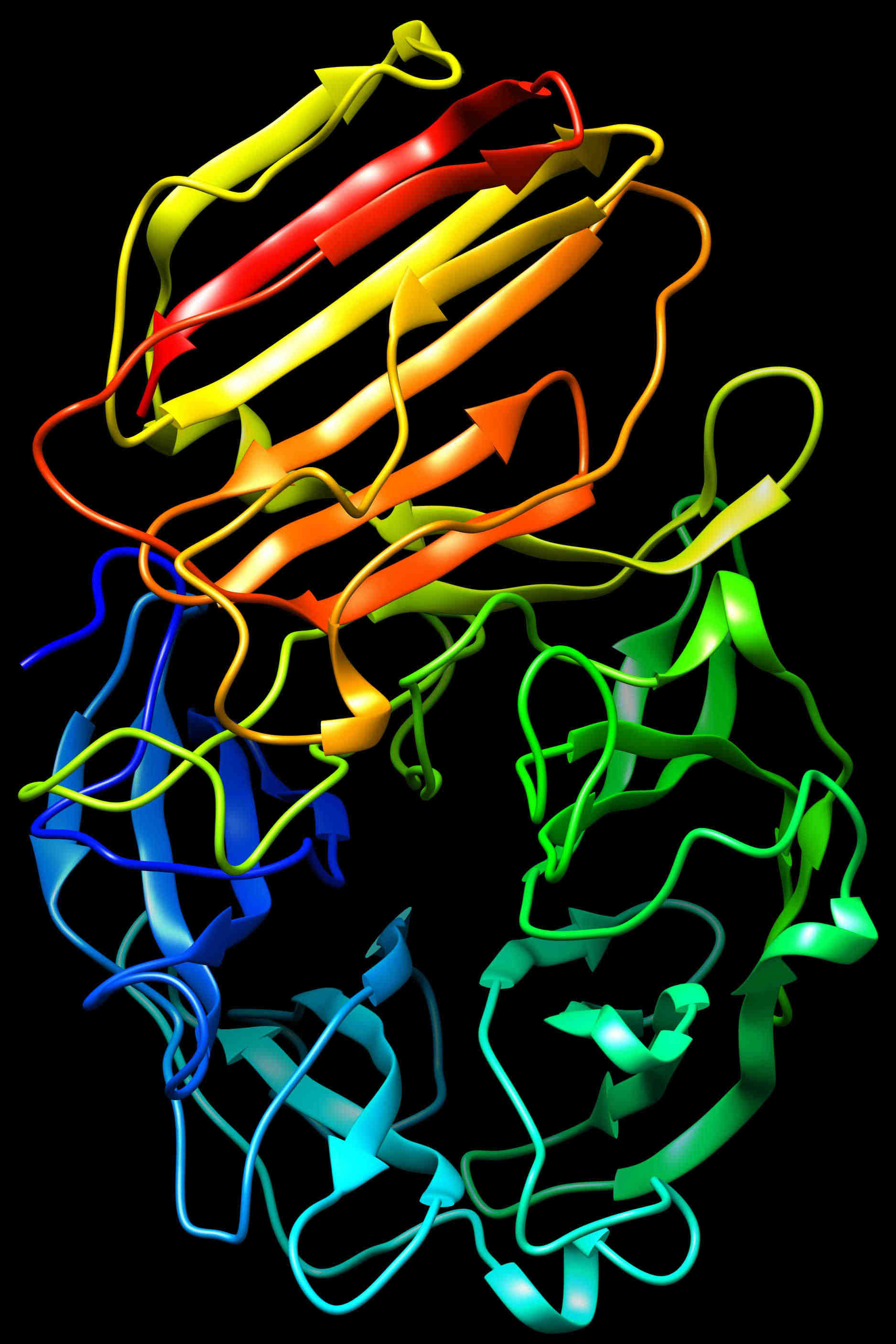

Supplement: S1 Dataset — 3D models were generated from sequences retrieved from the non-redundant protein sequence database using SWISS-MODEL. (ZIP) [file pone.0200607.s001.zip › Homology_Models/Tcellulolyticusp1m2.jpg]

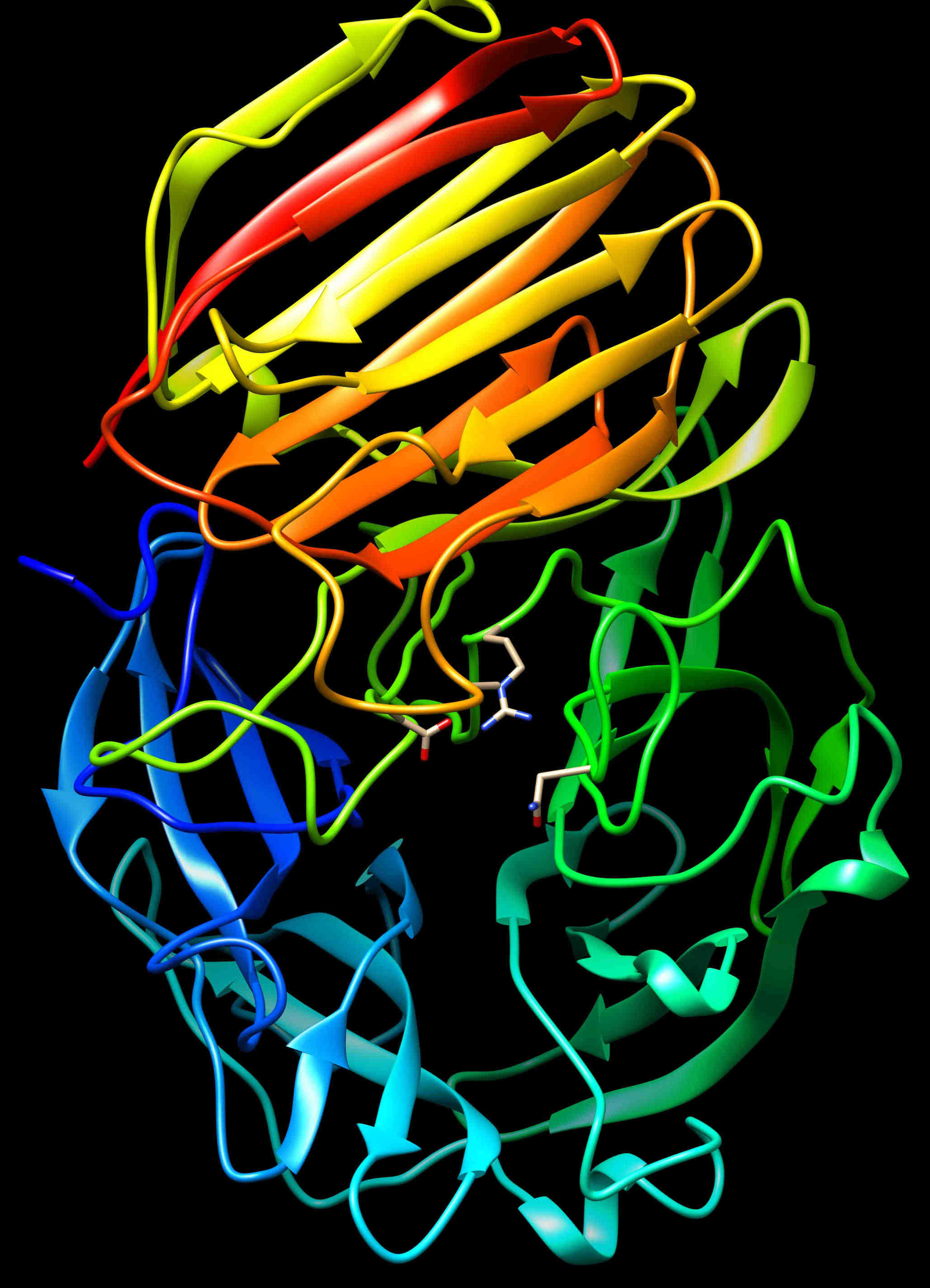

Supplement: S1 Dataset — 3D models were generated from sequences retrieved from the non-redundant protein sequence database using SWISS-MODEL. (ZIP) [file pone.0200607.s001.zip › Homology_Models/Tcellulolyticusp2m1.jpg]

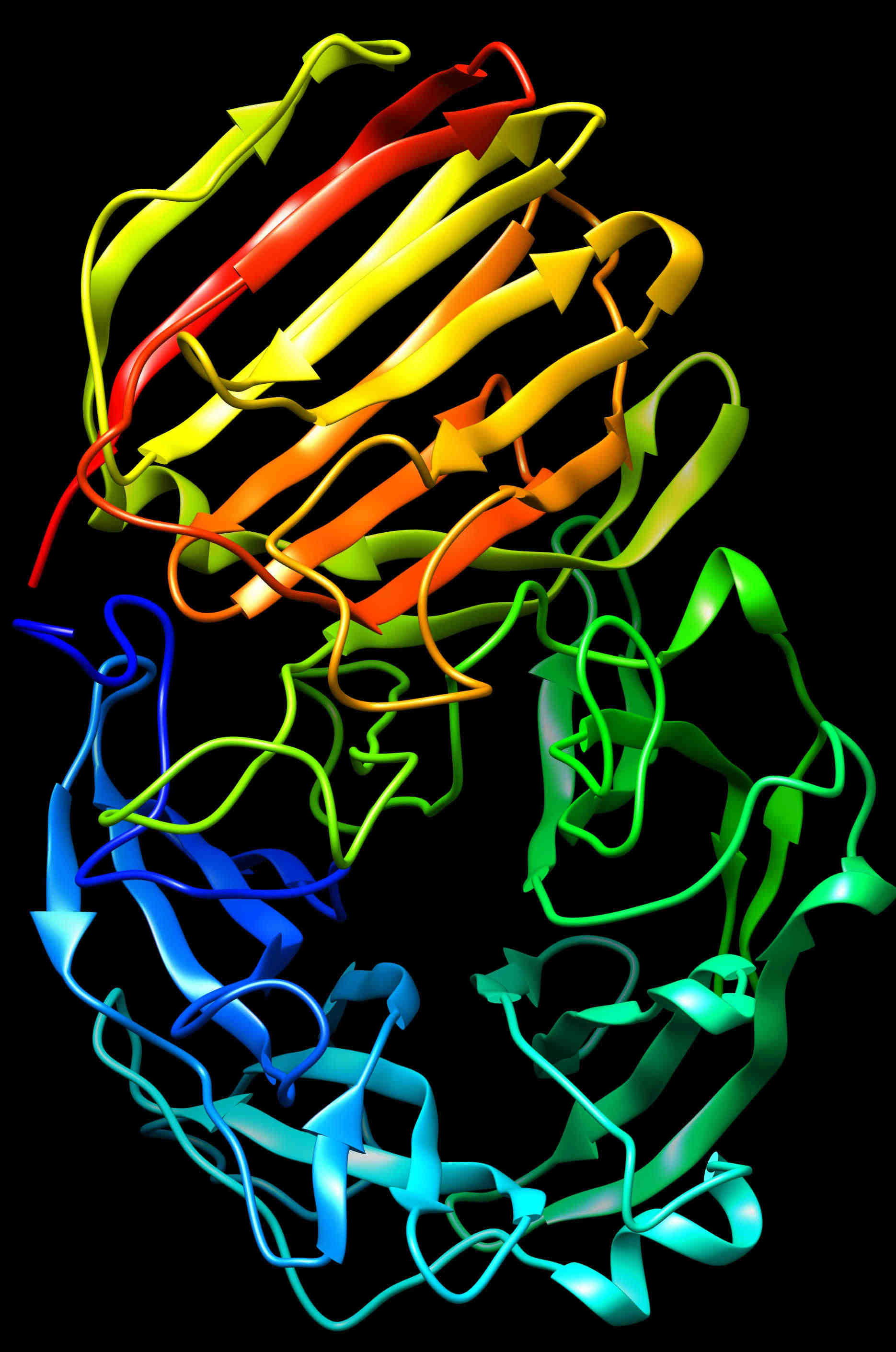

Supplement: S1 Dataset — 3D models were generated from sequences retrieved from the non-redundant protein sequence database using SWISS-MODEL. (ZIP) [file pone.0200607.s001.zip › Homology_Models/Tislandicusp1m1.jpg]

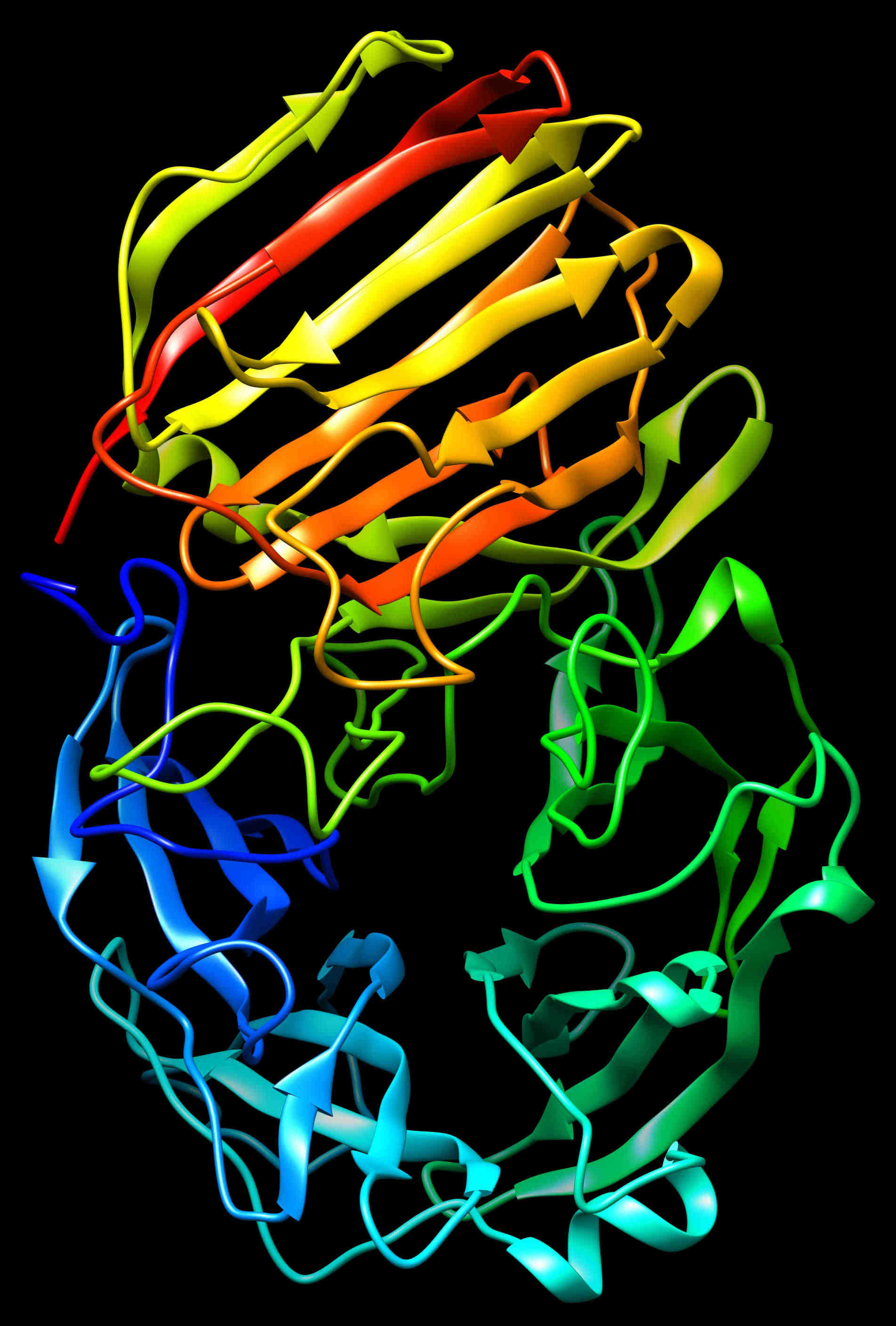

Supplement: S1 Dataset — 3D models were generated from sequences retrieved from the non-redundant protein sequence database using SWISS-MODEL. (ZIP) [file pone.0200607.s001.zip › Homology_Models/Tpurpureogenusp1m1.jpg]

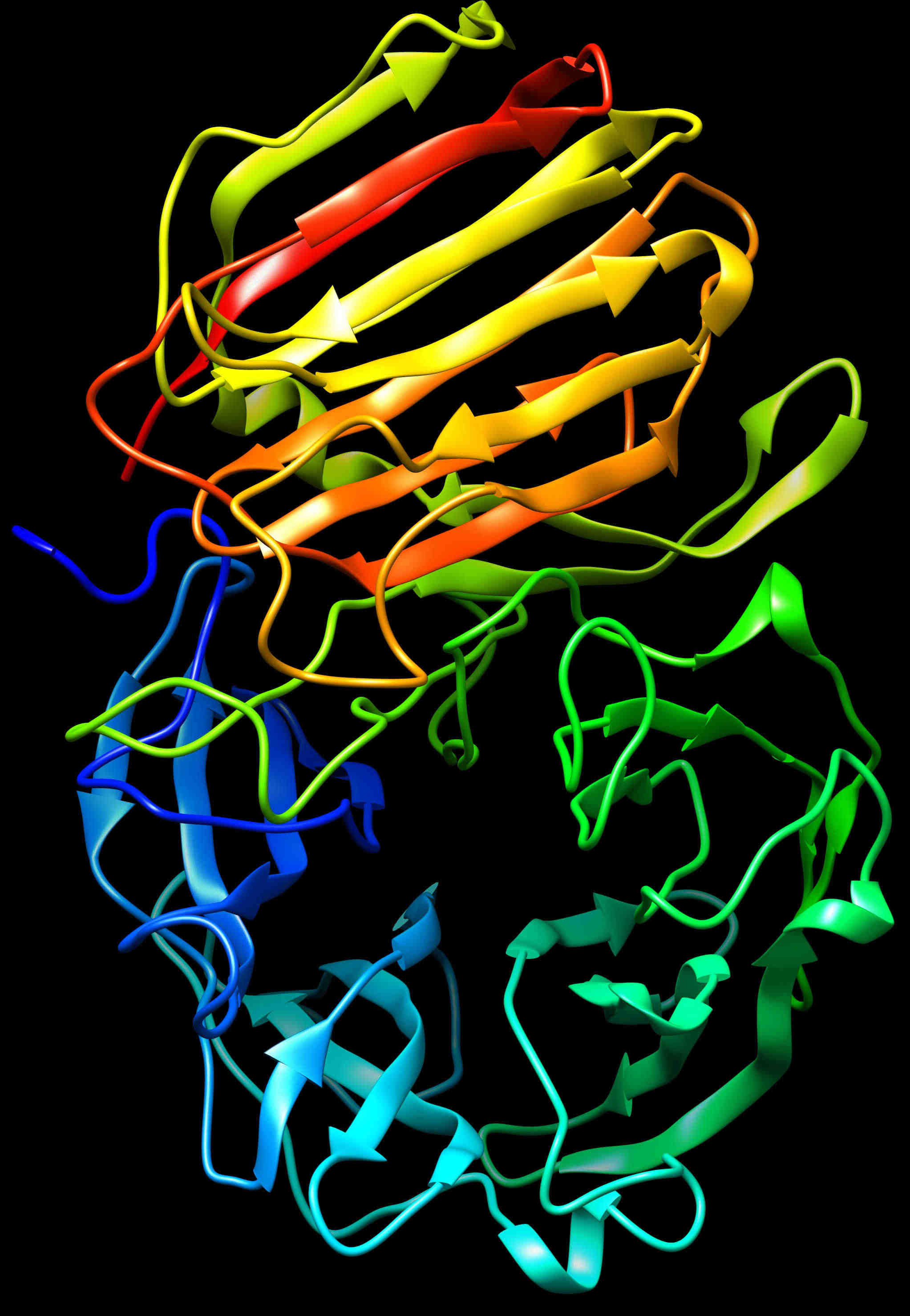

Supplement: S1 Dataset — 3D models were generated from sequences retrieved from the non-redundant protein sequence database using SWISS-MODEL. (ZIP) [file pone.0200607.s001.zip › Homology_Models/Tstipitatusp1m1.jpg]

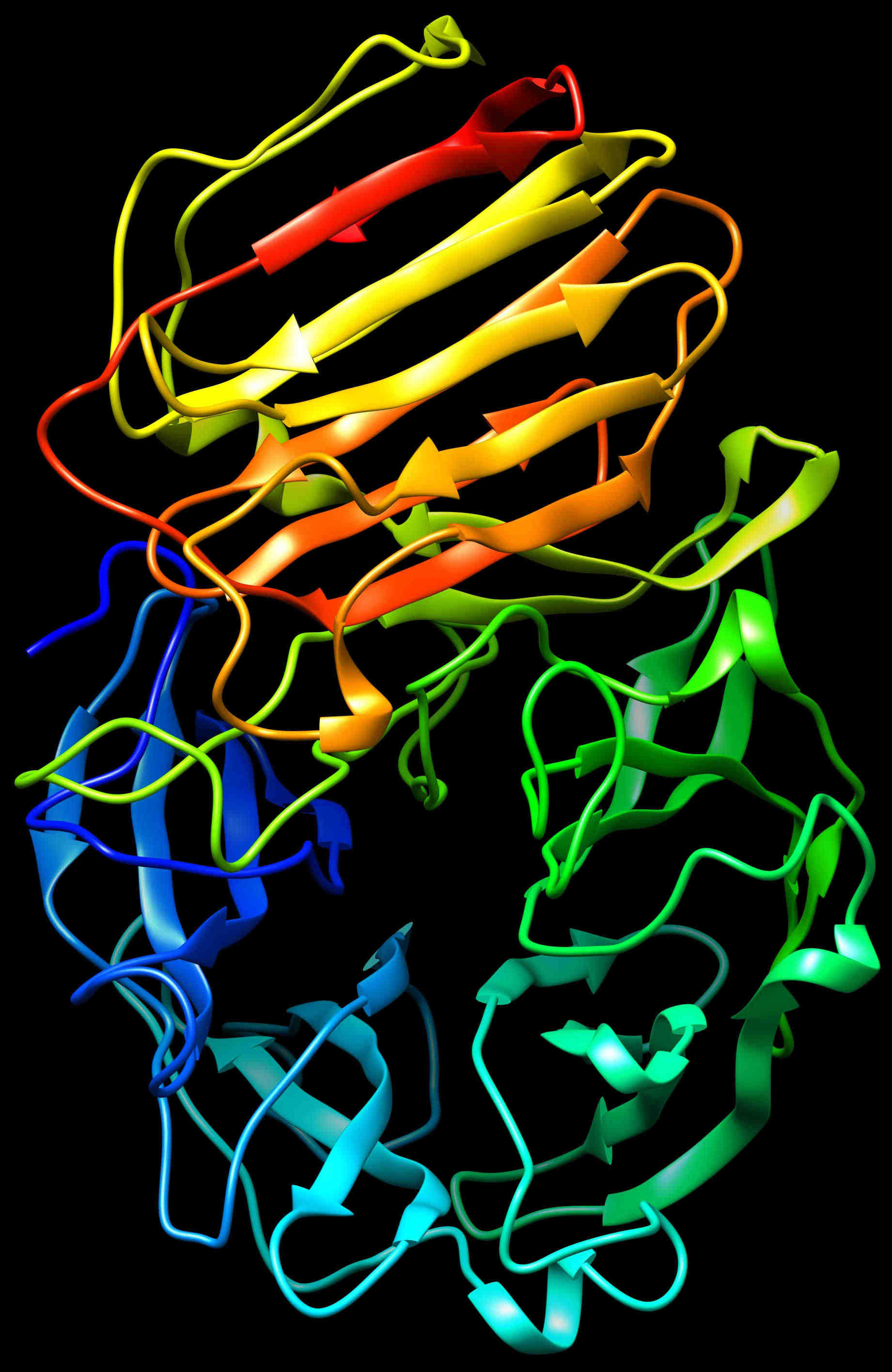

Supplement: S1 Dataset — 3D models were generated from sequences retrieved from the non-redundant protein sequence database using SWISS-MODEL. (ZIP) [file pone.0200607.s001.zip › Homology_Models/Tveeruculusp2m1.jpg]

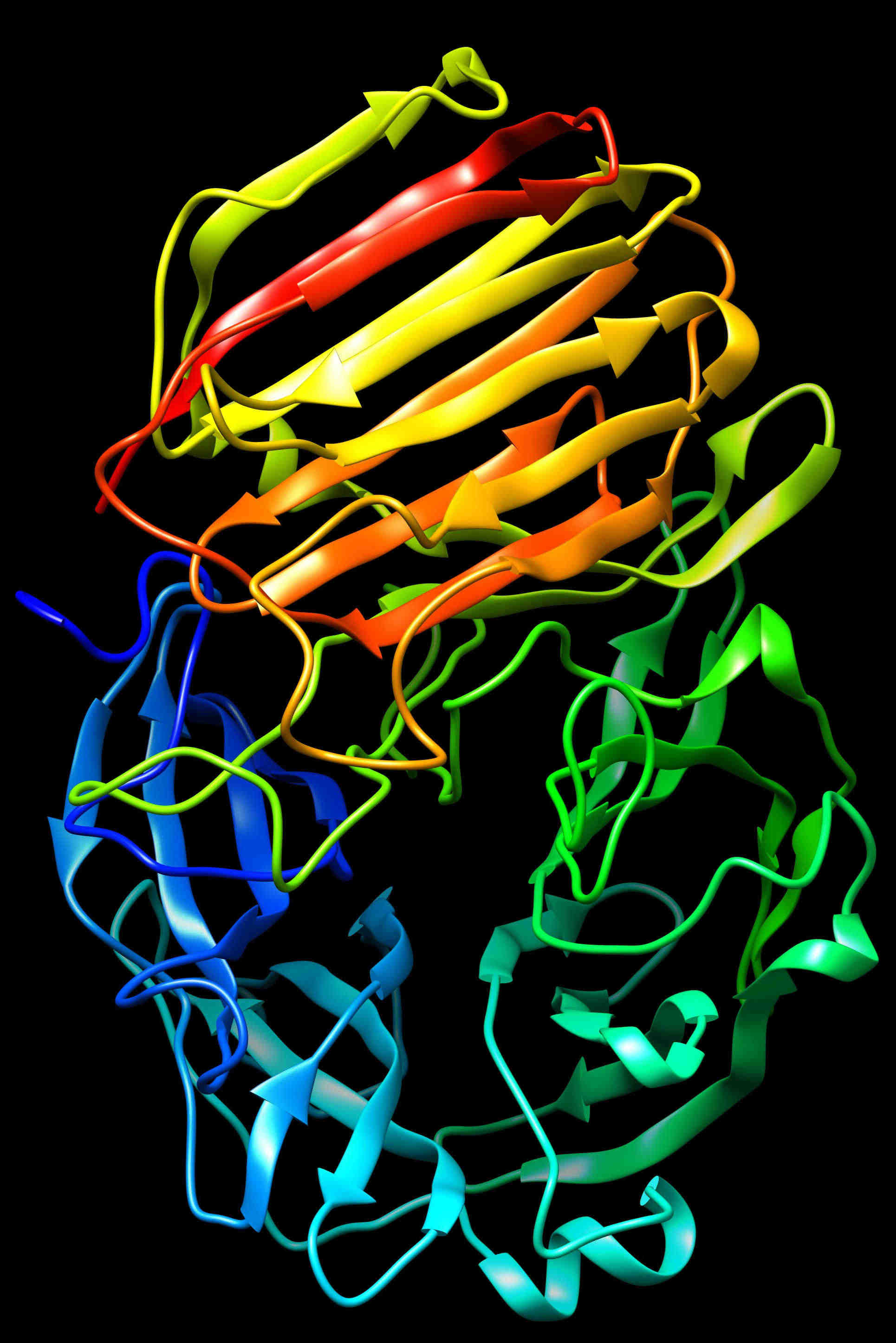

Supplement: S1 Dataset — 3D models were generated from sequences retrieved from the non-redundant protein sequence database using SWISS-MODEL. (ZIP) [file pone.0200607.s001.zip › Homology_Models/Tverruculusp1m1.jpg]

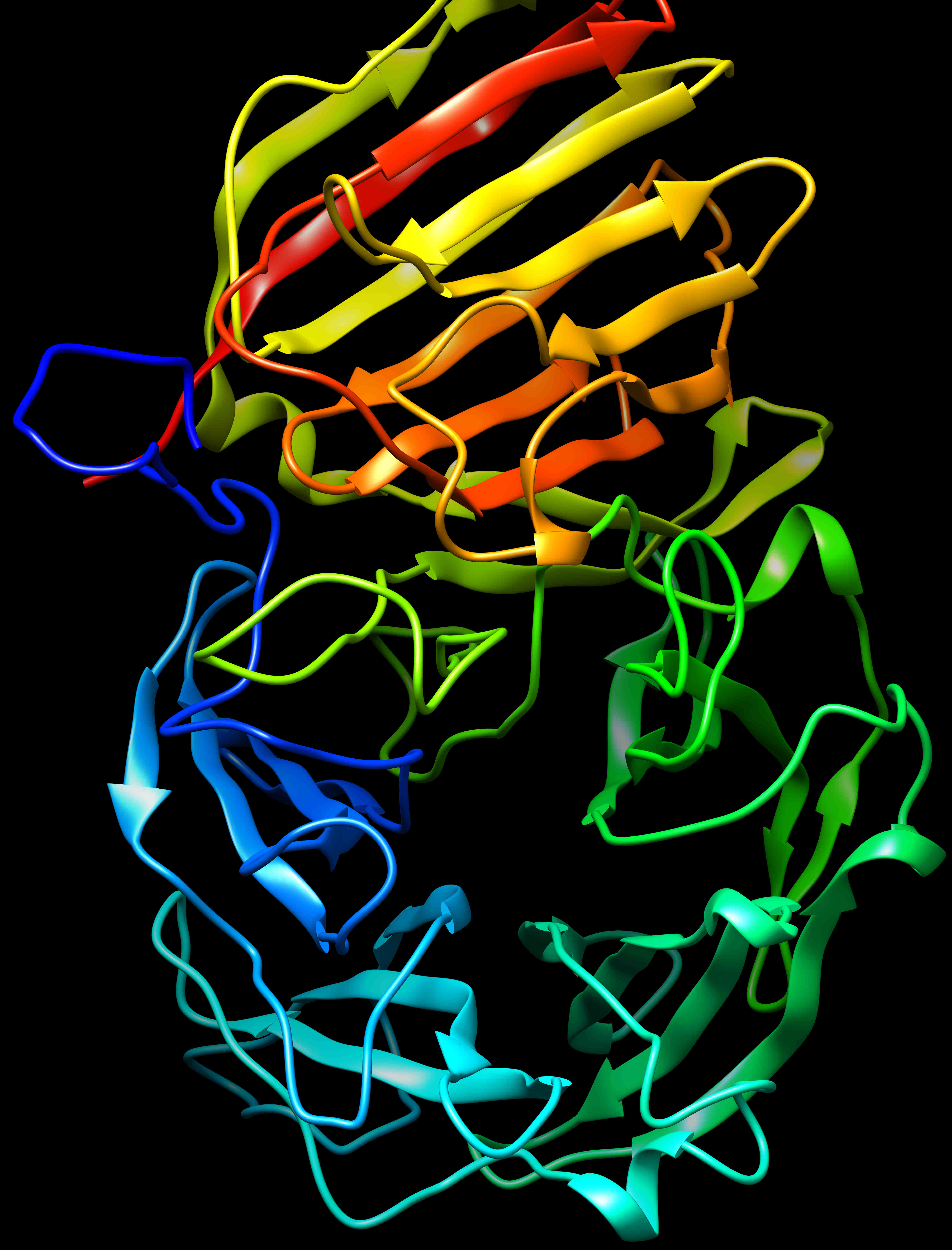

Supplement: S2 Dataset — (ZIP) [file pone.0200607.s002.zip › Abinitio_Models/ACP1.jpg]

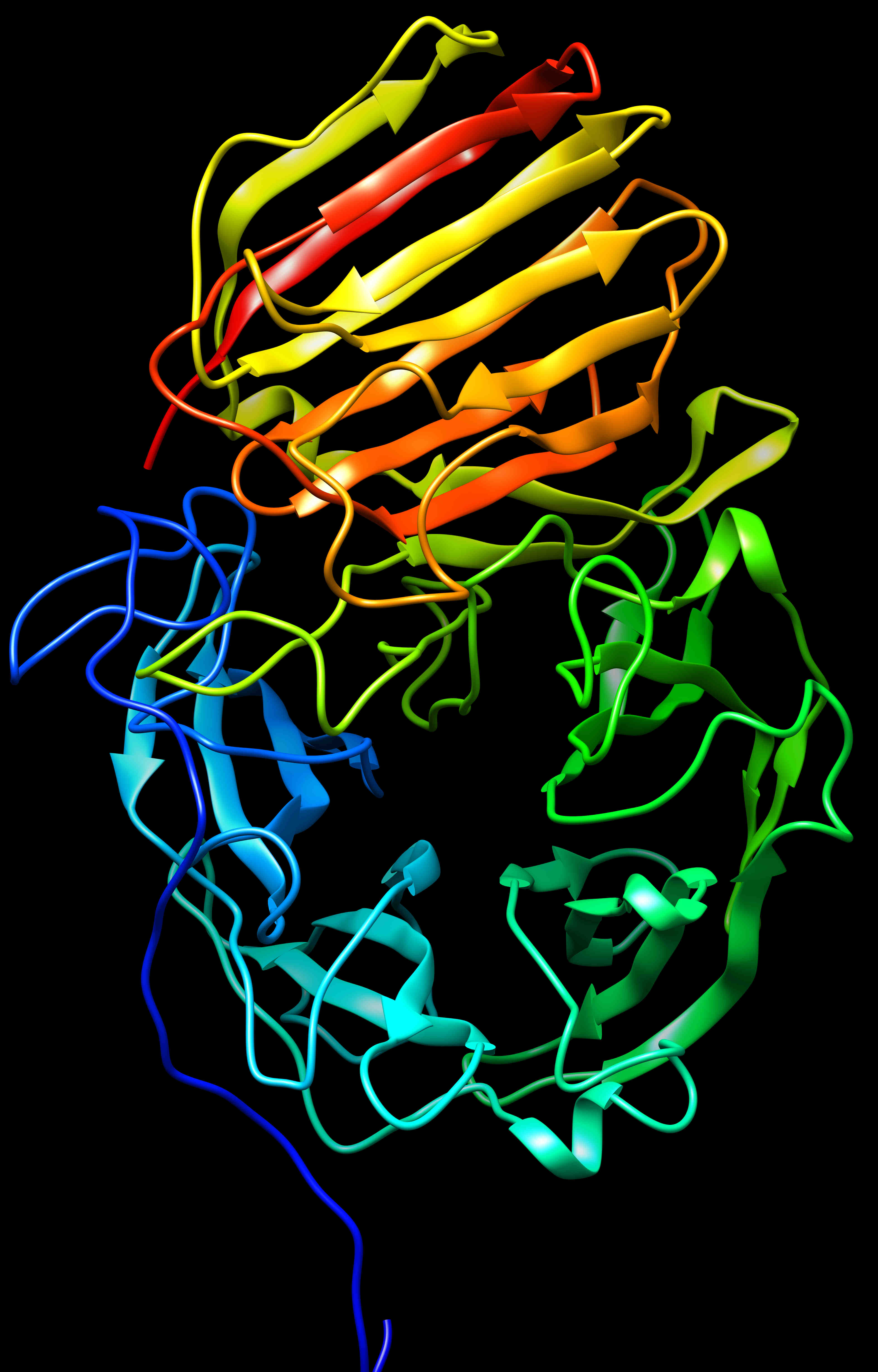

Supplement: S2 Dataset — (ZIP) [file pone.0200607.s002.zip › Abinitio_Models/AFP1.jpg]

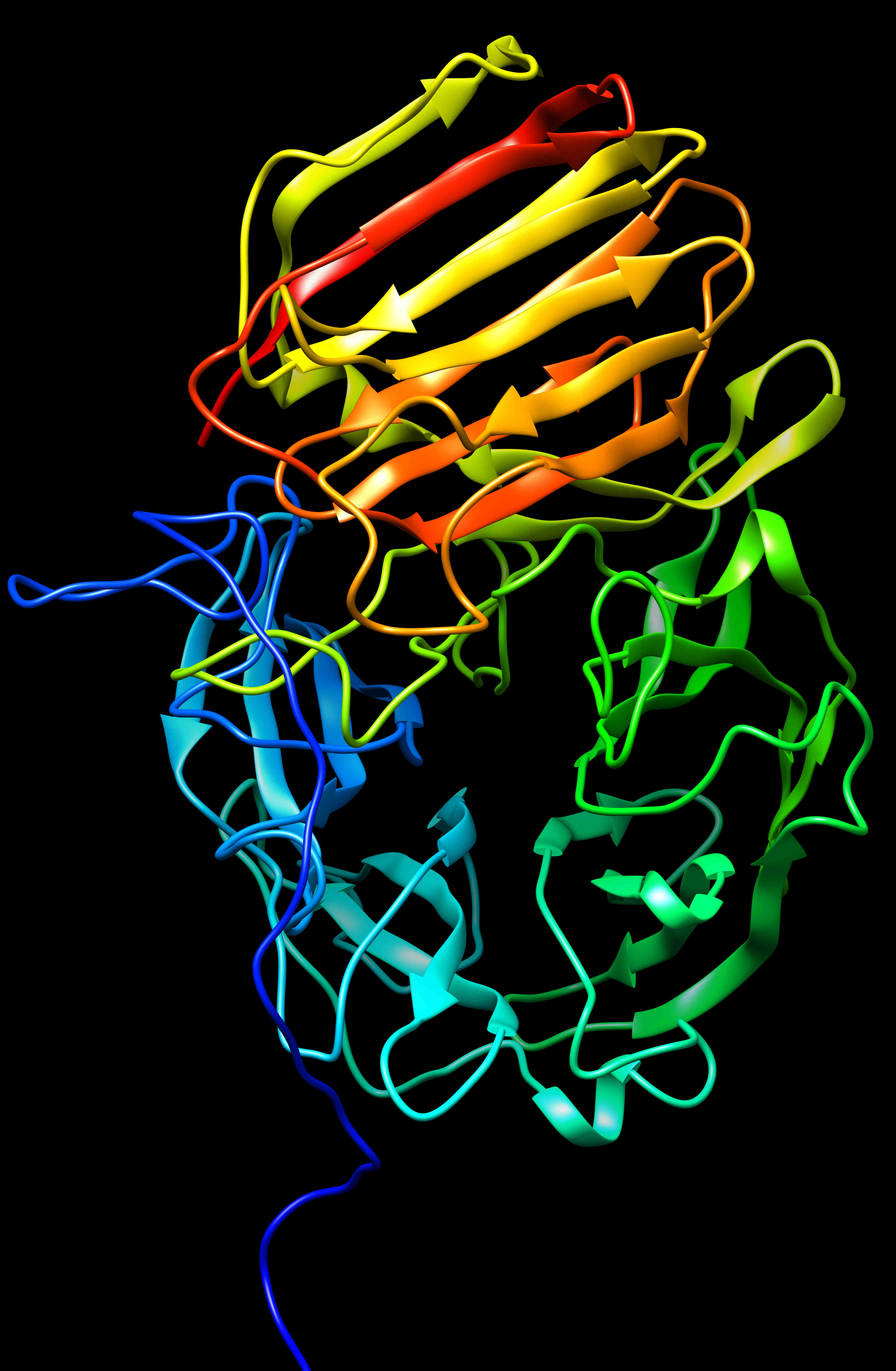

Supplement: S2 Dataset — (ZIP) [file pone.0200607.s002.zip › Abinitio_Models/AFP2.jpg]

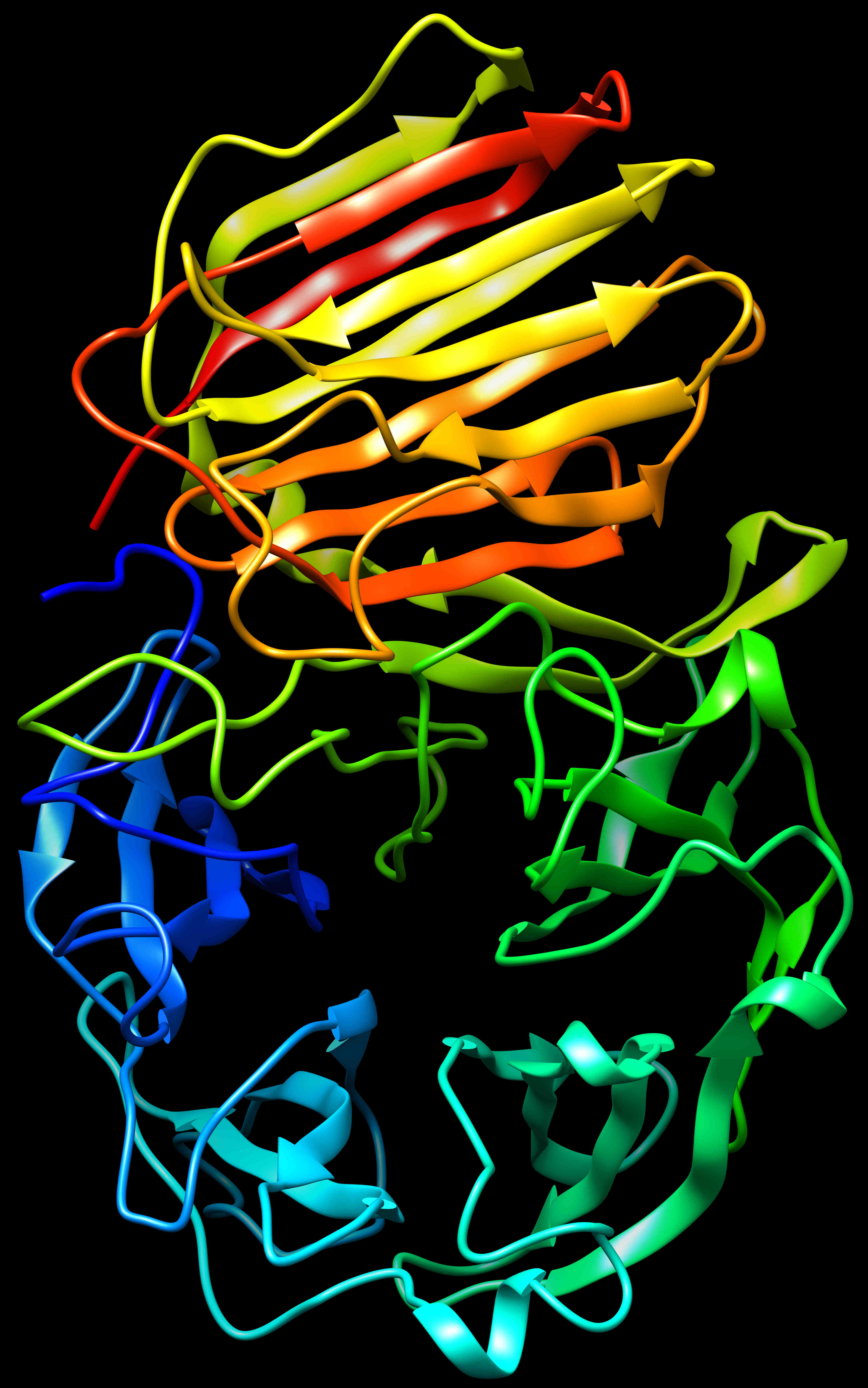

Supplement: S2 Dataset — (ZIP) [file pone.0200607.s002.zip › Abinitio_Models/AFSP1.jpg]

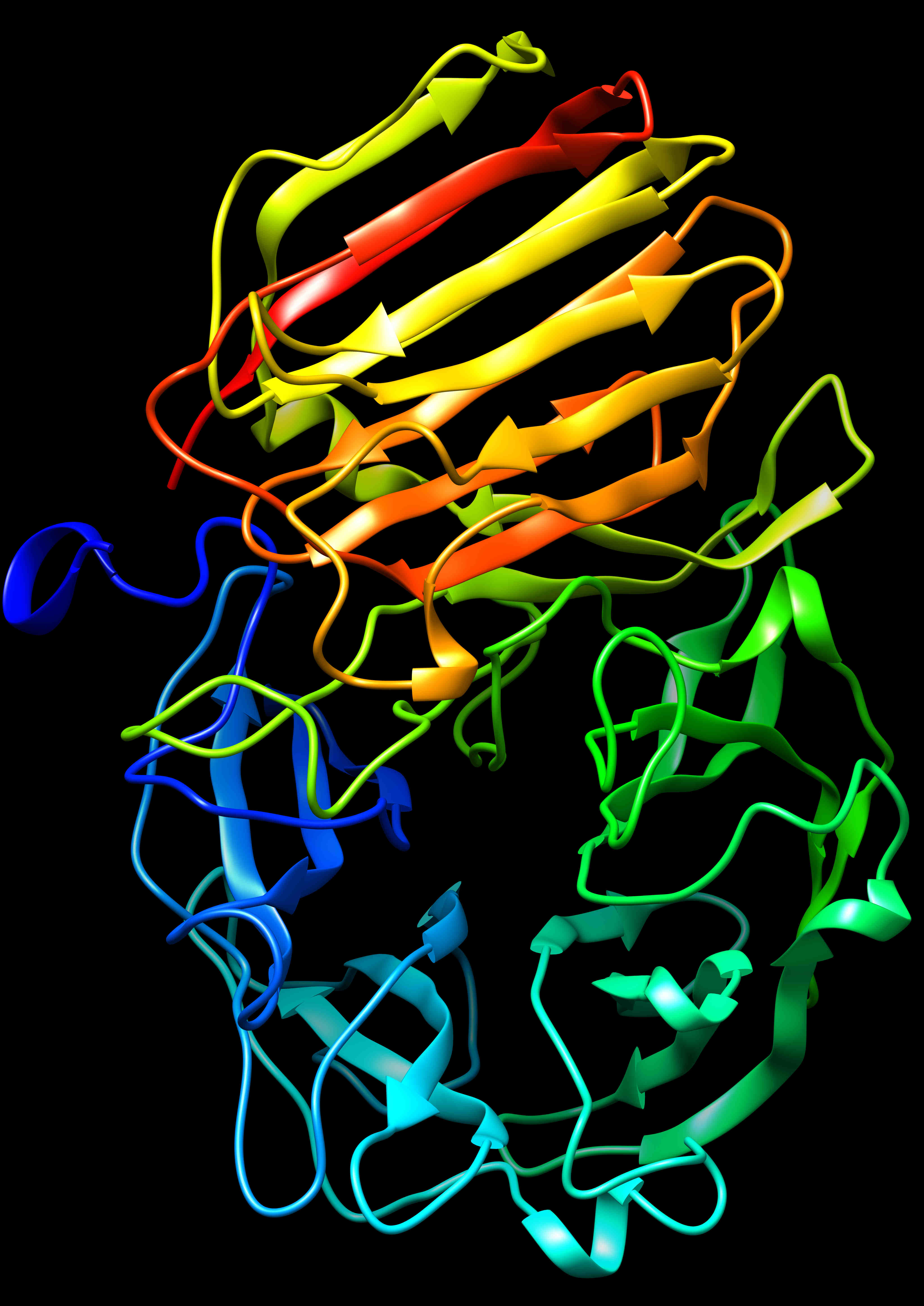

Supplement: S2 Dataset — (ZIP) [file pone.0200607.s002.zip › Abinitio_Models/ALP1.jpg]

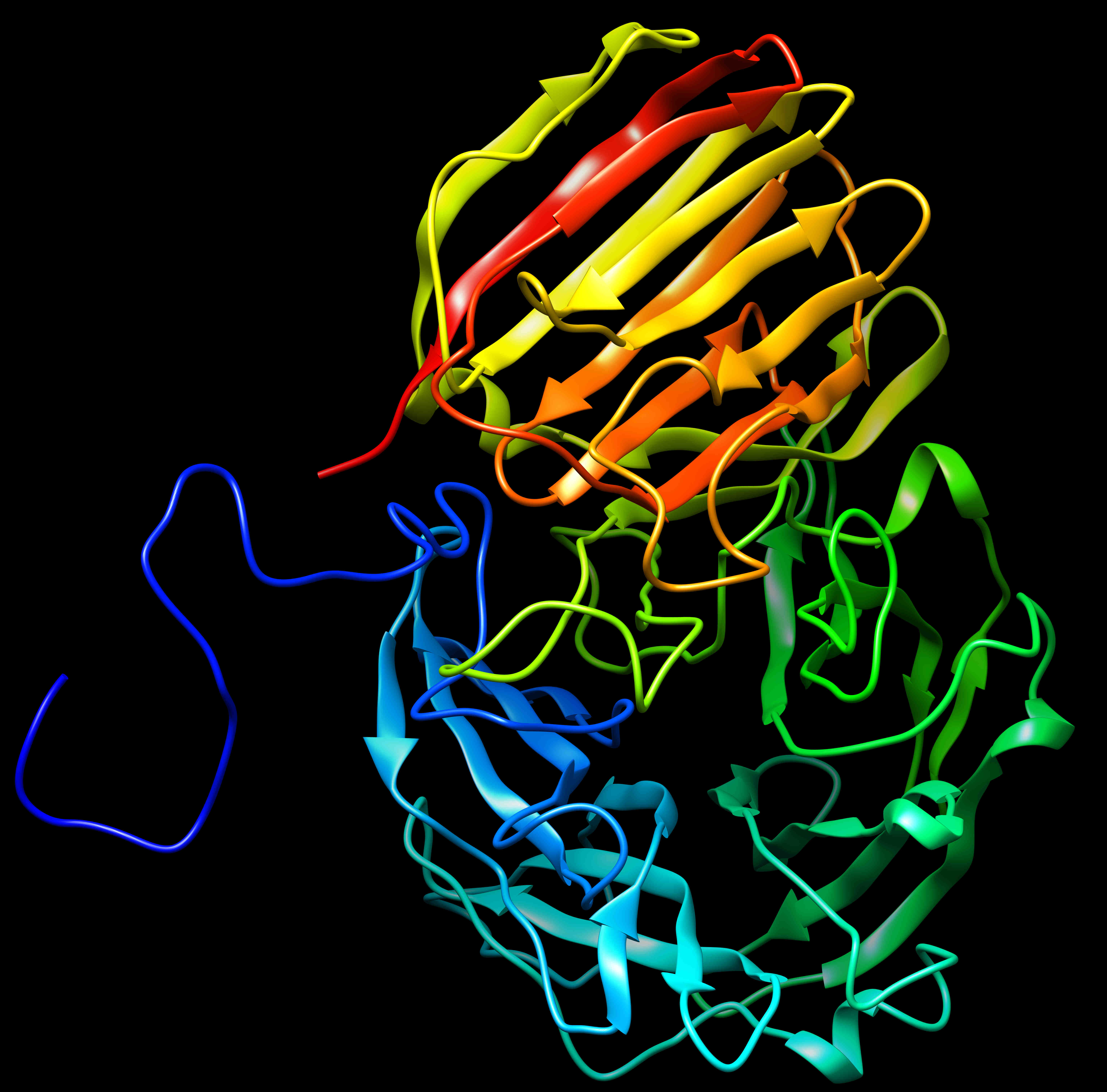

Supplement: S2 Dataset — (ZIP) [file pone.0200607.s002.zip › Abinitio_Models/ANP1.jpg]

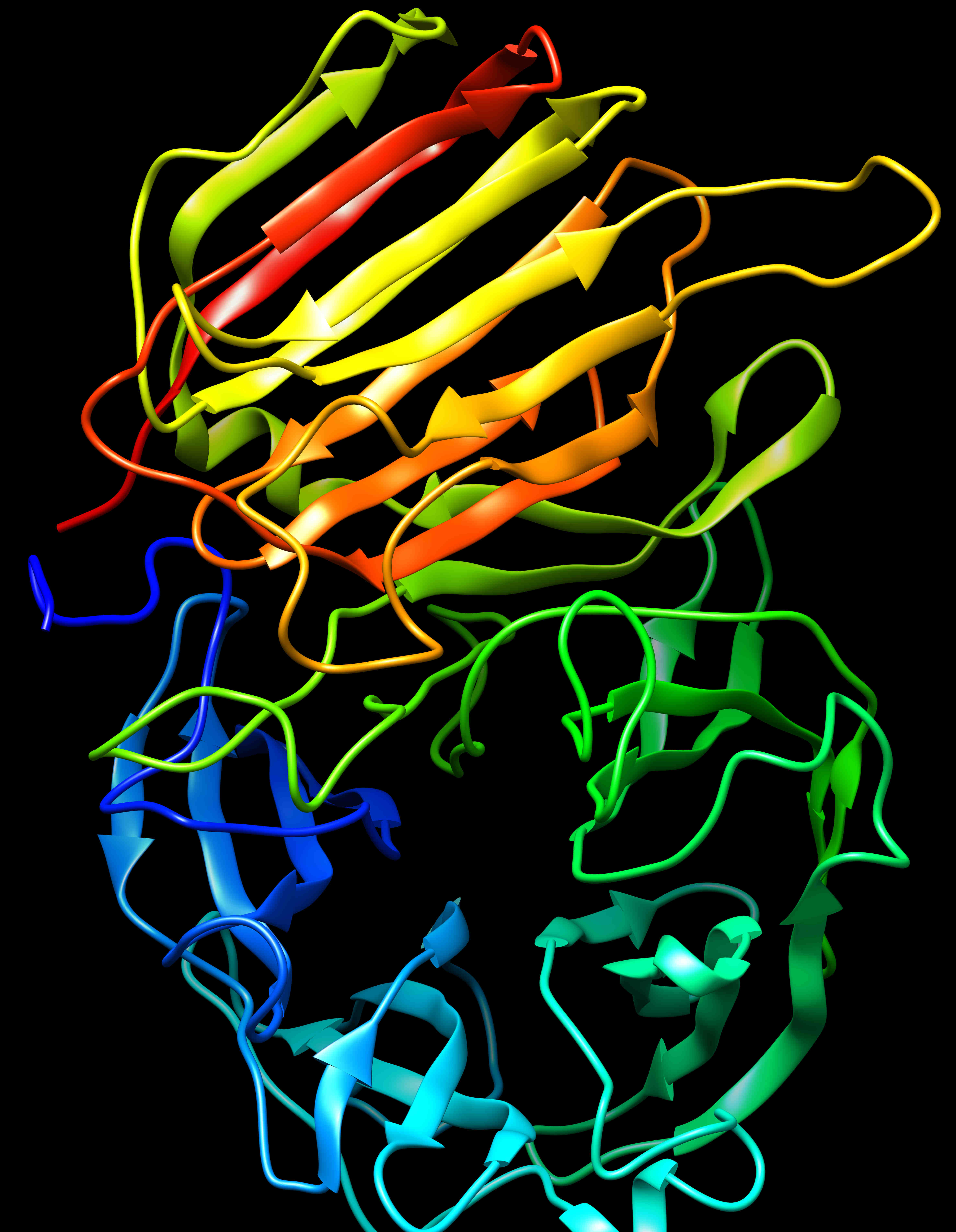

Supplement: S2 Dataset — (ZIP) [file pone.0200607.s002.zip › Abinitio_Models/ANP10.jpg]

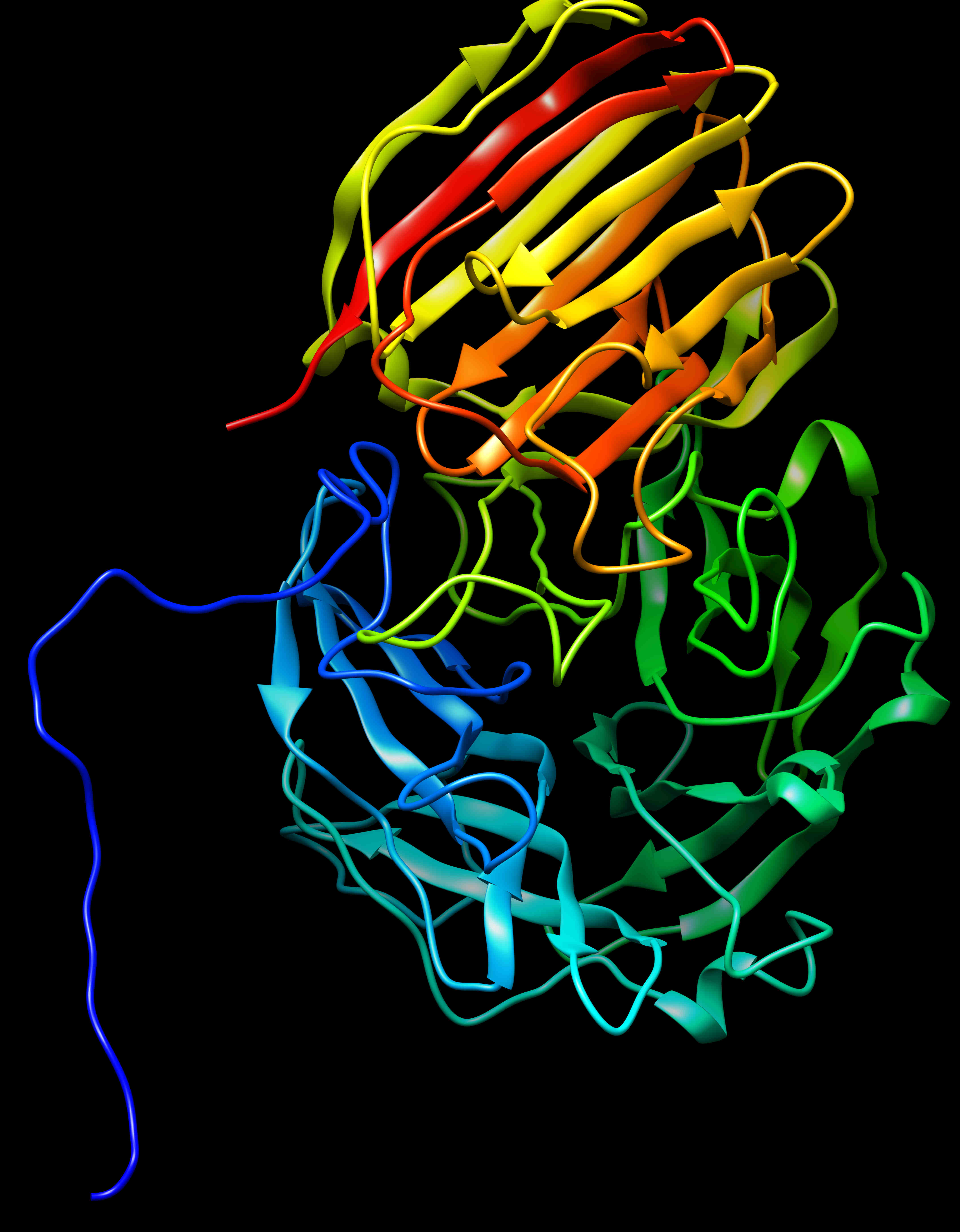

Supplement: S2 Dataset — (ZIP) [file pone.0200607.s002.zip › Abinitio_Models/ANP2.jpg]

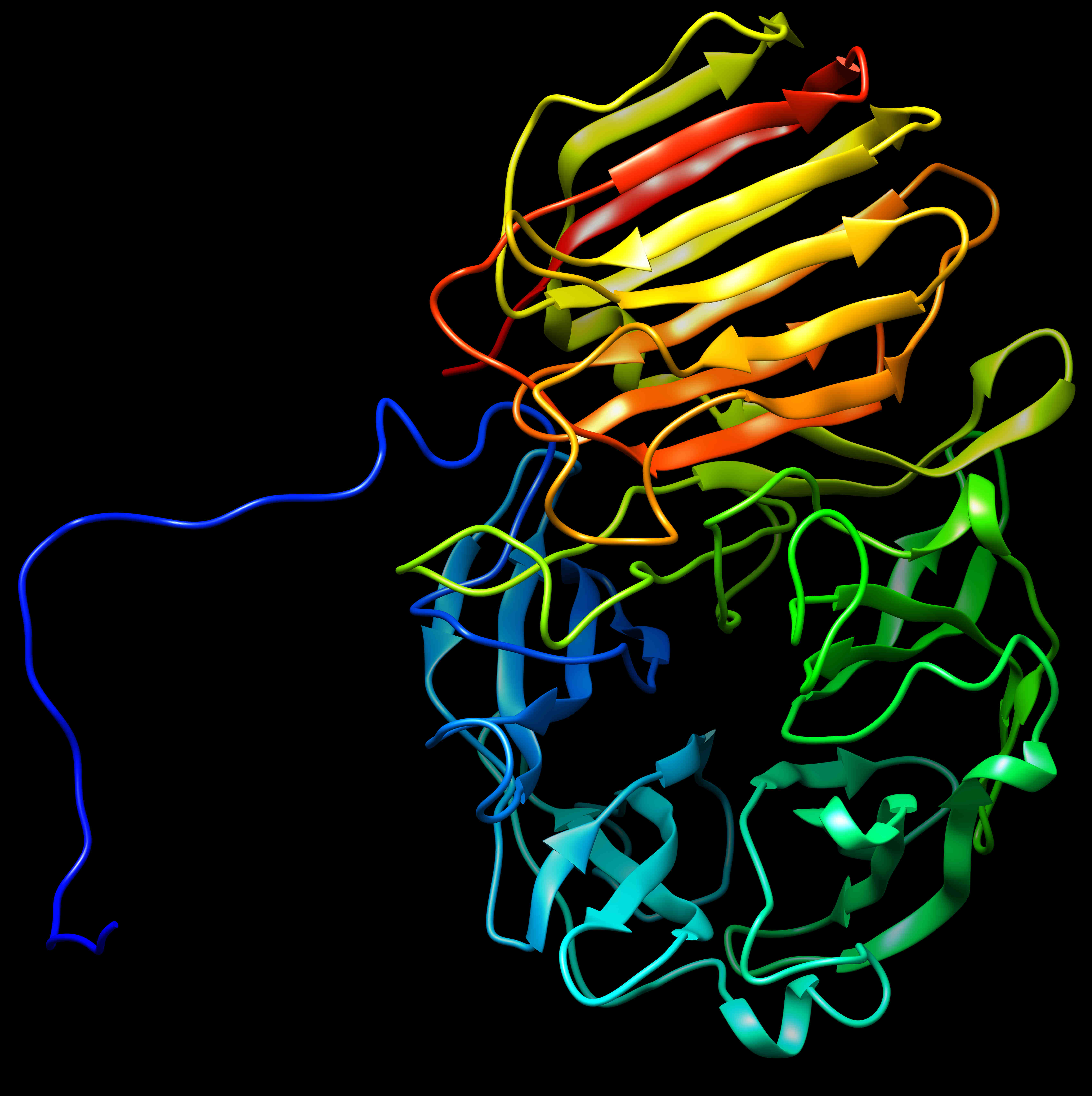

Supplement: S2 Dataset — (ZIP) [file pone.0200607.s002.zip › Abinitio_Models/ANP3.jpg]

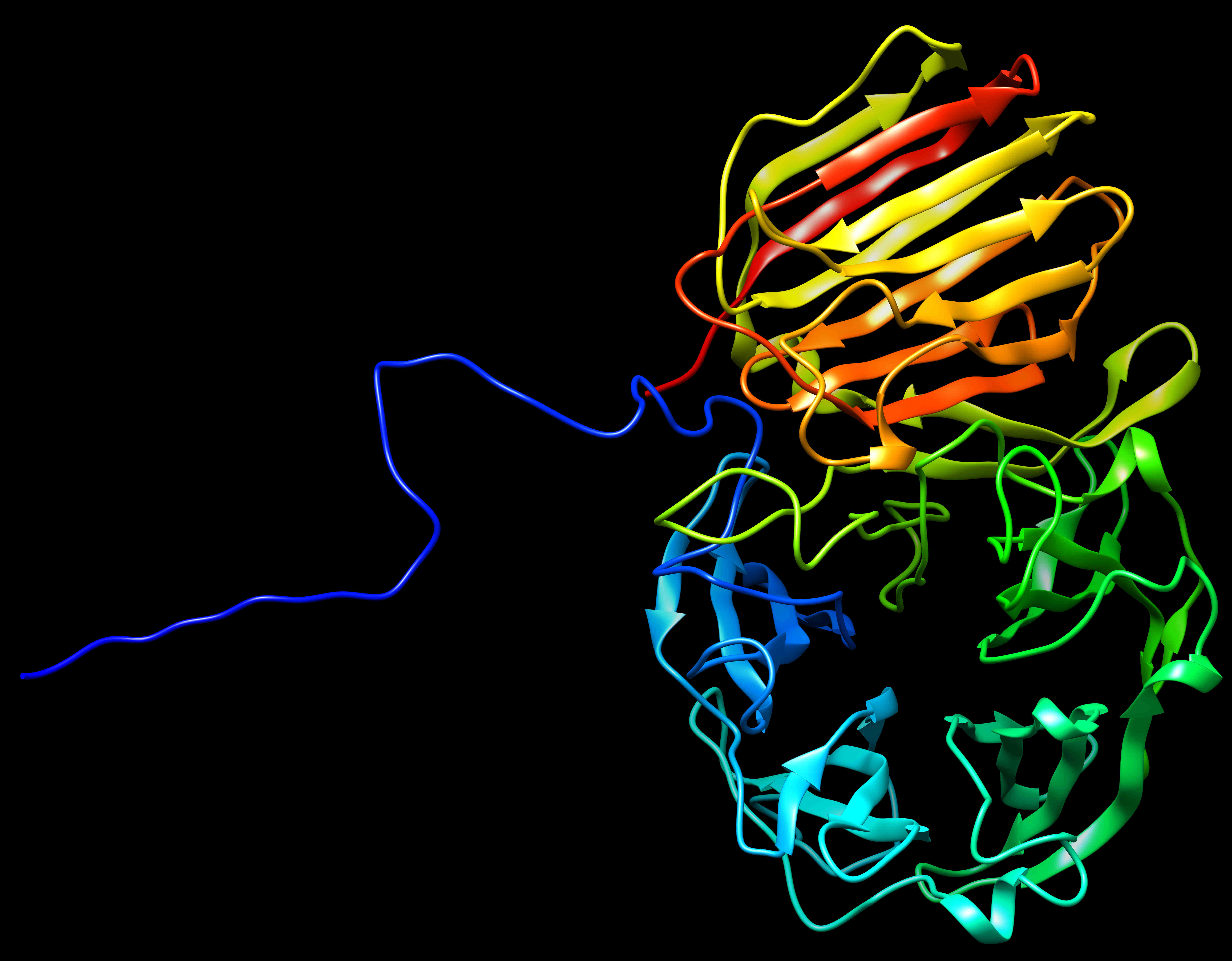

Supplement: S2 Dataset — (ZIP) [file pone.0200607.s002.zip › Abinitio_Models/ANP4.jpg]

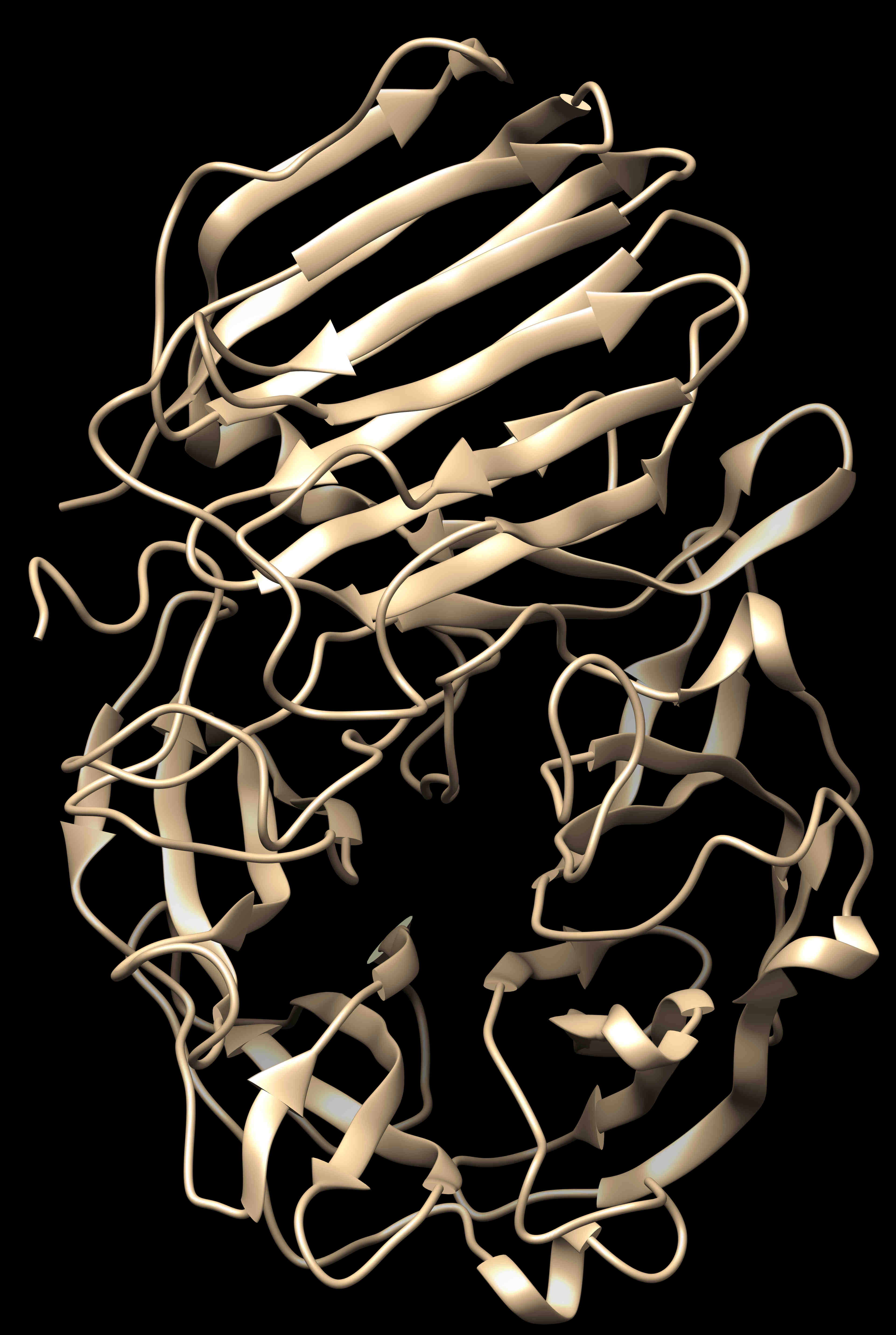

Supplement: S2 Dataset — (ZIP) [file pone.0200607.s002.zip › Abinitio_Models/ANP5.jpg]

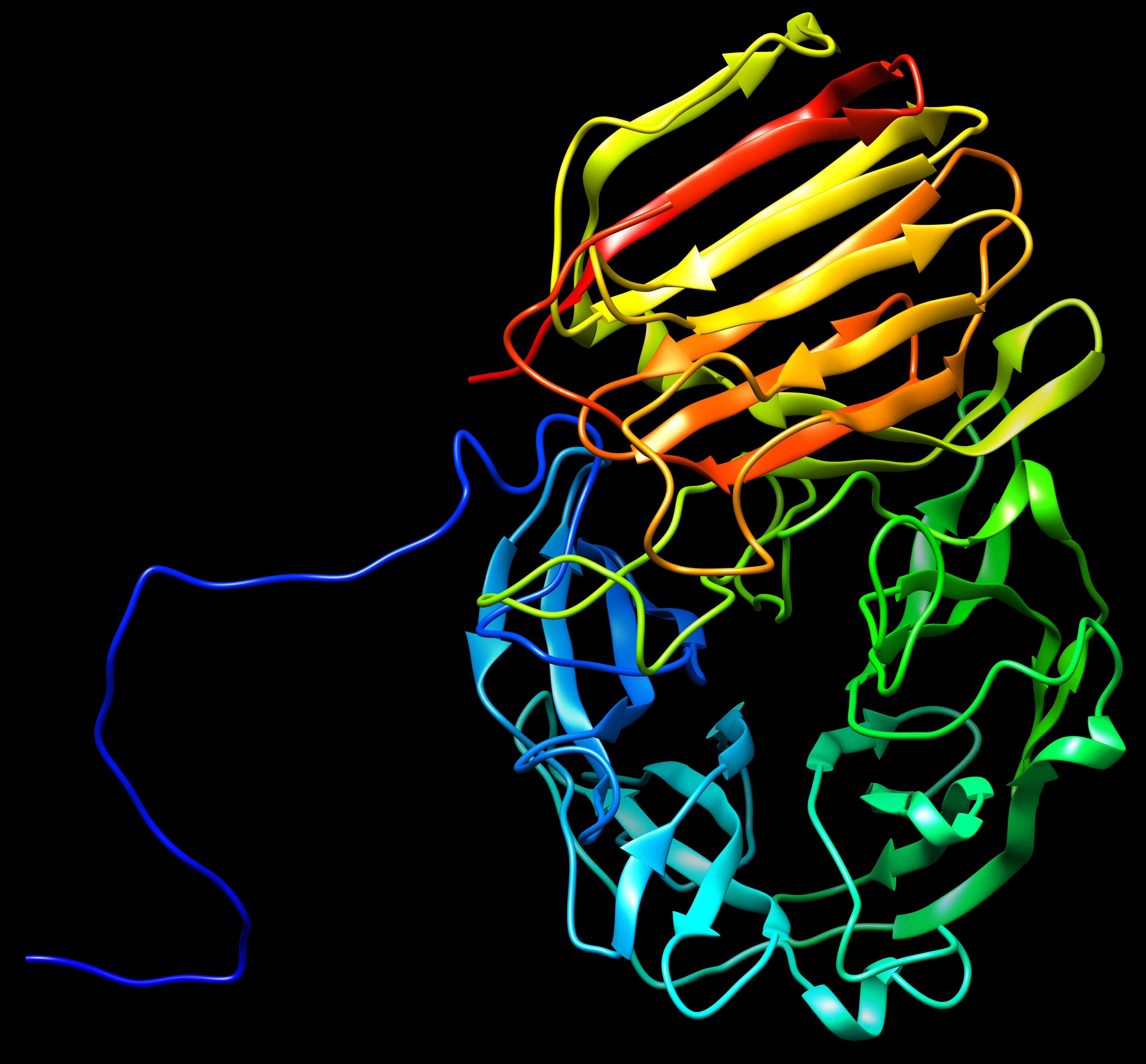

Supplement: S2 Dataset — (ZIP) [file pone.0200607.s002.zip › Abinitio_Models/ANP7.jpg]

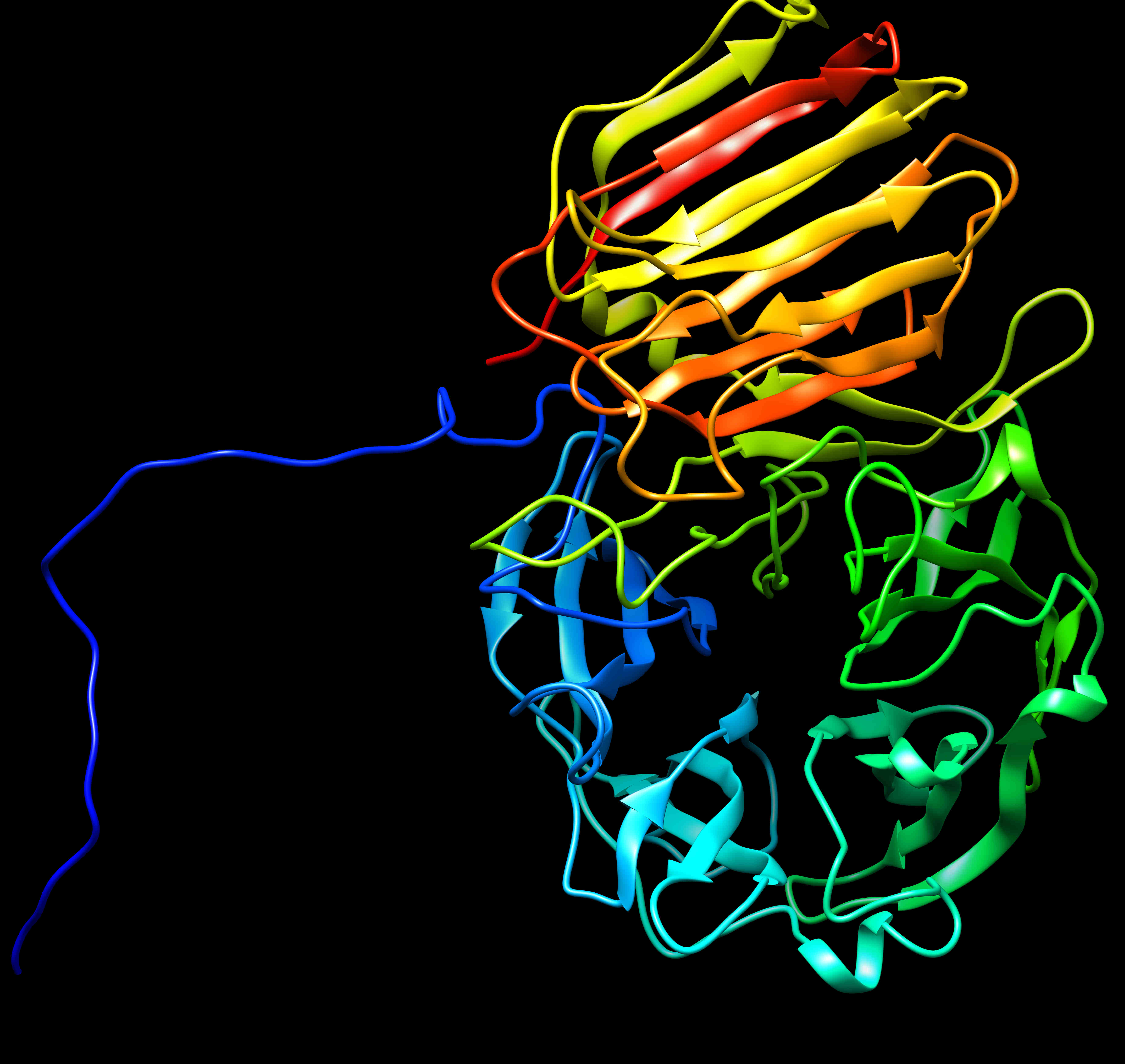

Supplement: S2 Dataset — (ZIP) [file pone.0200607.s002.zip › Abinitio_Models/ANP9.jpg]

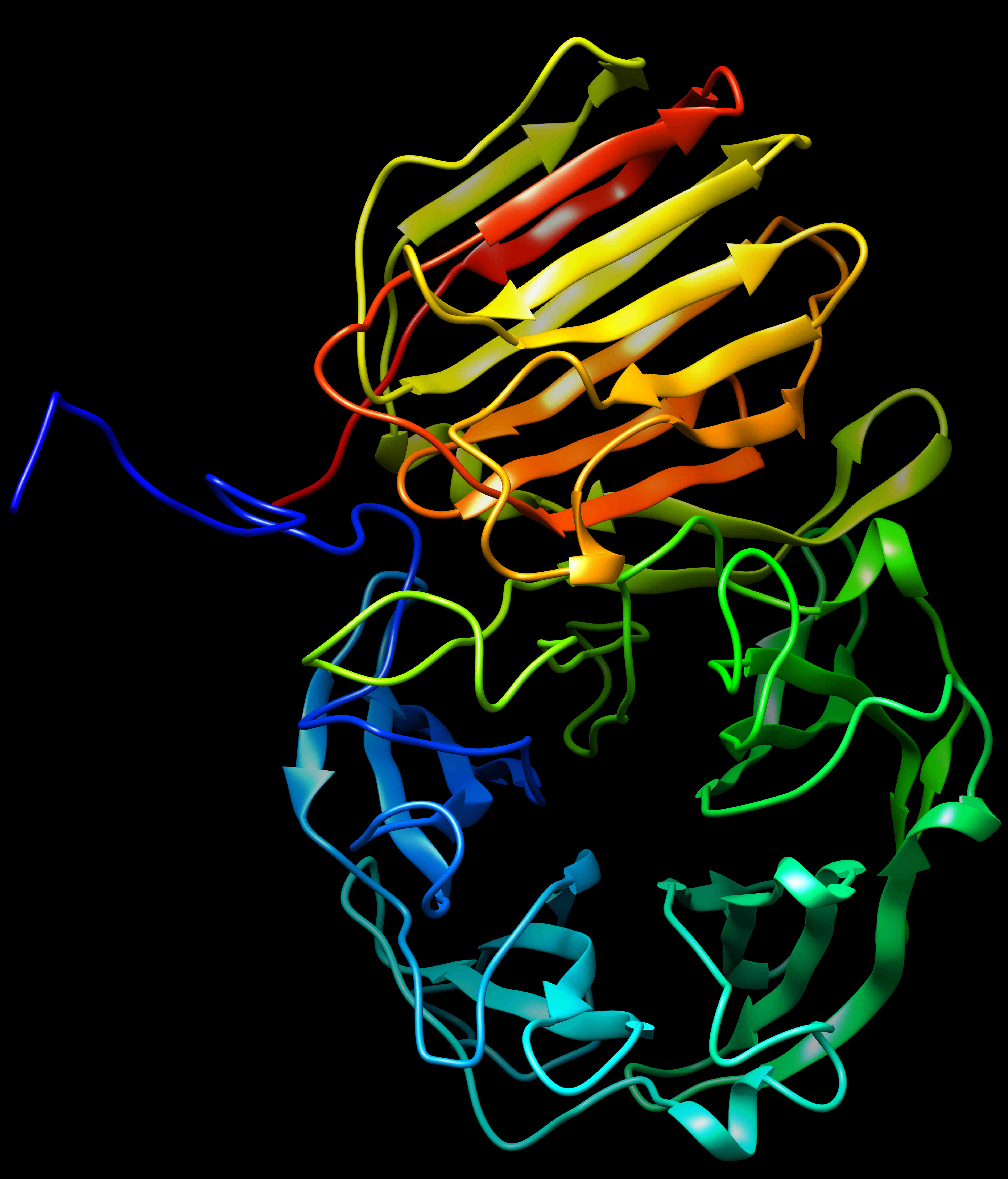

Supplement: S2 Dataset — (ZIP) [file pone.0200607.s002.zip › Abinitio_Models/ARP1.jpg]

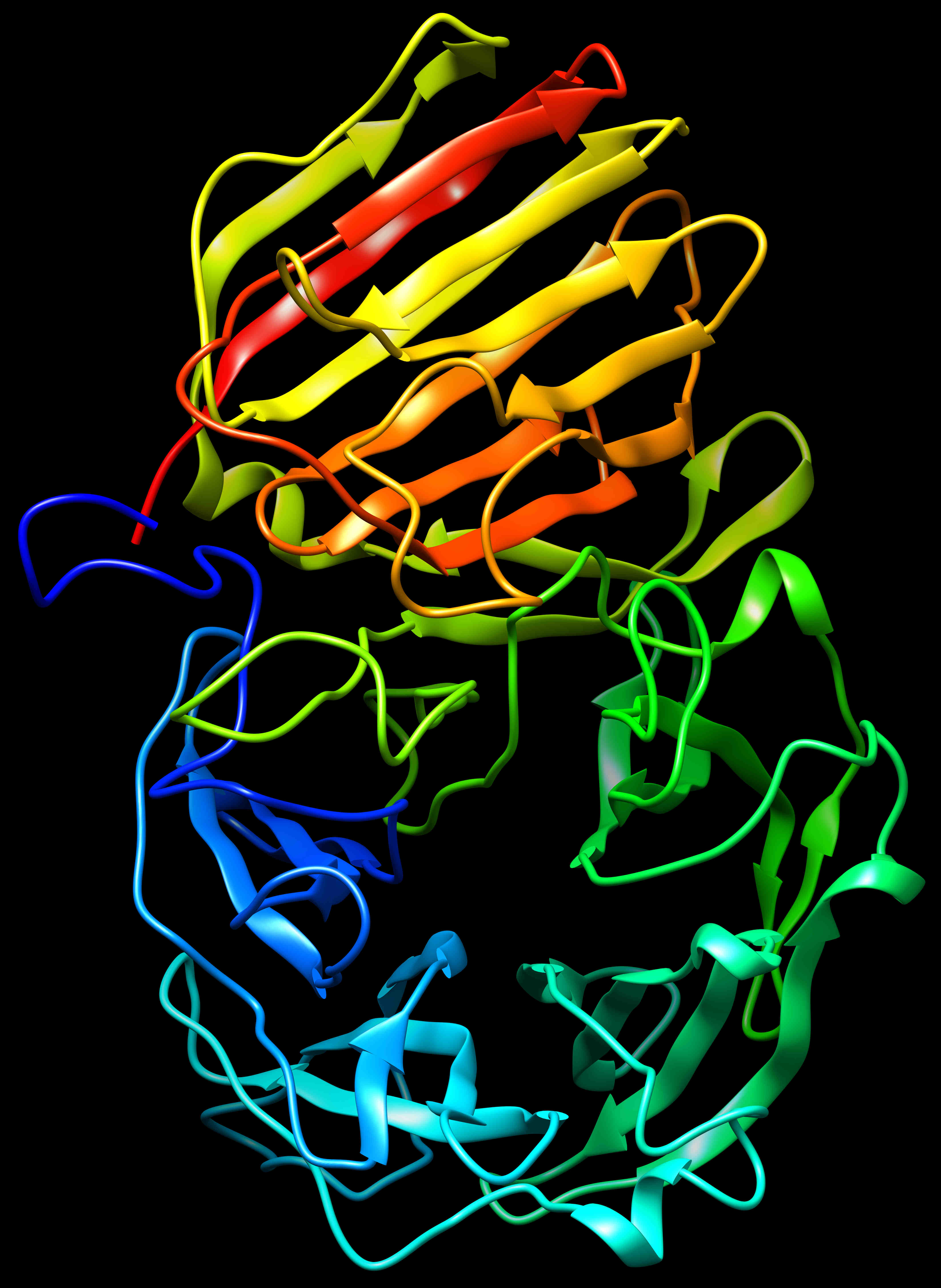

Supplement: S2 Dataset — (ZIP) [file pone.0200607.s002.zip › Abinitio_Models/BCP1.jpg]

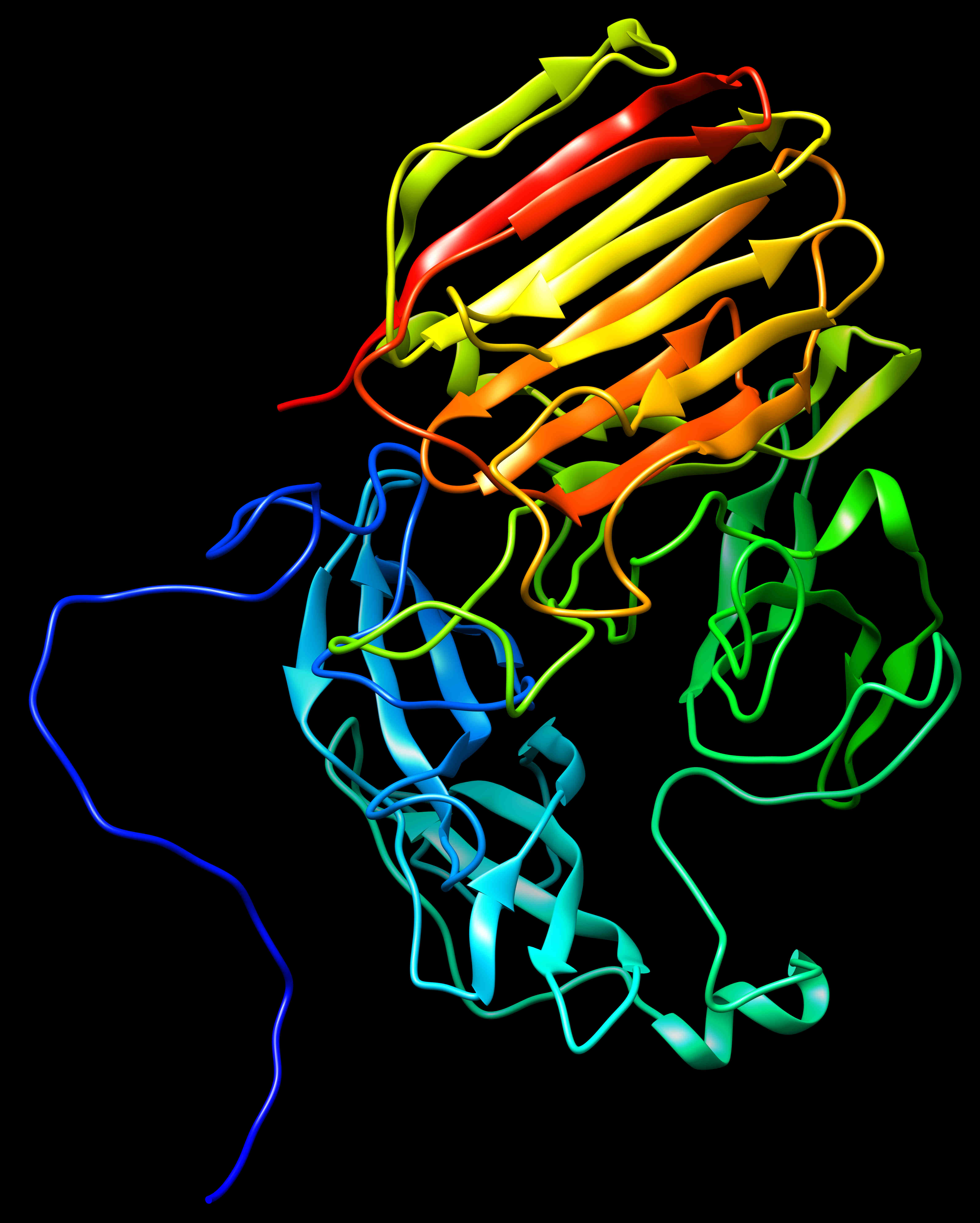

Supplement: S2 Dataset — (ZIP) [file pone.0200607.s002.zip › Abinitio_Models/BCP2.jpg]

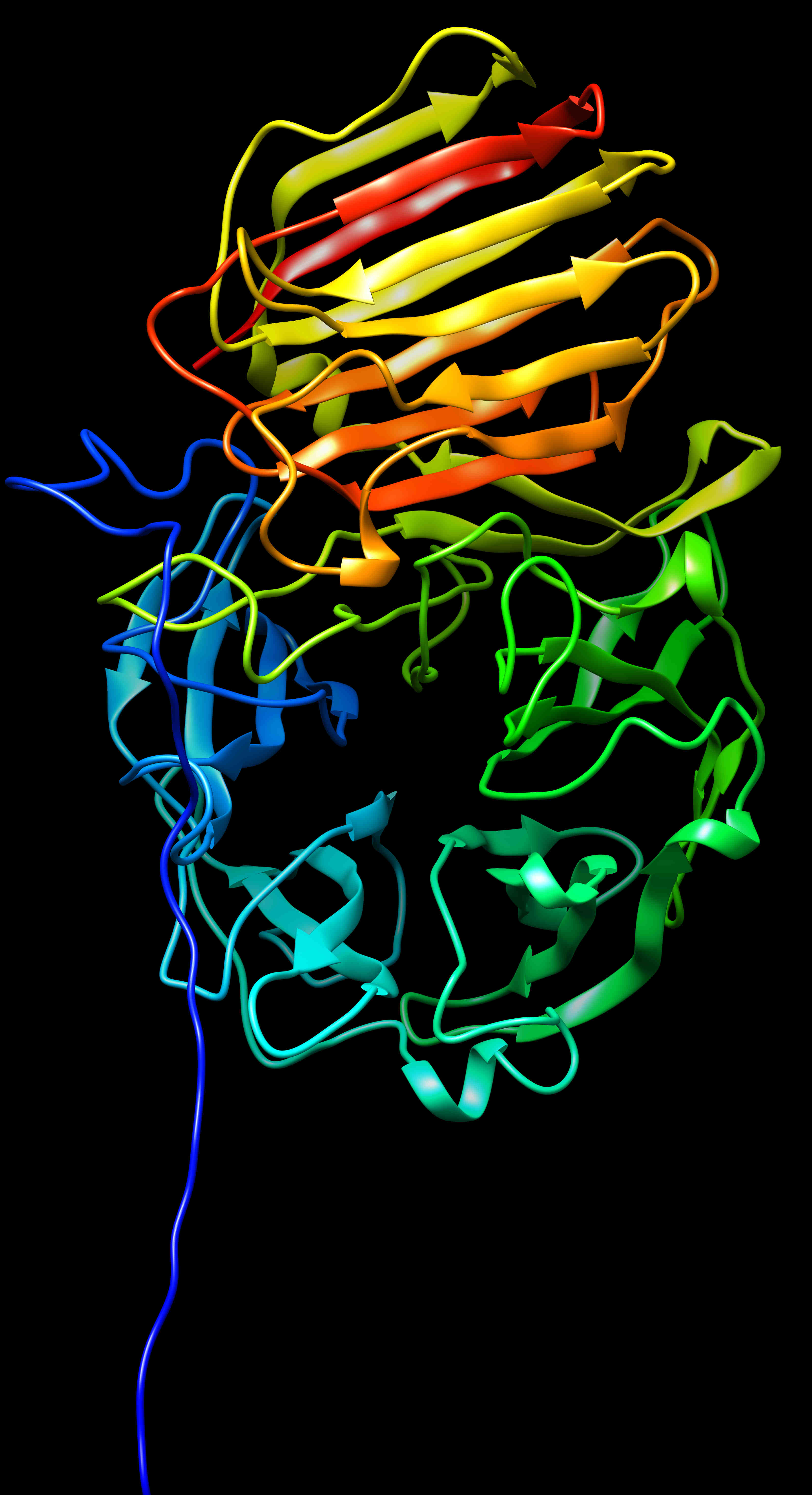

Supplement: S2 Dataset — (ZIP) [file pone.0200607.s002.zip › Abinitio_Models/FOHP1.jpg]

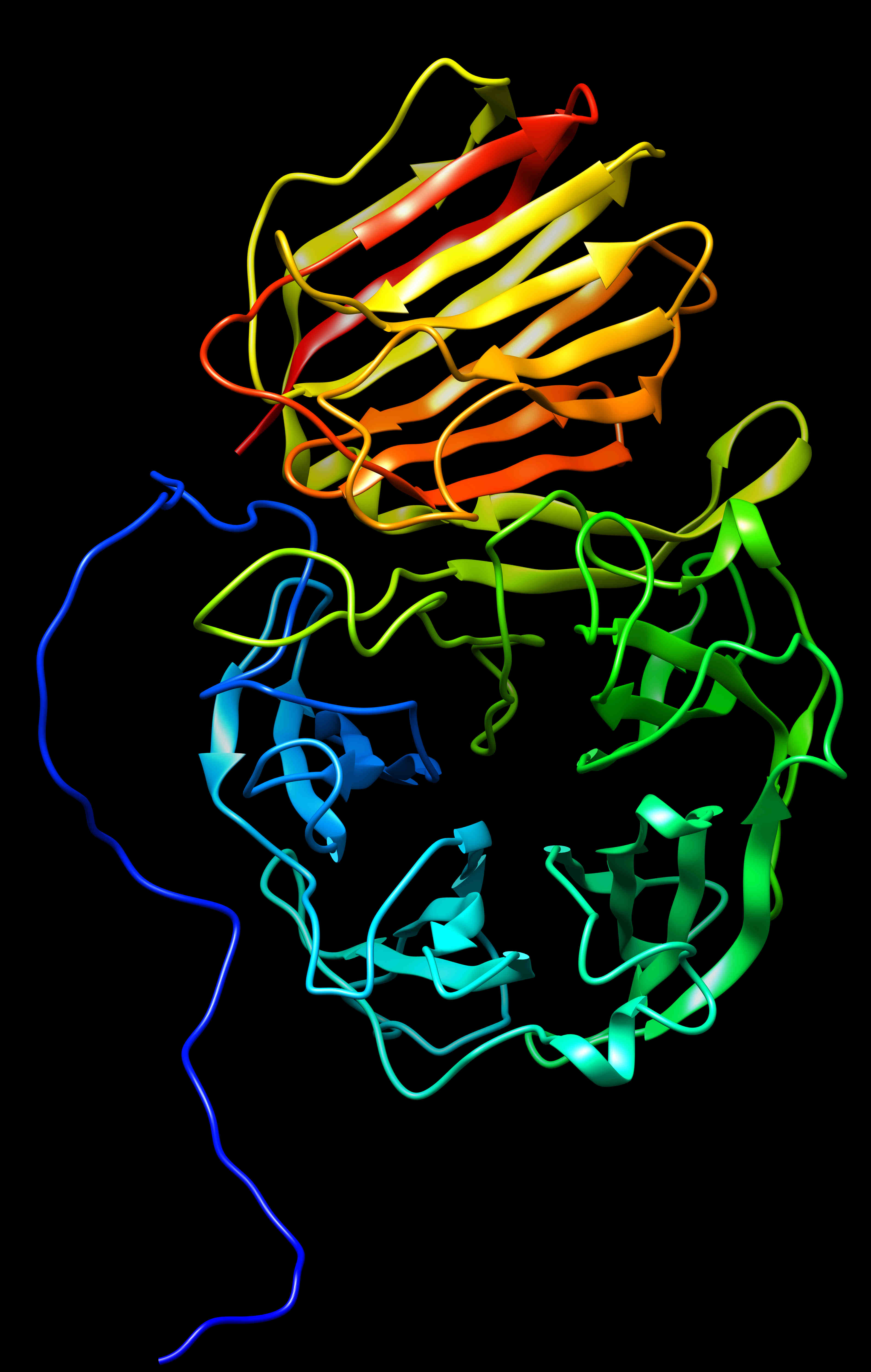

Supplement: S2 Dataset — (ZIP) [file pone.0200607.s002.zip › Abinitio_Models/FOHP2.jpg]

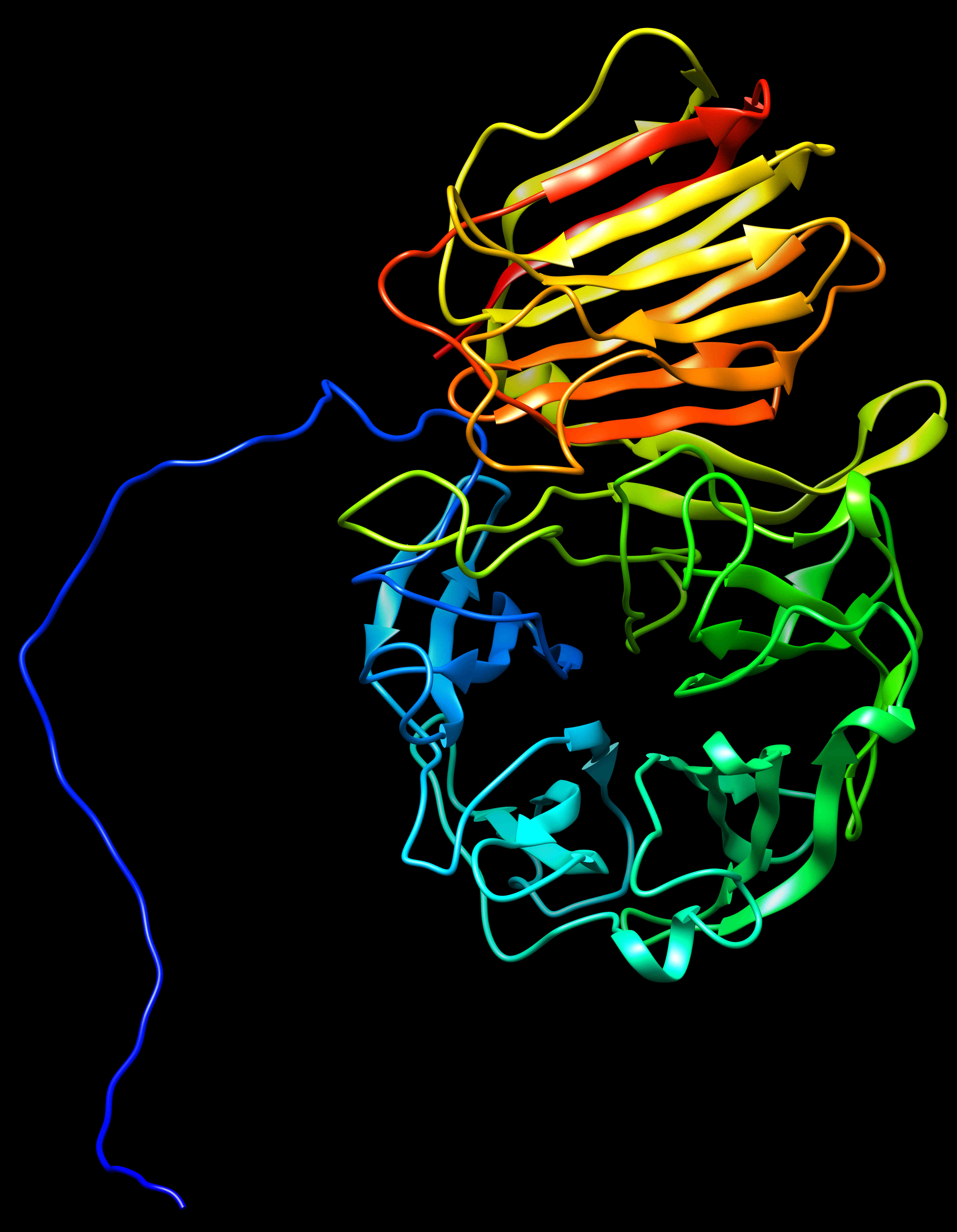

Supplement: S2 Dataset — (ZIP) [file pone.0200607.s002.zip › Abinitio_Models/FOP1.jpg]

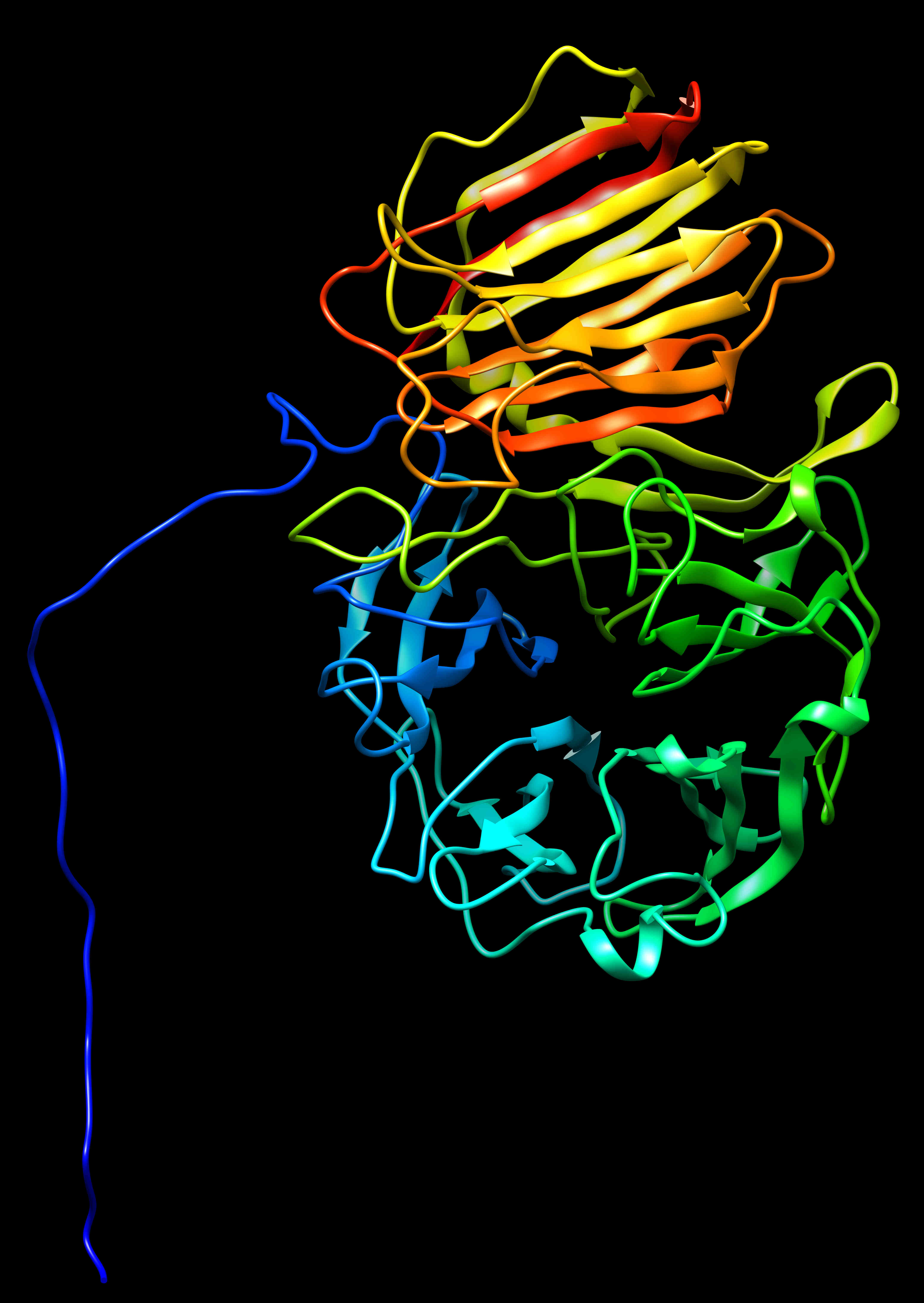

Supplement: S2 Dataset — (ZIP) [file pone.0200607.s002.zip › Abinitio_Models/FOP10.jpg]

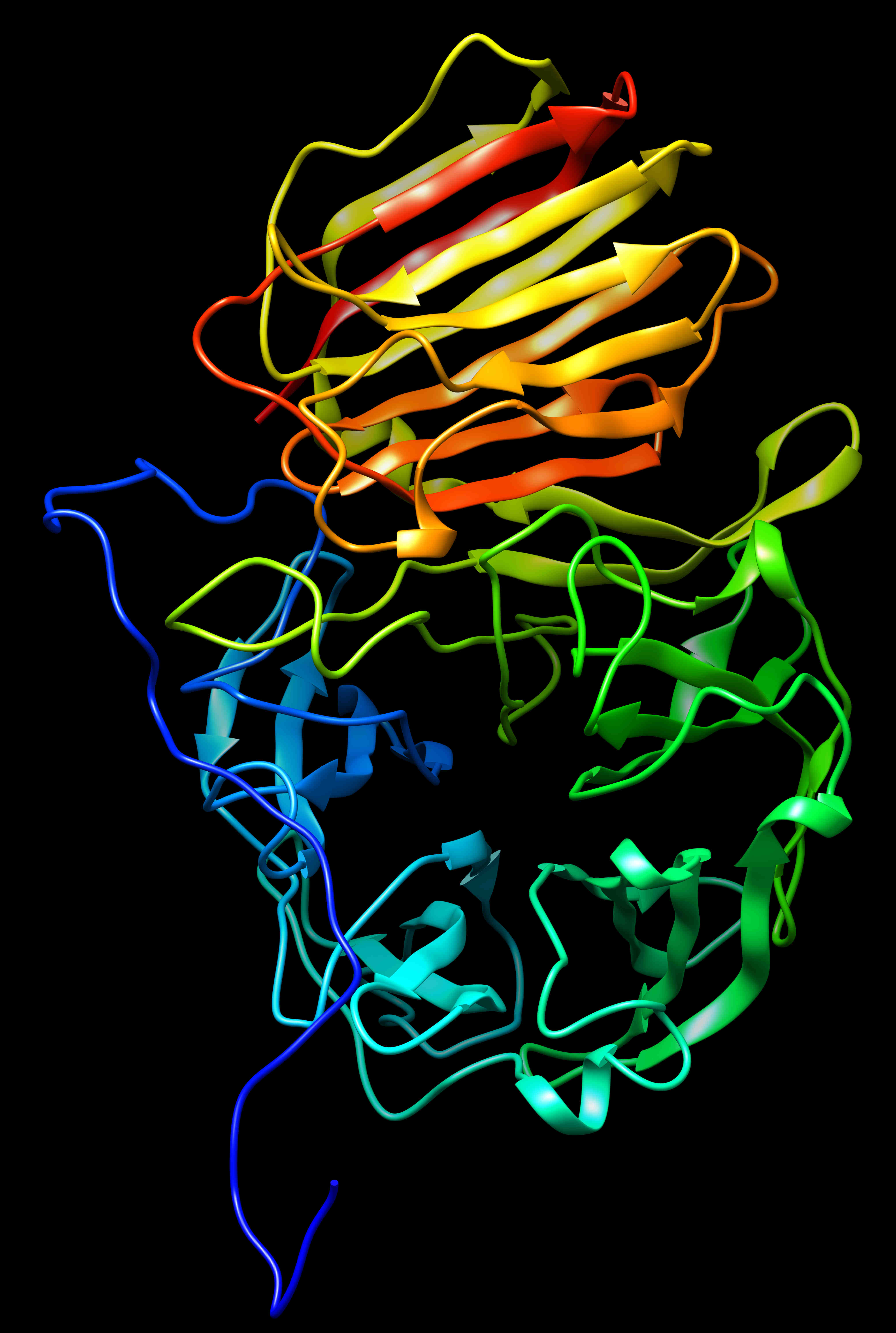

Supplement: S2 Dataset — (ZIP) [file pone.0200607.s002.zip › Abinitio_Models/FOP11.jpg]

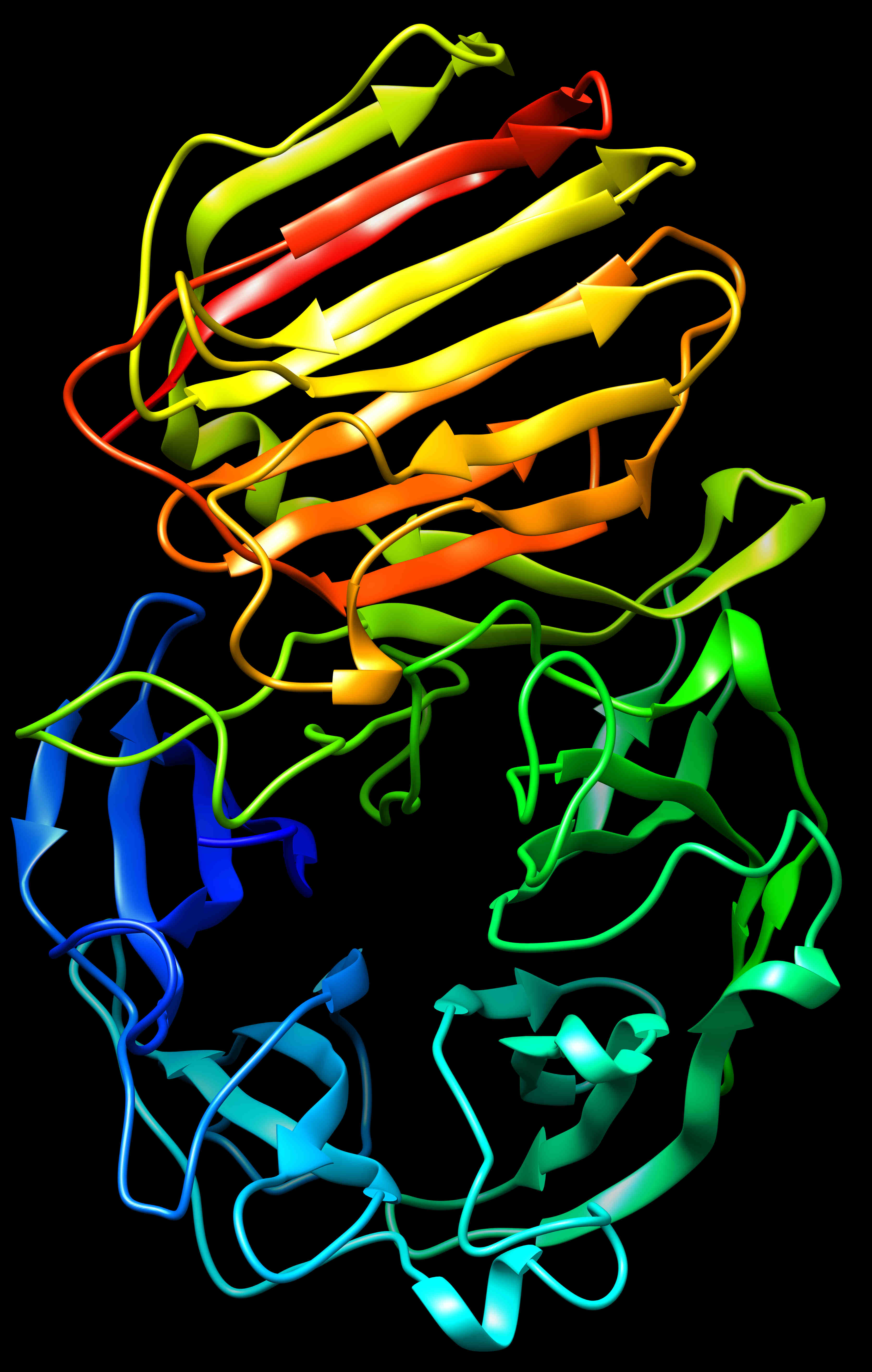

Supplement: S2 Dataset — (ZIP) [file pone.0200607.s002.zip › Abinitio_Models/FOP12.jpg]

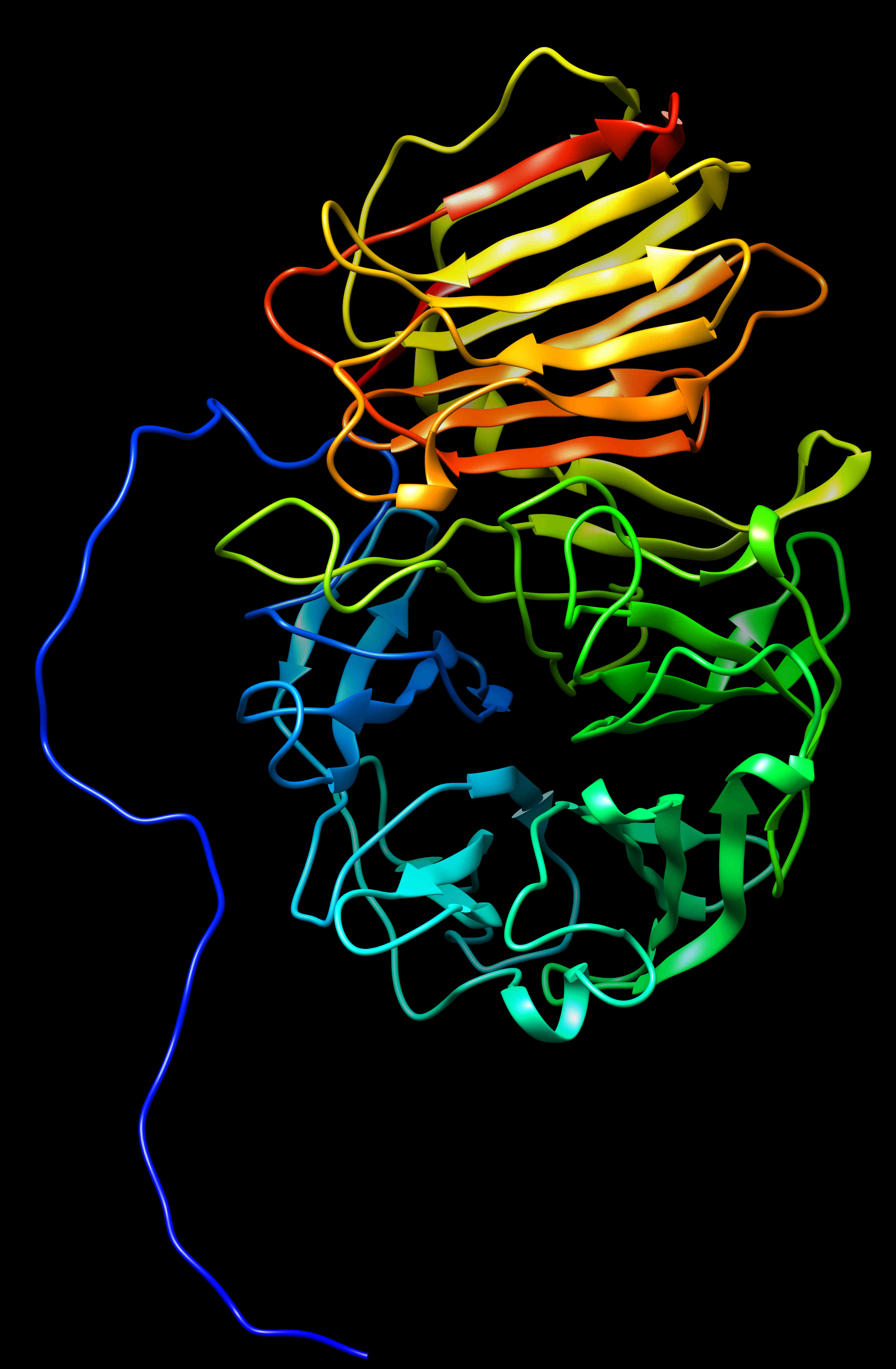

Supplement: S2 Dataset — (ZIP) [file pone.0200607.s002.zip › Abinitio_Models/FOP2.jpg]

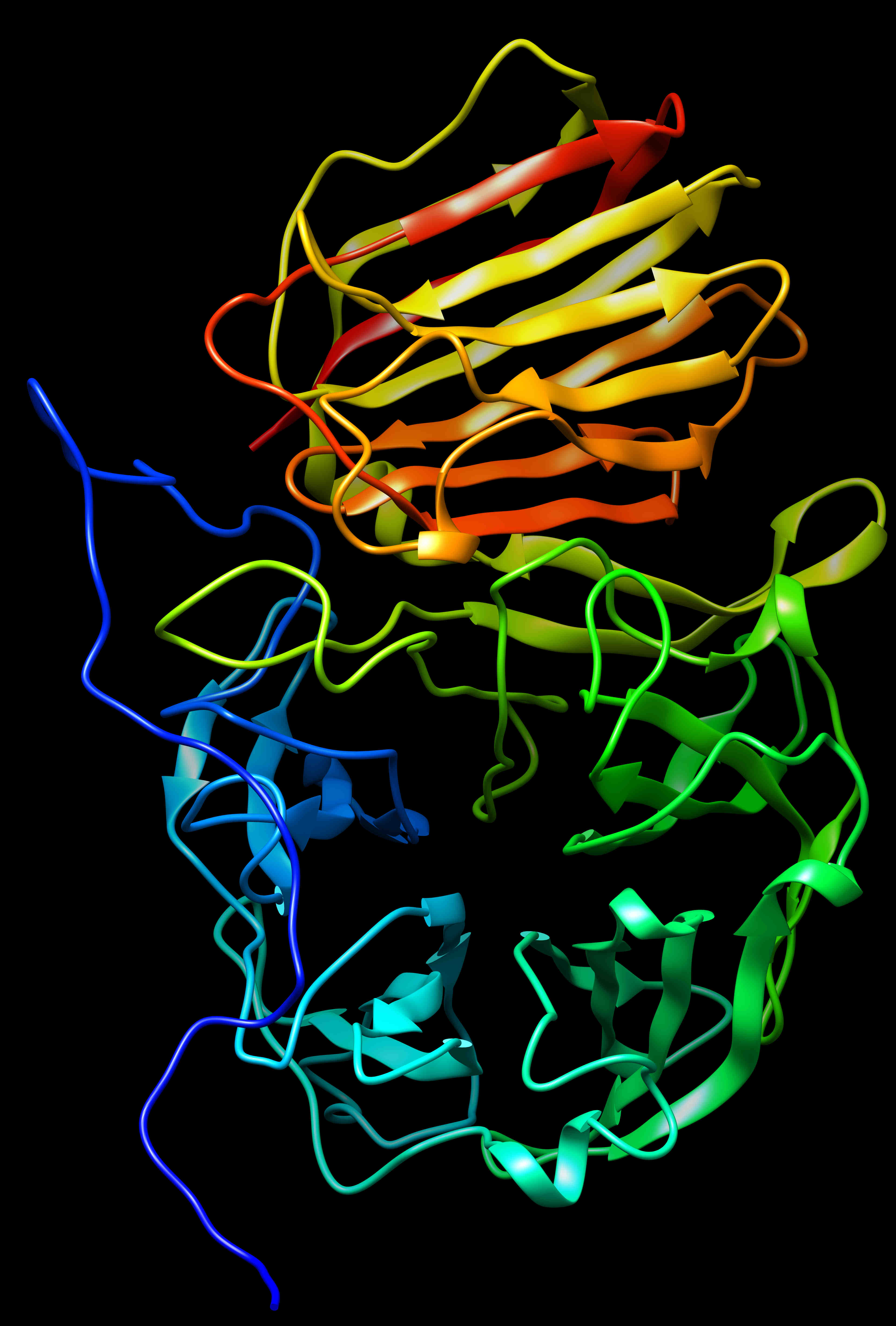

Supplement: S2 Dataset — (ZIP) [file pone.0200607.s002.zip › Abinitio_Models/FOP3.jpg]

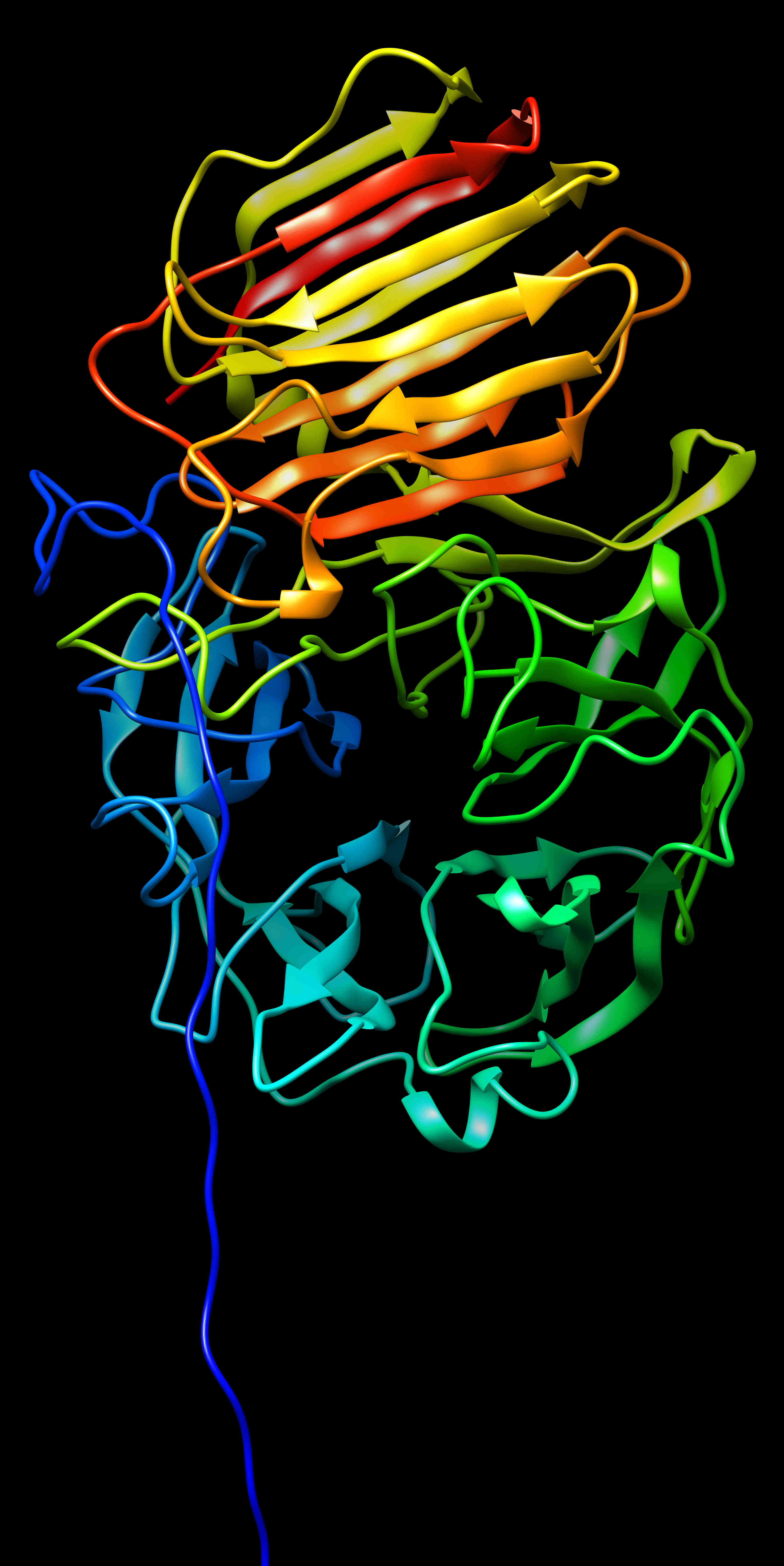

Supplement: S2 Dataset — (ZIP) [file pone.0200607.s002.zip › Abinitio_Models/FOP4.jpg]

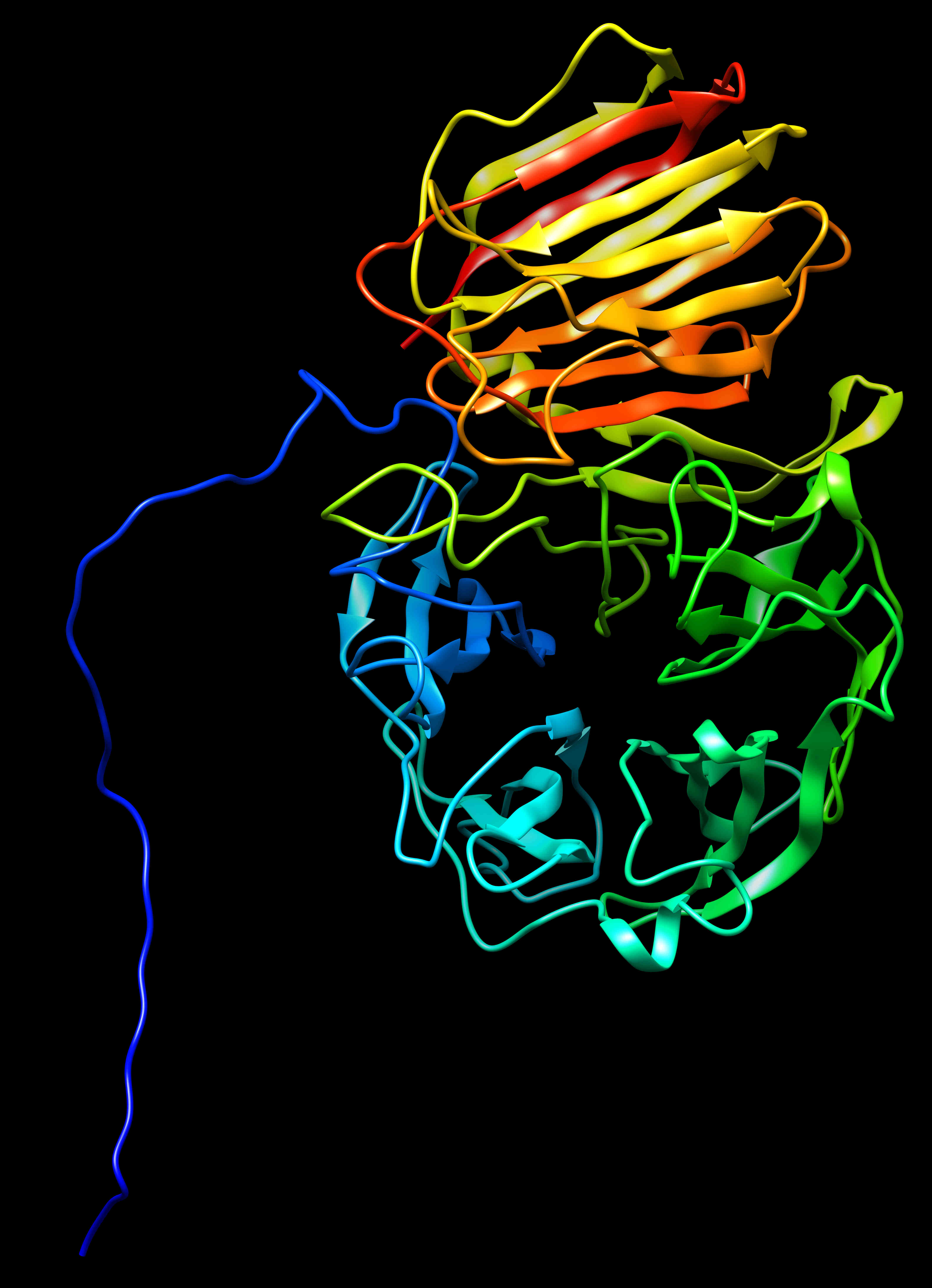

Supplement: S2 Dataset — (ZIP) [file pone.0200607.s002.zip › Abinitio_Models/FOP5.jpg]

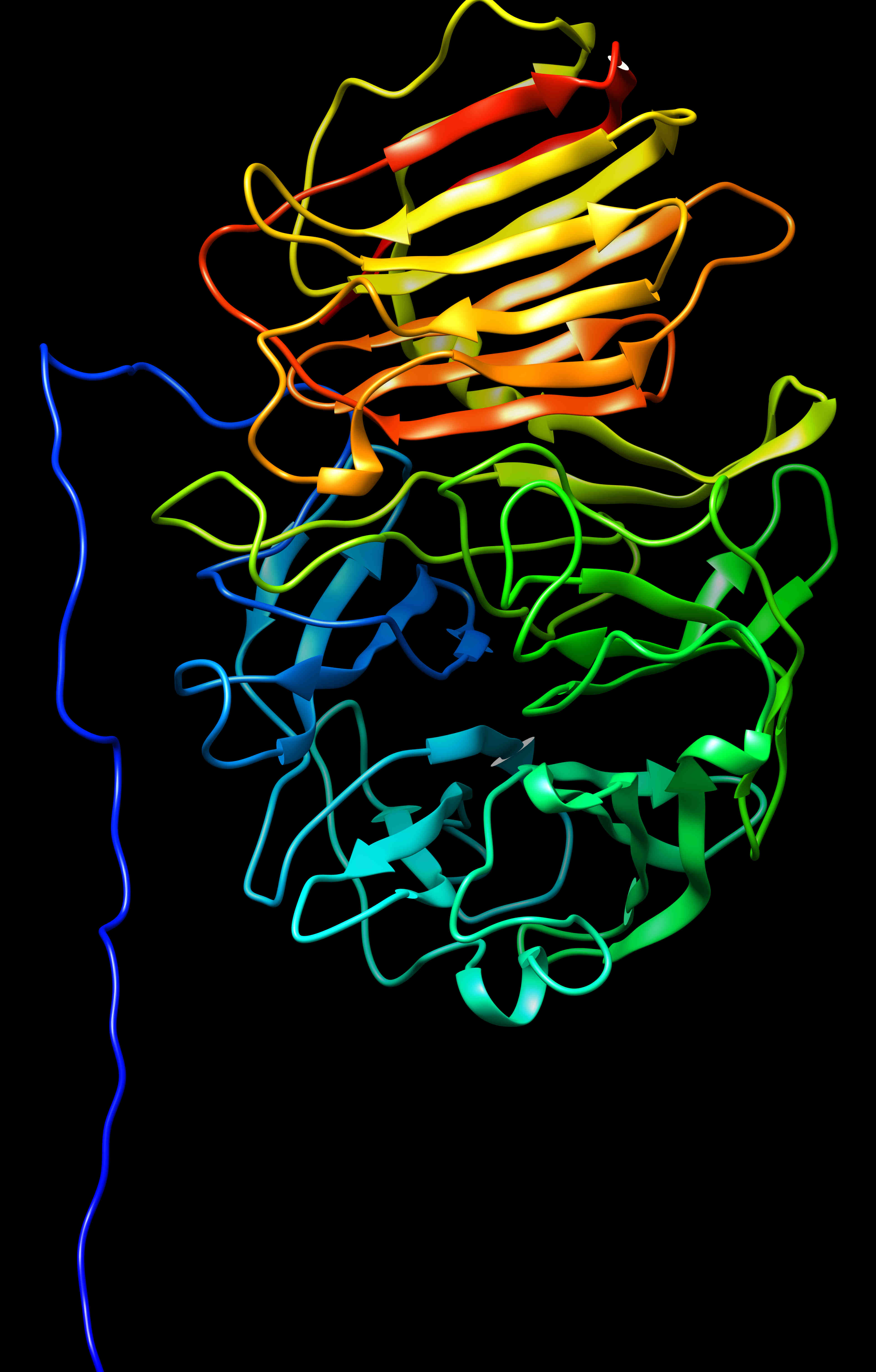

Supplement: S2 Dataset — (ZIP) [file pone.0200607.s002.zip › Abinitio_Models/FOP6.jpg]

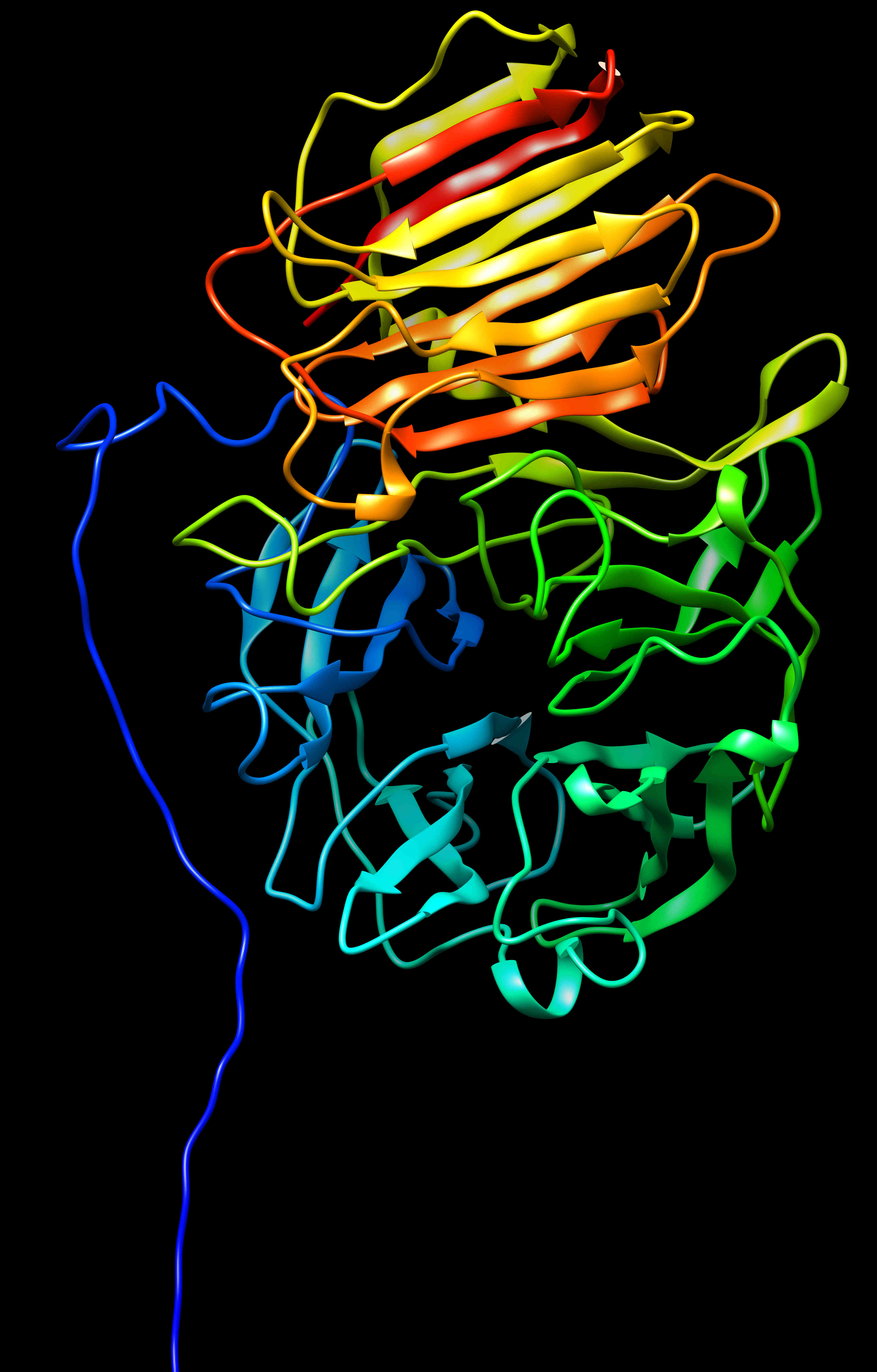

Supplement: S2 Dataset — (ZIP) [file pone.0200607.s002.zip › Abinitio_Models/FOP7.jpg]

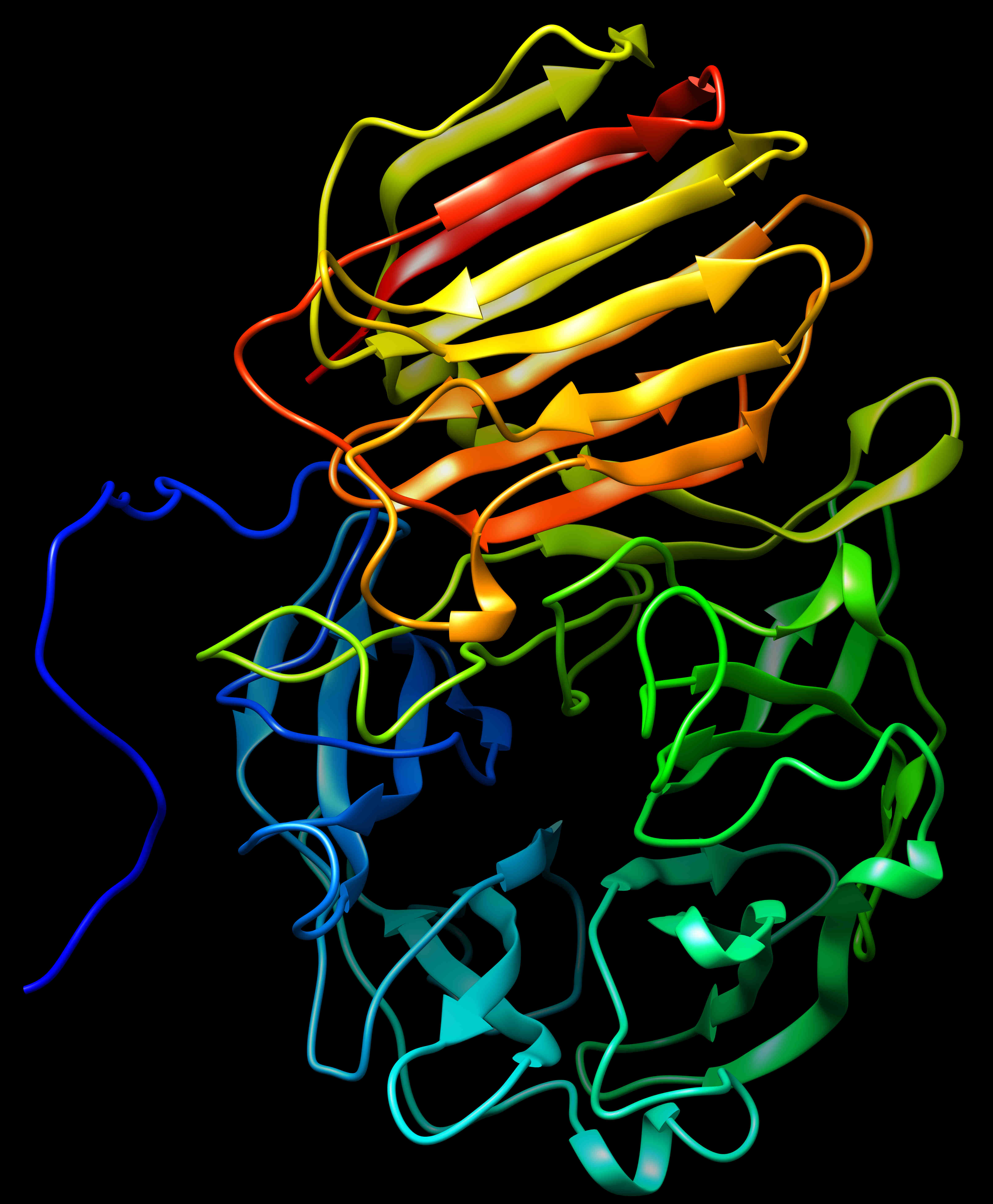

Supplement: S2 Dataset — (ZIP) [file pone.0200607.s002.zip › Abinitio_Models/FOP8.jpg]

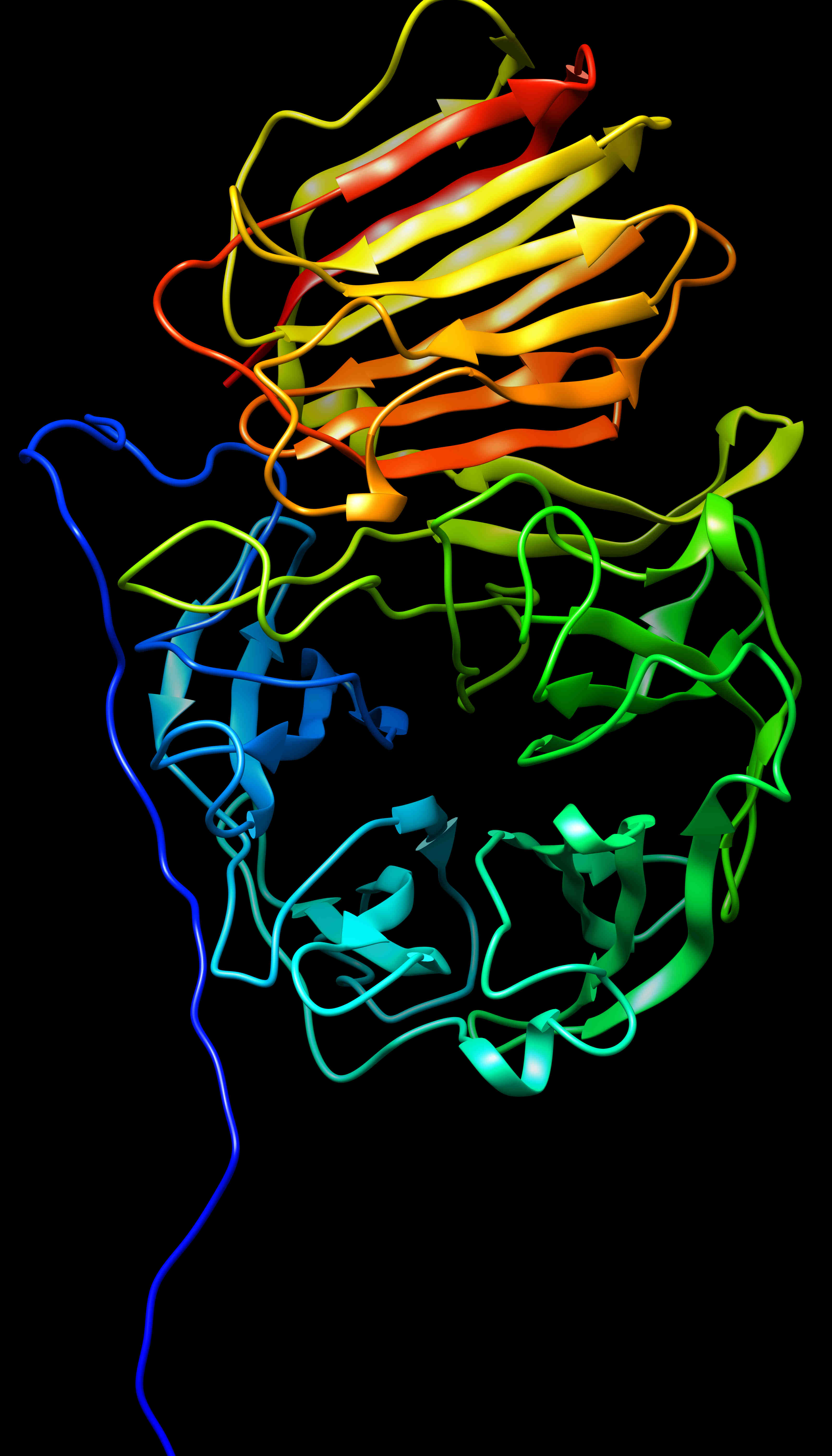

Supplement: S2 Dataset — (ZIP) [file pone.0200607.s002.zip › Abinitio_Models/FOP9.jpg]

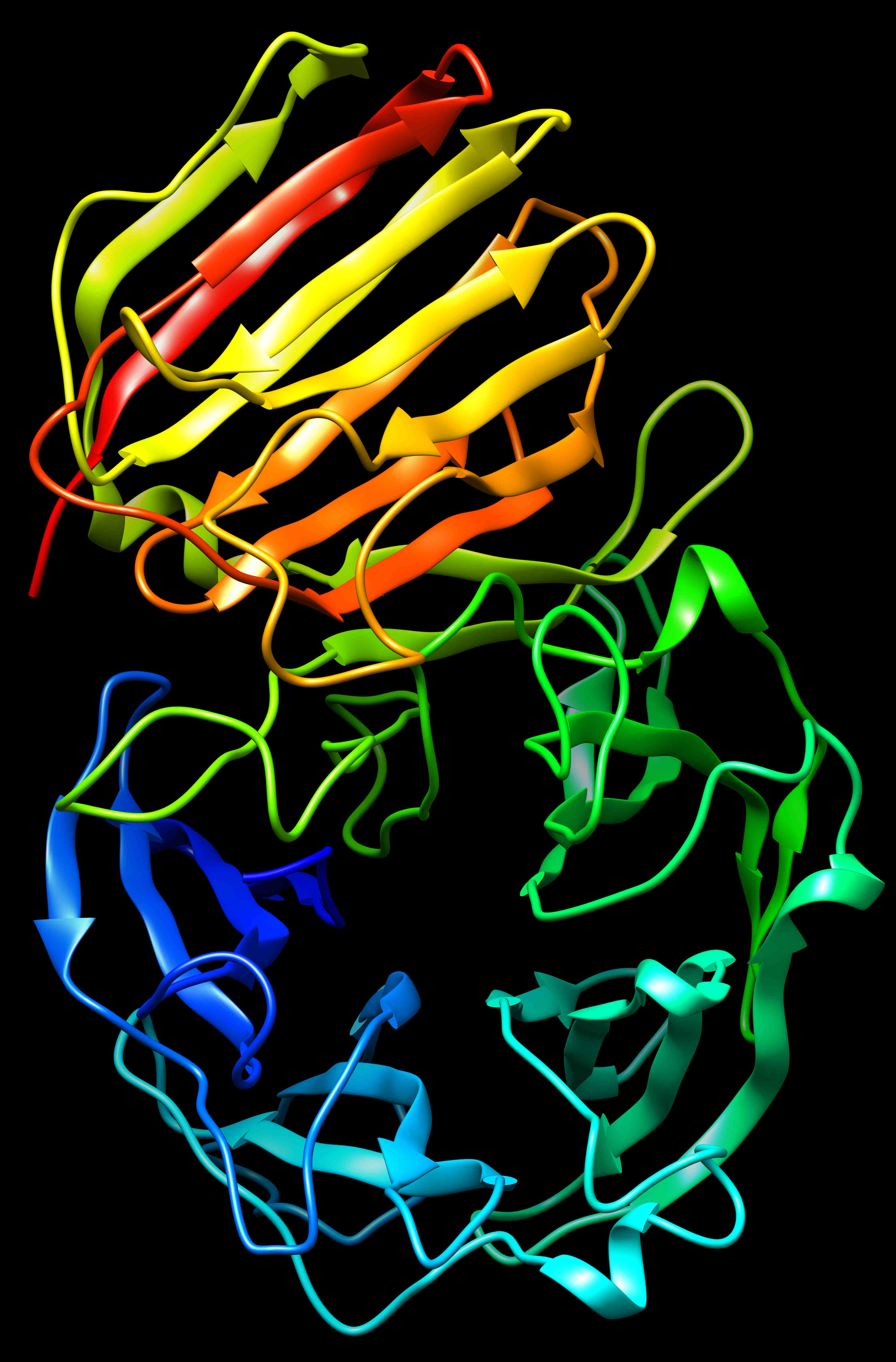

Supplement: S2 Dataset — (ZIP) [file pone.0200607.s002.zip › Abinitio_Models/MPP1.jpg]

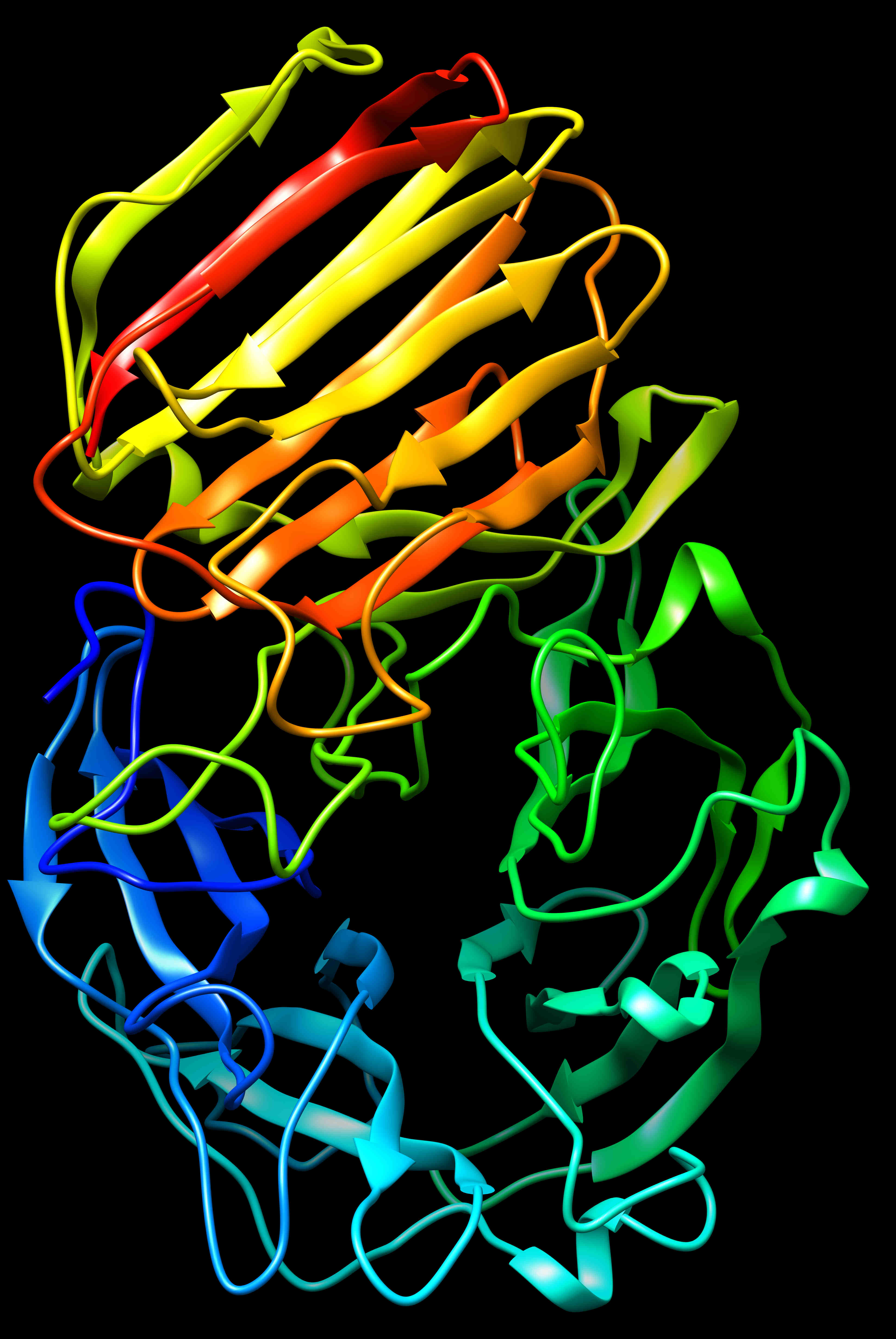

Supplement: S2 Dataset — (ZIP) [file pone.0200607.s002.zip › Abinitio_Models/OMP1.jpg]

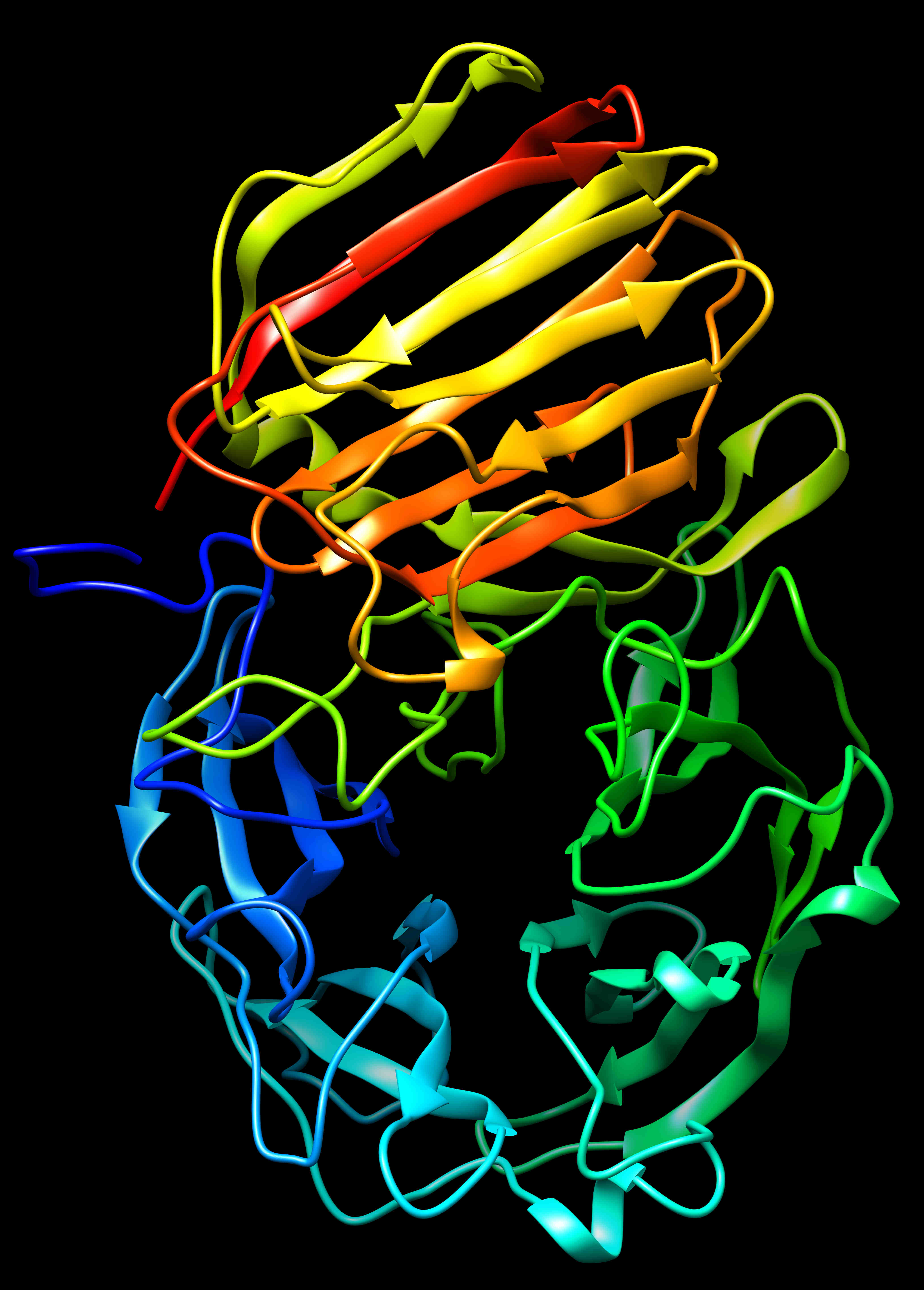

Supplement: S2 Dataset — (ZIP) [file pone.0200607.s002.zip › Abinitio_Models/PBP1.jpg]

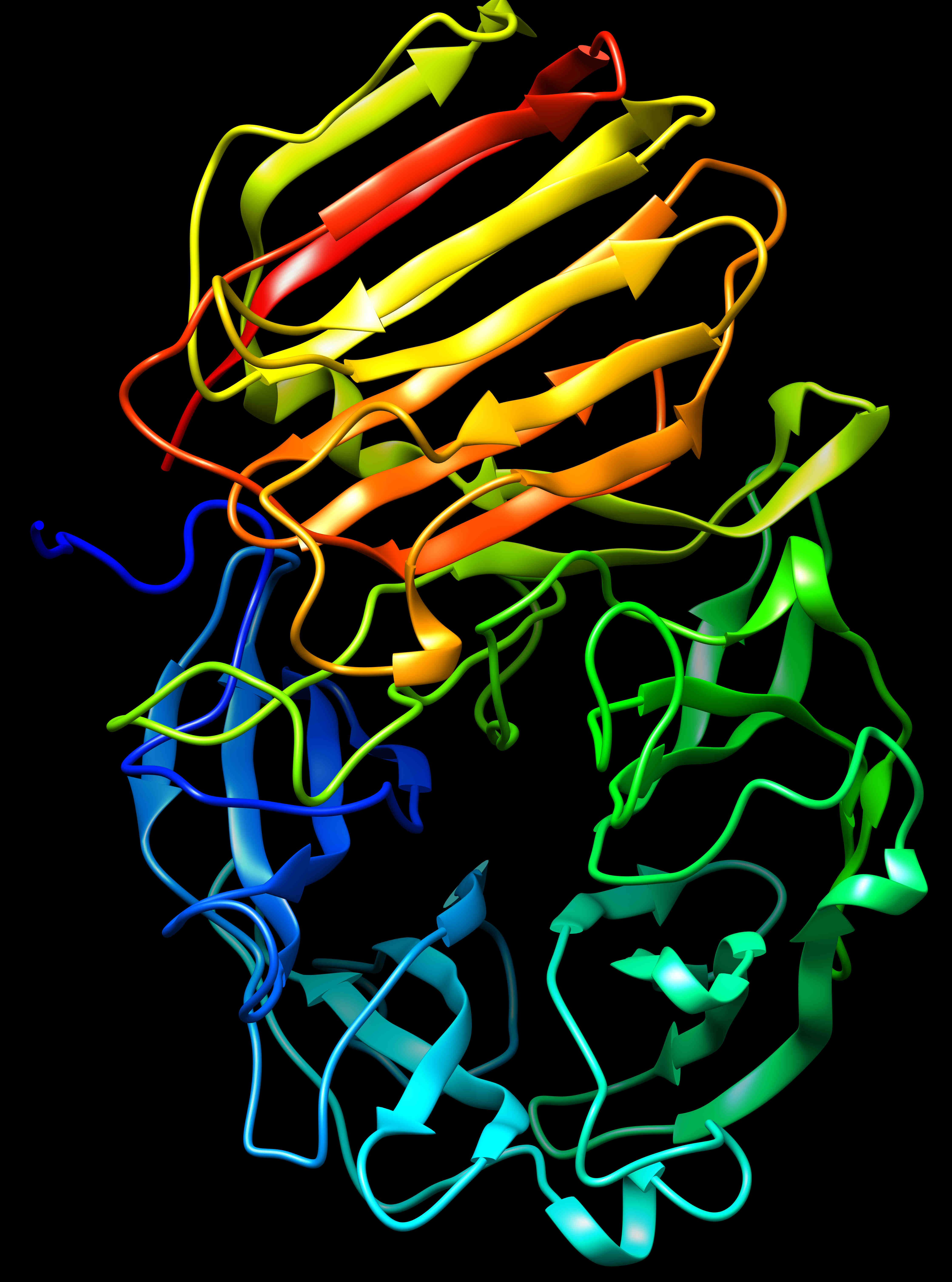

Supplement: S2 Dataset — (ZIP) [file pone.0200607.s002.zip › Abinitio_Models/PBP2.jpg]

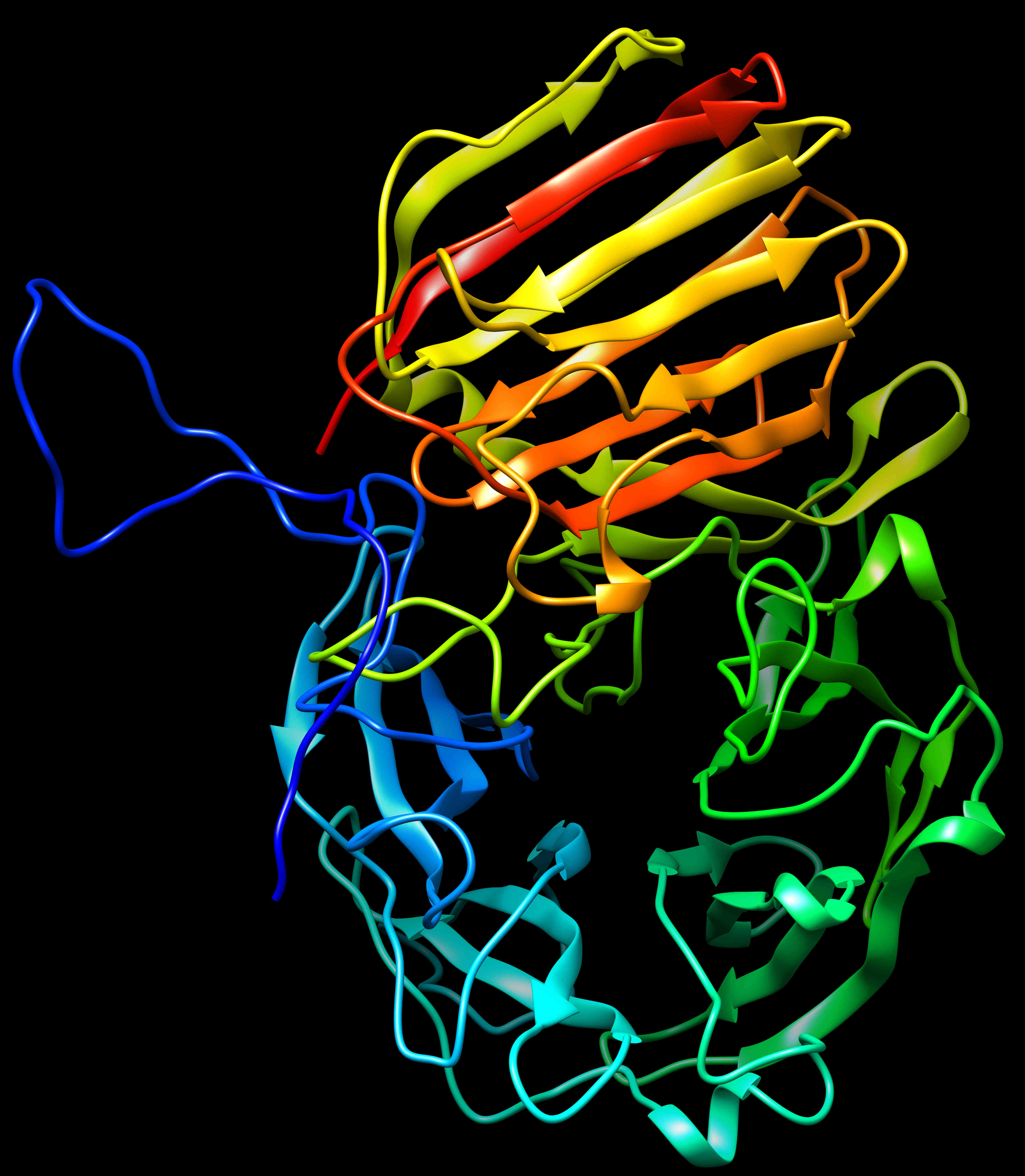

Supplement: S2 Dataset — (ZIP) [file pone.0200607.s002.zip › Abinitio_Models/PCP1.jpg]

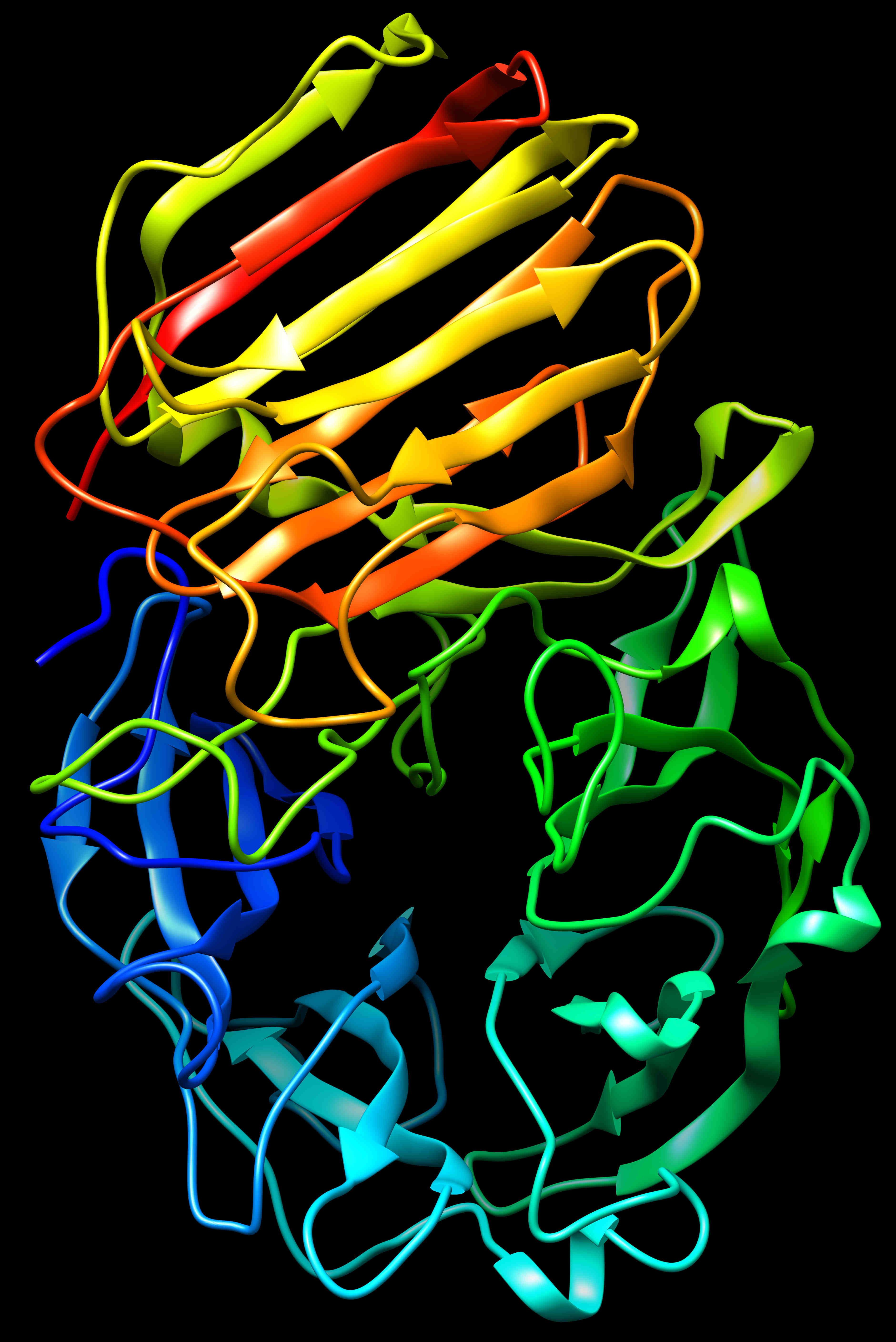

Supplement: S2 Dataset — (ZIP) [file pone.0200607.s002.zip › Abinitio_Models/PGP1.jpg]

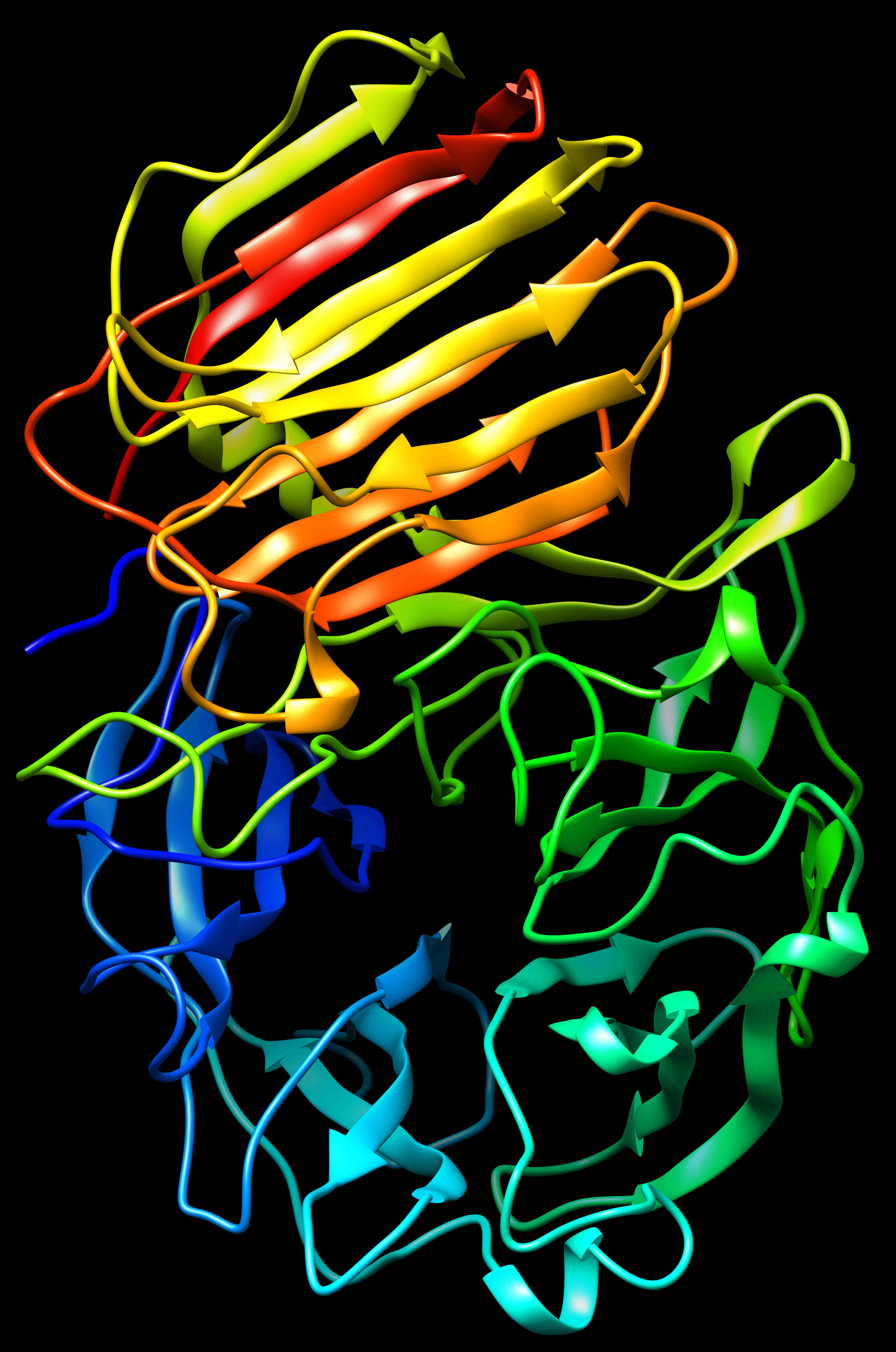

Supplement: S2 Dataset — (ZIP) [file pone.0200607.s002.zip › Abinitio_Models/PGP3.jpg]

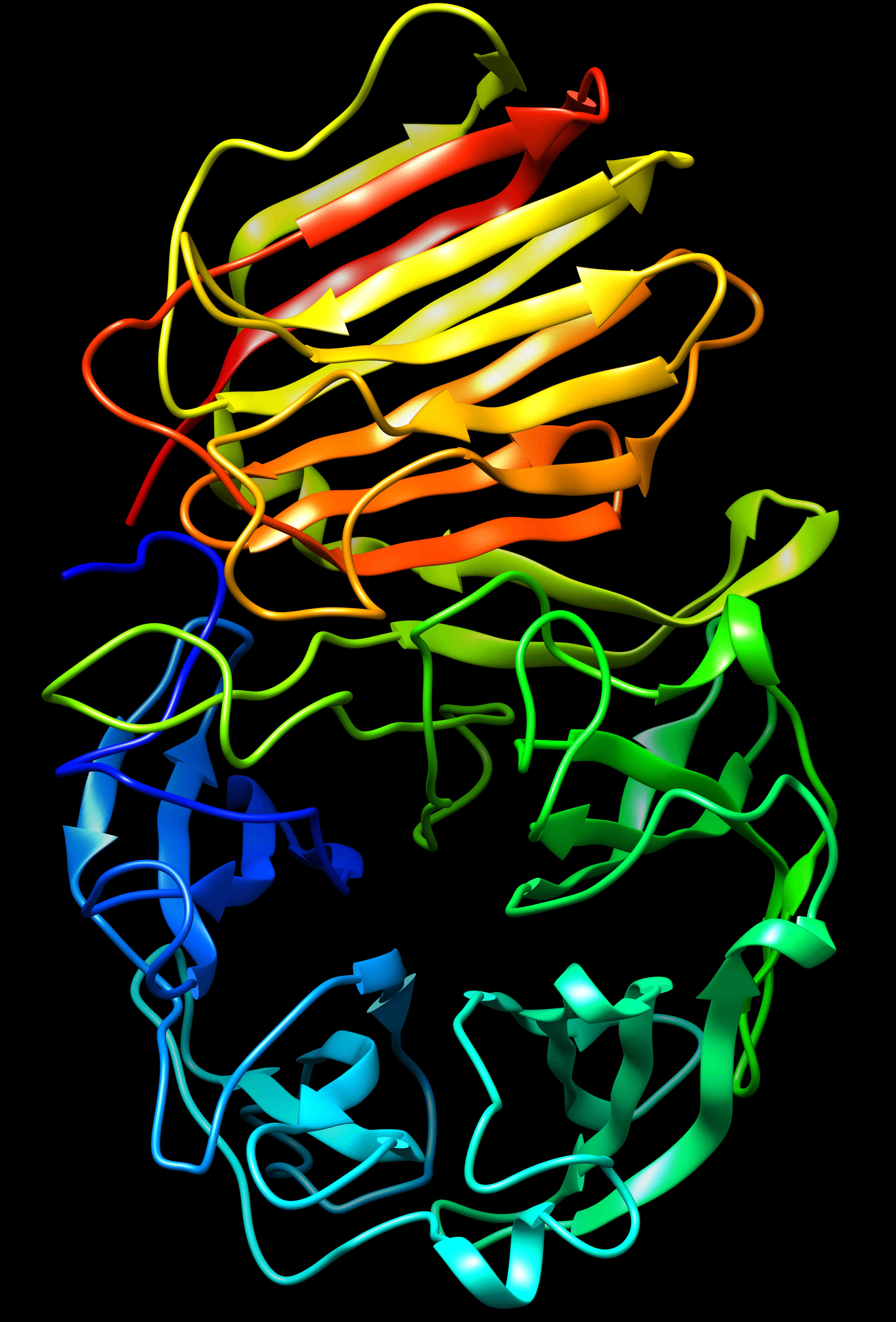

Supplement: S2 Dataset — (ZIP) [file pone.0200607.s002.zip › Abinitio_Models/PGP4.jpg]

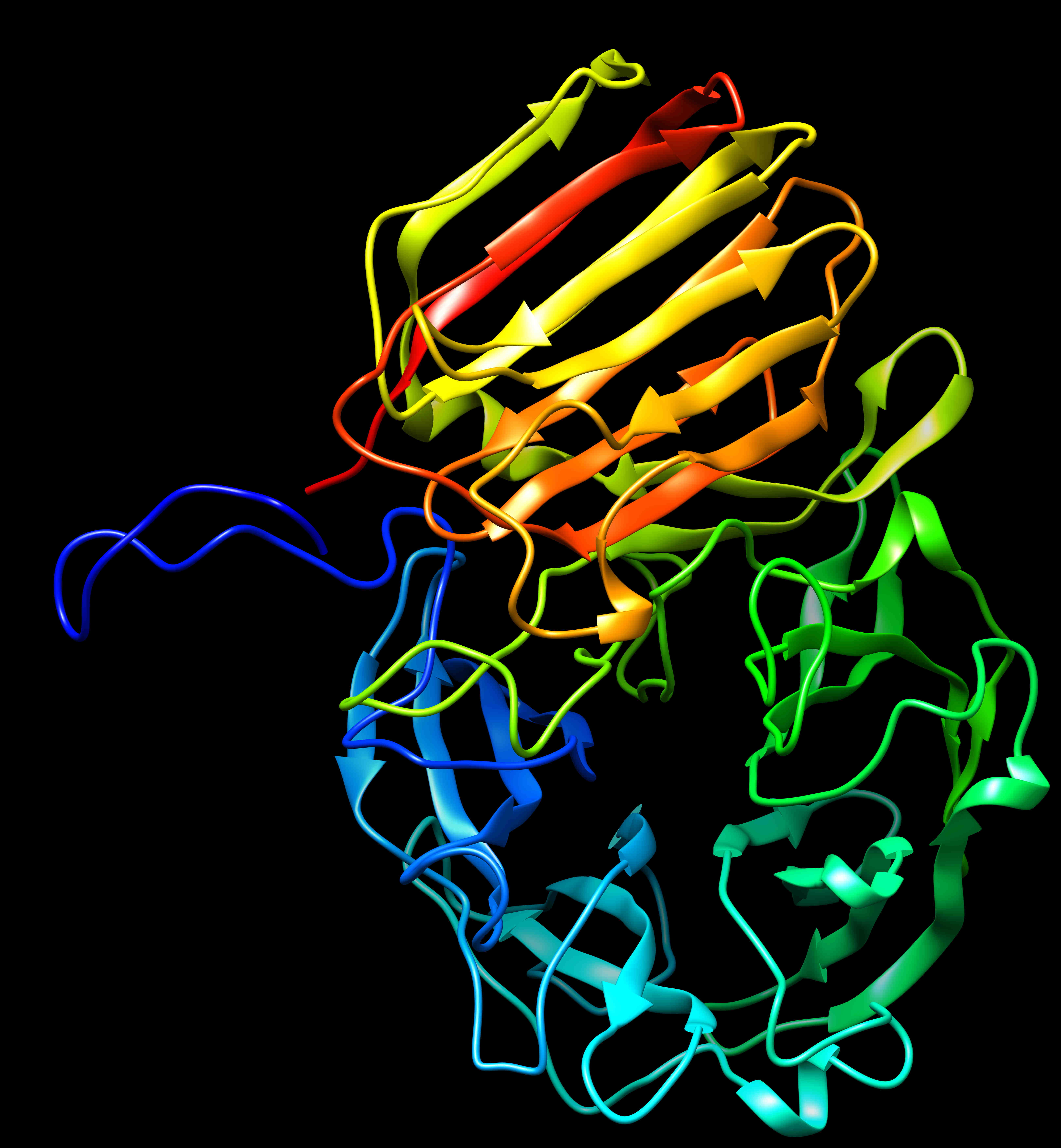

Supplement: S2 Dataset — (ZIP) [file pone.0200607.s002.zip › Abinitio_Models/PNP1.jpg]
