# Supplementary figures and images for: In Silico evaluation and identification of fungi capable of producing endo-inulinase enzyme (part 2 of 4)
Source: PLoS One. 2018 Jul 12;13(7):e0200607. doi: 10.1371/journal.pone.0200607 (PMC6042768; doi:10.1371/journal.pone.0200607)

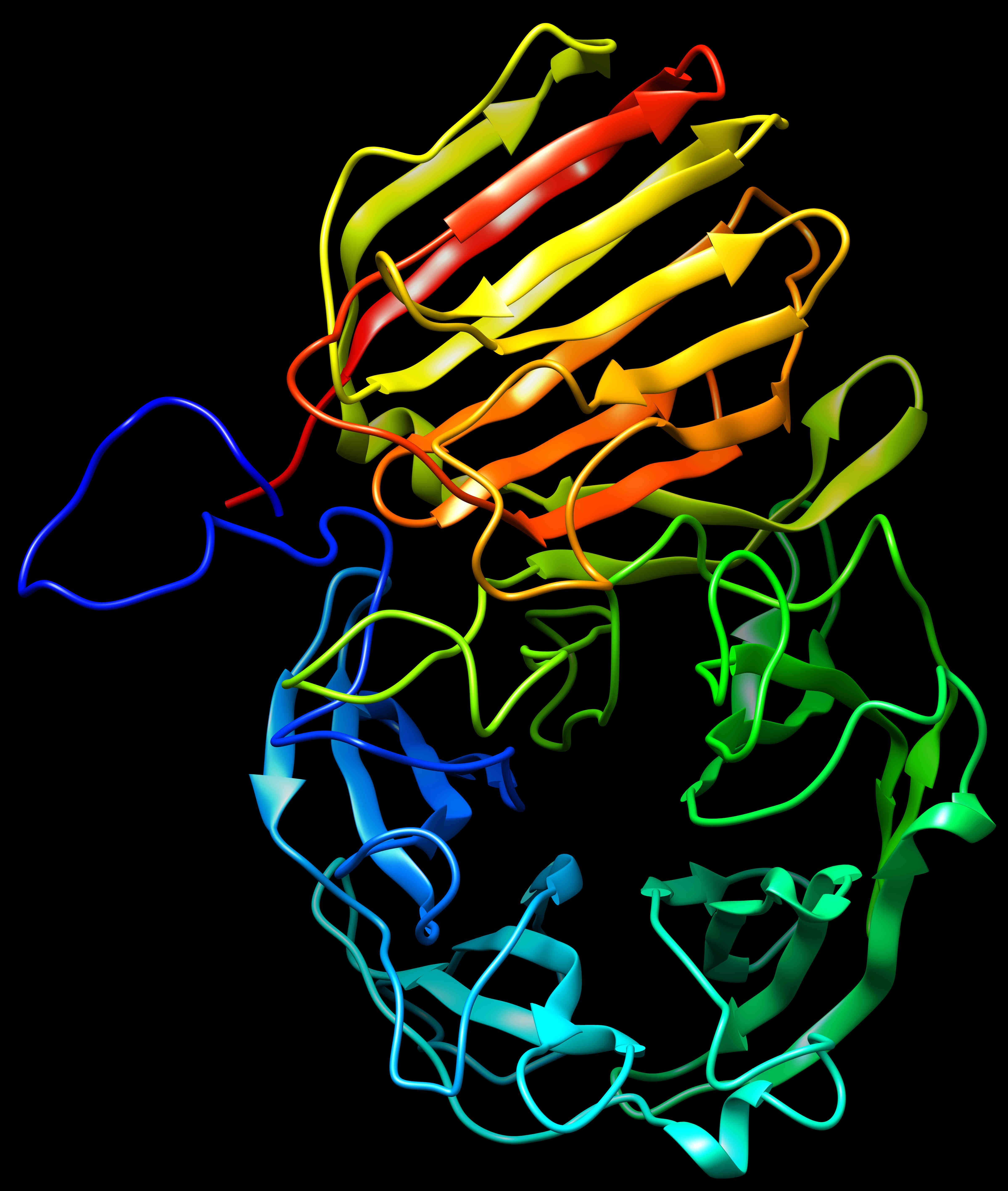

Supplement: S2 Dataset — (ZIP) [file pone.0200607.s002.zip › Abinitio_Models/PSR1.jpg]

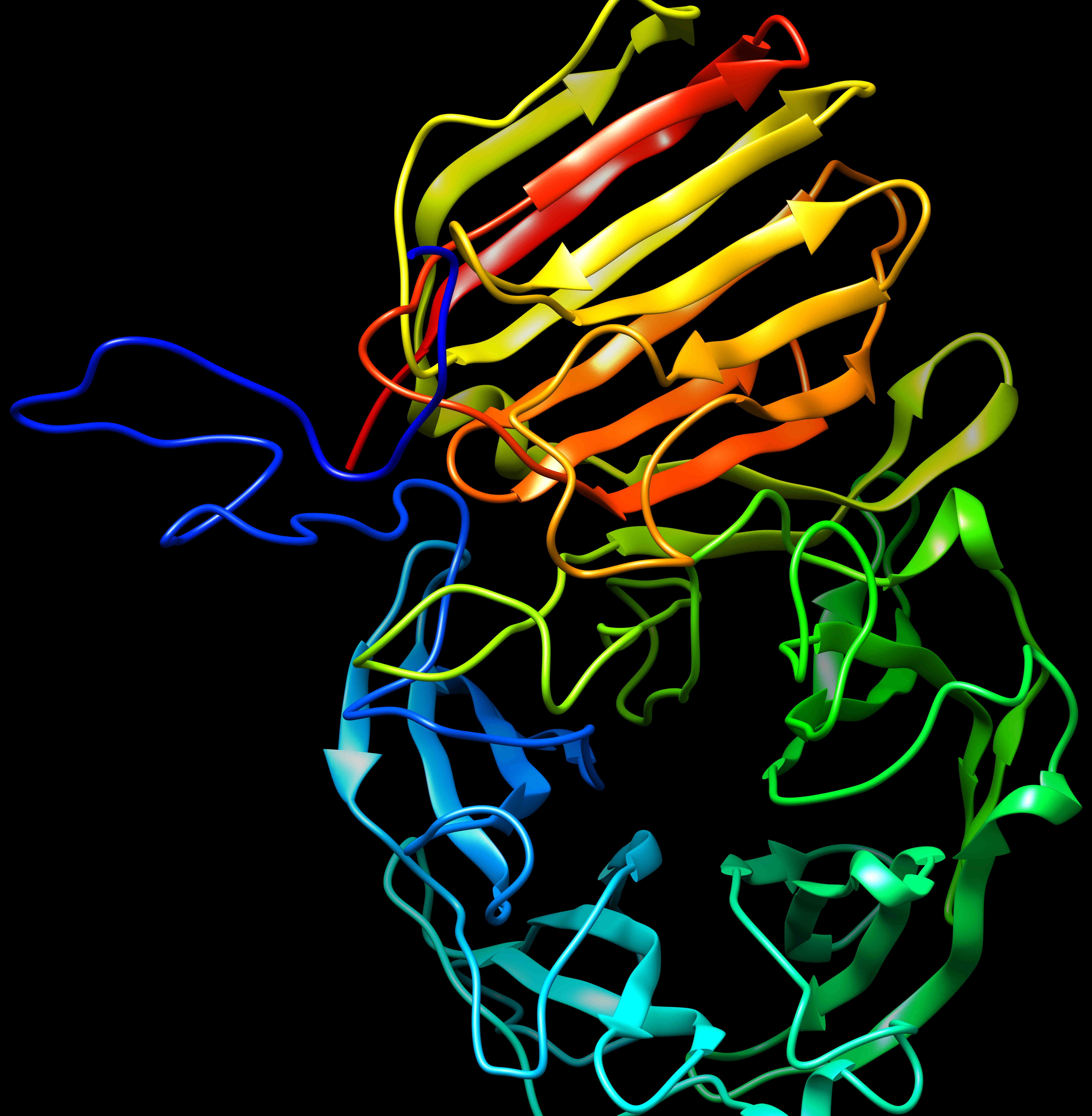

Supplement: S2 Dataset — (ZIP) [file pone.0200607.s002.zip › Abinitio_Models/PSR2.jpg]

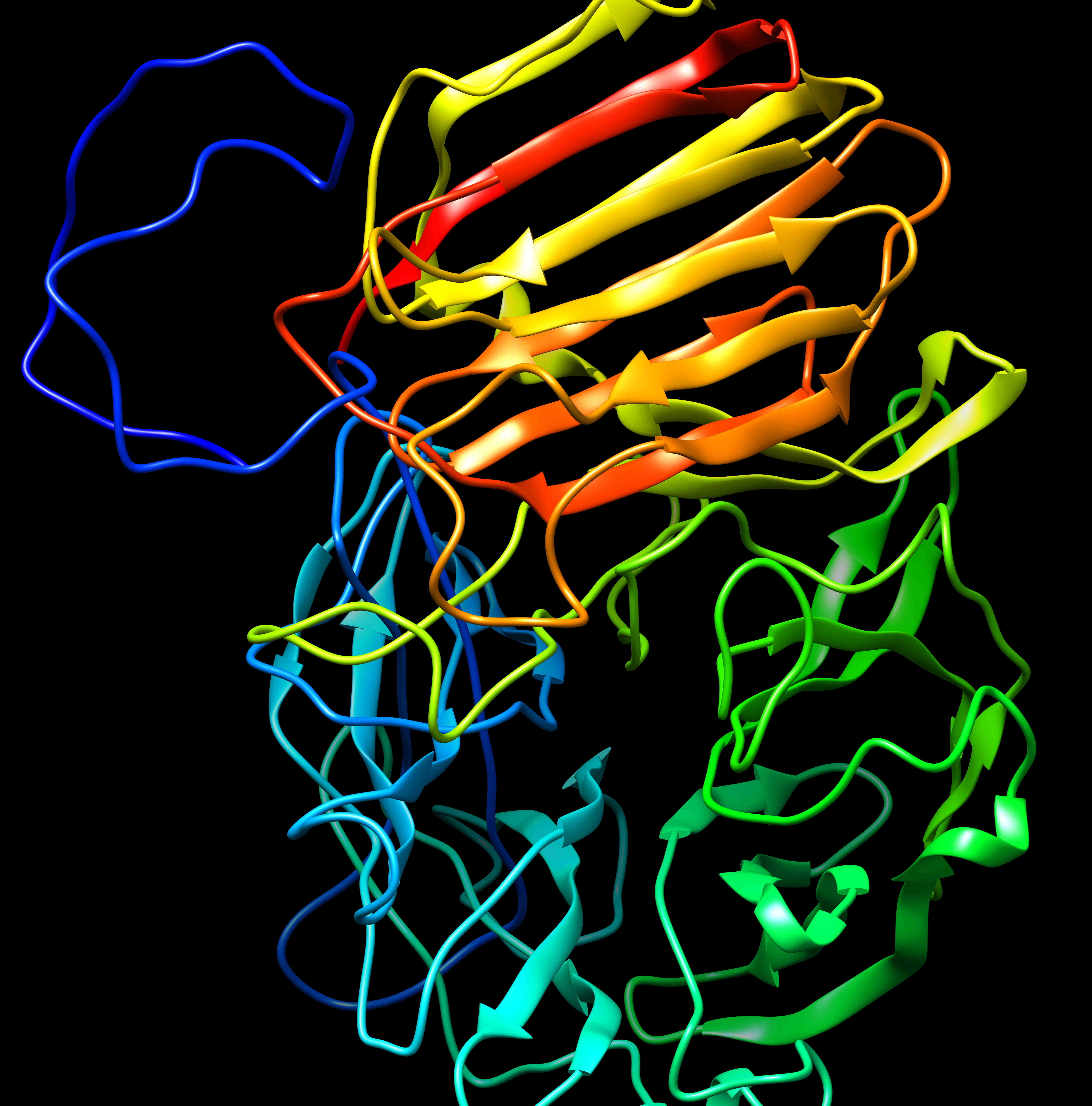

Supplement: S2 Dataset — (ZIP) [file pone.0200607.s002.zip › Abinitio_Models/PSR3.jpg]

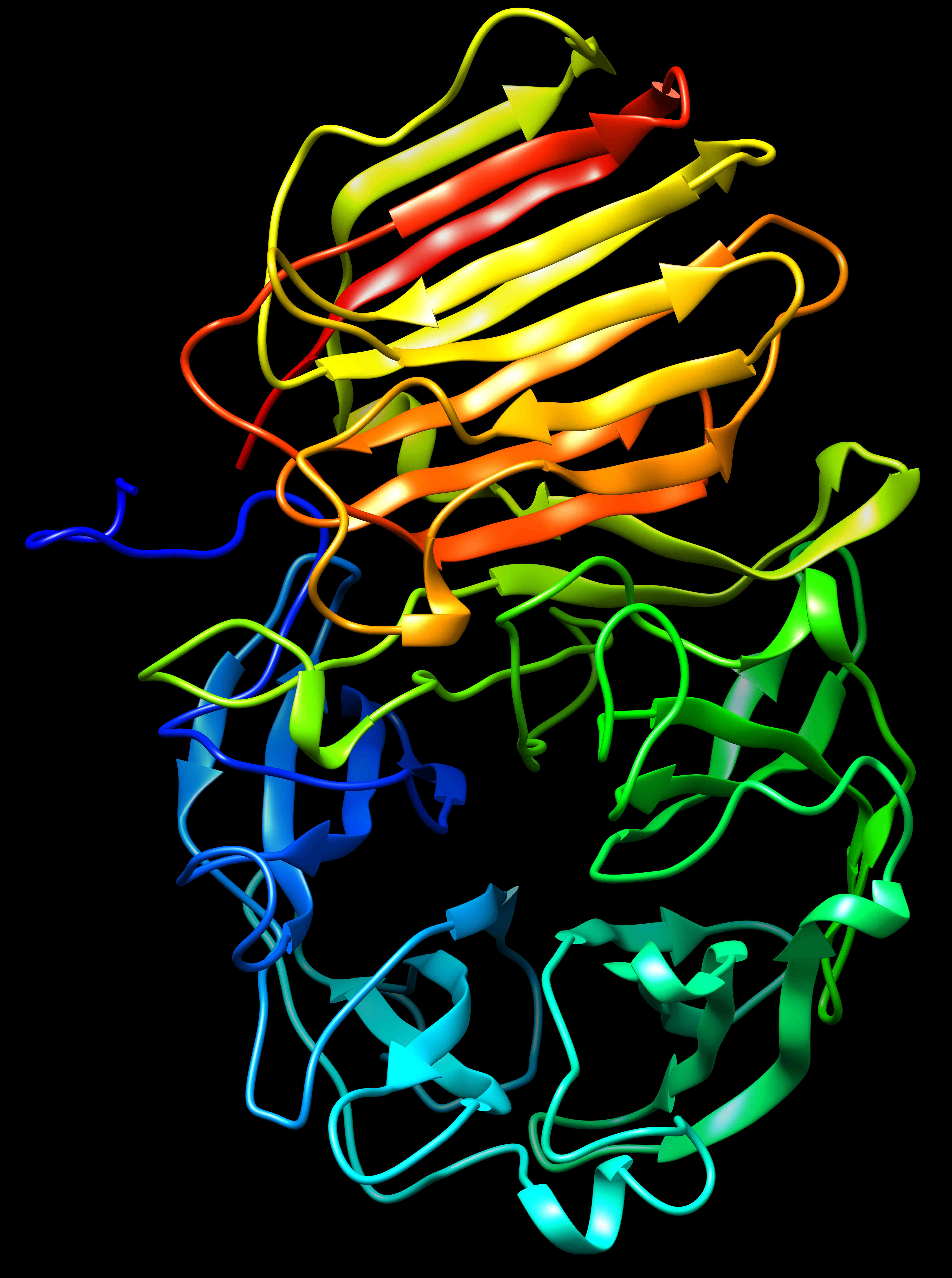

Supplement: S2 Dataset — (ZIP) [file pone.0200607.s002.zip › Abinitio_Models/SCHP1.jpg]

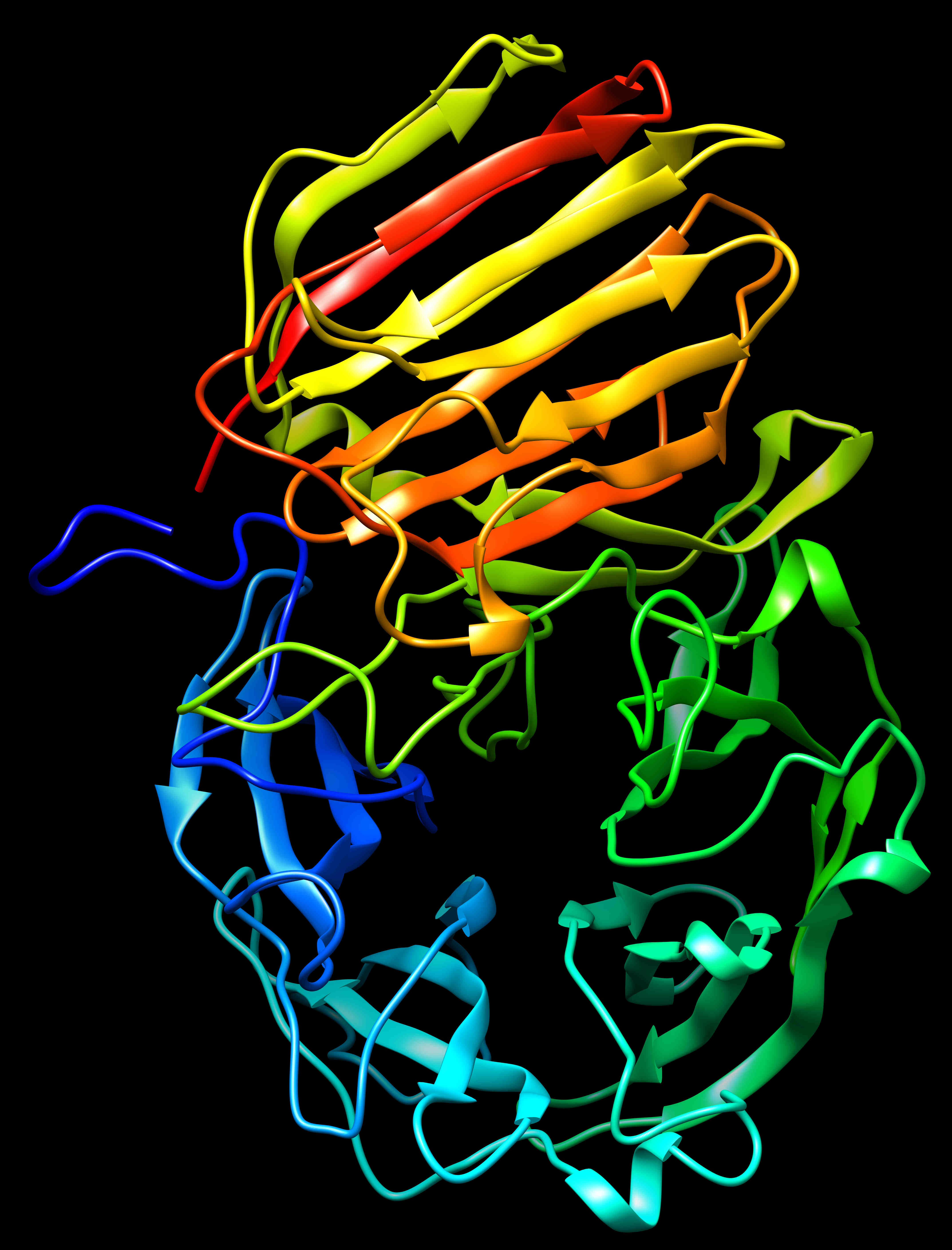

Supplement: S2 Dataset — (ZIP) [file pone.0200607.s002.zip › Abinitio_Models/SCHP2.jpg]

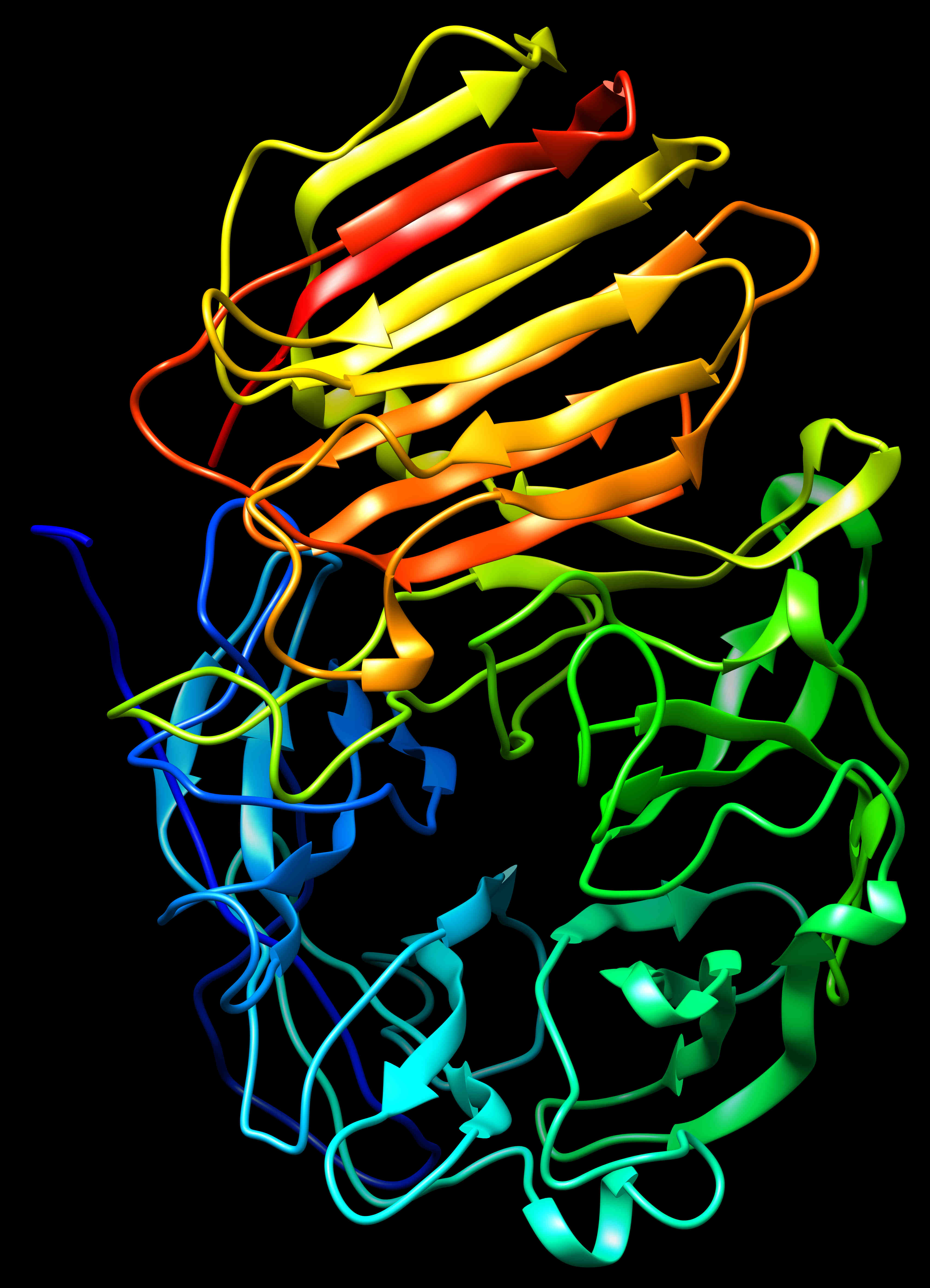

Supplement: S2 Dataset — (ZIP) [file pone.0200607.s002.zip › Abinitio_Models/SCP1.jpg]

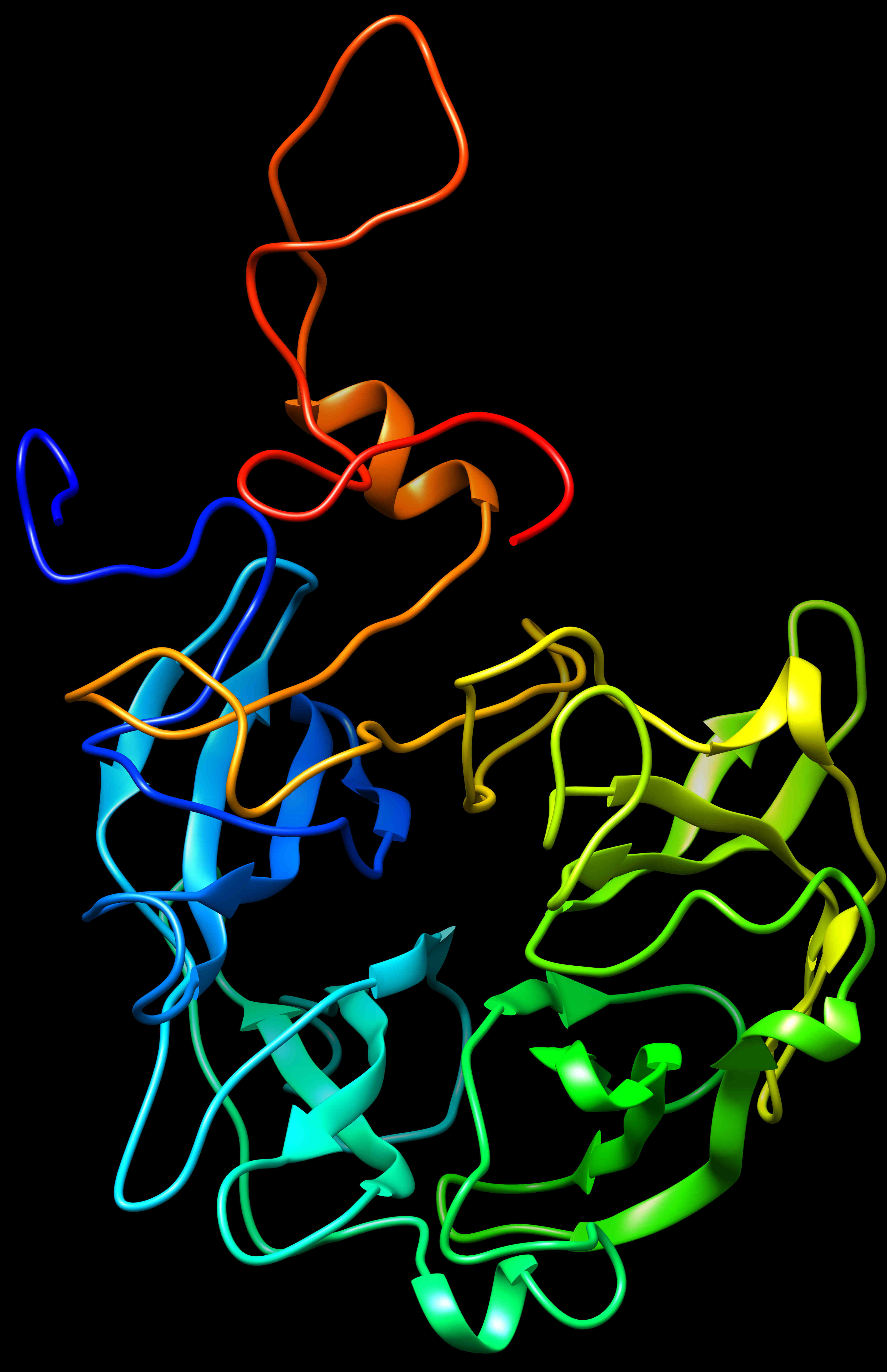

Supplement: S2 Dataset — (ZIP) [file pone.0200607.s002.zip › Abinitio_Models/SCP18.jpg]

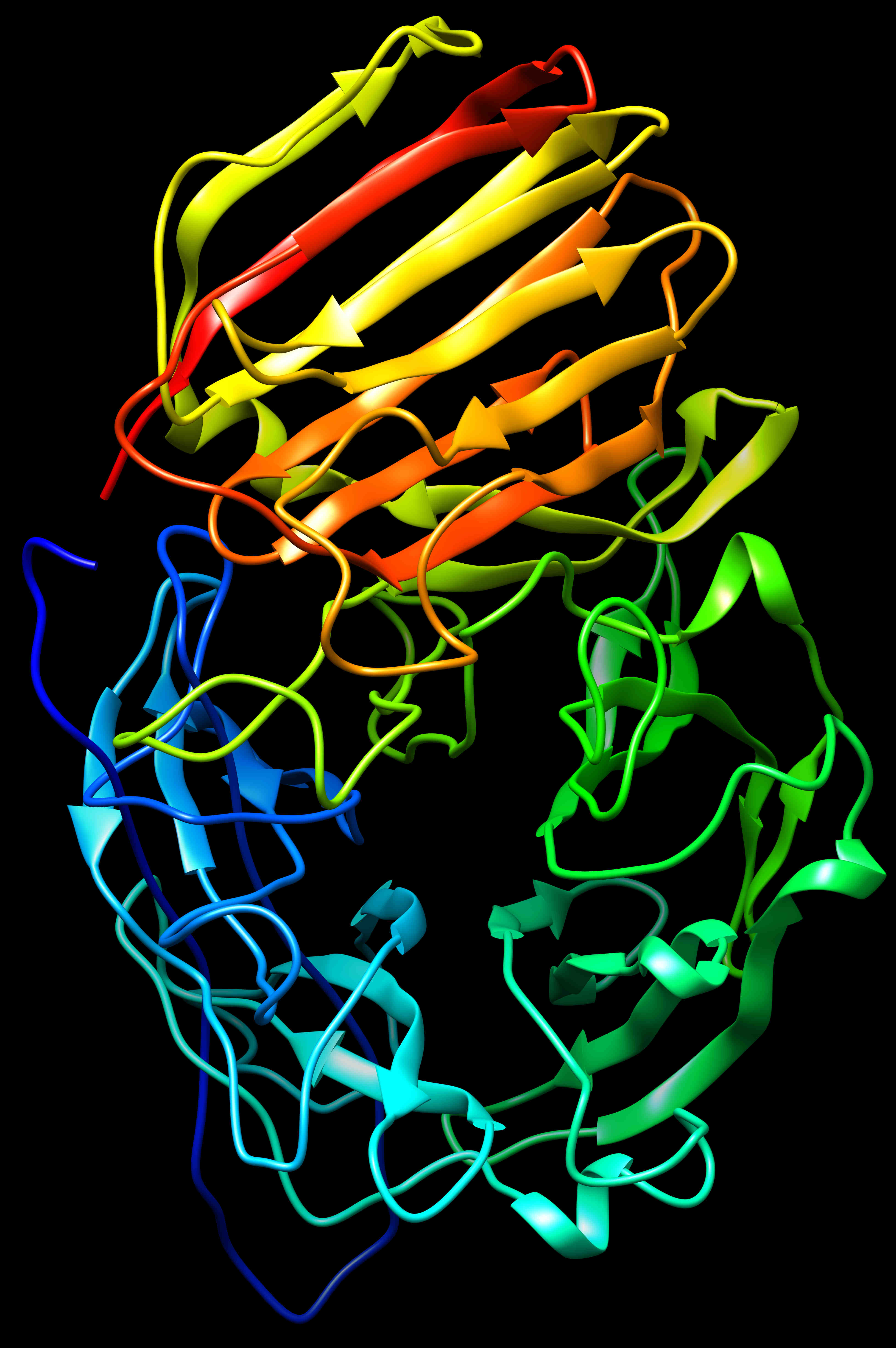

Supplement: S2 Dataset — (ZIP) [file pone.0200607.s002.zip › Abinitio_Models/SCP2.jpg]

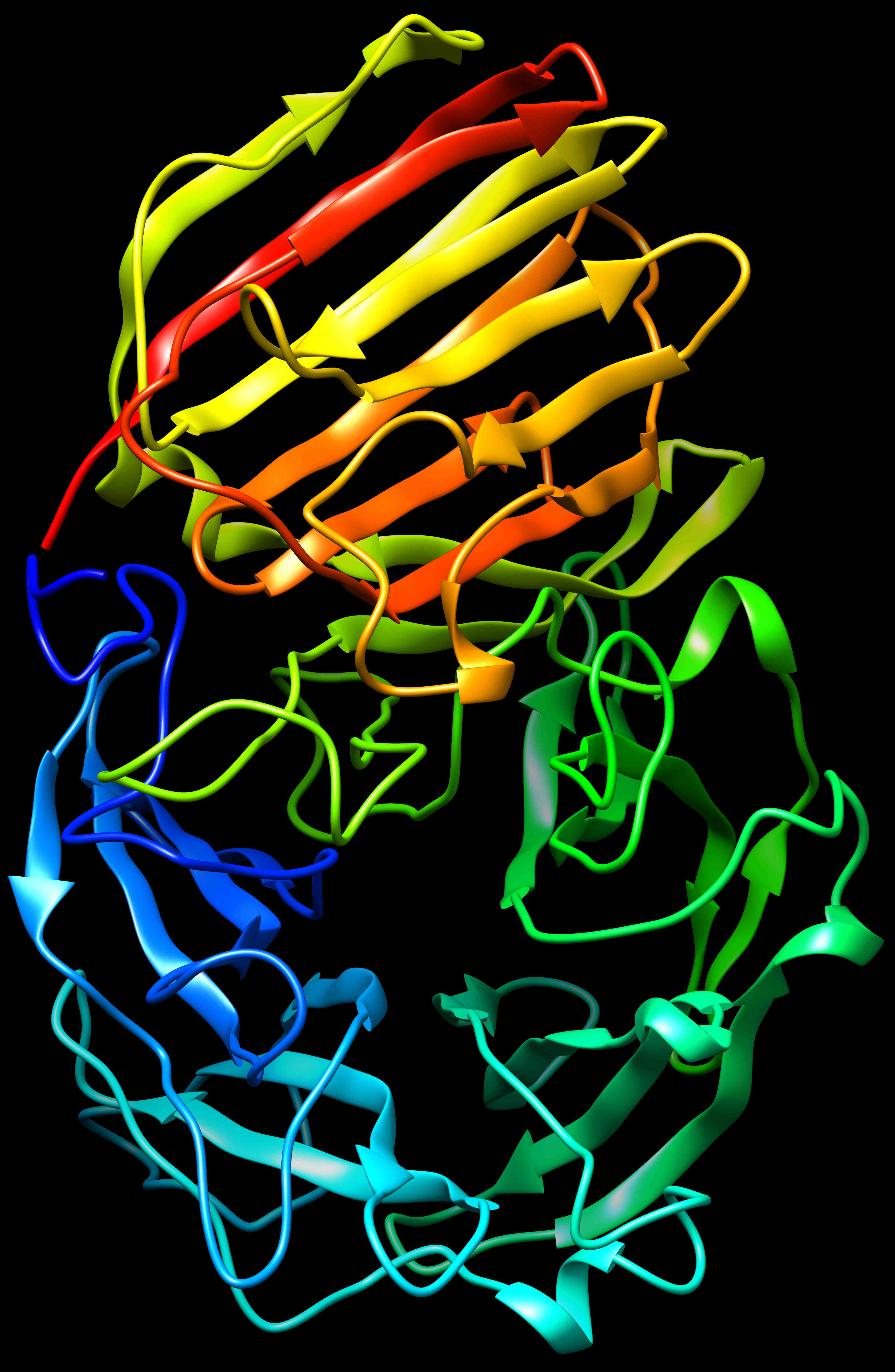

Supplement: S2 Dataset — (ZIP) [file pone.0200607.s002.zip › Abinitio_Models/SCP3.jpg]

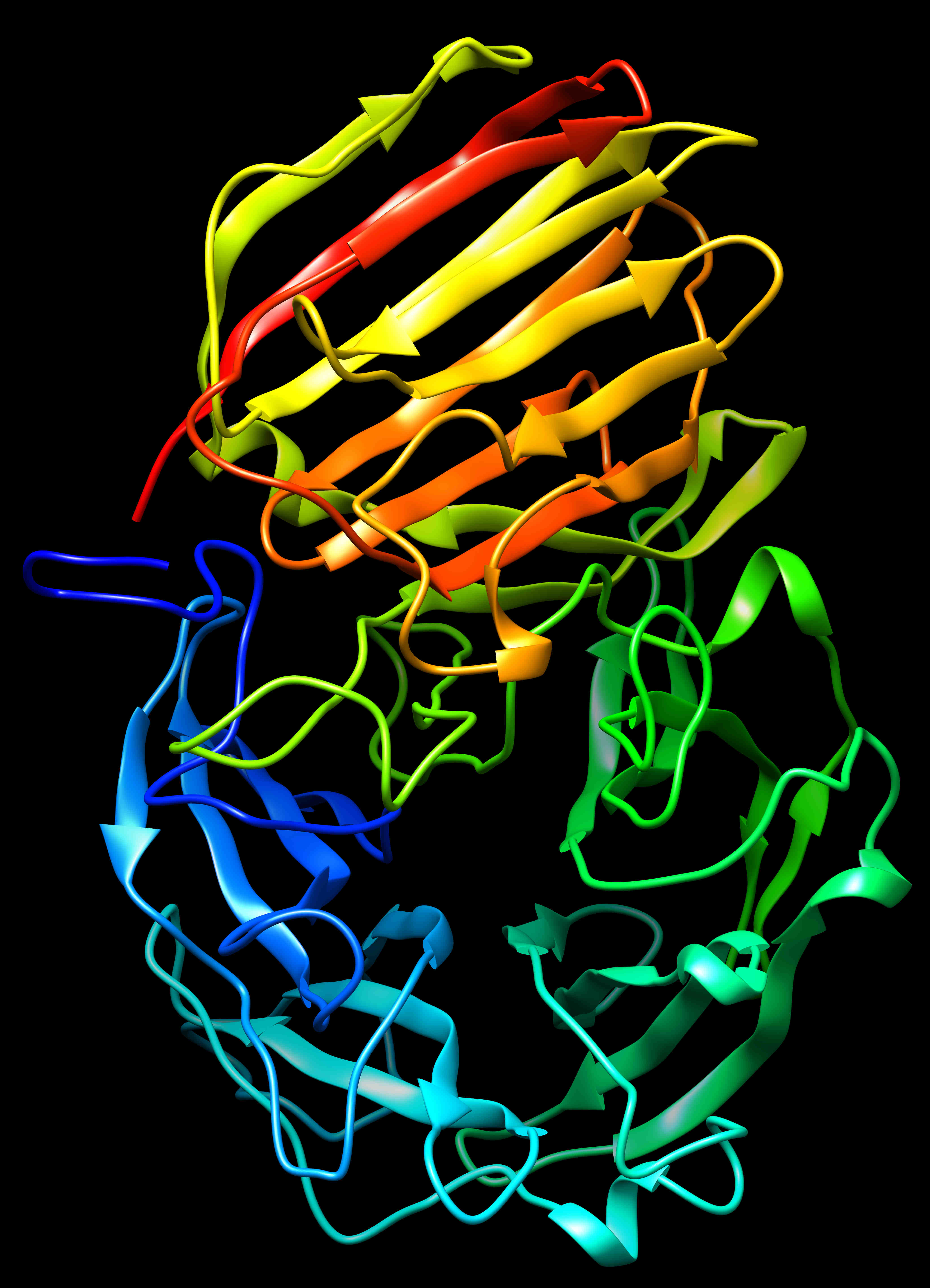

Supplement: S2 Dataset — (ZIP) [file pone.0200607.s002.zip › Abinitio_Models/SCP4.jpg]

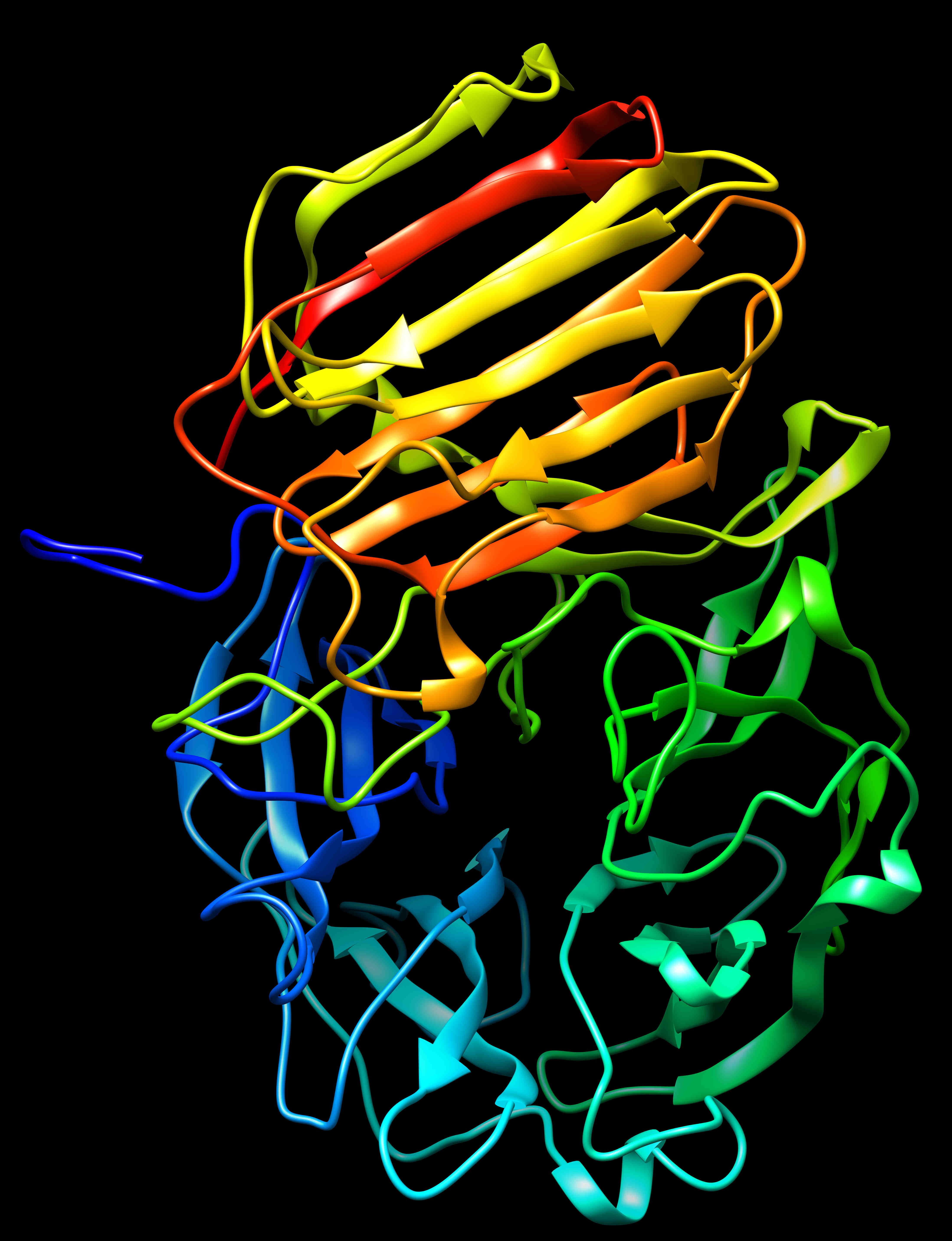

Supplement: S2 Dataset — (ZIP) [file pone.0200607.s002.zip › Abinitio_Models/SCP5.jpg]

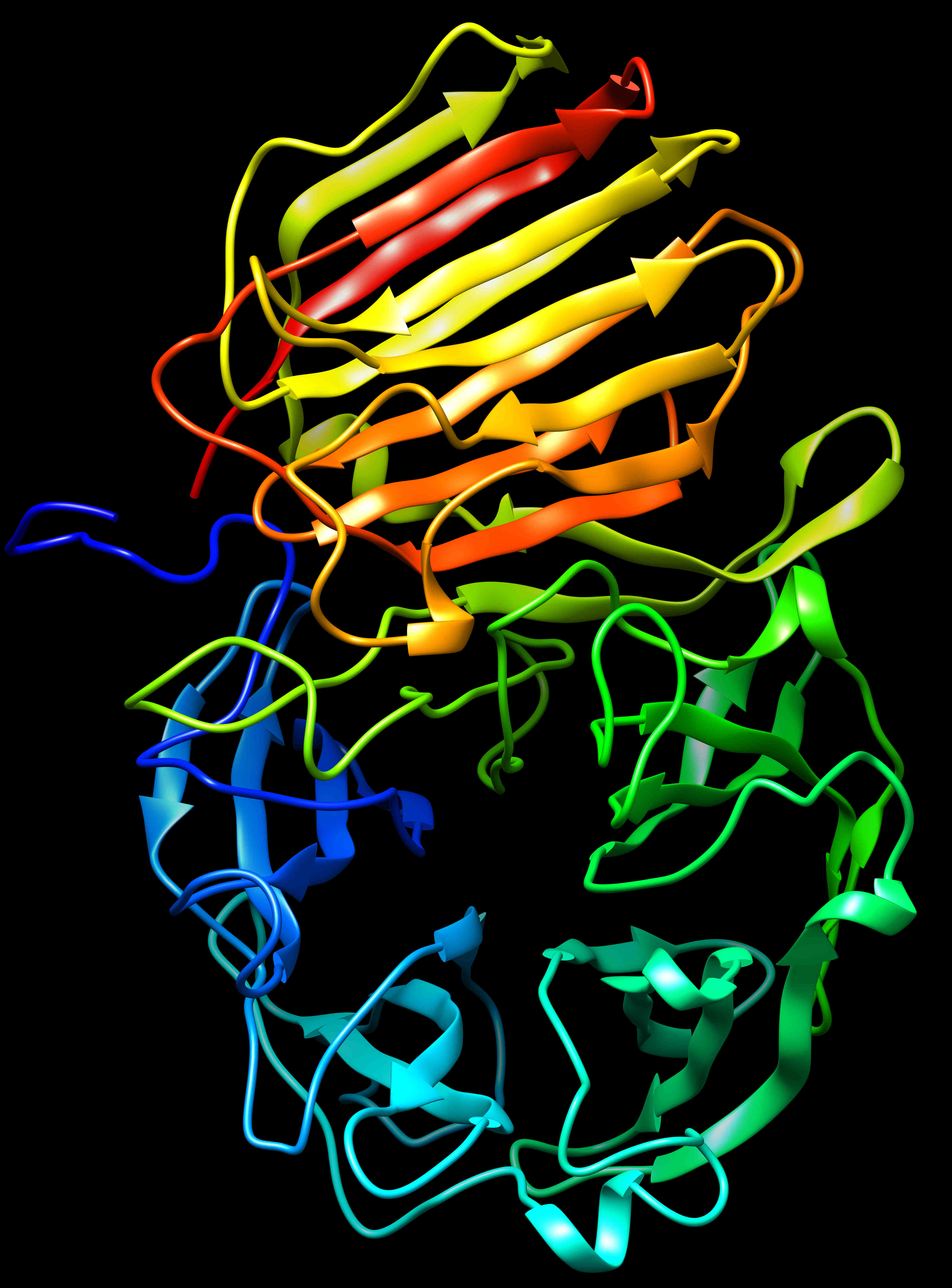

Supplement: S2 Dataset — (ZIP) [file pone.0200607.s002.zip › Abinitio_Models/SCP6.jpg]

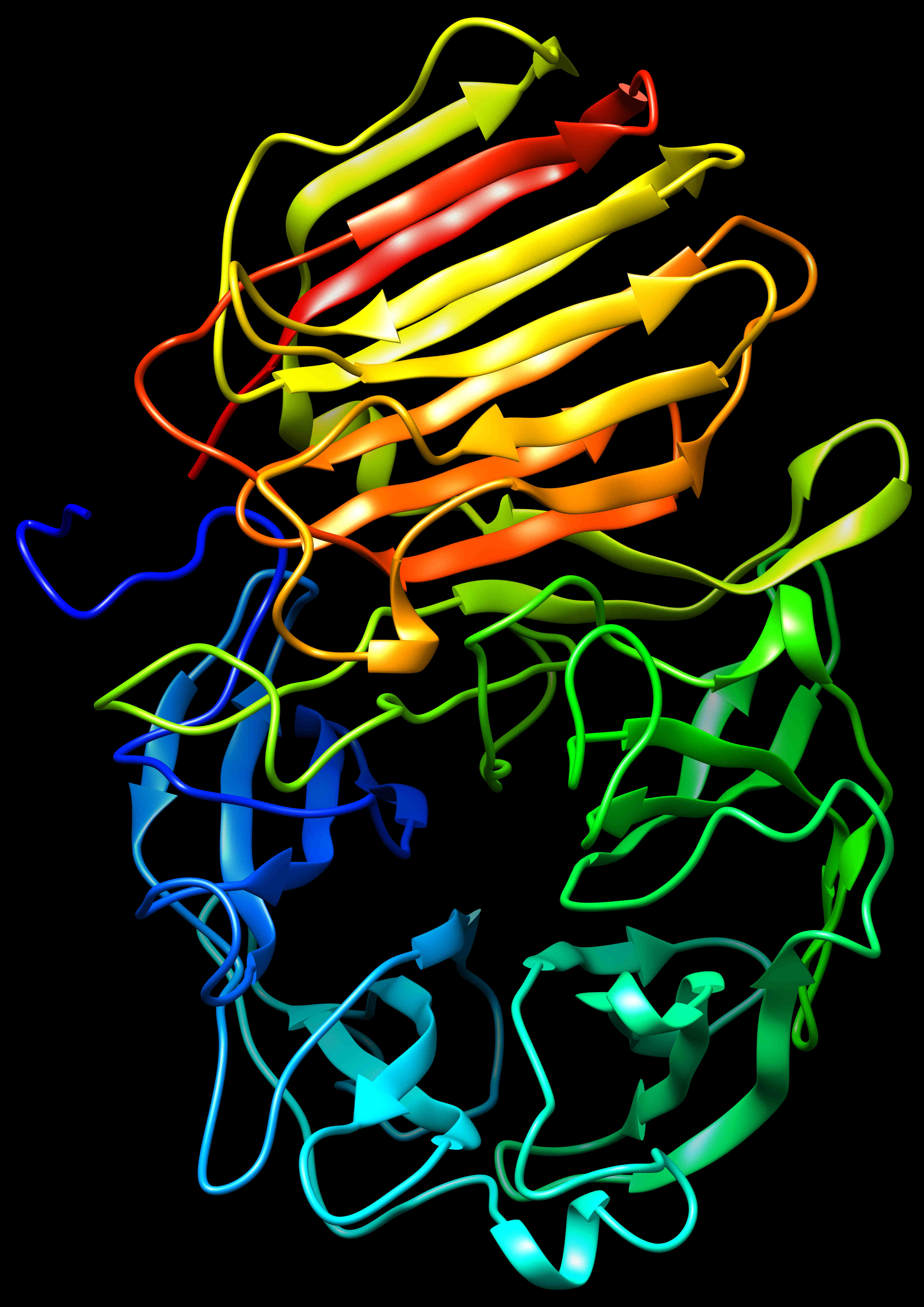

Supplement: S2 Dataset — (ZIP) [file pone.0200607.s002.zip › Abinitio_Models/SCP7.jpg]

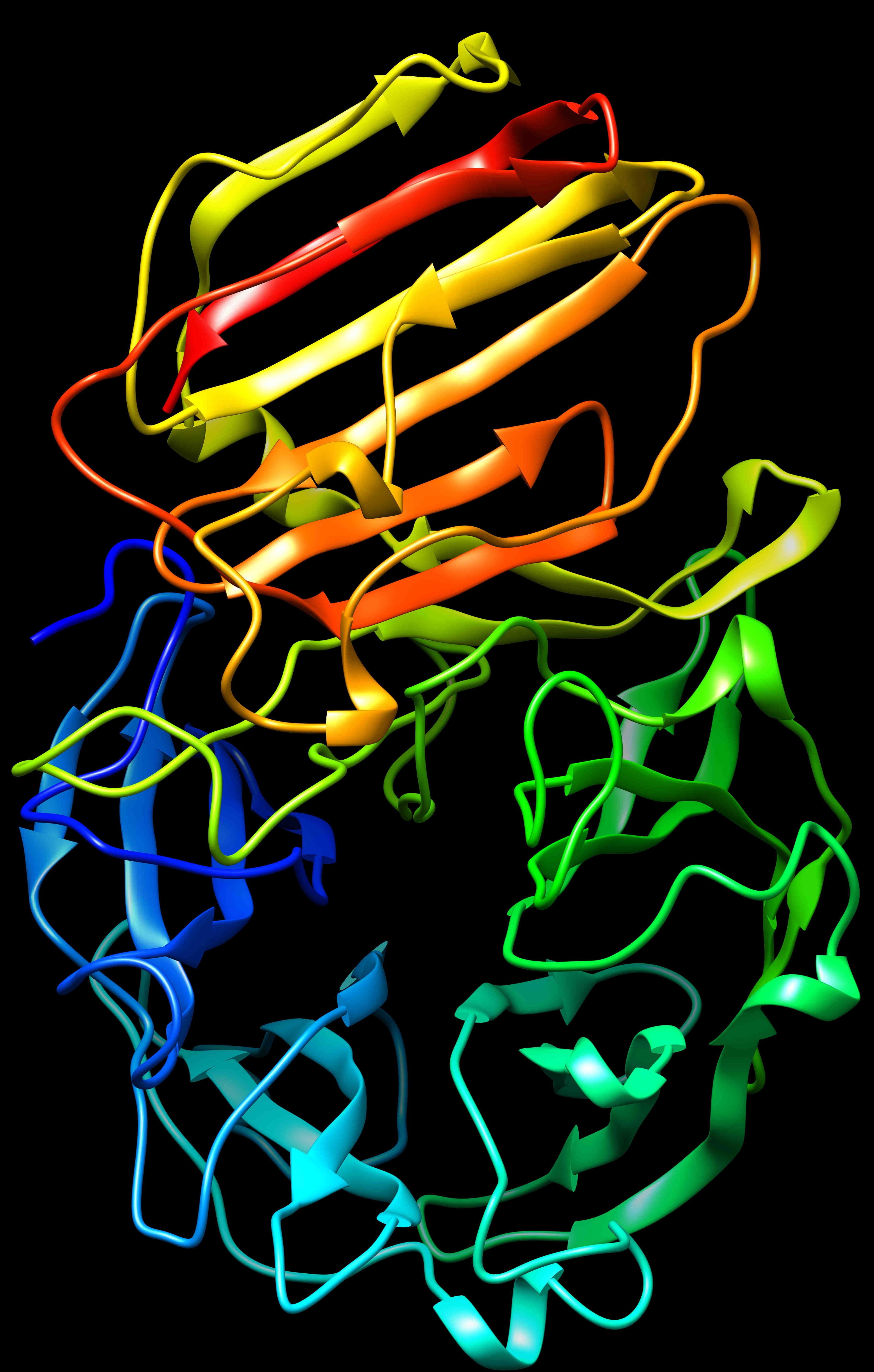

Supplement: S2 Dataset — (ZIP) [file pone.0200607.s002.zip › Abinitio_Models/TCP1.jpg]

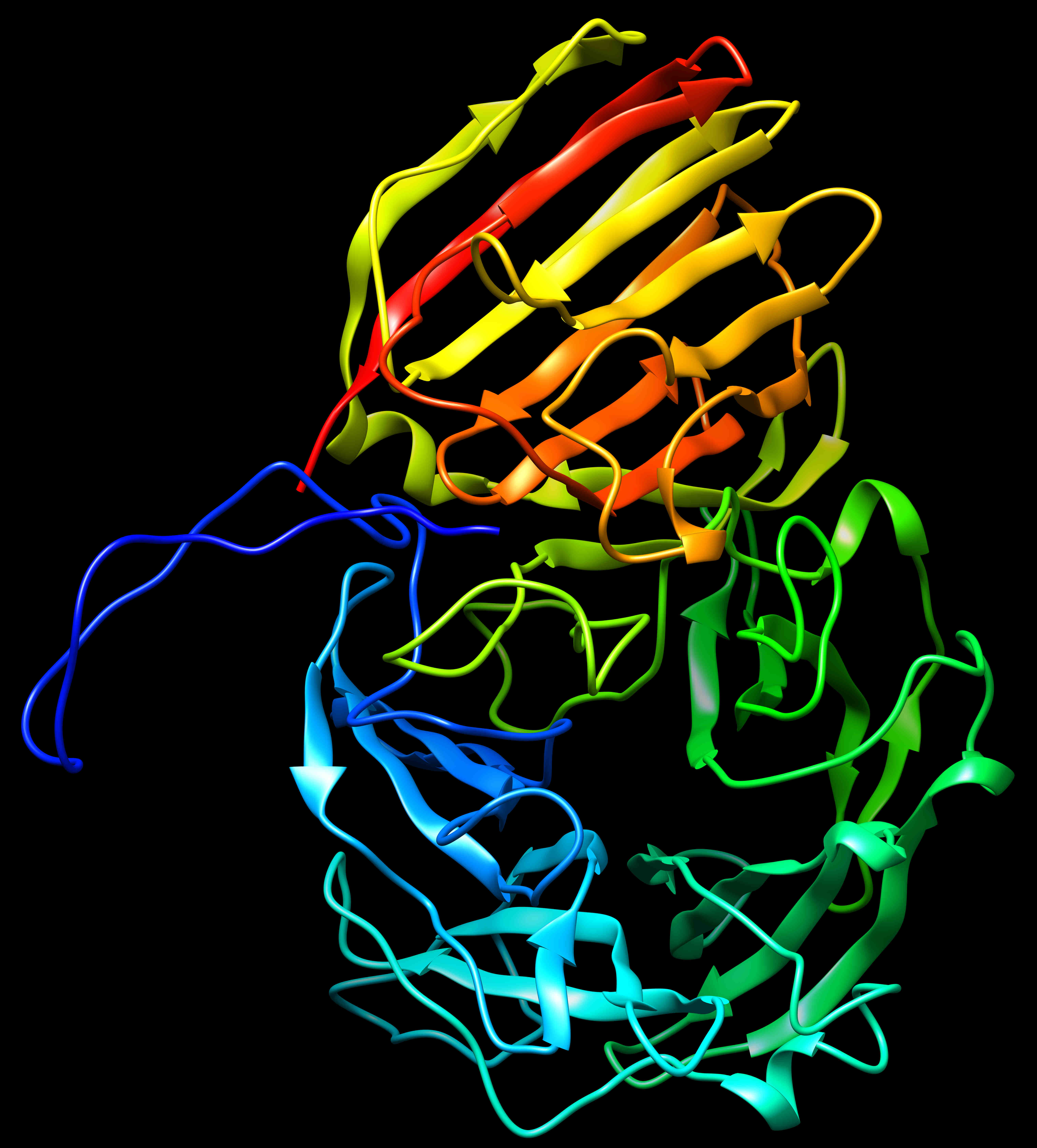

Supplement: S2 Dataset — (ZIP) [file pone.0200607.s002.zip › Abinitio_Models/TCP2.jpg]

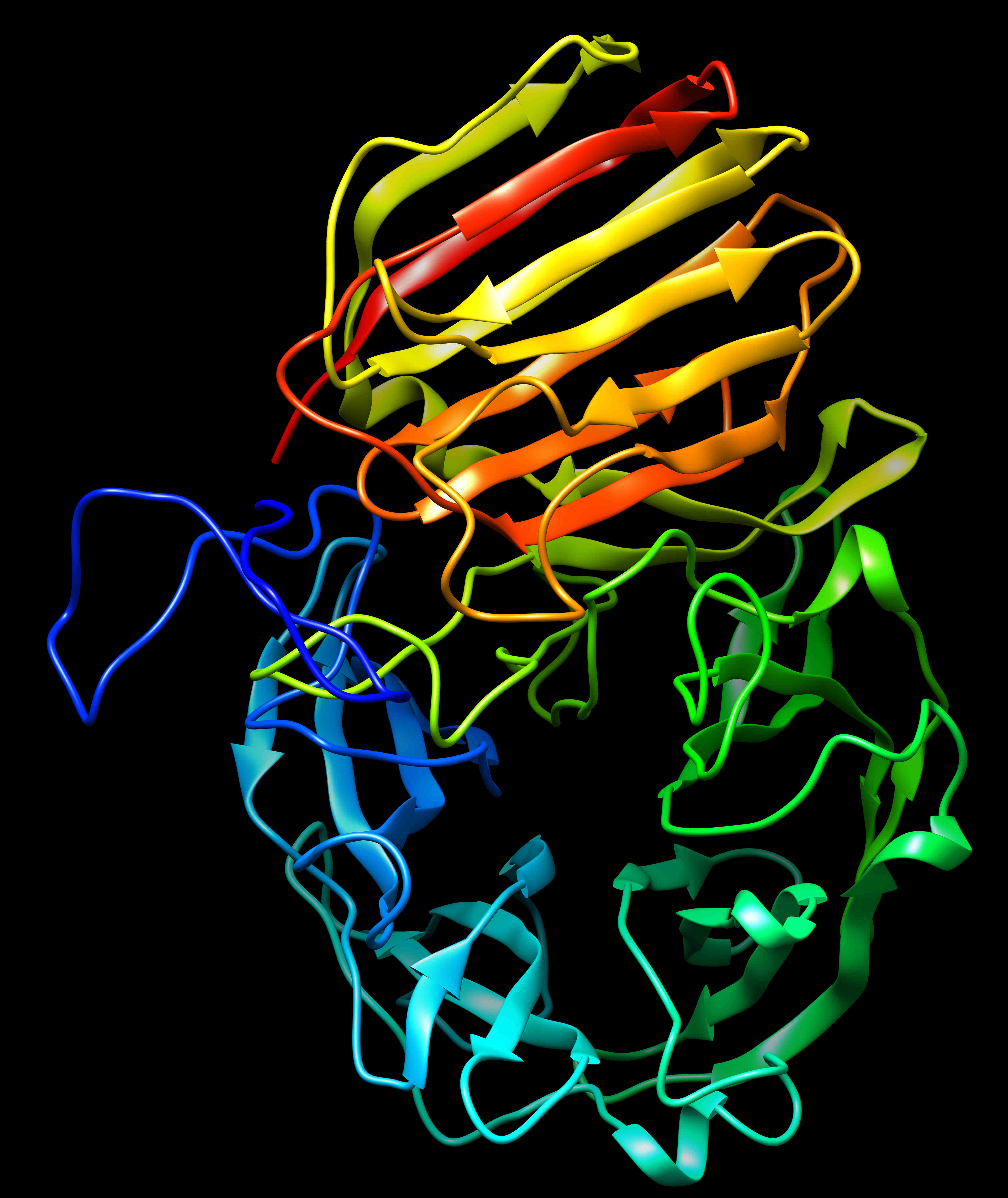

Supplement: S2 Dataset — (ZIP) [file pone.0200607.s002.zip › Abinitio_Models/TIP1.jpg]

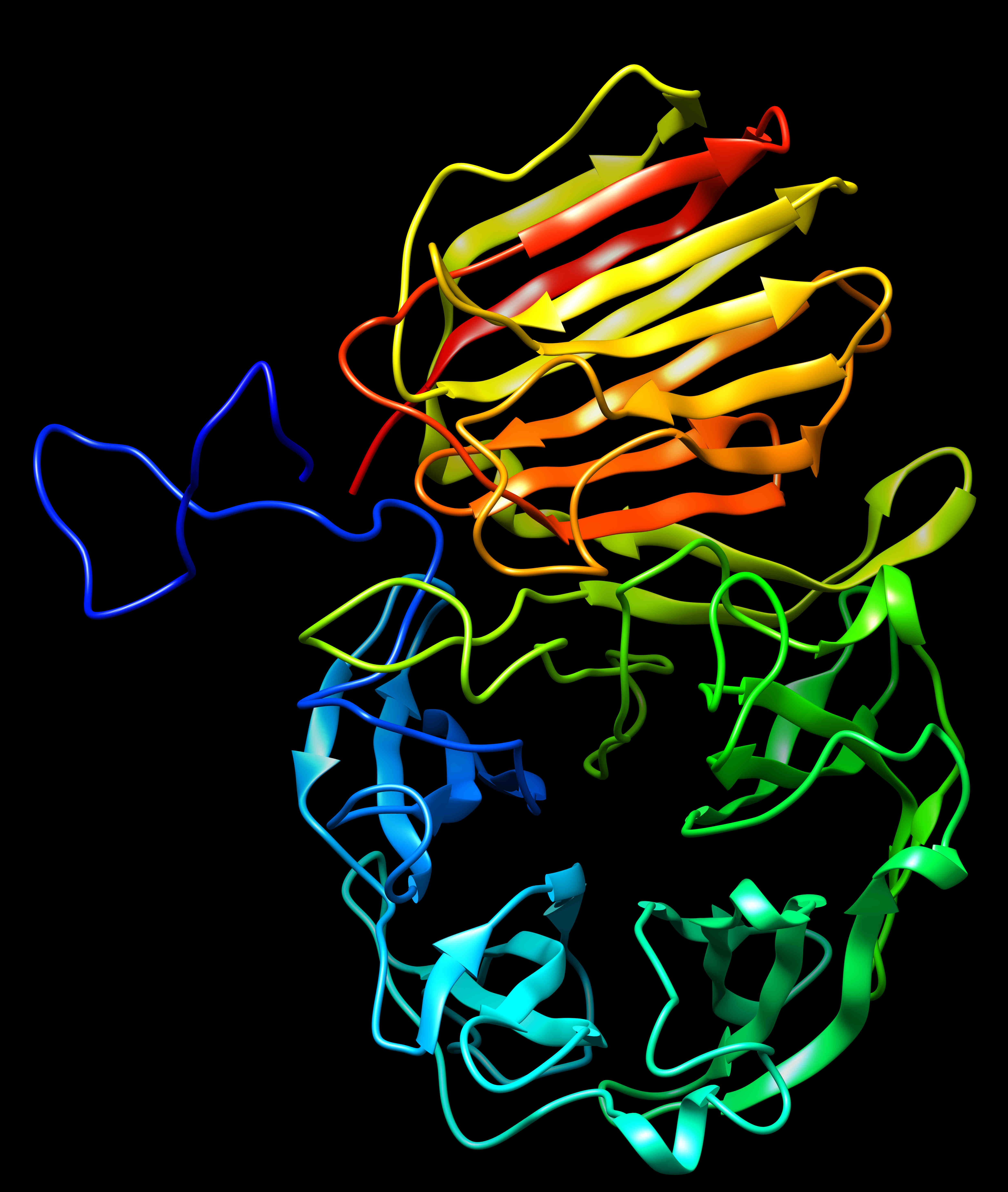

Supplement: S2 Dataset — (ZIP) [file pone.0200607.s002.zip › Abinitio_Models/TPP1.jpg]

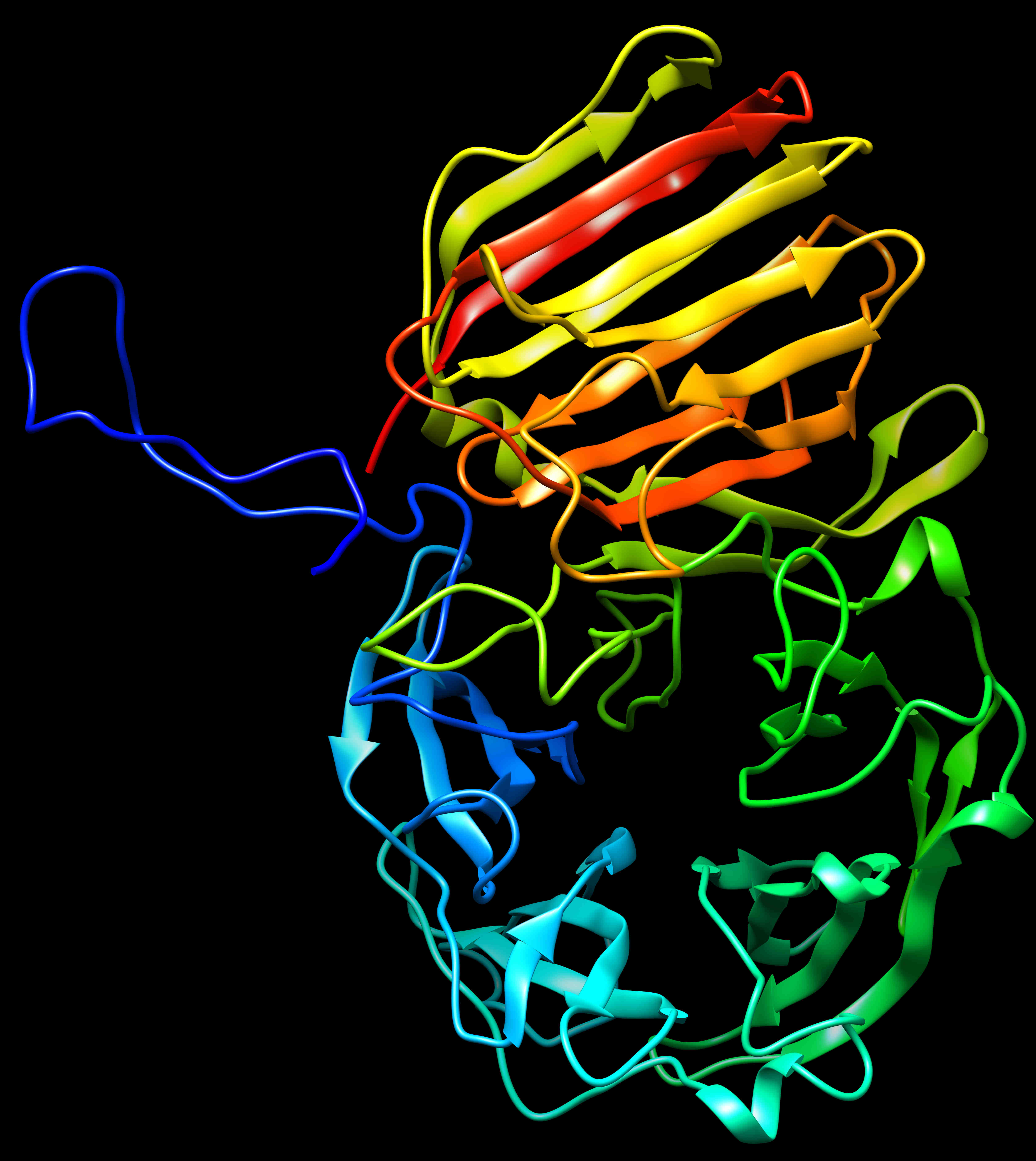

Supplement: S2 Dataset — (ZIP) [file pone.0200607.s002.zip › Abinitio_Models/TSP1.jpg]

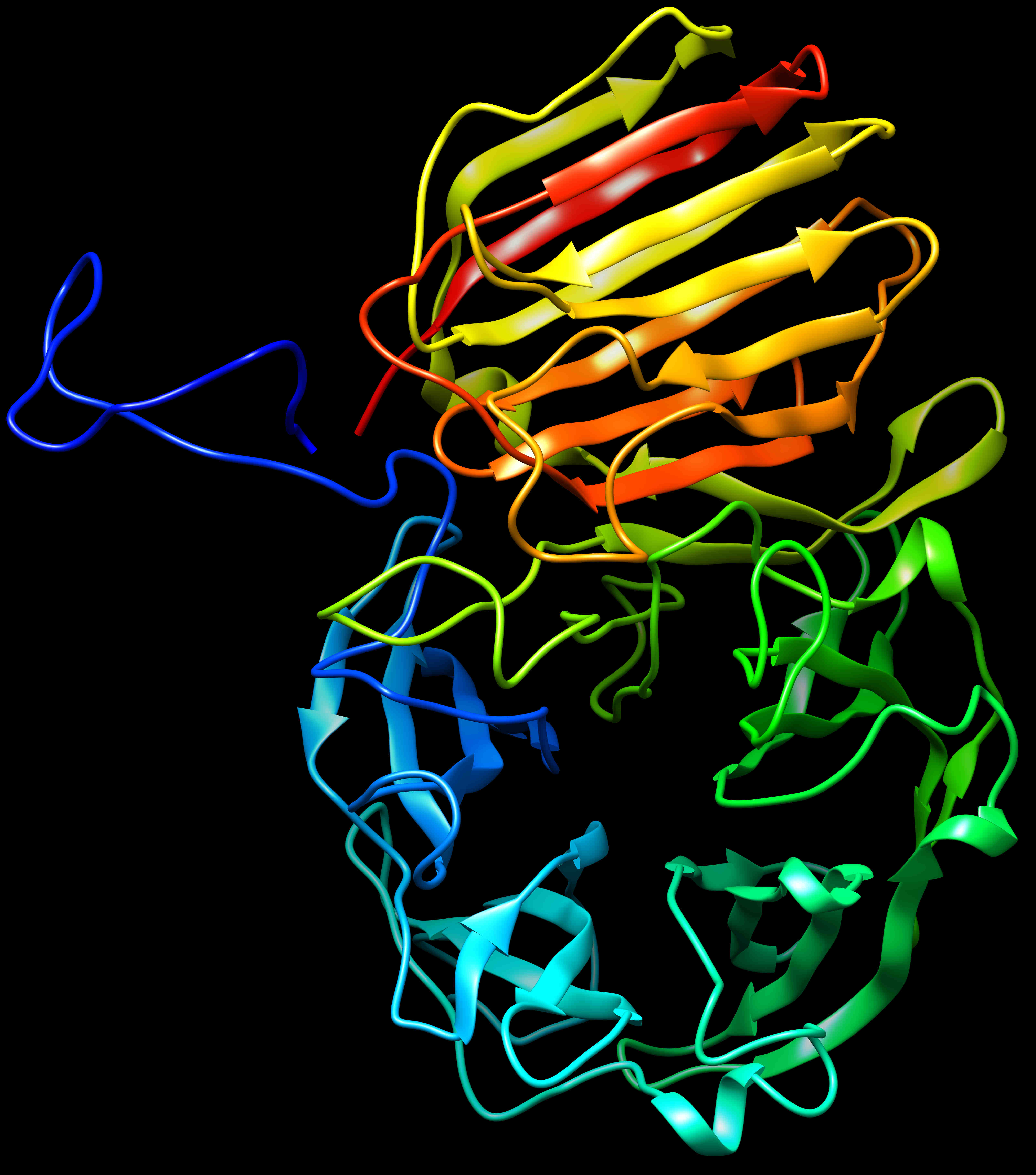

Supplement: S2 Dataset — (ZIP) [file pone.0200607.s002.zip › Abinitio_Models/TVP1.jpg]

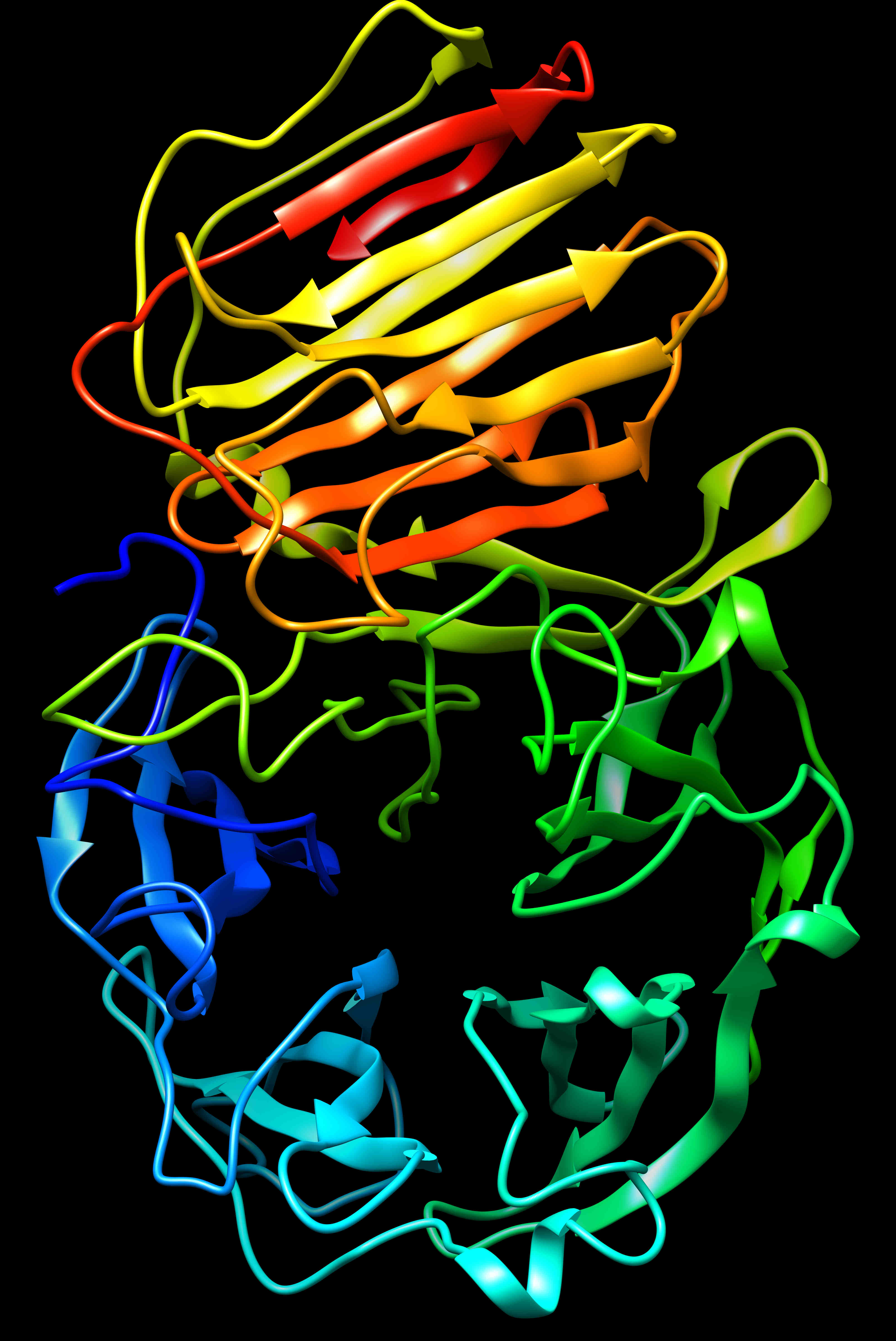

Supplement: S2 Dataset — (ZIP) [file pone.0200607.s002.zip › Abinitio_Models/TVP2.jpg]

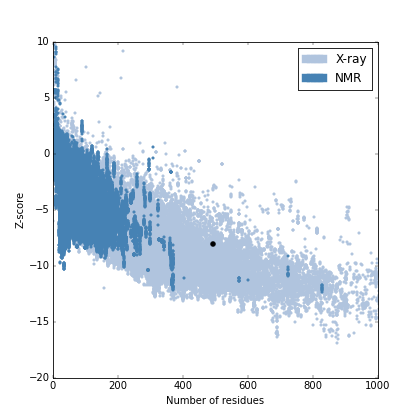

Supplement: S3 Dataset — (ZIP) [file pone.0200607.s003.zip › PROSA/3SC7.png]

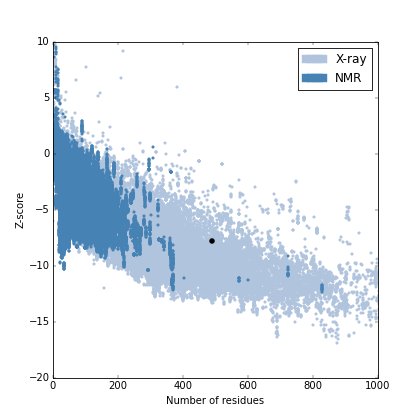

Supplement: S3 Dataset — (ZIP) [file pone.0200607.s003.zip › PROSA/A calidoustus p1m1 .png]

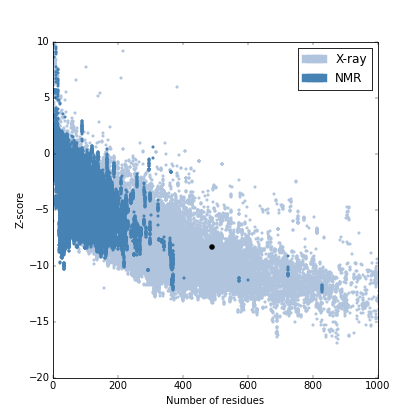

Supplement: S3 Dataset — (ZIP) [file pone.0200607.s003.zip › PROSA/A calidoustus p2m2 .png]

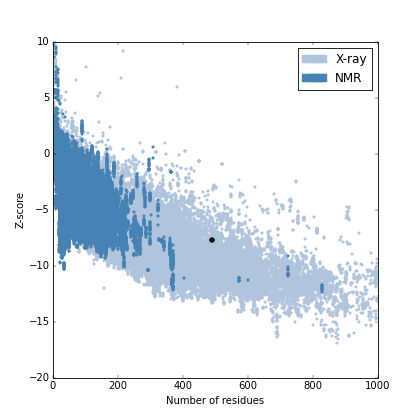

Supplement: S3 Dataset — (ZIP) [file pone.0200607.s003.zip › PROSA/A fischeri p1m2.png]

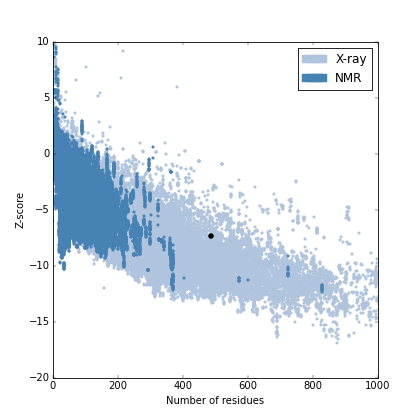

Supplement: S3 Dataset — (ZIP) [file pone.0200607.s003.zip › PROSA/A fumigatus p1m1.png]

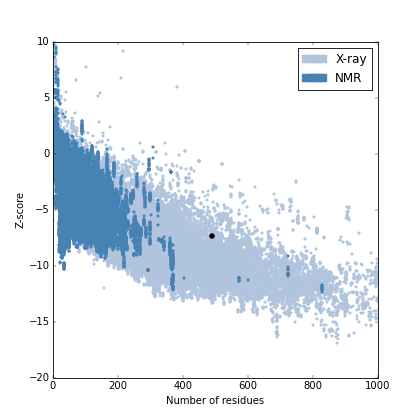

Supplement: S3 Dataset — (ZIP) [file pone.0200607.s003.zip › PROSA/A fumigatus p2m1.png]

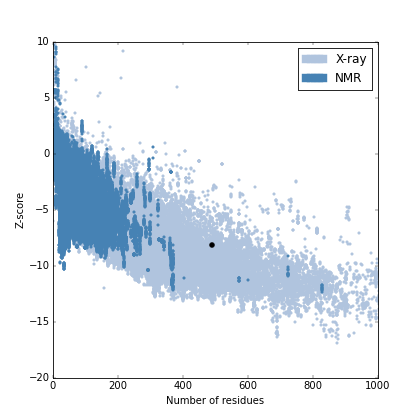

Supplement: S3 Dataset — (ZIP) [file pone.0200607.s003.zip › PROSA/A lentulus p1m2.png]

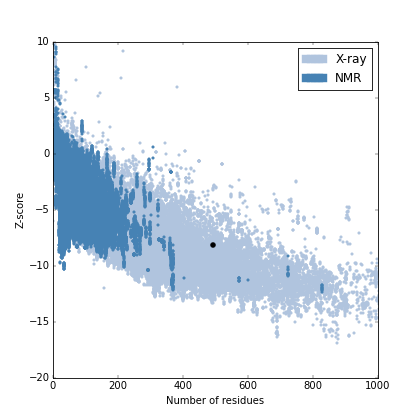

Supplement: S3 Dataset — (ZIP) [file pone.0200607.s003.zip › PROSA/A niger p1m1.png]

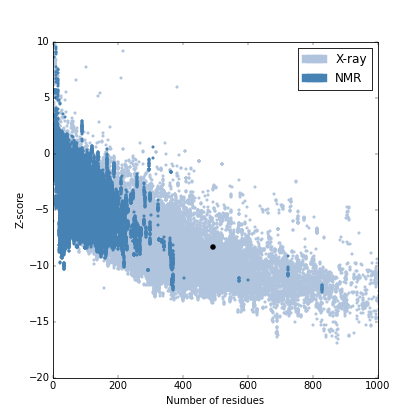

Supplement: S3 Dataset — (ZIP) [file pone.0200607.s003.zip › PROSA/A niger p2m1.png]

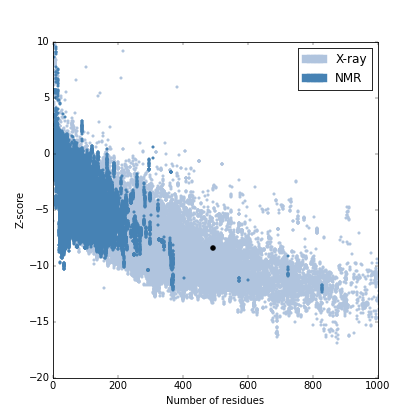

Supplement: S3 Dataset — (ZIP) [file pone.0200607.s003.zip › PROSA/A niger p4m1.png]

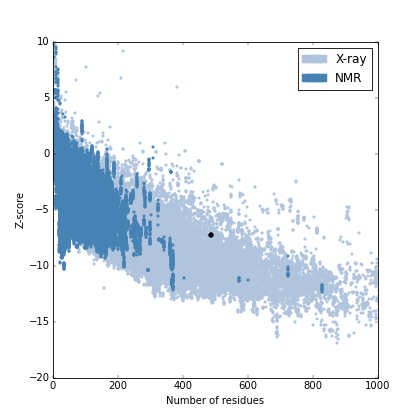

Supplement: S3 Dataset — (ZIP) [file pone.0200607.s003.zip › PROSA/A rugulosus p1m1.png]

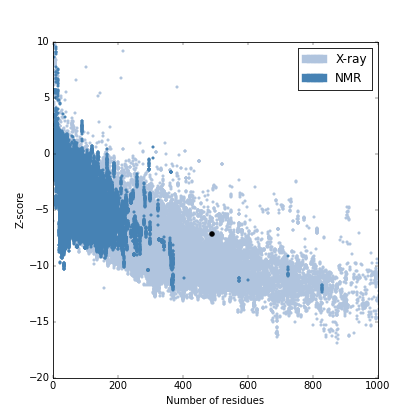

Supplement: S3 Dataset — (ZIP) [file pone.0200607.s003.zip › PROSA/B cenerea p1m1.png]

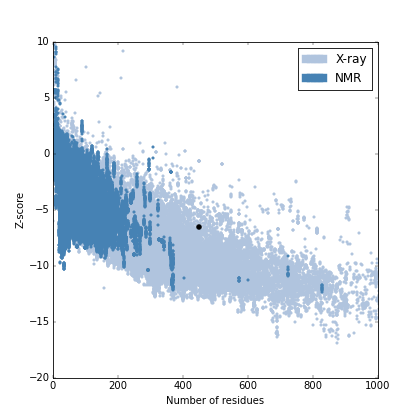

Supplement: S3 Dataset — (ZIP) [file pone.0200607.s003.zip › PROSA/B cenerea p2m1.png]

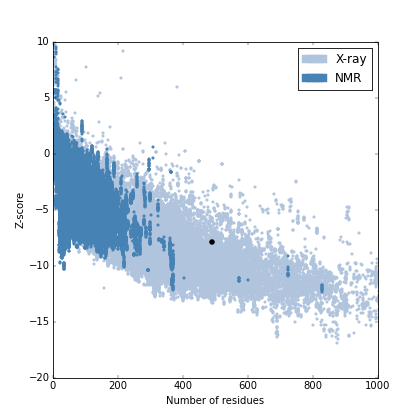

Supplement: S3 Dataset — (ZIP) [file pone.0200607.s003.zip › PROSA/F oxysporum hypo p7m1.png]

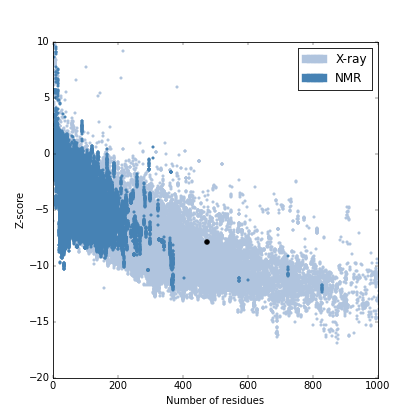

Supplement: S3 Dataset — (ZIP) [file pone.0200607.s003.zip › PROSA/F oxysporum inu p12m1.png]

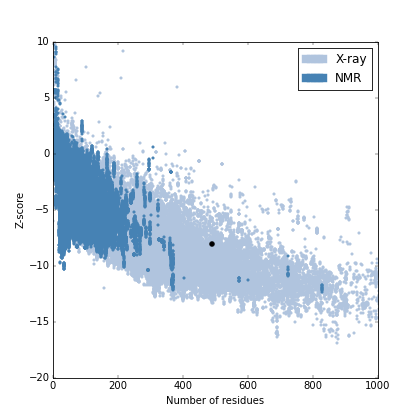

Supplement: S3 Dataset — (ZIP) [file pone.0200607.s003.zip › PROSA/F oxysporum inu p8m1.png]

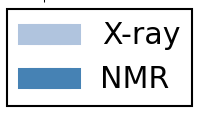

Supplement: S3 Dataset — (ZIP) [file pone.0200607.s003.zip › PROSA/Legend.tiff]

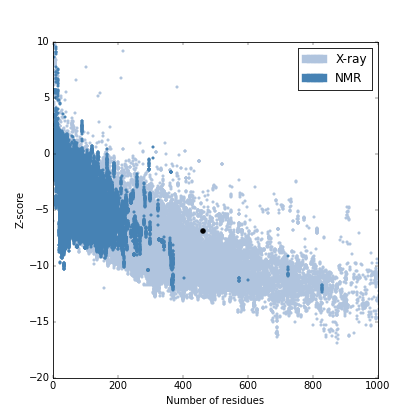

Supplement: S3 Dataset — (ZIP) [file pone.0200607.s003.zip › PROSA/M phaseolina p1m1.png]

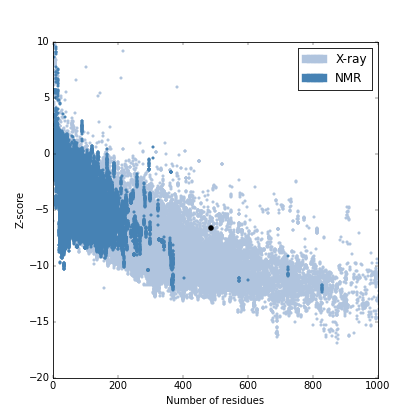

Supplement: S3 Dataset — (ZIP) [file pone.0200607.s003.zip › PROSA/O maius p1m2.png]

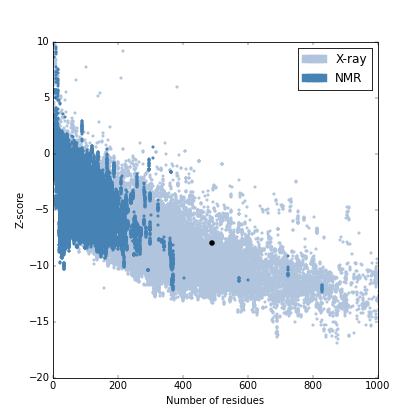

Supplement: S3 Dataset — (ZIP) [file pone.0200607.s003.zip › PROSA/P brasilianum p1m2.png]

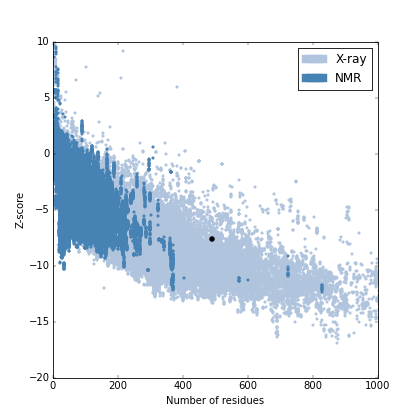

Supplement: S3 Dataset — (ZIP) [file pone.0200607.s003.zip › PROSA/P nordicum p1m1.png]

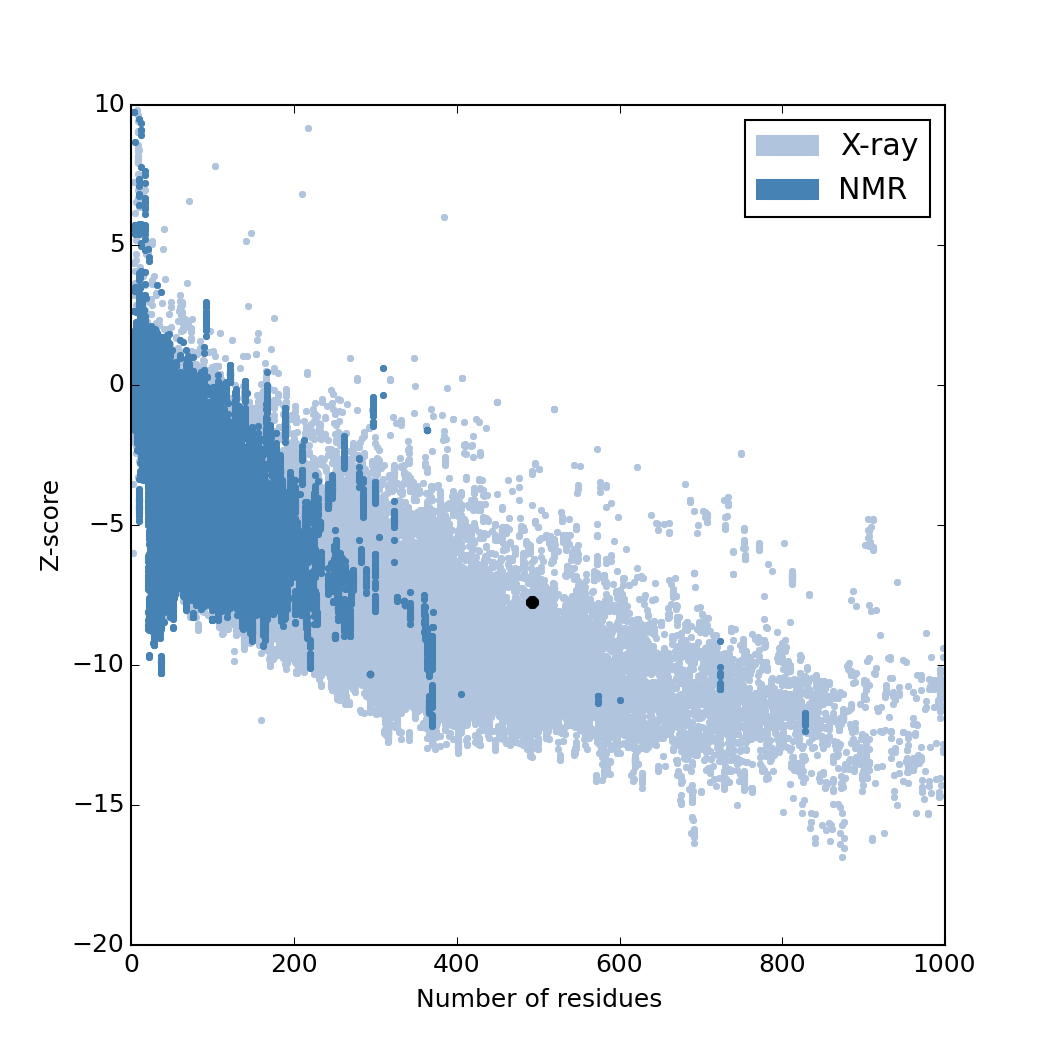

Supplement: S3 Dataset — (ZIP) [file pone.0200607.s003.zip › PROSA/PROSA_P_subrubescens_p1m1.tiff]

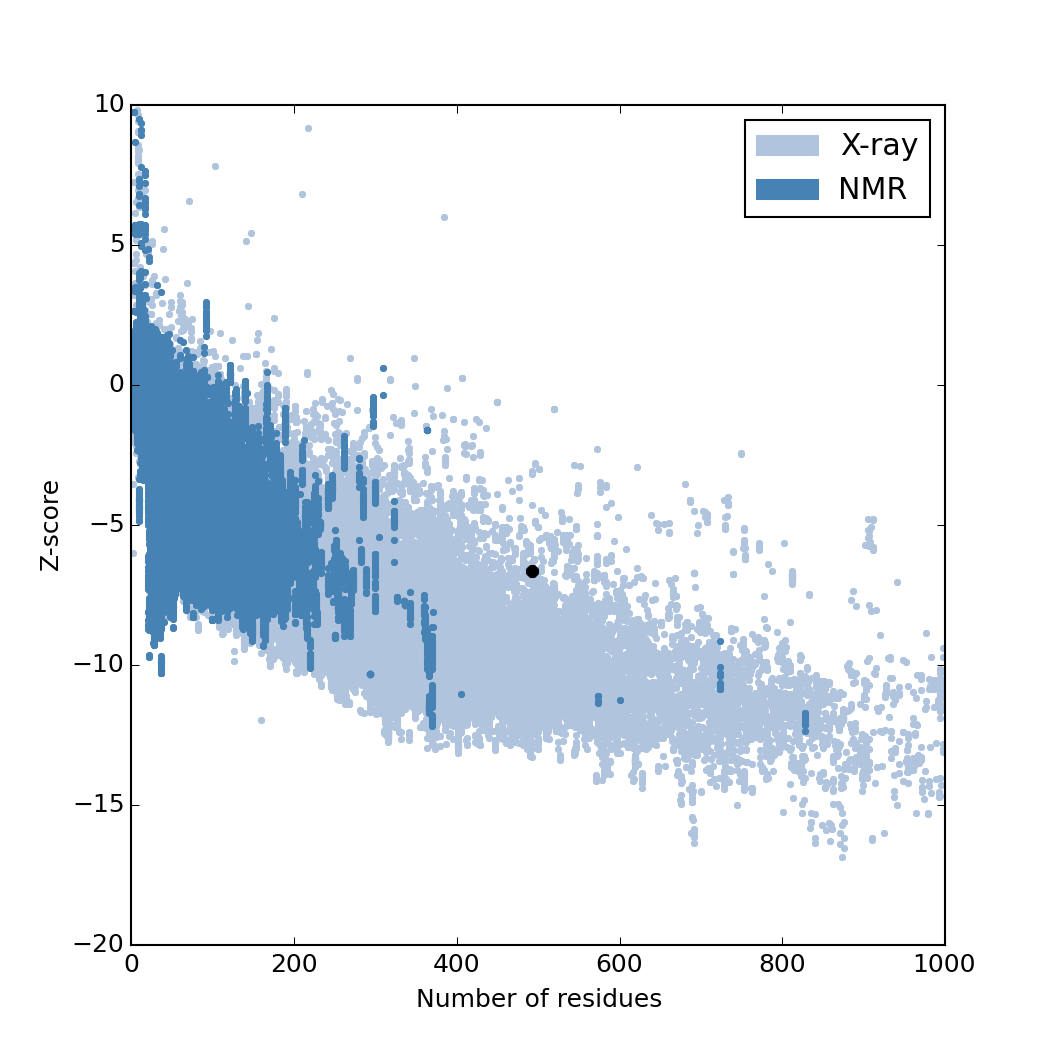

Supplement: S3 Dataset — (ZIP) [file pone.0200607.s003.zip › PROSA/PROSA_P_subrubescens_p2m1.tiff]

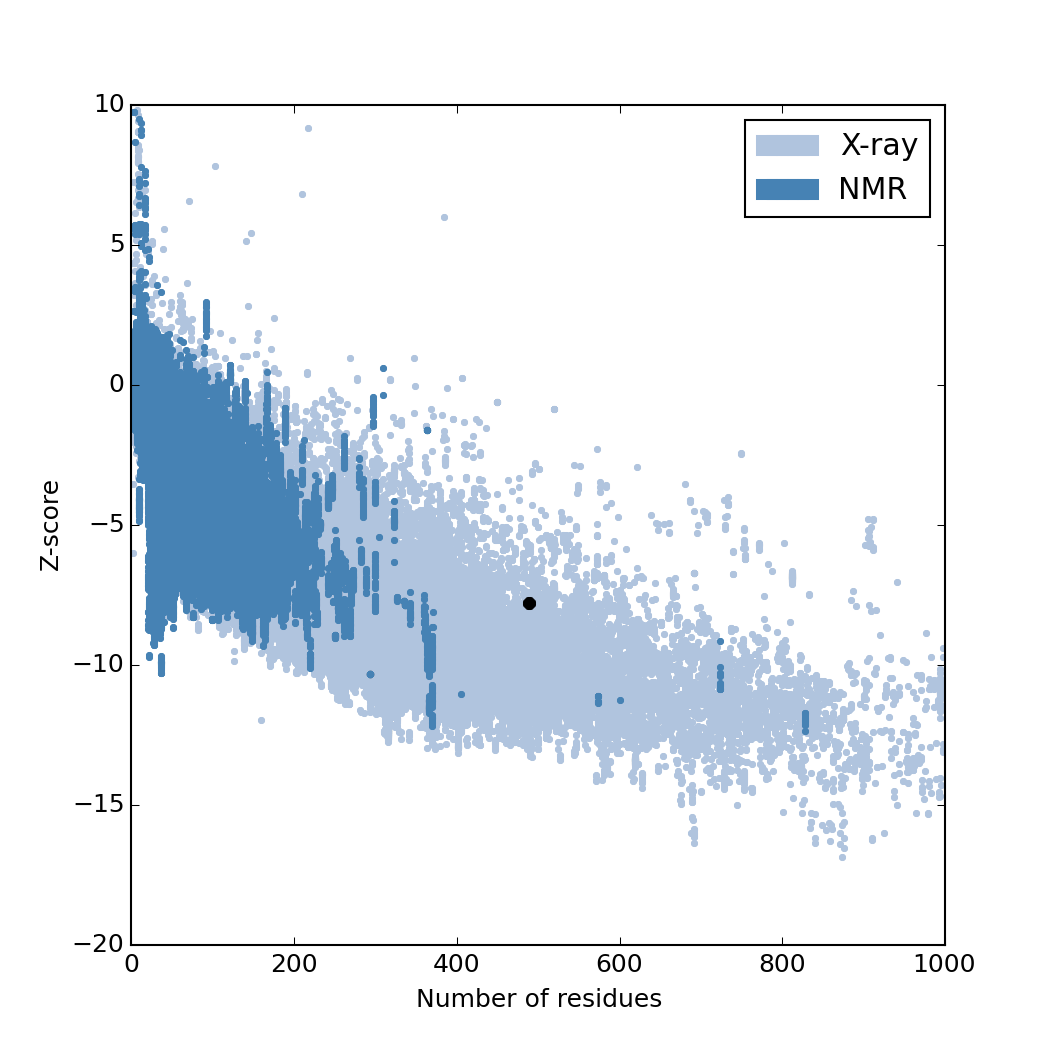

Supplement: S3 Dataset — (ZIP) [file pone.0200607.s003.zip › PROSA/PROSA_P_subrubescens_p3m2.tiff]

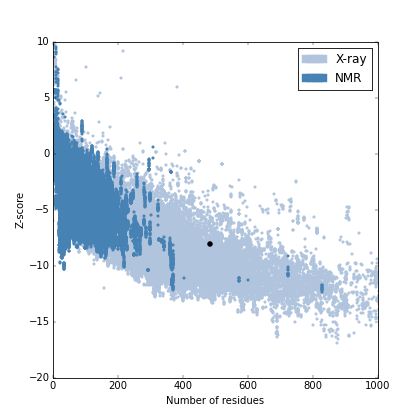

Supplement: S3 Dataset — (ZIP) [file pone.0200607.s003.zip › PROSA/Pseudogymnoascus p1m2.png]

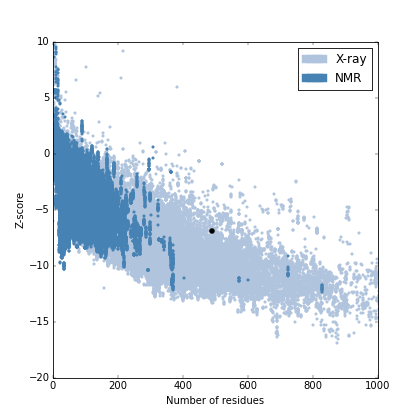

Supplement: S3 Dataset — (ZIP) [file pone.0200607.s003.zip › PROSA/Pseudogymnoascus p3m2.png]

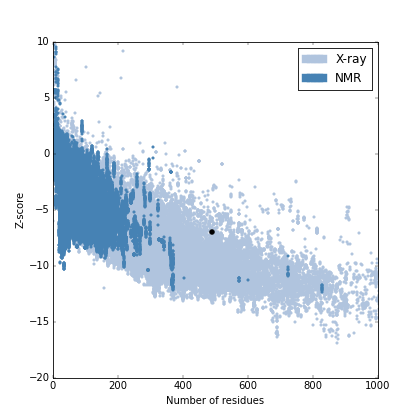

Supplement: S3 Dataset — (ZIP) [file pone.0200607.s003.zip › PROSA/Pseudogymnoascus p4m2.png]

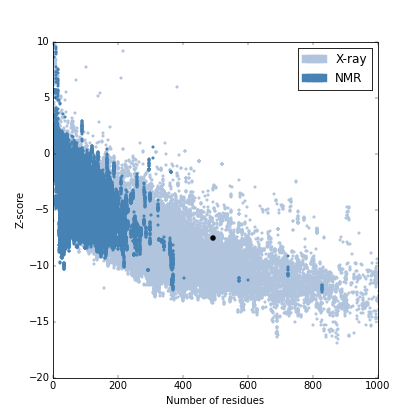

Supplement: S3 Dataset — (ZIP) [file pone.0200607.s003.zip › PROSA/Pyrenochaeta p1m1.png]

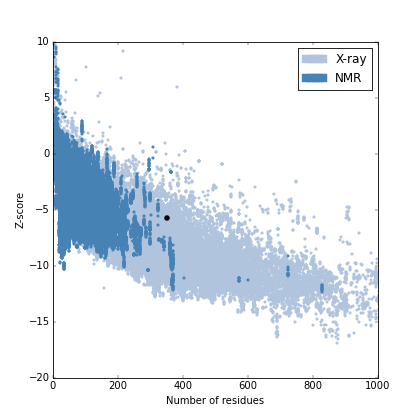

Supplement: S3 Dataset — (ZIP) [file pone.0200607.s003.zip › PROSA/S chartarum p18m2.png]

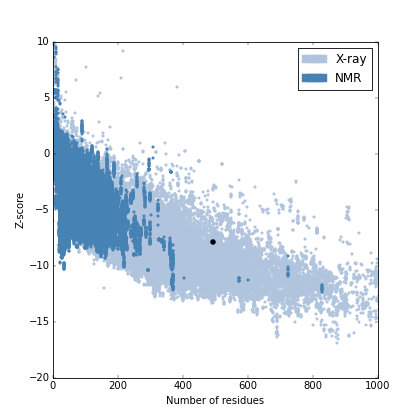

Supplement: S3 Dataset — (ZIP) [file pone.0200607.s003.zip › PROSA/S chartarum p1m1.png]

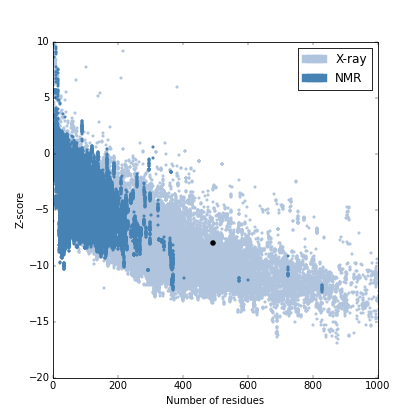

Supplement: S3 Dataset — (ZIP) [file pone.0200607.s003.zip › PROSA/S chartarum p2m1.png]

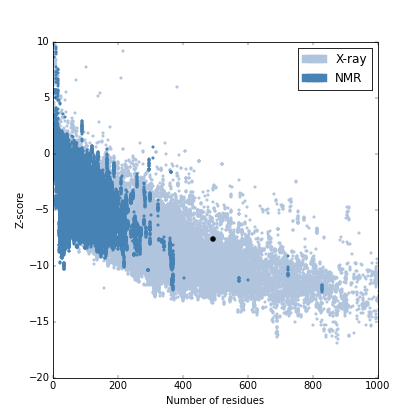

Supplement: S3 Dataset — (ZIP) [file pone.0200607.s003.zip › PROSA/S chartarum p4m2.png]

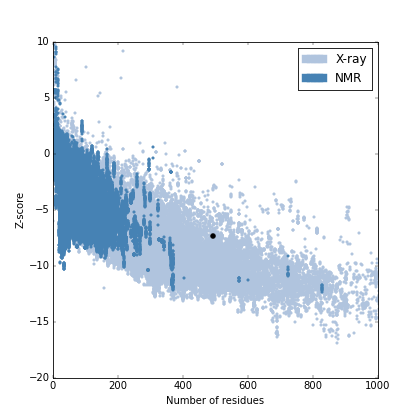

Supplement: S3 Dataset — (ZIP) [file pone.0200607.s003.zip › PROSA/S chartarum p5m2.png]

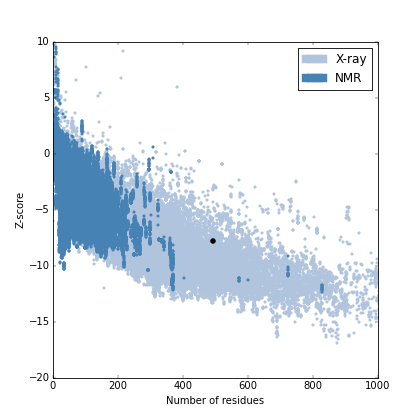

Supplement: S3 Dataset — (ZIP) [file pone.0200607.s003.zip › PROSA/S chartarum p6m2.png]

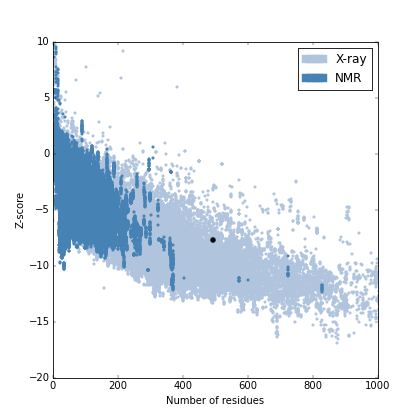

Supplement: S3 Dataset — (ZIP) [file pone.0200607.s003.zip › PROSA/S chartarum p7m1.png]

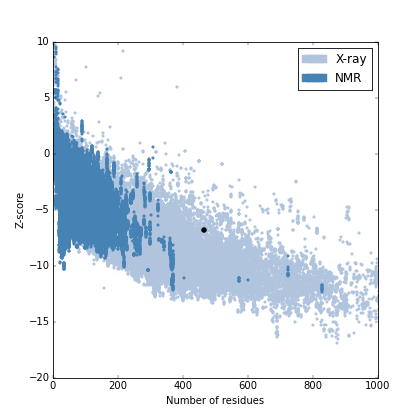

Supplement: S3 Dataset — (ZIP) [file pone.0200607.s003.zip › PROSA/T cellulolyticus p1m2.png]

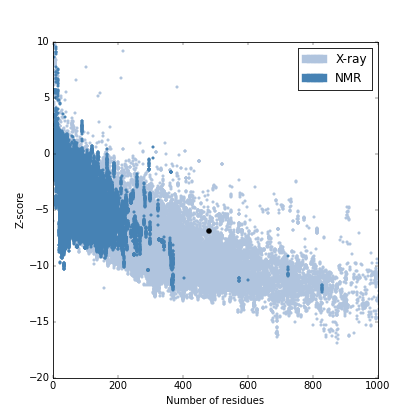

Supplement: S3 Dataset — (ZIP) [file pone.0200607.s003.zip › PROSA/T stipitatus p1m1.png]

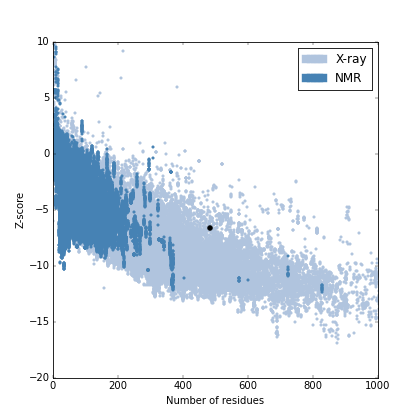

Supplement: S3 Dataset — (ZIP) [file pone.0200607.s003.zip › PROSA/T verruculosus p2m2.png]

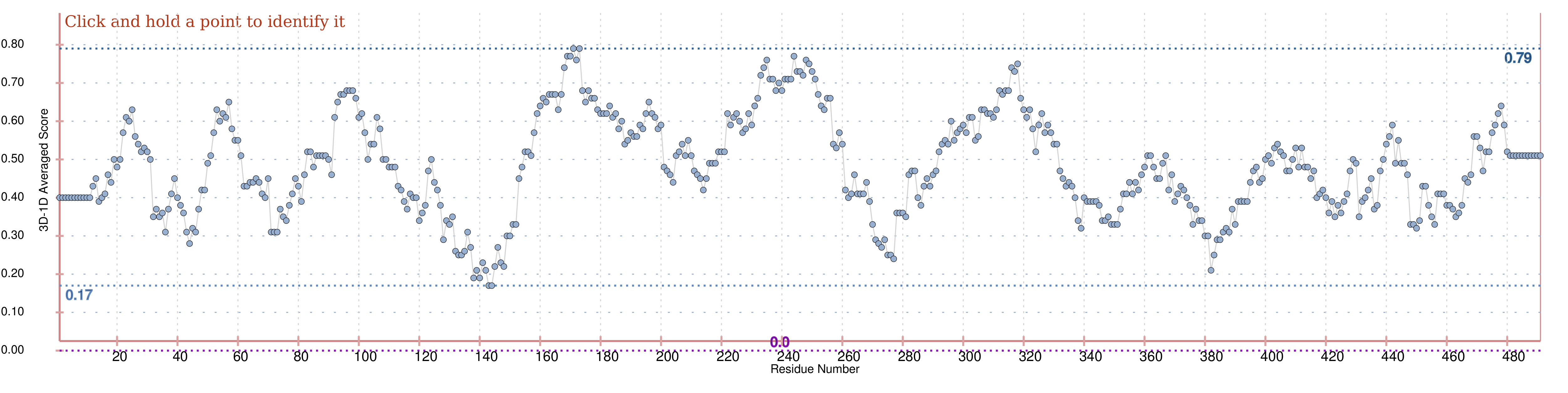

Supplement: S4 Dataset — (ZIP) [file pone.0200607.s004.zip › verify_3d/A calidoustus p1 m1.tiff]

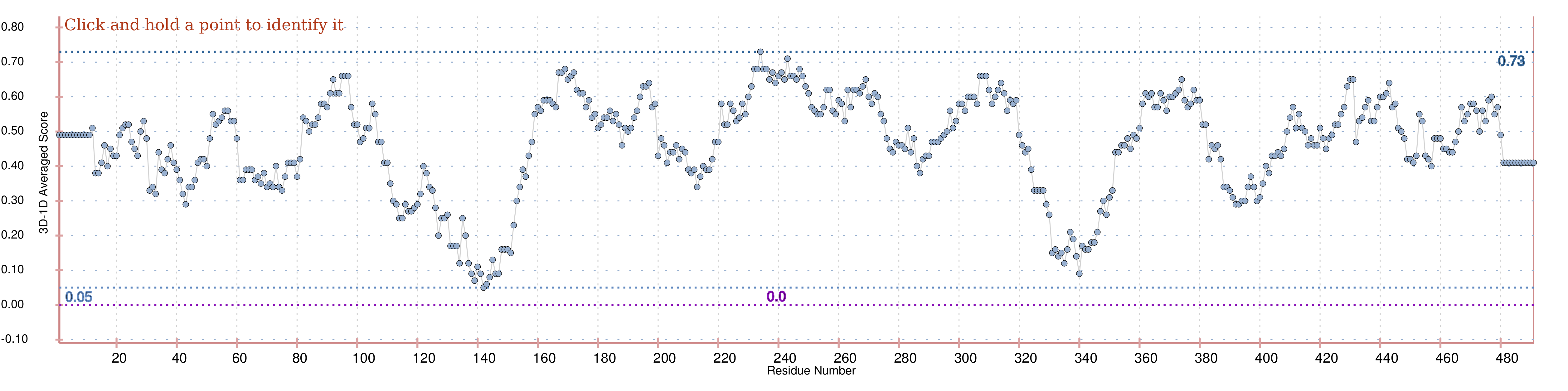

Supplement: S4 Dataset — (ZIP) [file pone.0200607.s004.zip › verify_3d/A calidoustus p2 m2.tiff]

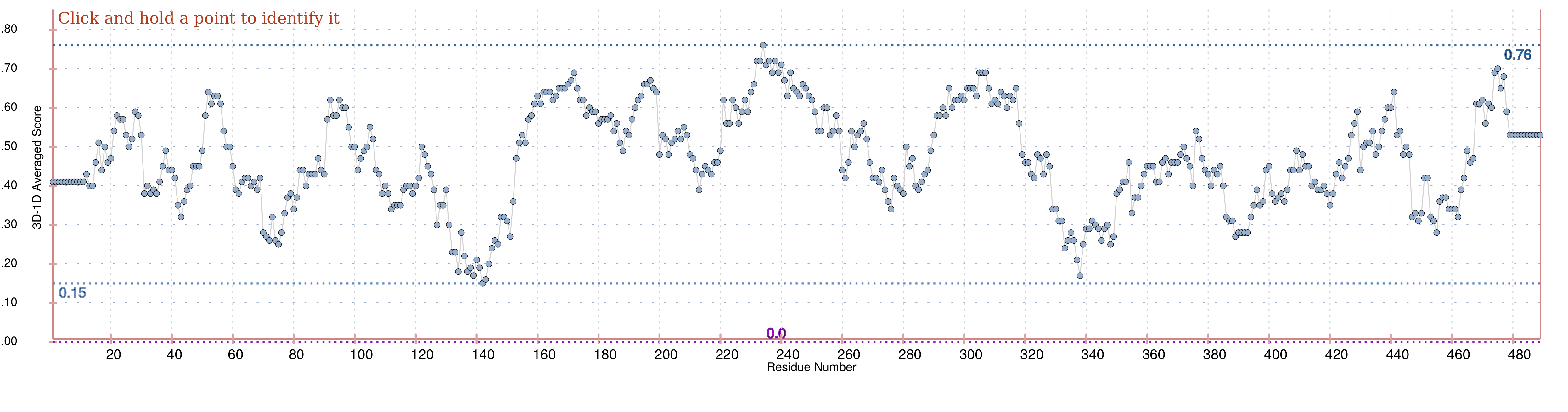

Supplement: S4 Dataset — (ZIP) [file pone.0200607.s004.zip › verify_3d/A fischeri NRRL 181 p1 m2.tiff]

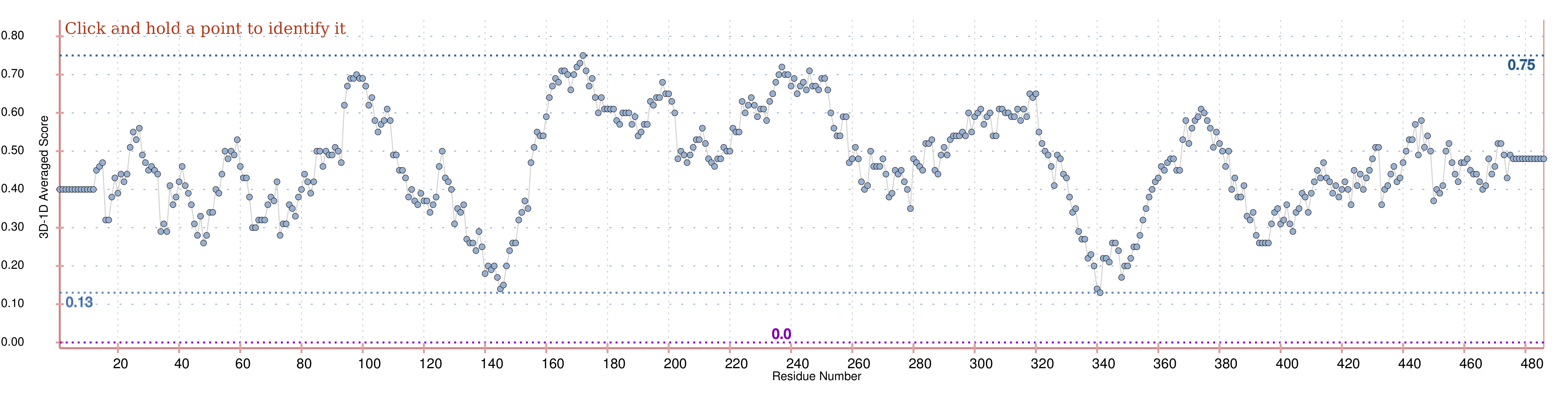

Supplement: S4 Dataset — (ZIP) [file pone.0200607.s004.zip › verify_3d/A rugulosus p1 m1.tiff]

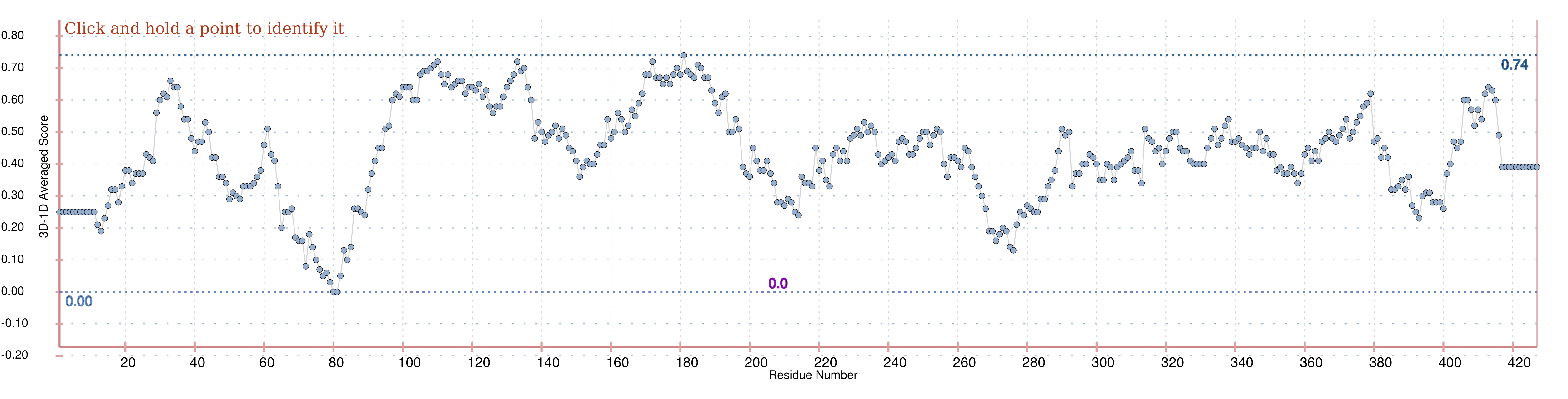

Supplement: S4 Dataset — (ZIP) [file pone.0200607.s004.zip › verify_3d/A udagawae p1 m2.tiff]

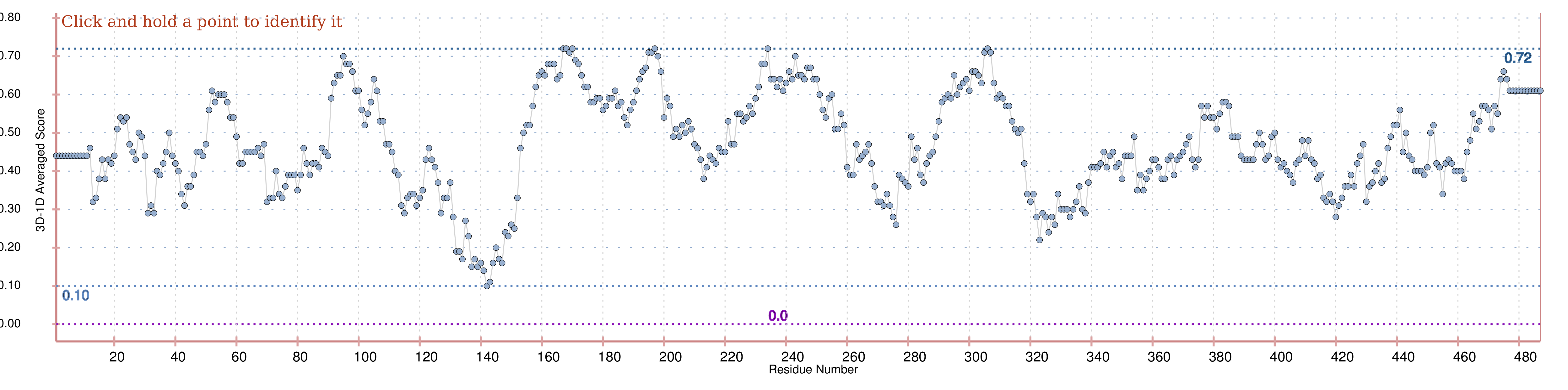

Supplement: S4 Dataset — (ZIP) [file pone.0200607.s004.zip › verify_3d/A. fumigatus af293 p1m1.tiff]

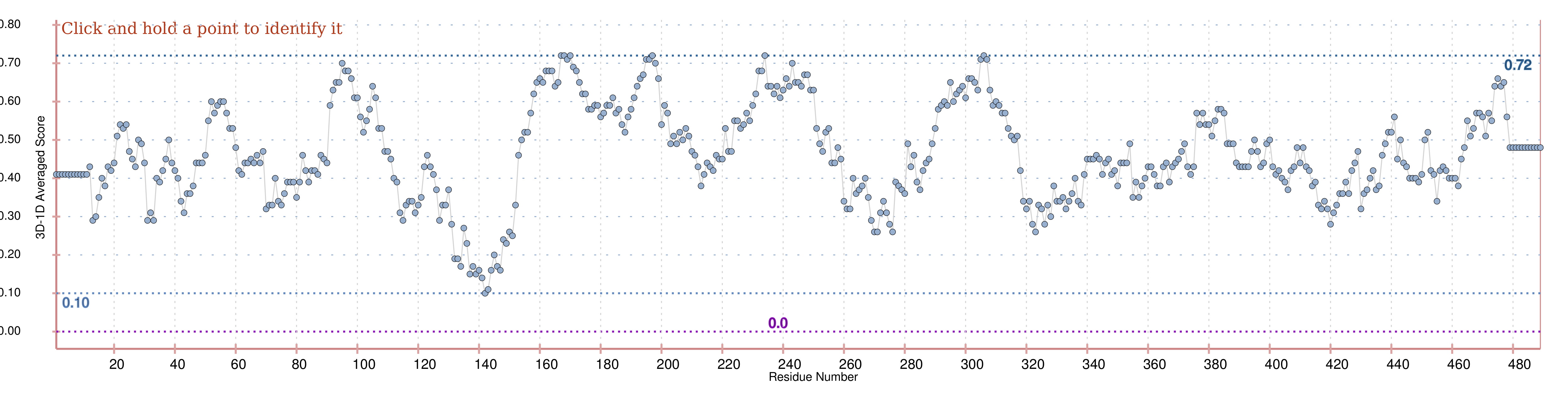

Supplement: S4 Dataset — (ZIP) [file pone.0200607.s004.zip › verify_3d/A. fumigatus z5 p2m1.tiff]

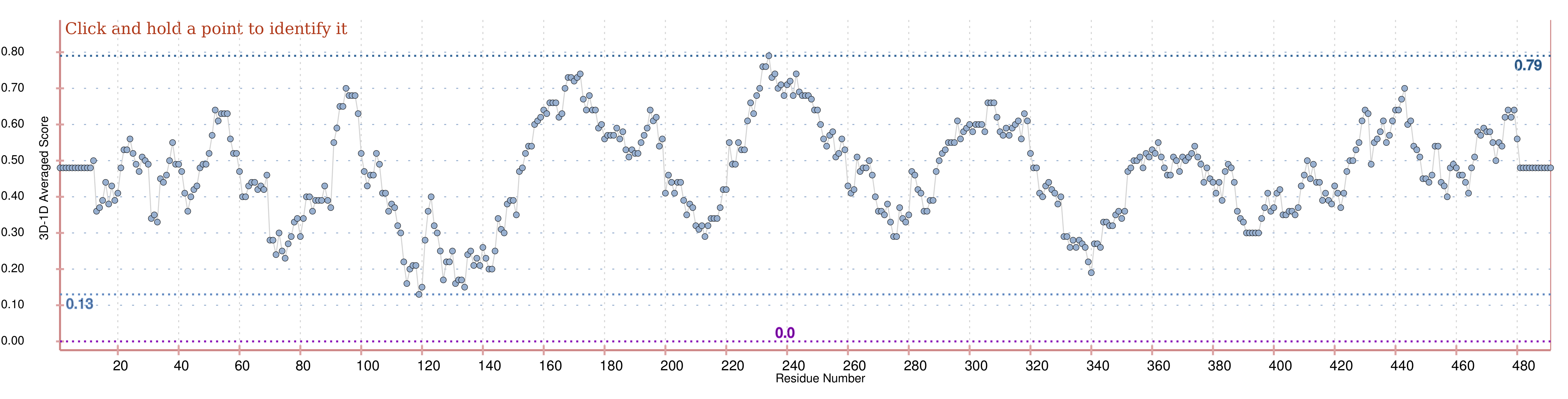

Supplement: S4 Dataset — (ZIP) [file pone.0200607.s004.zip › verify_3d/A. lentulus p1m2.tiff]

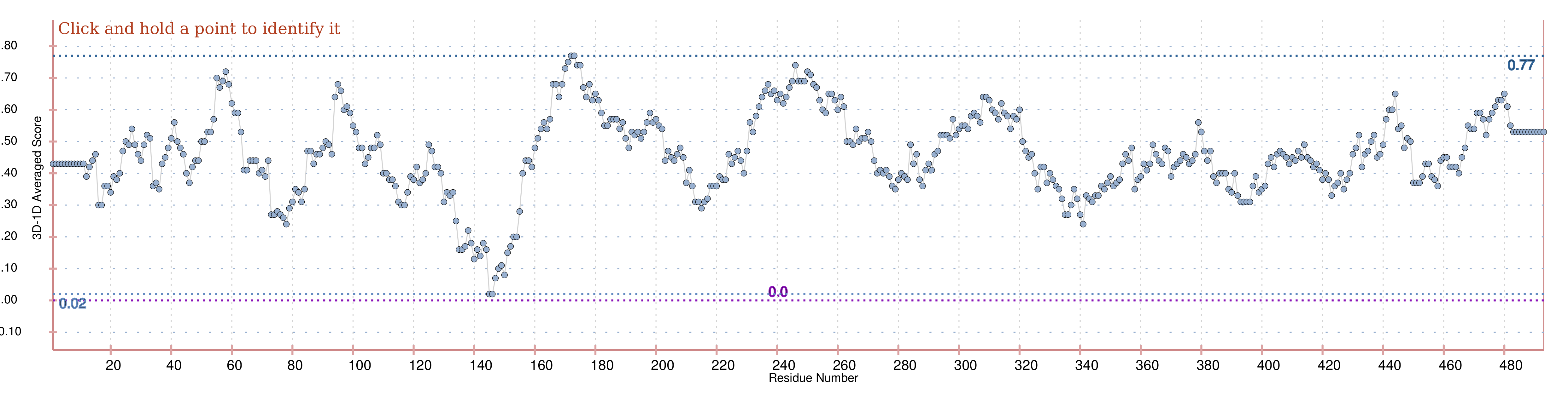

Supplement: S4 Dataset — (ZIP) [file pone.0200607.s004.zip › verify_3d/A. niger CBS 513.88 inu p7 m1.tiff]

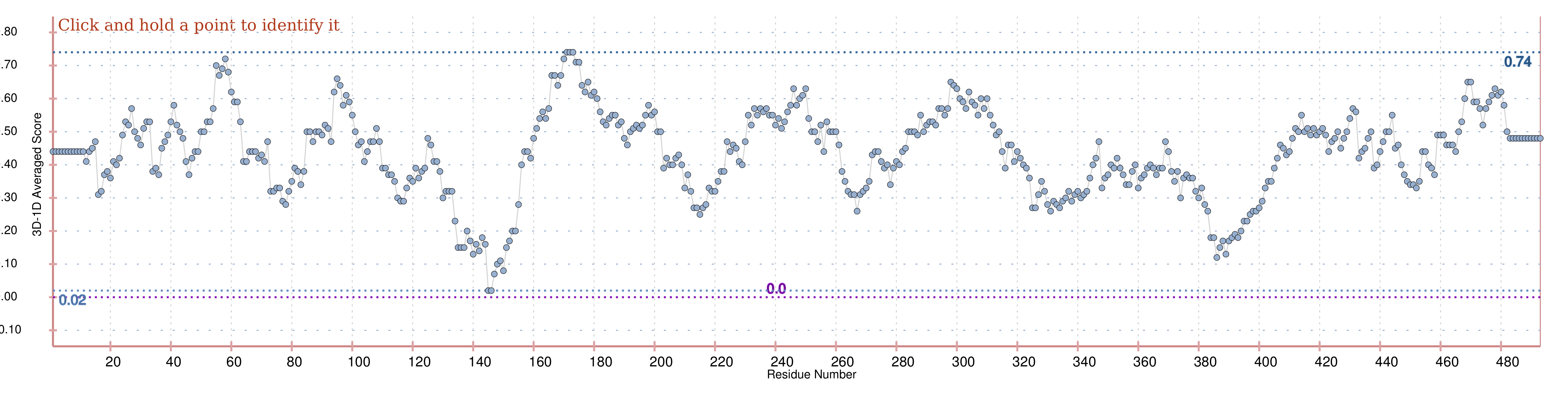

Supplement: S4 Dataset — (ZIP) [file pone.0200607.s004.zip › verify_3d/A. niger inu p10 m1.tiff]

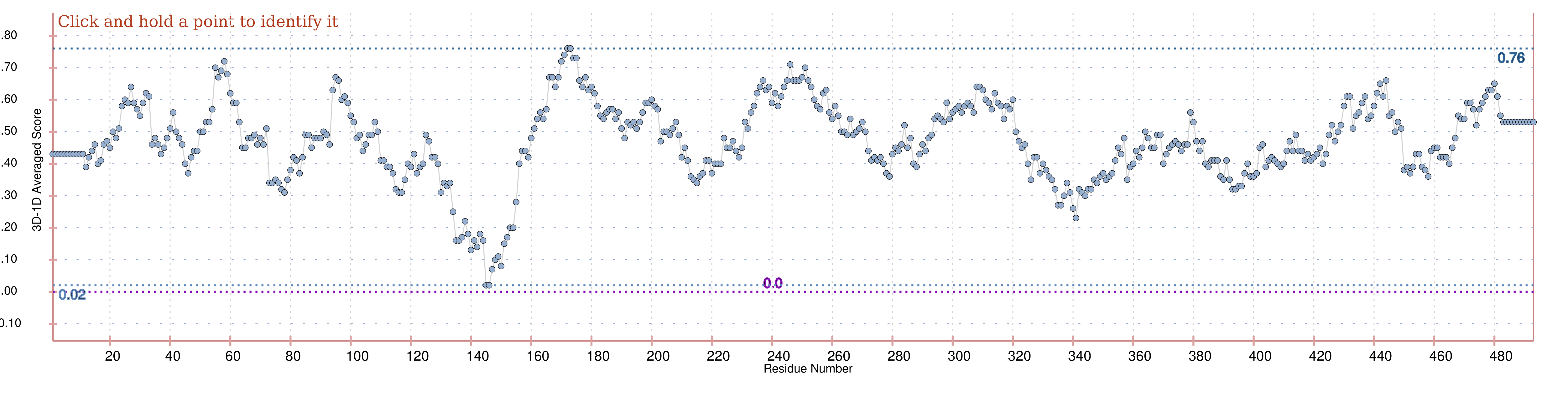

Supplement: S4 Dataset — (ZIP) [file pone.0200607.s004.zip › verify_3d/A. niger inu p2 m1.tiff]

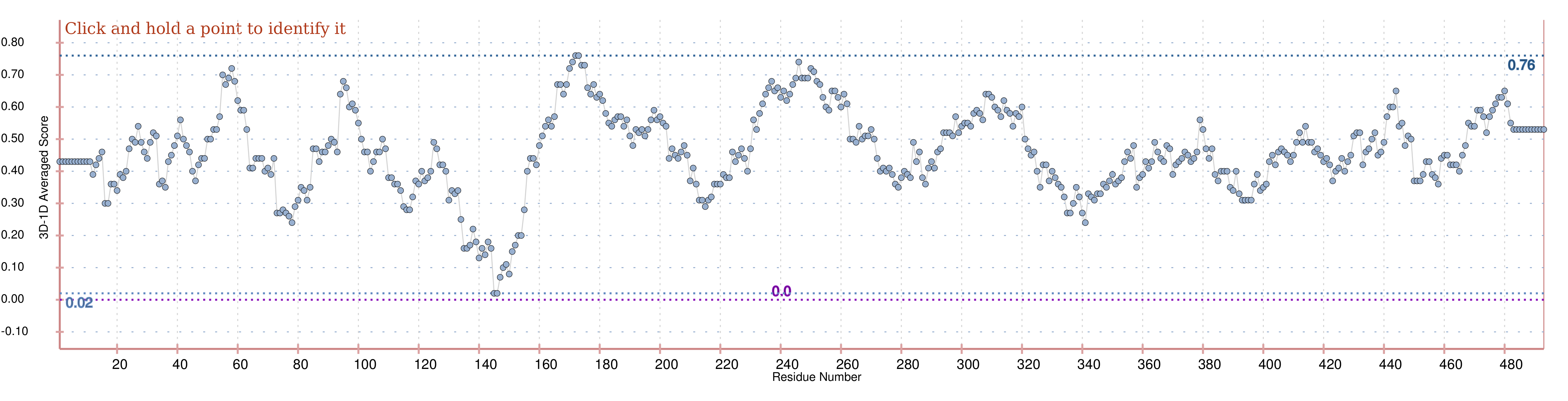

Supplement: S4 Dataset — (ZIP) [file pone.0200607.s004.zip › verify_3d/A. niger inu p3 m1.tiff]

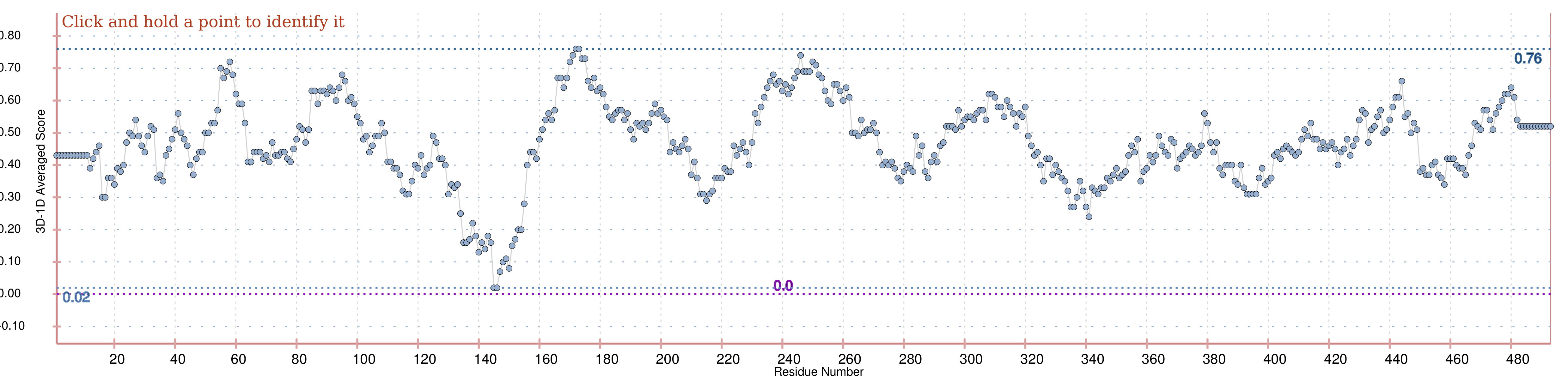

Supplement: S4 Dataset — (ZIP) [file pone.0200607.s004.zip › verify_3d/A. niger inu p4m1.tiff]

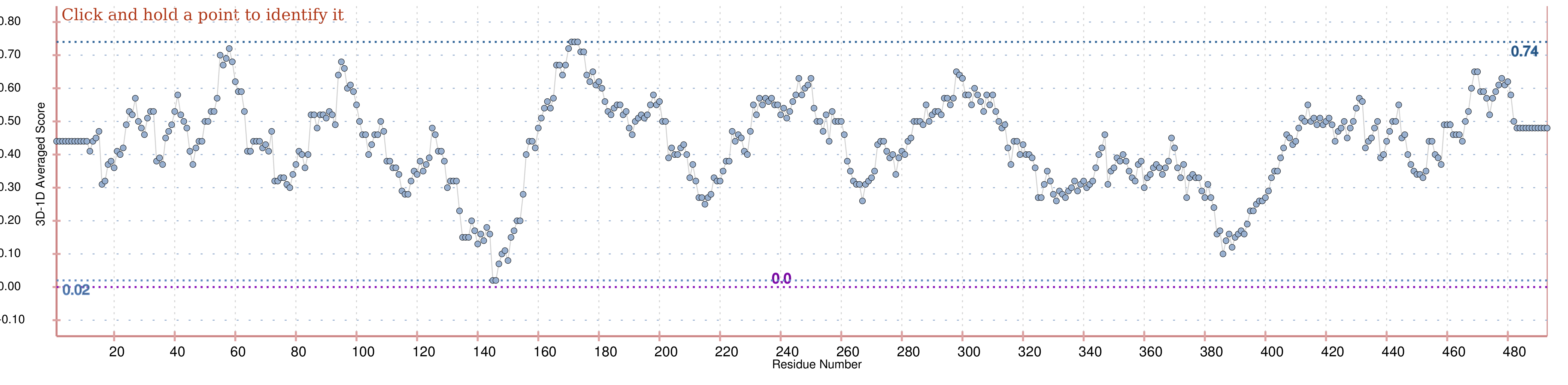

Supplement: S4 Dataset — (ZIP) [file pone.0200607.s004.zip › verify_3d/A. niger inu p5m1.tiff]

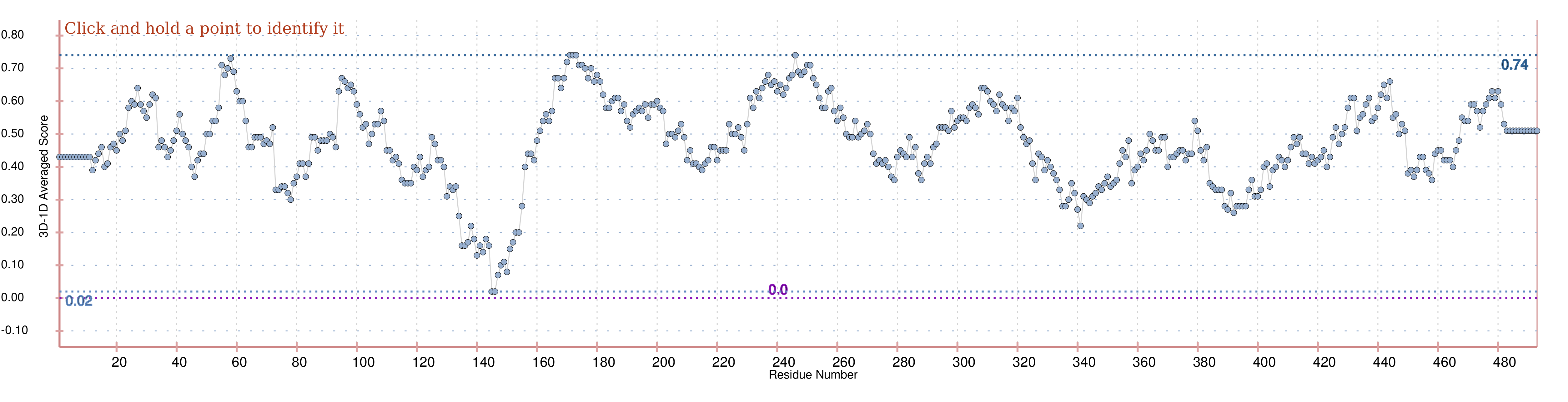

Supplement: S4 Dataset — (ZIP) [file pone.0200607.s004.zip › verify_3d/A. niger inu p9 m1.tiff]

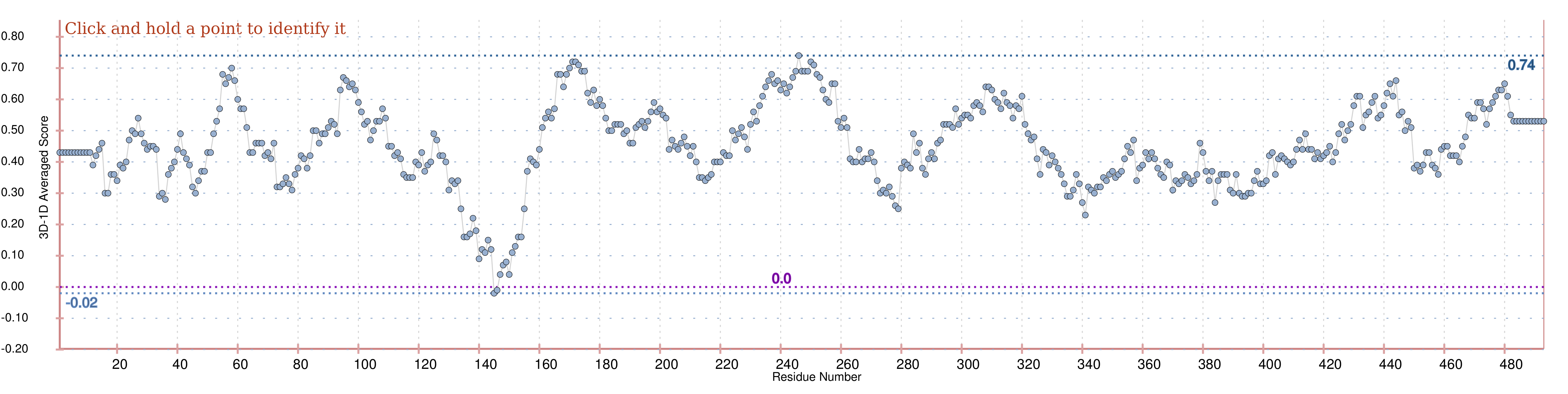

Supplement: S4 Dataset — (ZIP) [file pone.0200607.s004.zip › verify_3d/A. niger p1m1.tiff]

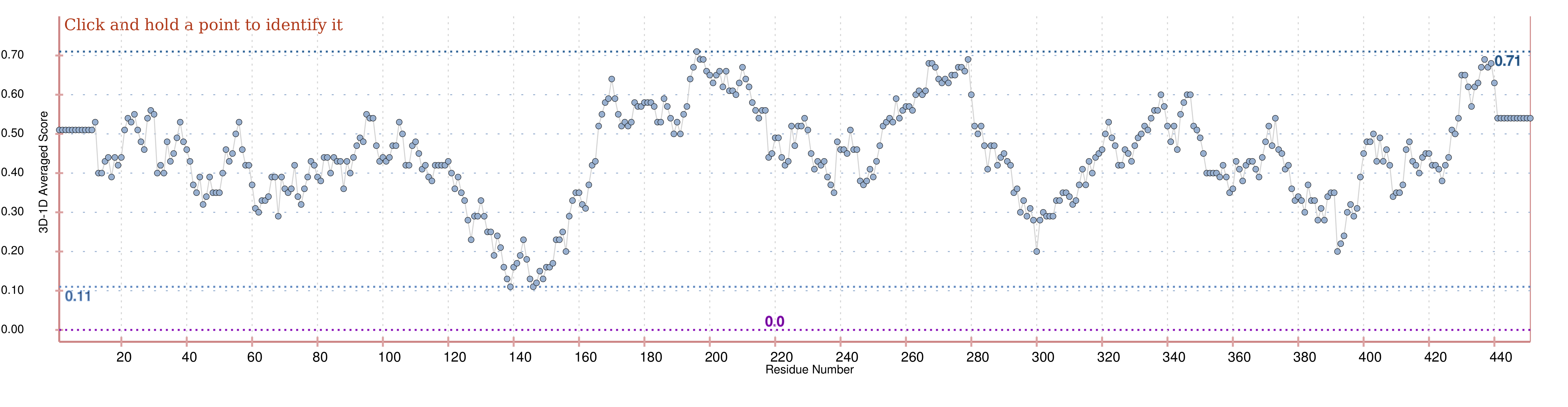

Supplement: S4 Dataset — (ZIP) [file pone.0200607.s004.zip › verify_3d/B. cinerea B05.10 p2 m1.tiff]

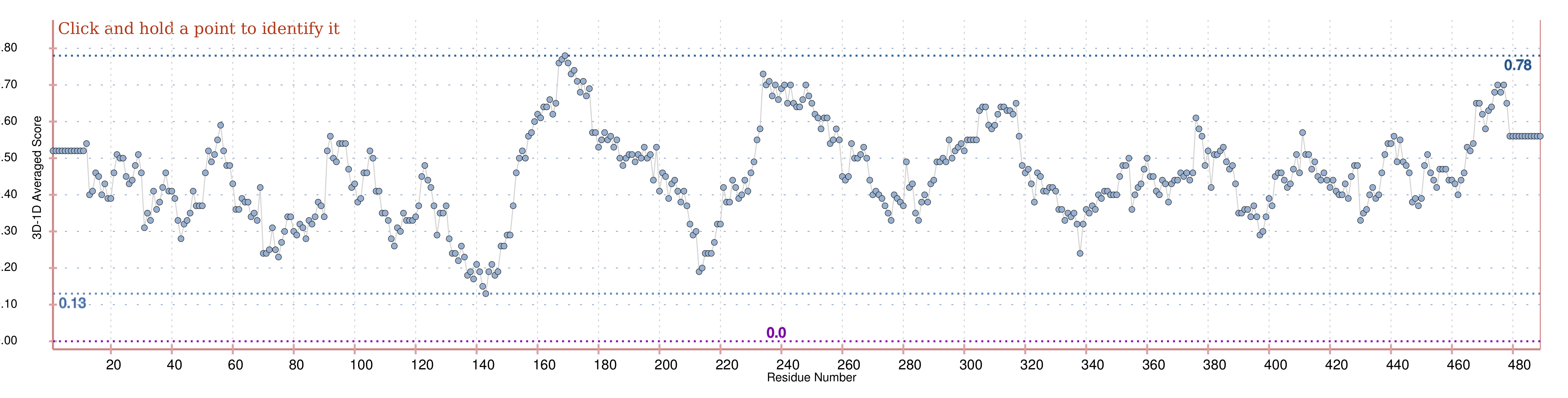

Supplement: S4 Dataset — (ZIP) [file pone.0200607.s004.zip › verify_3d/B. cinerea BcDw1 p1 m1.tiff]

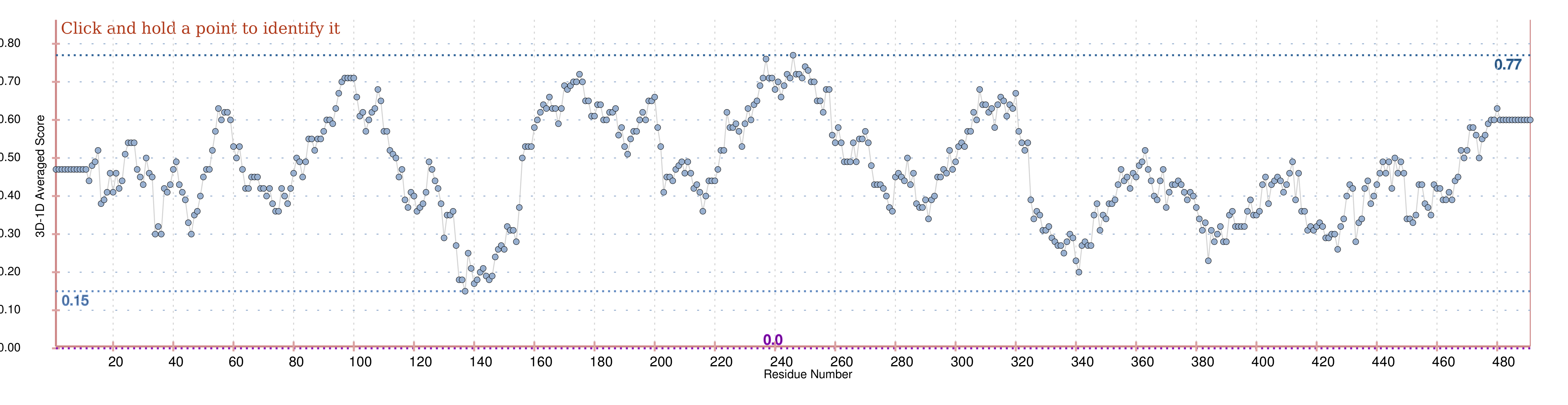

Supplement: S4 Dataset — (ZIP) [file pone.0200607.s004.zip › verify_3d/F oxysporum FOSC 3-a inu p2 m1.tiff]

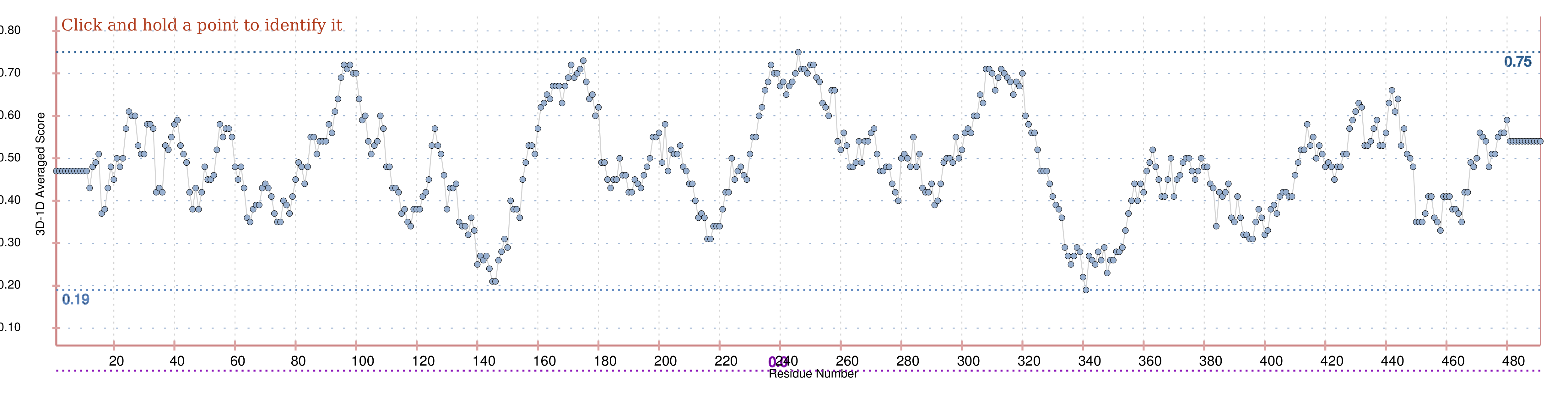

Supplement: S4 Dataset — (ZIP) [file pone.0200607.s004.zip › verify_3d/F oxysporum Fo47 inu p1 m1.tiff]

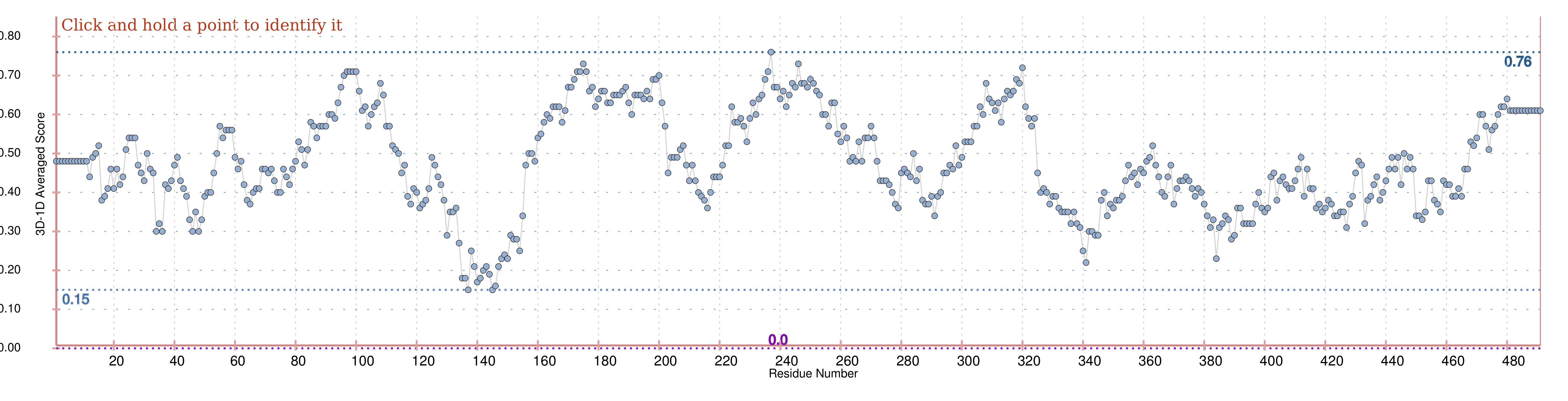

Supplement: S4 Dataset — (ZIP) [file pone.0200607.s004.zip › verify_3d/F oxysporum f. sp. HDV247, f. sp. raphani 54005 inu p3 m1.tiff]

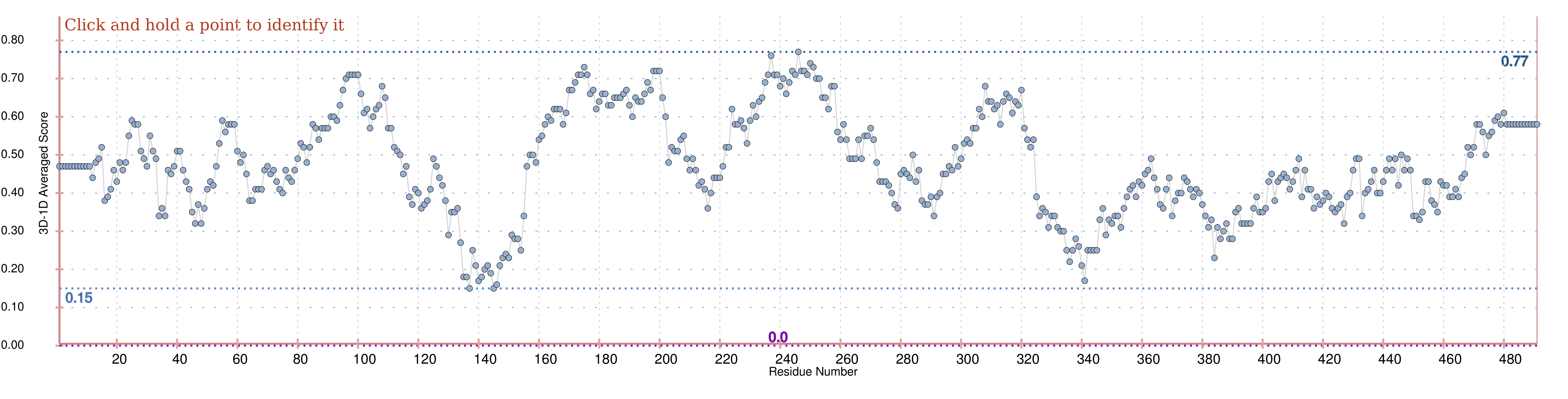

Supplement: S4 Dataset — (ZIP) [file pone.0200607.s004.zip › verify_3d/F oxysporum f. sp. cubense tropical race 4 54006 inu p6 m1.tiff]

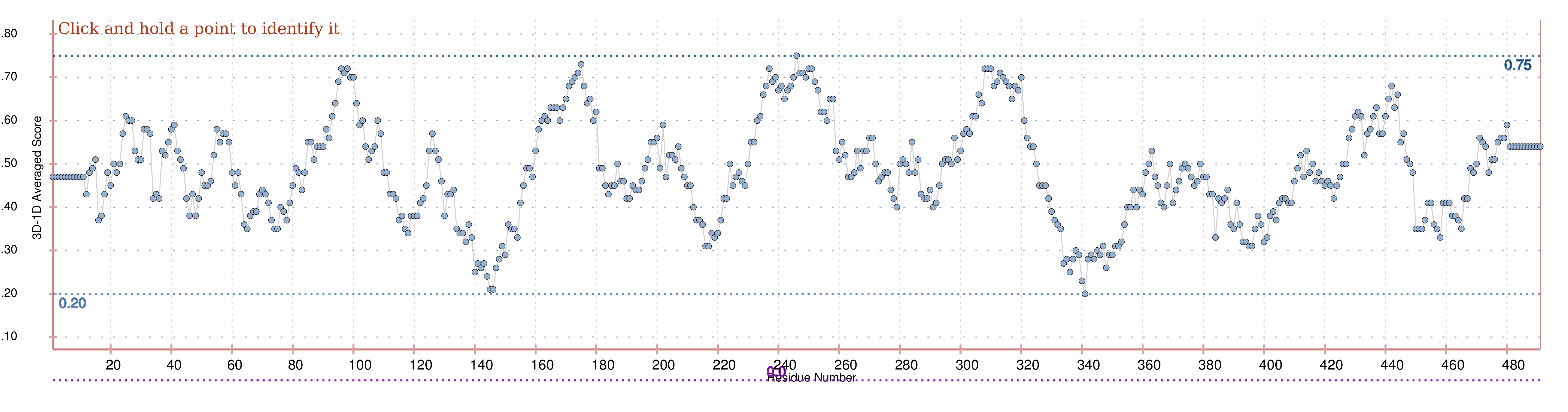

Supplement: S4 Dataset — (ZIP) [file pone.0200607.s004.zip › verify_3d/F oxysporum f. sp. raphani inu p5 m1.tiff]

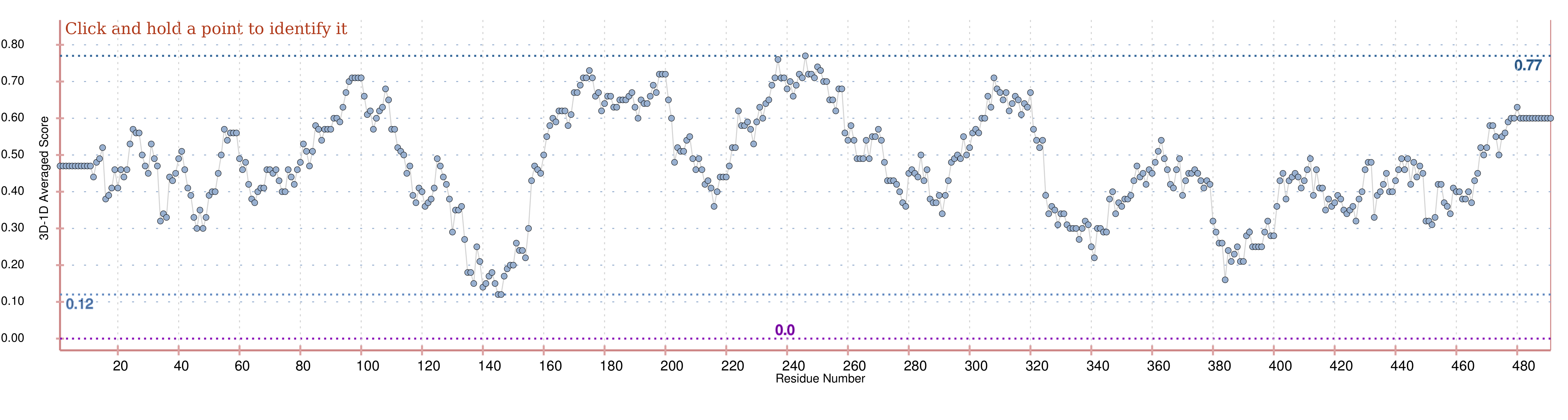

Supplement: S4 Dataset — (ZIP) [file pone.0200607.s004.zip › verify_3d/F oxysporum f. sp. vasinfectum 25433 inu p4 m1.tiff]

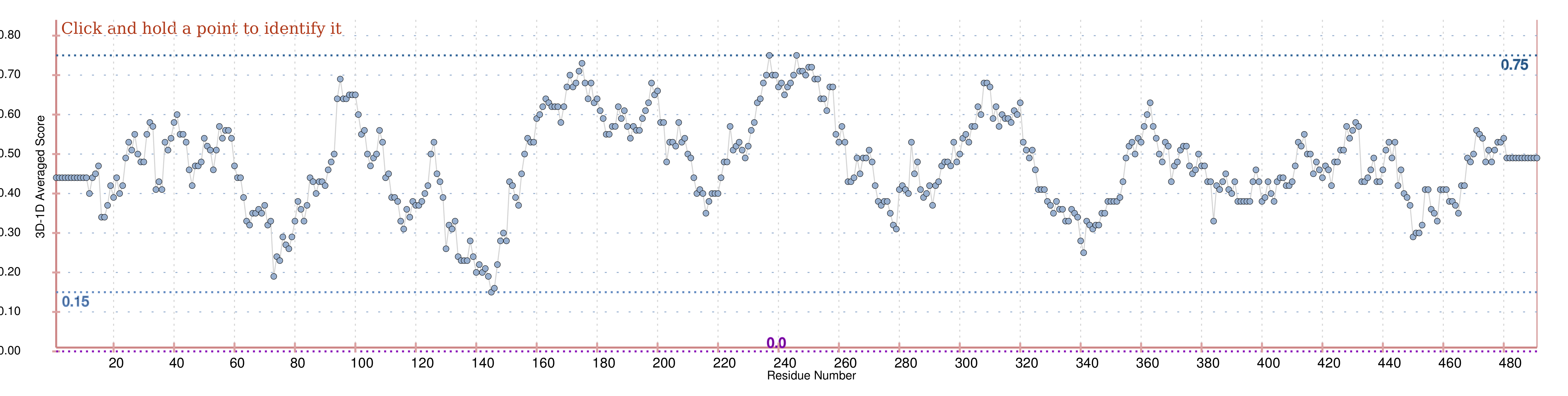

Supplement: S4 Dataset — (ZIP) [file pone.0200607.s004.zip › verify_3d/F oxysporum inu p10 m1.tiff]

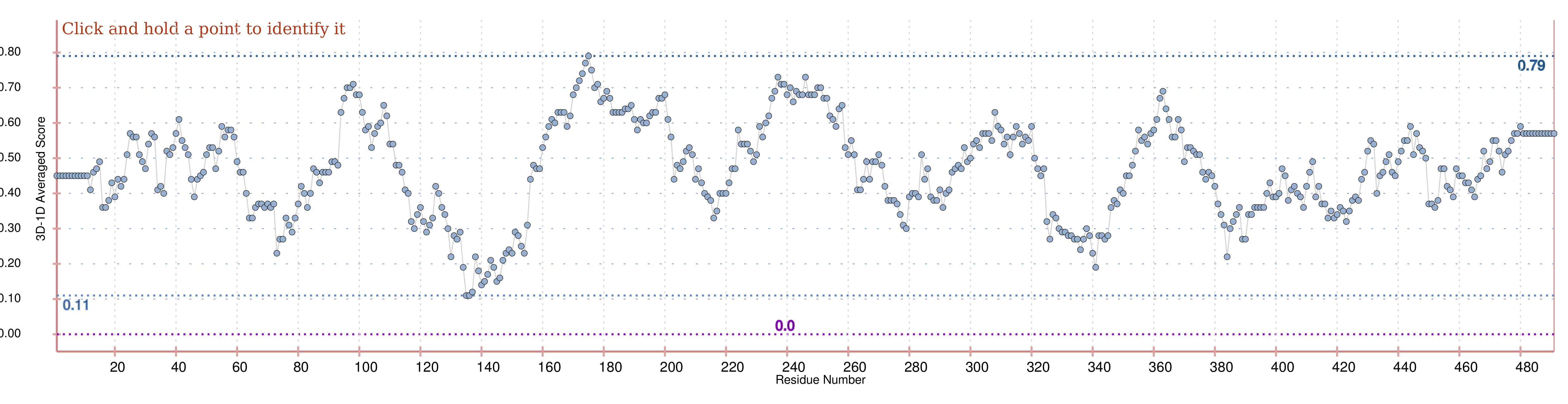

Supplement: S4 Dataset — (ZIP) [file pone.0200607.s004.zip › verify_3d/F oxysporum inu p11 m1.tiff]

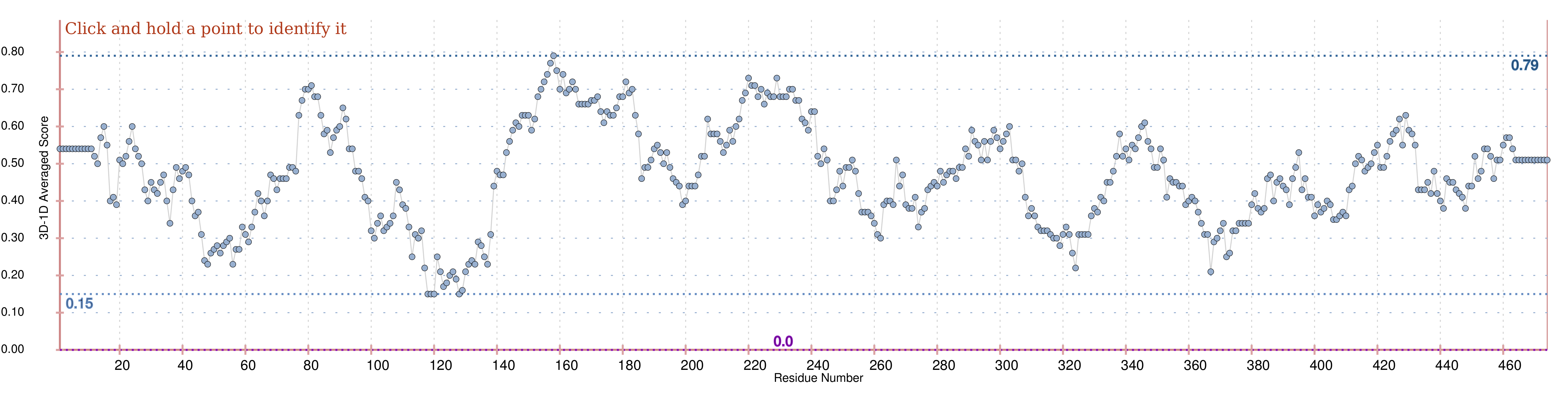

Supplement: S4 Dataset — (ZIP) [file pone.0200607.s004.zip › verify_3d/F oxysporum inu p12 m1.tiff]

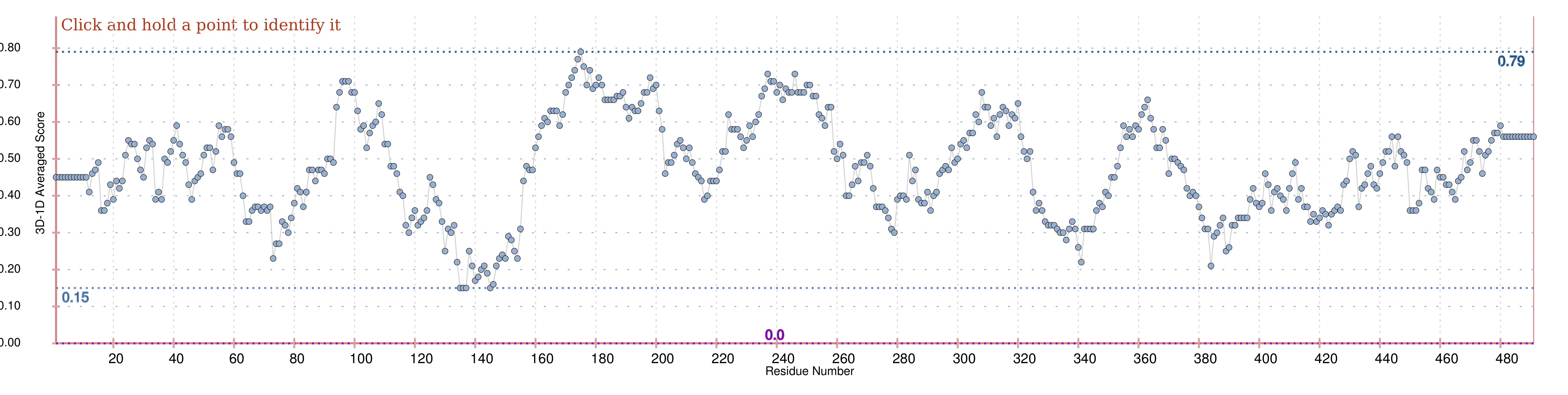

Supplement: S4 Dataset — (ZIP) [file pone.0200607.s004.zip › verify_3d/F oxysporum inu p13 m1.tiff]

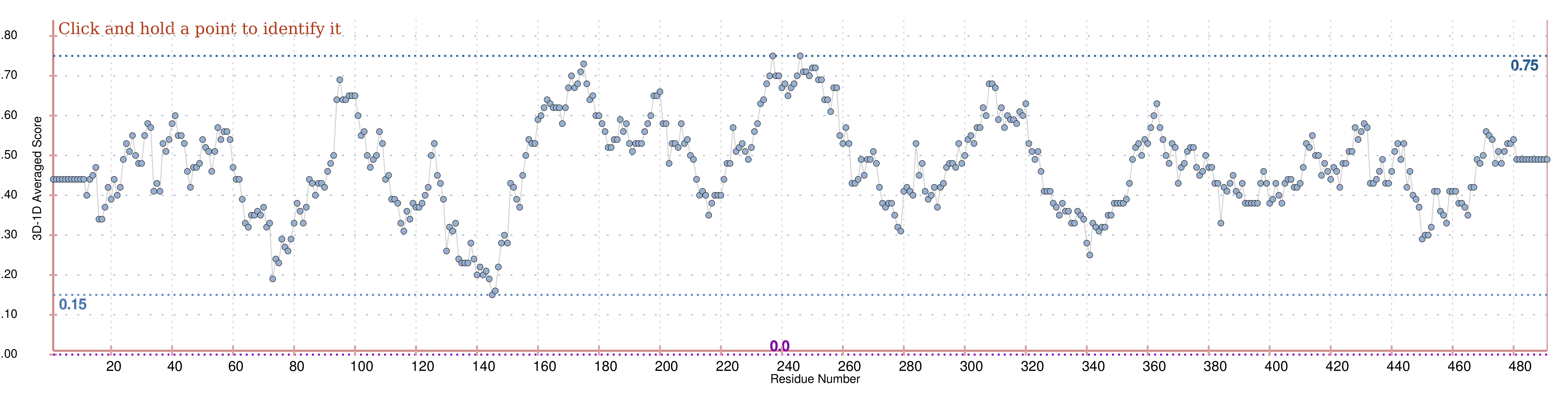

Supplement: S4 Dataset — (ZIP) [file pone.0200607.s004.zip › verify_3d/F oxysporum inu p14 m1.tiff]

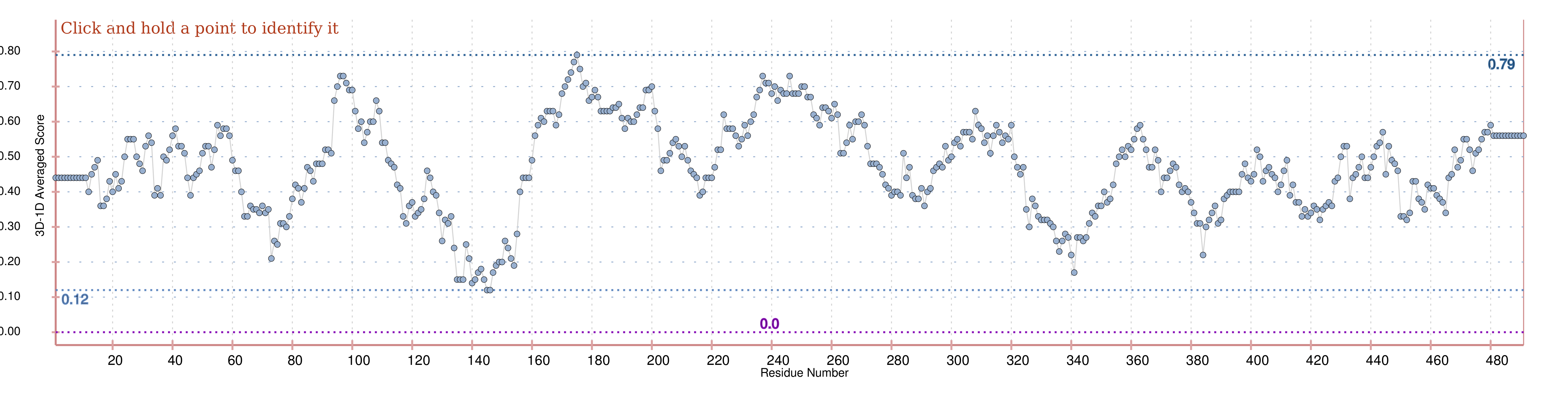

Supplement: S4 Dataset — (ZIP) [file pone.0200607.s004.zip › verify_3d/F oxysporum inu p8 m1.tiff]

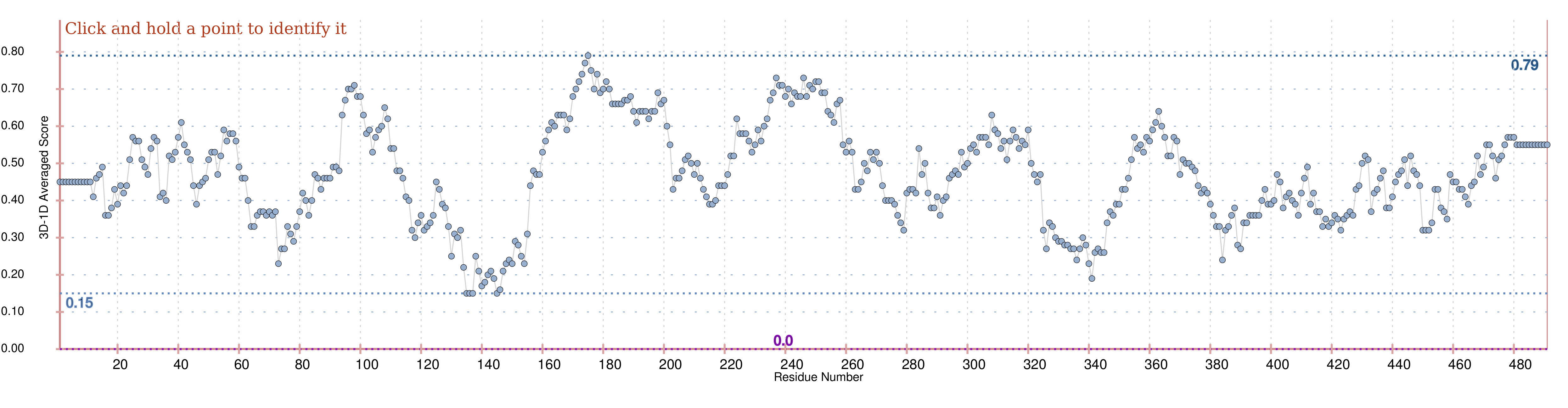

Supplement: S4 Dataset — (ZIP) [file pone.0200607.s004.zip › verify_3d/F oxysporum inu p9 m1.tiff]

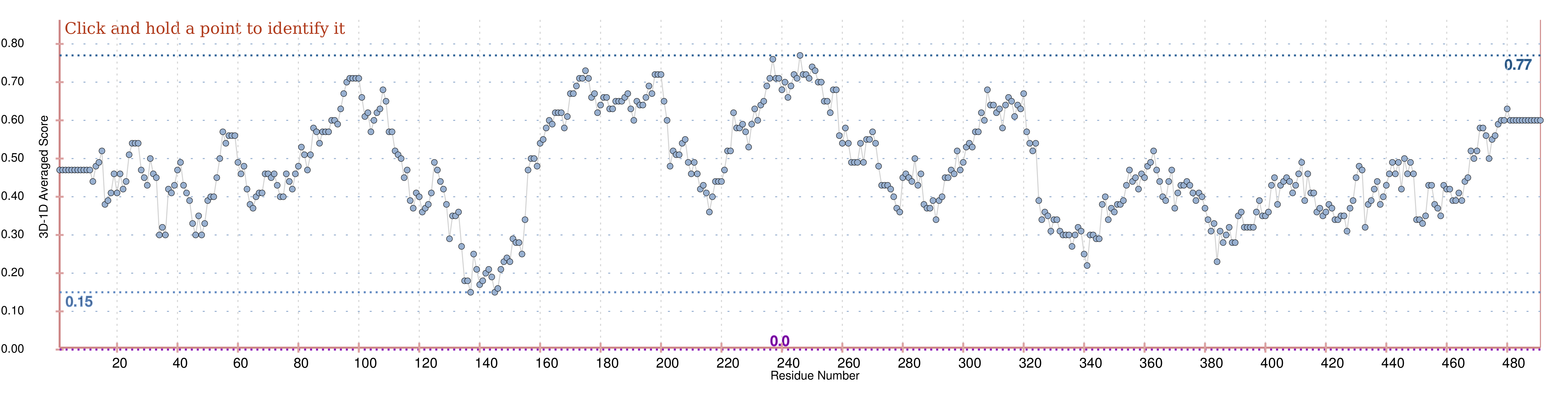

Supplement: S4 Dataset — (ZIP) [file pone.0200607.s004.zip › verify_3d/Fusarium oxysporum Fo5176 Hypo p1 m1.tiff]

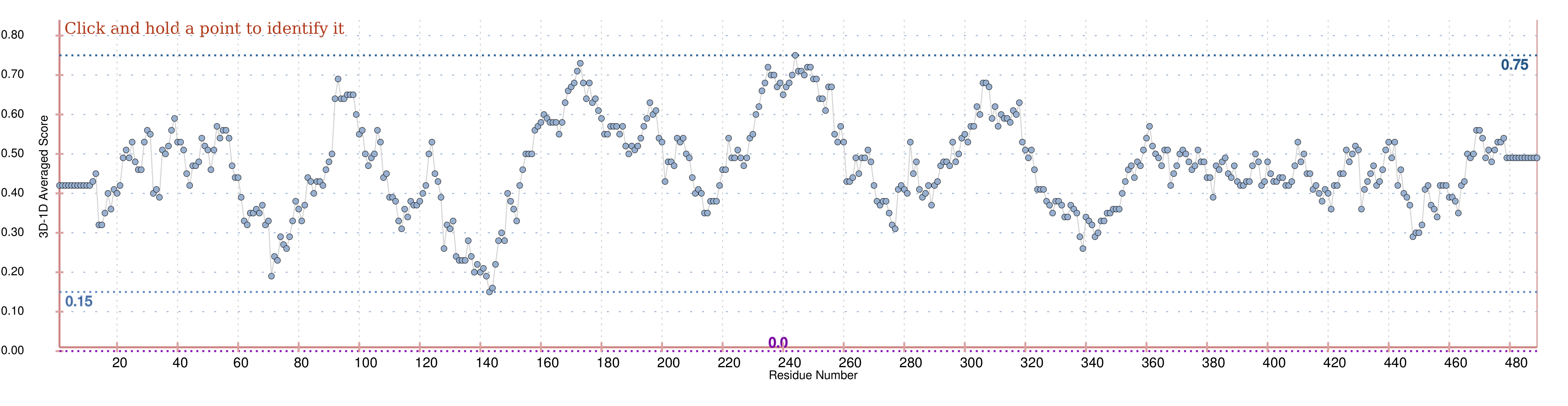

Supplement: S4 Dataset — (ZIP) [file pone.0200607.s004.zip › verify_3d/Fusarium oxysporum Fo5176 Hypo p7 m1.tiff]

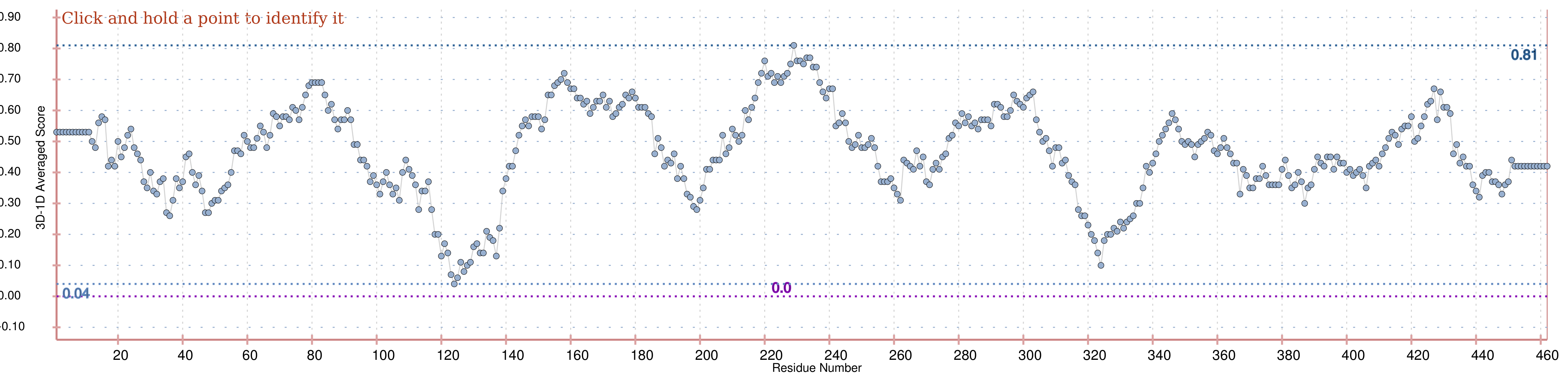

Supplement: S4 Dataset — (ZIP) [file pone.0200607.s004.zip › verify_3d/M. phaseolina MS6 p1 m1.tiff]

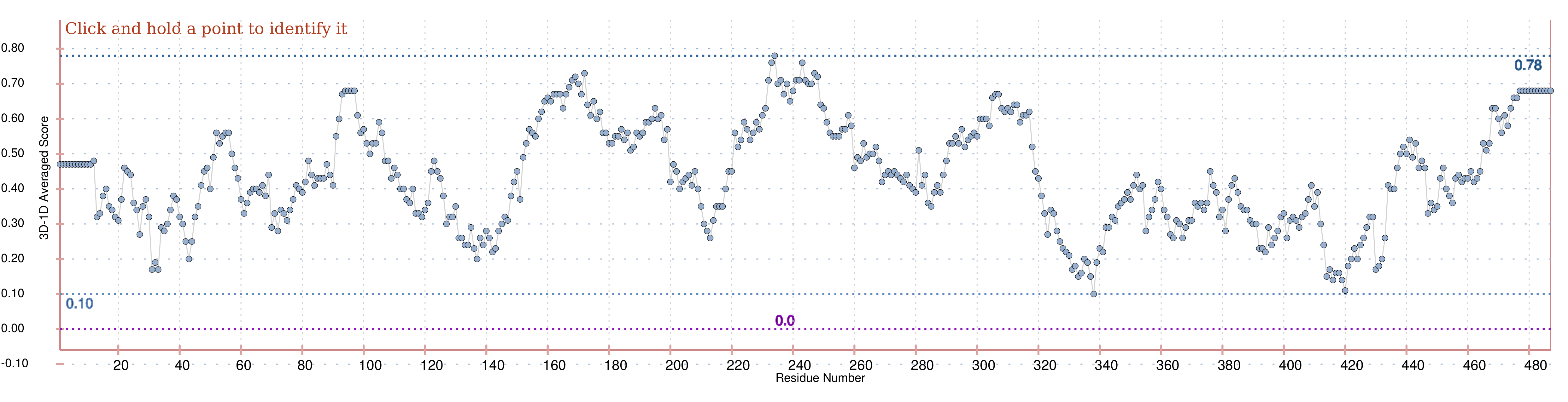

Supplement: S4 Dataset — (ZIP) [file pone.0200607.s004.zip › verify_3d/Odiodendron maius Zn p1 m2.tiff]

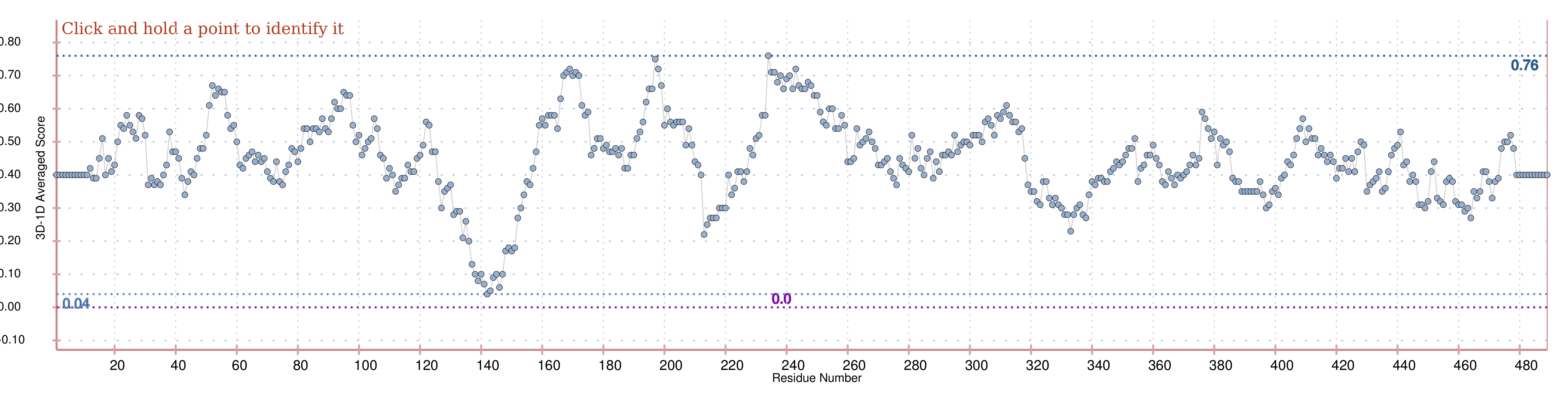

Supplement: S4 Dataset — (ZIP) [file pone.0200607.s004.zip › verify_3d/P. brasilianum p1 m2.tiff]

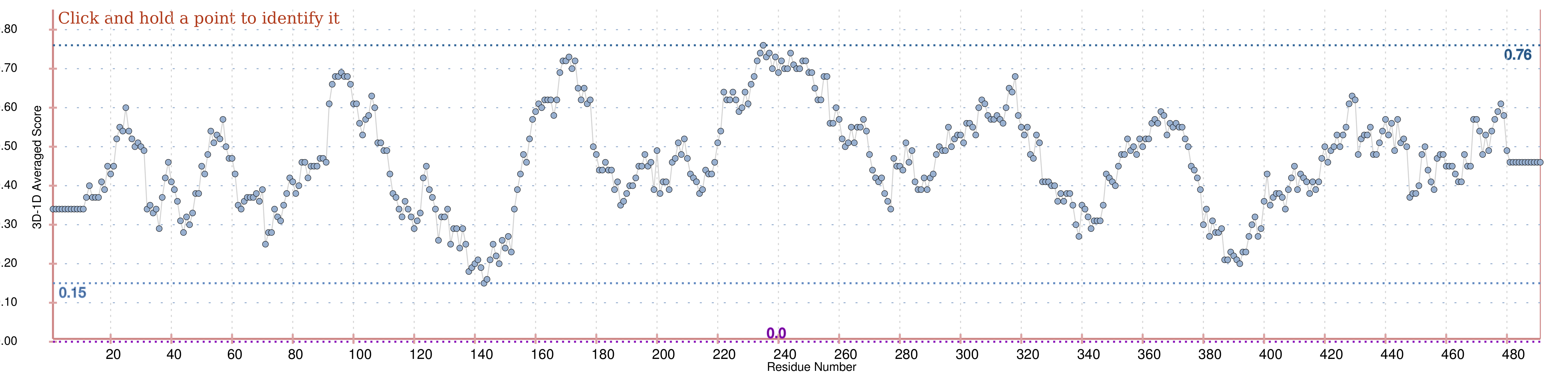

Supplement: S4 Dataset — (ZIP) [file pone.0200607.s004.zip › verify_3d/P. nordicum p1 m1.tiff]

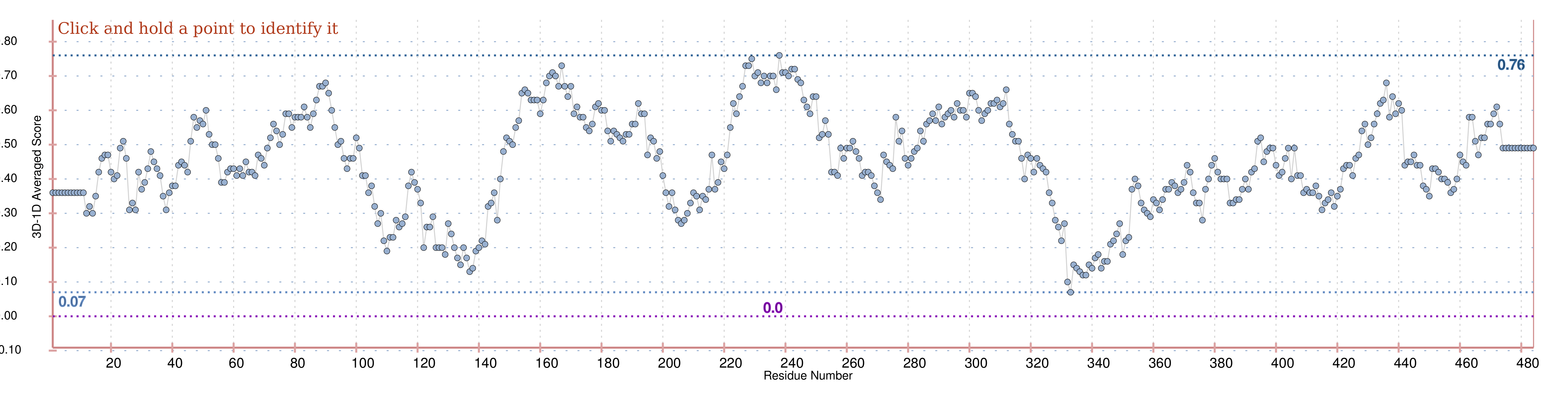

Supplement: S4 Dataset — (ZIP) [file pone.0200607.s004.zip › verify_3d/Pseudogymnoascus sp. VKMF-3775 p1 m2.tiff]

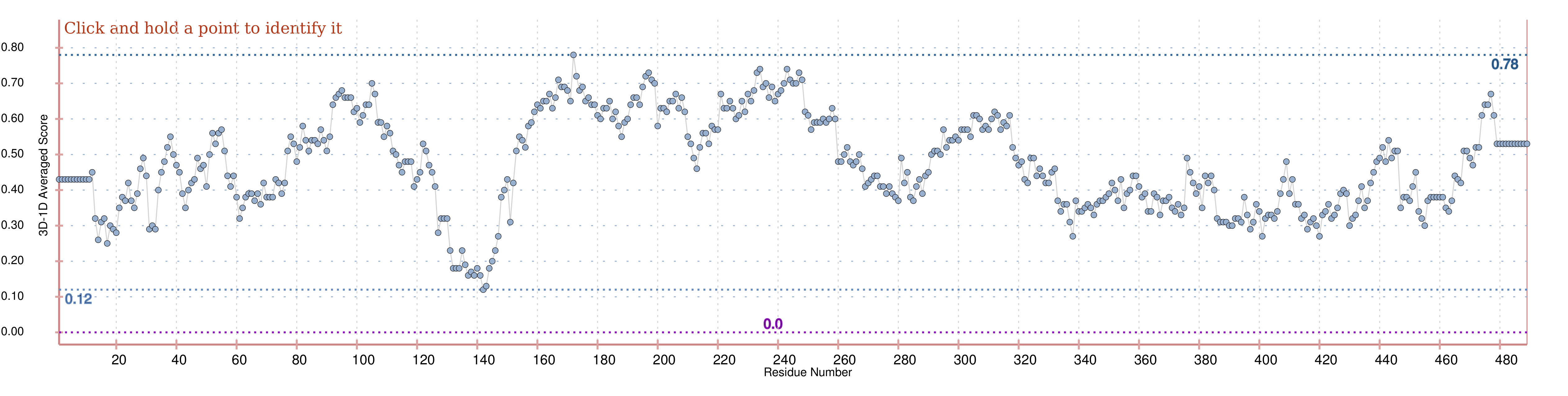

Supplement: S4 Dataset — (ZIP) [file pone.0200607.s004.zip › verify_3d/Pseudogymnoascus sp. VKMF-3775 p3 m2.tiff]

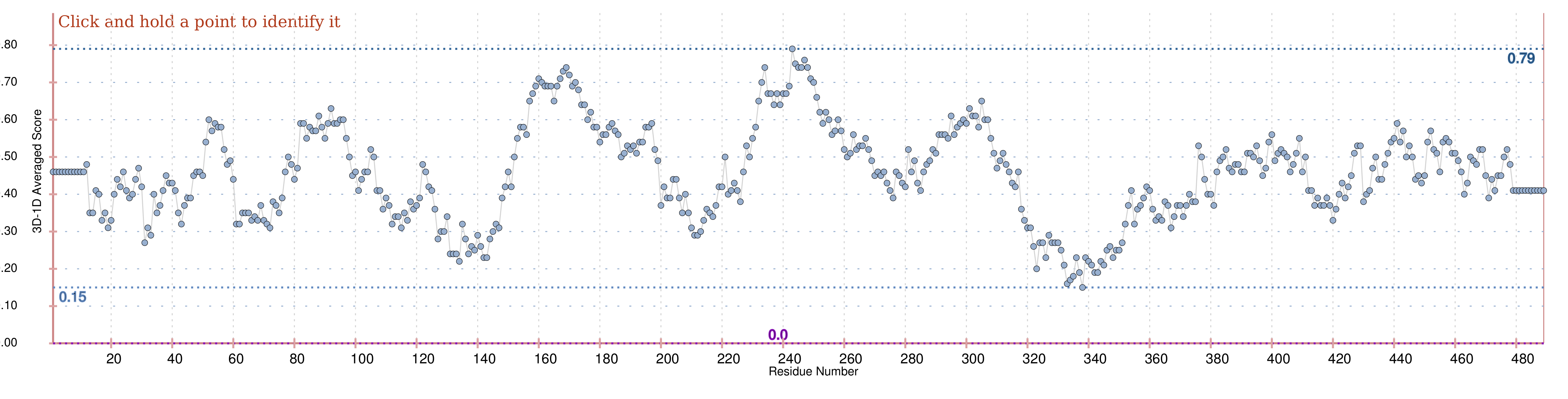

Supplement: S4 Dataset — (ZIP) [file pone.0200607.s004.zip › verify_3d/Pseudogymnoascus sp. VKMF-4515 (FW-2607) p2 m1.tiff]

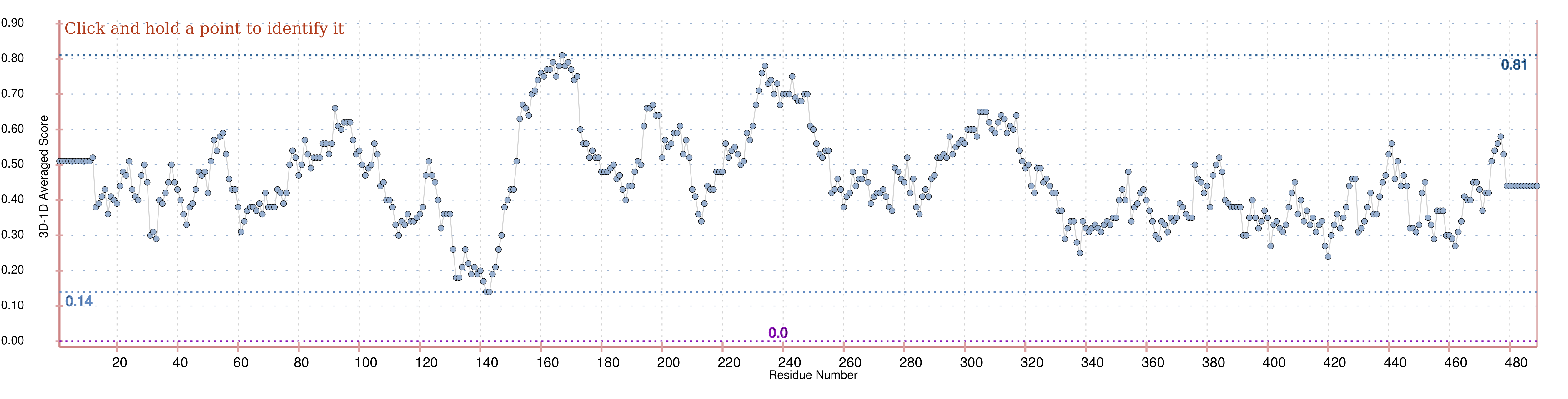

Supplement: S4 Dataset — (ZIP) [file pone.0200607.s004.zip › verify_3d/Pseudogymnoascus sp. WSF 3629 p4 m2.tiff]

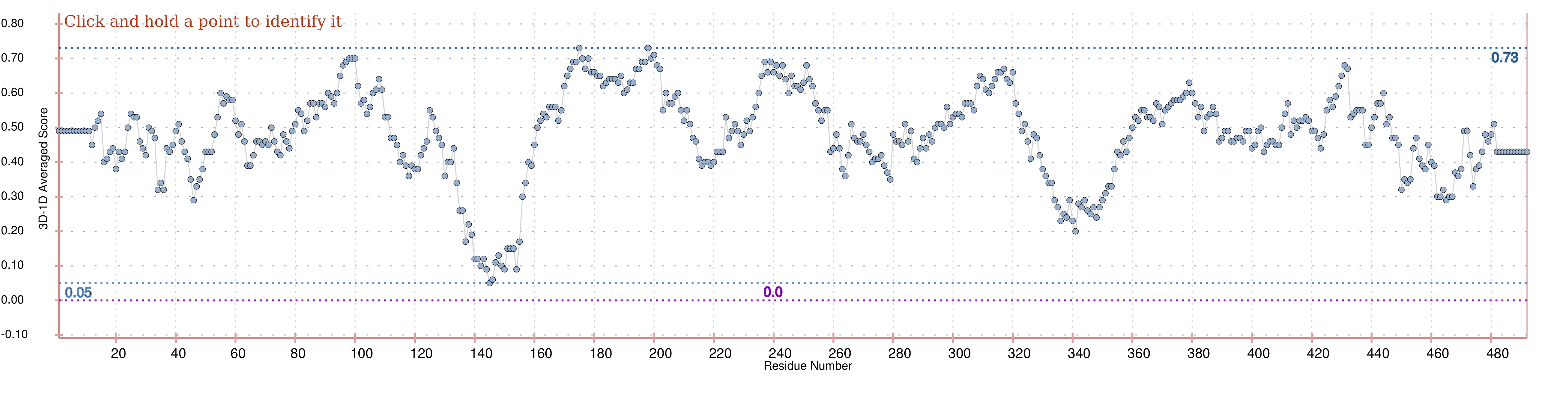

Supplement: S4 Dataset — (ZIP) [file pone.0200607.s004.zip › verify_3d/Pyrenochaeta sp. DS3sAY3a p1 m1.tiff]
