# Supplementary figures and images for: In Silico evaluation and identification of fungi capable of producing endo-inulinase enzyme (part 3 of 4)
Source: PLoS One. 2018 Jul 12;13(7):e0200607. doi: 10.1371/journal.pone.0200607 (PMC6042768; doi:10.1371/journal.pone.0200607)

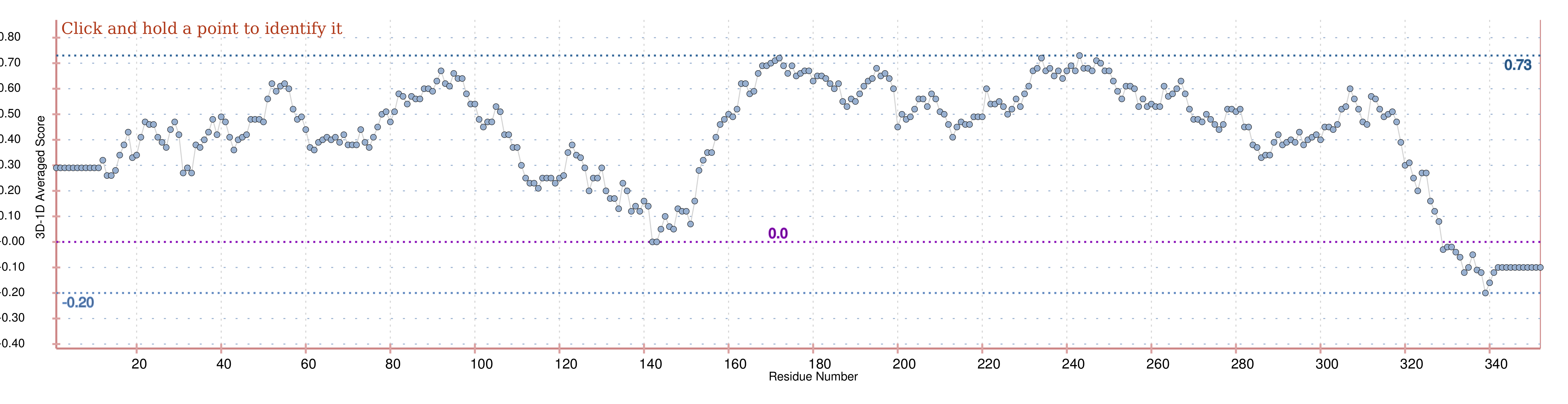

Supplement: S4 Dataset — (ZIP) [file pone.0200607.s004.zip › verify_3d/Stachybotrys chartarum IBT 40288 p18 m2.tiff]

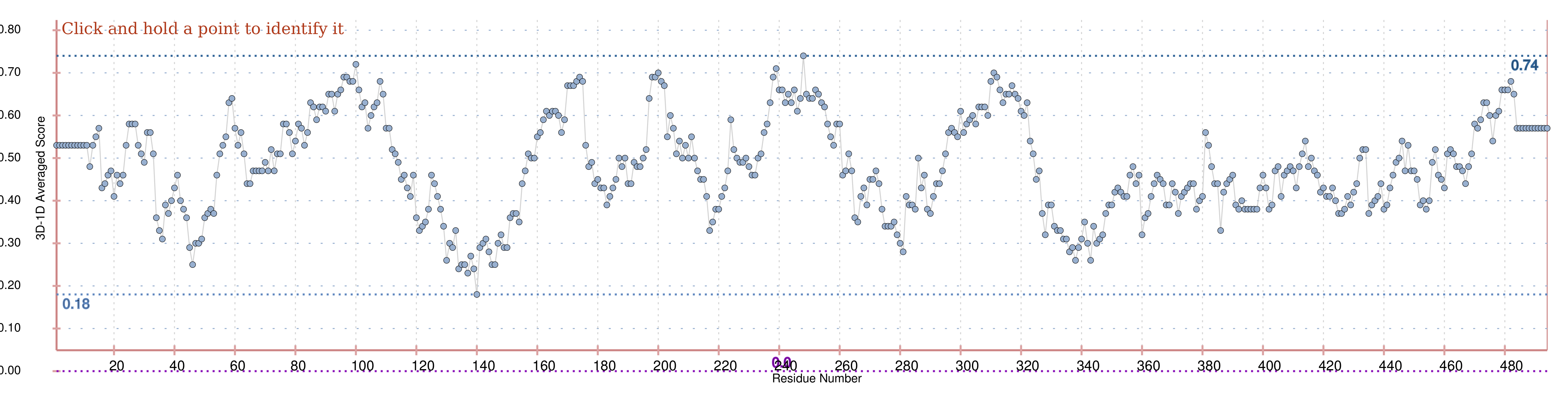

Supplement: S4 Dataset — (ZIP) [file pone.0200607.s004.zip › verify_3d/Stachybotrys chartarum IBT 40288 p2 m1.tiff]

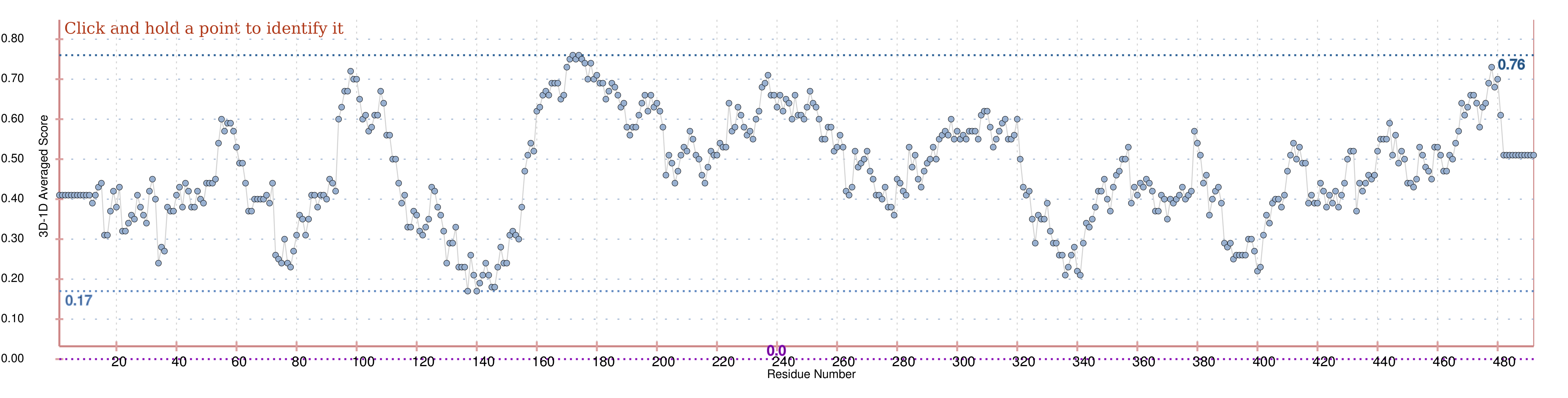

Supplement: S4 Dataset — (ZIP) [file pone.0200607.s004.zip › verify_3d/Stachybotrys chartarum IBT 40288 p6 m2.tiff]

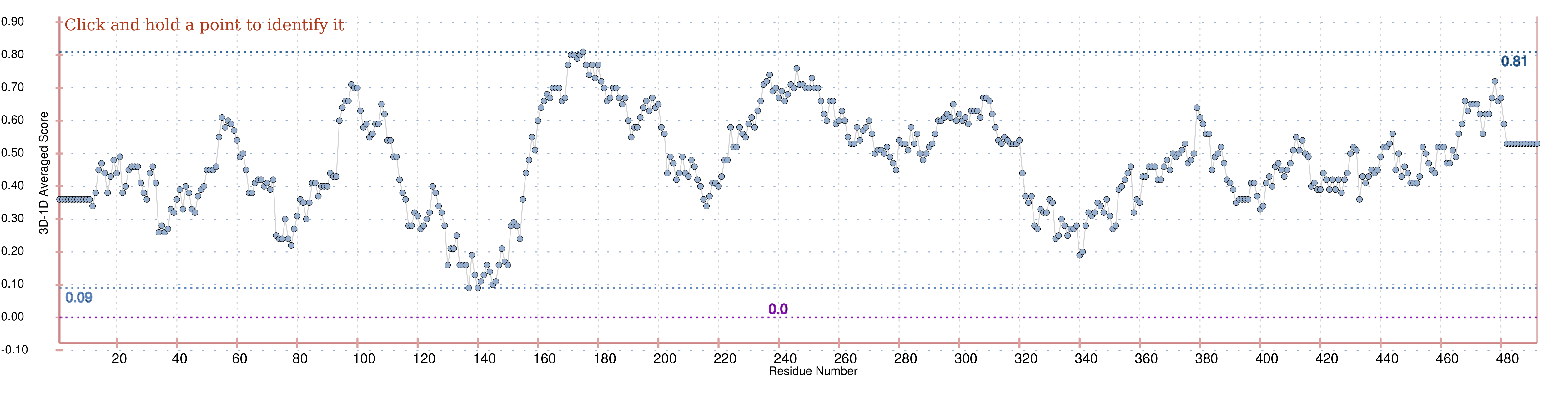

Supplement: S4 Dataset — (ZIP) [file pone.0200607.s004.zip › verify_3d/Stachybotrys chartarum IBT 40293 p 4m 2.tiff]

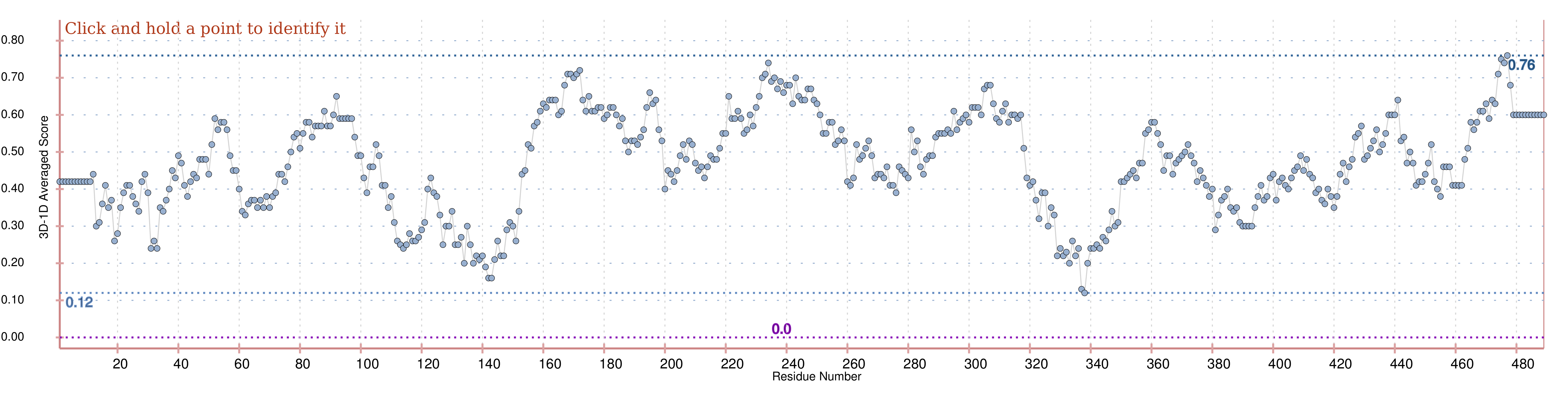

Supplement: S4 Dataset — (ZIP) [file pone.0200607.s004.zip › verify_3d/Stachybotrys chartarum IBT 40293 p3m2.tiff]

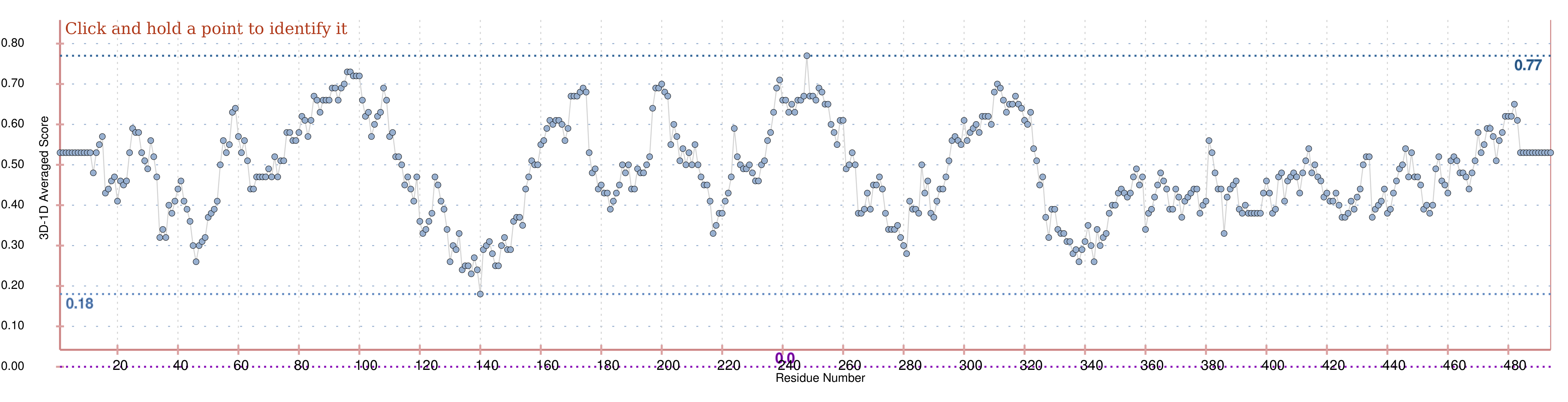

Supplement: S4 Dataset — (ZIP) [file pone.0200607.s004.zip › verify_3d/Stachybotrys chartarum IBT 7711 p1 m1.tiff]

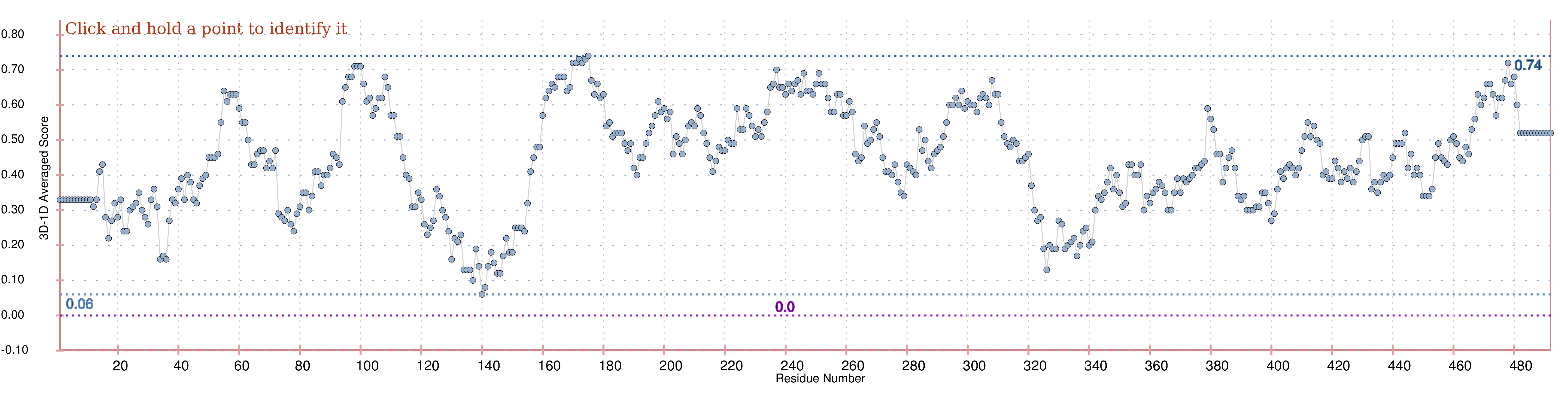

Supplement: S4 Dataset — (ZIP) [file pone.0200607.s004.zip › verify_3d/Stachybotrys chartarum IBT 7711 p5 m2.tiff]

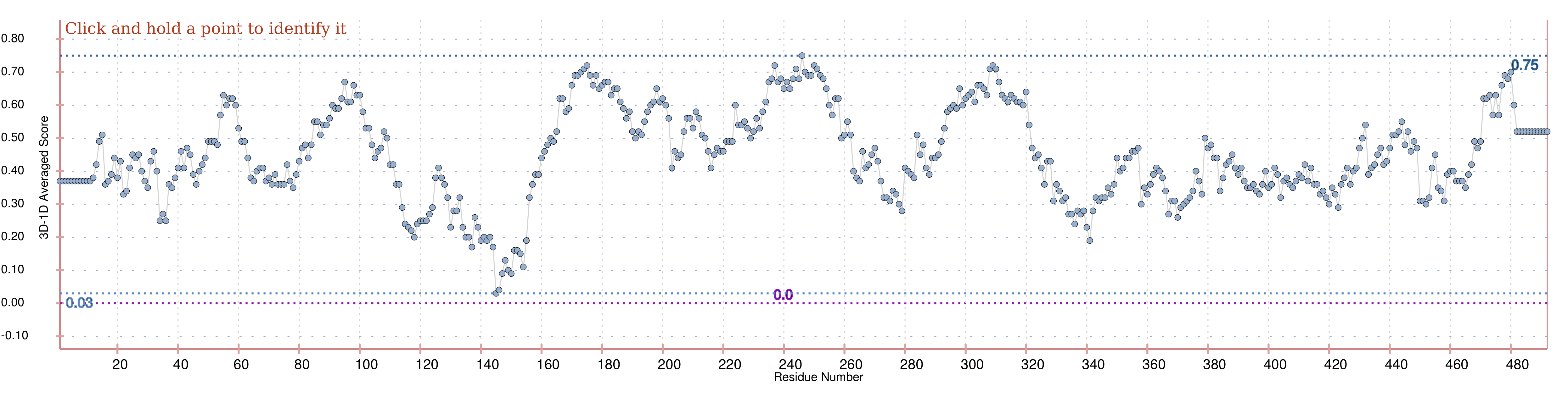

Supplement: S4 Dataset — (ZIP) [file pone.0200607.s004.zip › verify_3d/Stachybotrys chartarum IBT 7711 p7 m1.tiff]

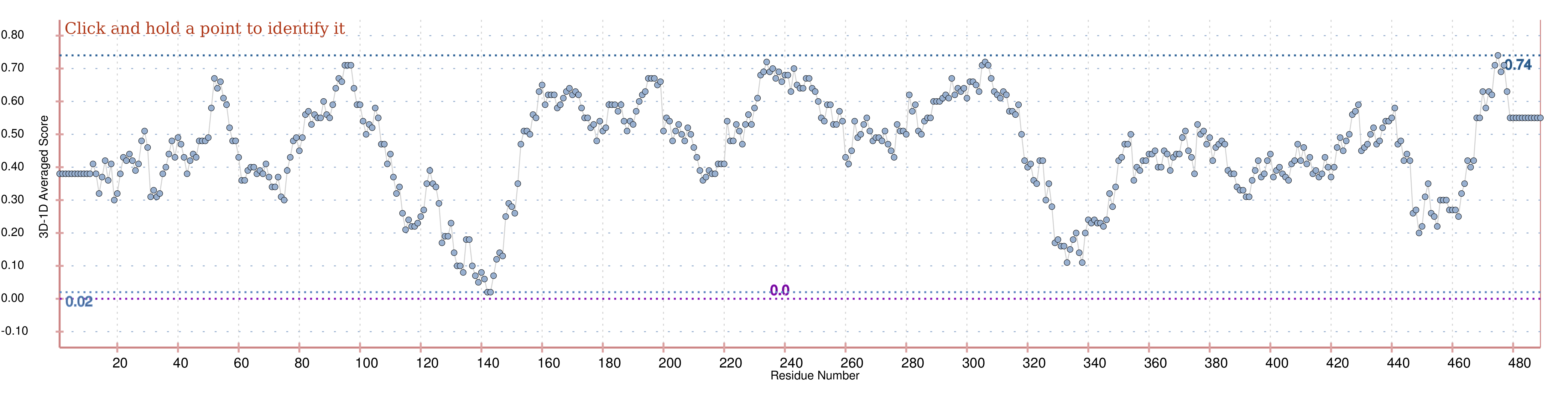

Supplement: S4 Dataset — (ZIP) [file pone.0200607.s004.zip › verify_3d/Stachybotrys chlorohalonata IBT 40285 p1 m1.tiff]

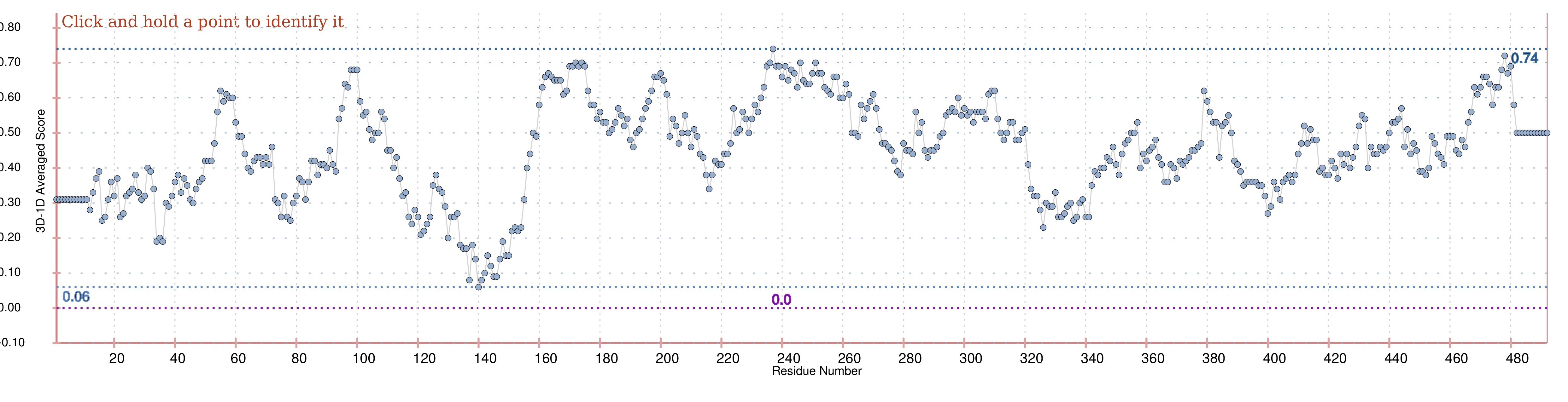

Supplement: S4 Dataset — (ZIP) [file pone.0200607.s004.zip › verify_3d/Stachybotrys chlorohalonata IBT 40285 p2 m2.tiff]

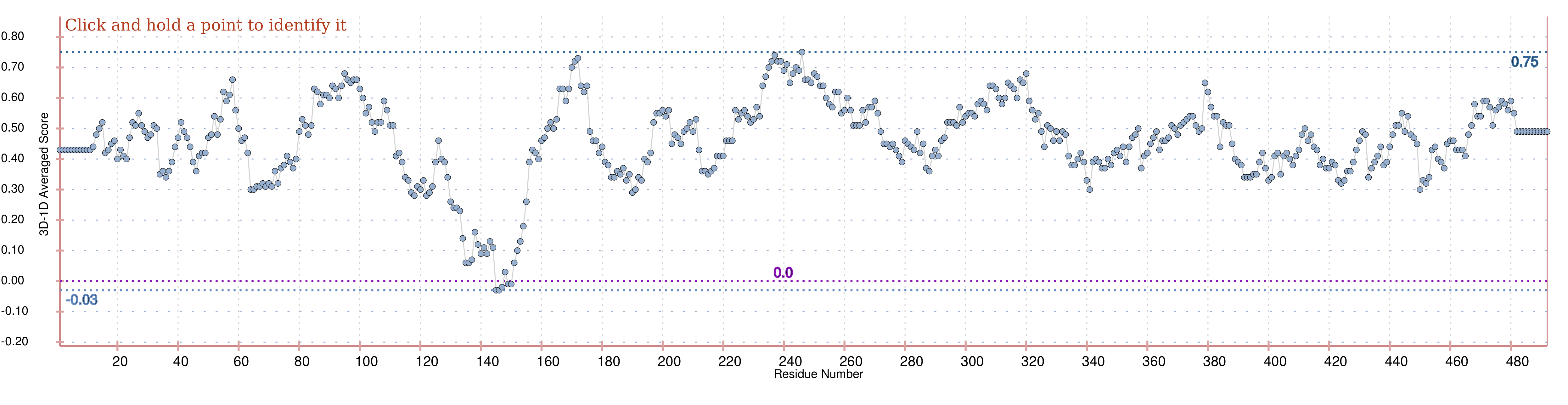

Supplement: S4 Dataset — (ZIP) [file pone.0200607.s004.zip › verify_3d/T cellulolyticus inu p2 m1.tiff]

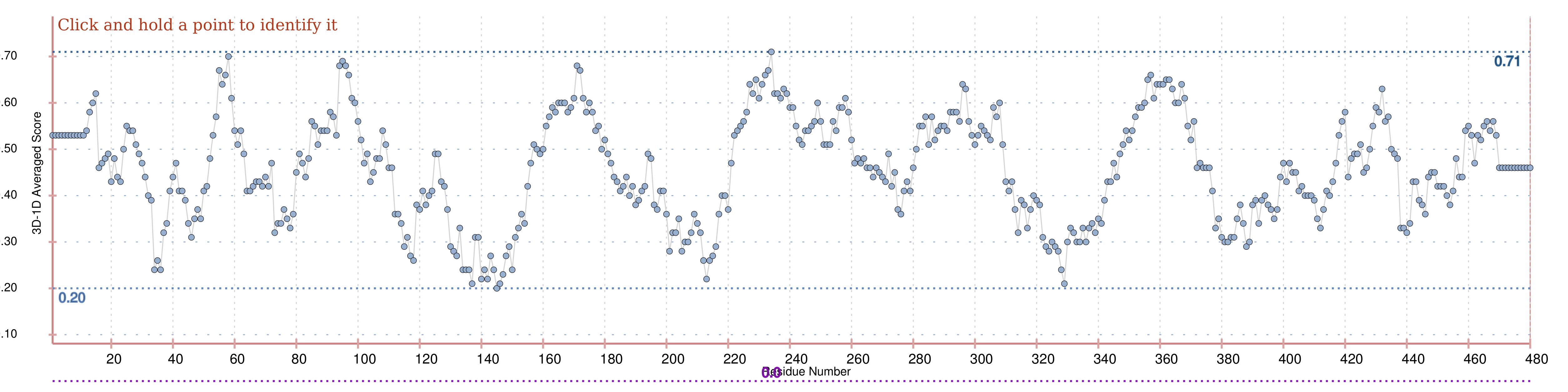

Supplement: S4 Dataset — (ZIP) [file pone.0200607.s004.zip › verify_3d/T stipitatus p1 m1.tiff]

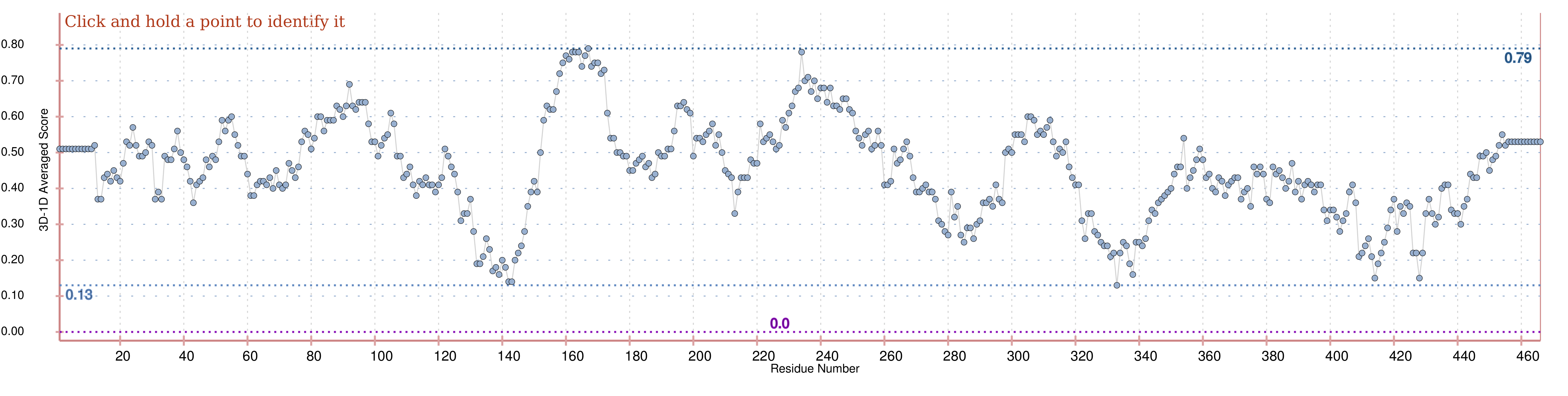

Supplement: S4 Dataset — (ZIP) [file pone.0200607.s004.zip › verify_3d/T. cellulolyticus p1 m2.tiff]

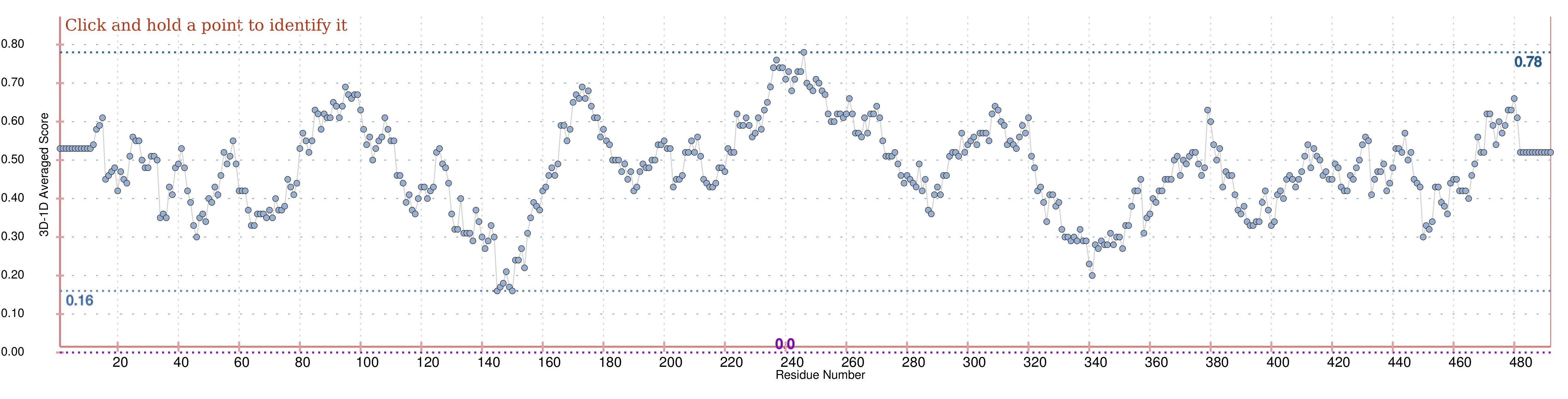

Supplement: S4 Dataset — (ZIP) [file pone.0200607.s004.zip › verify_3d/T. islandicus p1 m1.tiff]

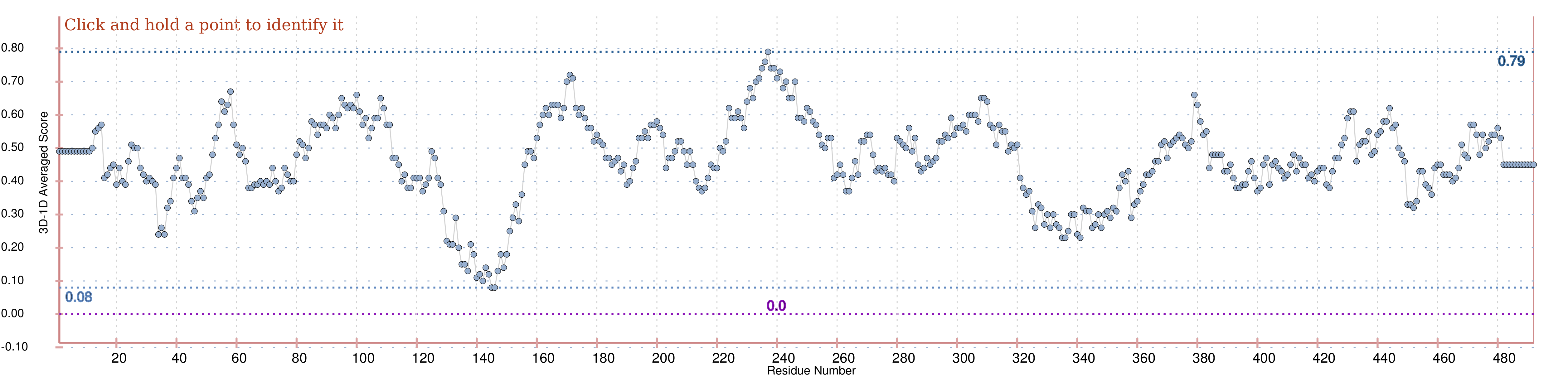

Supplement: S4 Dataset — (ZIP) [file pone.0200607.s004.zip › verify_3d/T. purpureogenus p1m1.tiff]

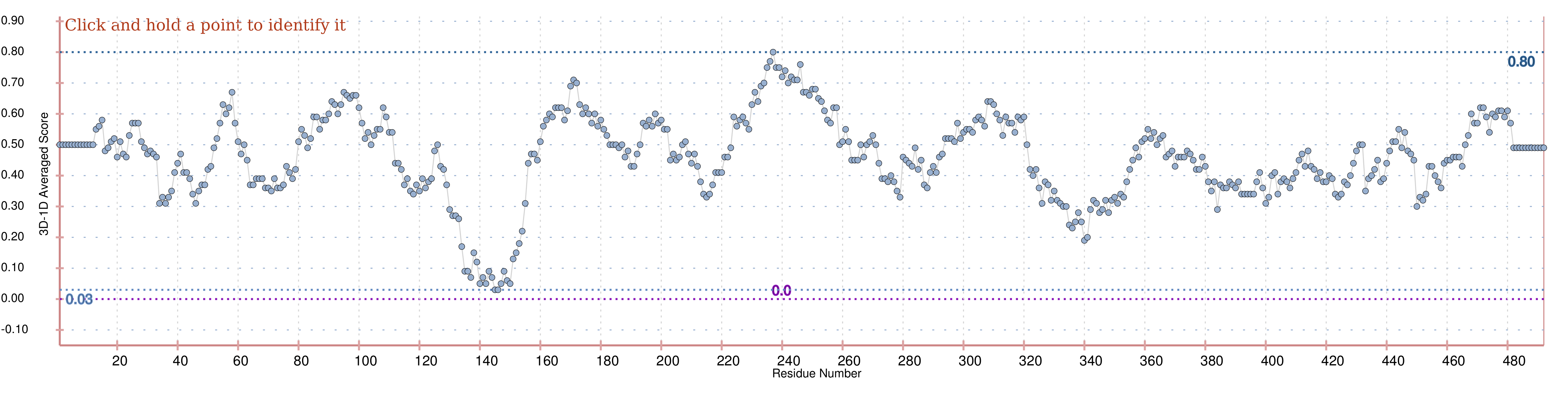

Supplement: S4 Dataset — (ZIP) [file pone.0200607.s004.zip › verify_3d/T. verruculosus p1 m1.tiff]

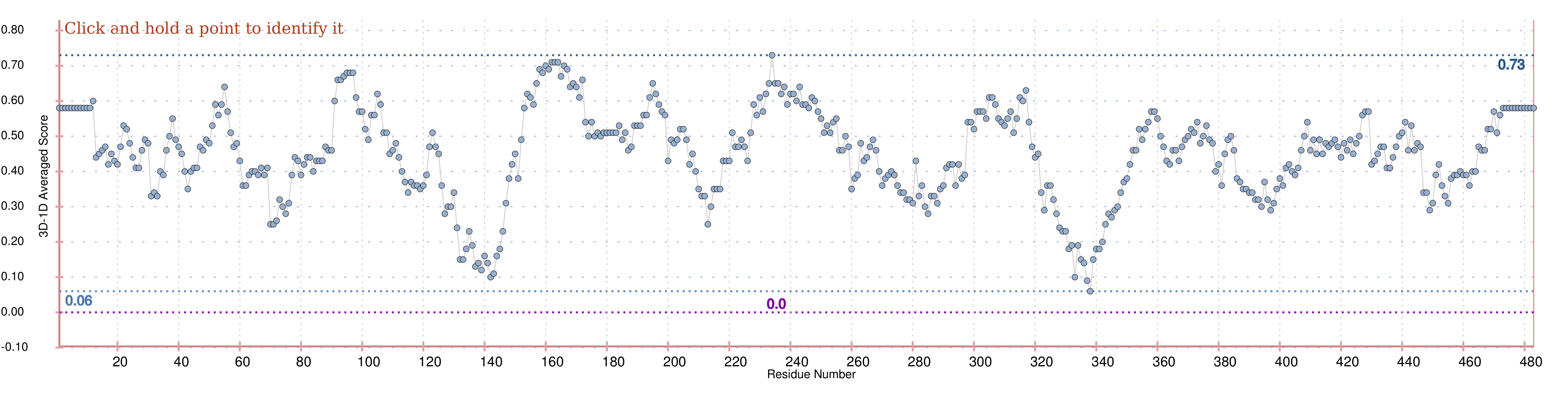

Supplement: S4 Dataset — (ZIP) [file pone.0200607.s004.zip › verify_3d/T. verruculosus p2 m2.tiff]

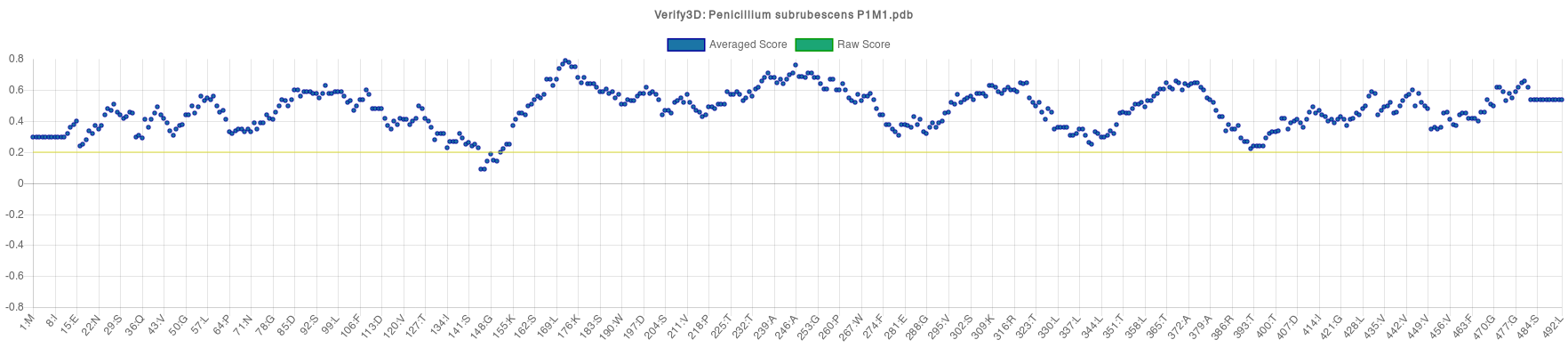

Supplement: S4 Dataset — (ZIP) [file pone.0200607.s004.zip › verify_3d/verify3d_P_subrubescens_p1m1.tiff]

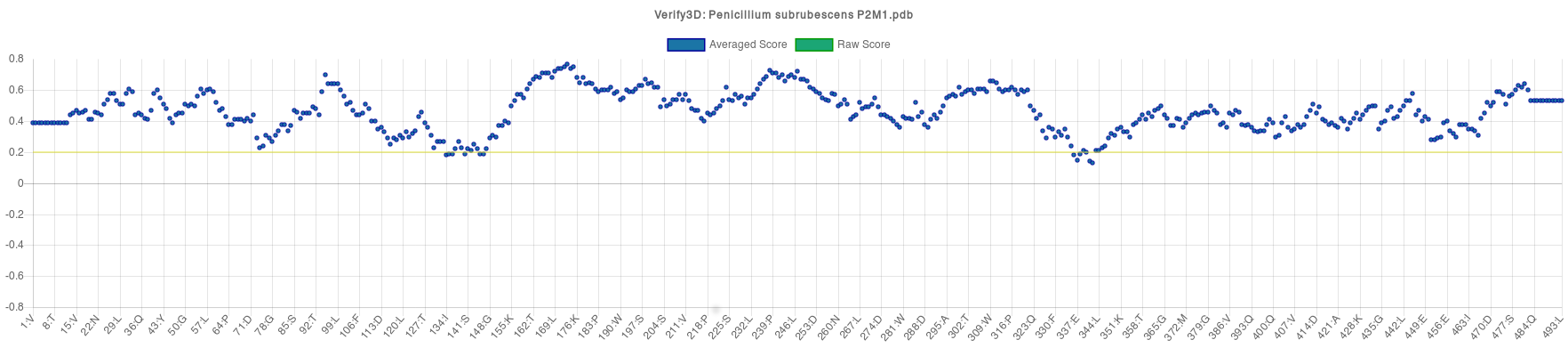

Supplement: S4 Dataset — (ZIP) [file pone.0200607.s004.zip › verify_3d/verify3d_P_subrubescens_p2m1.tiff]

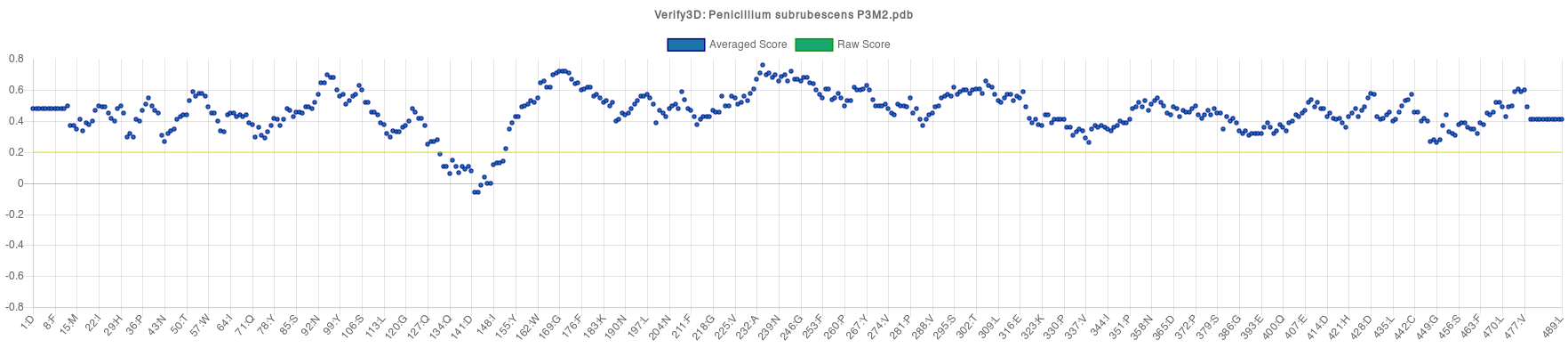

Supplement: S4 Dataset — (ZIP) [file pone.0200607.s004.zip › verify_3d/verify3d_P_subrubescens_p3m2.tiff]

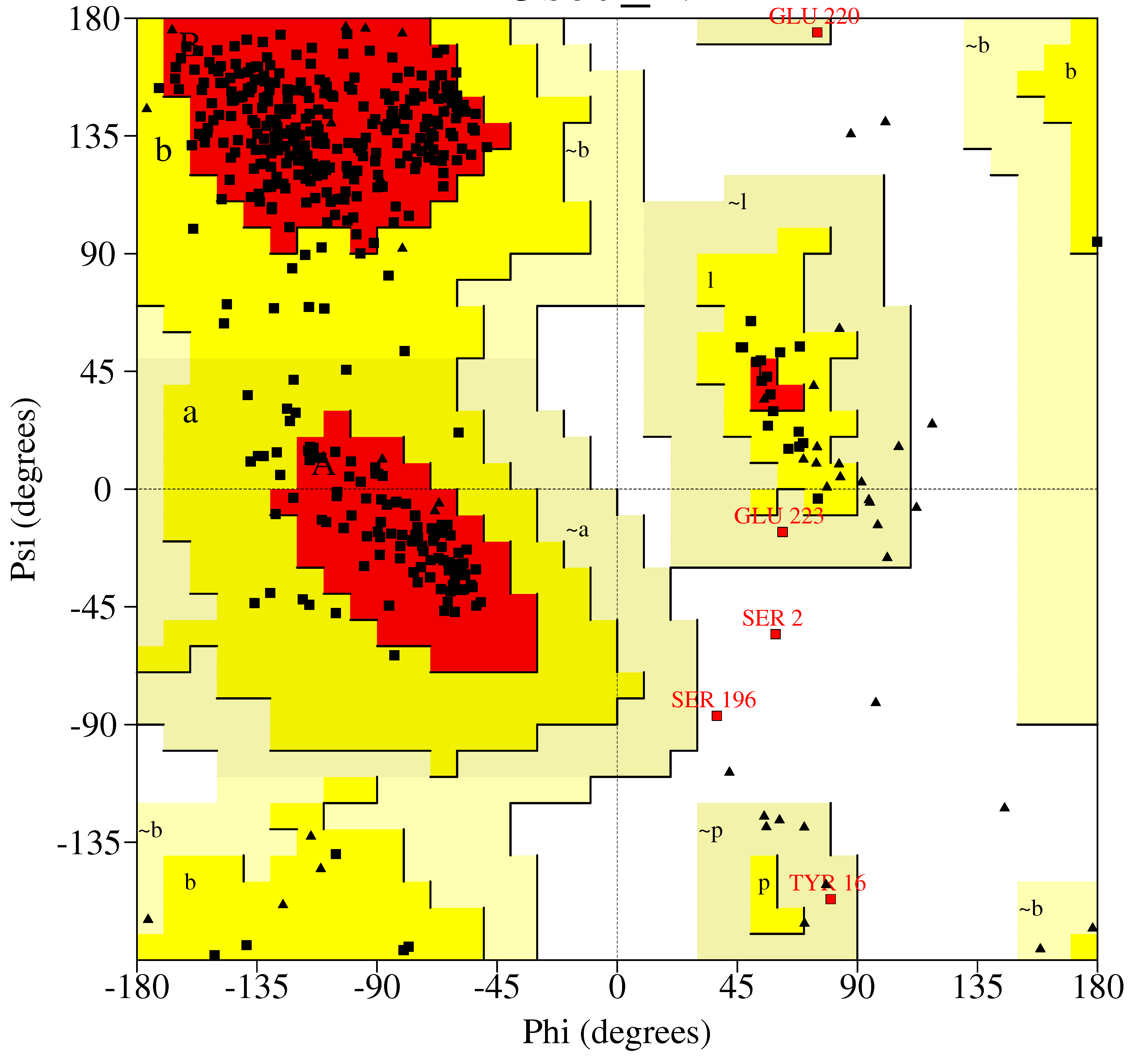

Supplement: S5 Dataset — The plots were generated through PROCHECK analysis. (ZIP) [file pone.0200607.s005.zip › Ramachandranplots/3SC7.tiff]

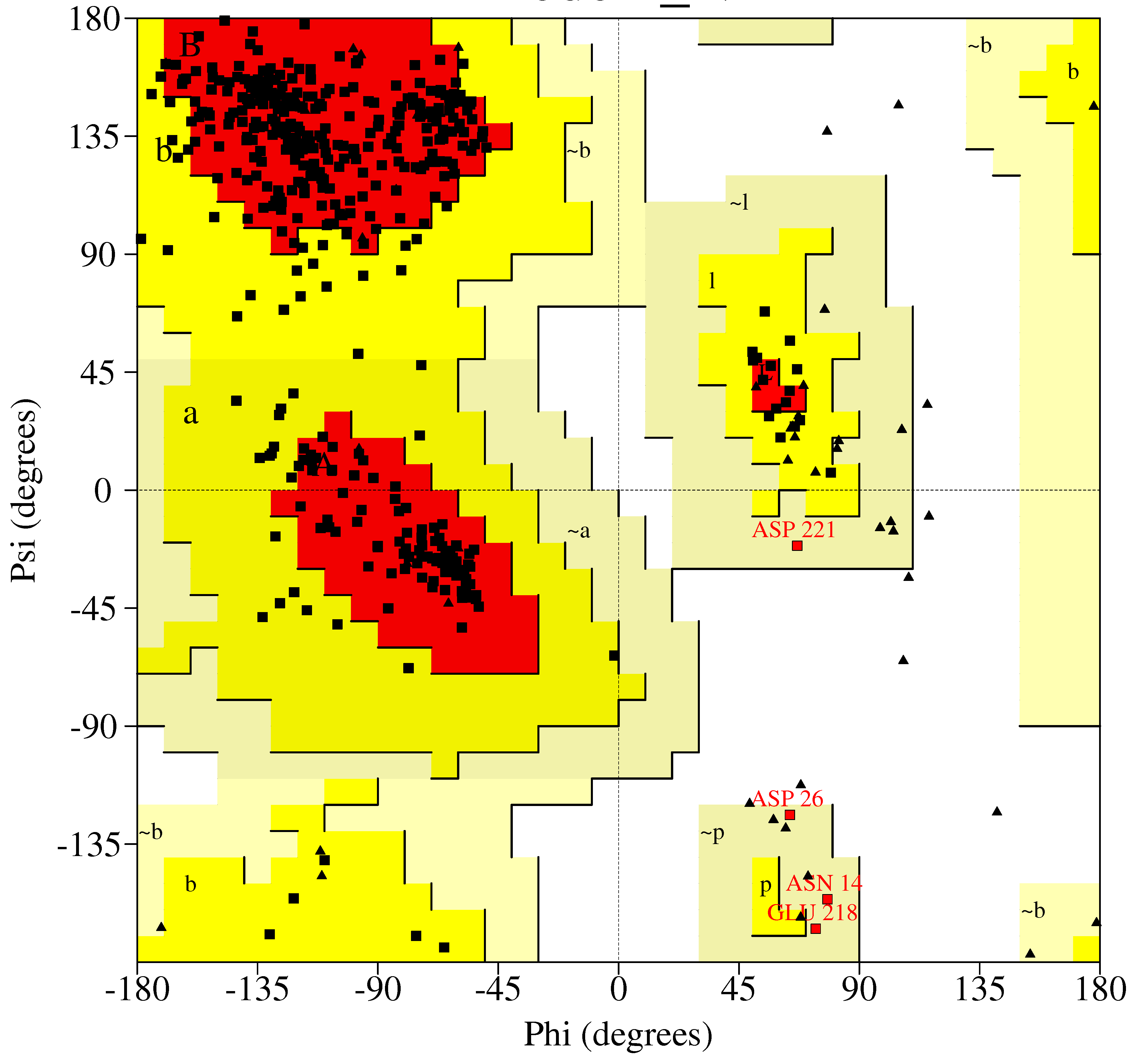

Supplement: S5 Dataset — The plots were generated through PROCHECK analysis. (ZIP) [file pone.0200607.s005.zip › Ramachandranplots/ACP1.tiff]

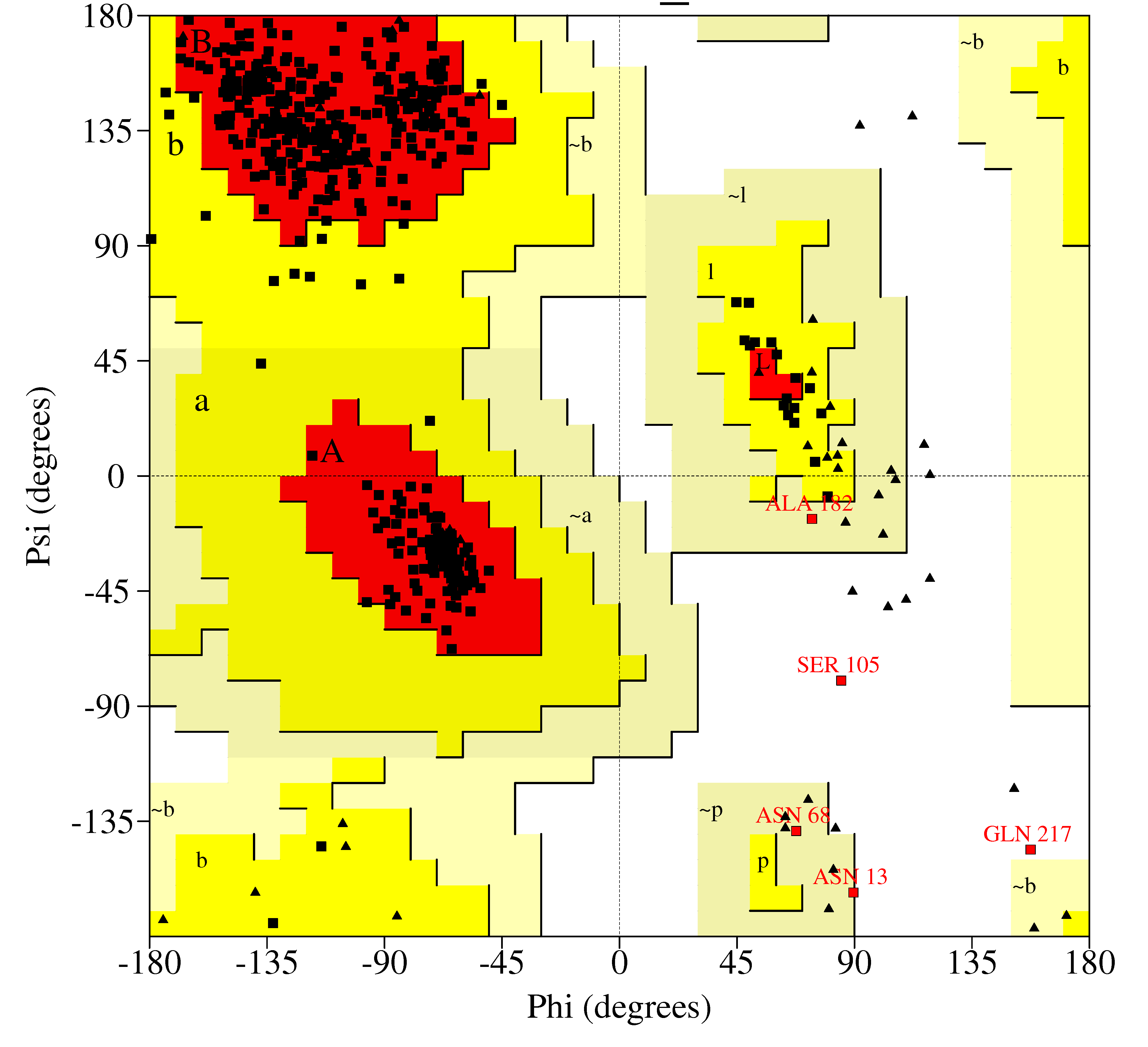

Supplement: S5 Dataset — The plots were generated through PROCHECK analysis. (ZIP) [file pone.0200607.s005.zip › Ramachandranplots/ACP2.tiff]

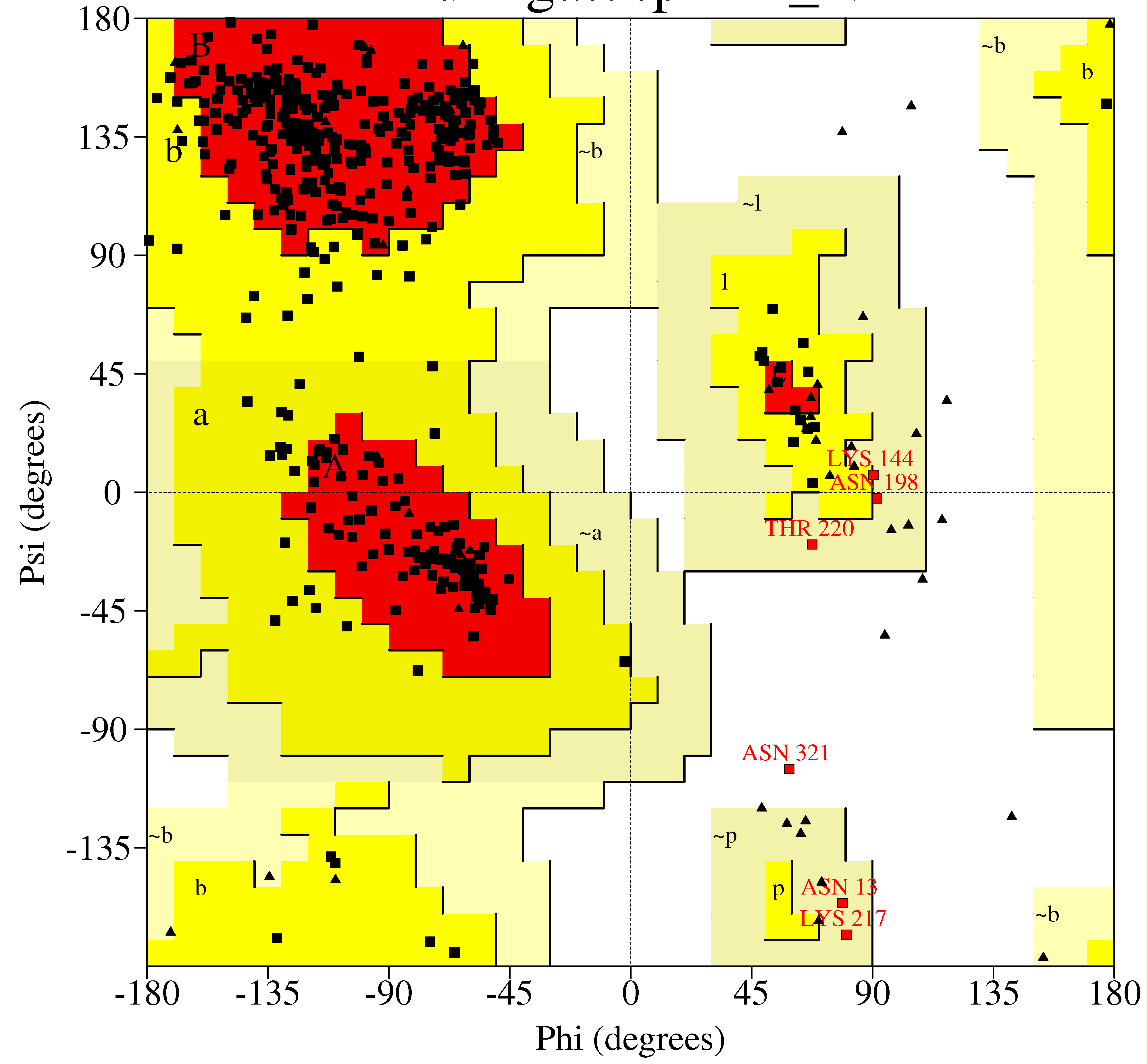

Supplement: S5 Dataset — The plots were generated through PROCHECK analysis. (ZIP) [file pone.0200607.s005.zip › Ramachandranplots/AFP1.tiff]

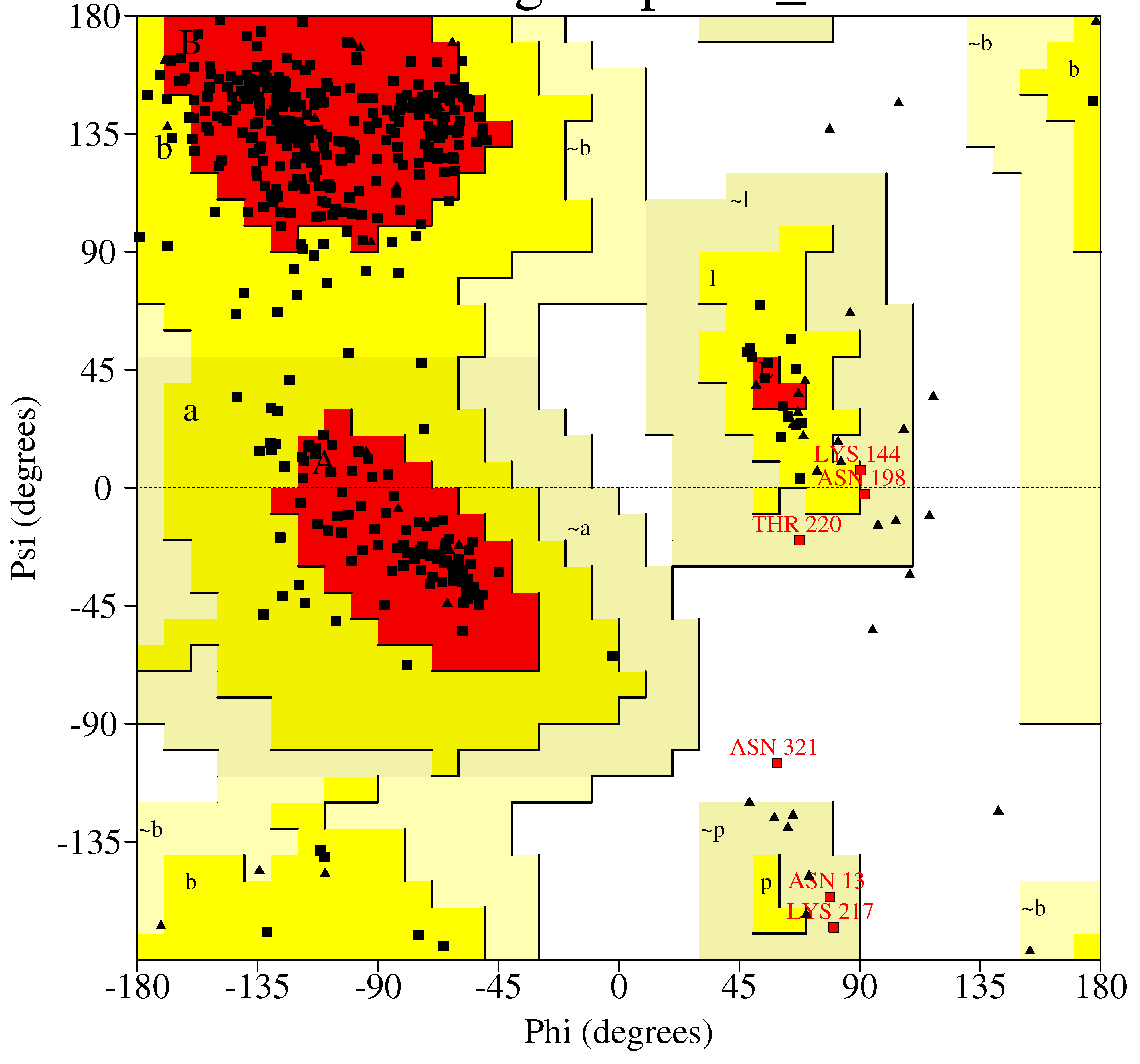

Supplement: S5 Dataset — The plots were generated through PROCHECK analysis. (ZIP) [file pone.0200607.s005.zip › Ramachandranplots/AFP2.tiff]

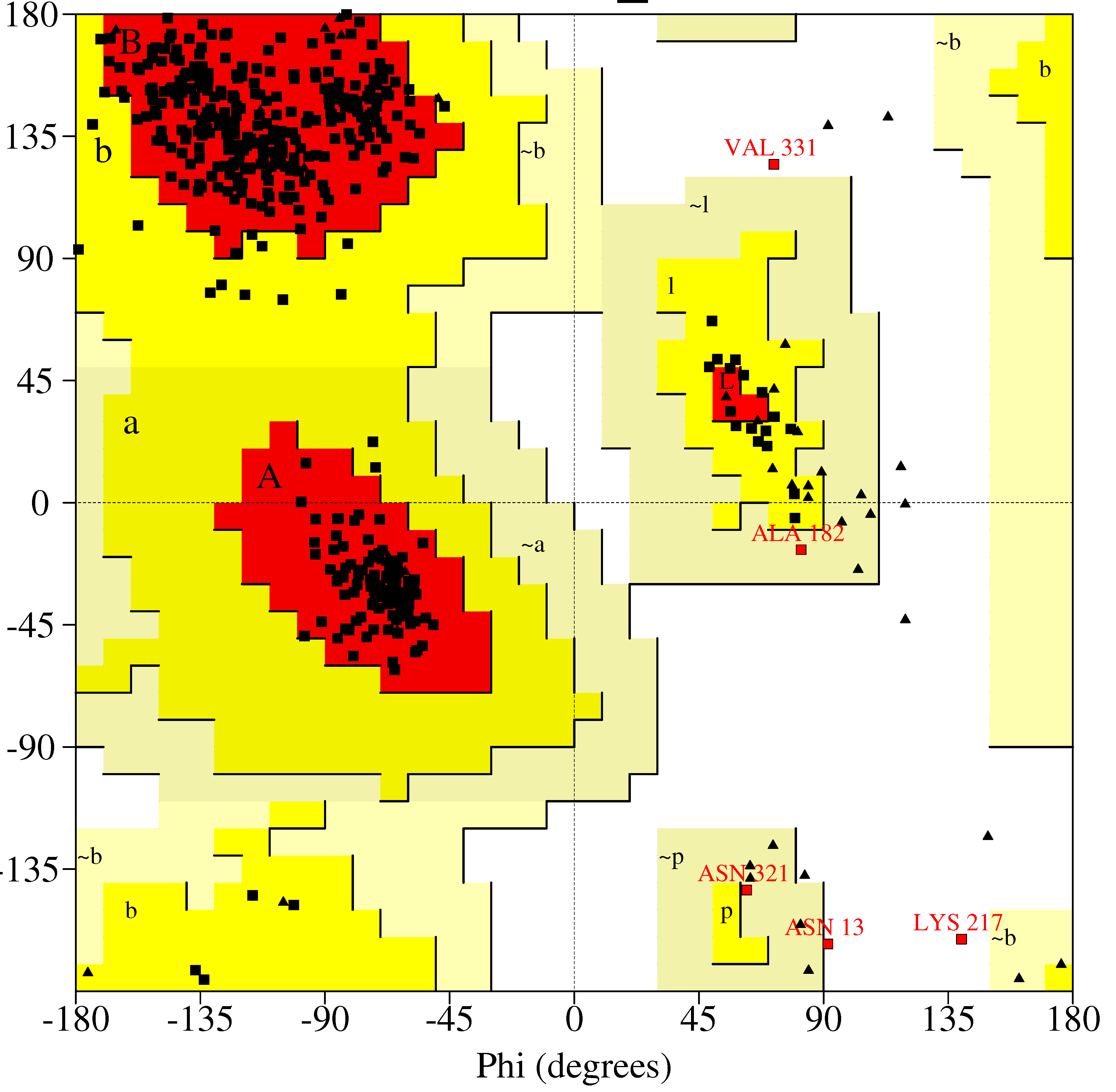

Supplement: S5 Dataset — The plots were generated through PROCHECK analysis. (ZIP) [file pone.0200607.s005.zip › Ramachandranplots/AFSP1.tiff]

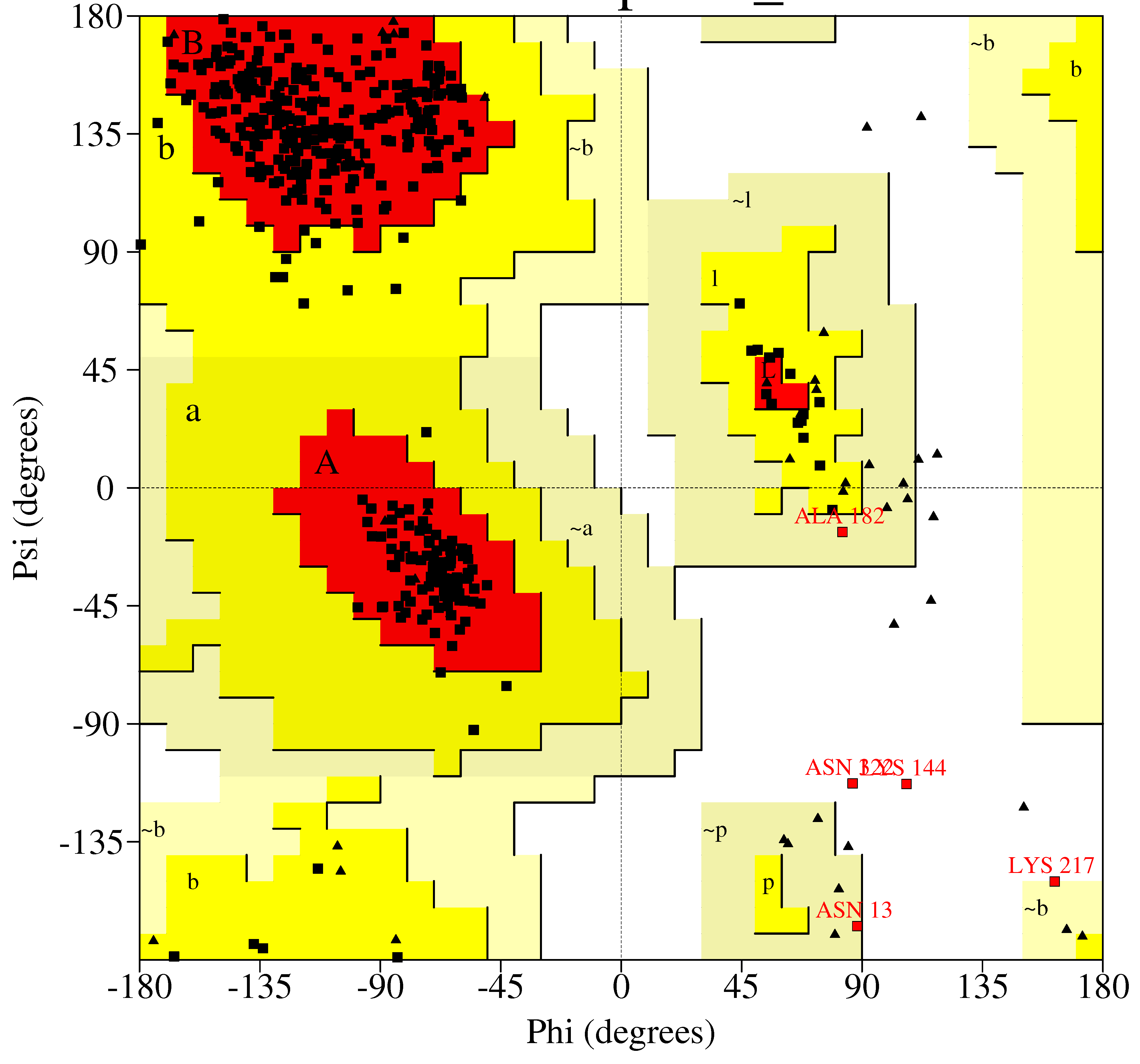

Supplement: S5 Dataset — The plots were generated through PROCHECK analysis. (ZIP) [file pone.0200607.s005.zip › Ramachandranplots/ALP1.tiff]

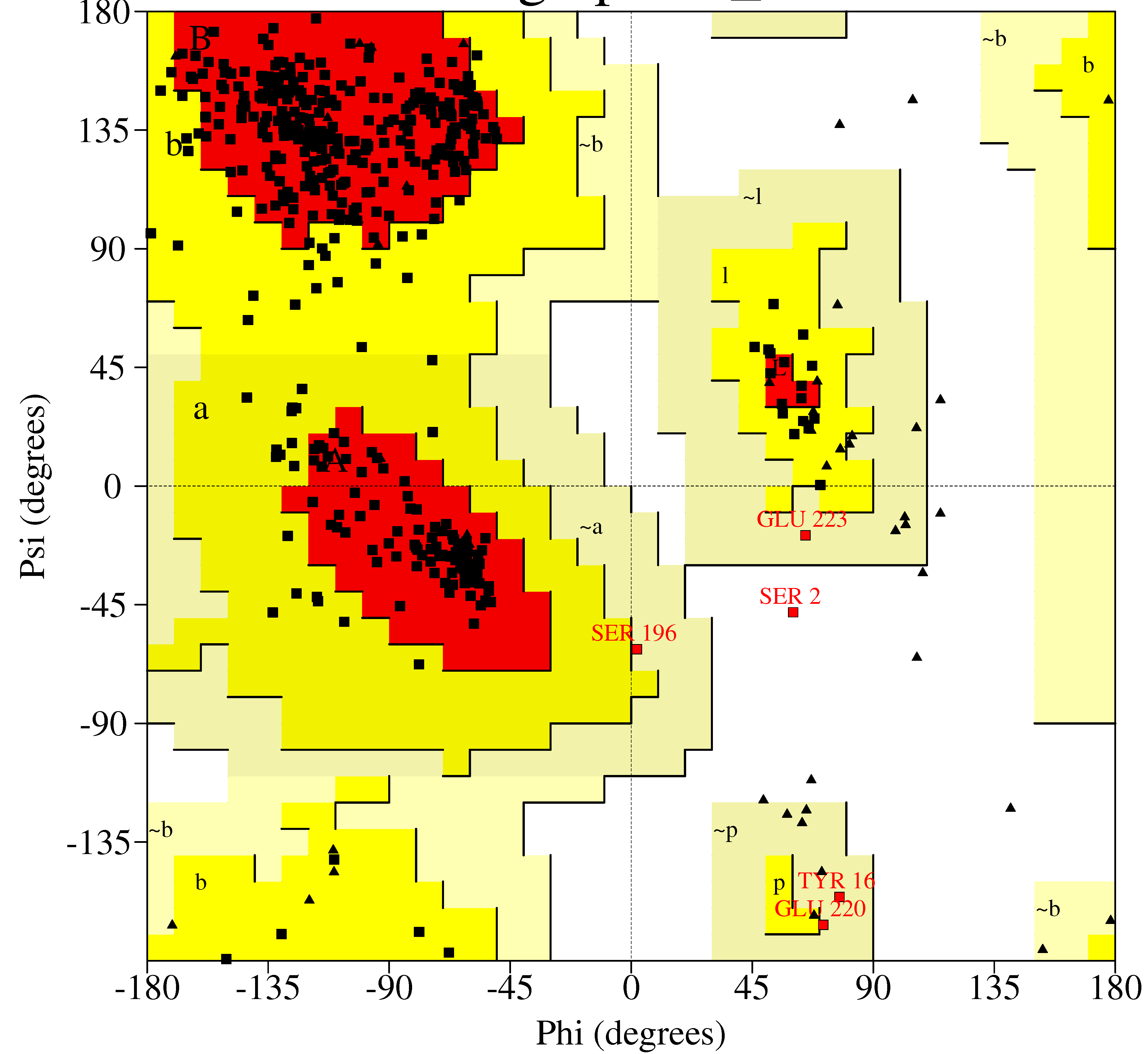

Supplement: S5 Dataset — The plots were generated through PROCHECK analysis. (ZIP) [file pone.0200607.s005.zip › Ramachandranplots/ANP1.tiff]

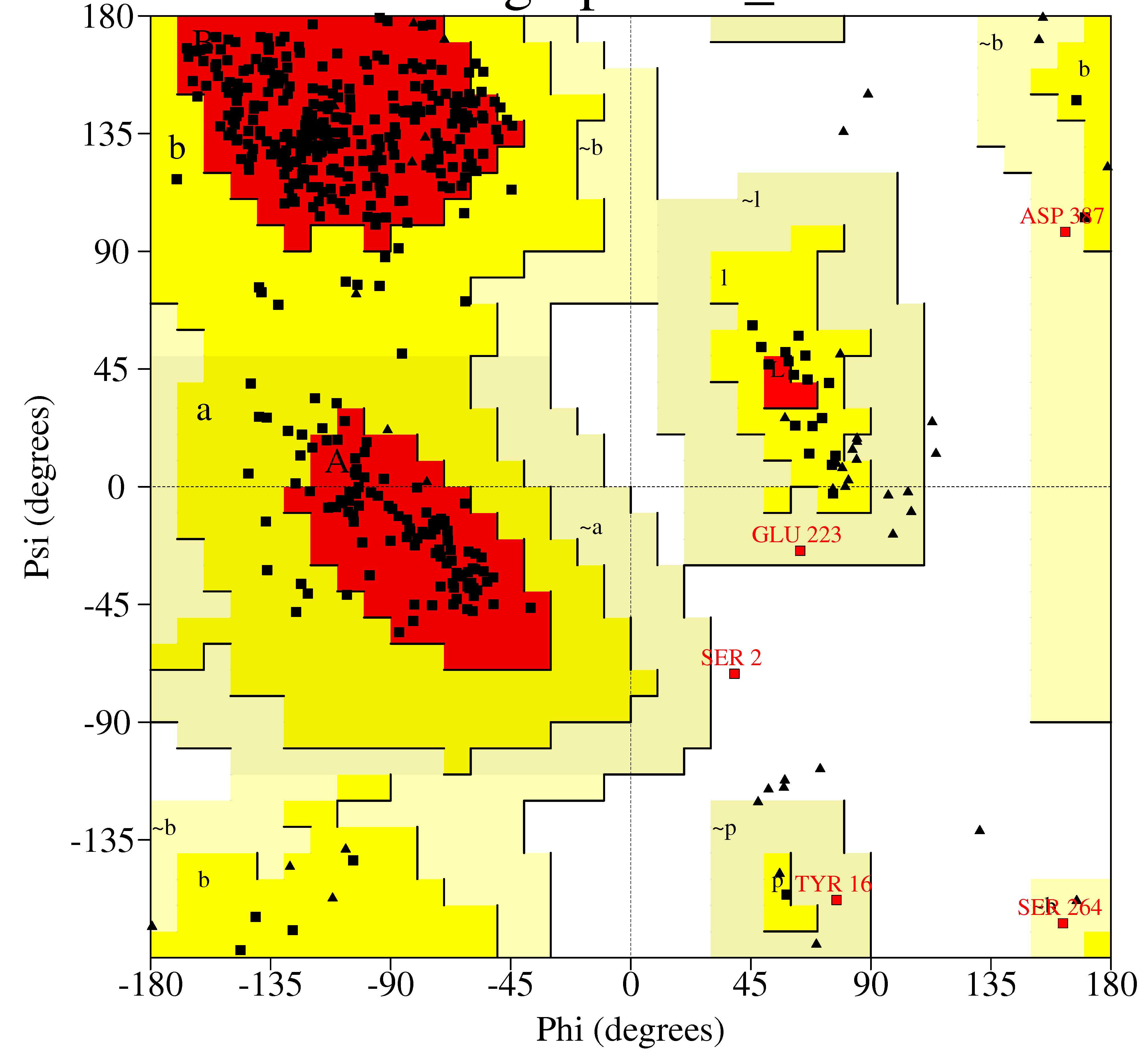

Supplement: S5 Dataset — The plots were generated through PROCHECK analysis. (ZIP) [file pone.0200607.s005.zip › Ramachandranplots/ANP10.tiff]

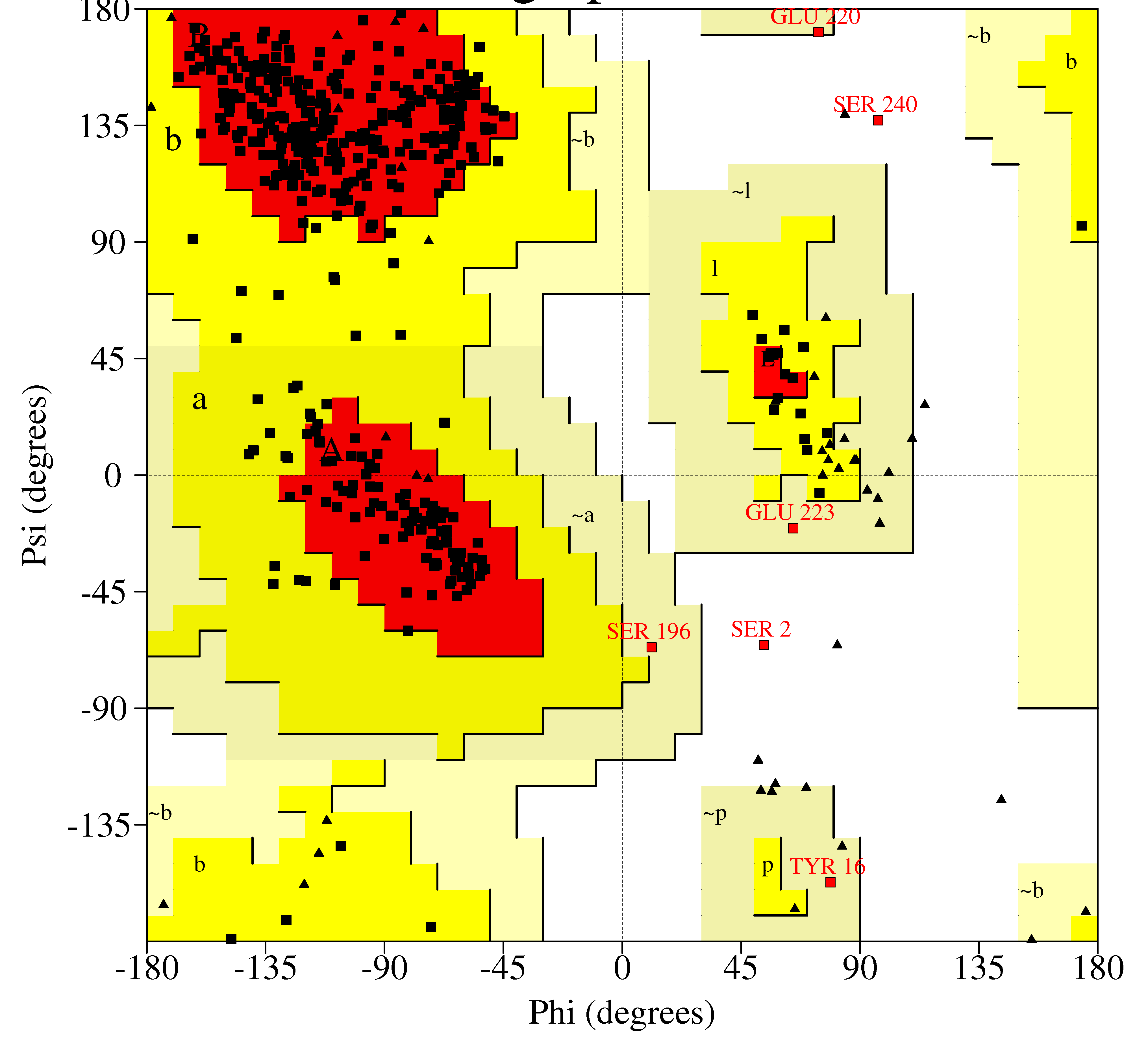

Supplement: S5 Dataset — The plots were generated through PROCHECK analysis. (ZIP) [file pone.0200607.s005.zip › Ramachandranplots/ANP2.tiff]

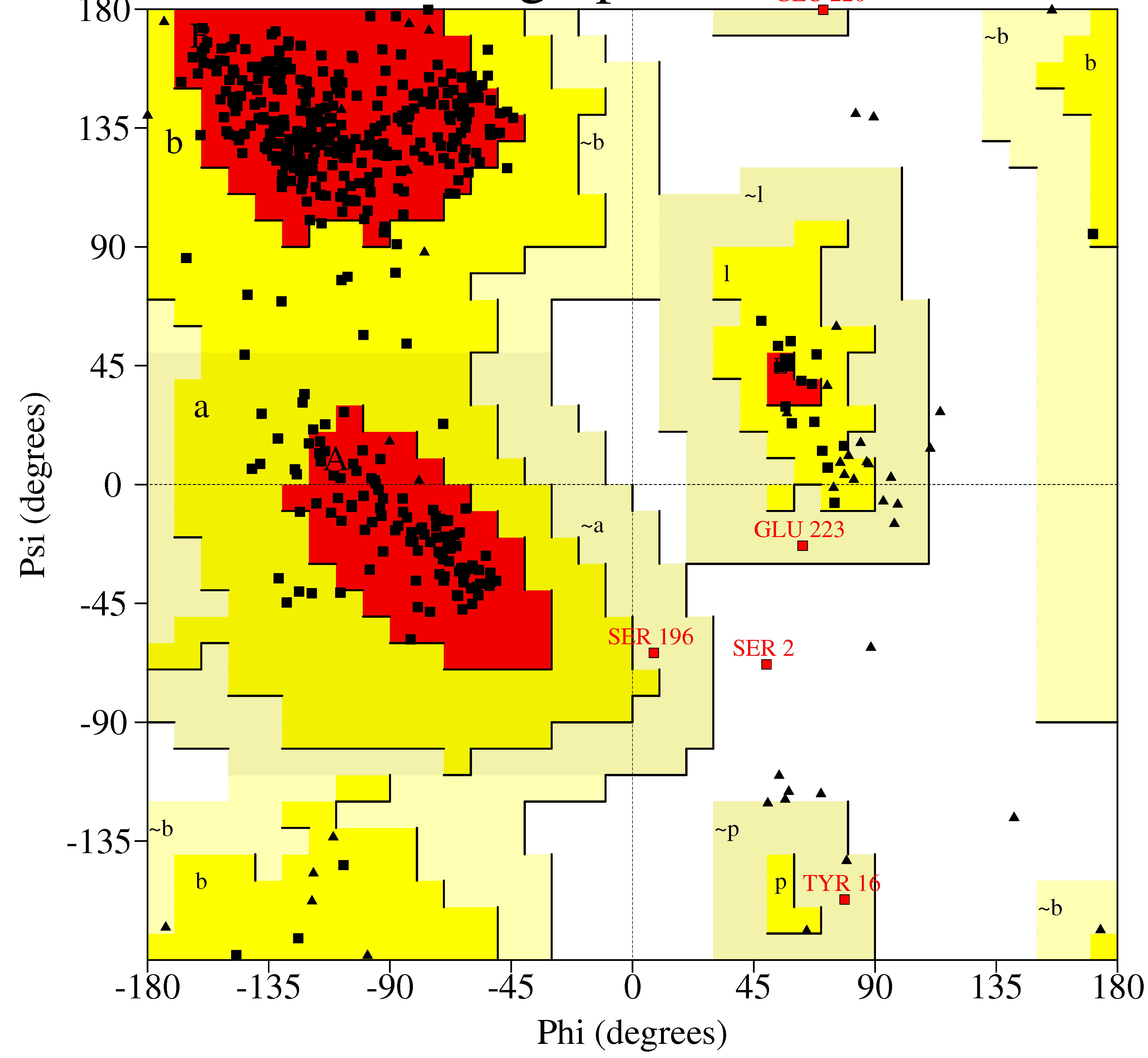

Supplement: S5 Dataset — The plots were generated through PROCHECK analysis. (ZIP) [file pone.0200607.s005.zip › Ramachandranplots/ANP3.tiff]

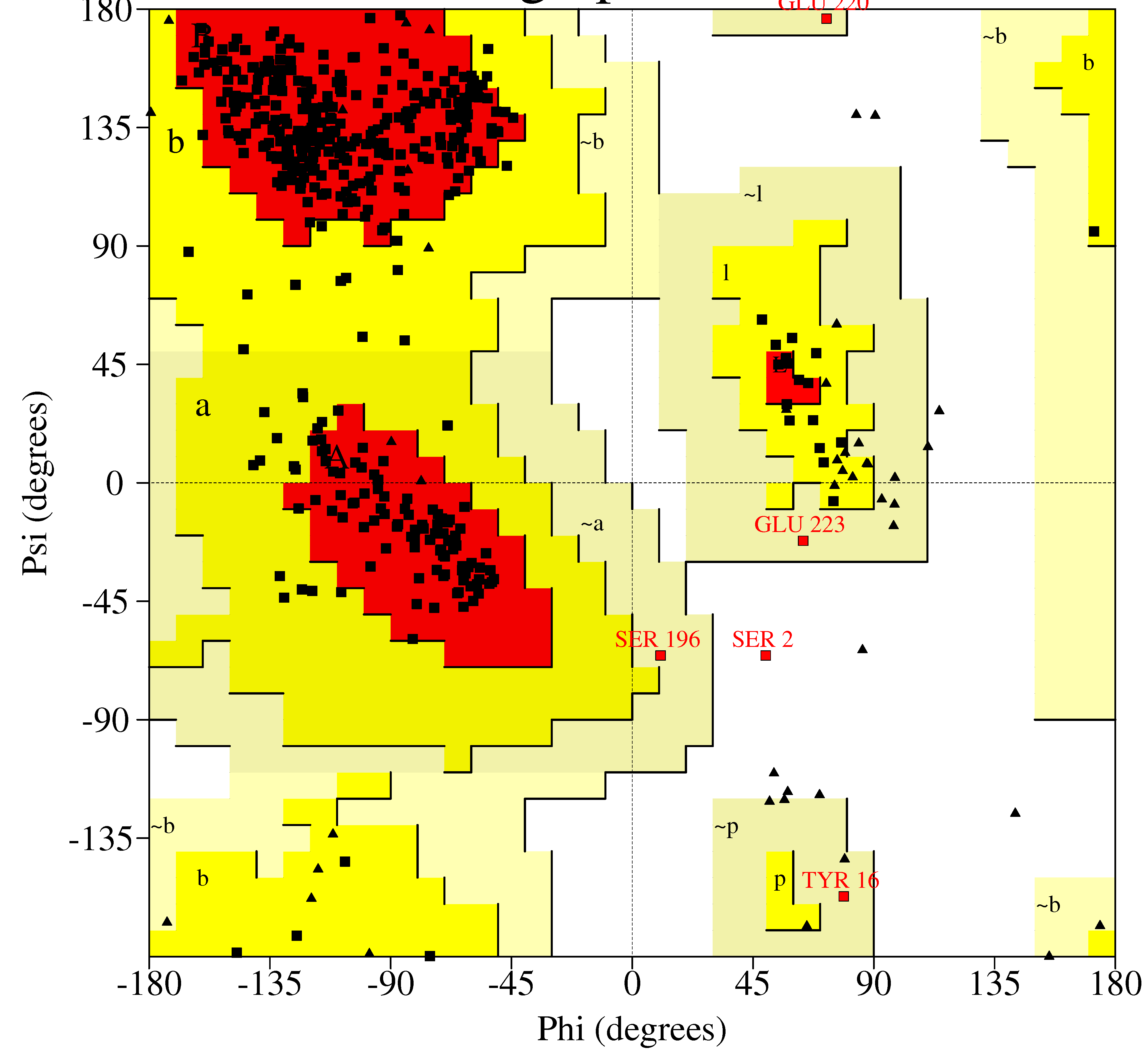

Supplement: S5 Dataset — The plots were generated through PROCHECK analysis. (ZIP) [file pone.0200607.s005.zip › Ramachandranplots/ANP4.tiff]

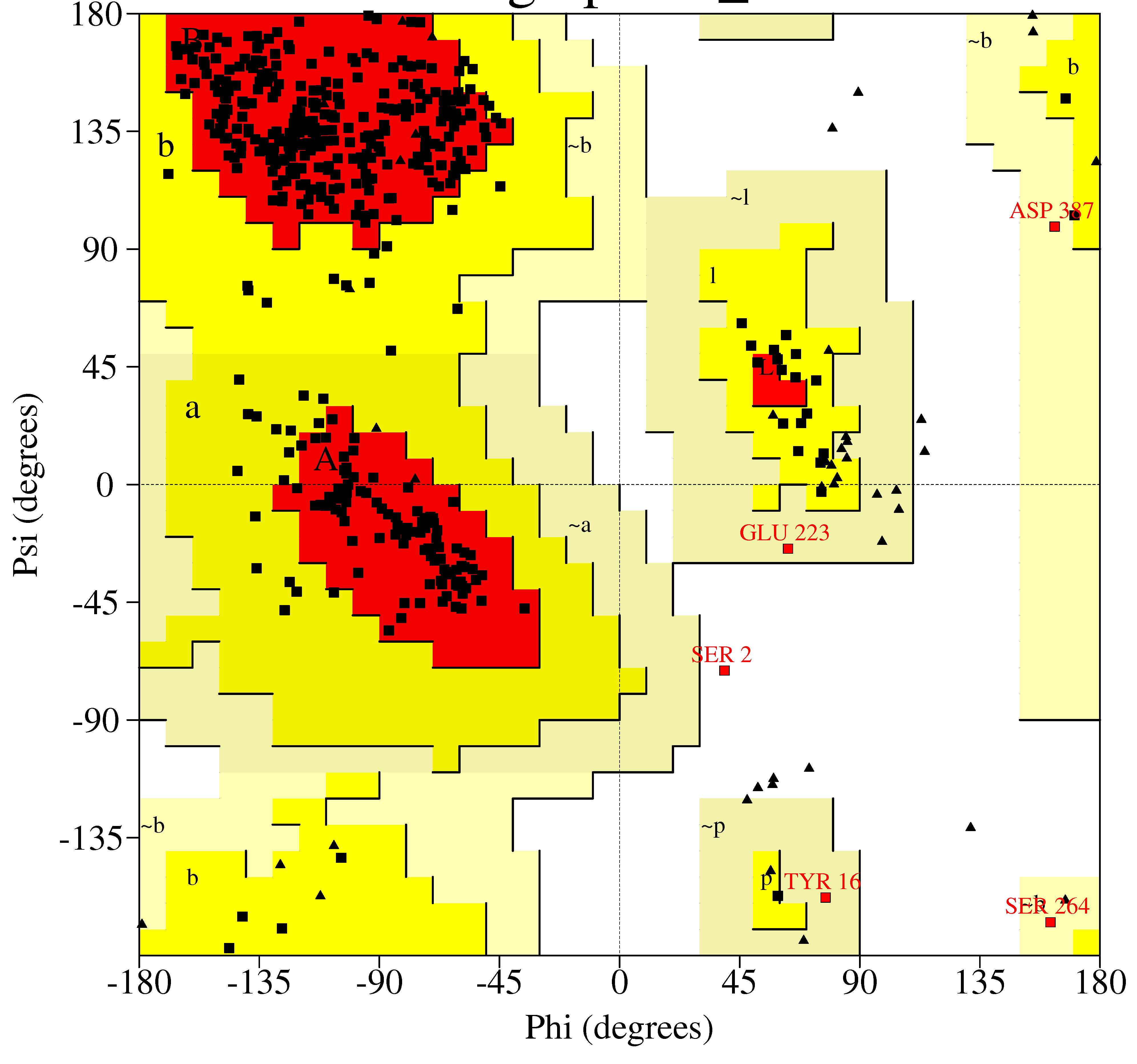

Supplement: S5 Dataset — The plots were generated through PROCHECK analysis. (ZIP) [file pone.0200607.s005.zip › Ramachandranplots/ANP5.tiff]

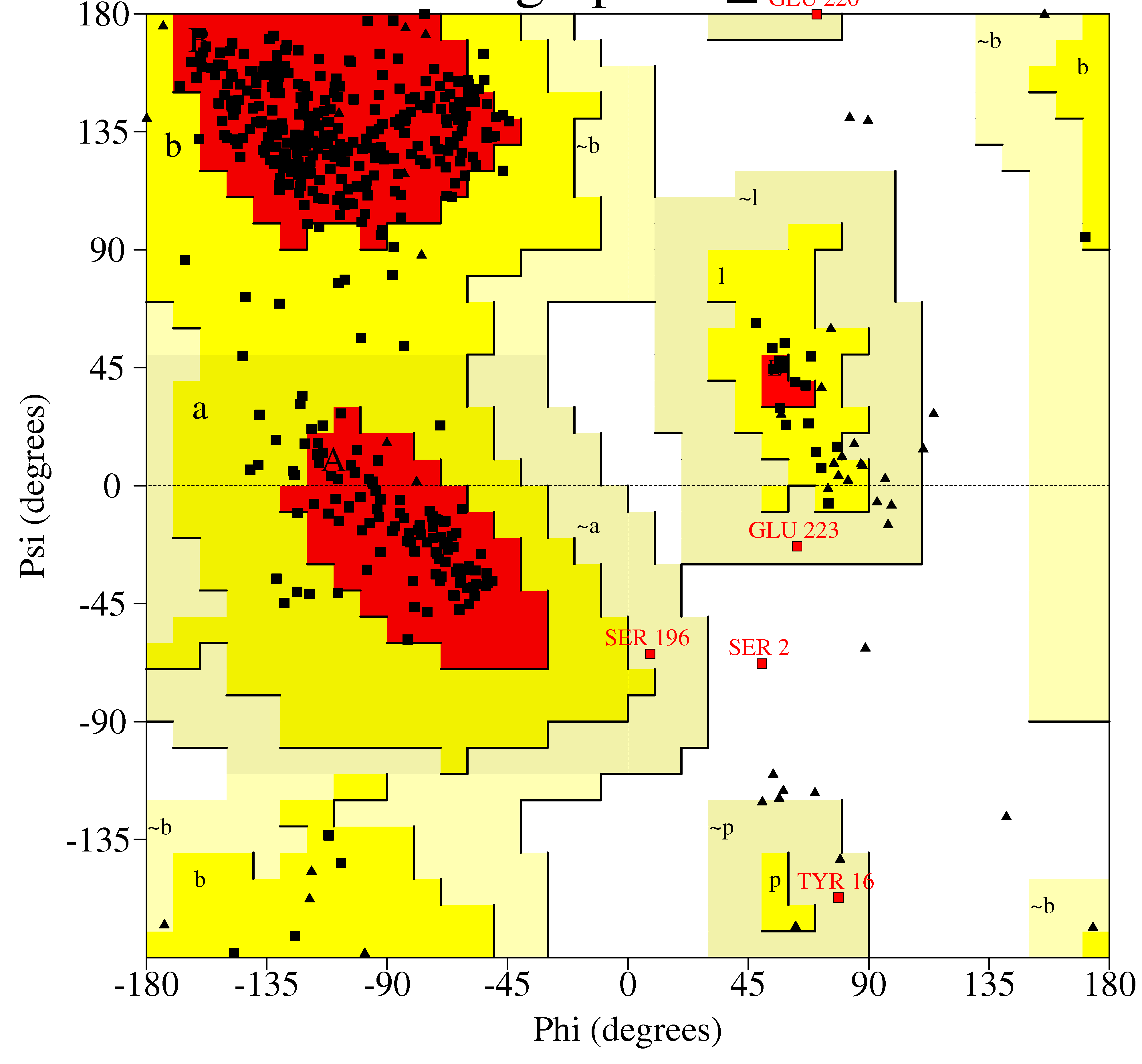

Supplement: S5 Dataset — The plots were generated through PROCHECK analysis. (ZIP) [file pone.0200607.s005.zip › Ramachandranplots/ANP7.tiff]

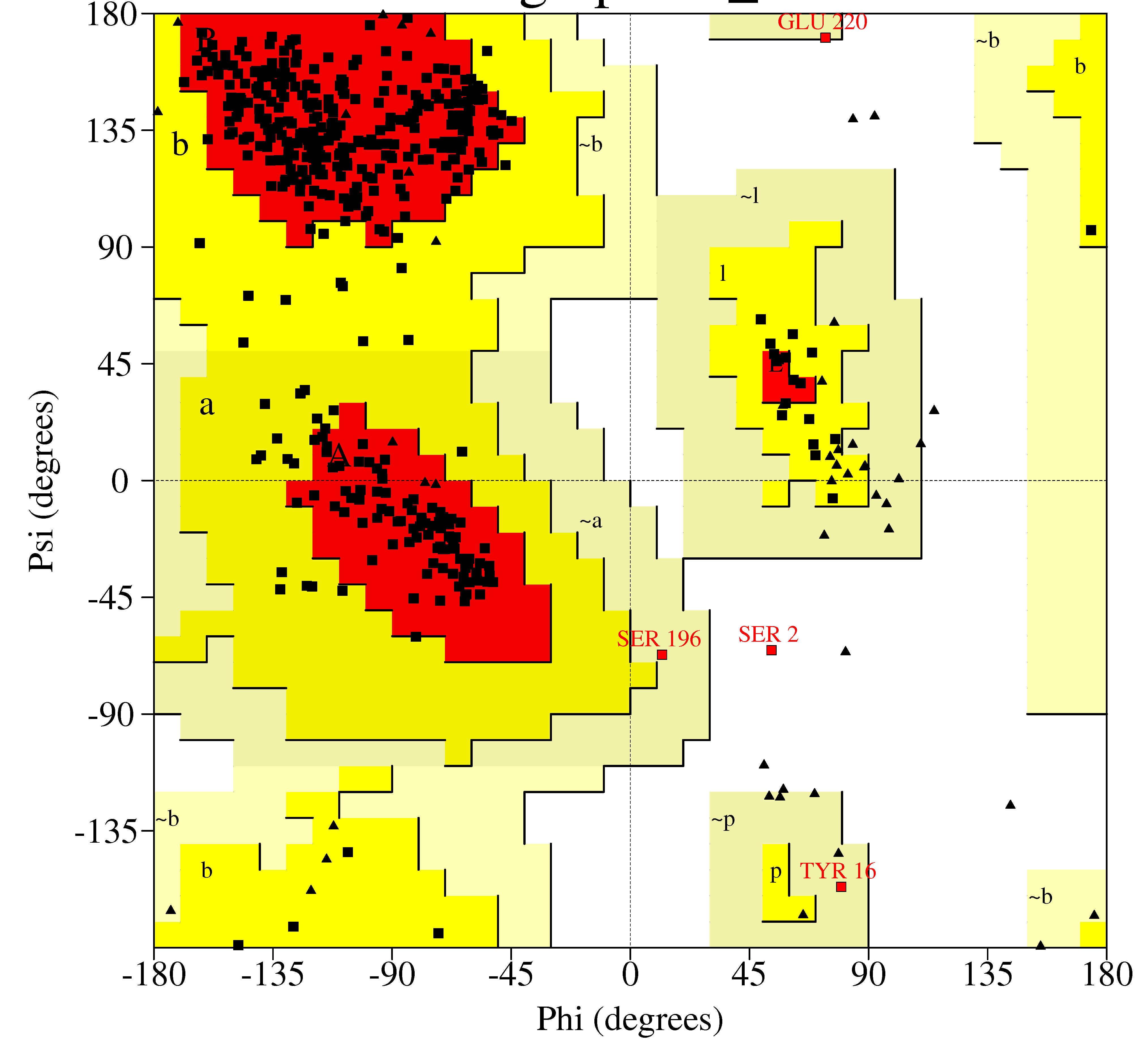

Supplement: S5 Dataset — The plots were generated through PROCHECK analysis. (ZIP) [file pone.0200607.s005.zip › Ramachandranplots/ANP9.tiff]

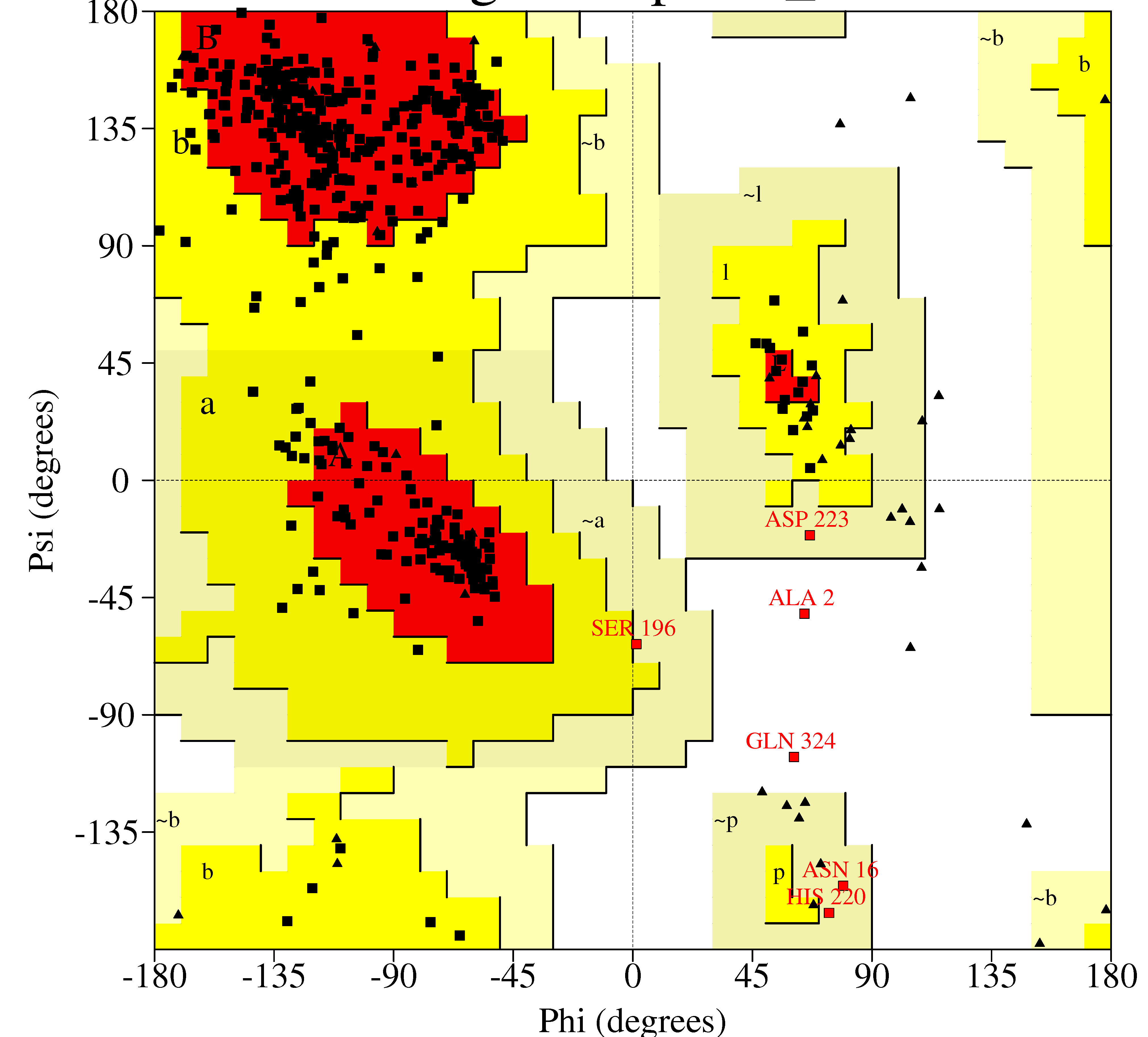

Supplement: S5 Dataset — The plots were generated through PROCHECK analysis. (ZIP) [file pone.0200607.s005.zip › Ramachandranplots/ARP1.tiff]

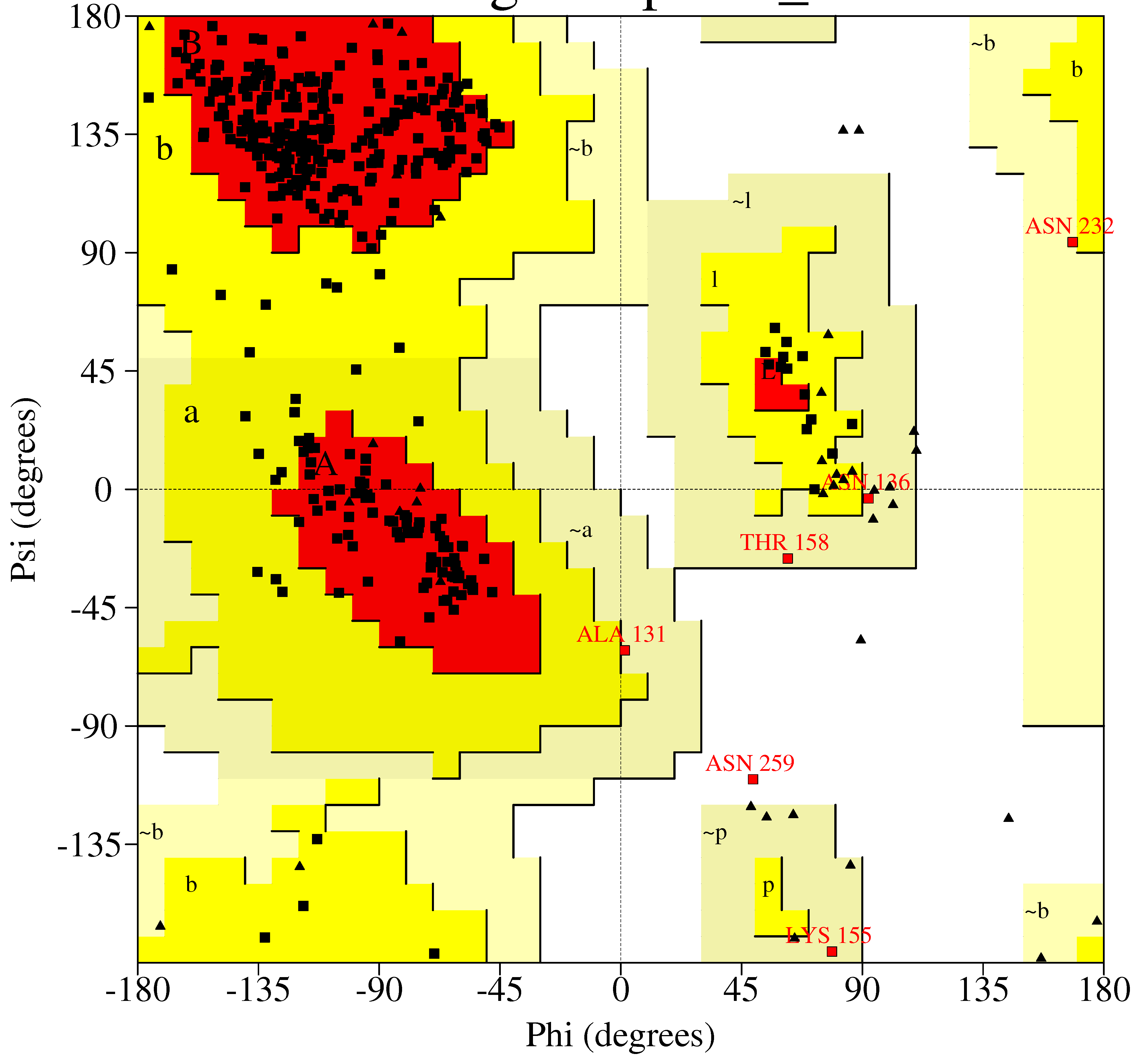

Supplement: S5 Dataset — The plots were generated through PROCHECK analysis. (ZIP) [file pone.0200607.s005.zip › Ramachandranplots/AUP1.tiff]

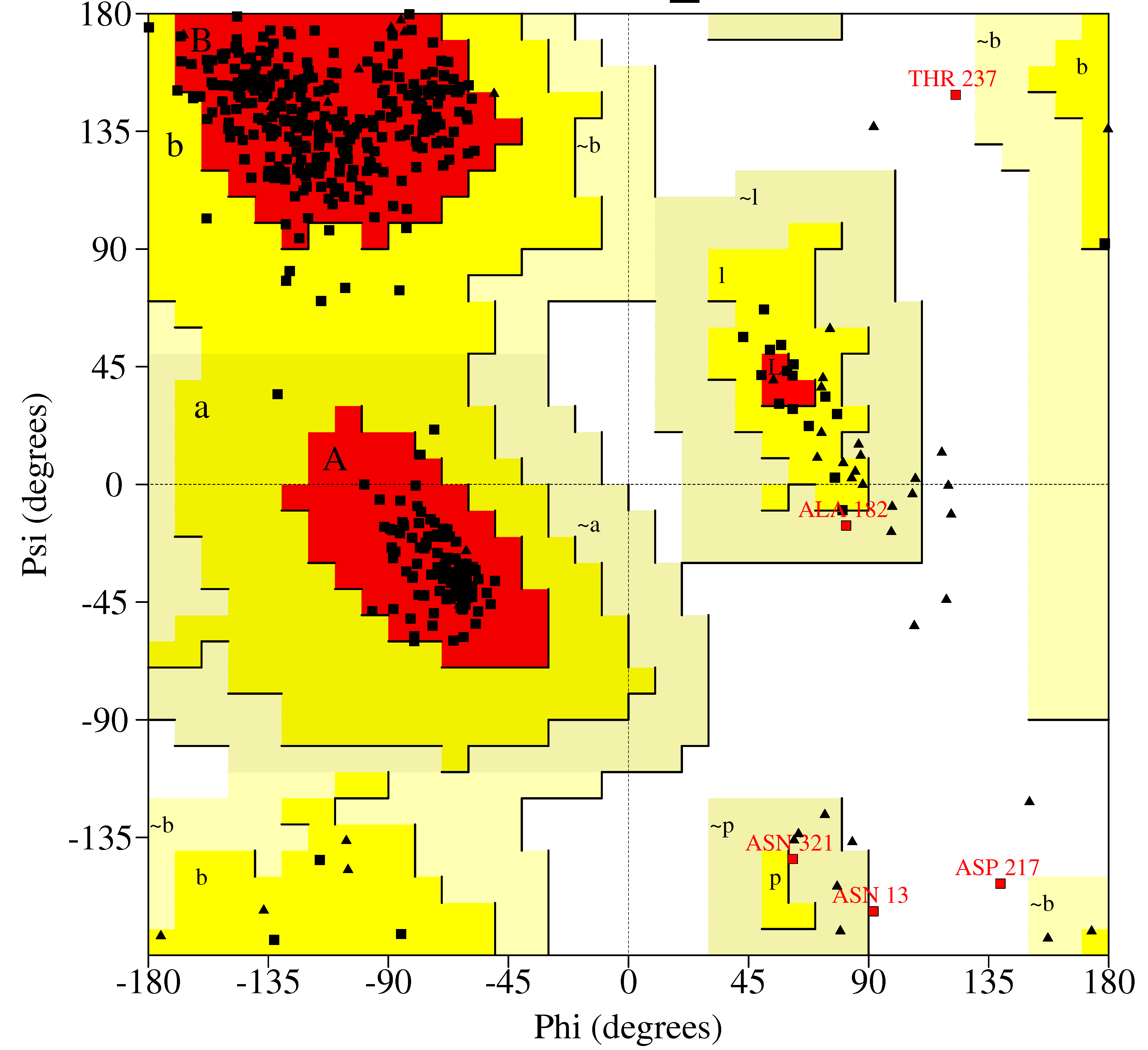

Supplement: S5 Dataset — The plots were generated through PROCHECK analysis. (ZIP) [file pone.0200607.s005.zip › Ramachandranplots/BCP1.tiff]

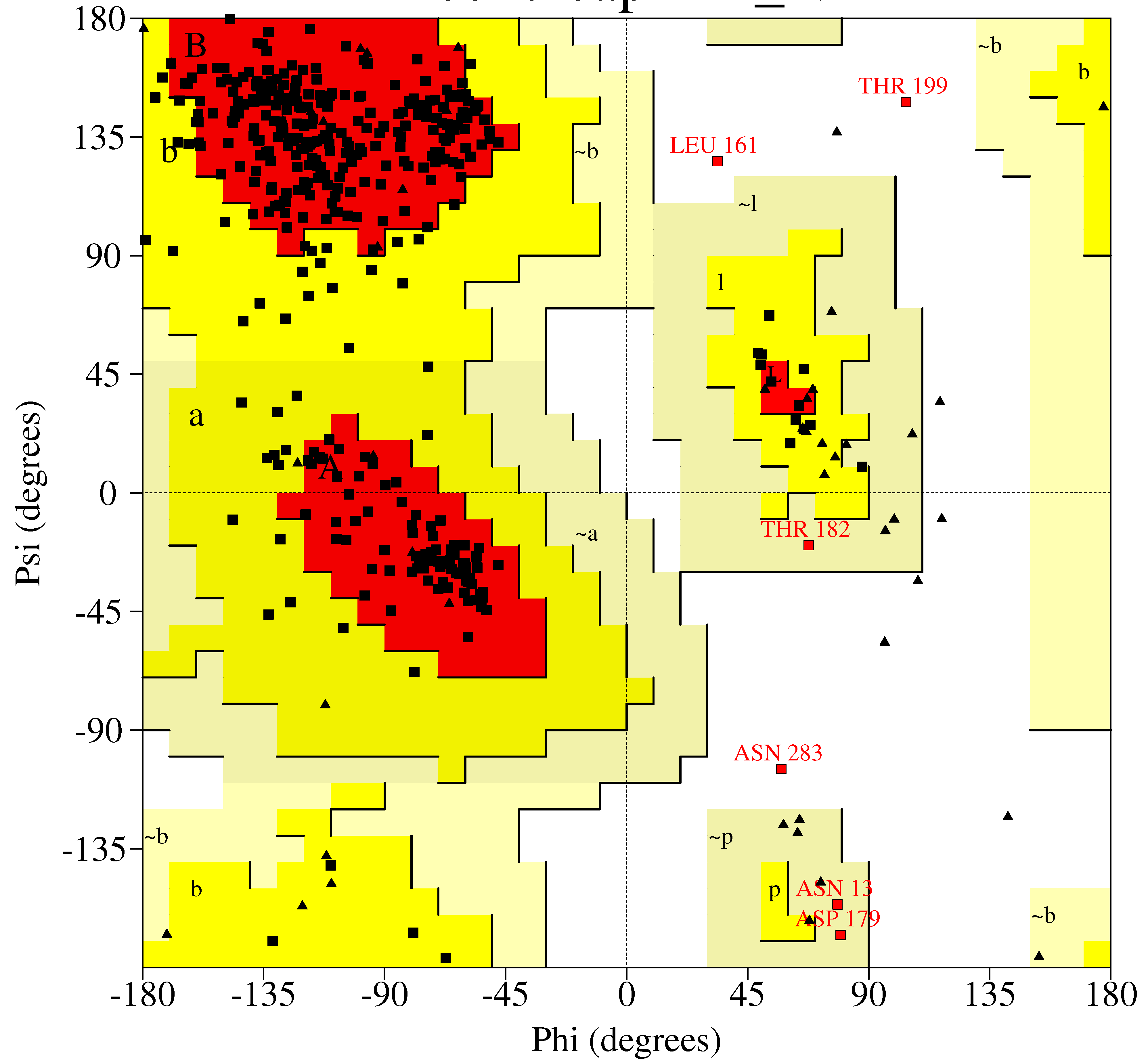

Supplement: S5 Dataset — The plots were generated through PROCHECK analysis. (ZIP) [file pone.0200607.s005.zip › Ramachandranplots/BCP2.tiff]

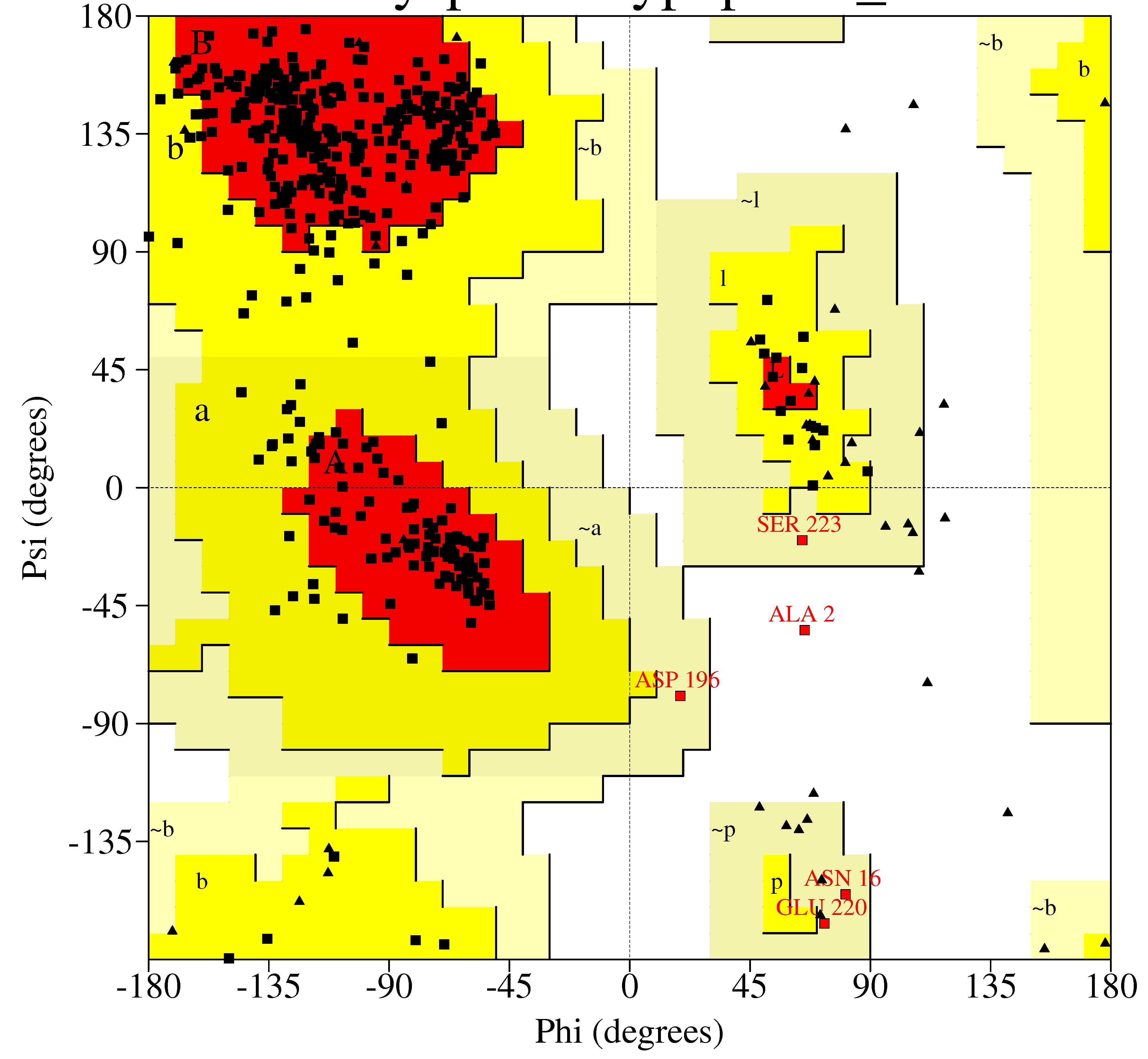

Supplement: S5 Dataset — The plots were generated through PROCHECK analysis. (ZIP) [file pone.0200607.s005.zip › Ramachandranplots/FOHP1.tiff]

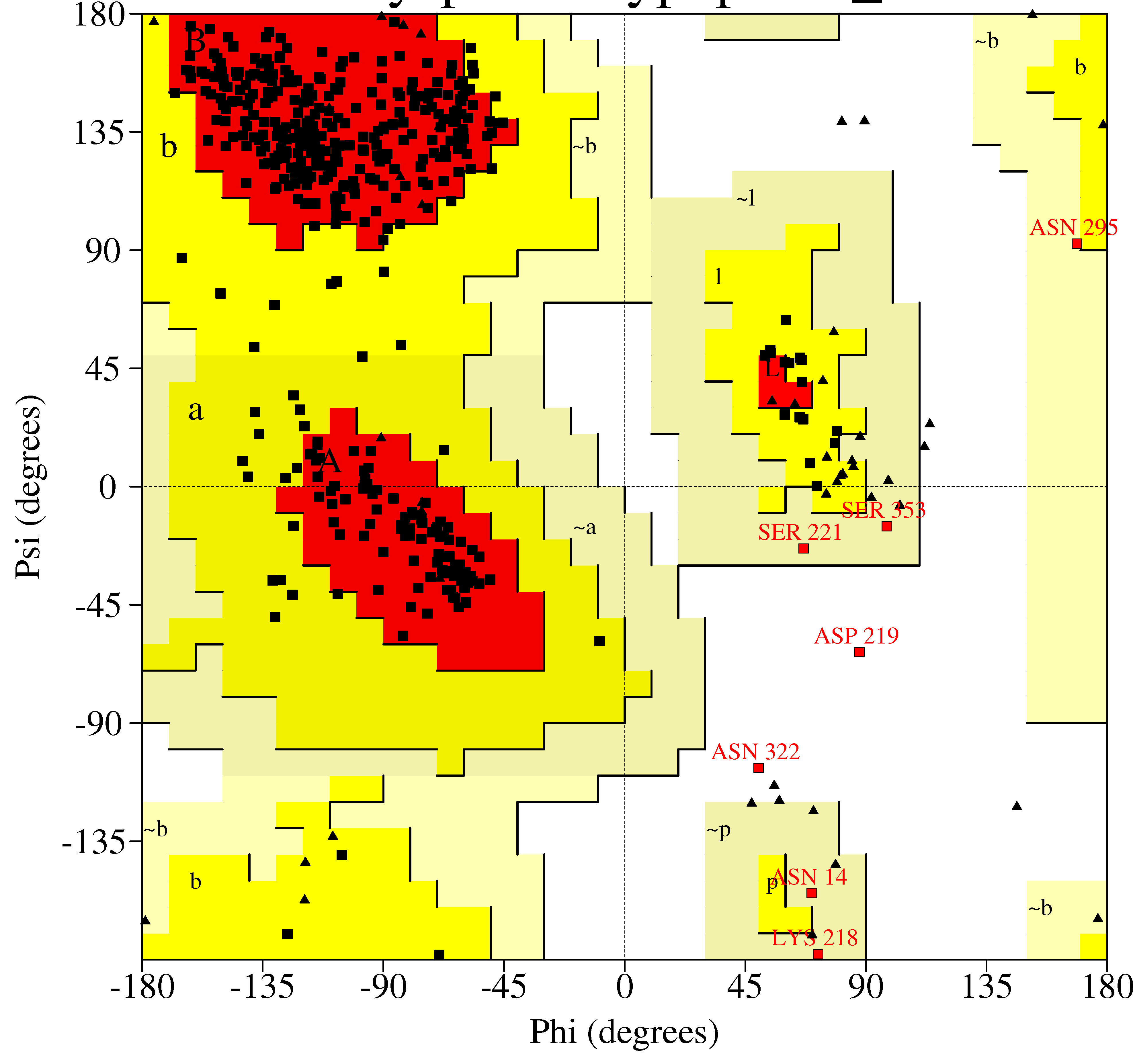

Supplement: S5 Dataset — The plots were generated through PROCHECK analysis. (ZIP) [file pone.0200607.s005.zip › Ramachandranplots/FOHP7.tiff]

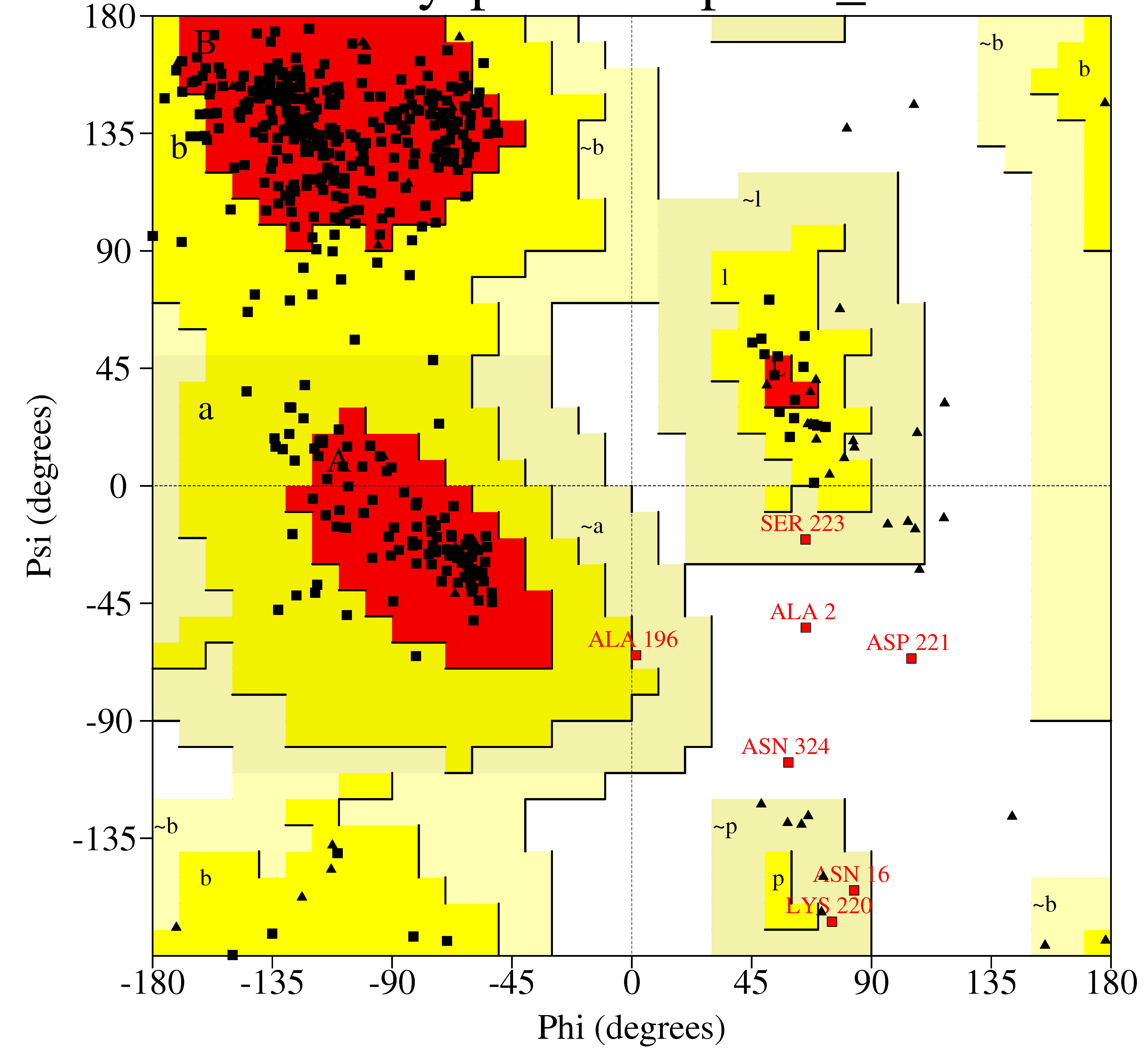

Supplement: S5 Dataset — The plots were generated through PROCHECK analysis. (ZIP) [file pone.0200607.s005.zip › Ramachandranplots/FOP1.tiff]

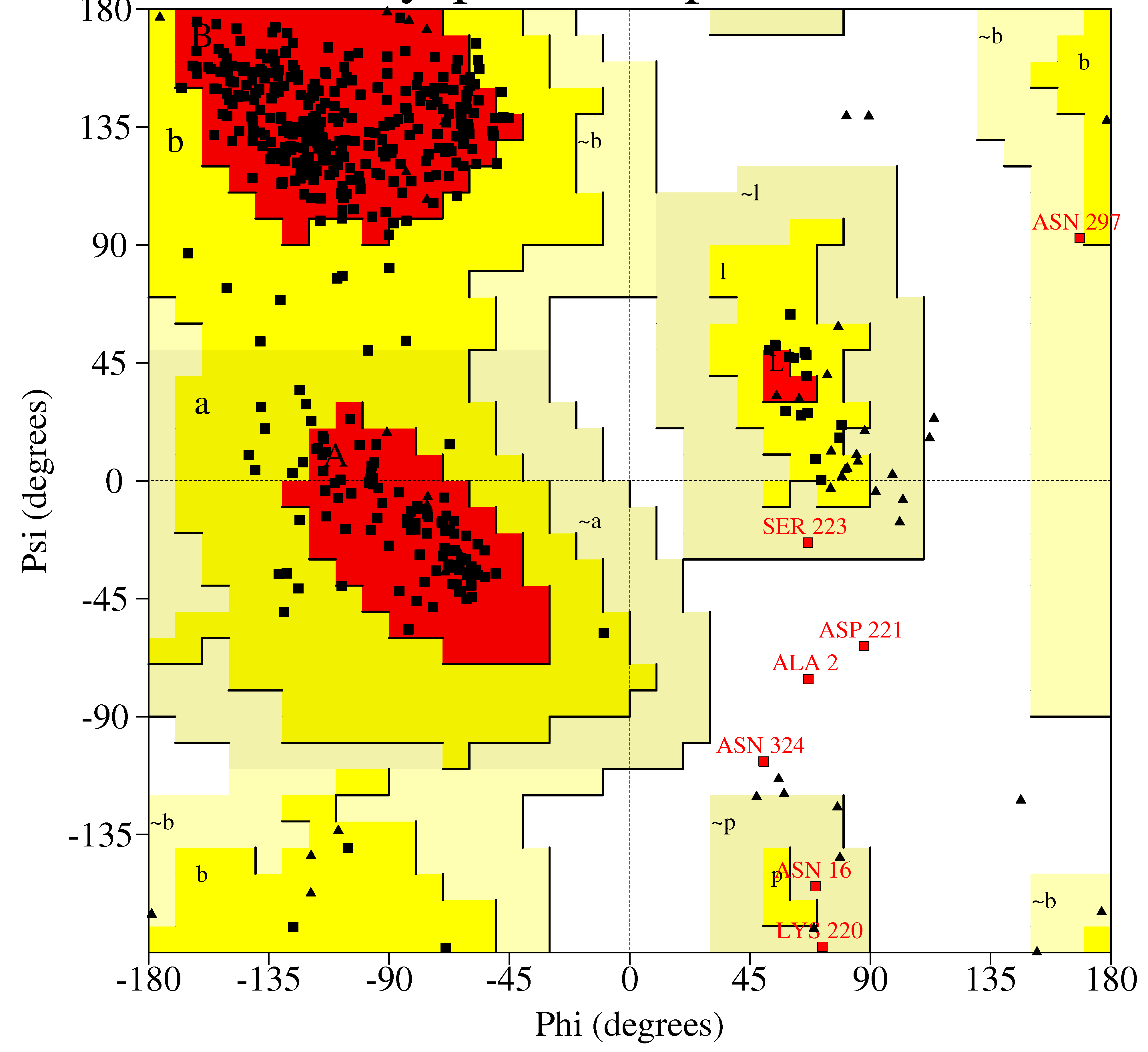

Supplement: S5 Dataset — The plots were generated through PROCHECK analysis. (ZIP) [file pone.0200607.s005.zip › Ramachandranplots/FOP10.tiff]

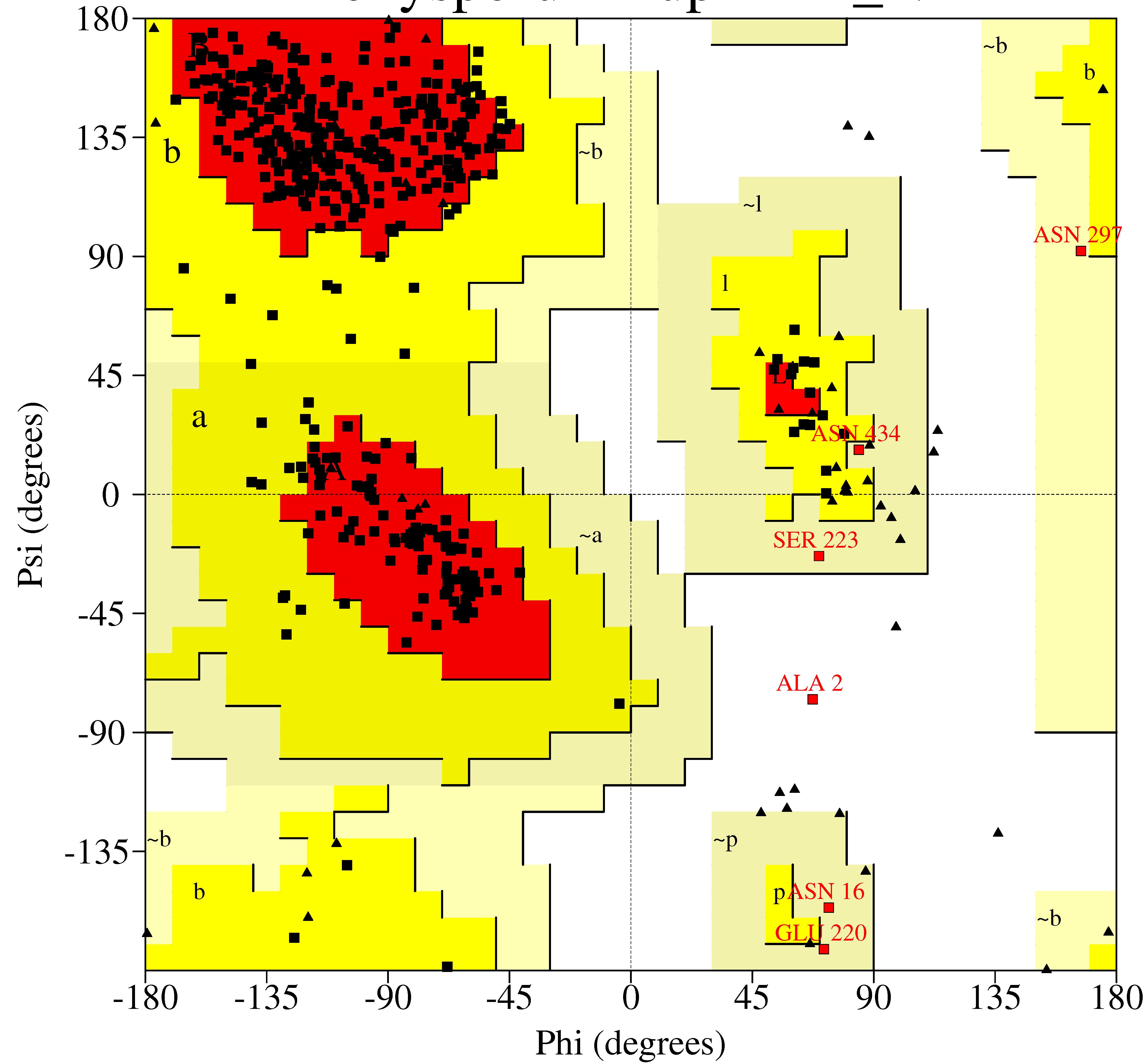

Supplement: S5 Dataset — The plots were generated through PROCHECK analysis. (ZIP) [file pone.0200607.s005.zip › Ramachandranplots/FOP11.tiff]

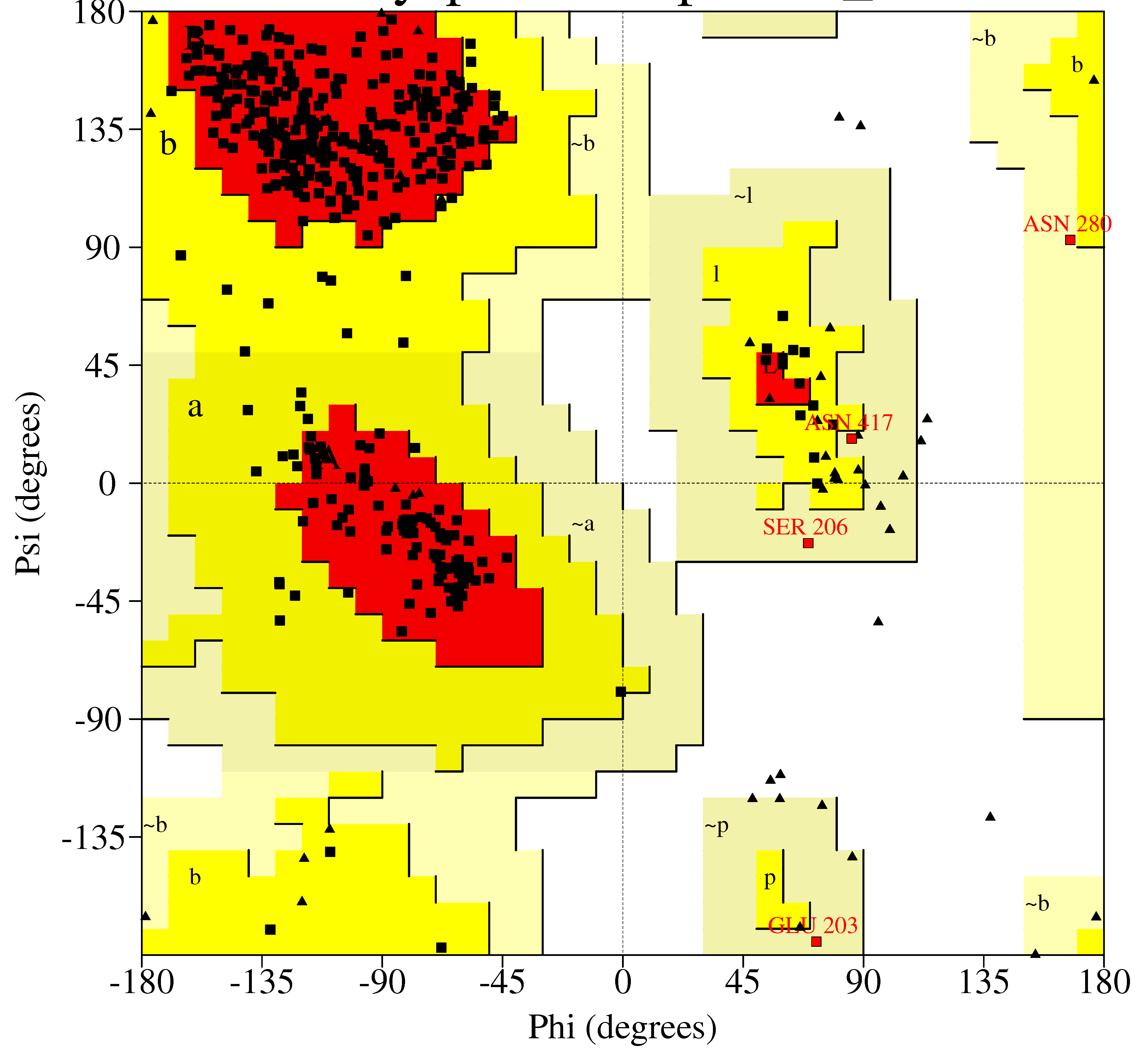

Supplement: S5 Dataset — The plots were generated through PROCHECK analysis. (ZIP) [file pone.0200607.s005.zip › Ramachandranplots/FOP12.tiff]

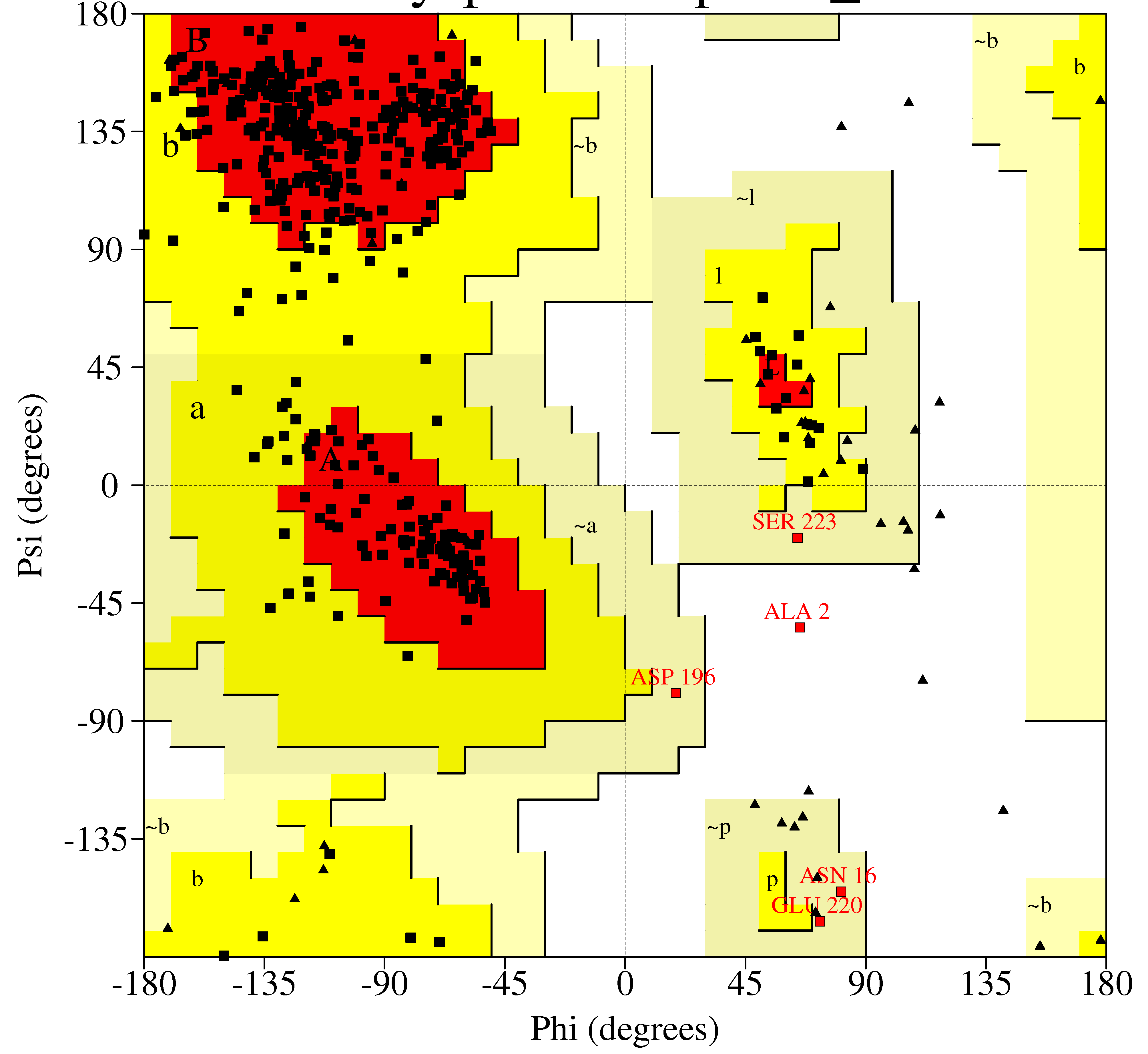

Supplement: S5 Dataset — The plots were generated through PROCHECK analysis. (ZIP) [file pone.0200607.s005.zip › Ramachandranplots/FOP2.tiff]

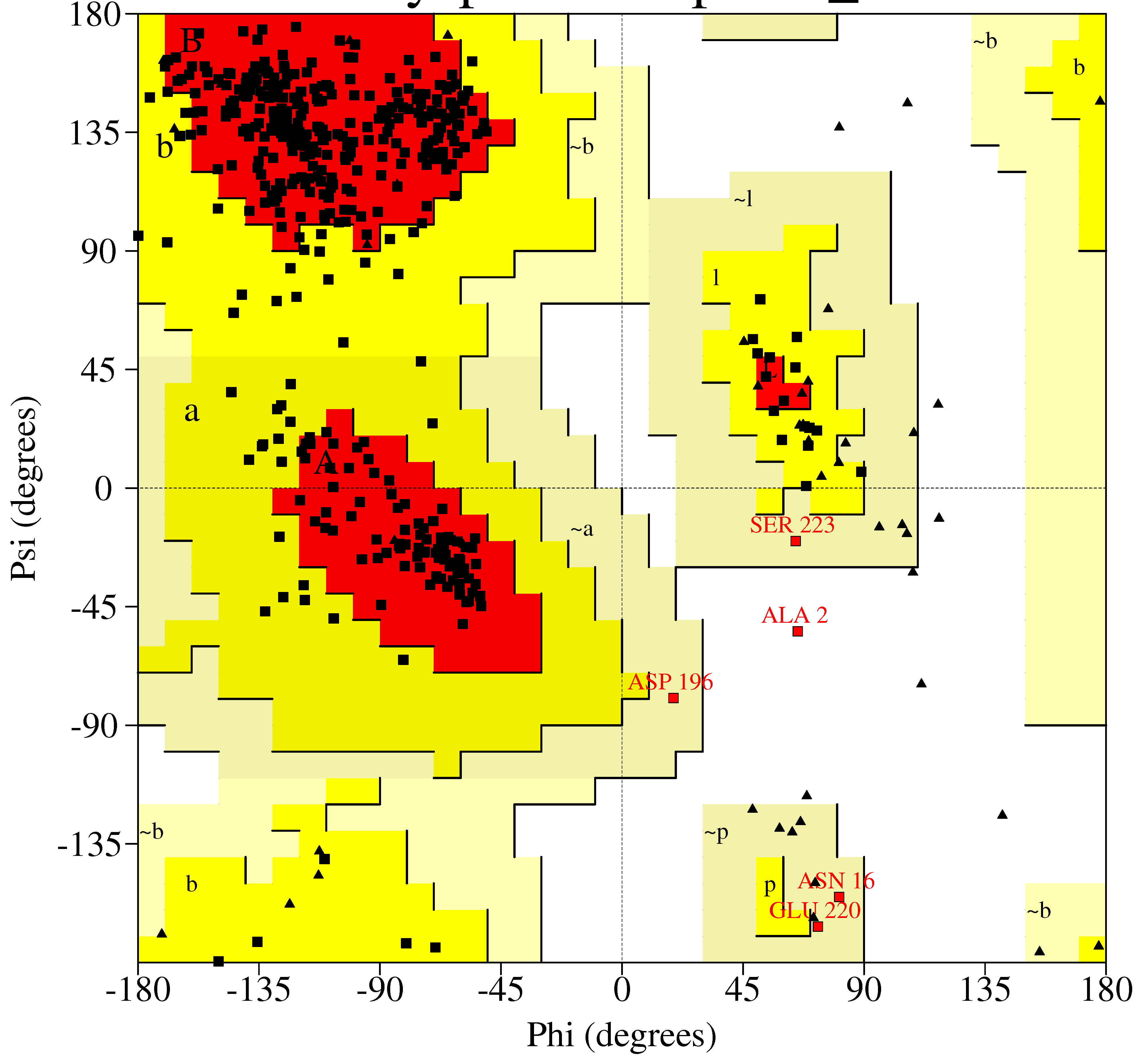

Supplement: S5 Dataset — The plots were generated through PROCHECK analysis. (ZIP) [file pone.0200607.s005.zip › Ramachandranplots/FOP3.tiff]

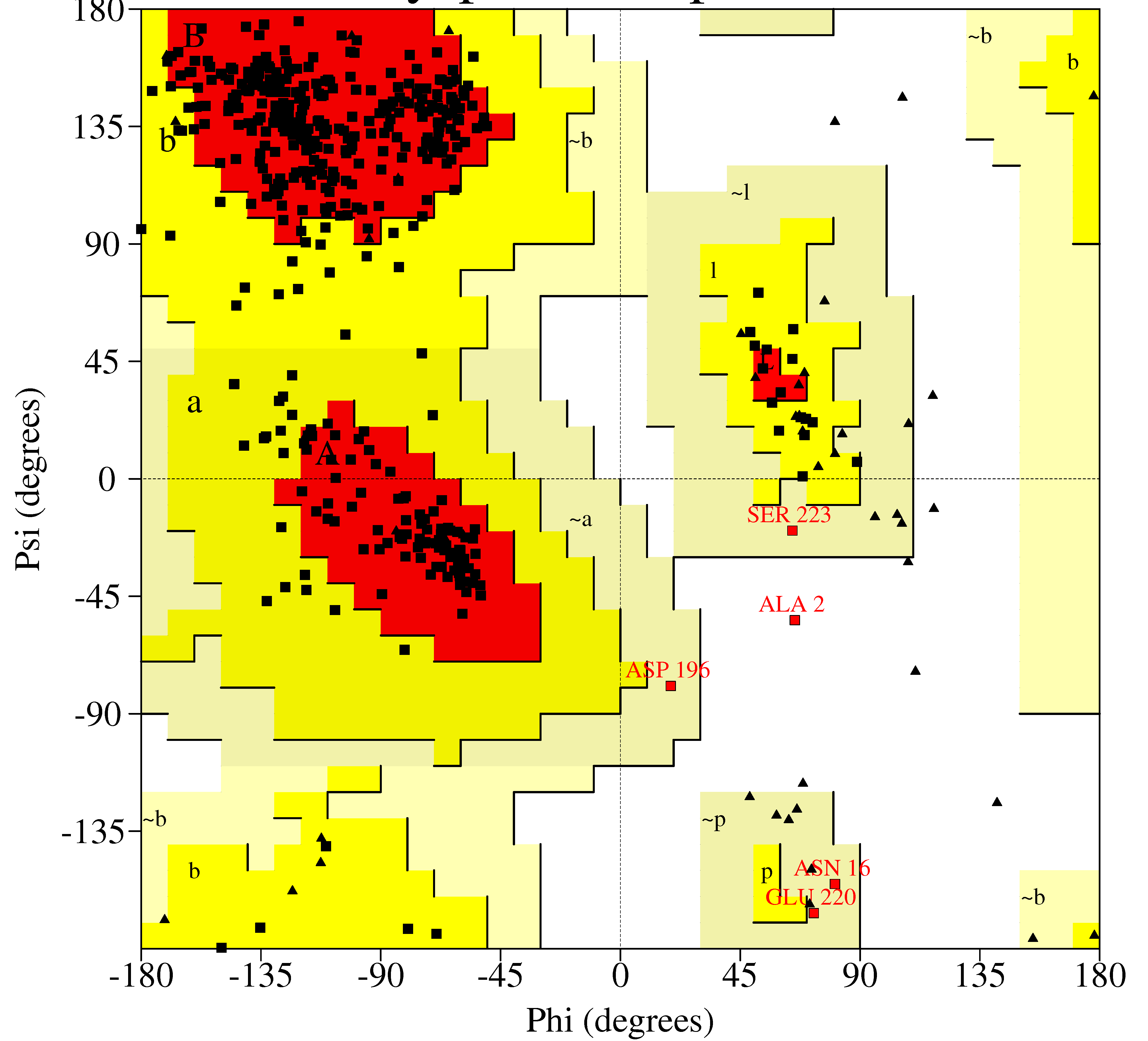

Supplement: S5 Dataset — The plots were generated through PROCHECK analysis. (ZIP) [file pone.0200607.s005.zip › Ramachandranplots/FOP4.tiff]

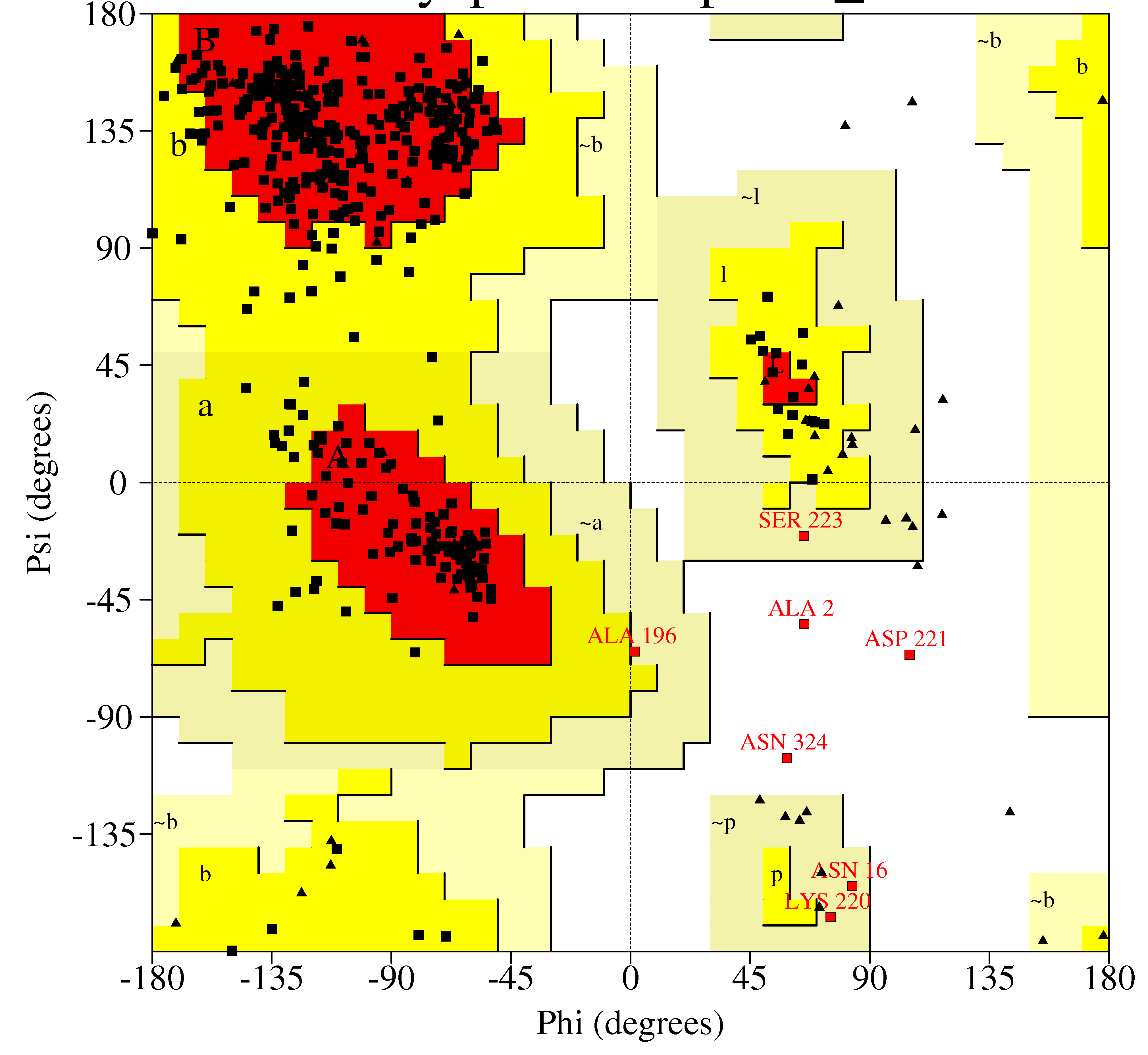

Supplement: S5 Dataset — The plots were generated through PROCHECK analysis. (ZIP) [file pone.0200607.s005.zip › Ramachandranplots/FOP5.tiff]

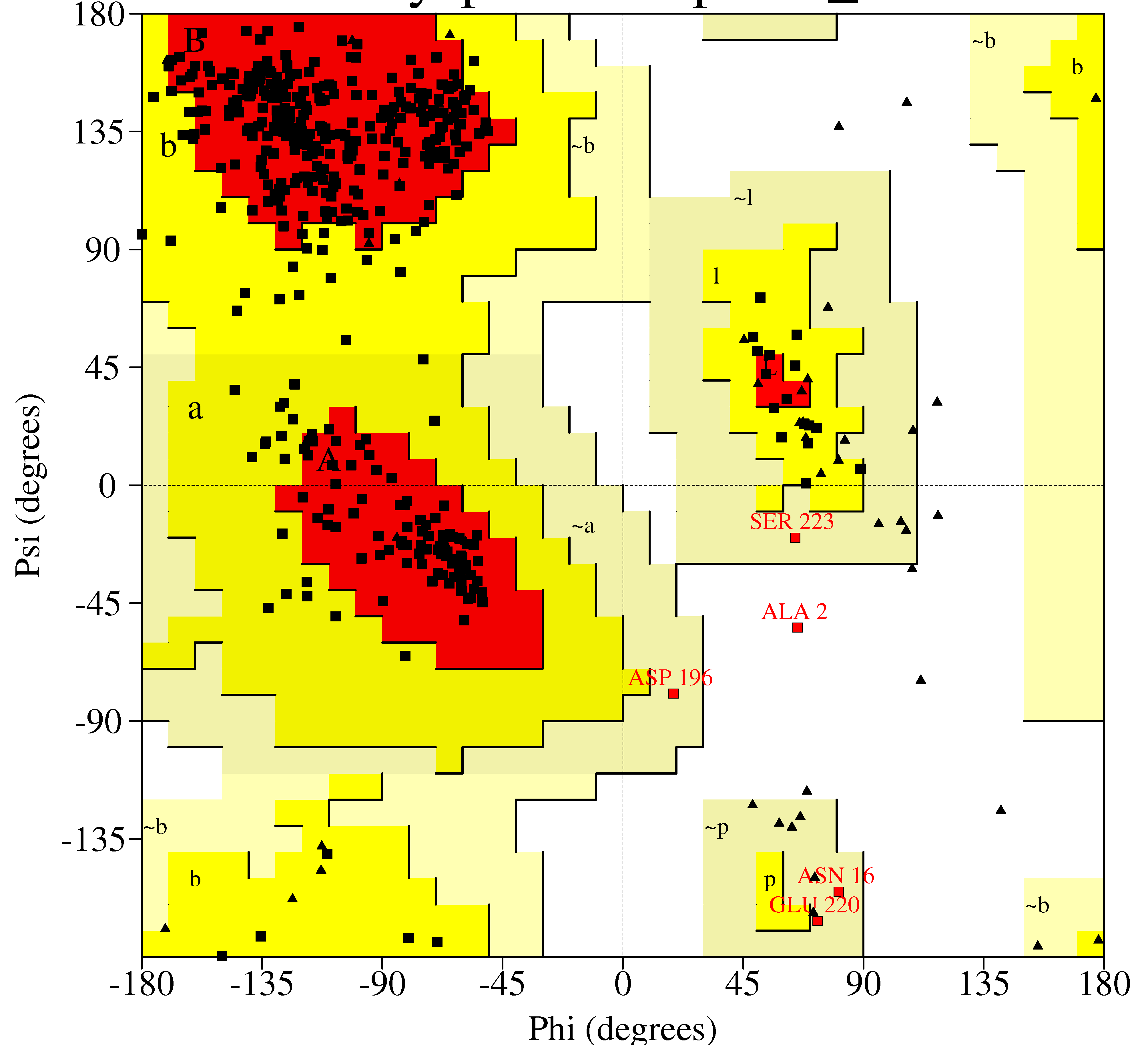

Supplement: S5 Dataset — The plots were generated through PROCHECK analysis. (ZIP) [file pone.0200607.s005.zip › Ramachandranplots/FOP6.tiff]

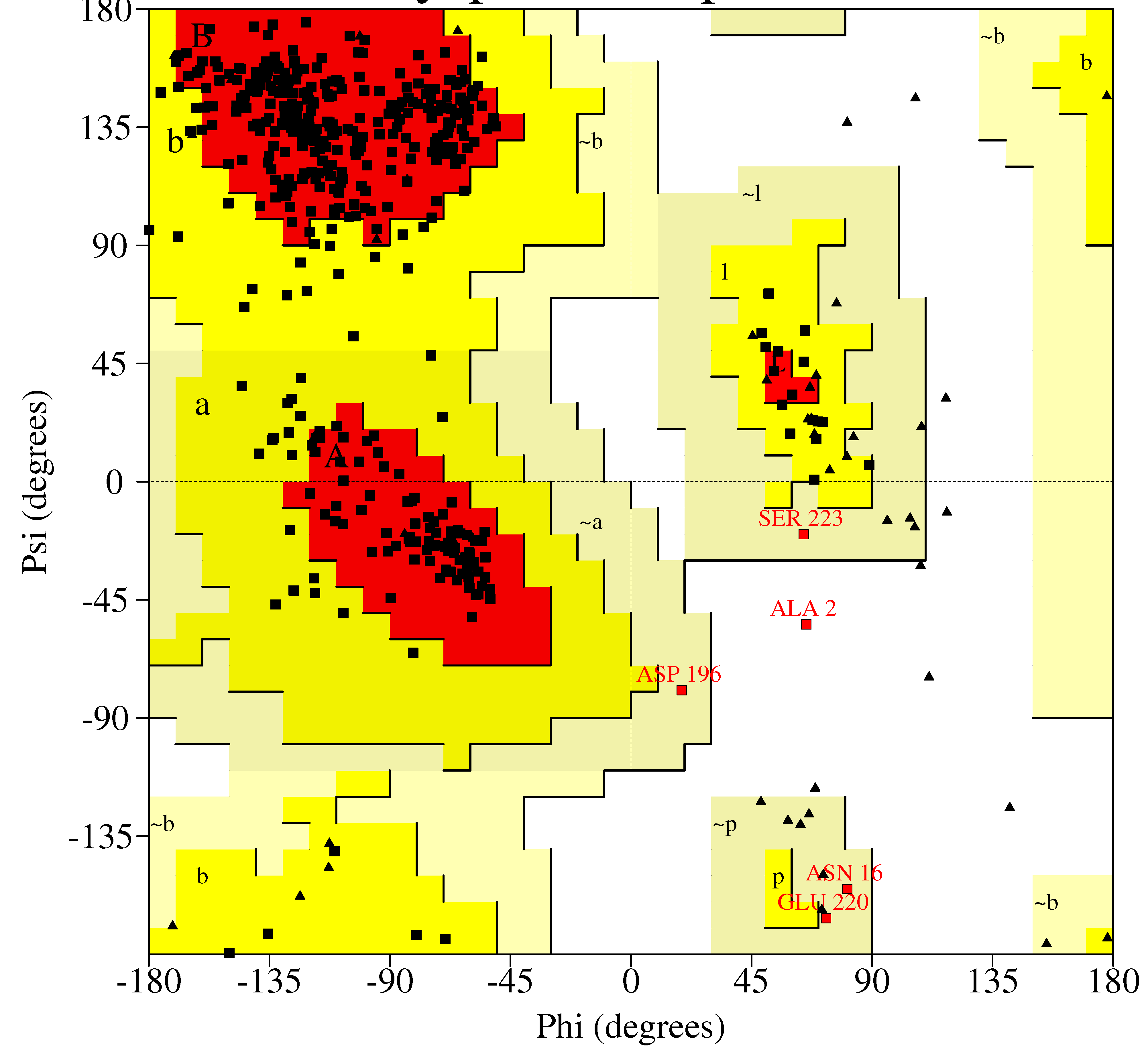

Supplement: S5 Dataset — The plots were generated through PROCHECK analysis. (ZIP) [file pone.0200607.s005.zip › Ramachandranplots/FOP7.tiff]

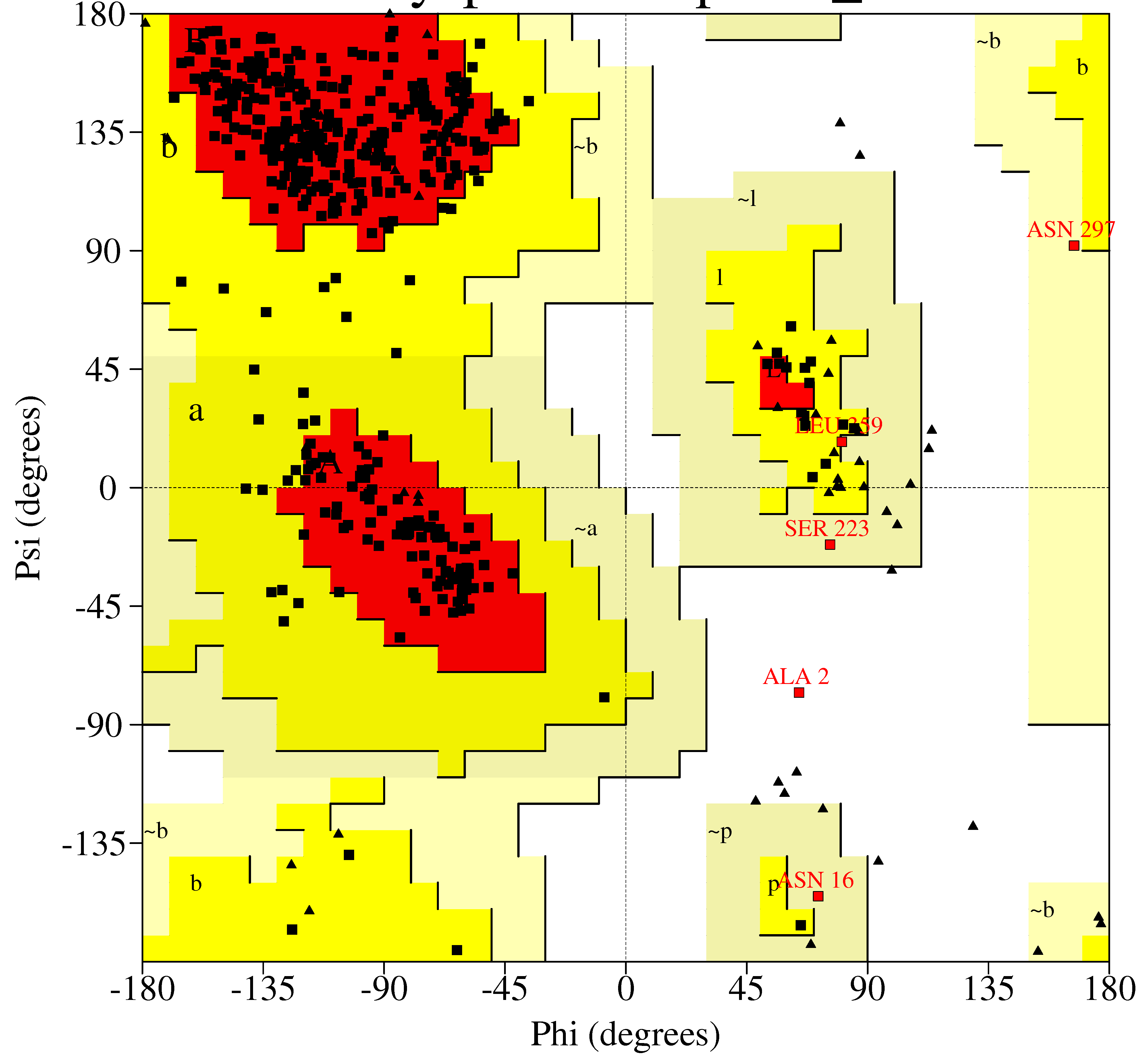

Supplement: S5 Dataset — The plots were generated through PROCHECK analysis. (ZIP) [file pone.0200607.s005.zip › Ramachandranplots/FOP8.tiff]

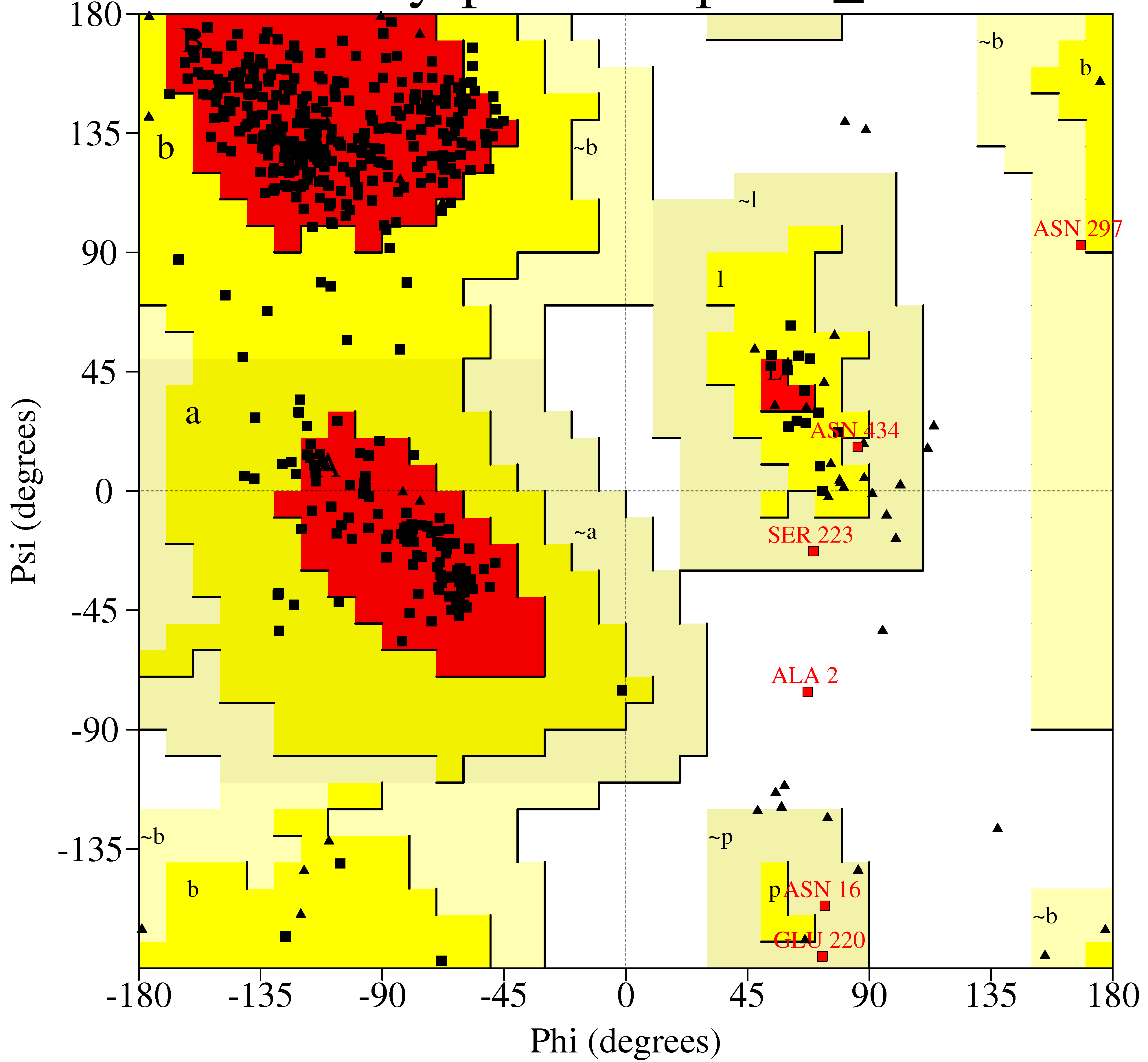

Supplement: S5 Dataset — The plots were generated through PROCHECK analysis. (ZIP) [file pone.0200607.s005.zip › Ramachandranplots/FOP9.tiff]

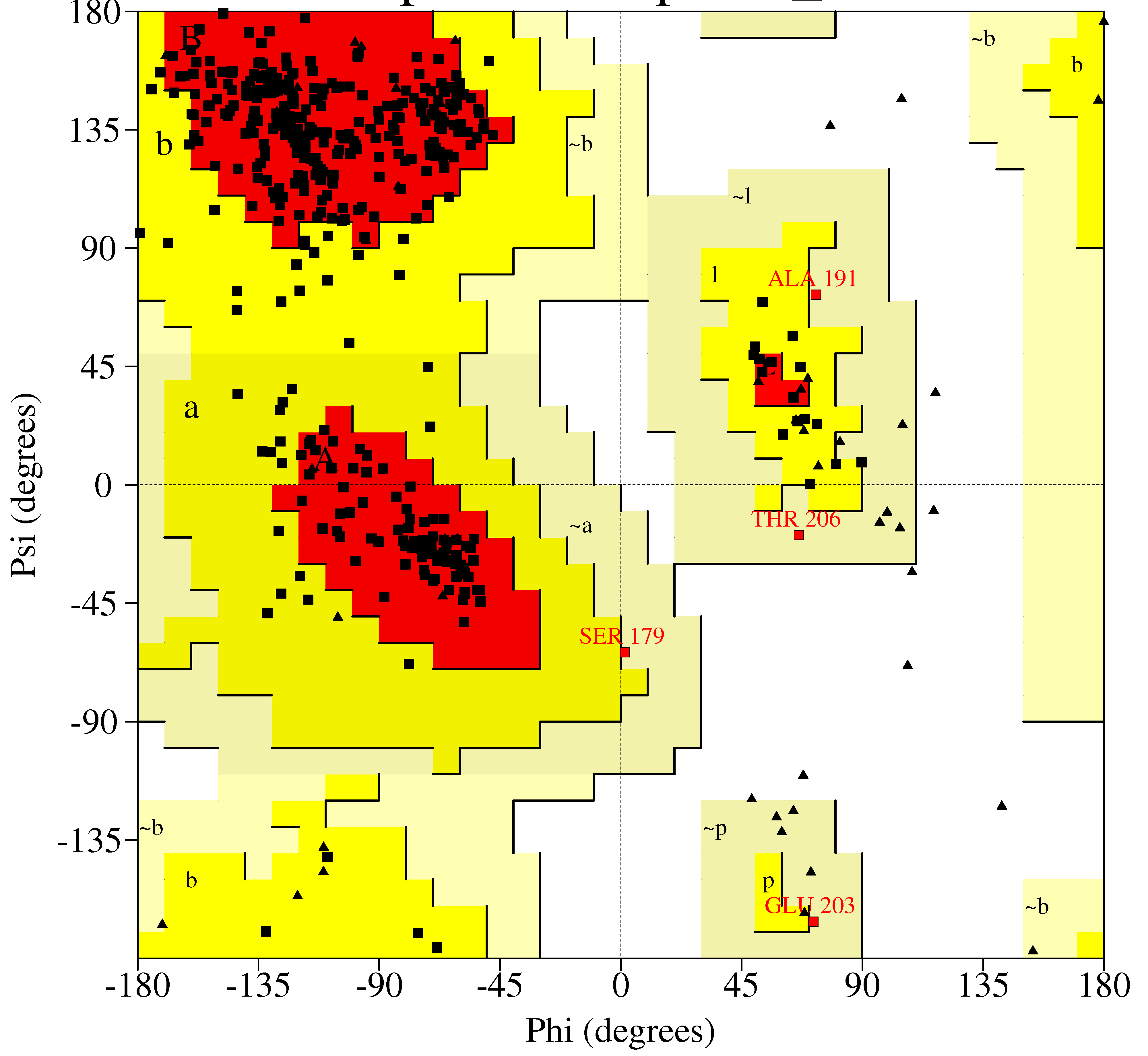

Supplement: S5 Dataset — The plots were generated through PROCHECK analysis. (ZIP) [file pone.0200607.s005.zip › Ramachandranplots/MPP1.tiff]

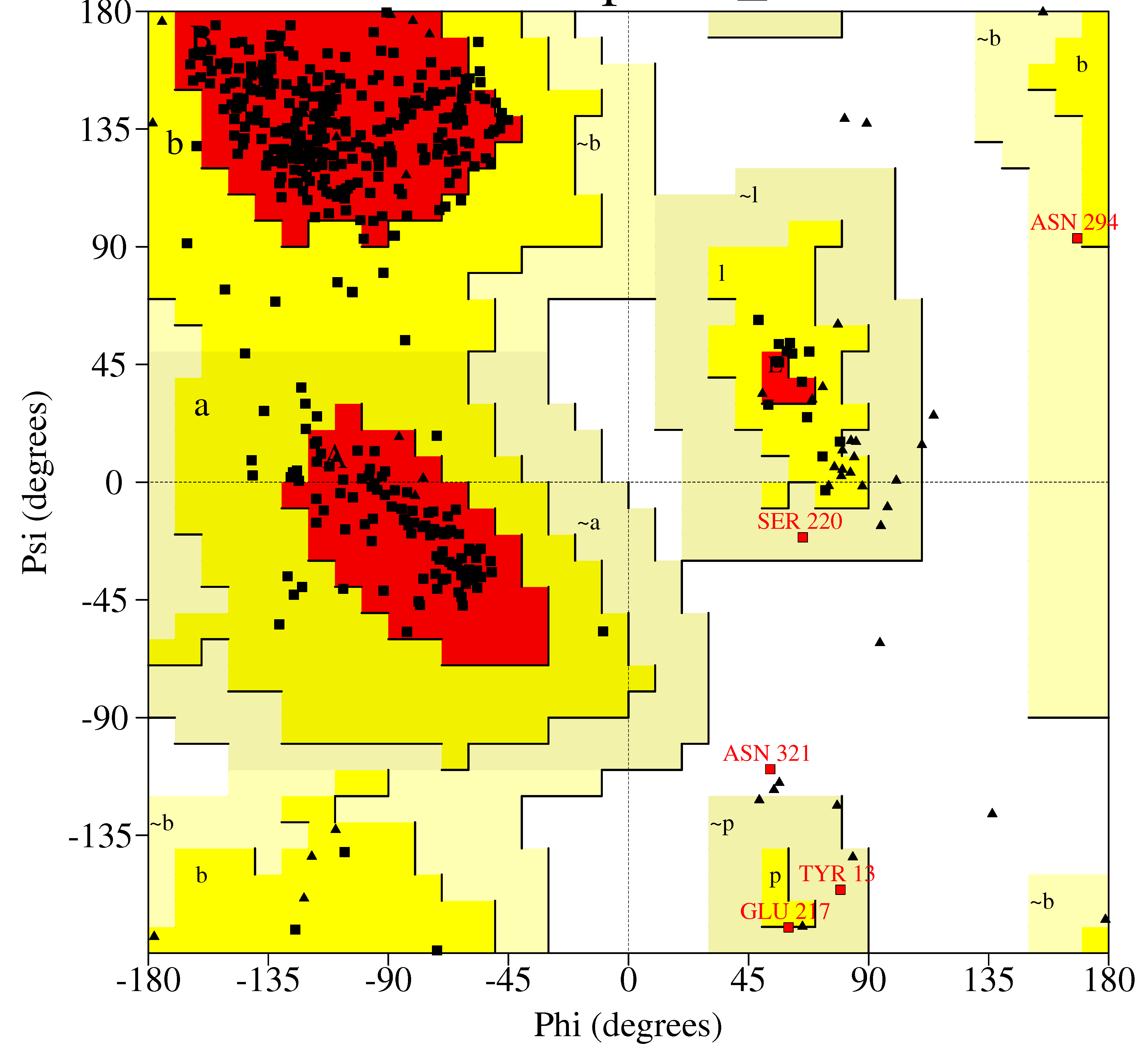

Supplement: S5 Dataset — The plots were generated through PROCHECK analysis. (ZIP) [file pone.0200607.s005.zip › Ramachandranplots/OMP1.tiff]

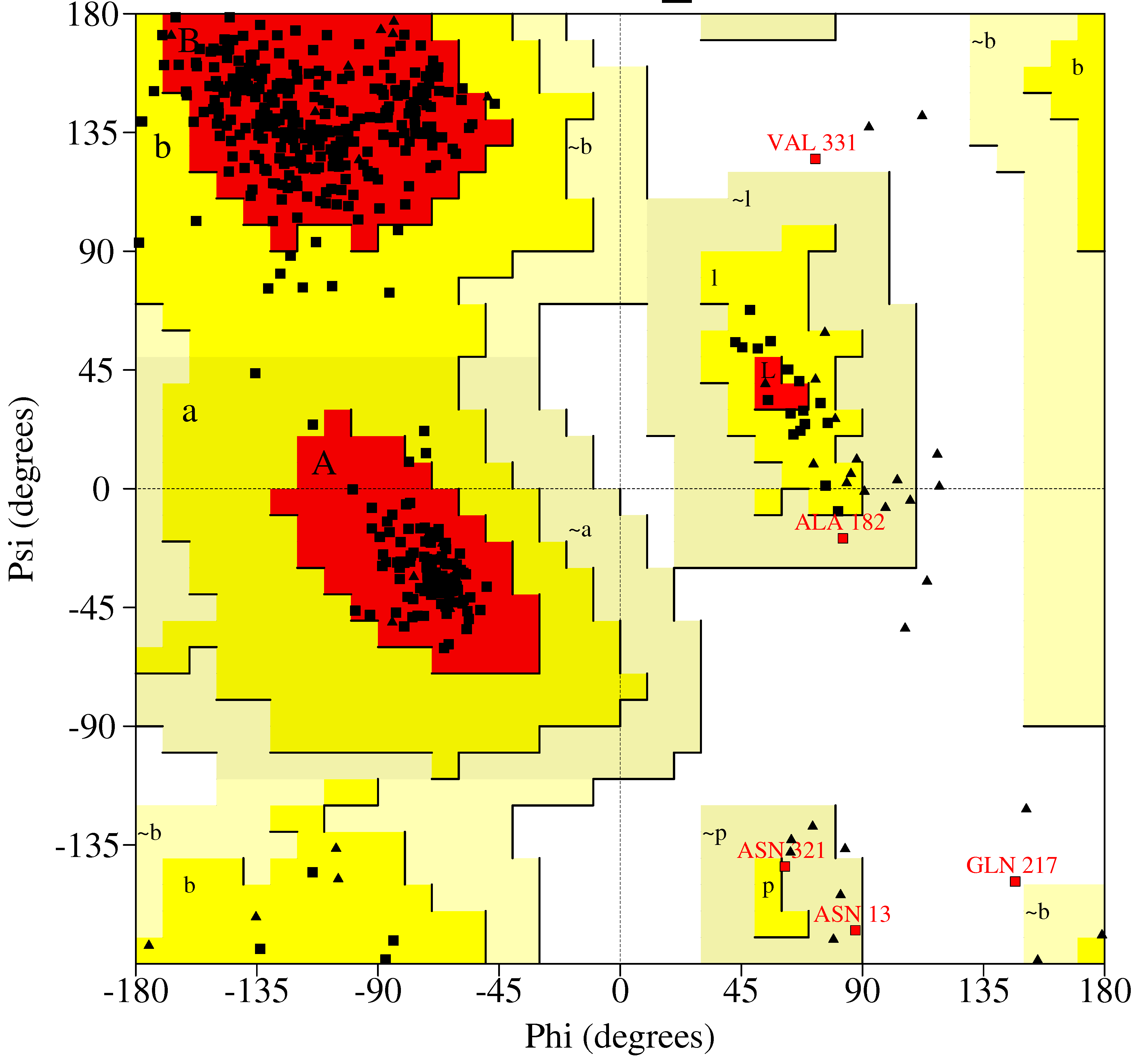

Supplement: S5 Dataset — The plots were generated through PROCHECK analysis. (ZIP) [file pone.0200607.s005.zip › Ramachandranplots/PBP1.tiff]

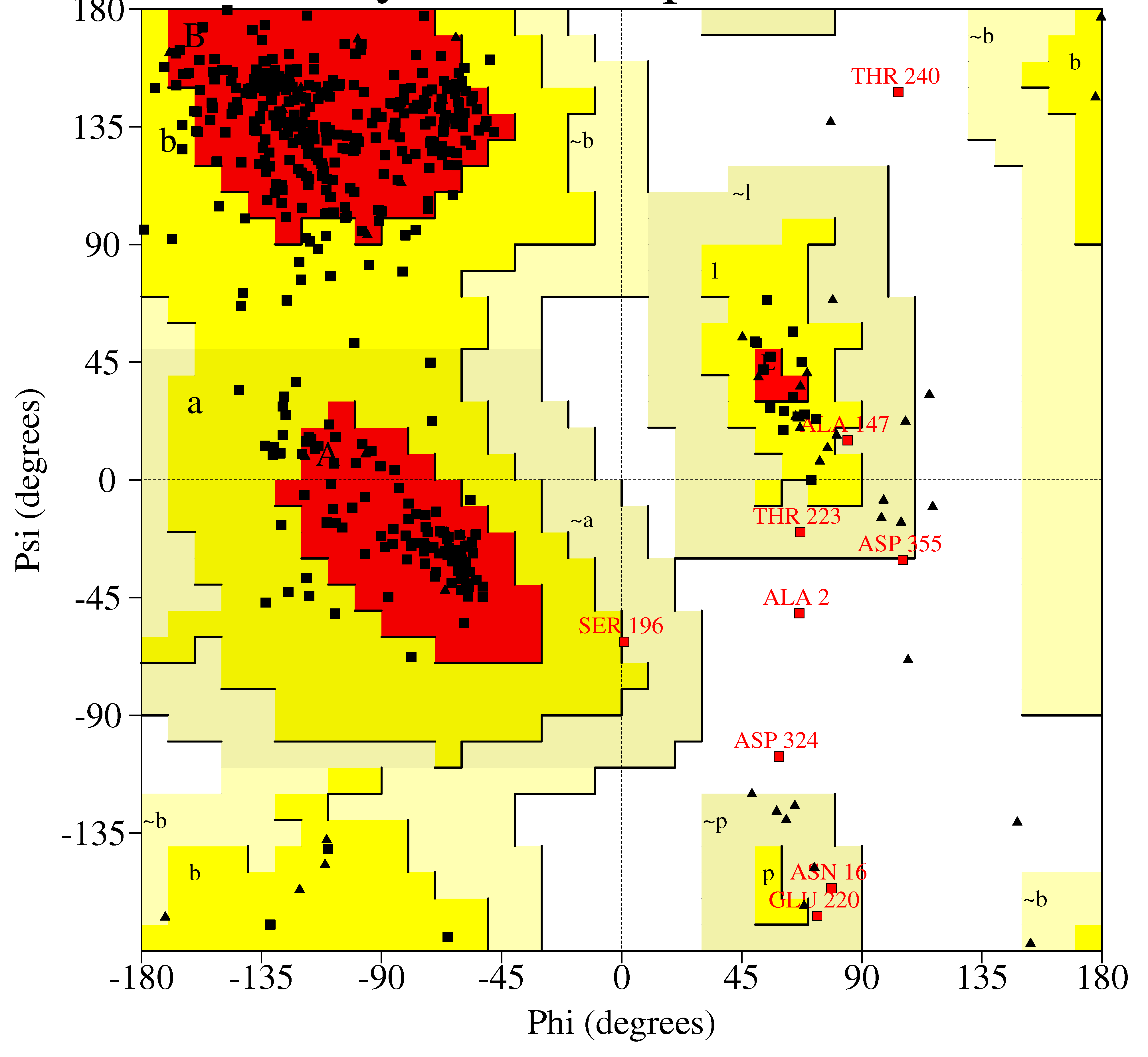

Supplement: S5 Dataset — The plots were generated through PROCHECK analysis. (ZIP) [file pone.0200607.s005.zip › Ramachandranplots/PCP1.tiff]

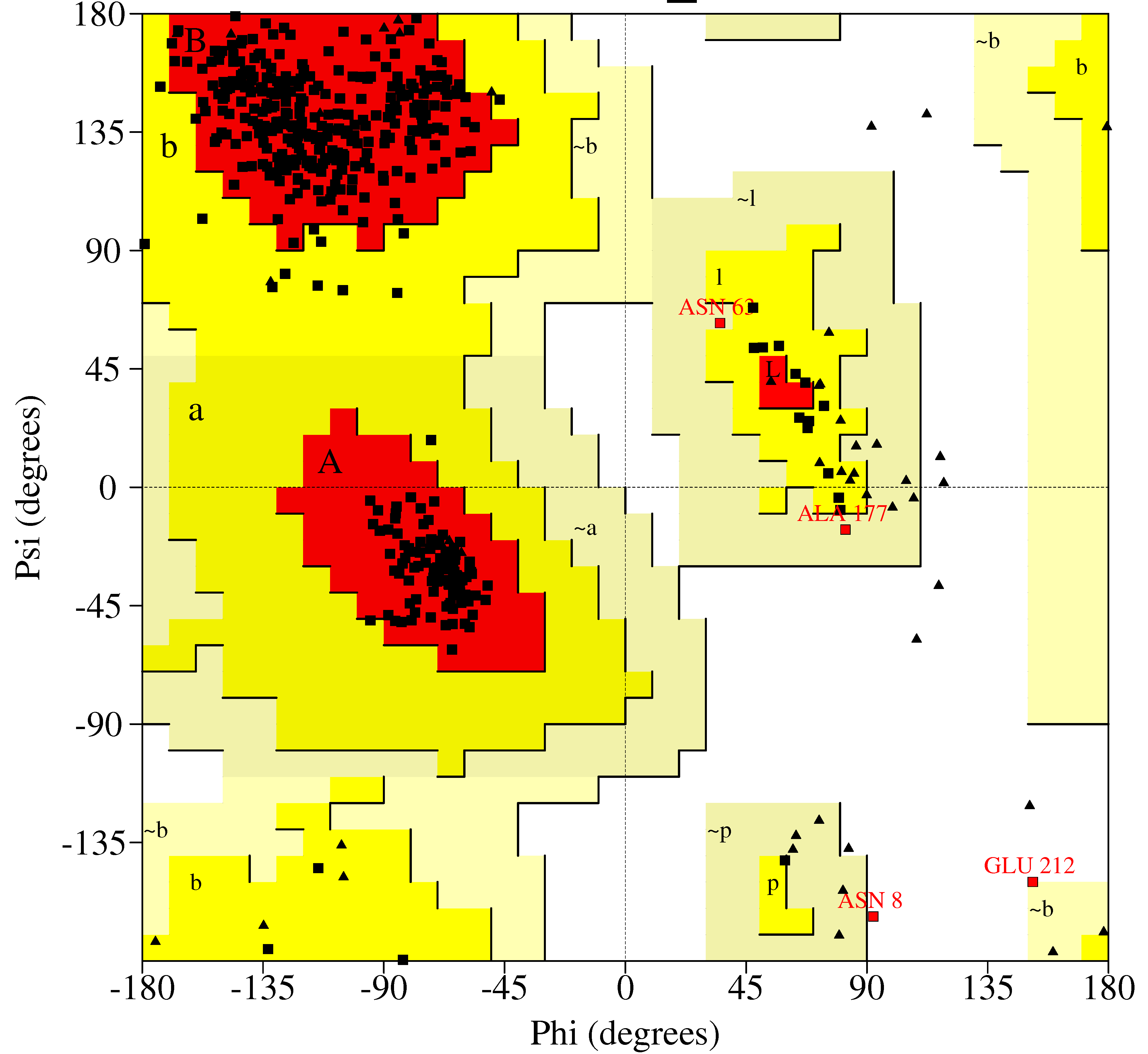

Supplement: S5 Dataset — The plots were generated through PROCHECK analysis. (ZIP) [file pone.0200607.s005.zip › Ramachandranplots/PGP1.tiff]

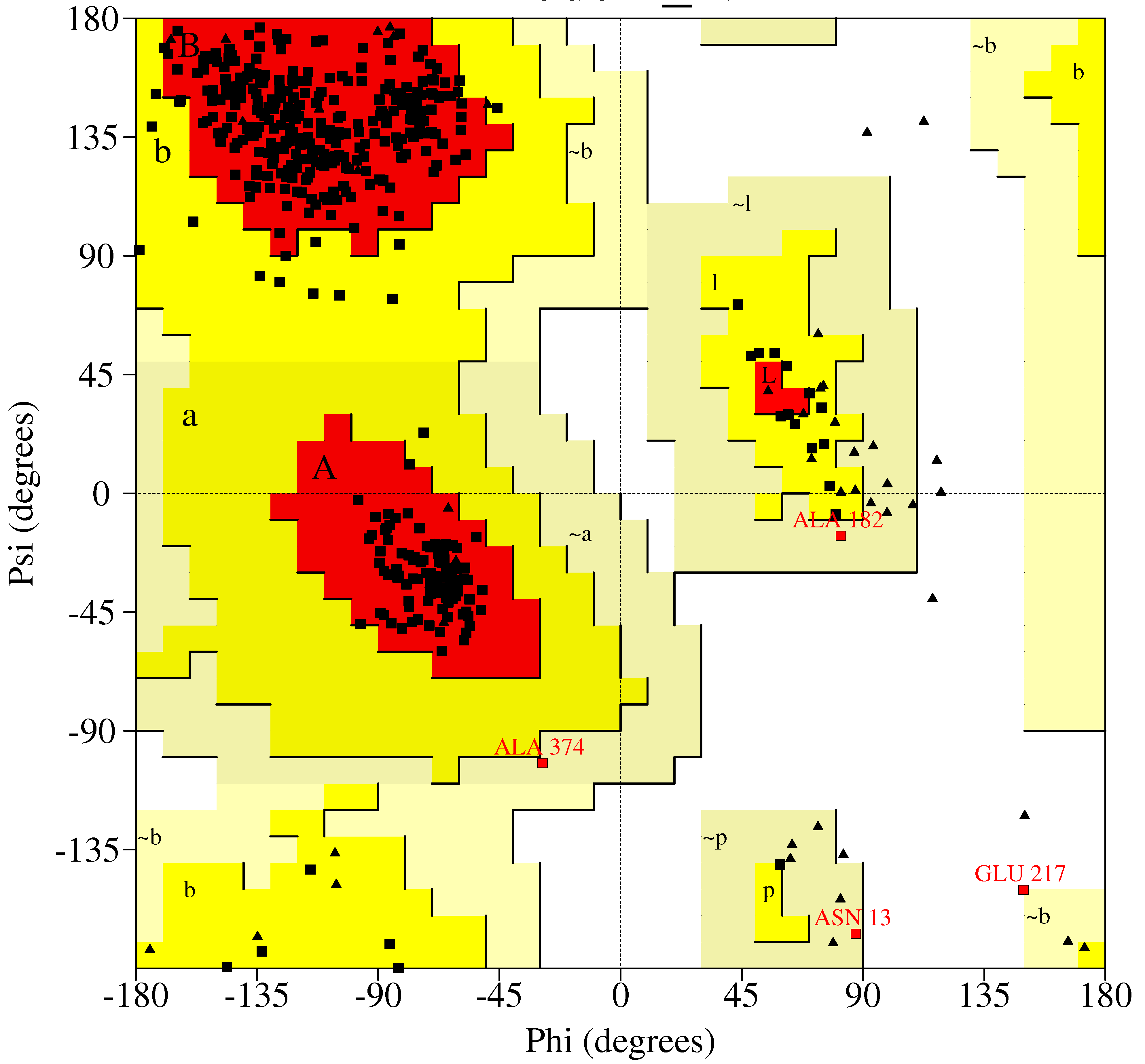

Supplement: S5 Dataset — The plots were generated through PROCHECK analysis. (ZIP) [file pone.0200607.s005.zip › Ramachandranplots/PGP2.tiff]

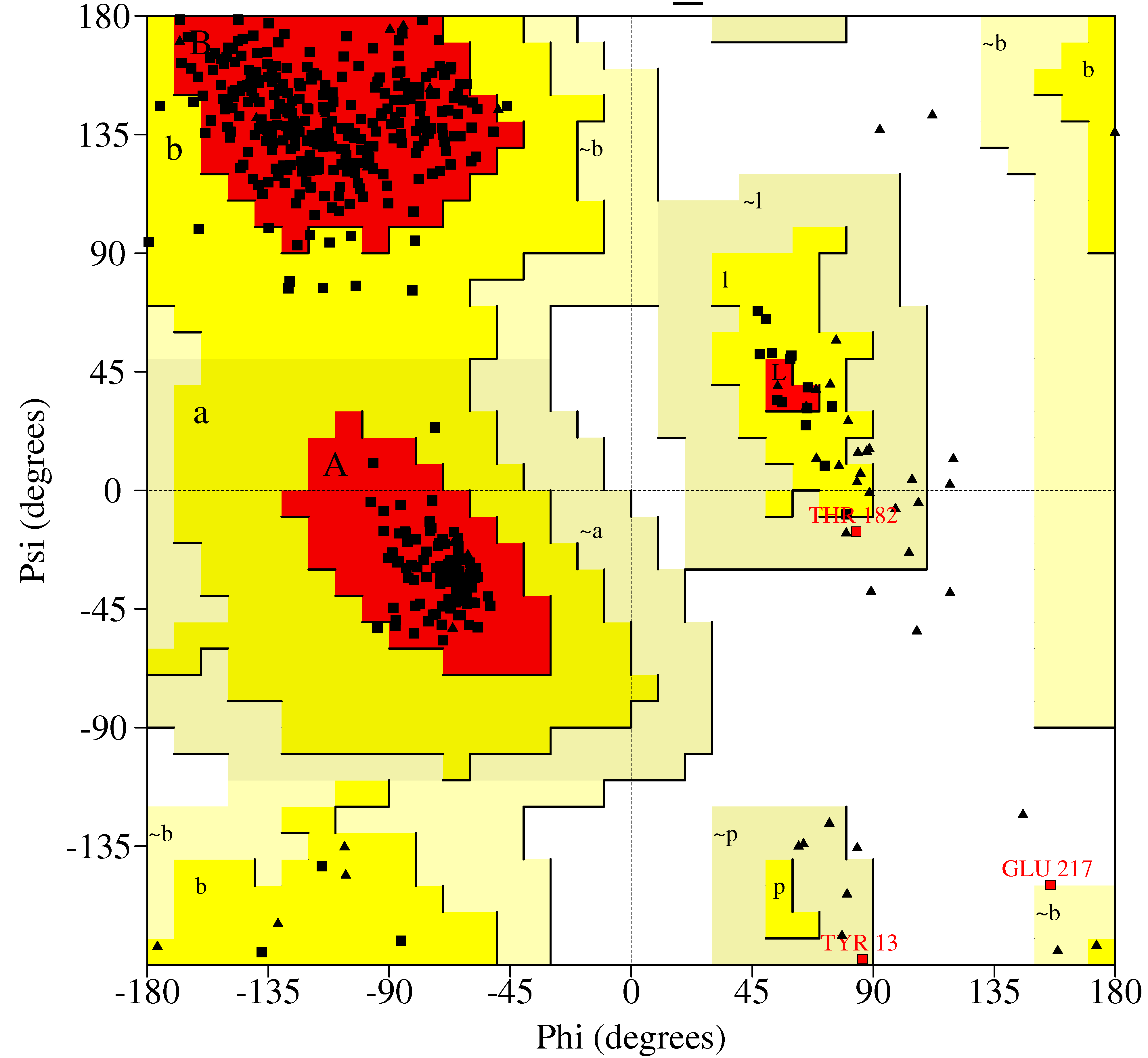

Supplement: S5 Dataset — The plots were generated through PROCHECK analysis. (ZIP) [file pone.0200607.s005.zip › Ramachandranplots/PGP3.tiff]

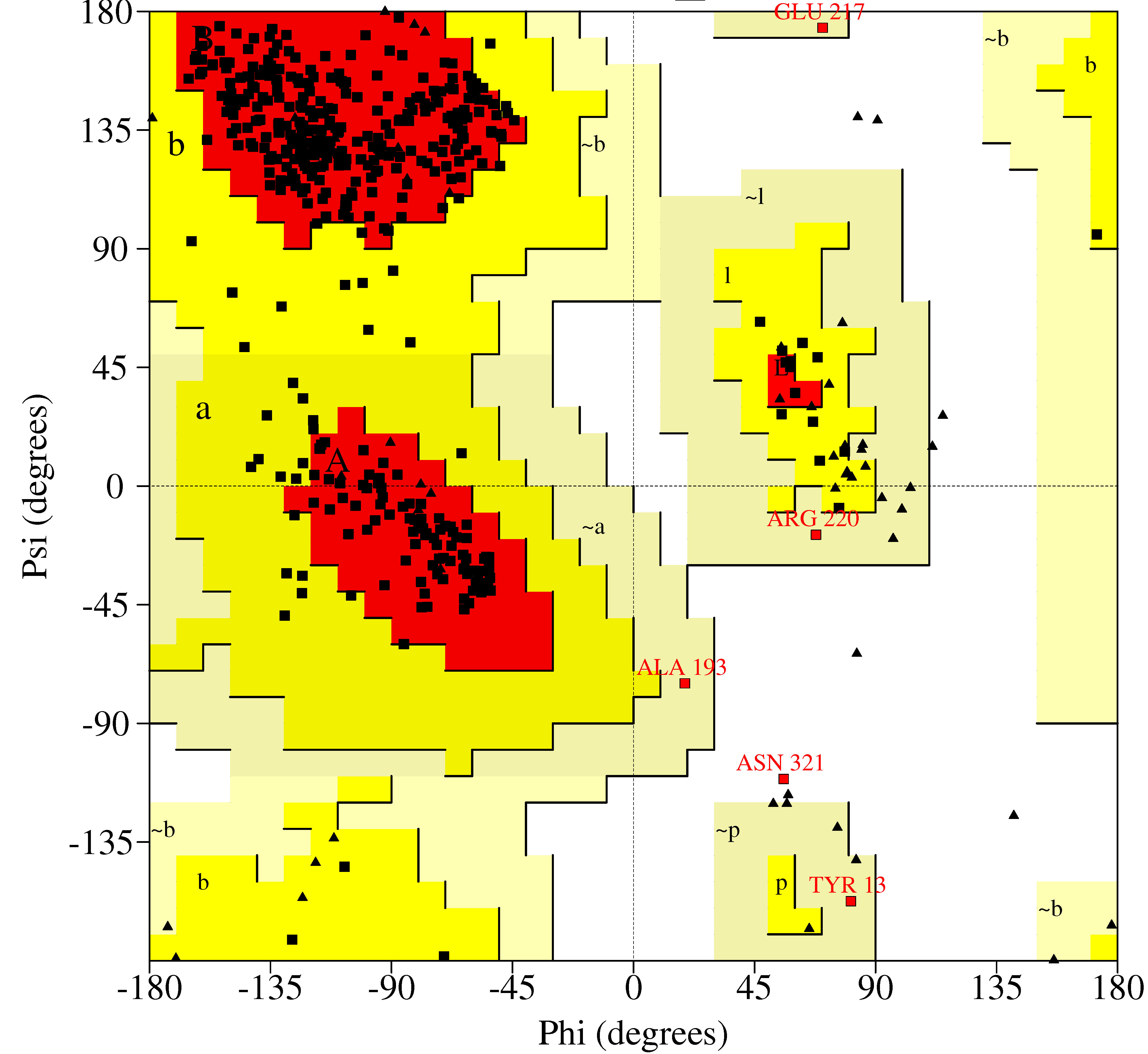

Supplement: S5 Dataset — The plots were generated through PROCHECK analysis. (ZIP) [file pone.0200607.s005.zip › Ramachandranplots/PGP4.tiff]

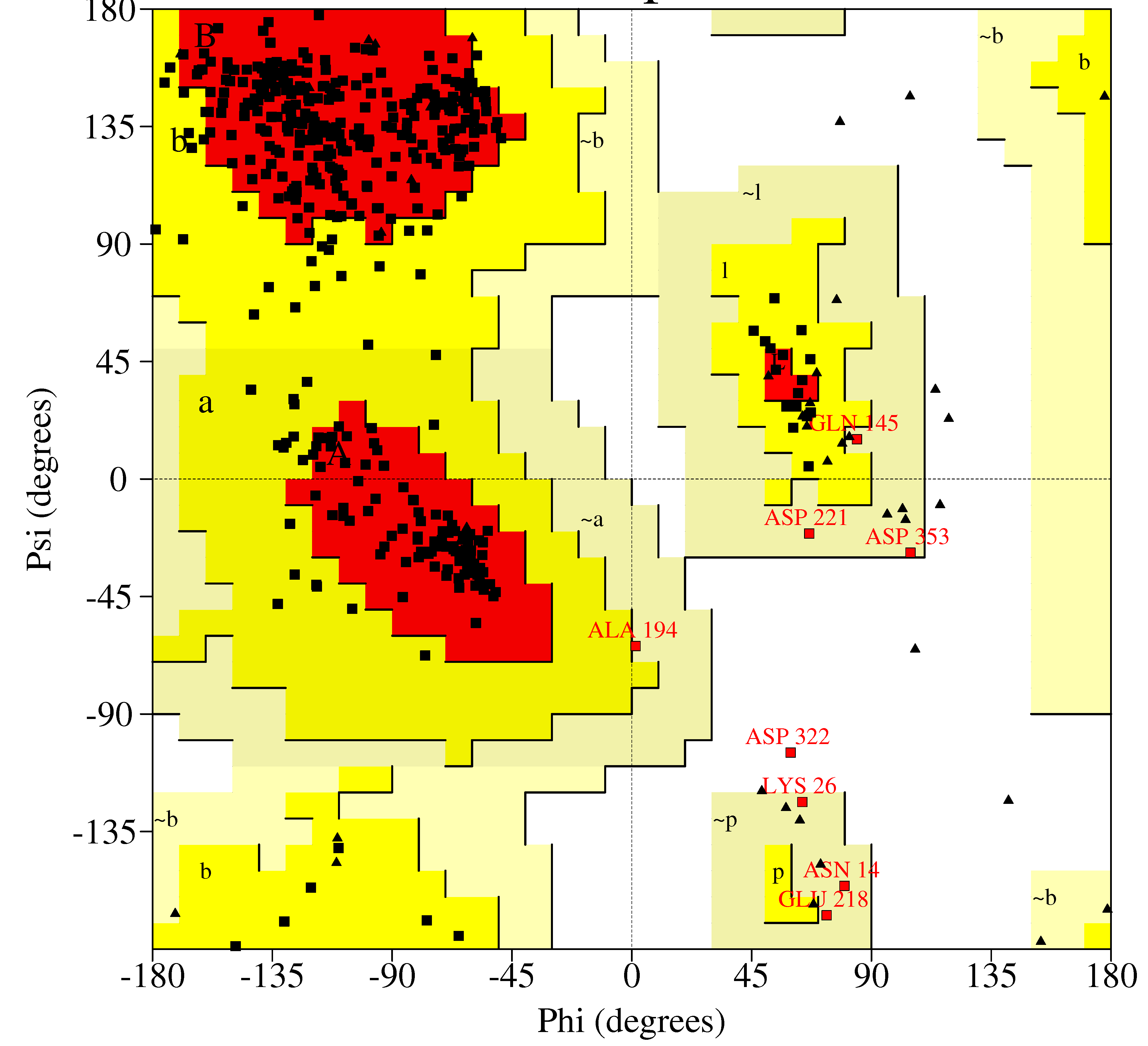

Supplement: S5 Dataset — The plots were generated through PROCHECK analysis. (ZIP) [file pone.0200607.s005.zip › Ramachandranplots/PNP1.tiff]

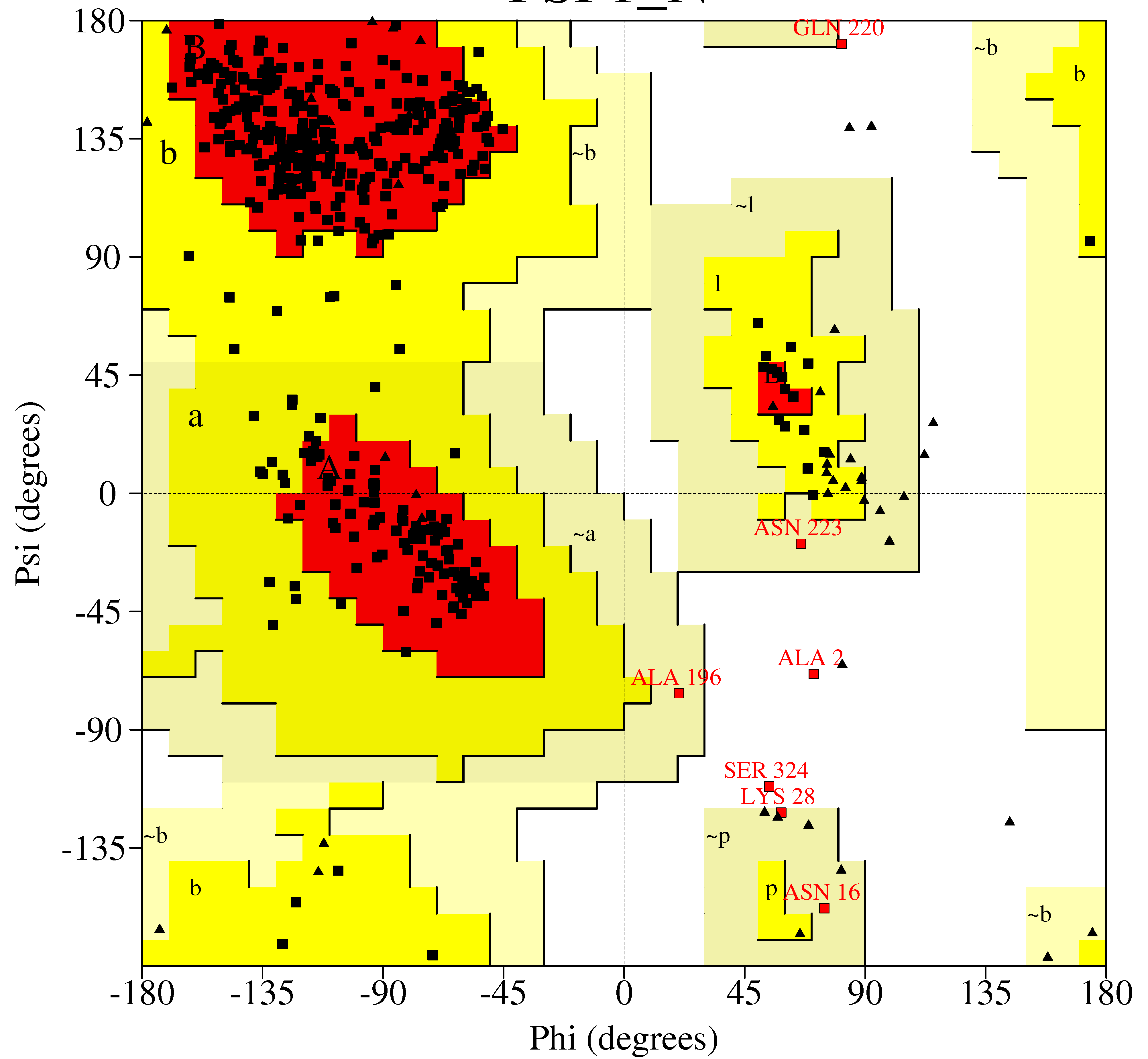

Supplement: S5 Dataset — The plots were generated through PROCHECK analysis. (ZIP) [file pone.0200607.s005.zip › Ramachandranplots/PSP1.tiff]

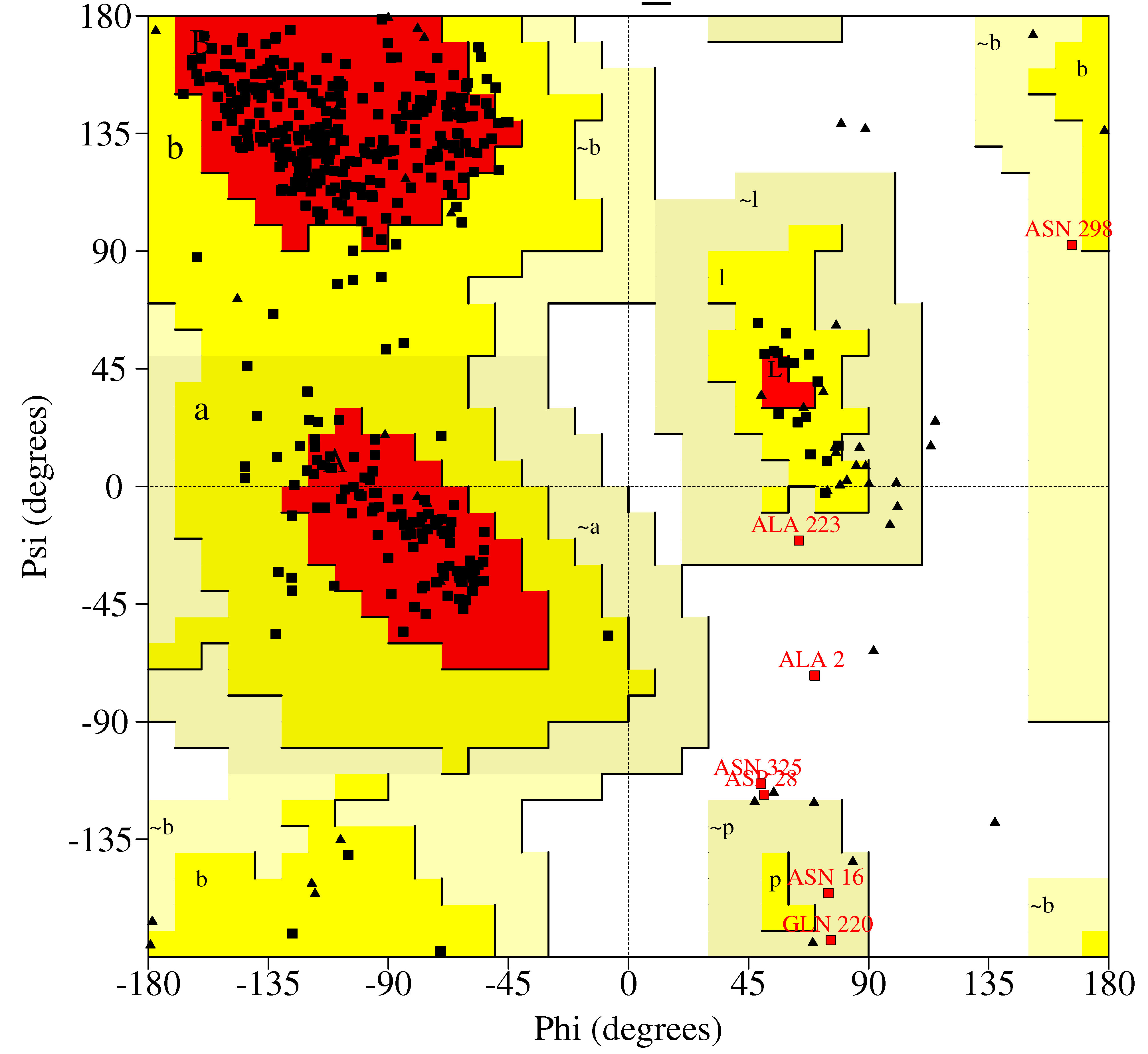

Supplement: S5 Dataset — The plots were generated through PROCHECK analysis. (ZIP) [file pone.0200607.s005.zip › Ramachandranplots/PSP2.tiff]

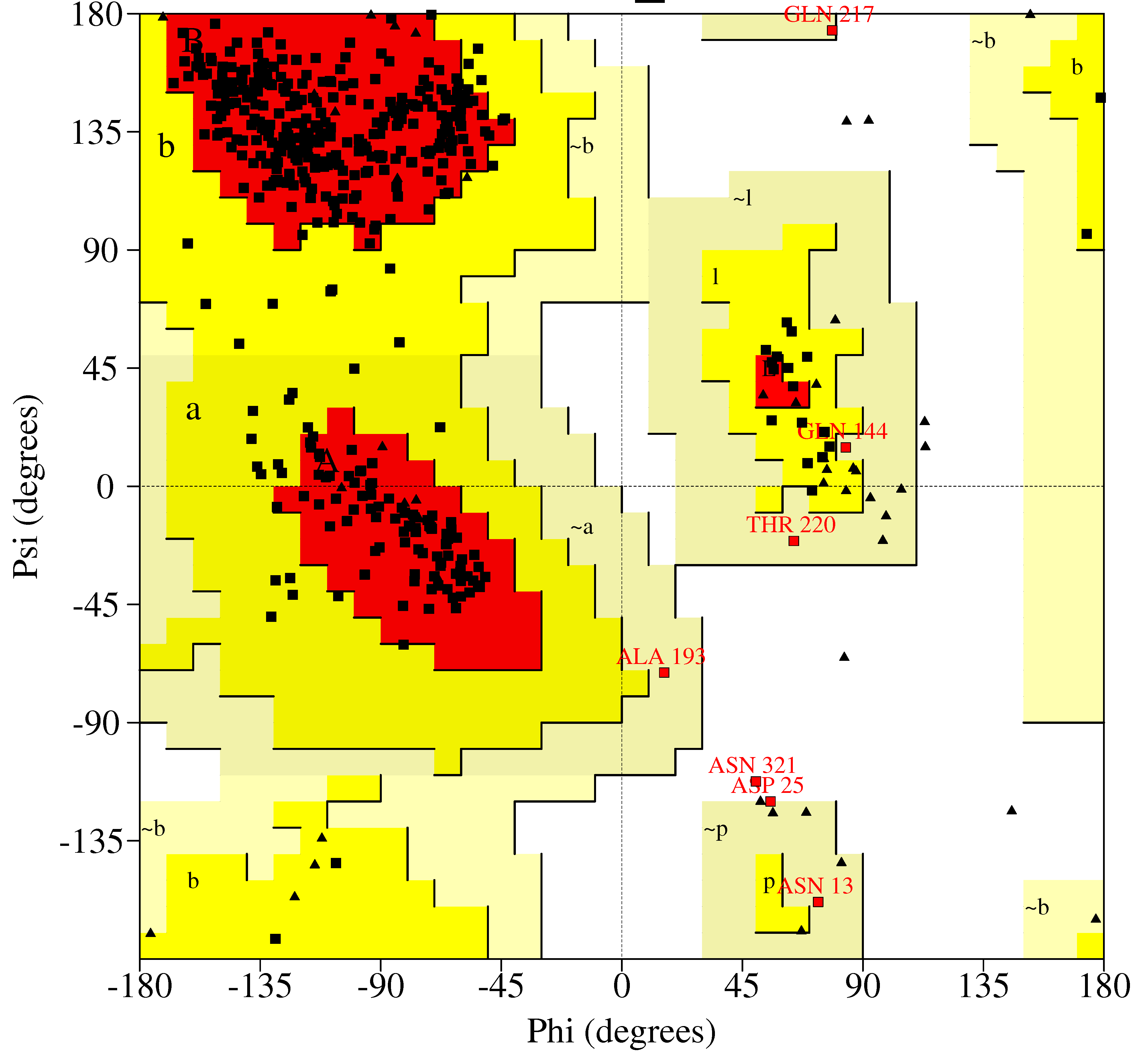

Supplement: S5 Dataset — The plots were generated through PROCHECK analysis. (ZIP) [file pone.0200607.s005.zip › Ramachandranplots/PSP3.tiff]

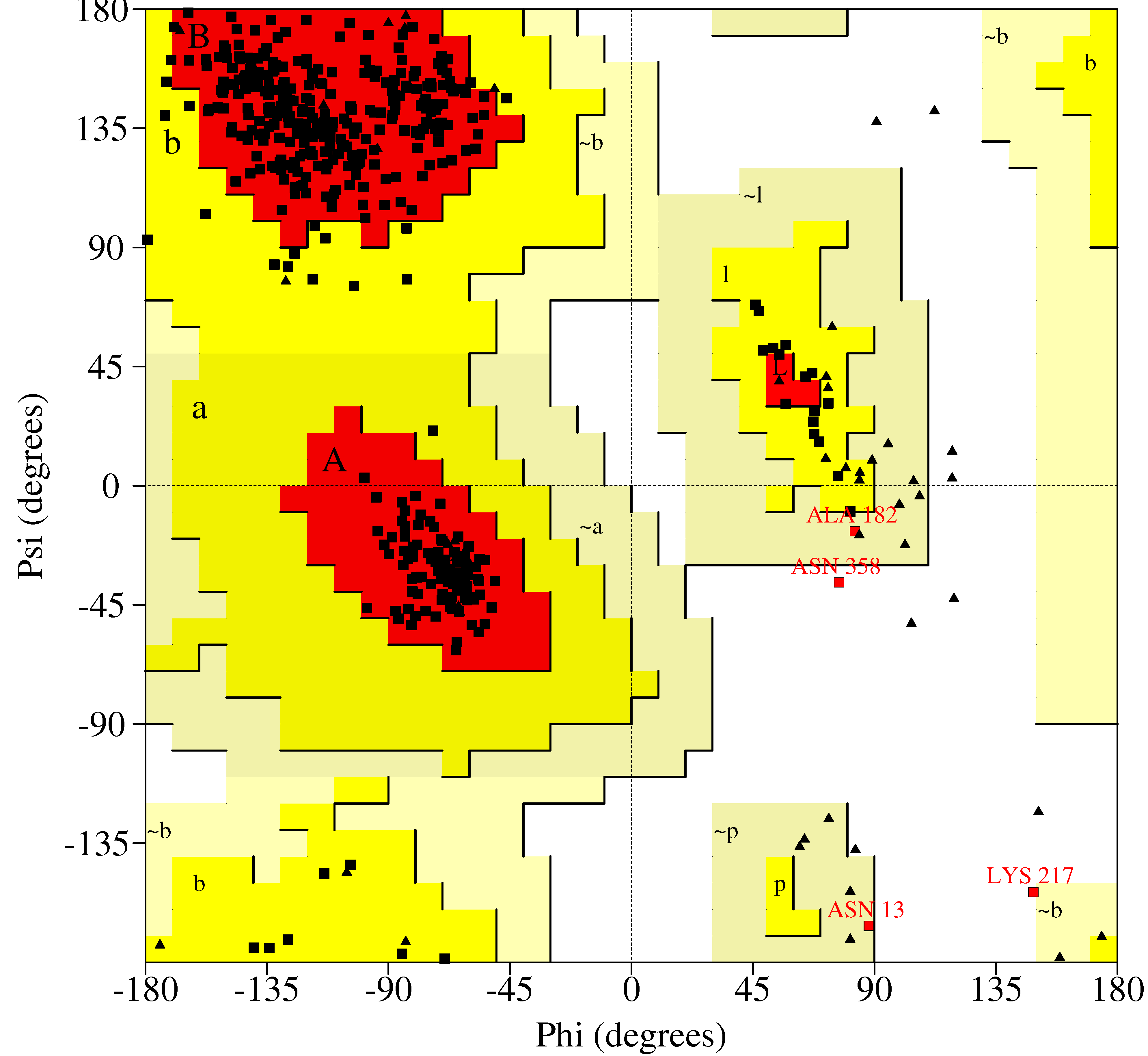

Supplement: S5 Dataset — The plots were generated through PROCHECK analysis. (ZIP) [file pone.0200607.s005.zip › Ramachandranplots/SCHP1.tiff]

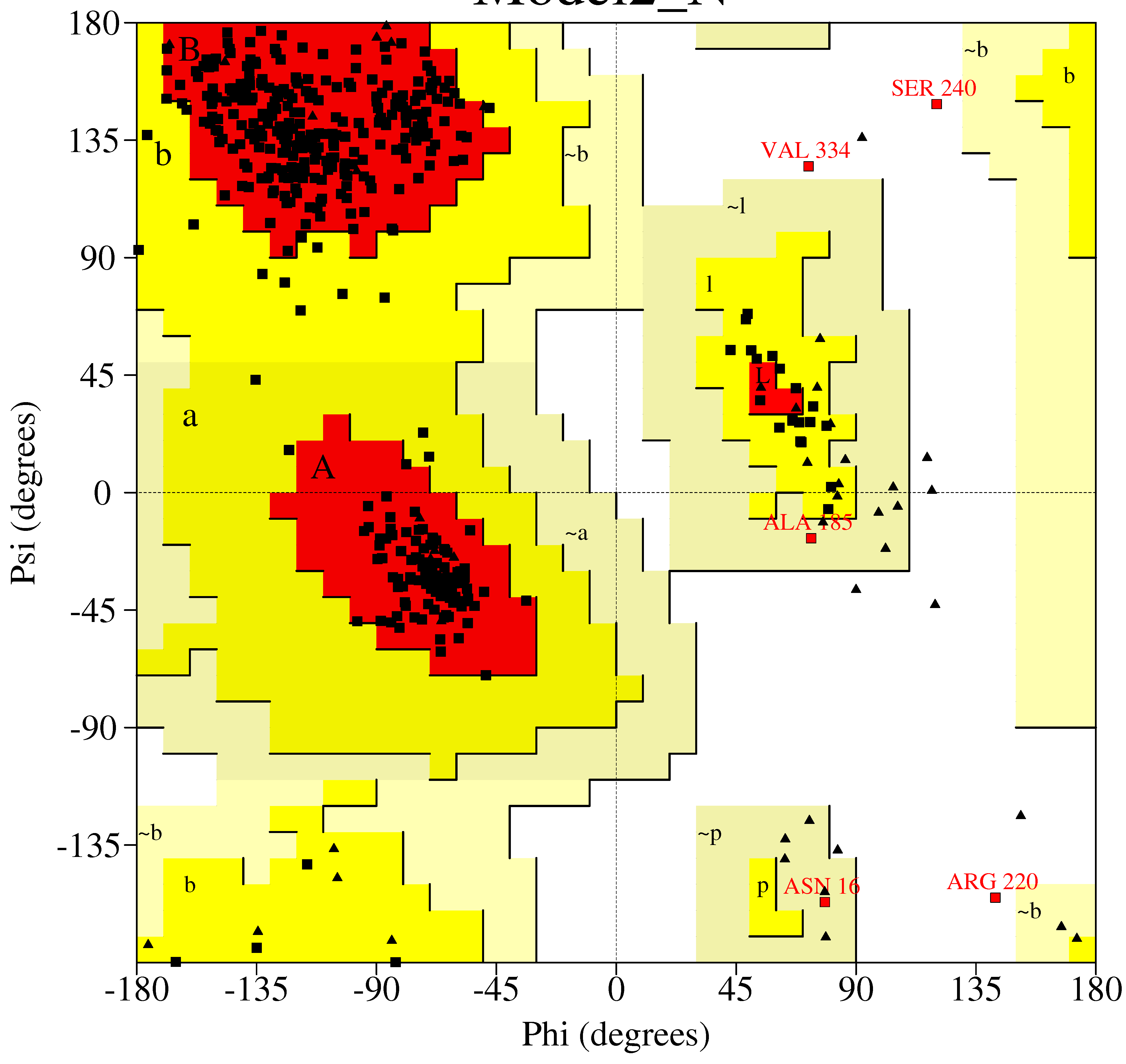

Supplement: S5 Dataset — The plots were generated through PROCHECK analysis. (ZIP) [file pone.0200607.s005.zip › Ramachandranplots/SCHP2.tiff]

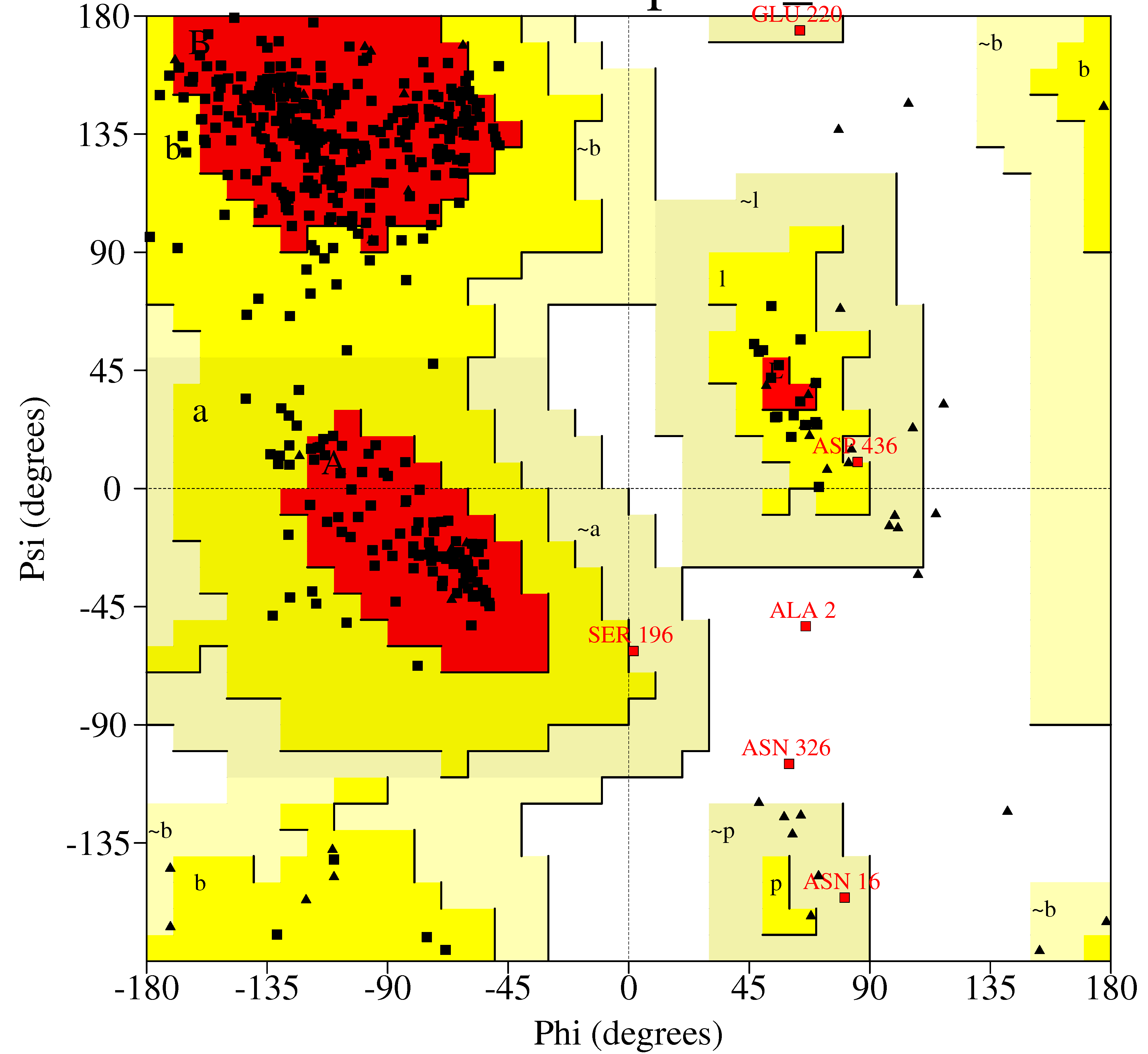

Supplement: S5 Dataset — The plots were generated through PROCHECK analysis. (ZIP) [file pone.0200607.s005.zip › Ramachandranplots/SCP1.tiff]

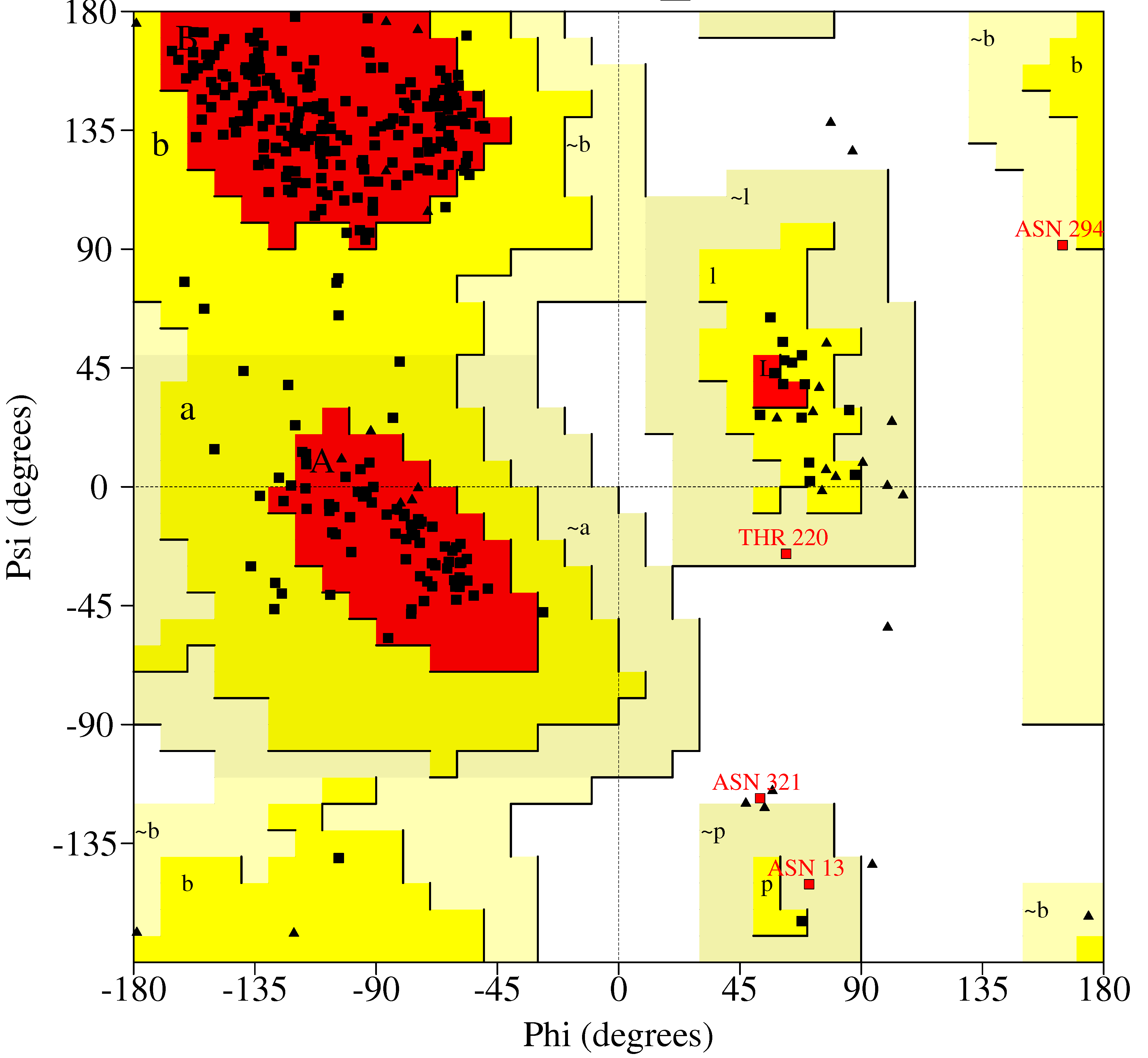

Supplement: S5 Dataset — The plots were generated through PROCHECK analysis. (ZIP) [file pone.0200607.s005.zip › Ramachandranplots/SCP18.tiff]

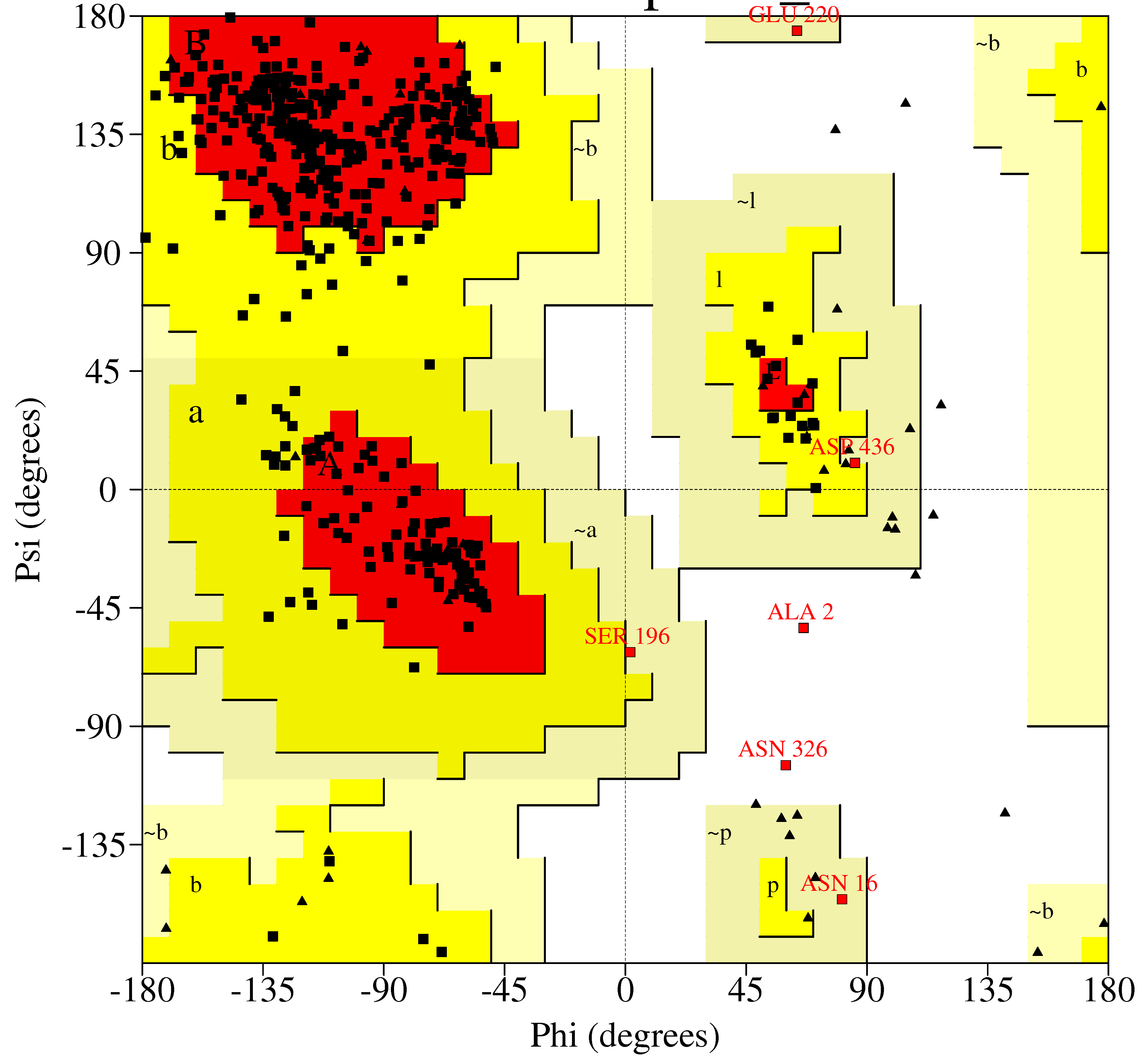

Supplement: S5 Dataset — The plots were generated through PROCHECK analysis. (ZIP) [file pone.0200607.s005.zip › Ramachandranplots/SCP2.tiff]

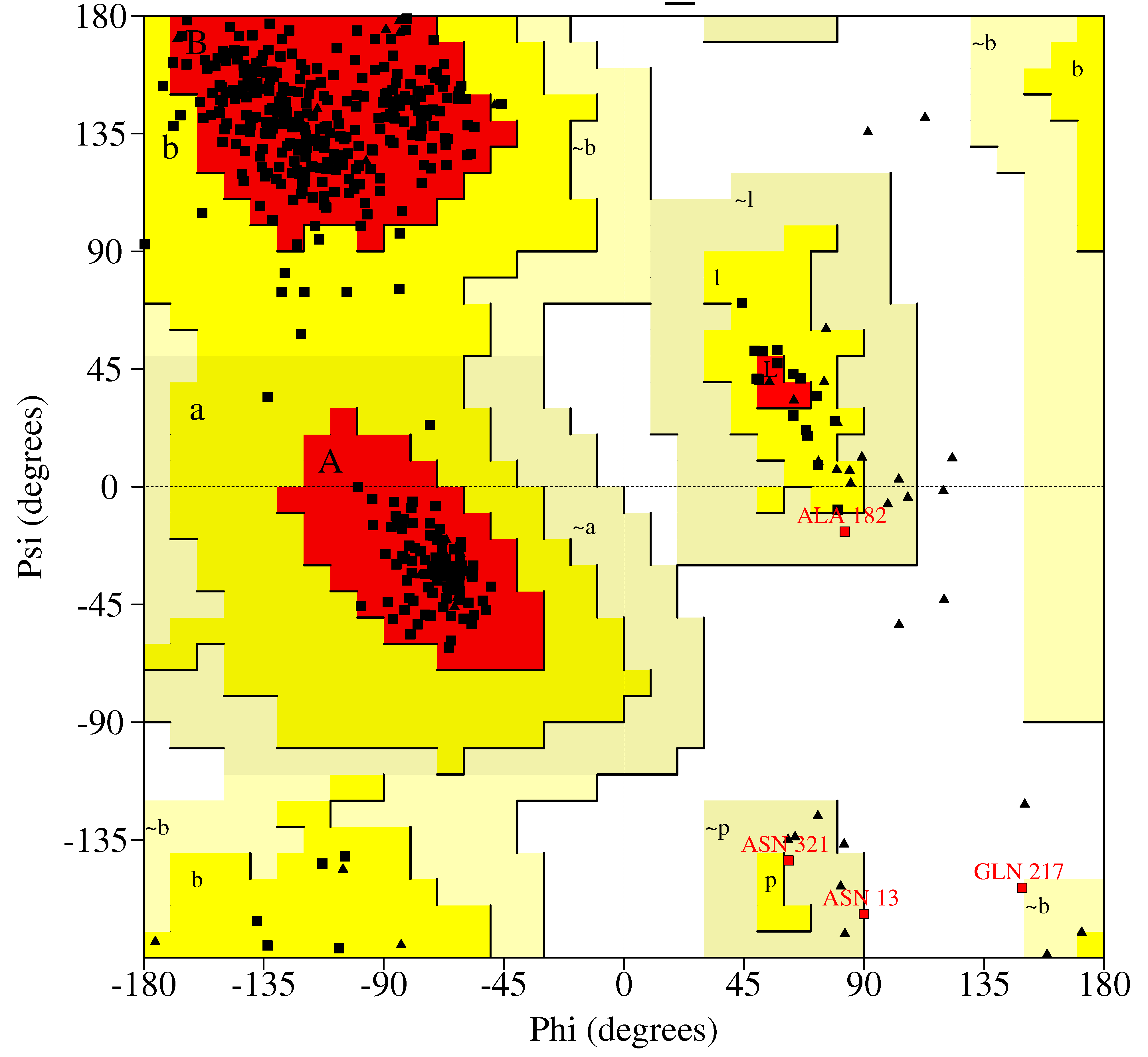

Supplement: S5 Dataset — The plots were generated through PROCHECK analysis. (ZIP) [file pone.0200607.s005.zip › Ramachandranplots/SCP3.tiff]

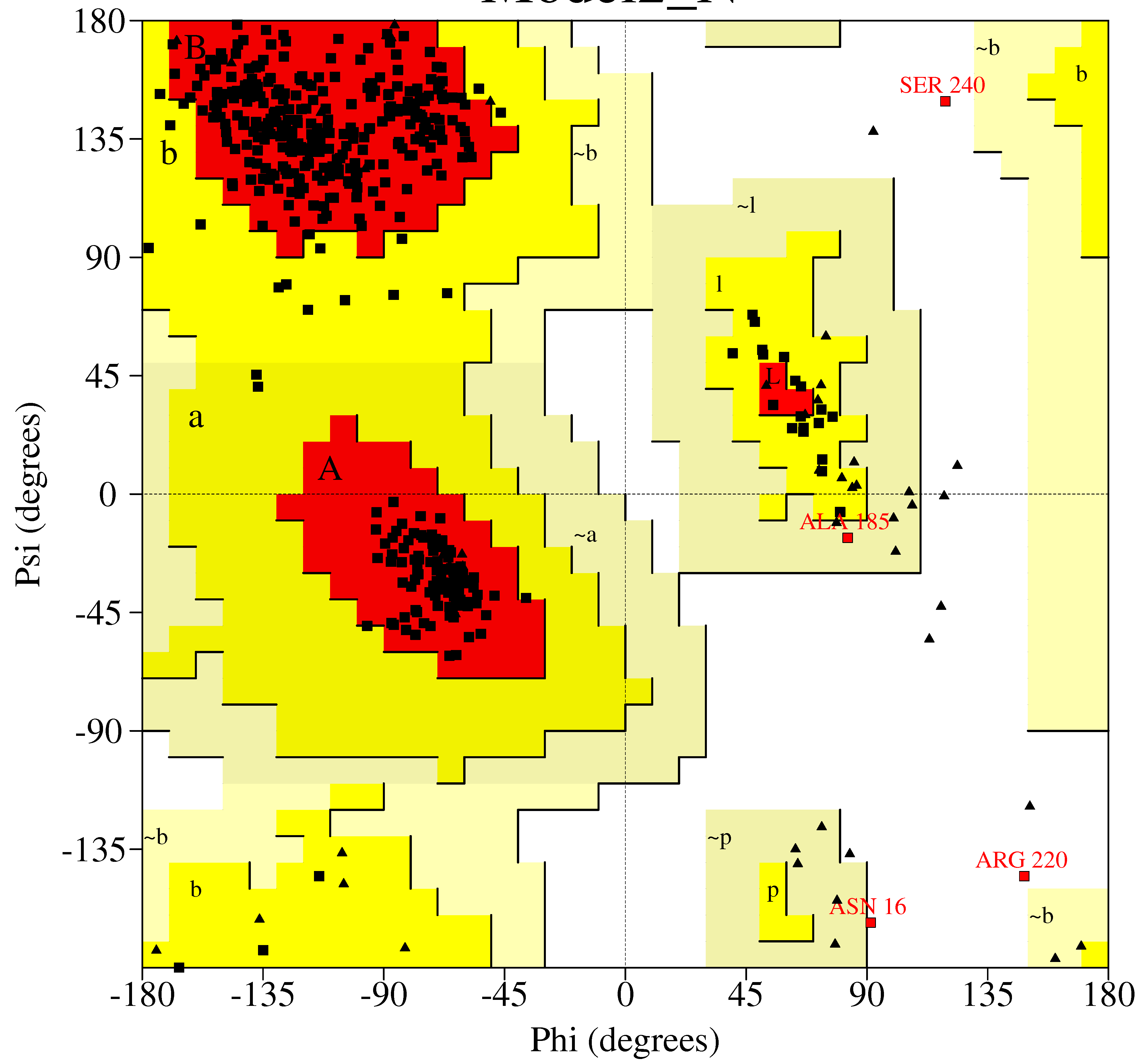

Supplement: S5 Dataset — The plots were generated through PROCHECK analysis. (ZIP) [file pone.0200607.s005.zip › Ramachandranplots/SCP4.tiff]

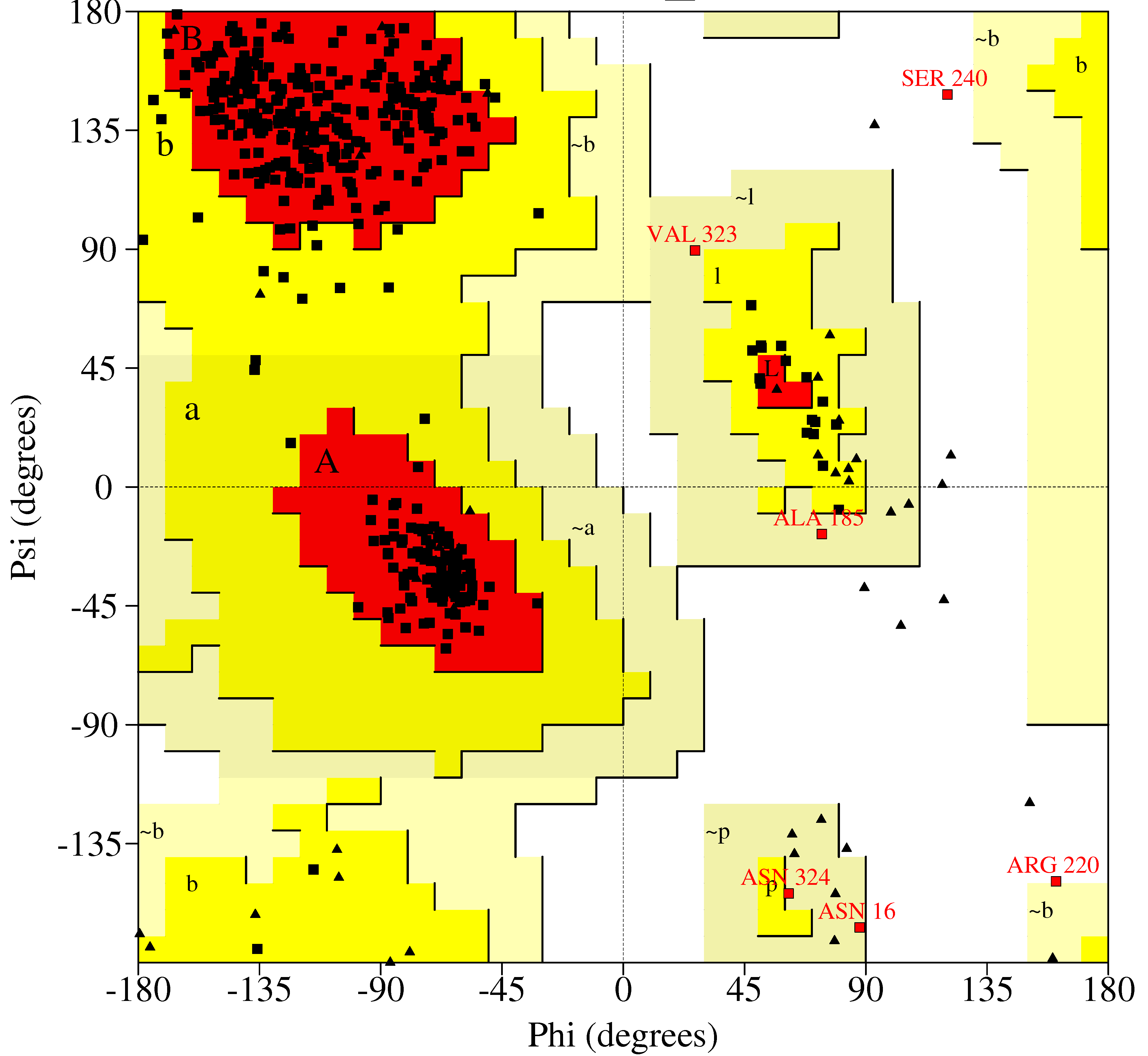

Supplement: S5 Dataset — The plots were generated through PROCHECK analysis. (ZIP) [file pone.0200607.s005.zip › Ramachandranplots/SCP5.tiff]

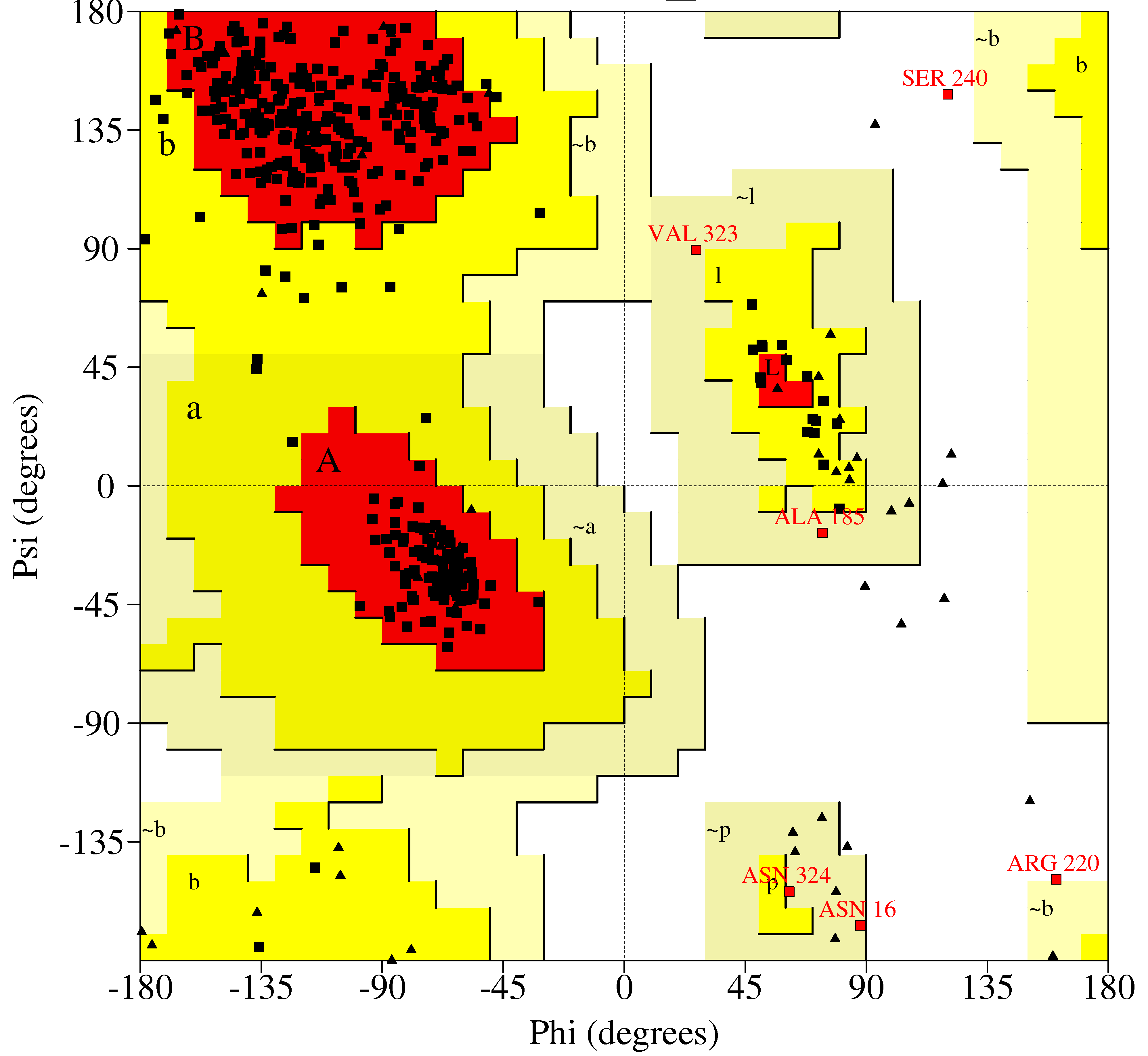

Supplement: S5 Dataset — The plots were generated through PROCHECK analysis. (ZIP) [file pone.0200607.s005.zip › Ramachandranplots/SCP6.tiff]

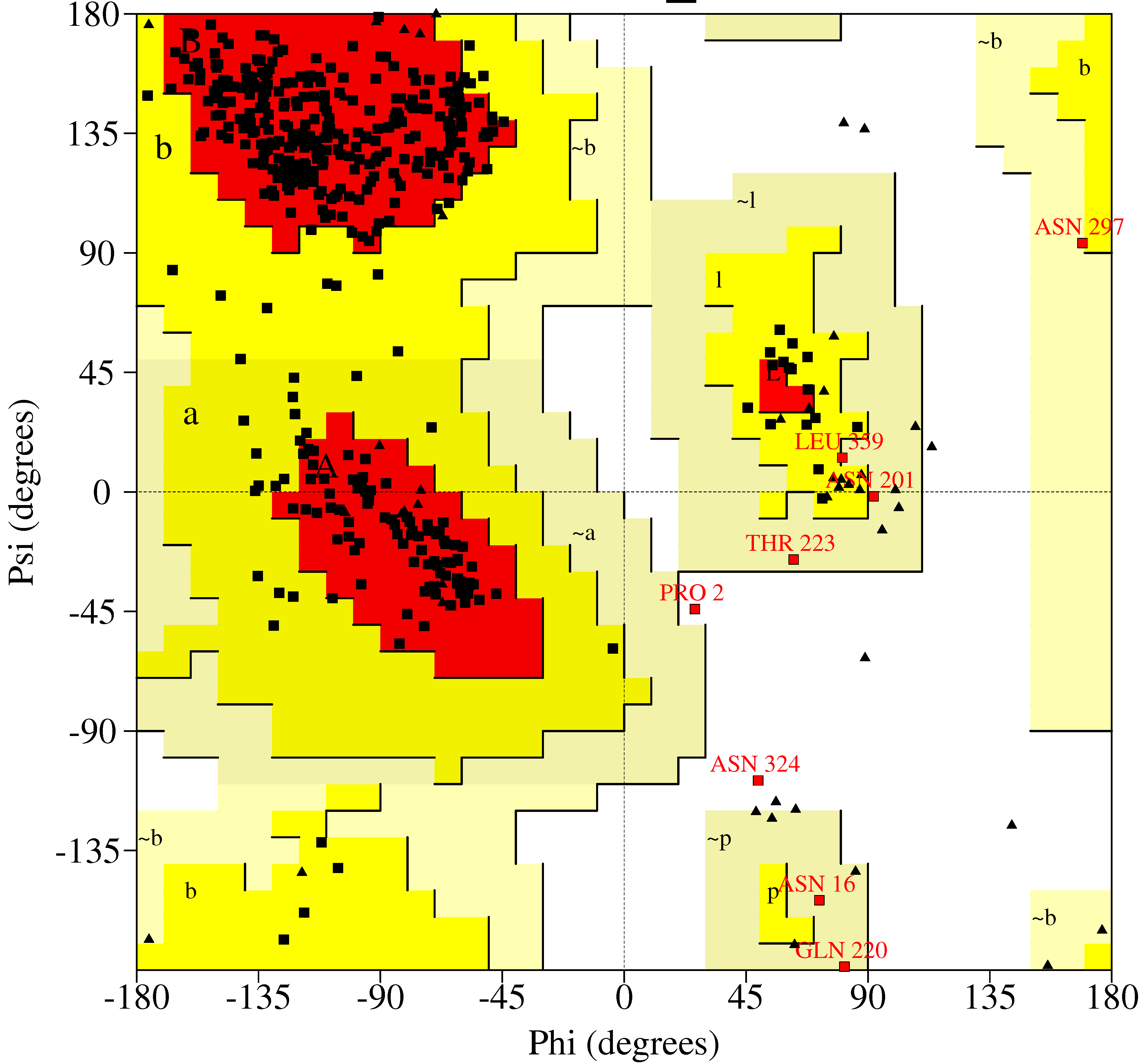

Supplement: S5 Dataset — The plots were generated through PROCHECK analysis. (ZIP) [file pone.0200607.s005.zip › Ramachandranplots/SCP7.tiff]

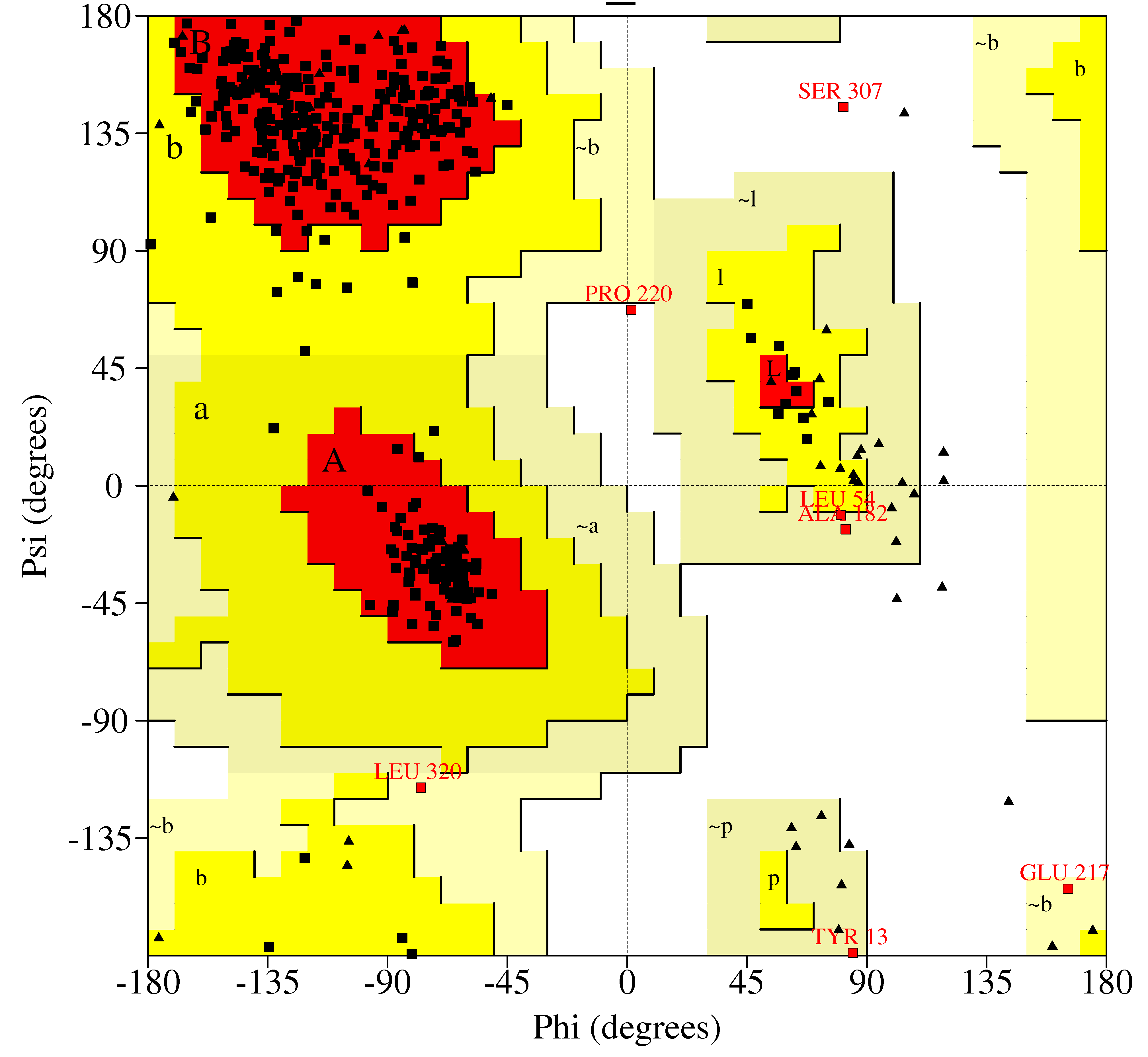

Supplement: S5 Dataset — The plots were generated through PROCHECK analysis. (ZIP) [file pone.0200607.s005.zip › Ramachandranplots/TCP1.tiff]

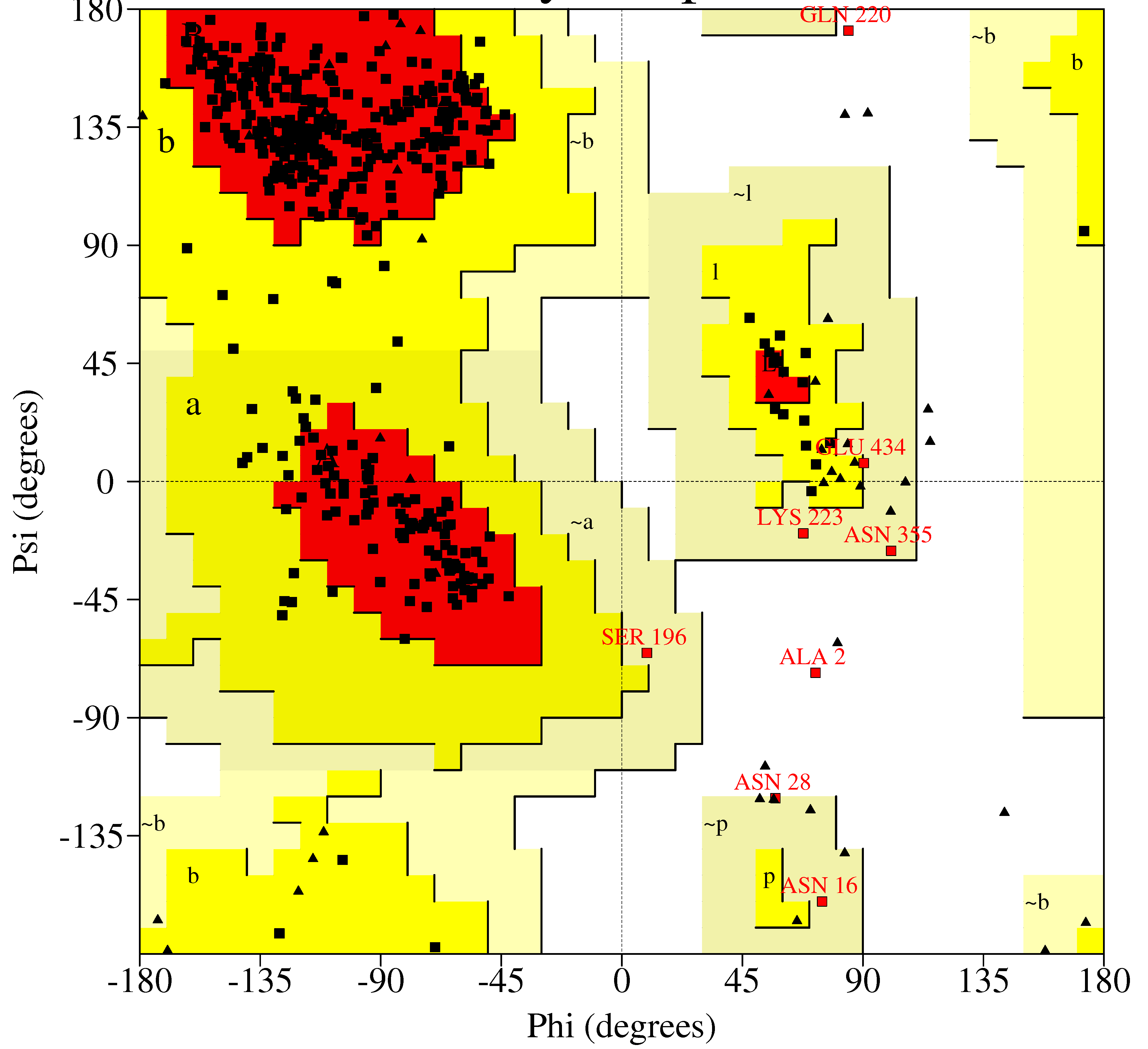

Supplement: S5 Dataset — The plots were generated through PROCHECK analysis. (ZIP) [file pone.0200607.s005.zip › Ramachandranplots/TCP2.tiff]

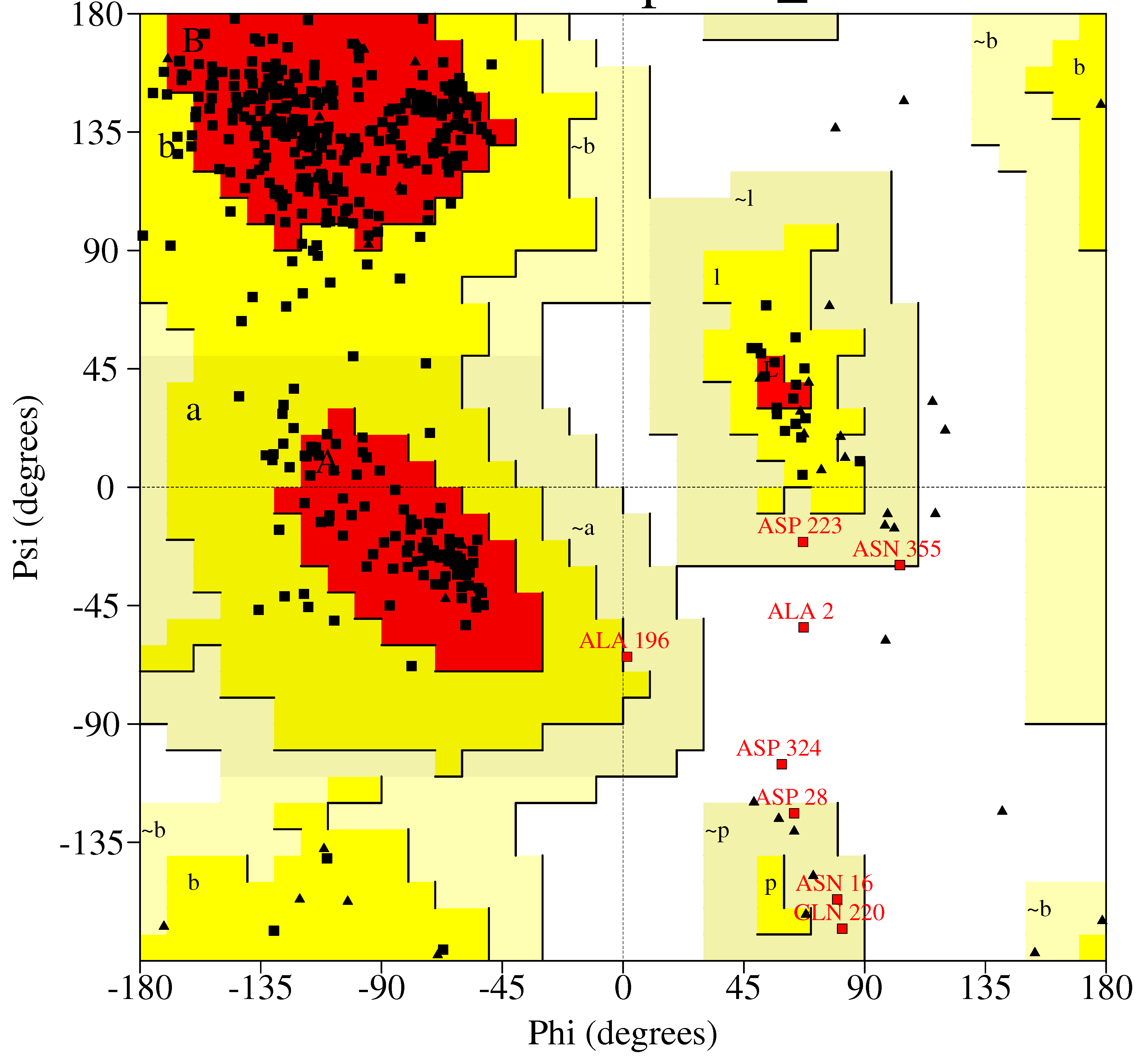

Supplement: S5 Dataset — The plots were generated through PROCHECK analysis. (ZIP) [file pone.0200607.s005.zip › Ramachandranplots/TIP1.tiff]

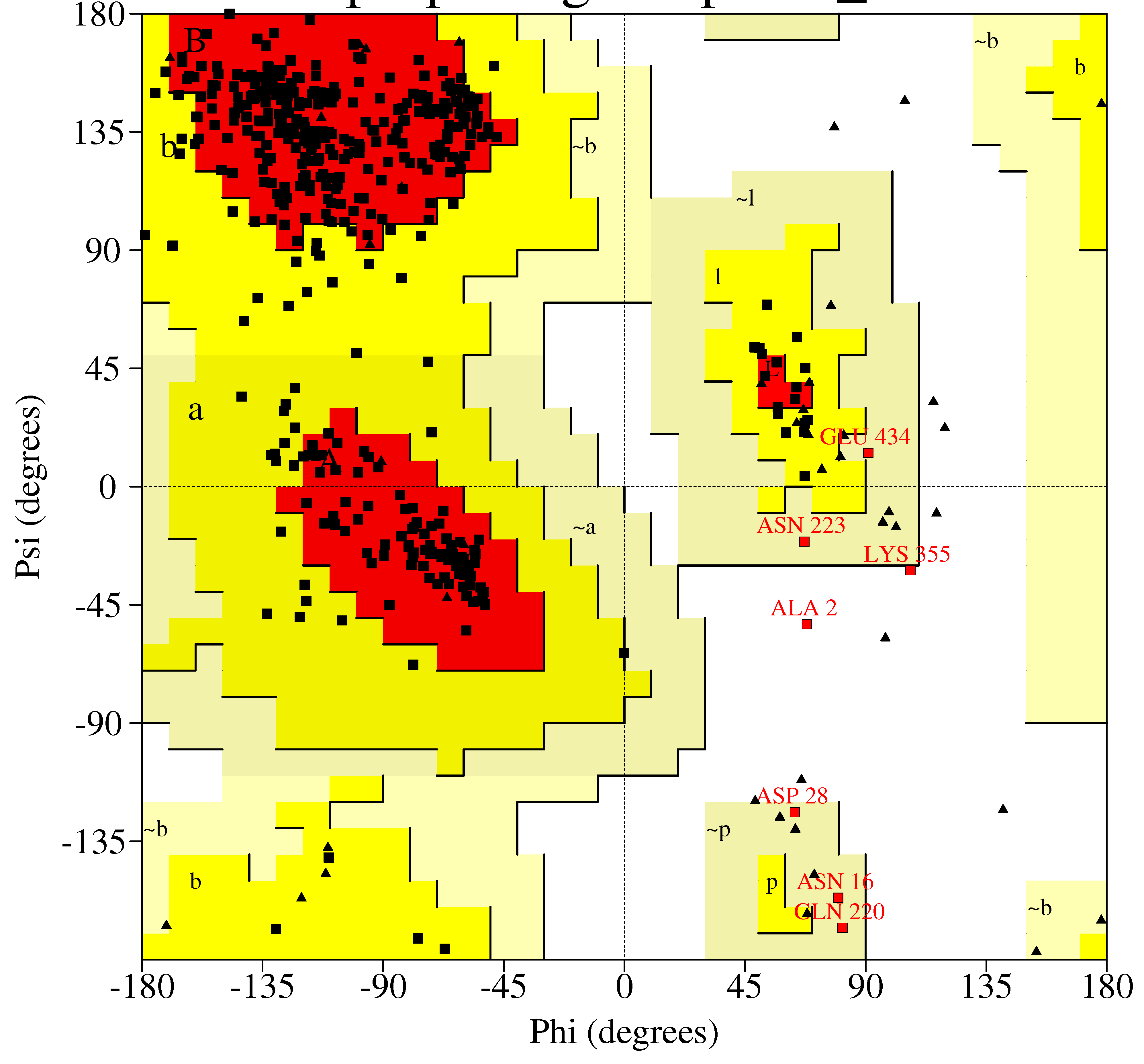

Supplement: S5 Dataset — The plots were generated through PROCHECK analysis. (ZIP) [file pone.0200607.s005.zip › Ramachandranplots/TPP1.tiff]

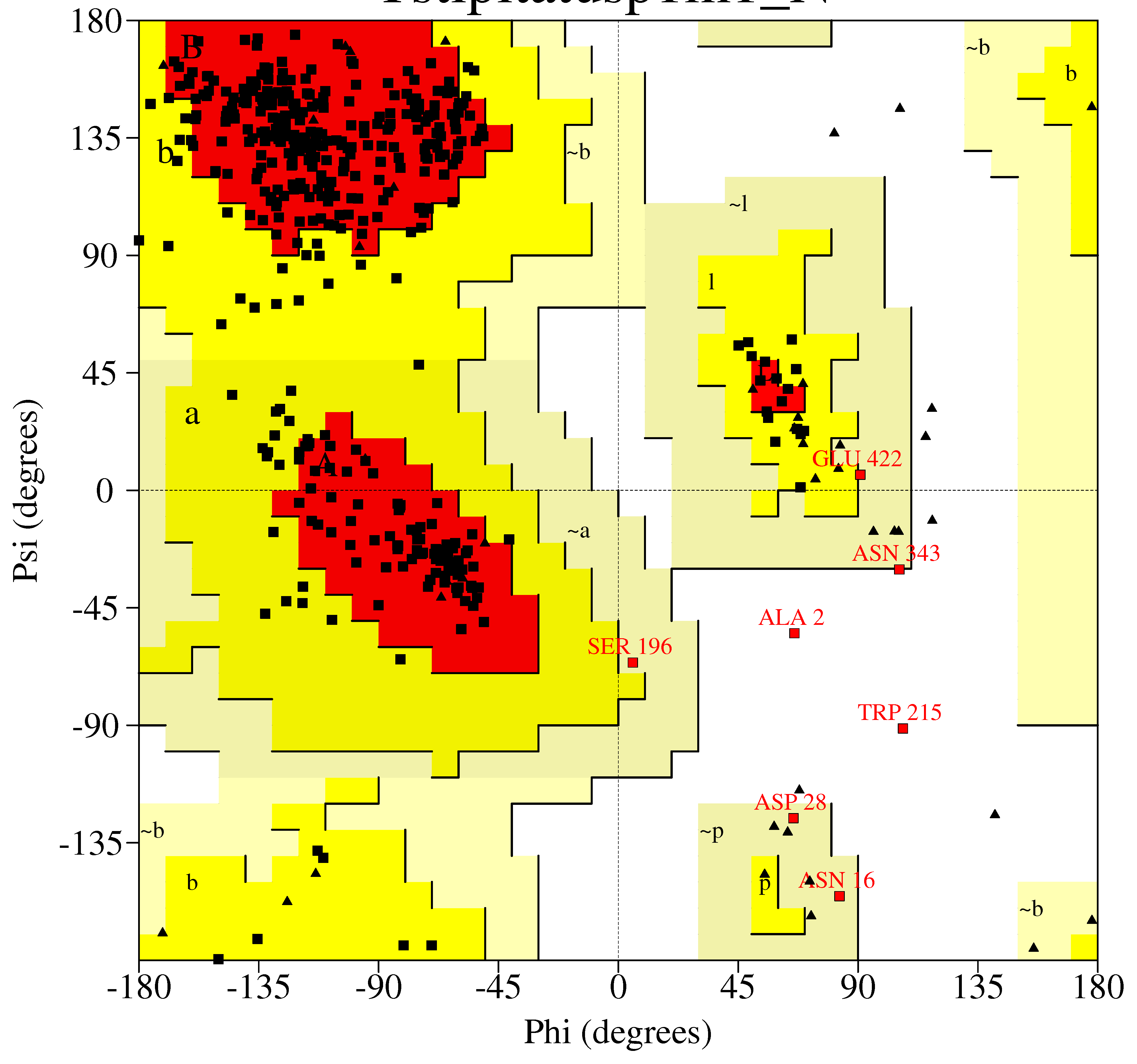

Supplement: S5 Dataset — The plots were generated through PROCHECK analysis. (ZIP) [file pone.0200607.s005.zip › Ramachandranplots/TSP1.tiff]

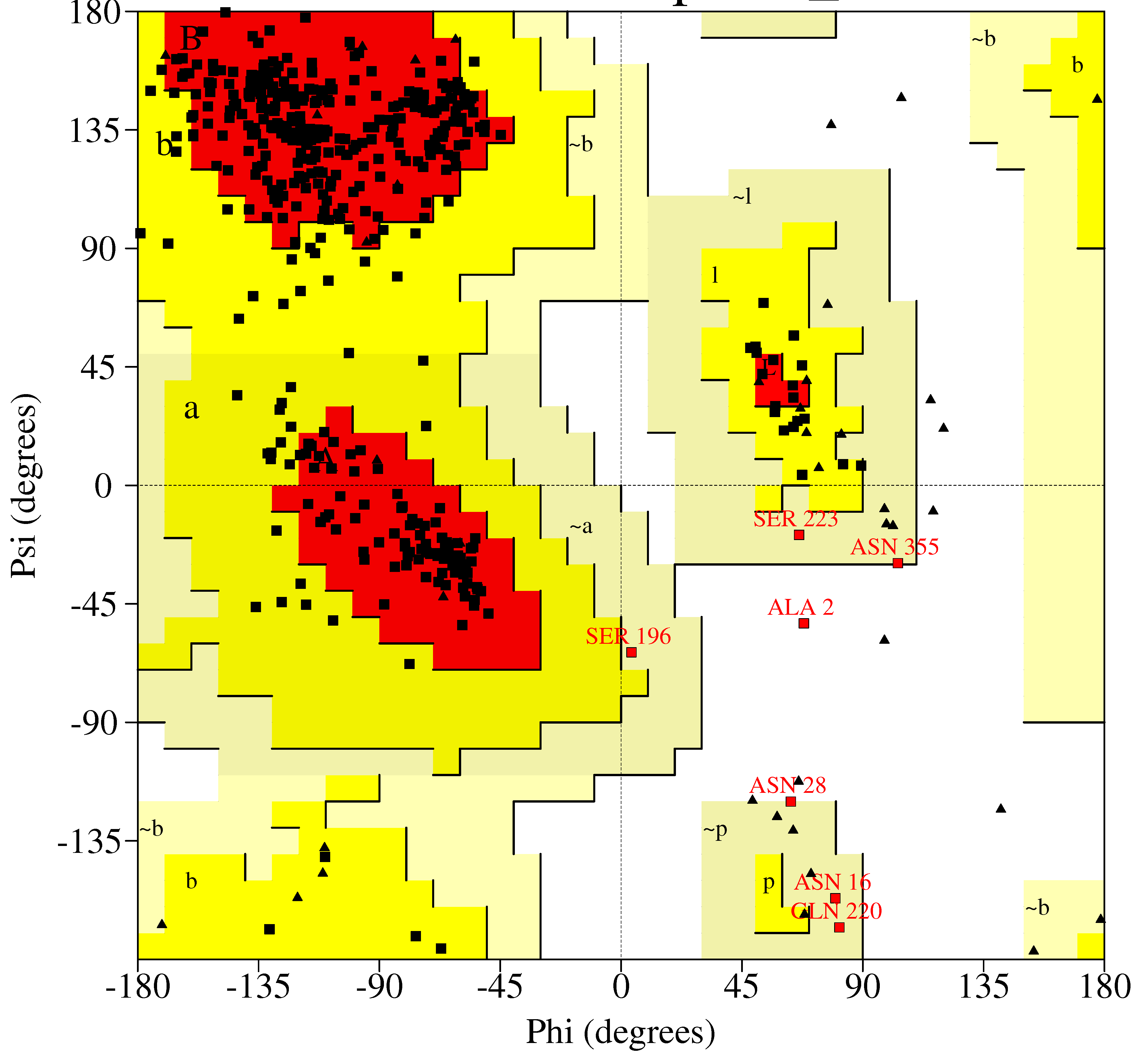

Supplement: S5 Dataset — The plots were generated through PROCHECK analysis. (ZIP) [file pone.0200607.s005.zip › Ramachandranplots/TVP1.tiff]

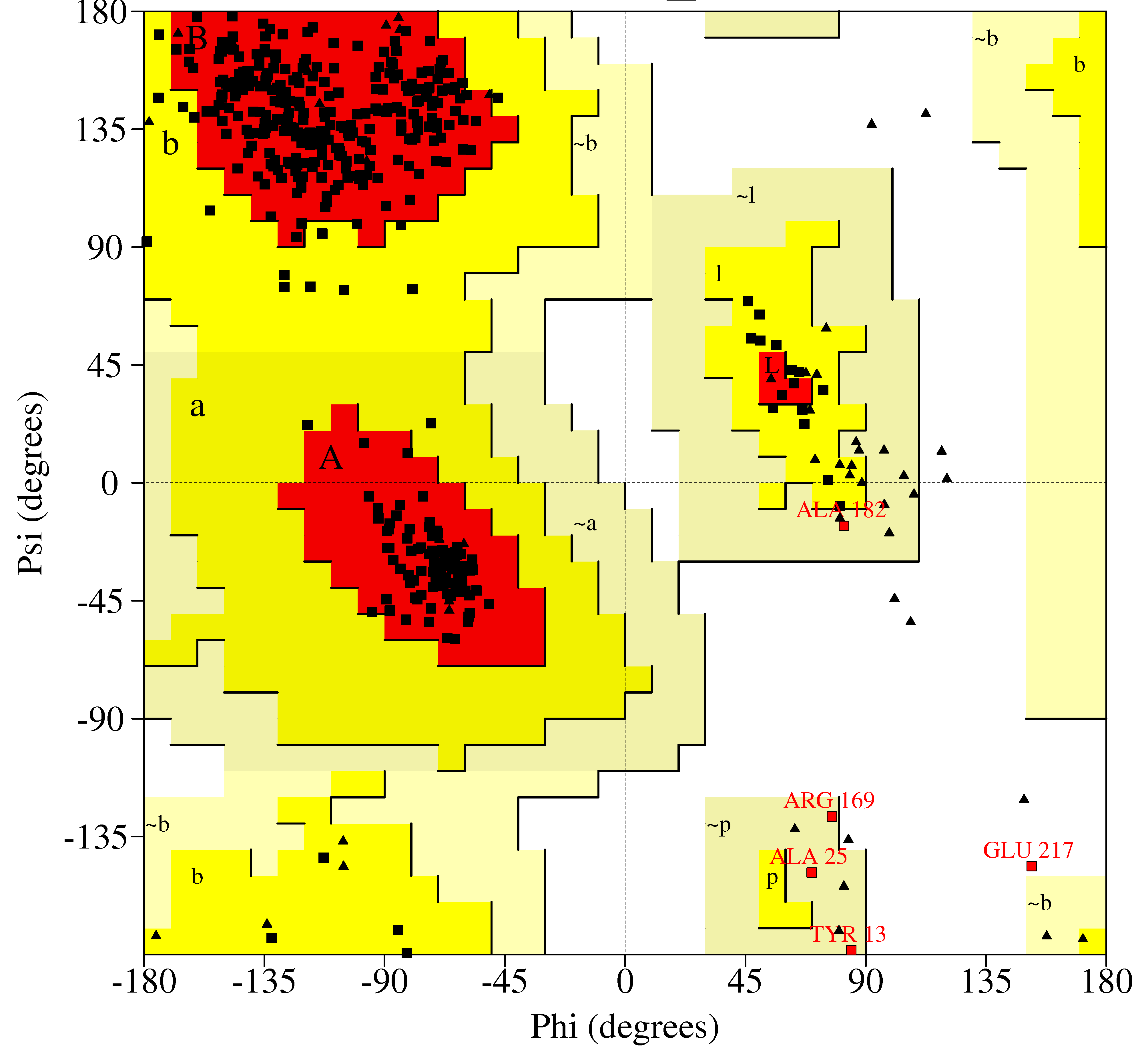

Supplement: S5 Dataset — The plots were generated through PROCHECK analysis. (ZIP) [file pone.0200607.s005.zip › Ramachandranplots/TVP2.tiff]

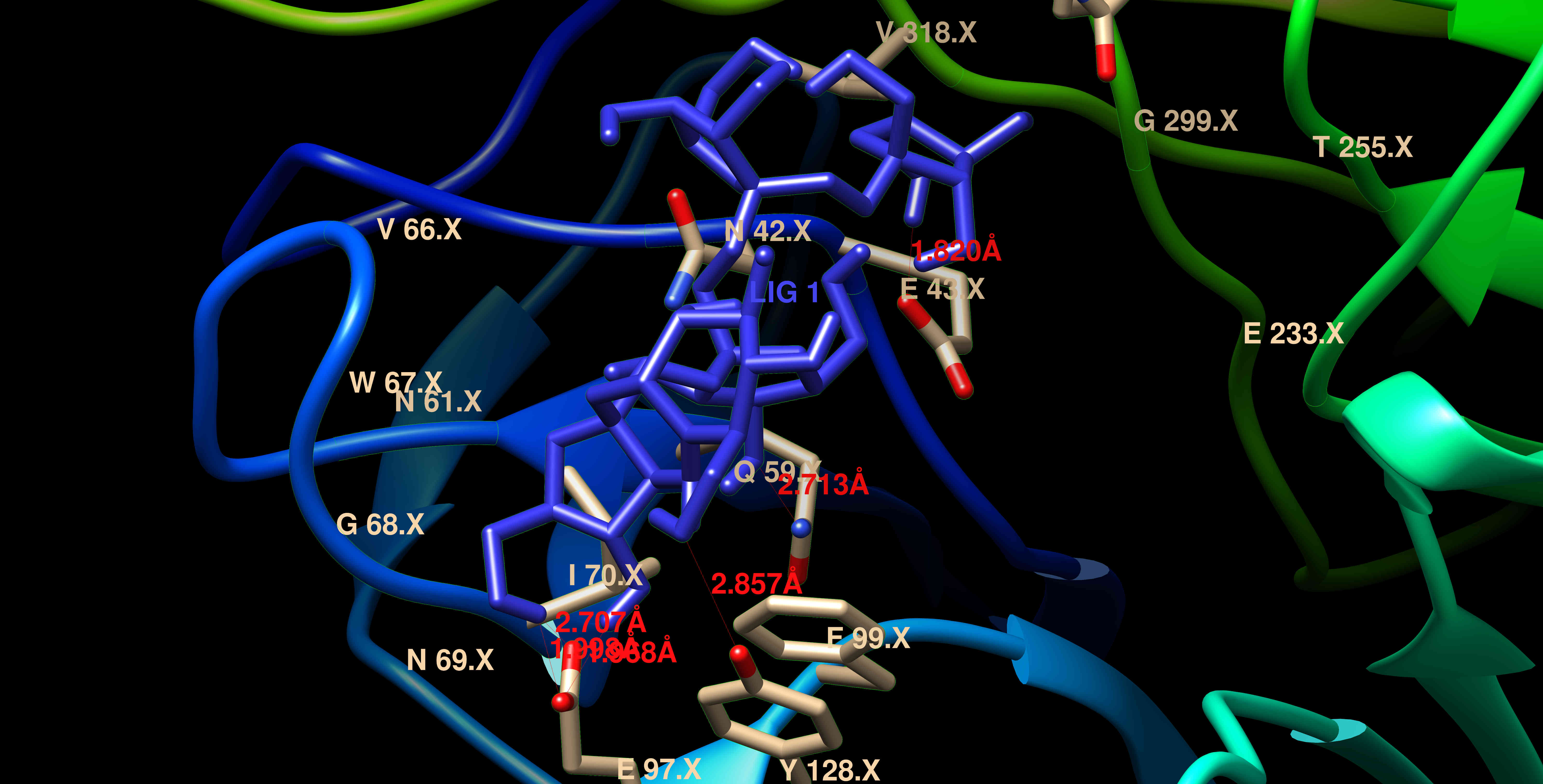

Supplement: S7 Dataset — (ZIP) [file pone.0200607.s007.zip › Docking_Images/3SC7_Docked.jpg]

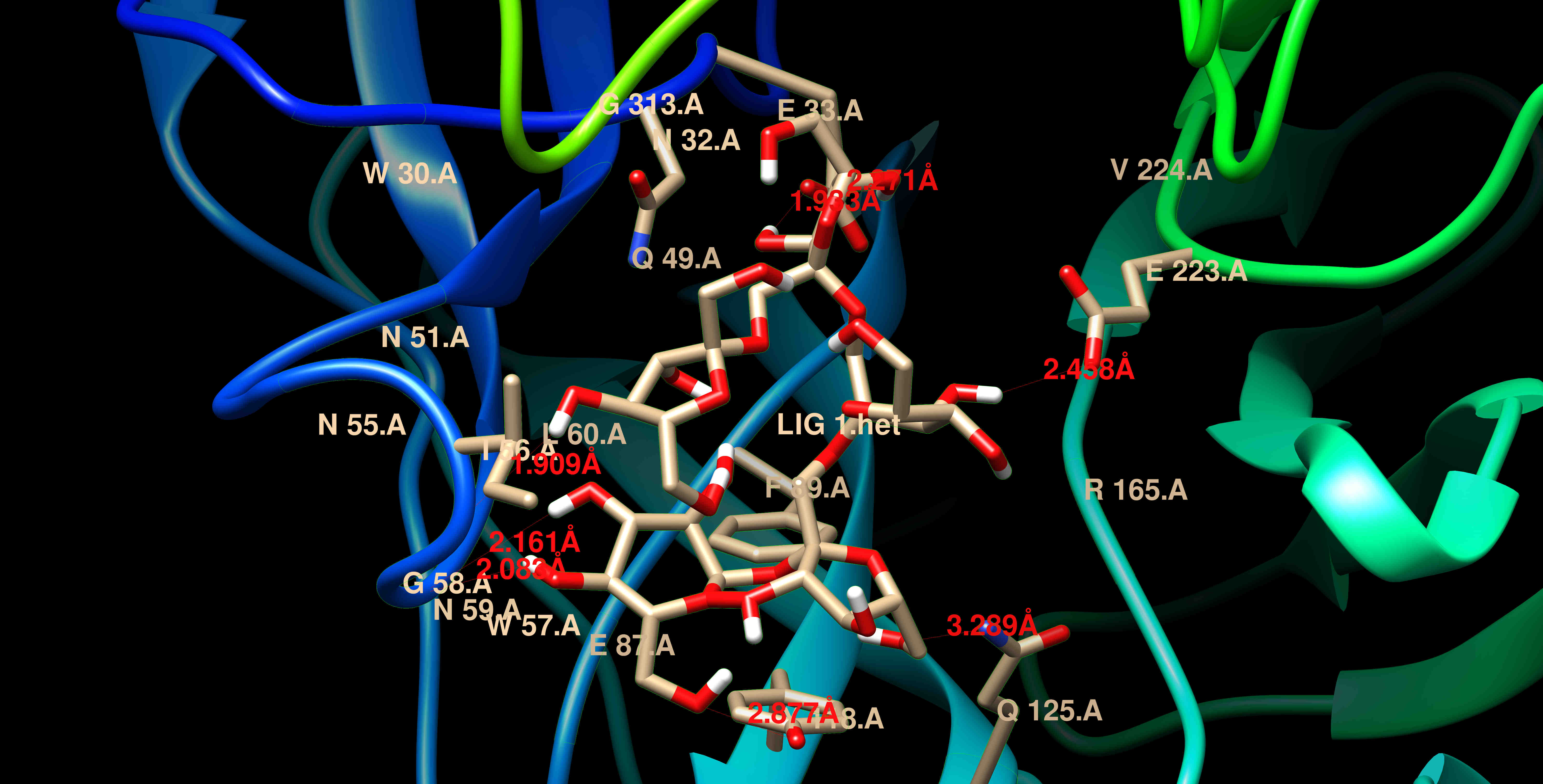

Supplement: S7 Dataset — (ZIP) [file pone.0200607.s007.zip › Docking_Images/ACP1_Docked.jpg]

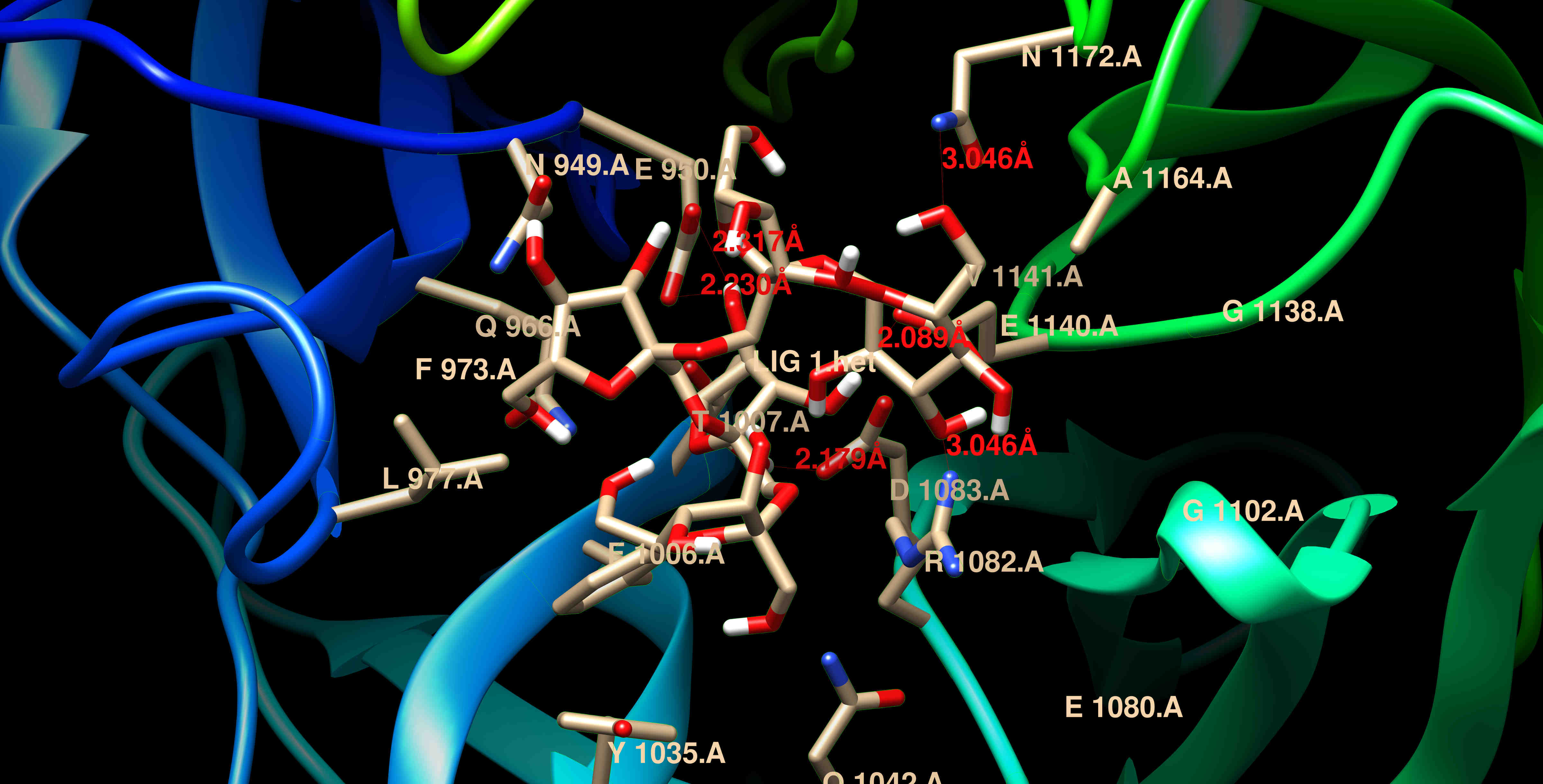

Supplement: S7 Dataset — (ZIP) [file pone.0200607.s007.zip › Docking_Images/ACP2_Docked.jpg]

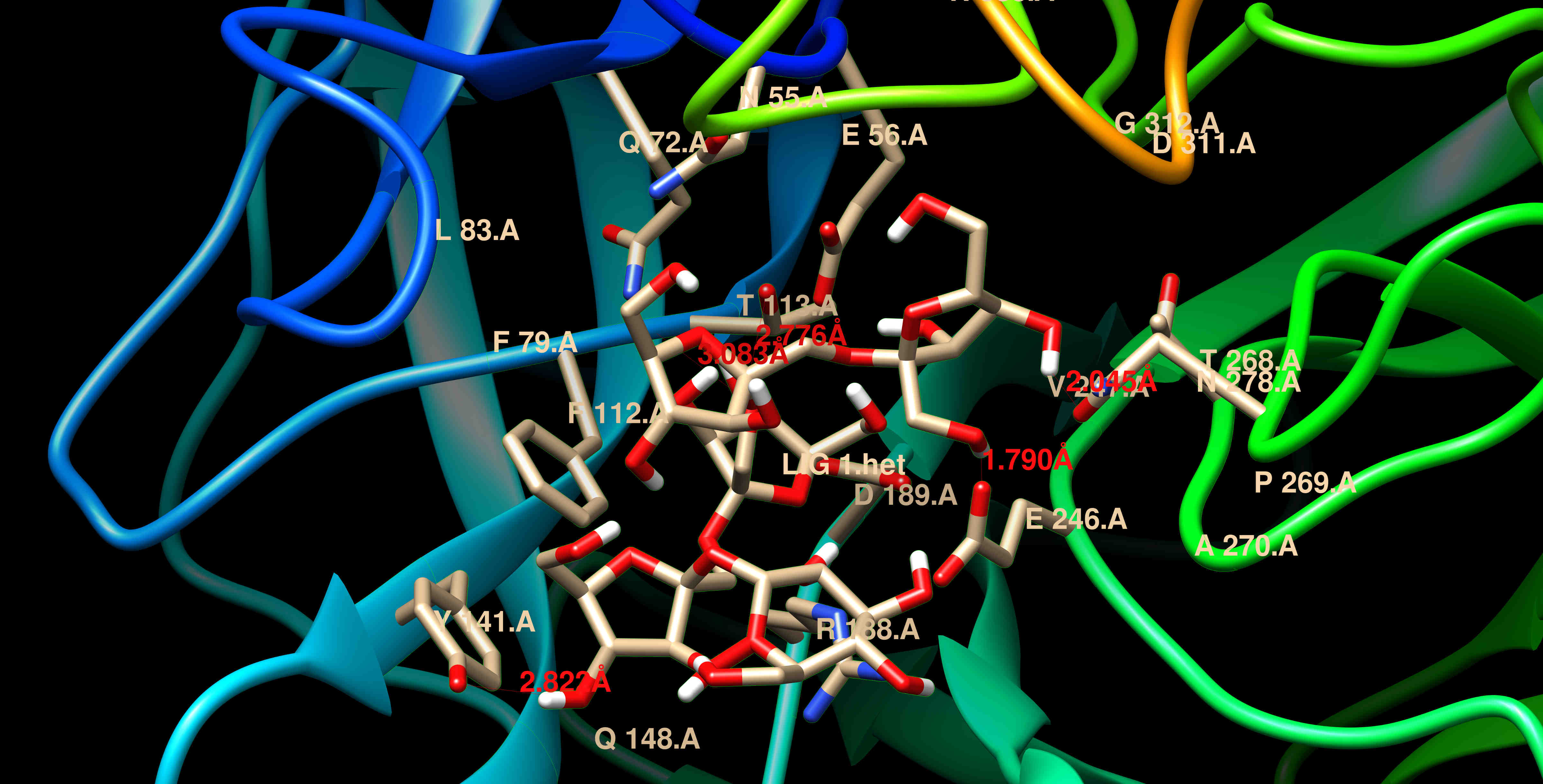

Supplement: S7 Dataset — (ZIP) [file pone.0200607.s007.zip › Docking_Images/AFP1_Docked.jpg]

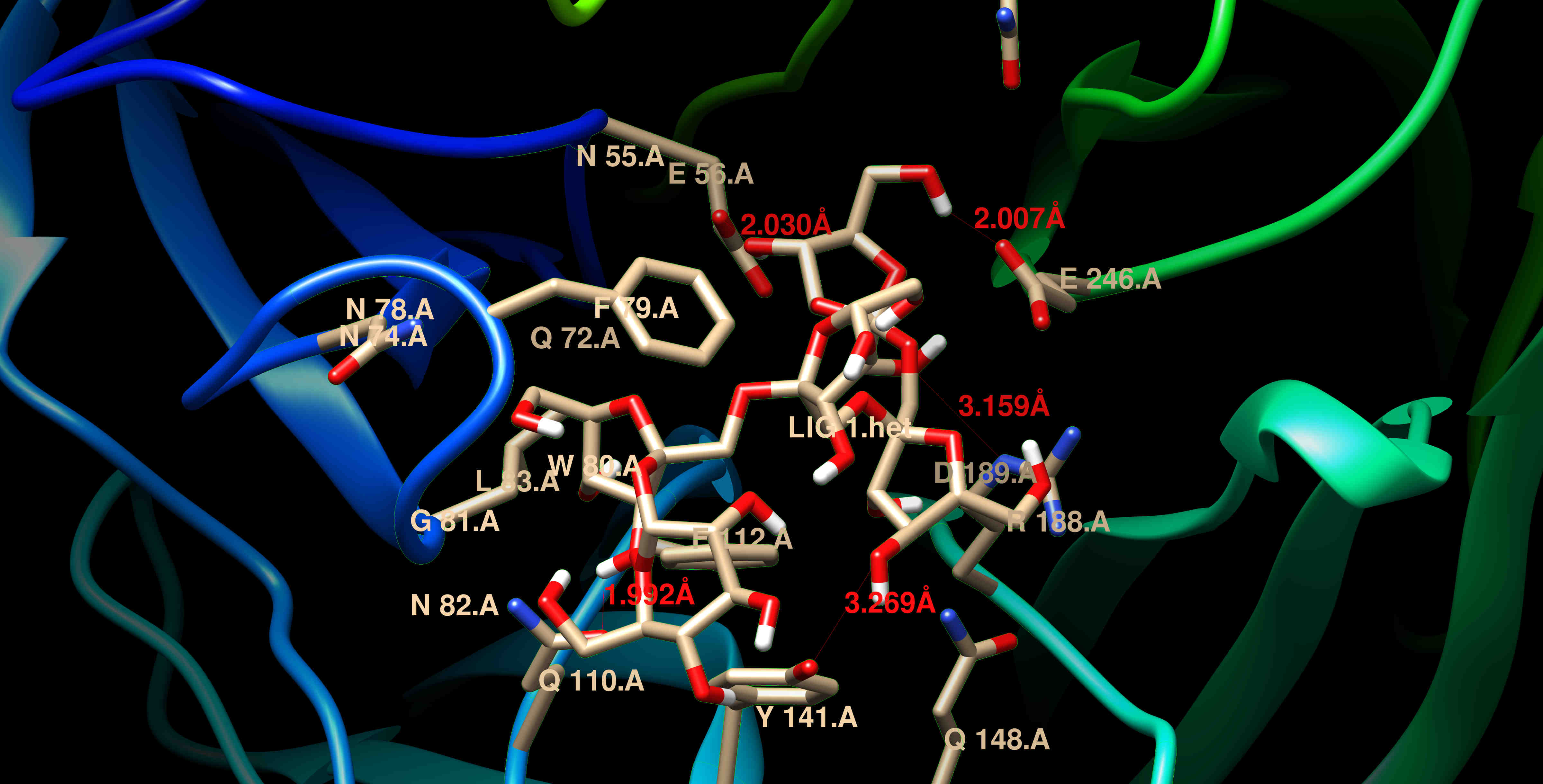

Supplement: S7 Dataset — (ZIP) [file pone.0200607.s007.zip › Docking_Images/AFP2_Docked.jpg]

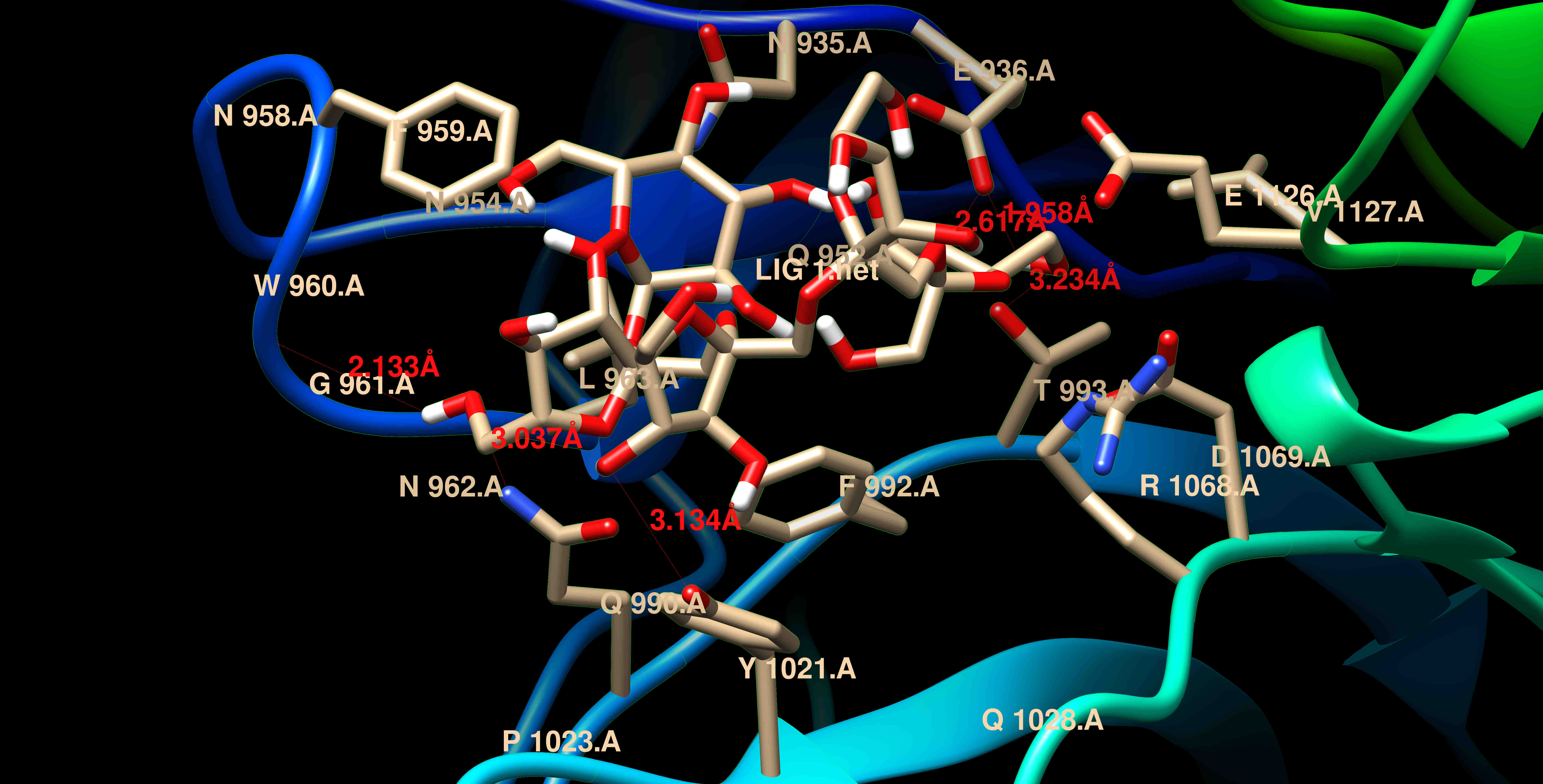

Supplement: S7 Dataset — (ZIP) [file pone.0200607.s007.zip › Docking_Images/AFSP1.jpg]

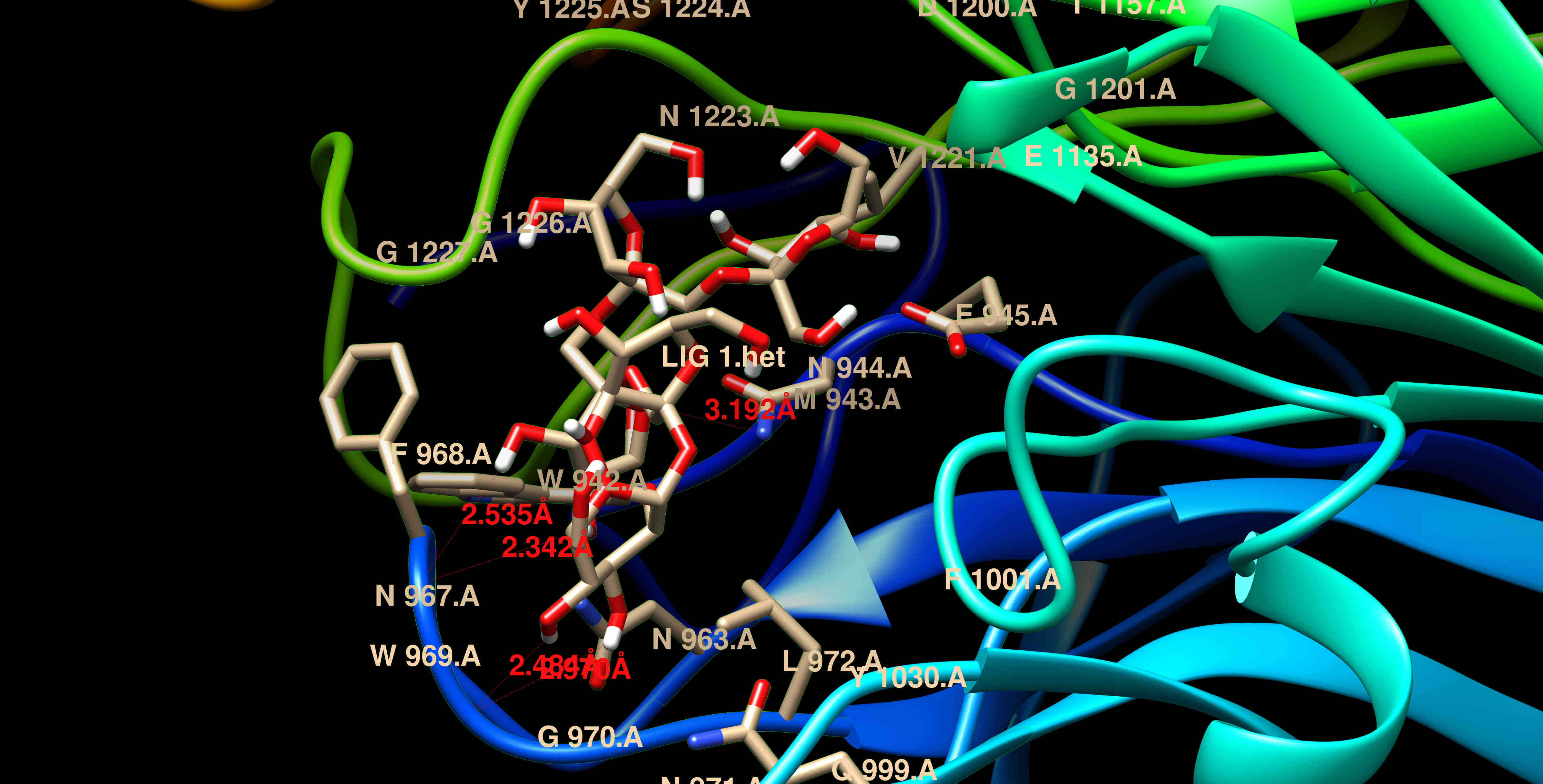

Supplement: S7 Dataset — (ZIP) [file pone.0200607.s007.zip › Docking_Images/ALP1_Docked.jpg]

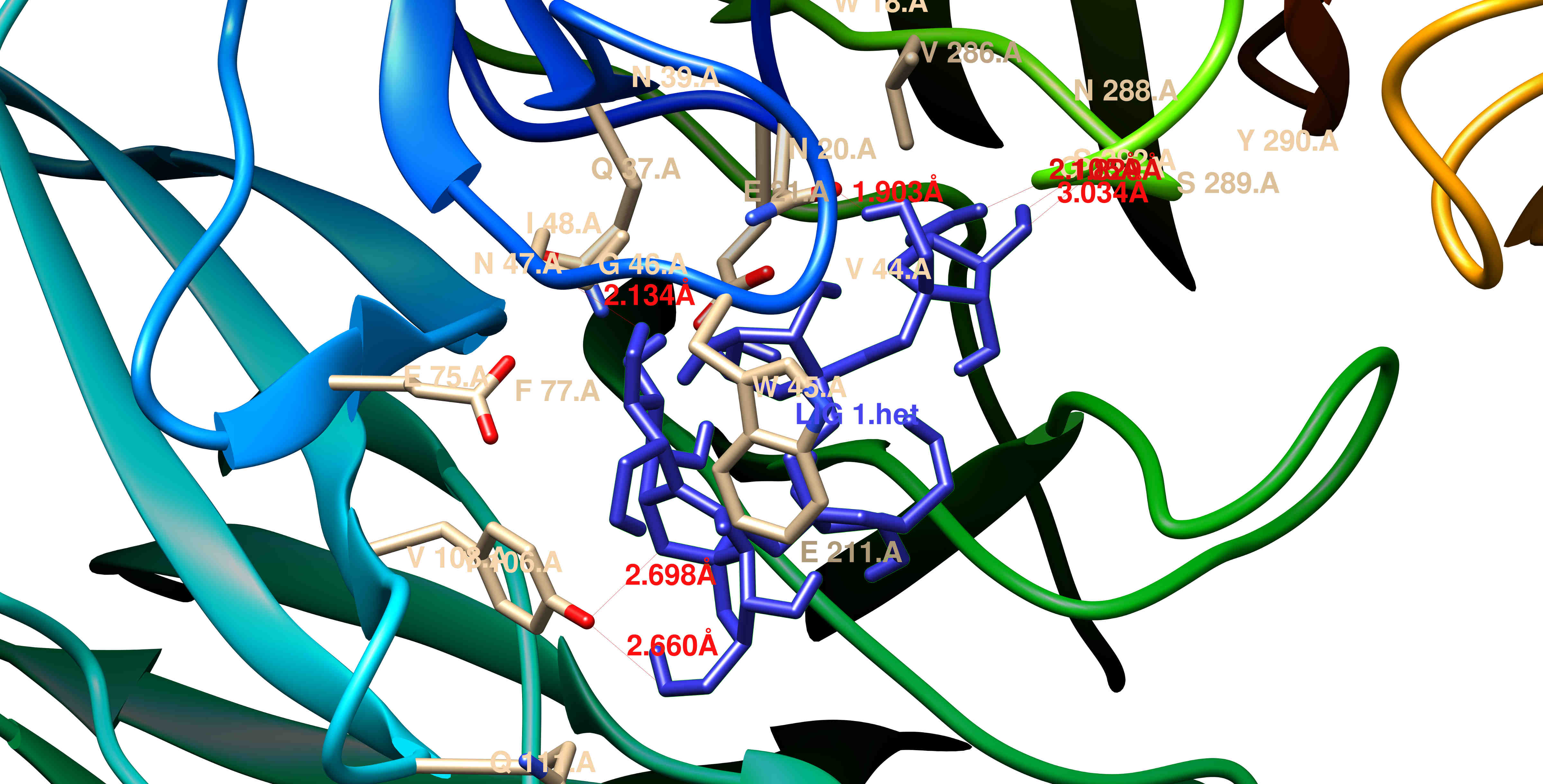

Supplement: S7 Dataset — (ZIP) [file pone.0200607.s007.zip › Docking_Images/ANP10_Docked.jpg]

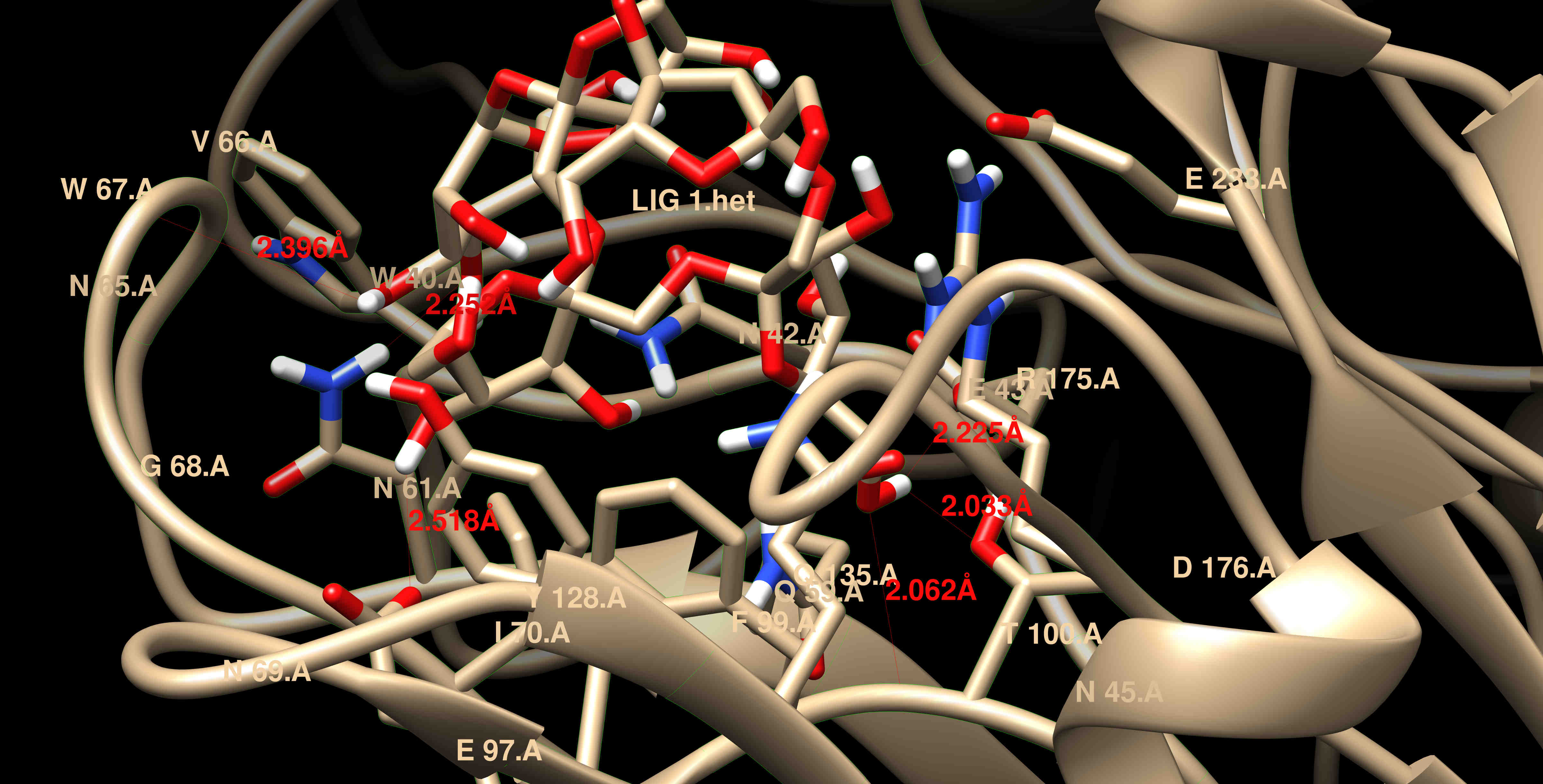

Supplement: S7 Dataset — (ZIP) [file pone.0200607.s007.zip › Docking_Images/ANP1_Docked.jpg]

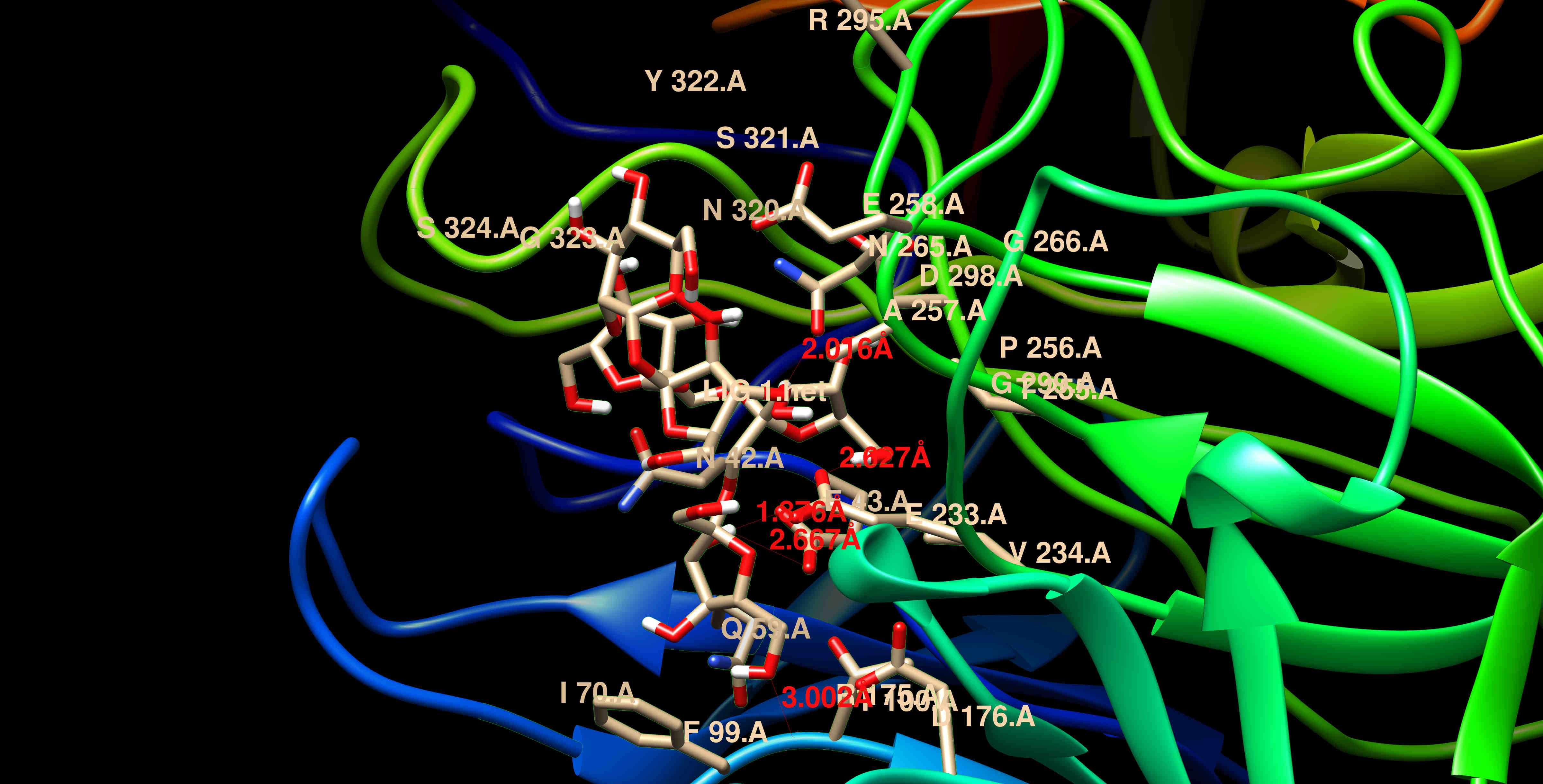

Supplement: S7 Dataset — (ZIP) [file pone.0200607.s007.zip › Docking_Images/ANP2_Docked2.jpg]

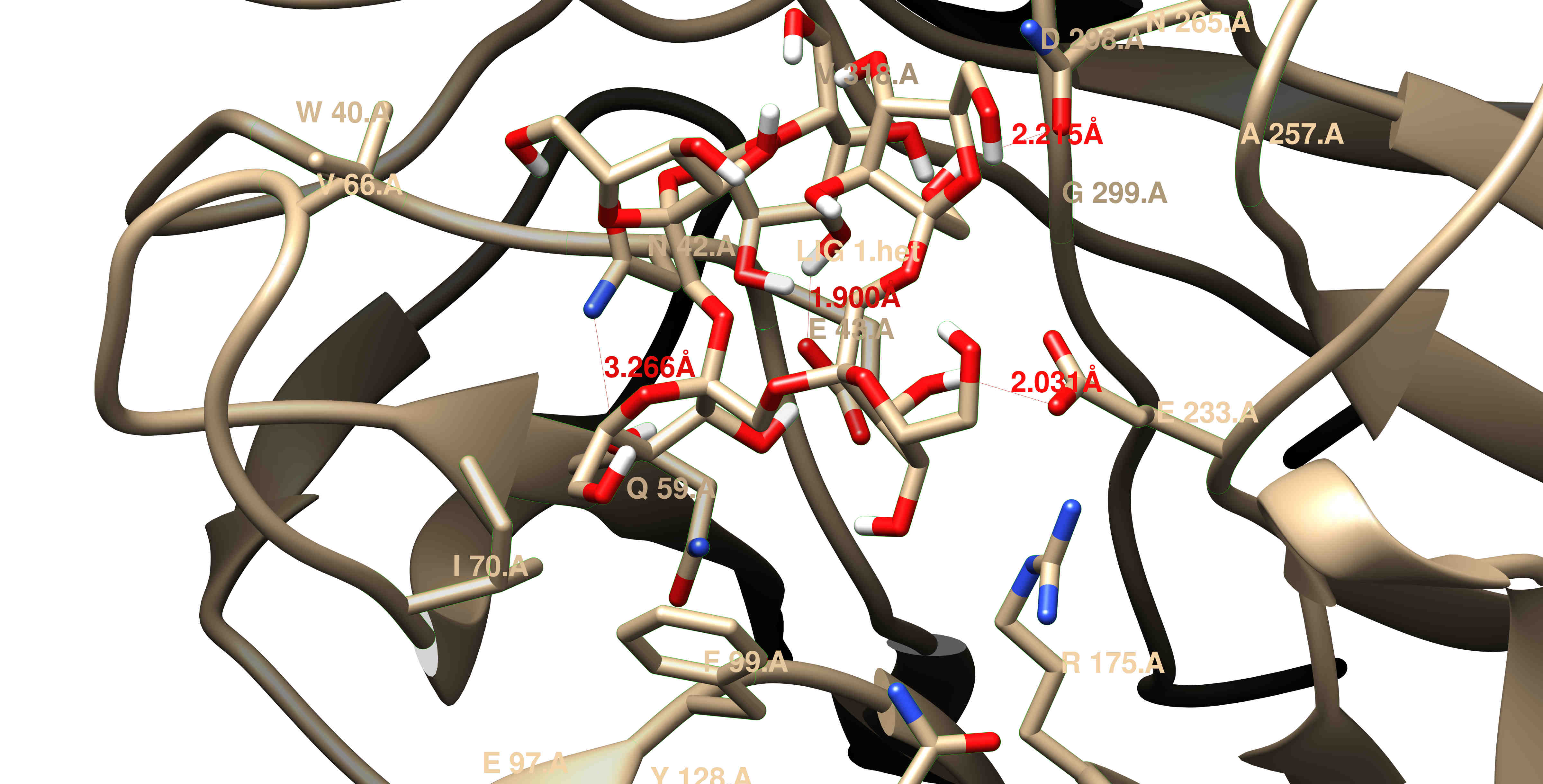

Supplement: S7 Dataset — (ZIP) [file pone.0200607.s007.zip › Docking_Images/ANP3_Docked.jpg]

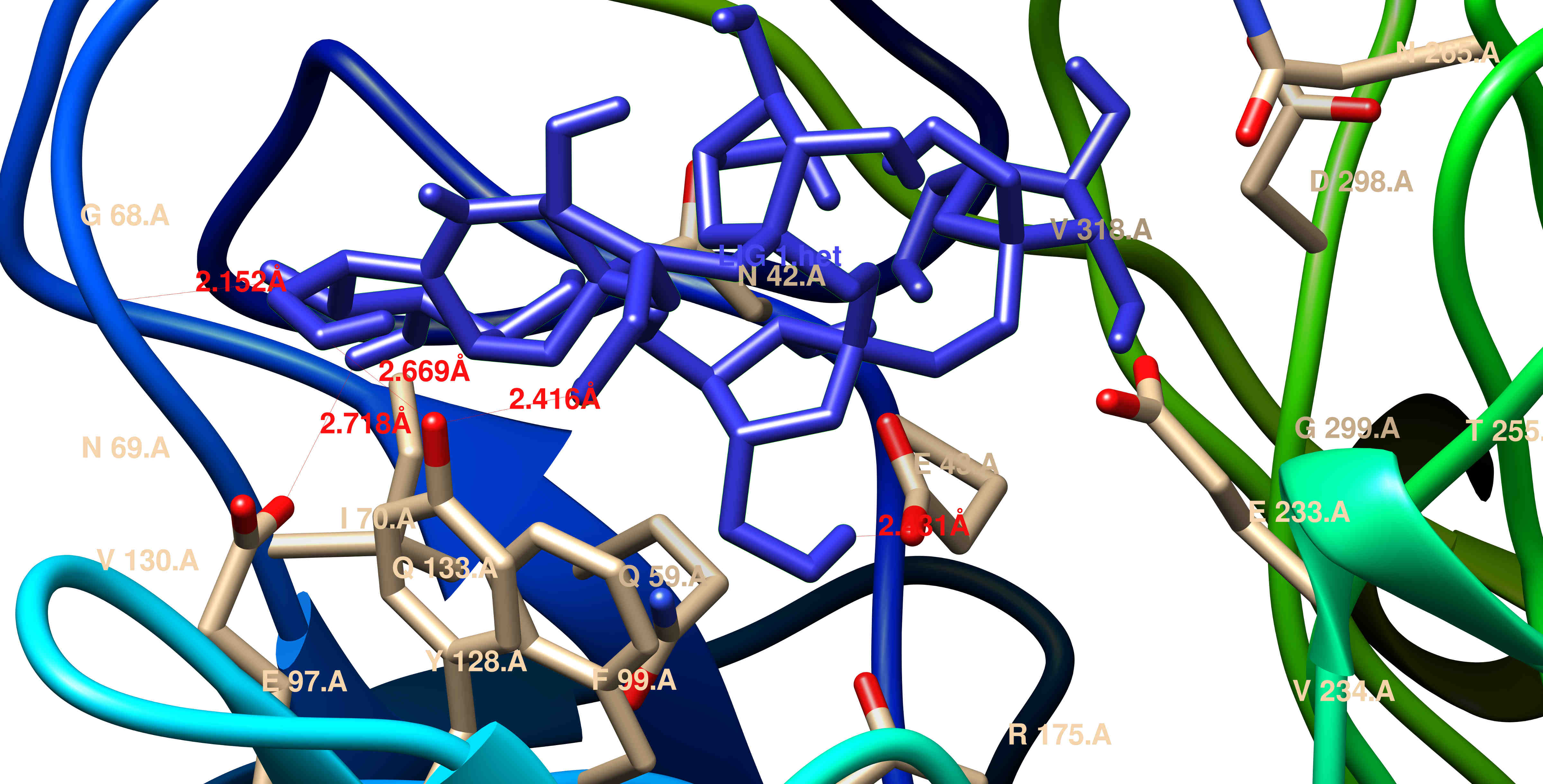

Supplement: S7 Dataset — (ZIP) [file pone.0200607.s007.zip › Docking_Images/ANP4_Docked.jpg]

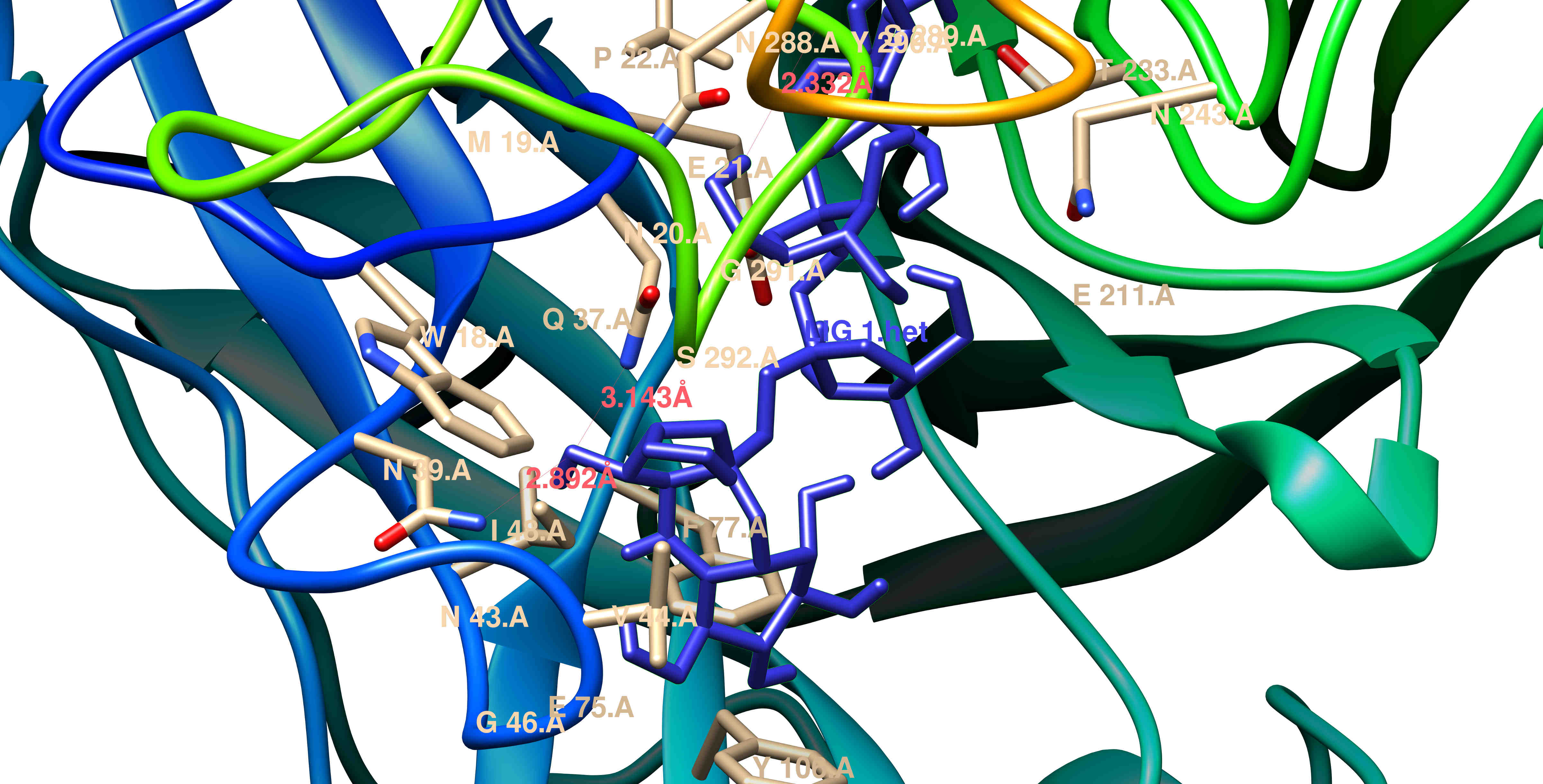

Supplement: S7 Dataset — (ZIP) [file pone.0200607.s007.zip › Docking_Images/ANP5_Docked.jpg]

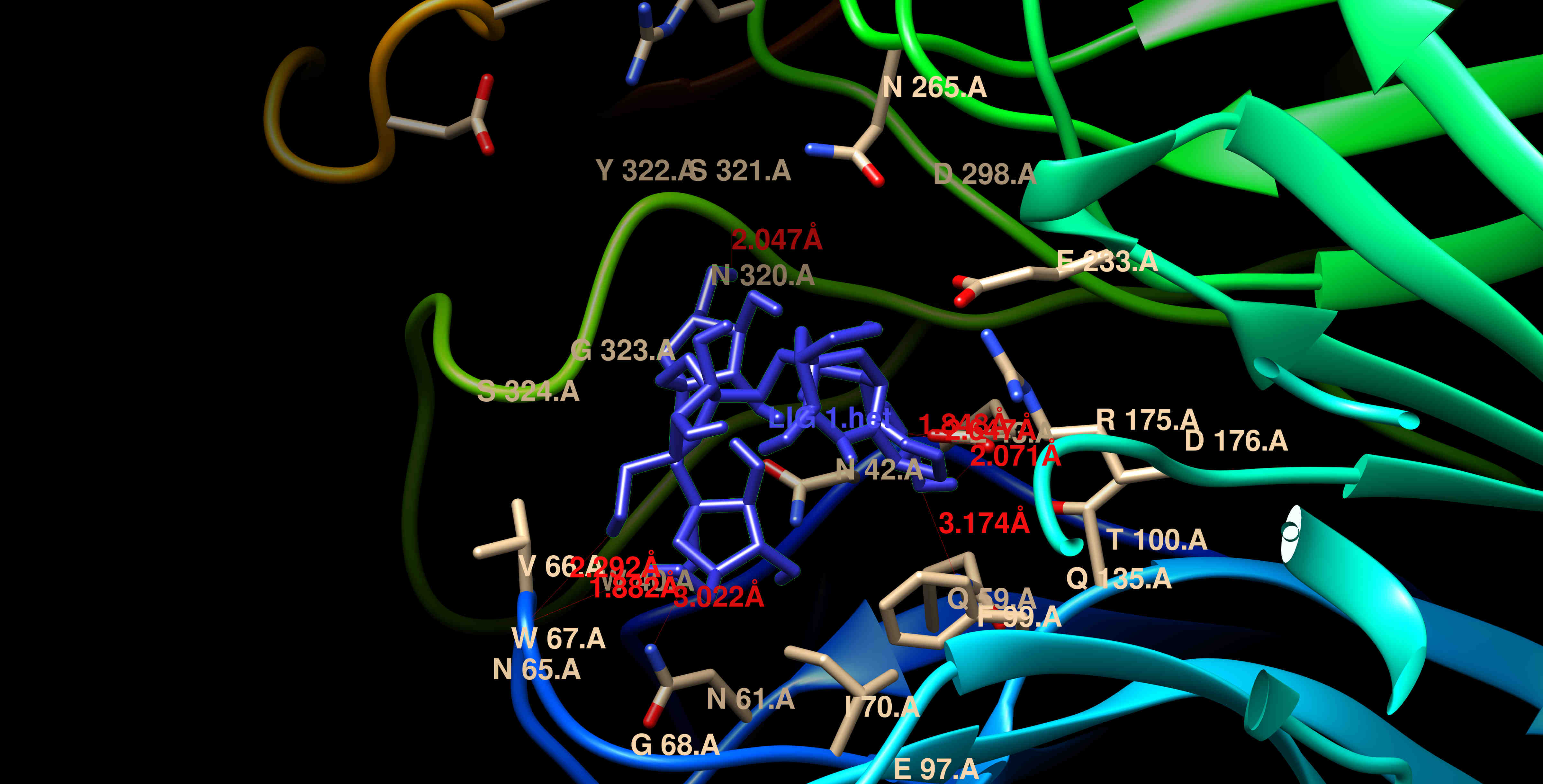

Supplement: S7 Dataset — (ZIP) [file pone.0200607.s007.zip › Docking_Images/ANP7_Docked.jpg]

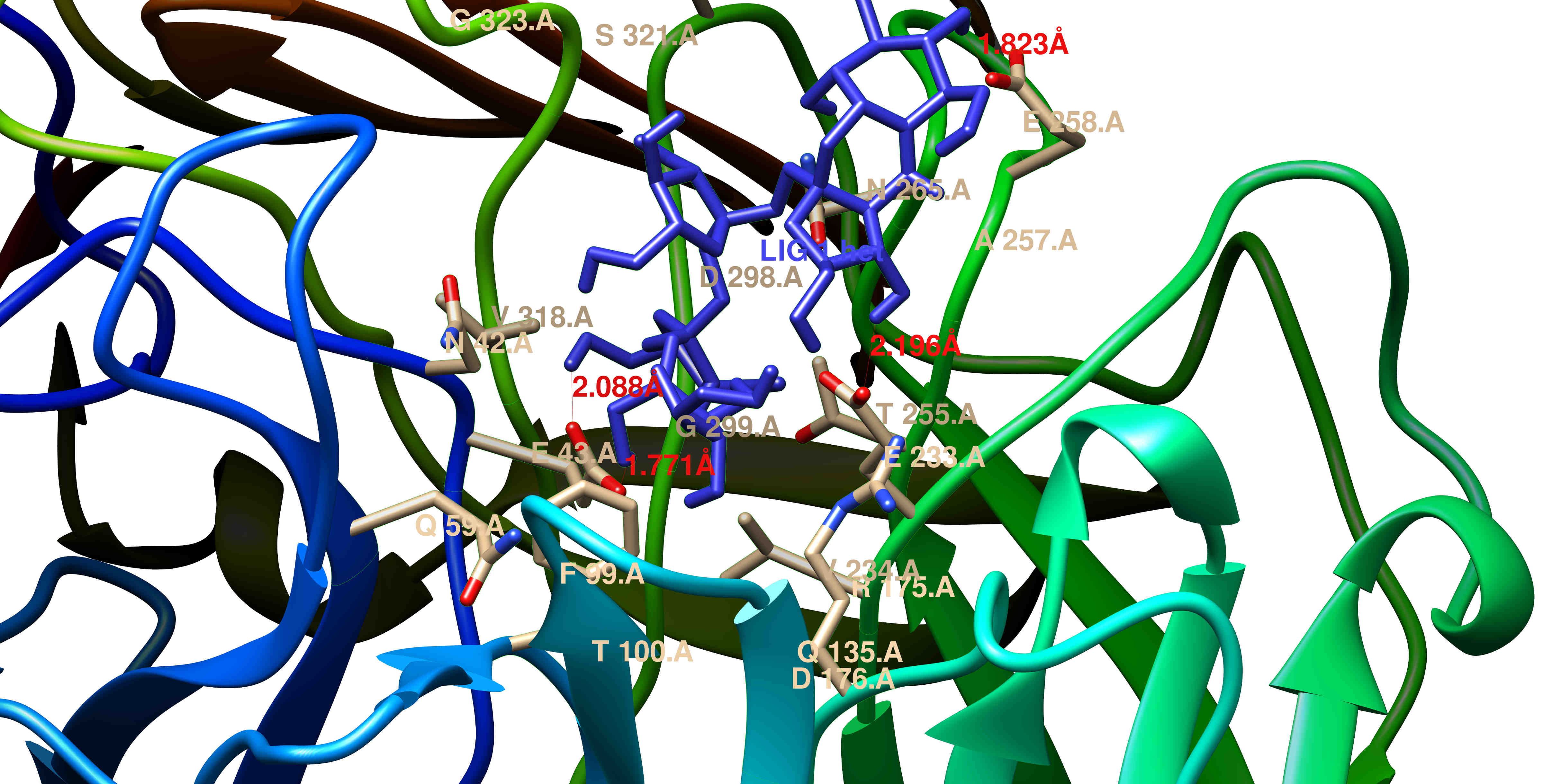

Supplement: S7 Dataset — (ZIP) [file pone.0200607.s007.zip › Docking_Images/ANP9_Docked.jpg]

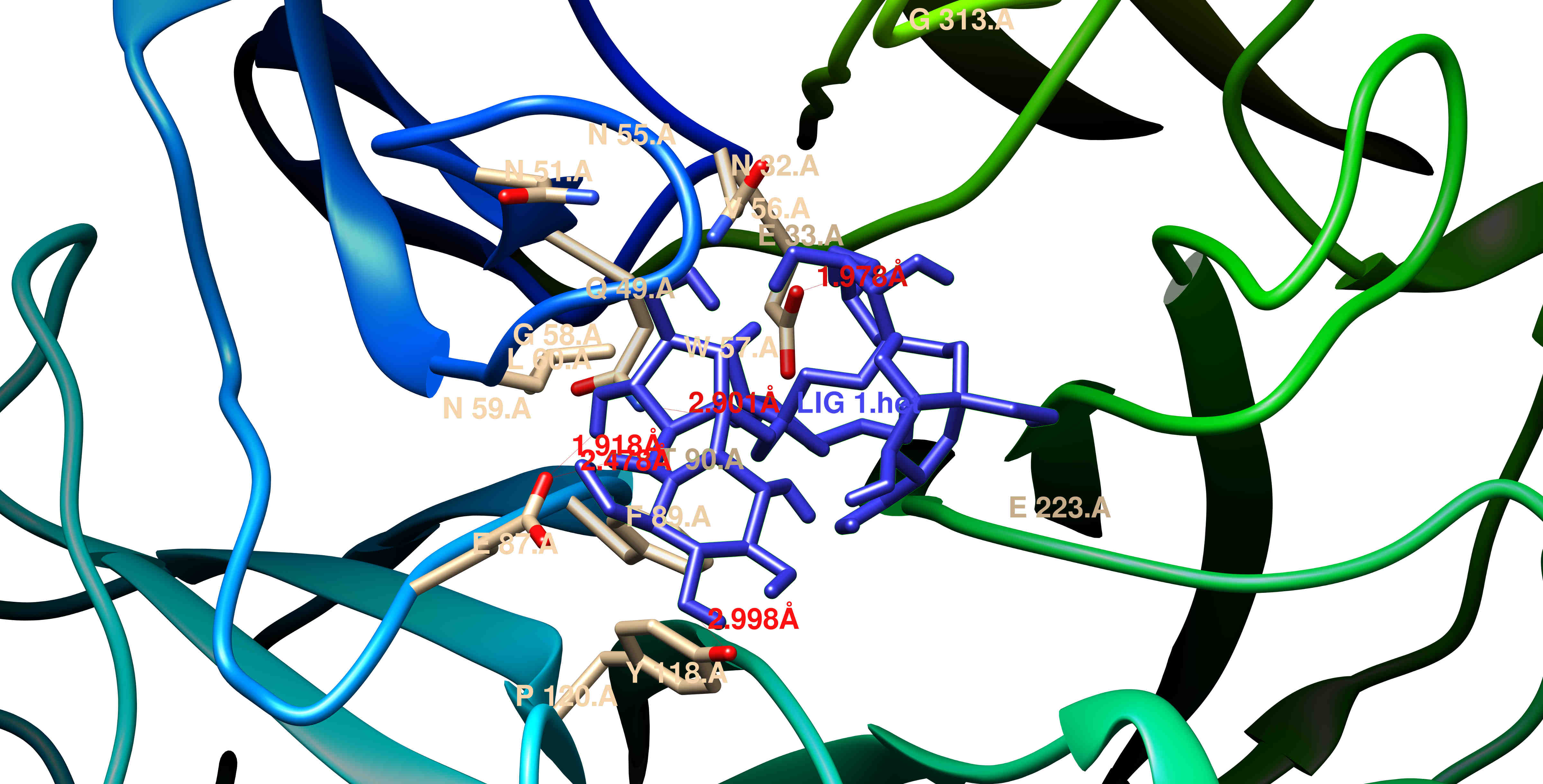

Supplement: S7 Dataset — (ZIP) [file pone.0200607.s007.zip › Docking_Images/ARP1_Docked.jpg]

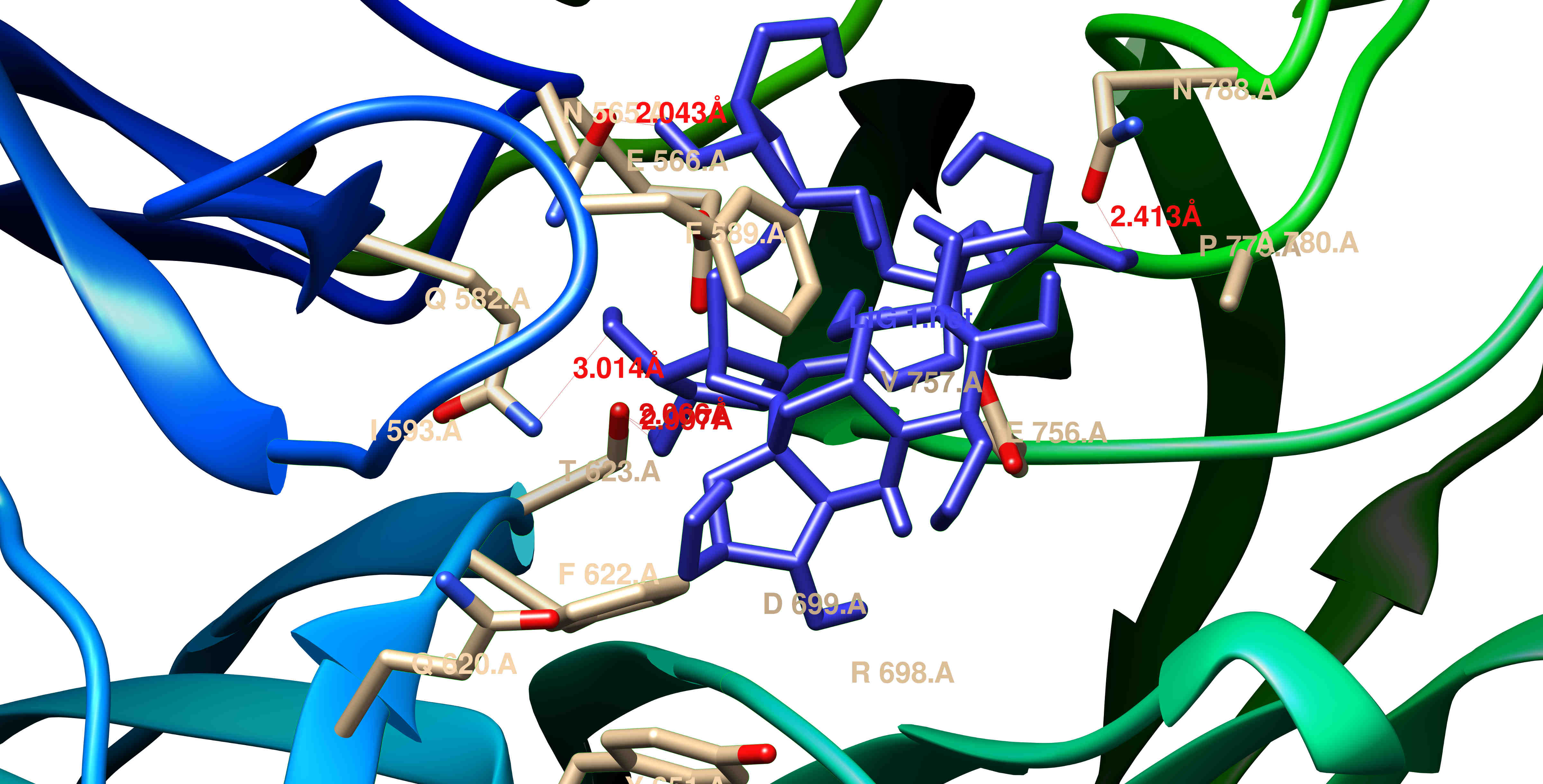

Supplement: S7 Dataset — (ZIP) [file pone.0200607.s007.zip › Docking_Images/BCP1_Docked.jpg]

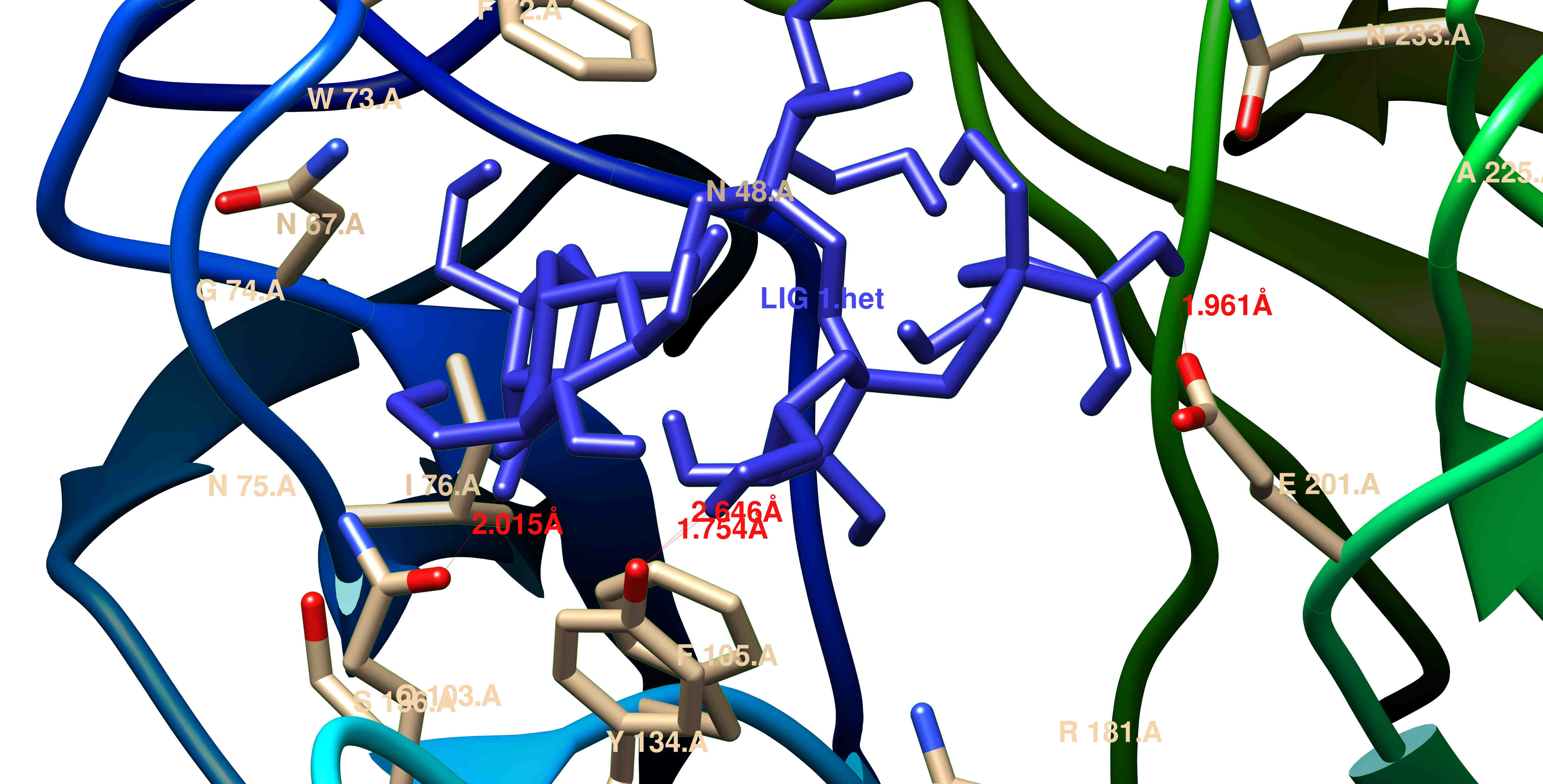

Supplement: S7 Dataset — (ZIP) [file pone.0200607.s007.zip › Docking_Images/BCP2_Docked.jpg]
