# Supplementary figures and images for: In Silico evaluation and identification of fungi capable of producing endo-inulinase enzyme (part 4 of 4)
Source: PLoS One. 2018 Jul 12;13(7):e0200607. doi: 10.1371/journal.pone.0200607 (PMC6042768; doi:10.1371/journal.pone.0200607)

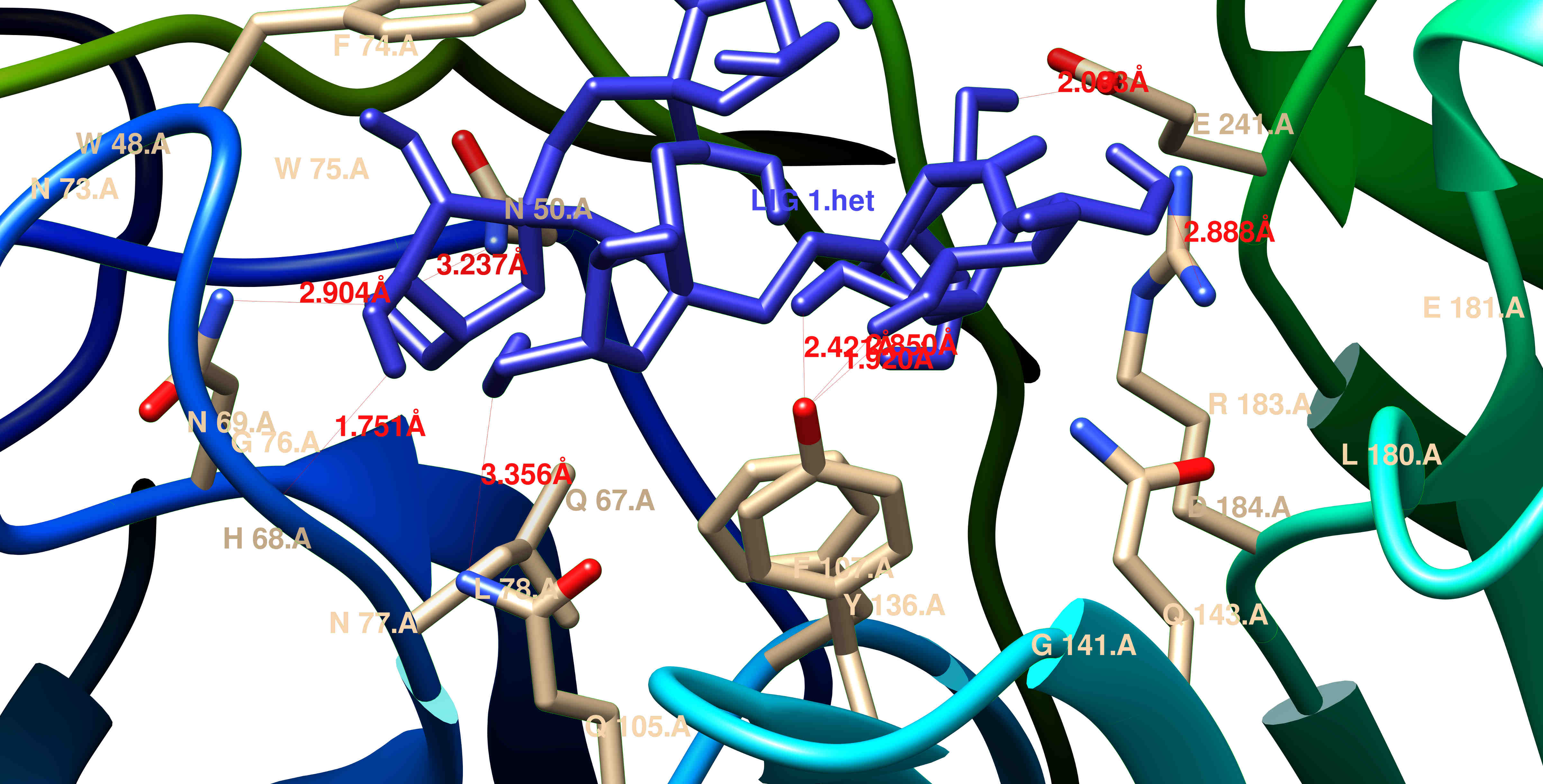

Supplement: S7 Dataset — (ZIP) [file pone.0200607.s007.zip › Docking_Images/FOHP1_Docked.jpg]

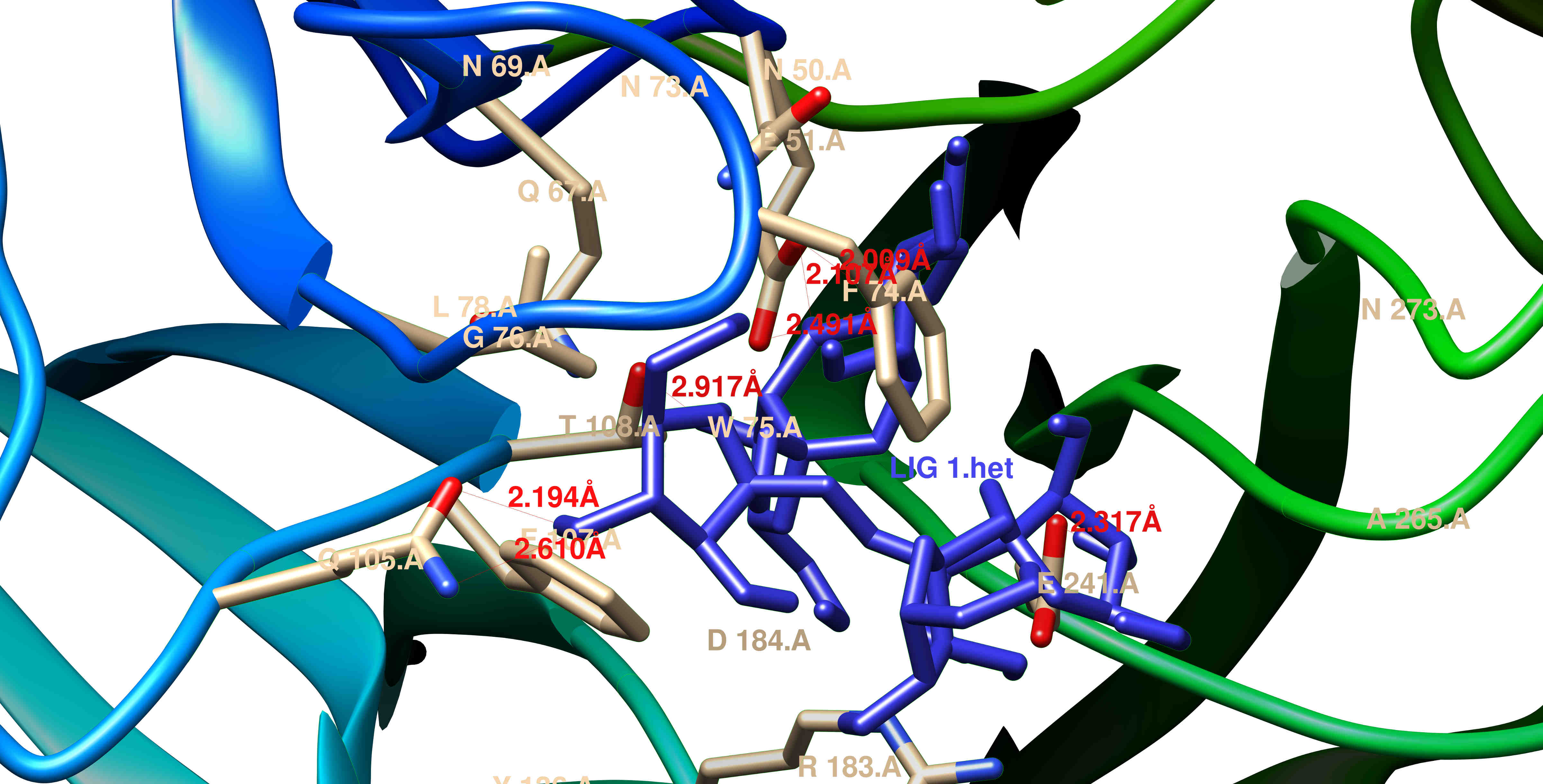

Supplement: S7 Dataset — (ZIP) [file pone.0200607.s007.zip › Docking_Images/FOHP7_Docked.jpg]

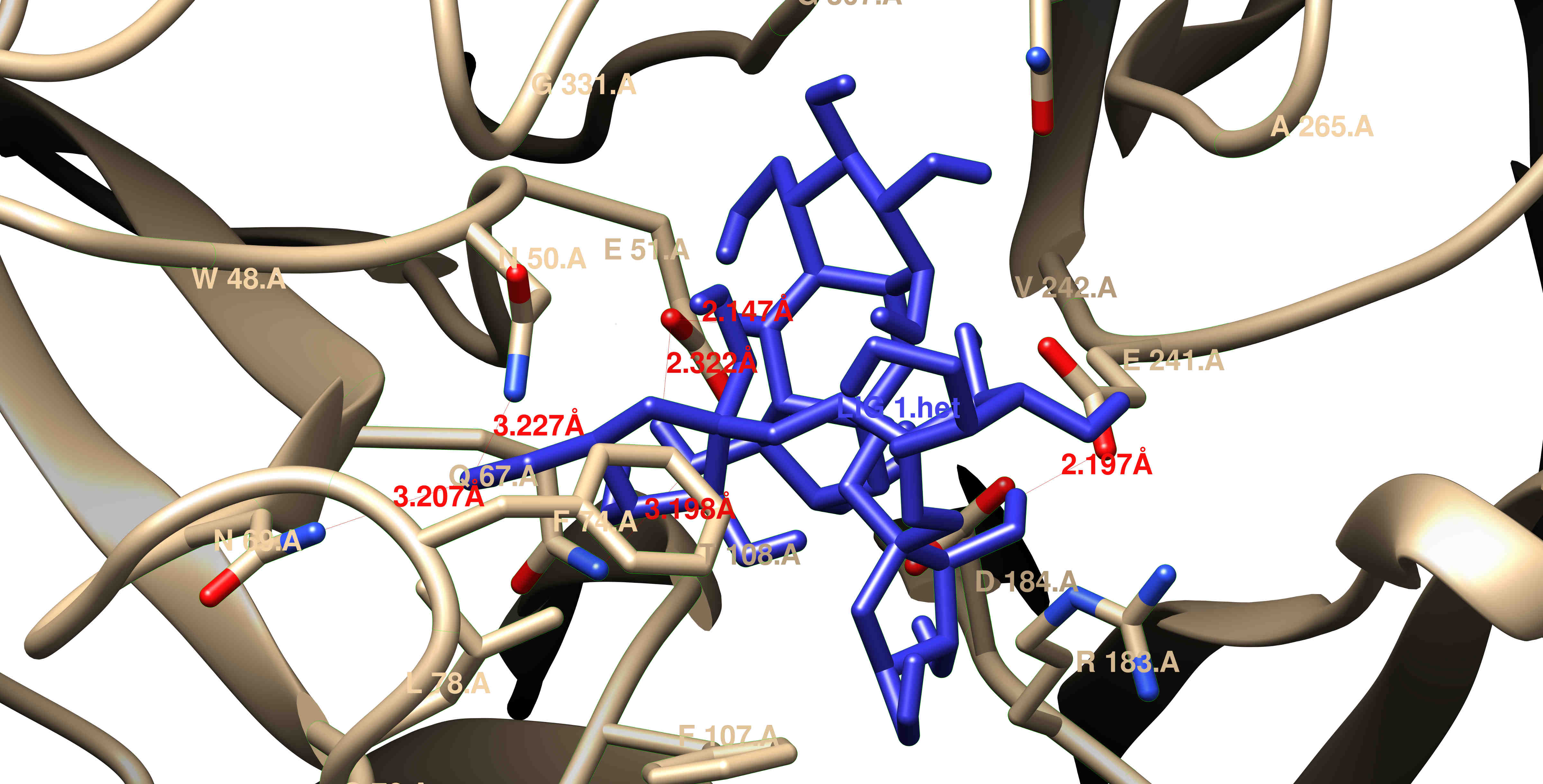

Supplement: S7 Dataset — (ZIP) [file pone.0200607.s007.zip › Docking_Images/FOP10_Docked.jpg]

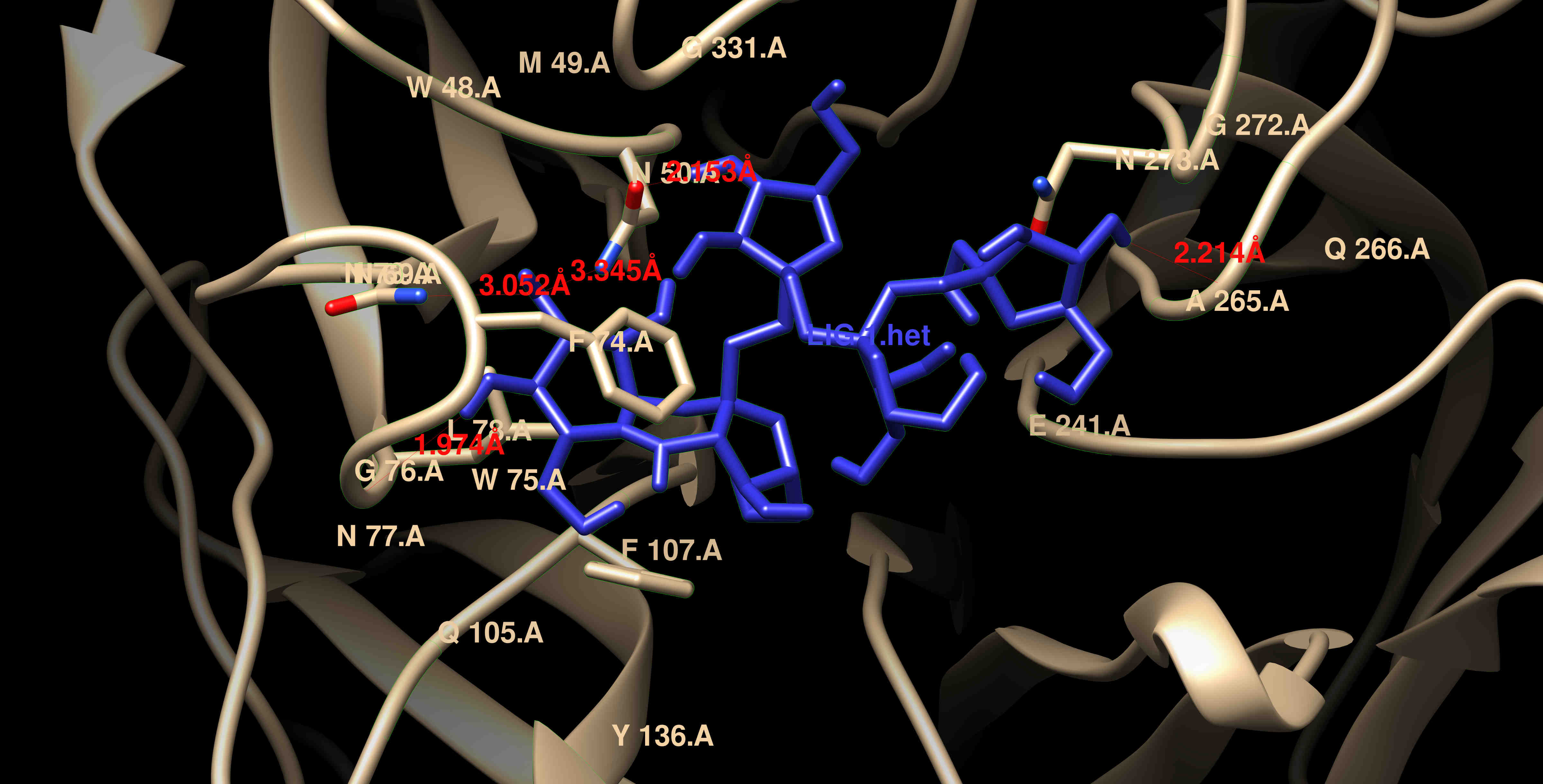

Supplement: S7 Dataset — (ZIP) [file pone.0200607.s007.zip › Docking_Images/FOP11_Docked.jpg]

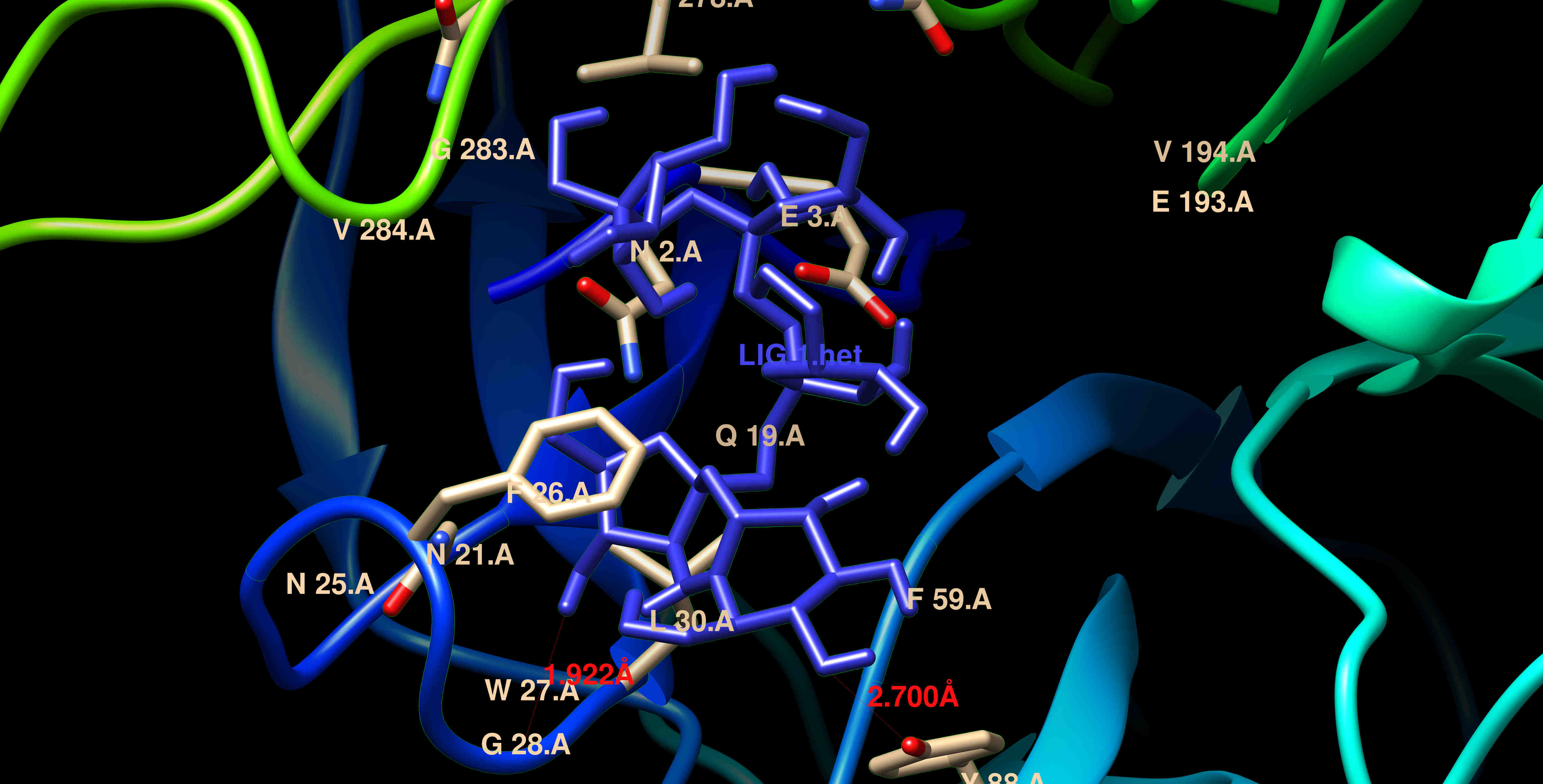

Supplement: S7 Dataset — (ZIP) [file pone.0200607.s007.zip › Docking_Images/FOP12_Docked.jpg]

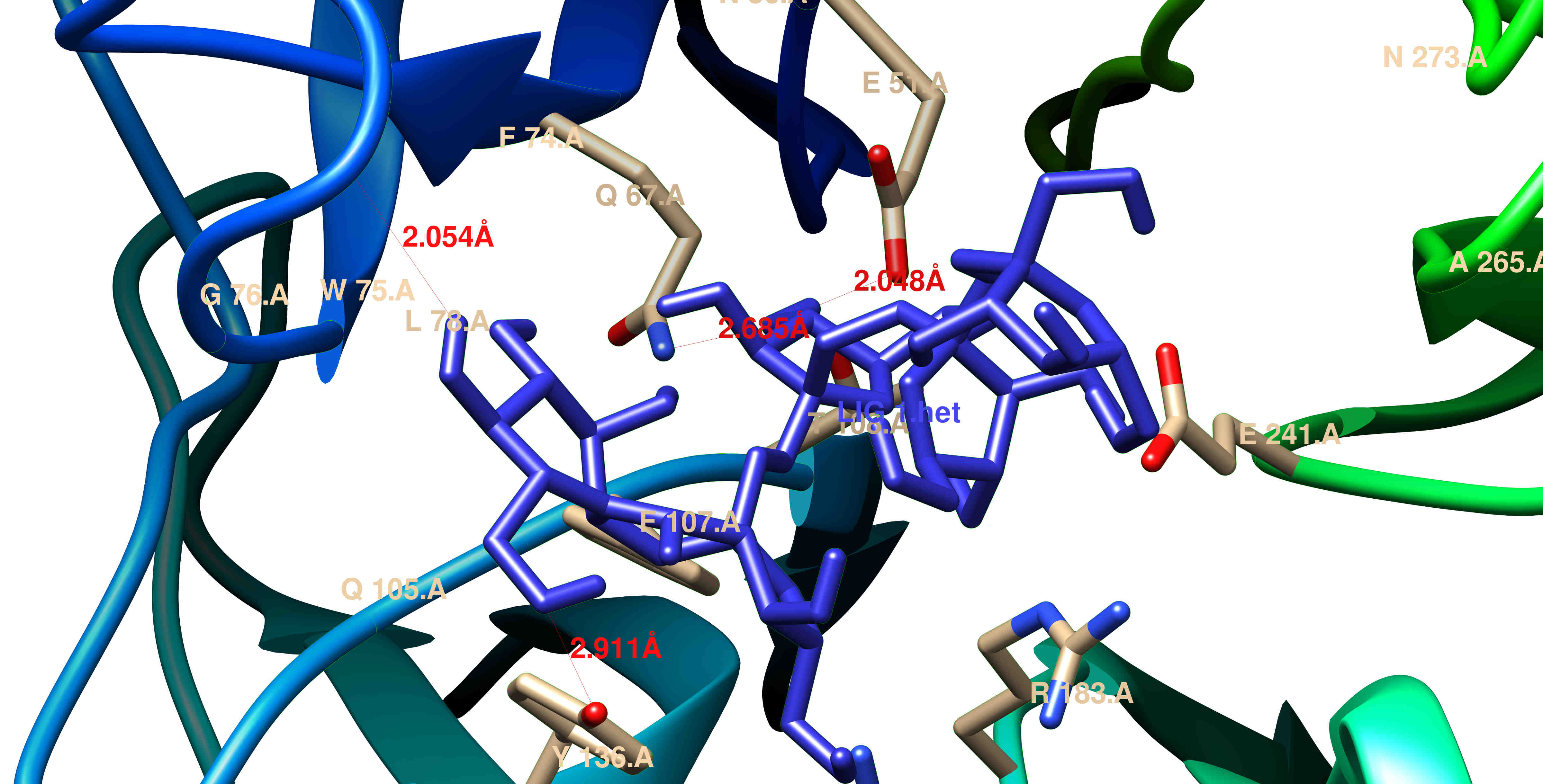

Supplement: S7 Dataset — (ZIP) [file pone.0200607.s007.zip › Docking_Images/FOP1_Docked.jpg]

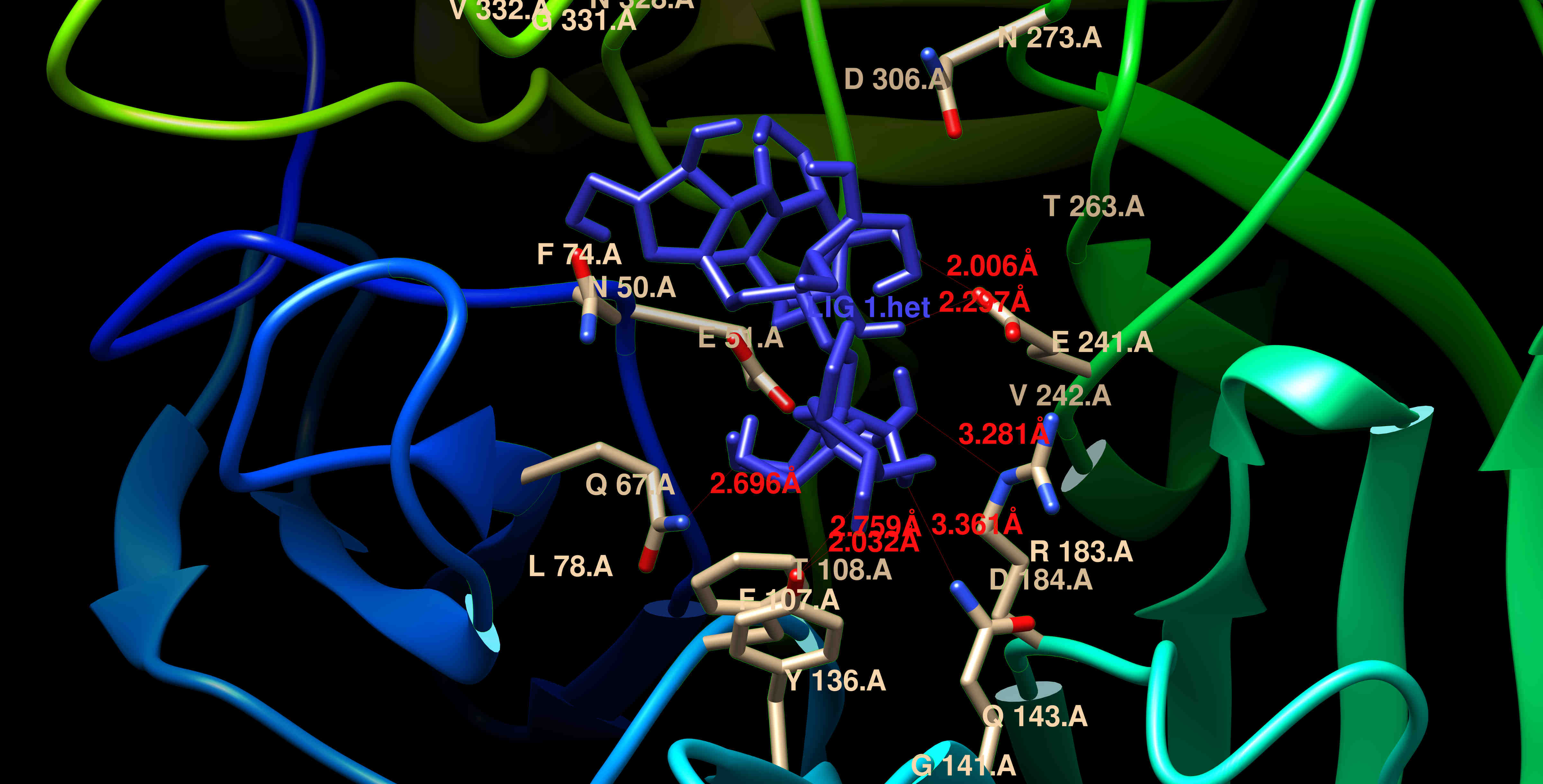

Supplement: S7 Dataset — (ZIP) [file pone.0200607.s007.zip › Docking_Images/FOP2_Docked.jpg]

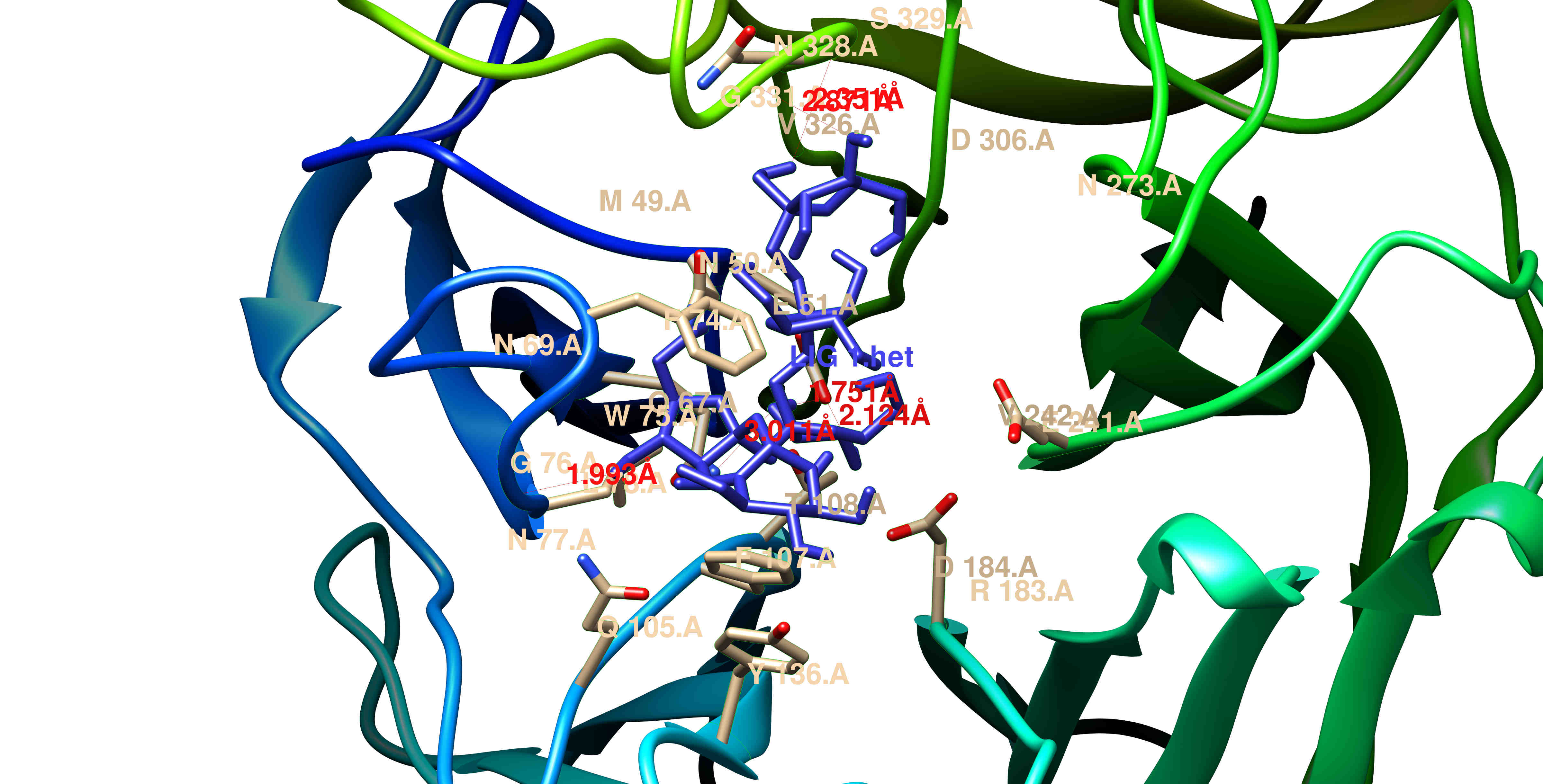

Supplement: S7 Dataset — (ZIP) [file pone.0200607.s007.zip › Docking_Images/FOP3_Docked.jpg]

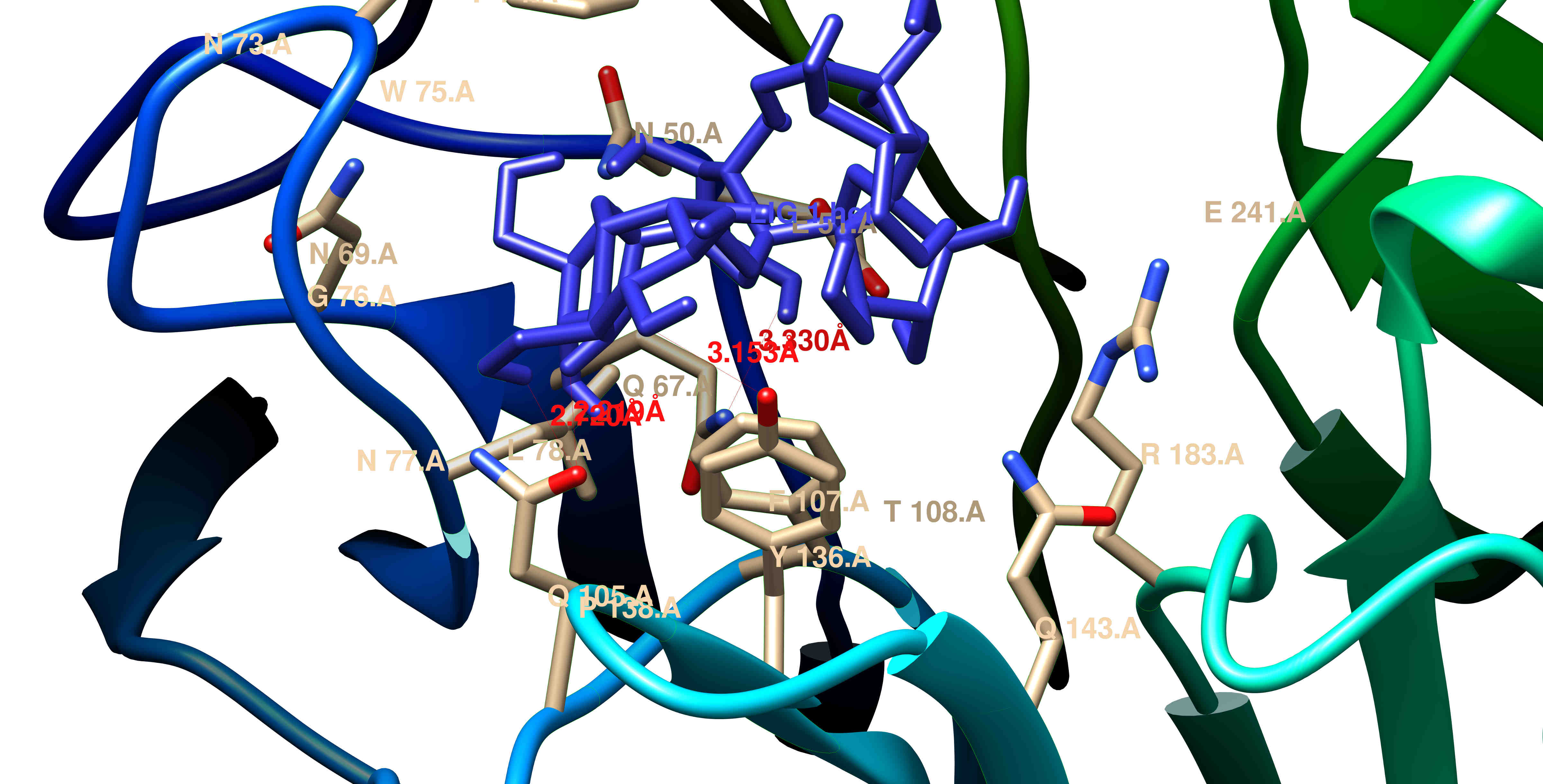

Supplement: S7 Dataset — (ZIP) [file pone.0200607.s007.zip › Docking_Images/FOP4_Docked.jpg]

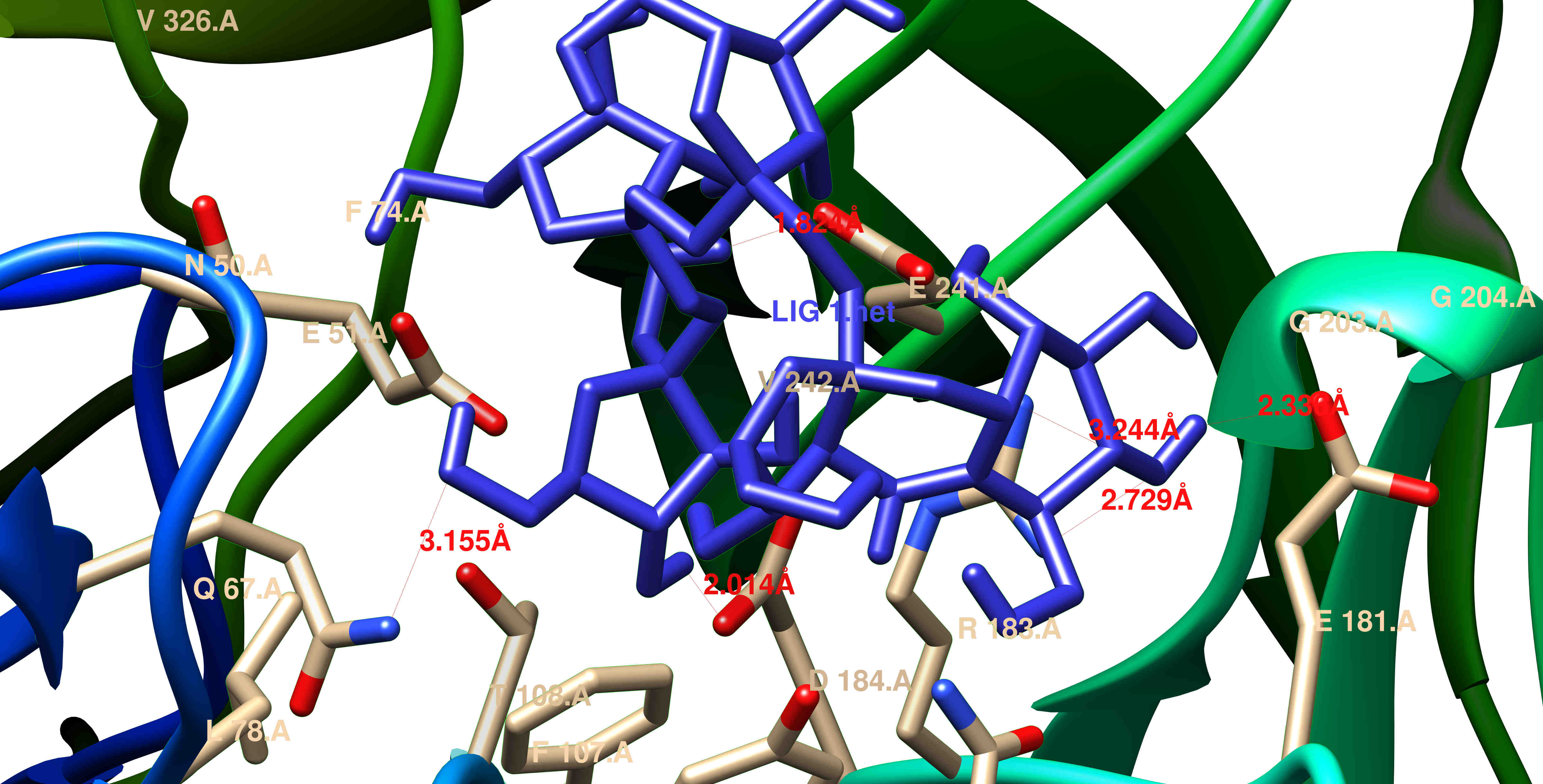

Supplement: S7 Dataset — (ZIP) [file pone.0200607.s007.zip › Docking_Images/FOP5_Docked.jpg]

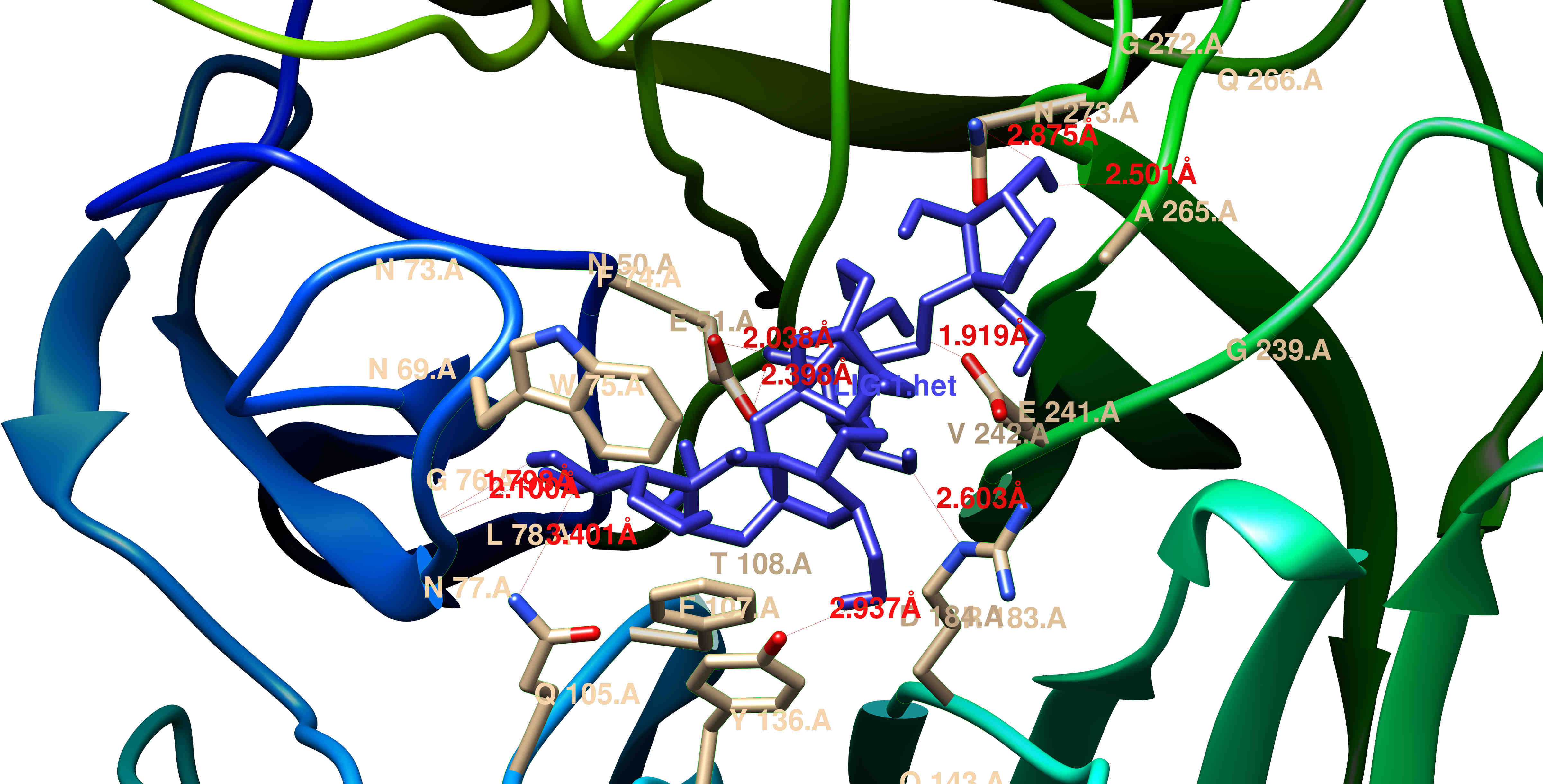

Supplement: S7 Dataset — (ZIP) [file pone.0200607.s007.zip › Docking_Images/FOP6_Docked.jpg]

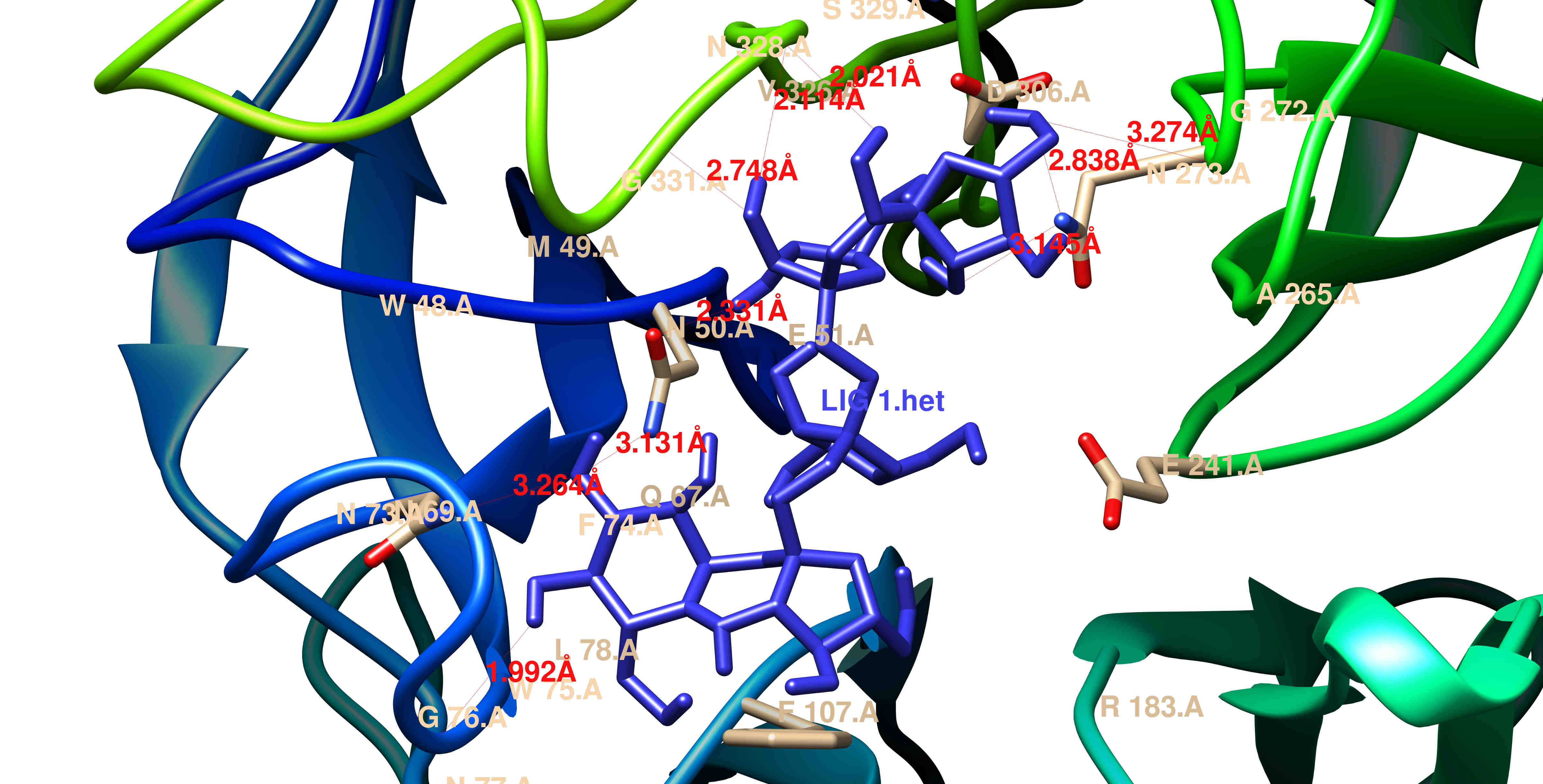

Supplement: S7 Dataset — (ZIP) [file pone.0200607.s007.zip › Docking_Images/FOP7_Docked.jpg]

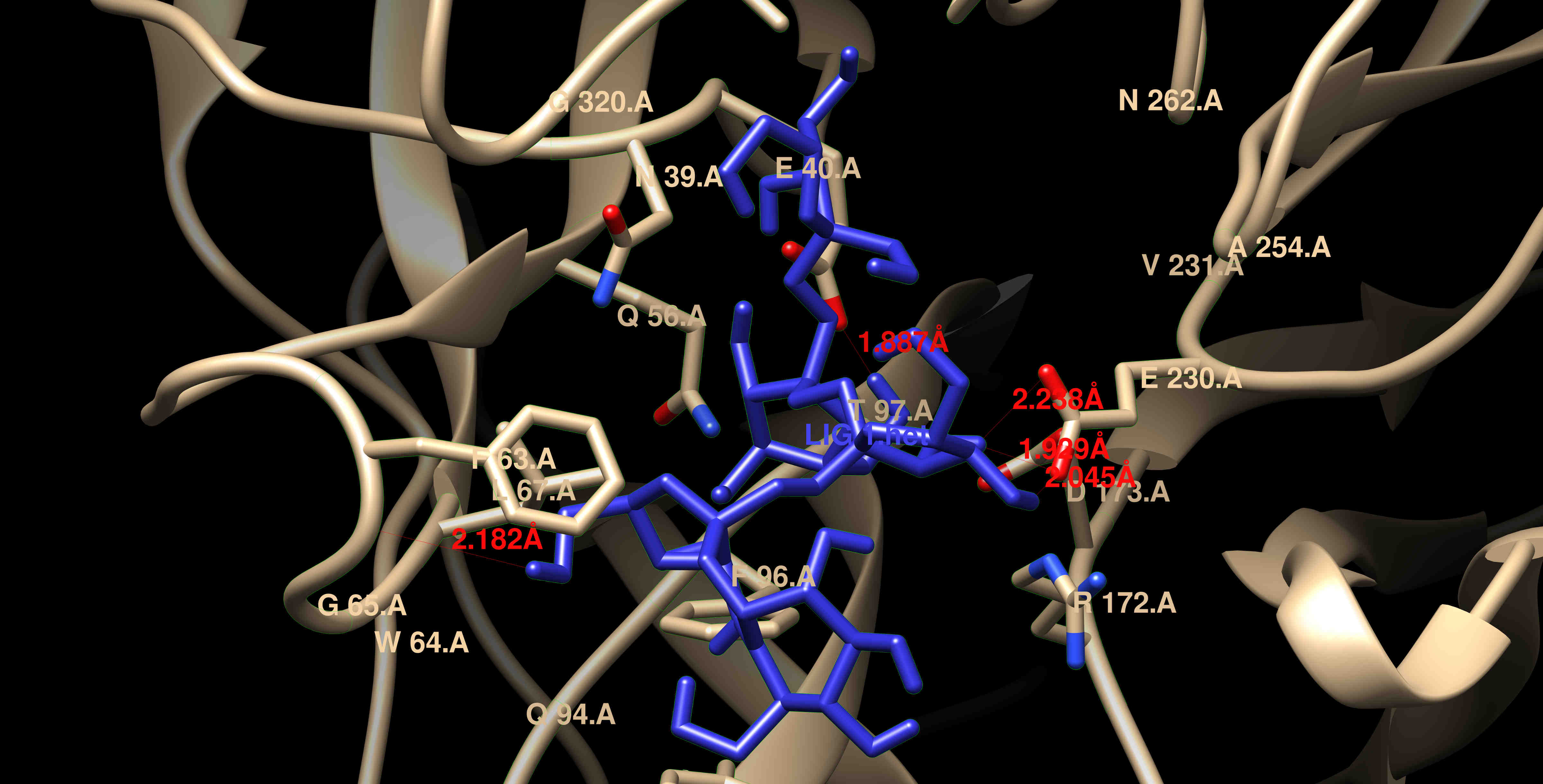

Supplement: S7 Dataset — (ZIP) [file pone.0200607.s007.zip › Docking_Images/FOP8_Docked.jpg]

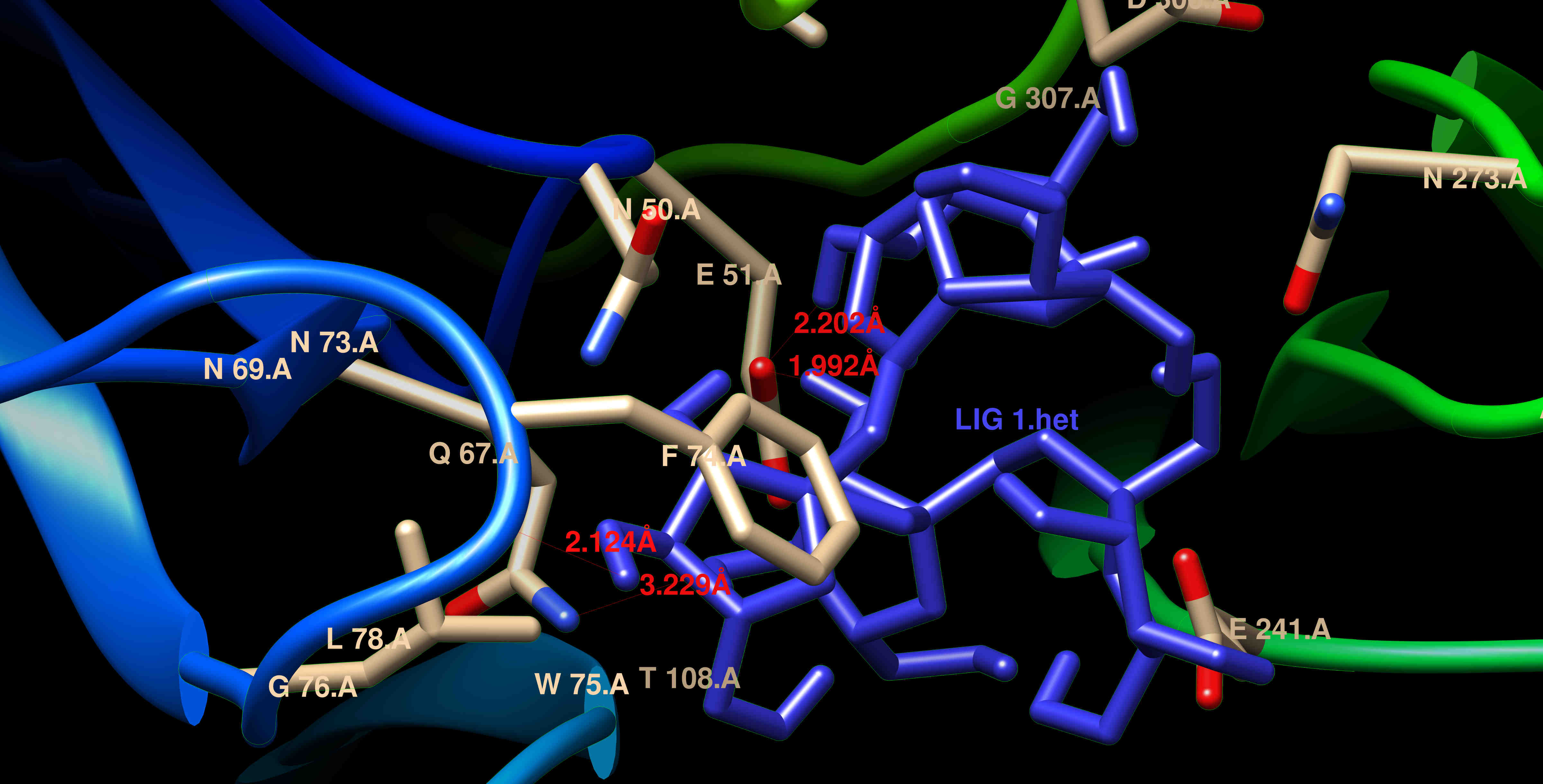

Supplement: S7 Dataset — (ZIP) [file pone.0200607.s007.zip › Docking_Images/FOP9_Docked.jpg]

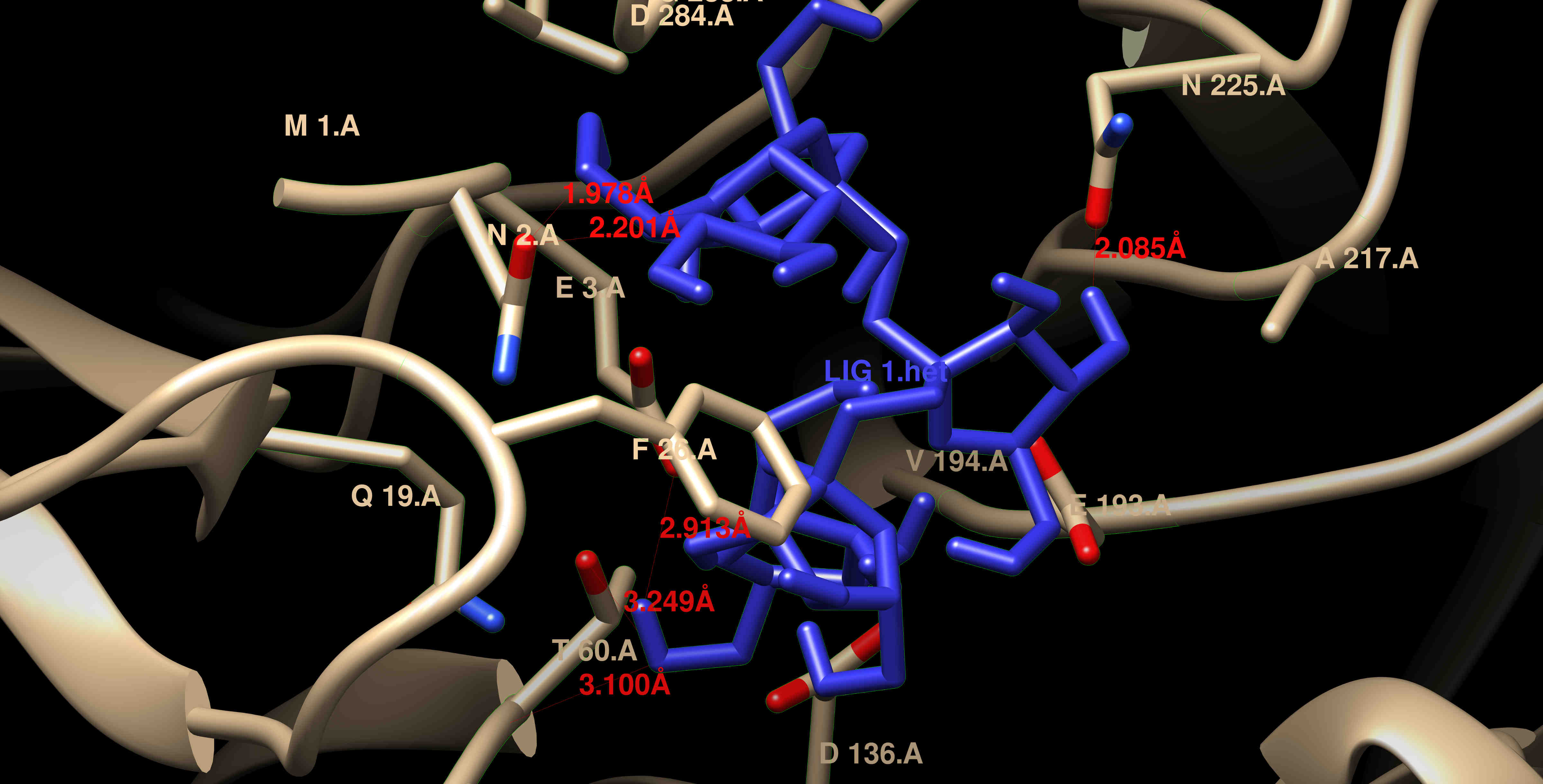

Supplement: S7 Dataset — (ZIP) [file pone.0200607.s007.zip › Docking_Images/MPP1_Docked.jpg]

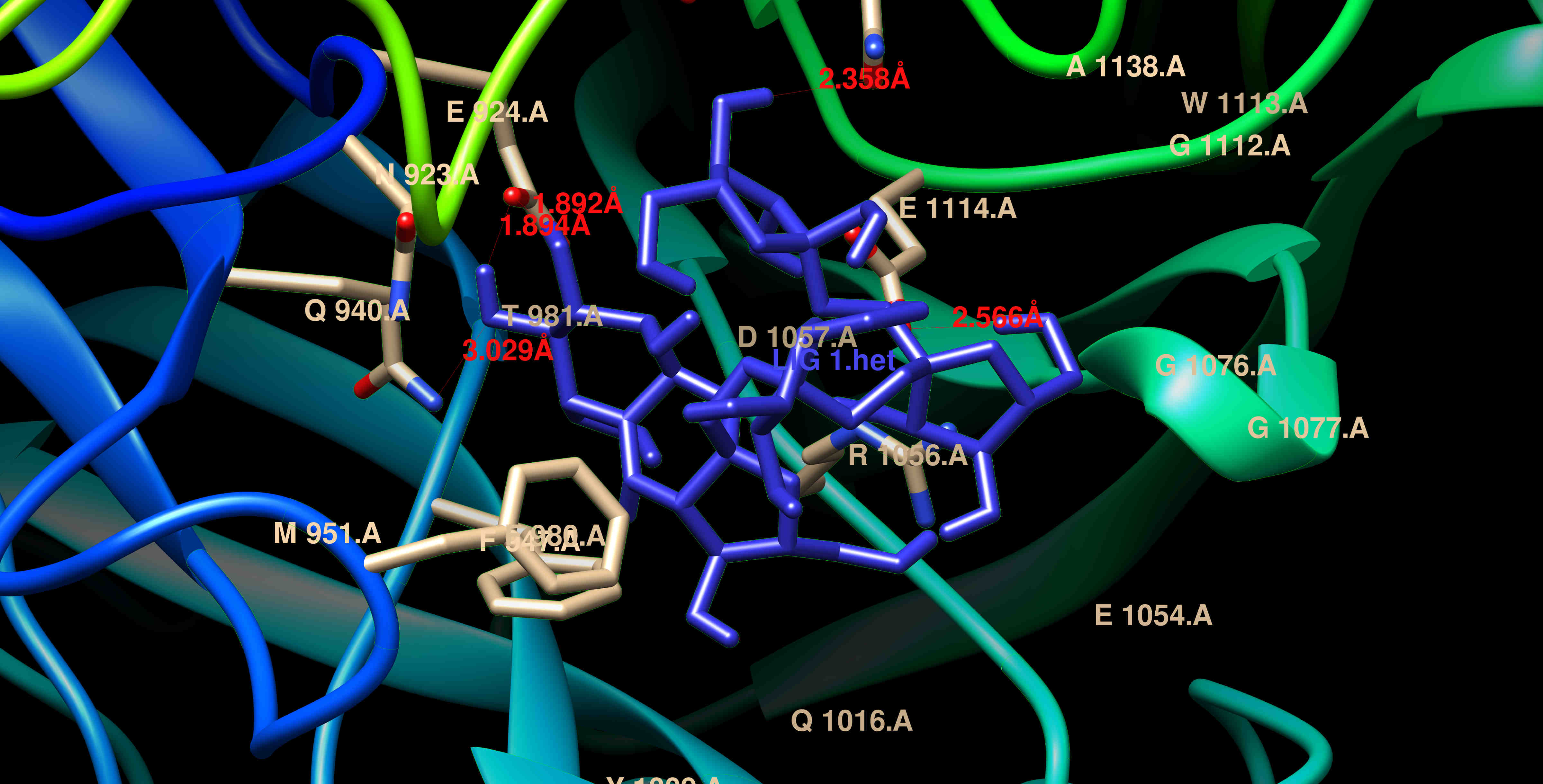

Supplement: S7 Dataset — (ZIP) [file pone.0200607.s007.zip › Docking_Images/OMP1_Docked.jpg]

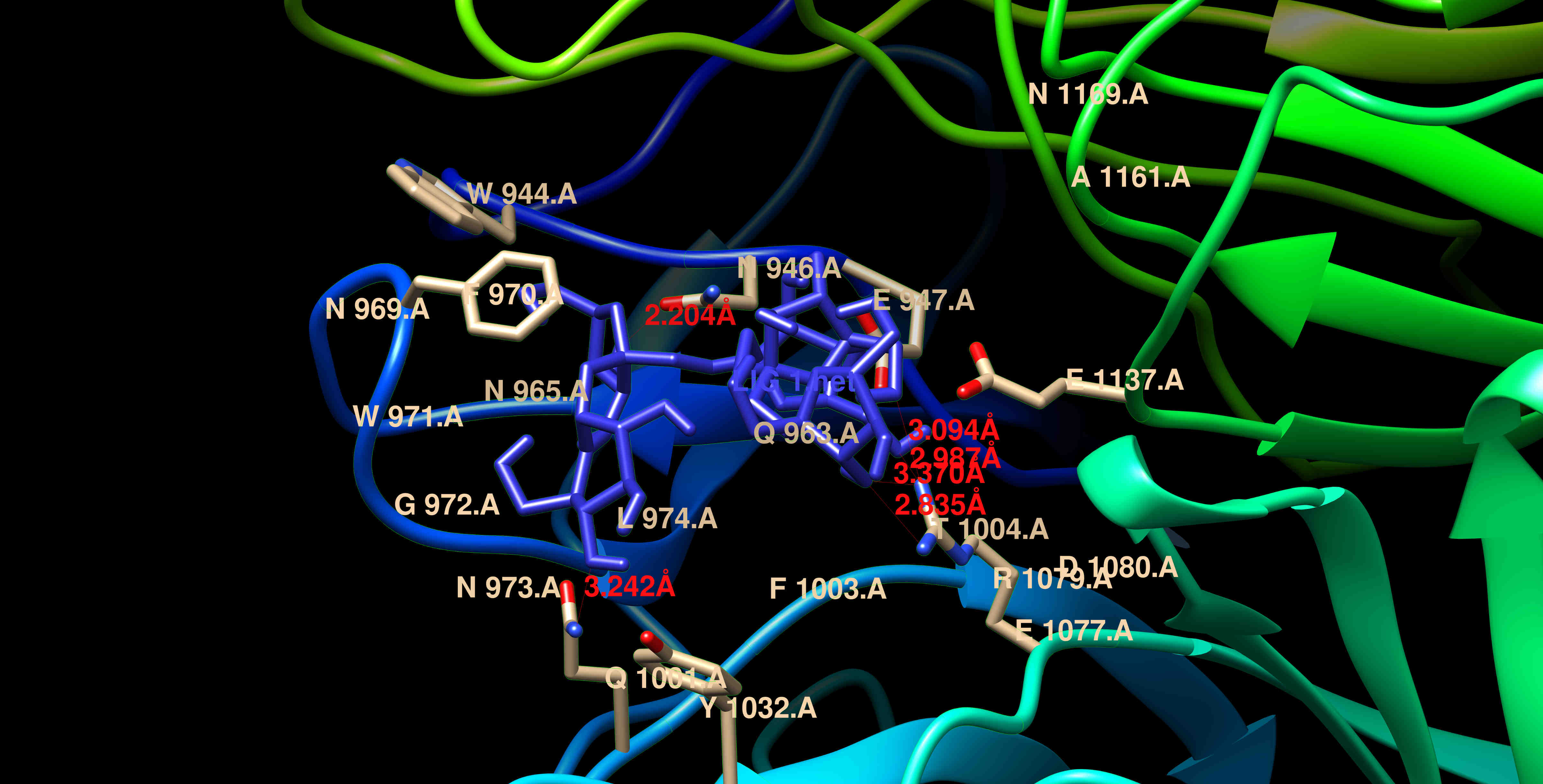

Supplement: S7 Dataset — (ZIP) [file pone.0200607.s007.zip › Docking_Images/PBP1_Docked.jpg]

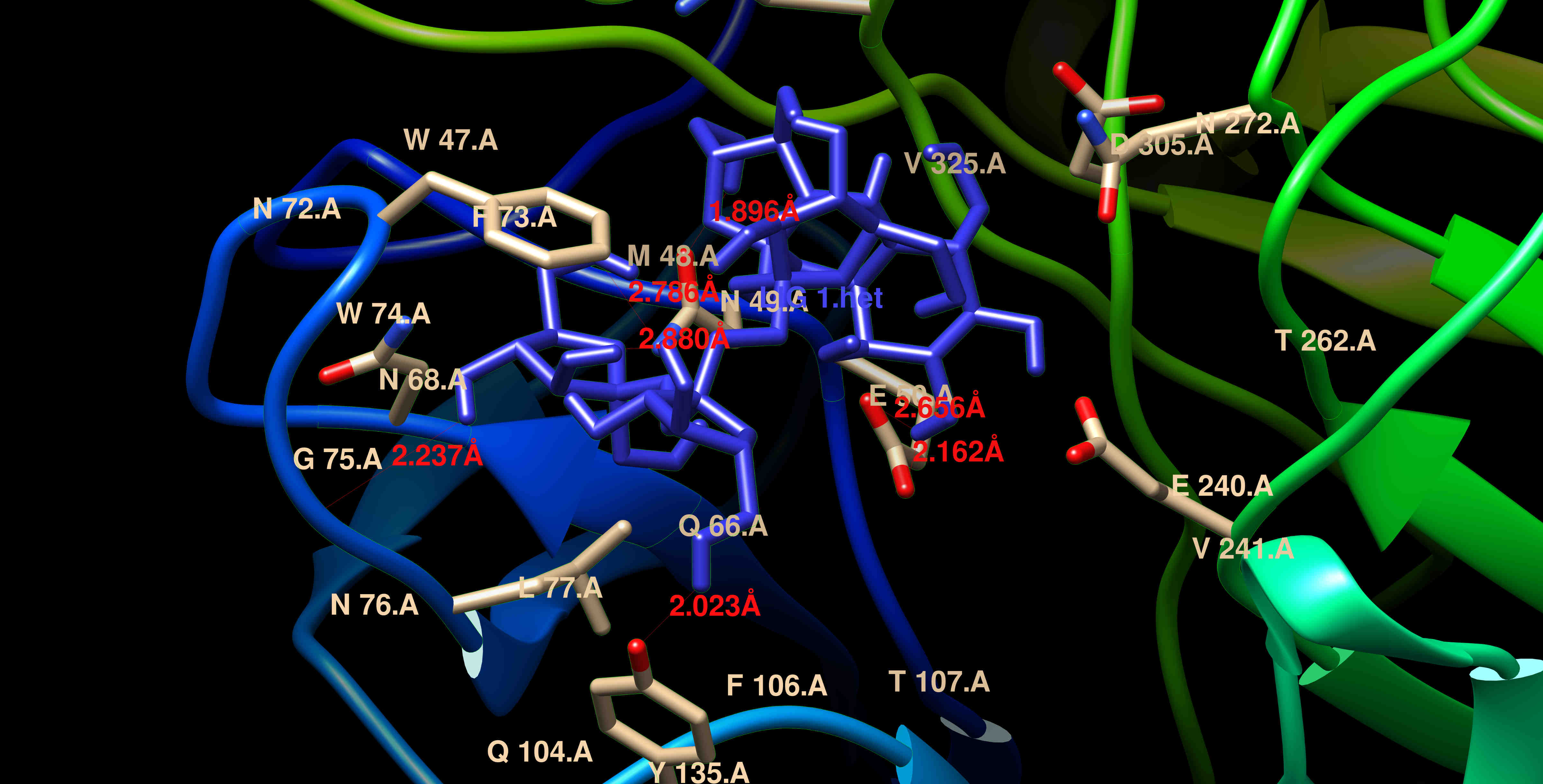

Supplement: S7 Dataset — (ZIP) [file pone.0200607.s007.zip › Docking_Images/PCP1_Docked.jpg]

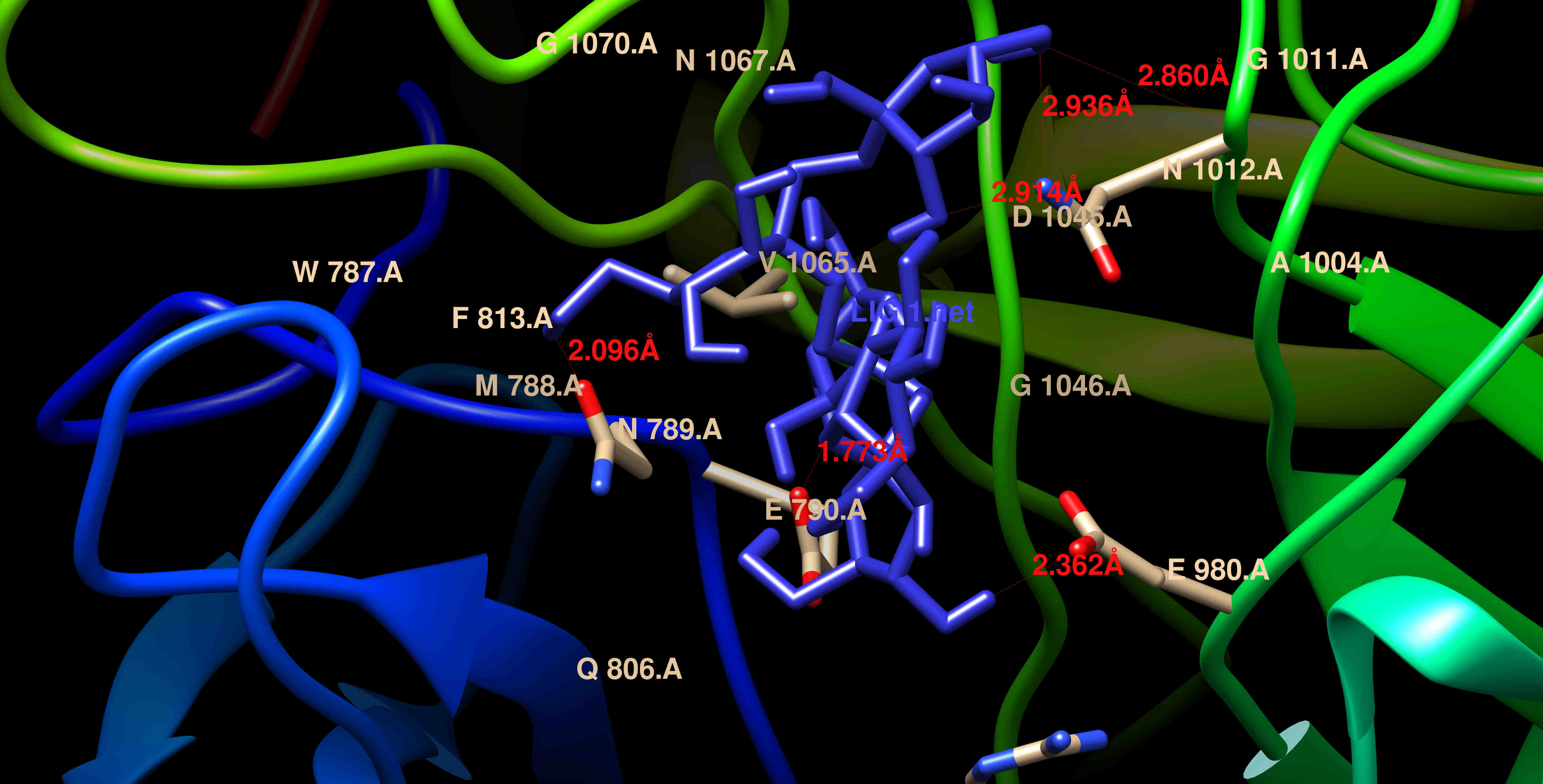

Supplement: S7 Dataset — (ZIP) [file pone.0200607.s007.zip › Docking_Images/PGP1_Docked.jpg]

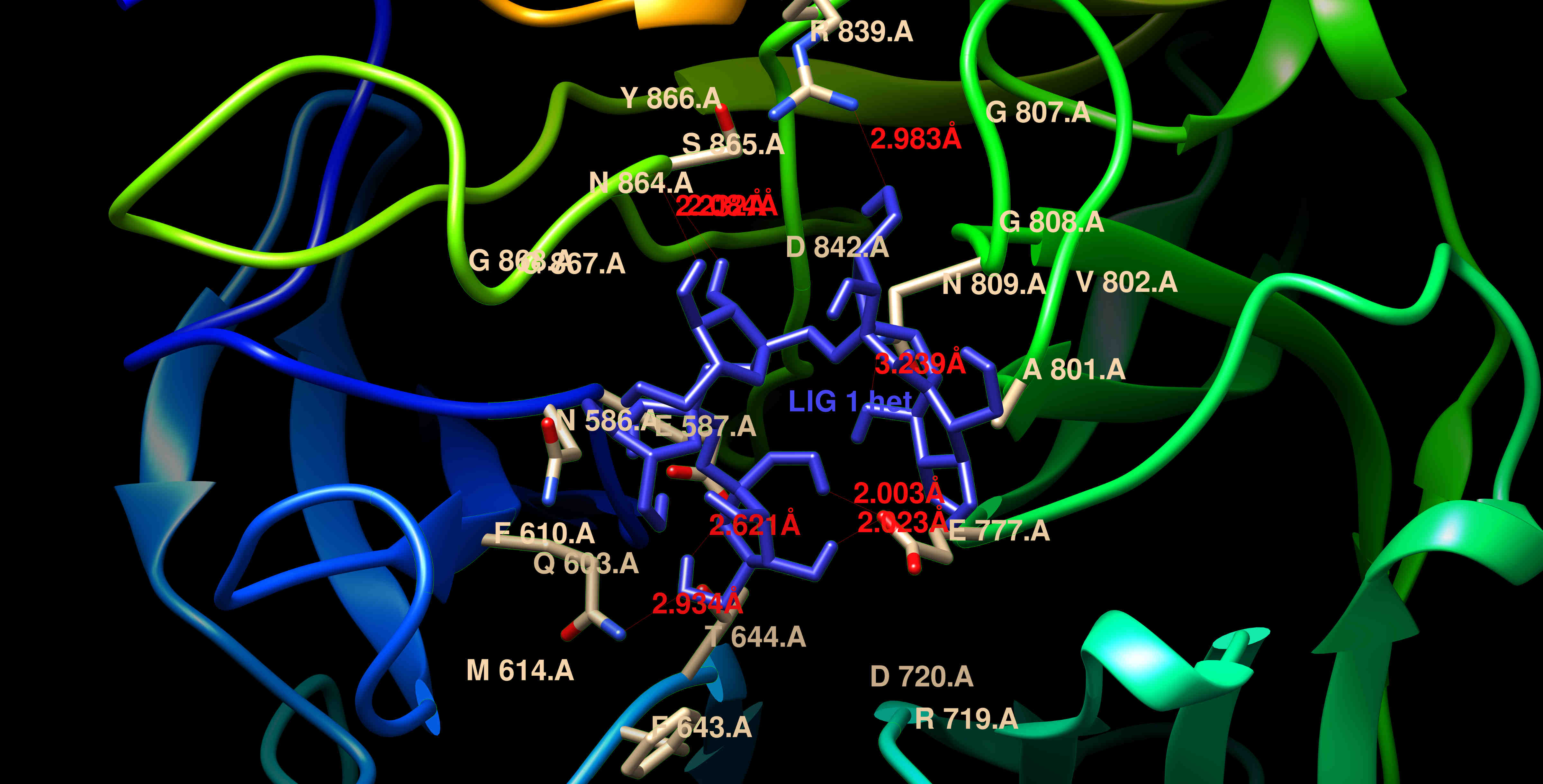

Supplement: S7 Dataset — (ZIP) [file pone.0200607.s007.zip › Docking_Images/PGP2_Docked.jpg]

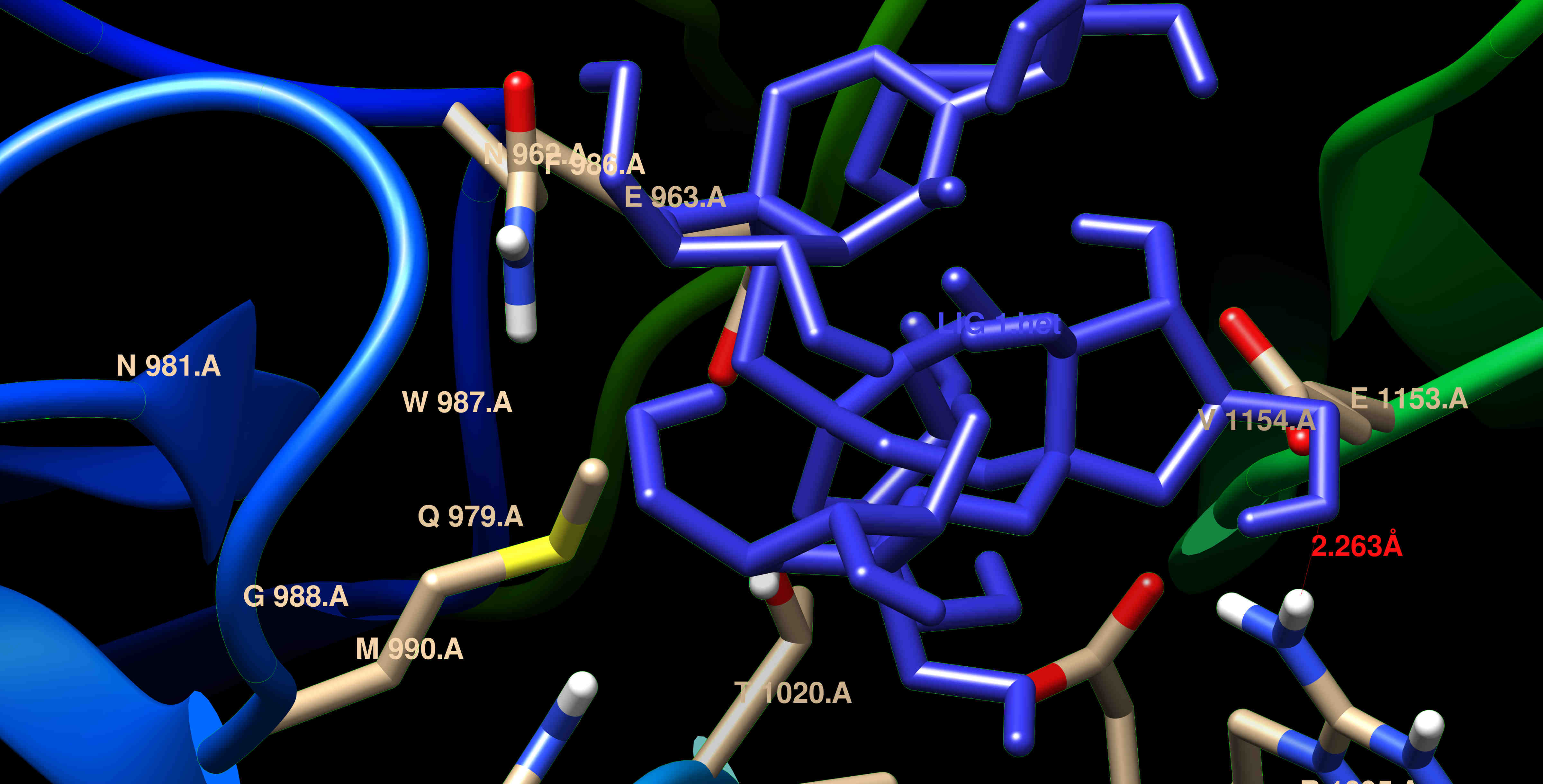

Supplement: S7 Dataset — (ZIP) [file pone.0200607.s007.zip › Docking_Images/PGP3_Docked.jpg]

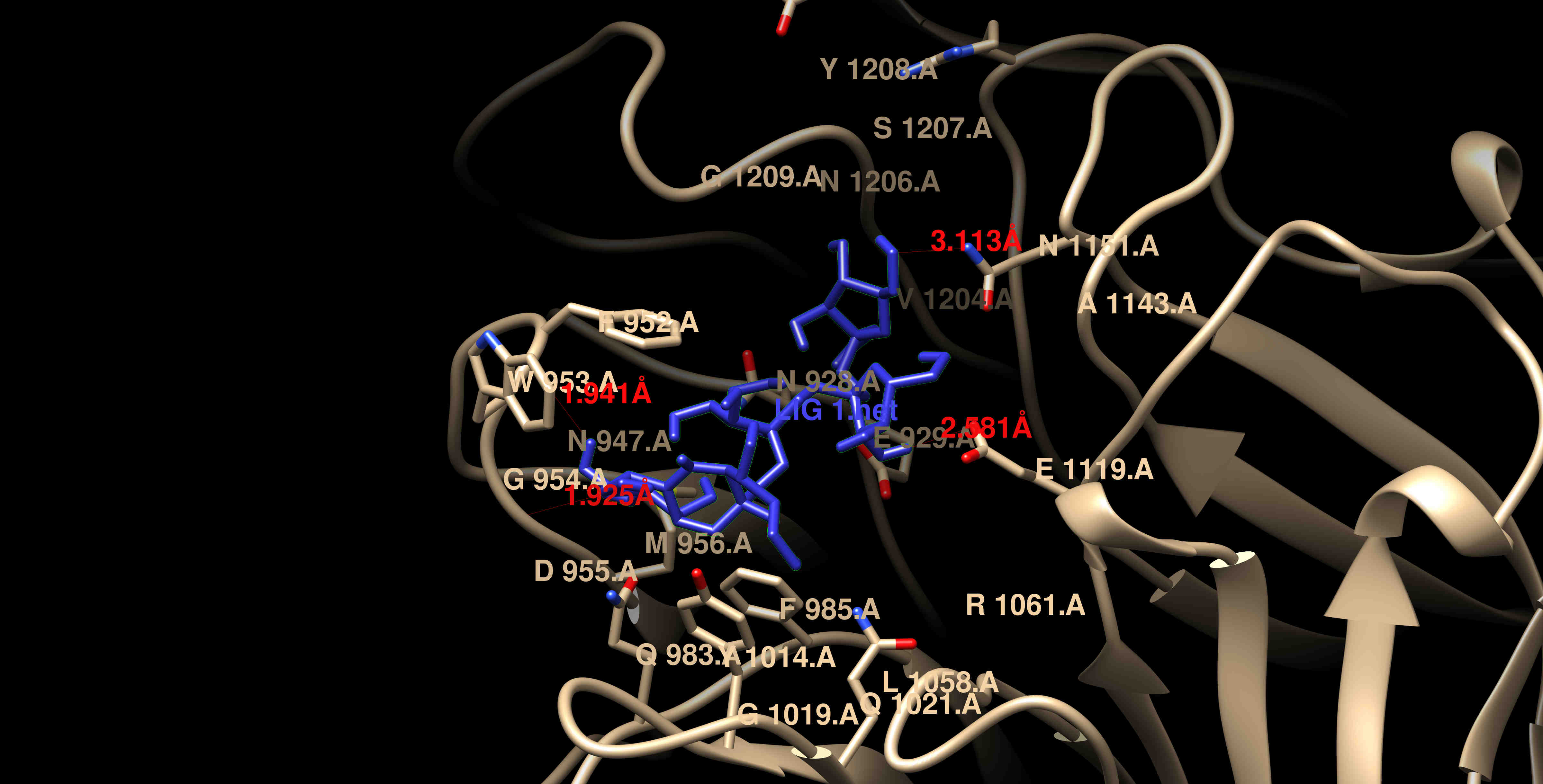

Supplement: S7 Dataset — (ZIP) [file pone.0200607.s007.zip › Docking_Images/PGP4_Docked.jpg]

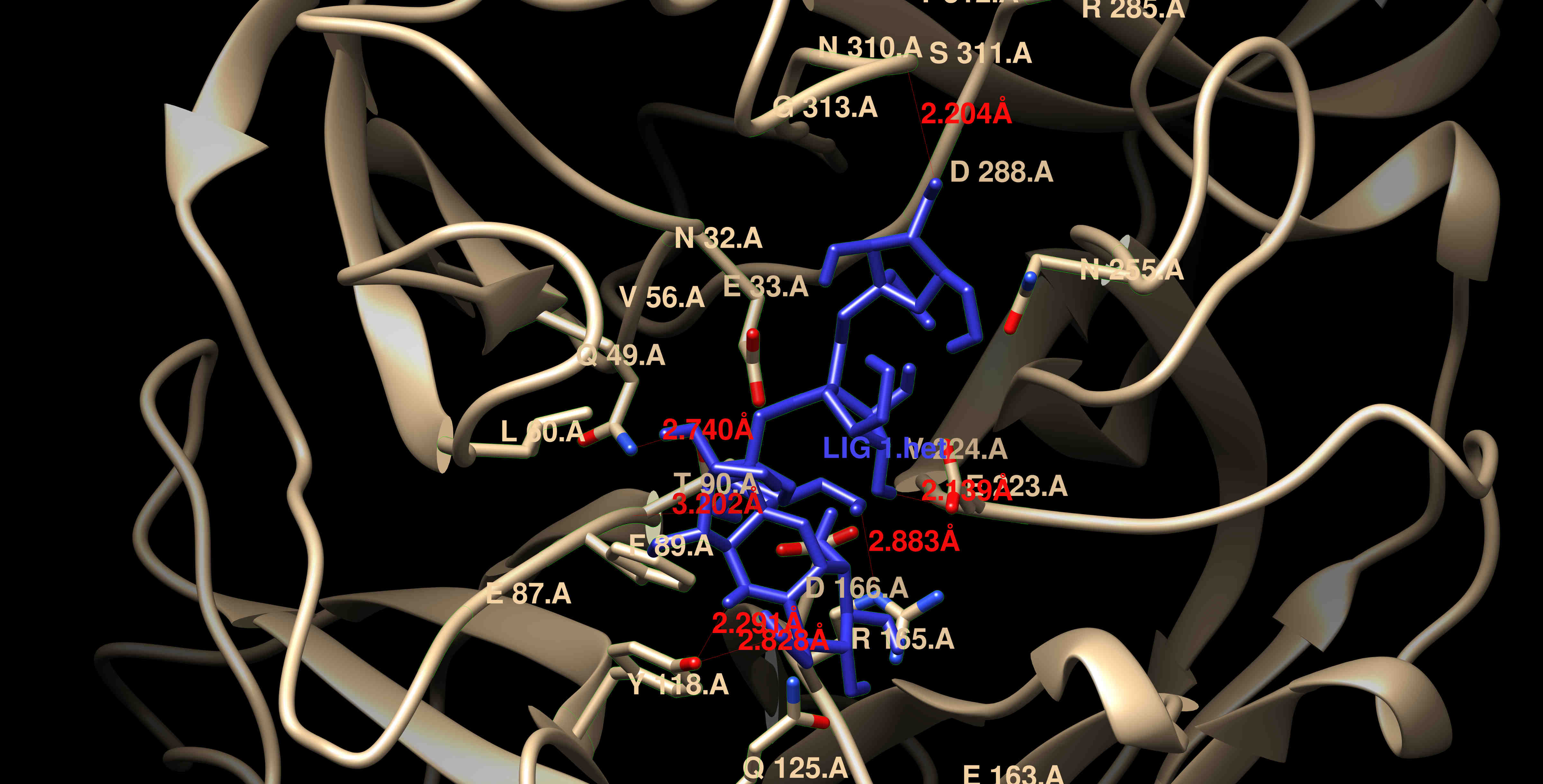

Supplement: S7 Dataset — (ZIP) [file pone.0200607.s007.zip › Docking_Images/PNP1_Docked.jpg]

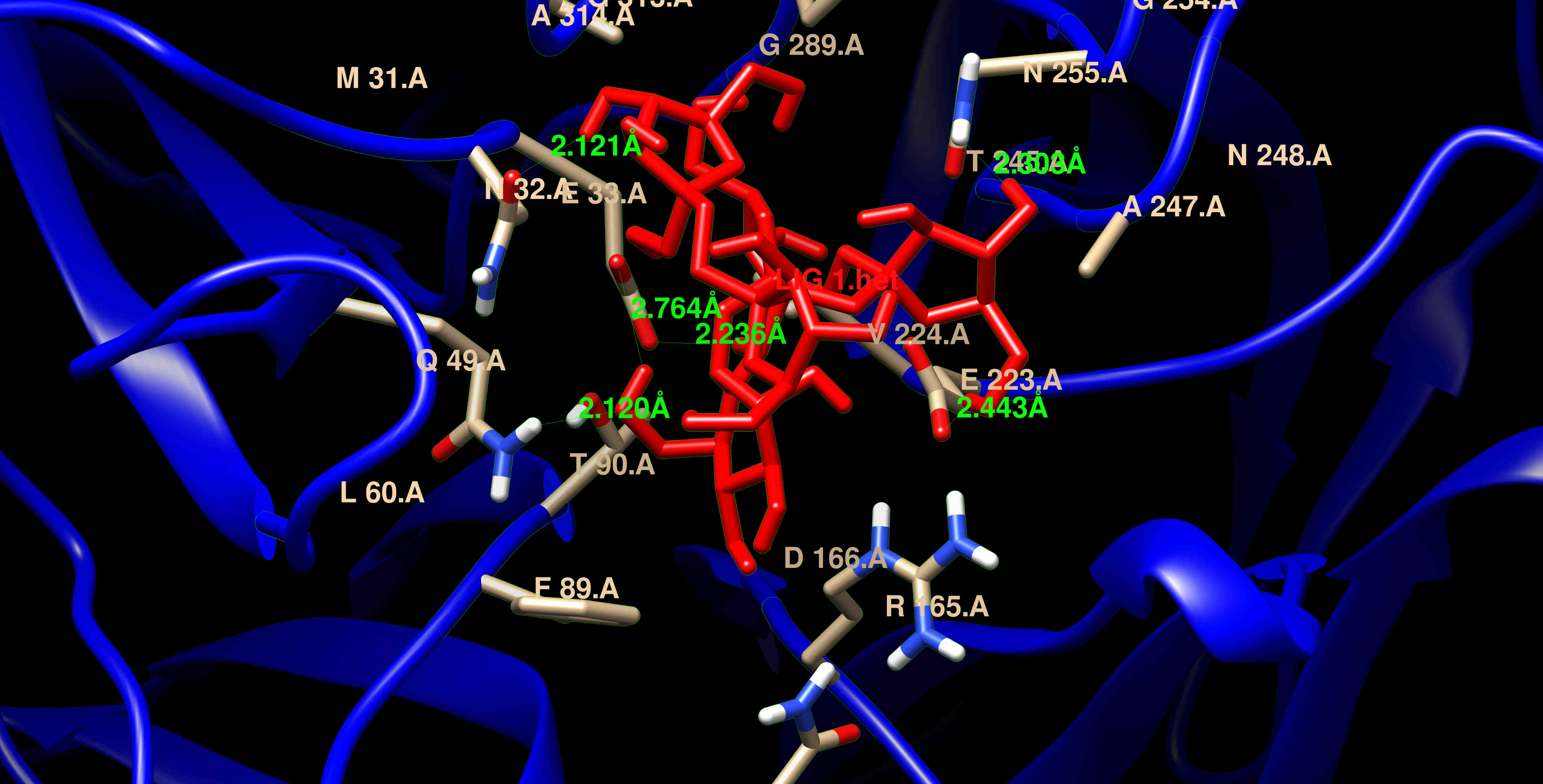

Supplement: S7 Dataset — (ZIP) [file pone.0200607.s007.zip › Docking_Images/PSP1_Docked.jpg]

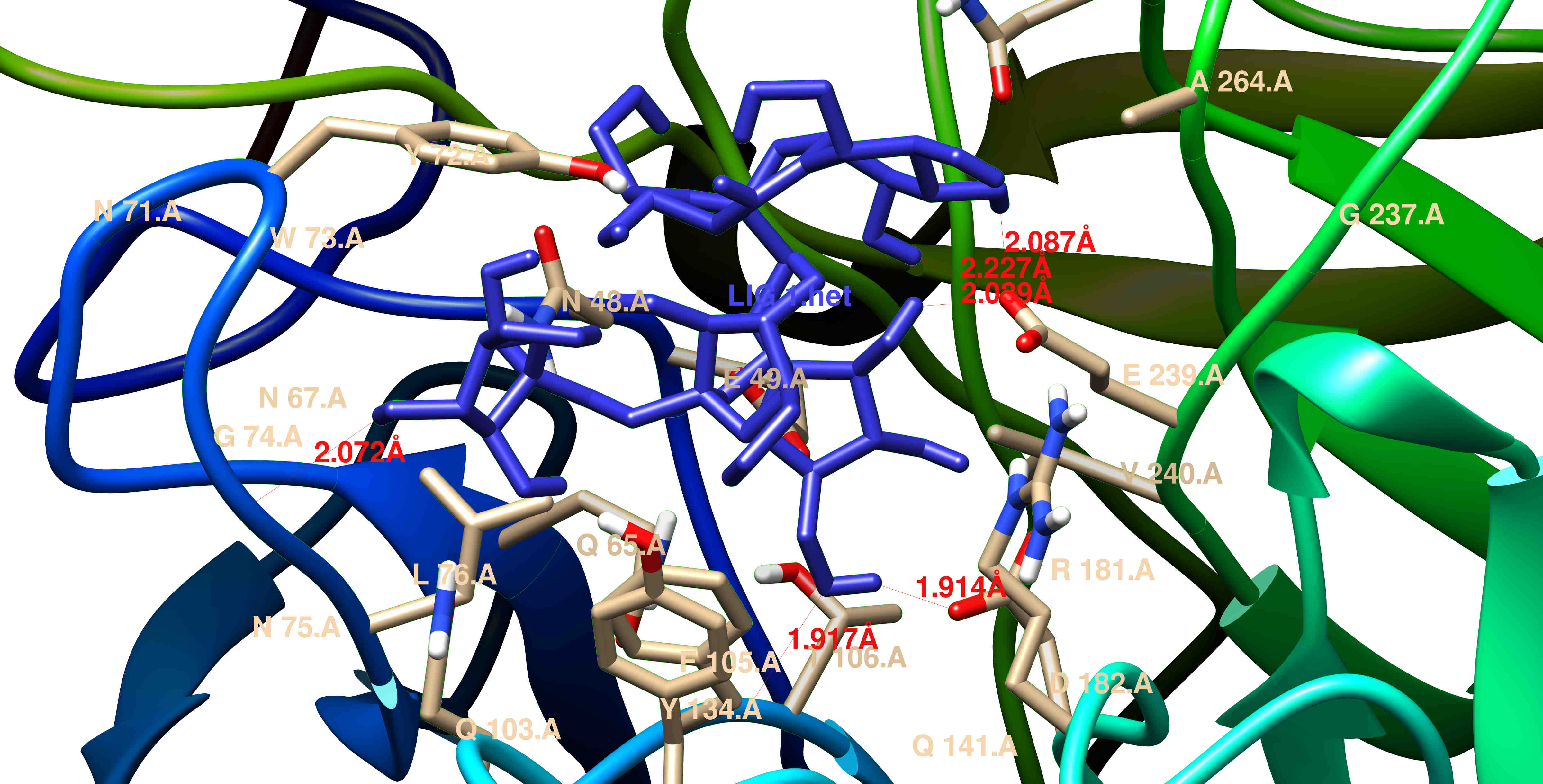

Supplement: S7 Dataset — (ZIP) [file pone.0200607.s007.zip › Docking_Images/PSP2_Docked.jpg]

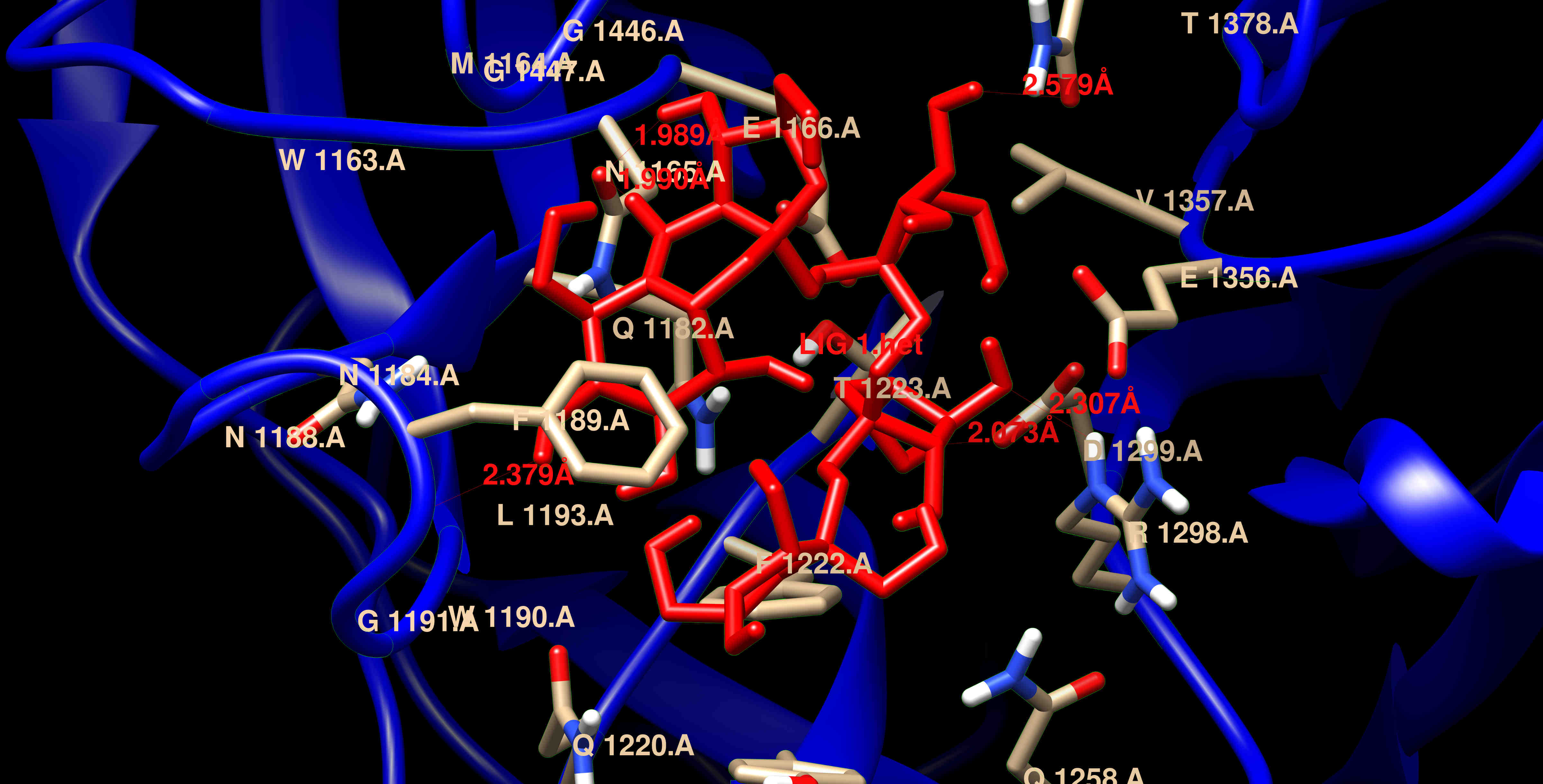

Supplement: S7 Dataset — (ZIP) [file pone.0200607.s007.zip › Docking_Images/PSP3_Docked.jpg]

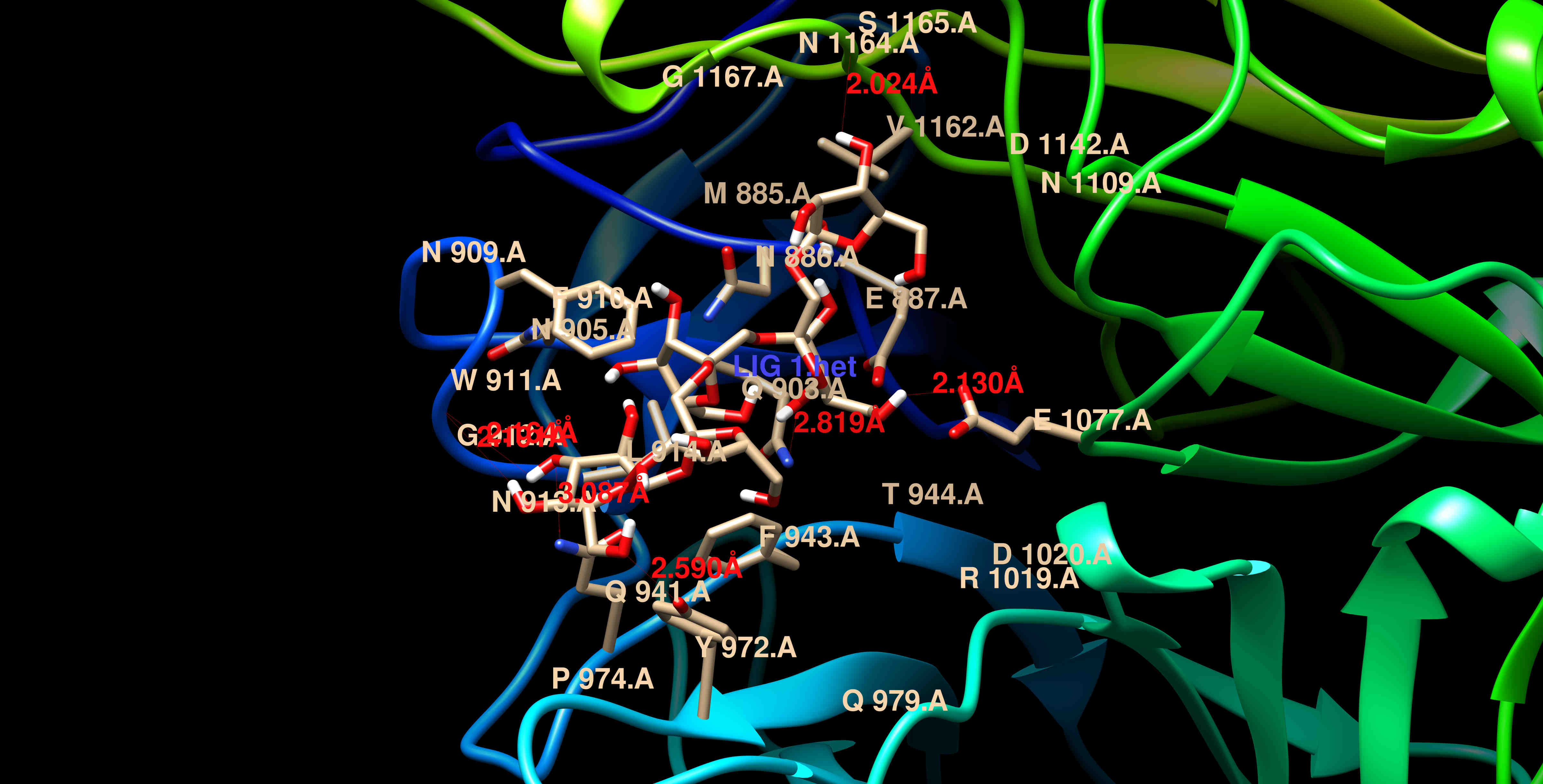

Supplement: S7 Dataset — (ZIP) [file pone.0200607.s007.zip › Docking_Images/SCHP1_Docked.jpg]

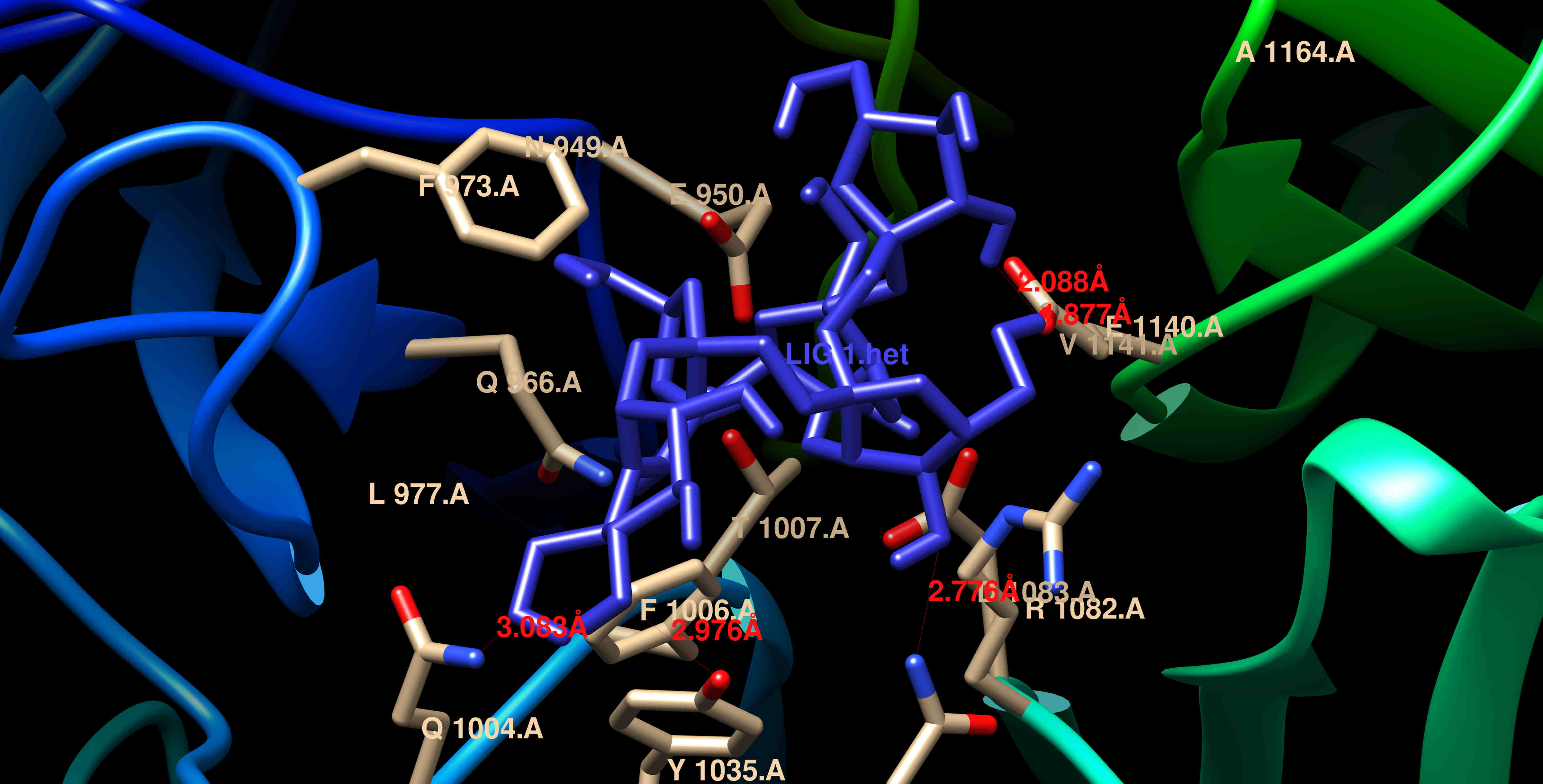

Supplement: S7 Dataset — (ZIP) [file pone.0200607.s007.zip › Docking_Images/SCHP2_Docked.jpg]

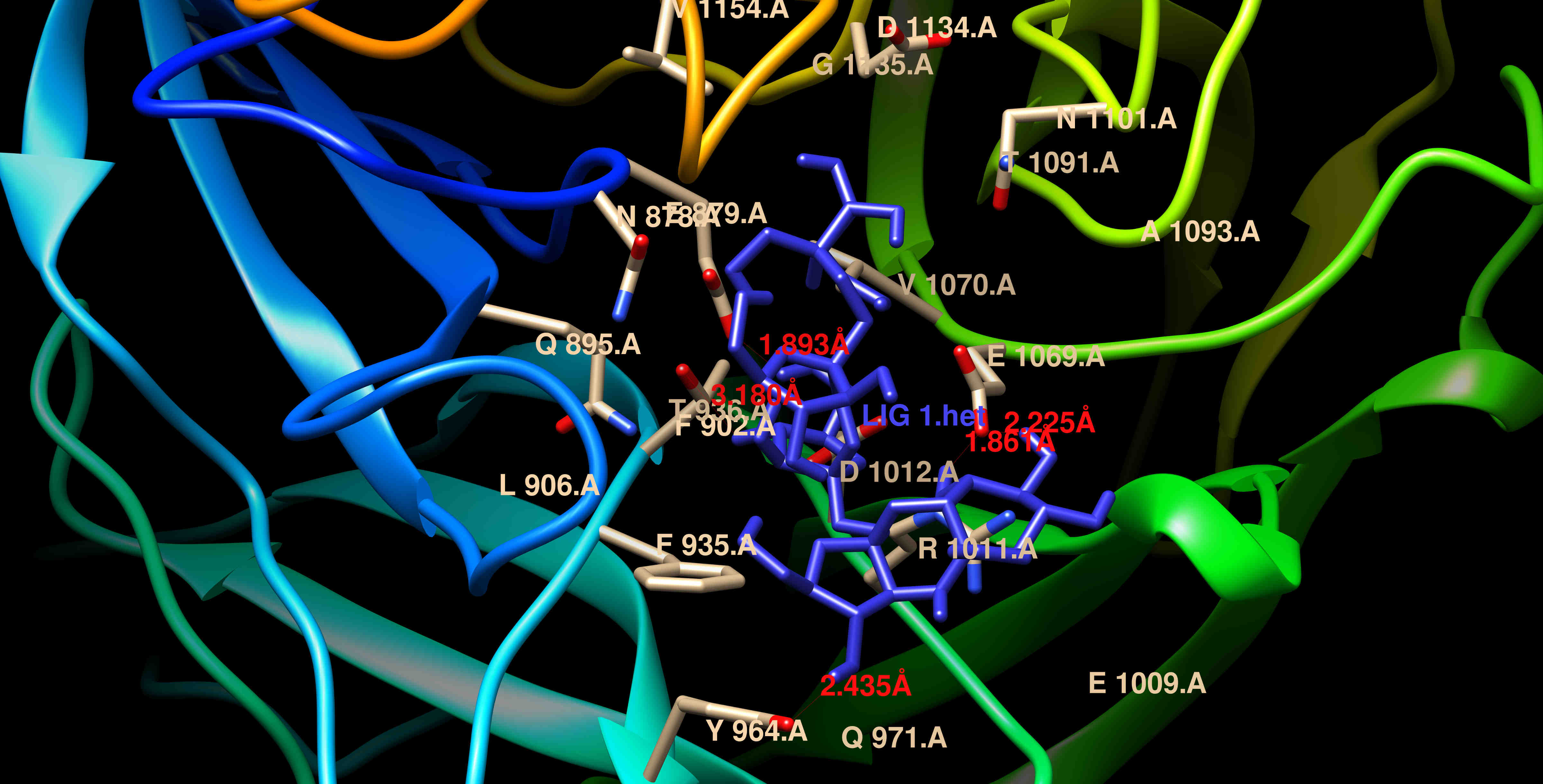

Supplement: S7 Dataset — (ZIP) [file pone.0200607.s007.zip › Docking_Images/SCP18_Docked.jpg]

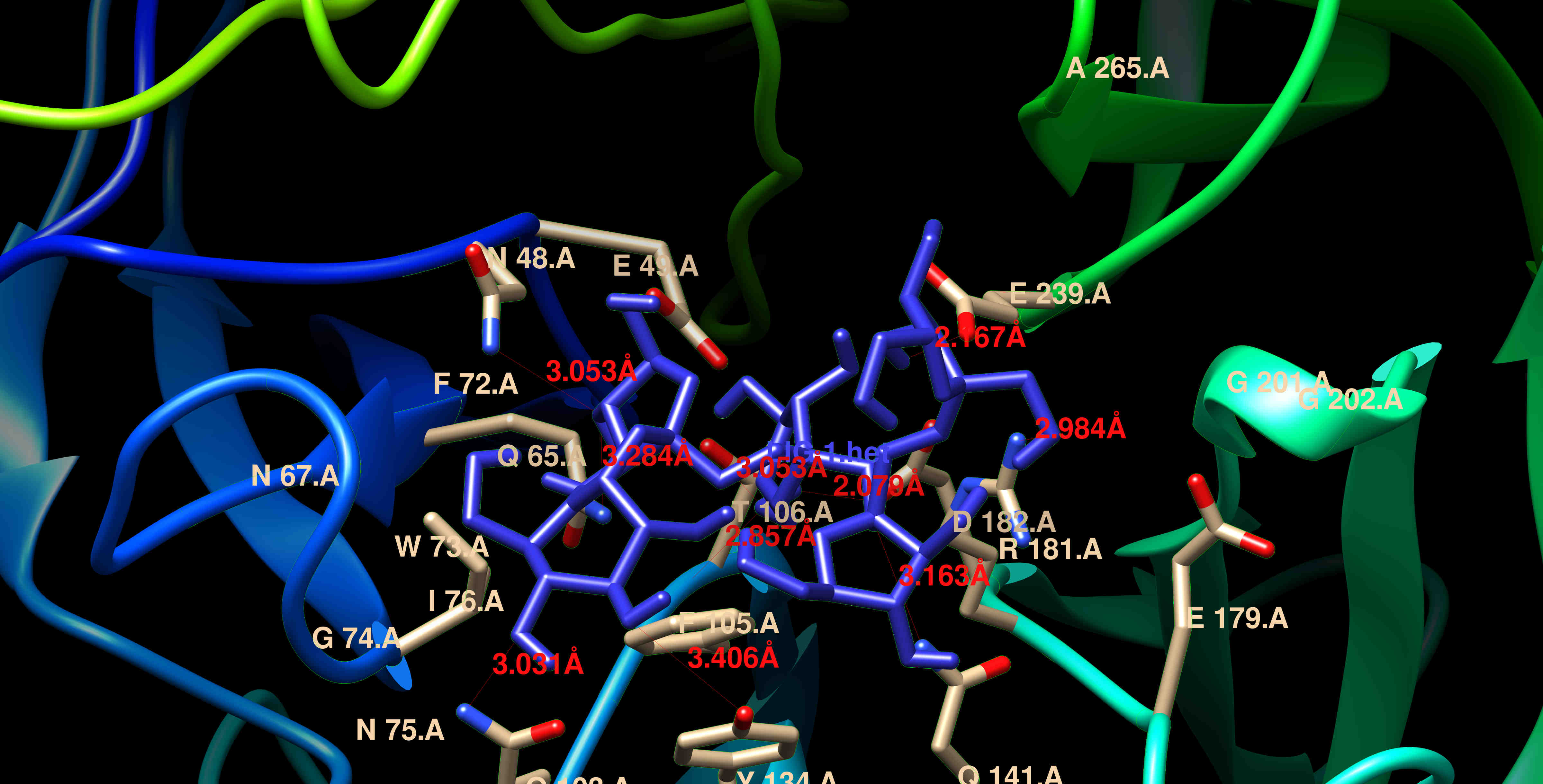

Supplement: S7 Dataset — (ZIP) [file pone.0200607.s007.zip › Docking_Images/SCP1_Docked.jpg]

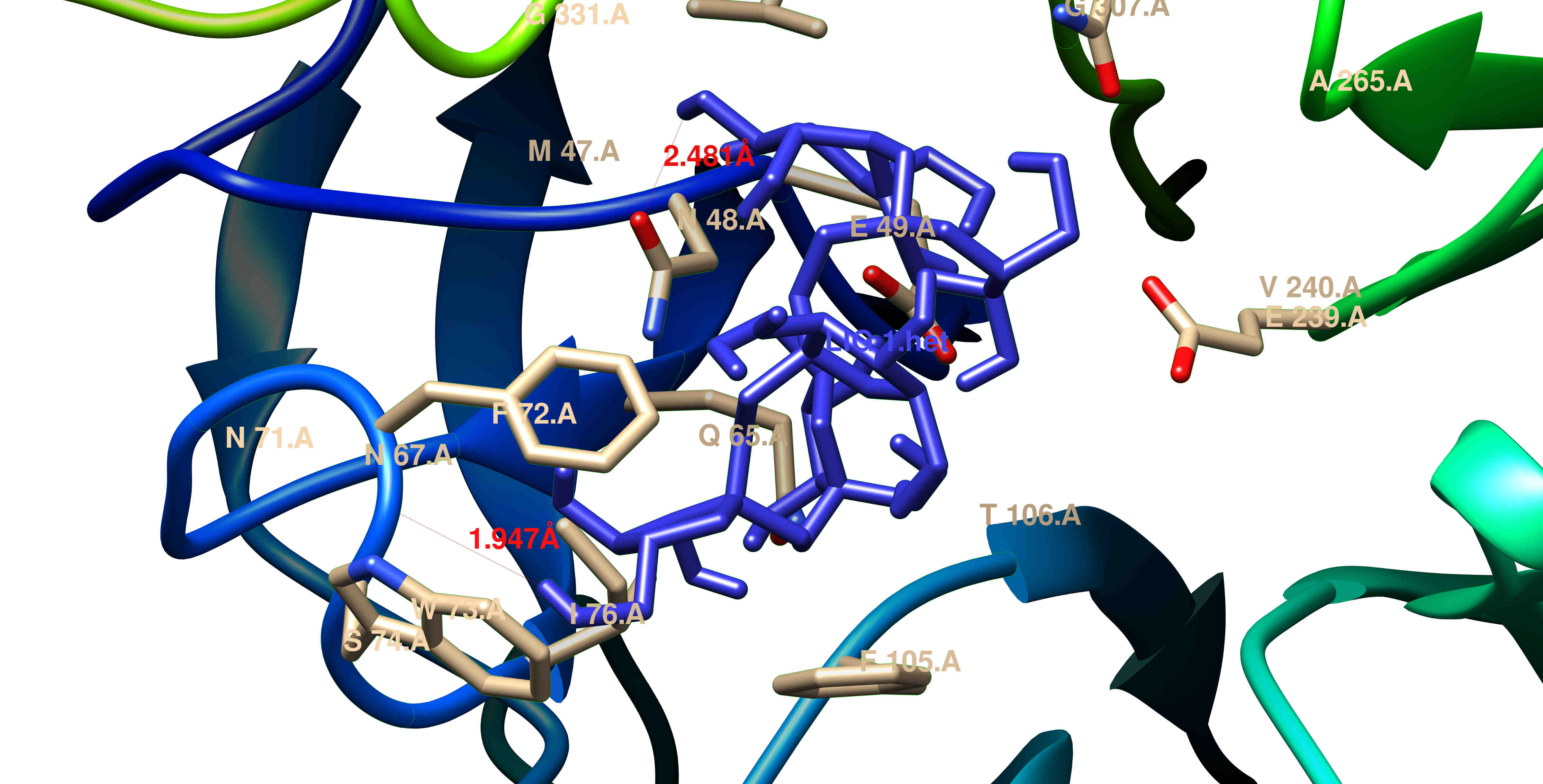

Supplement: S7 Dataset — (ZIP) [file pone.0200607.s007.zip › Docking_Images/SCP2_Docked.jpg]

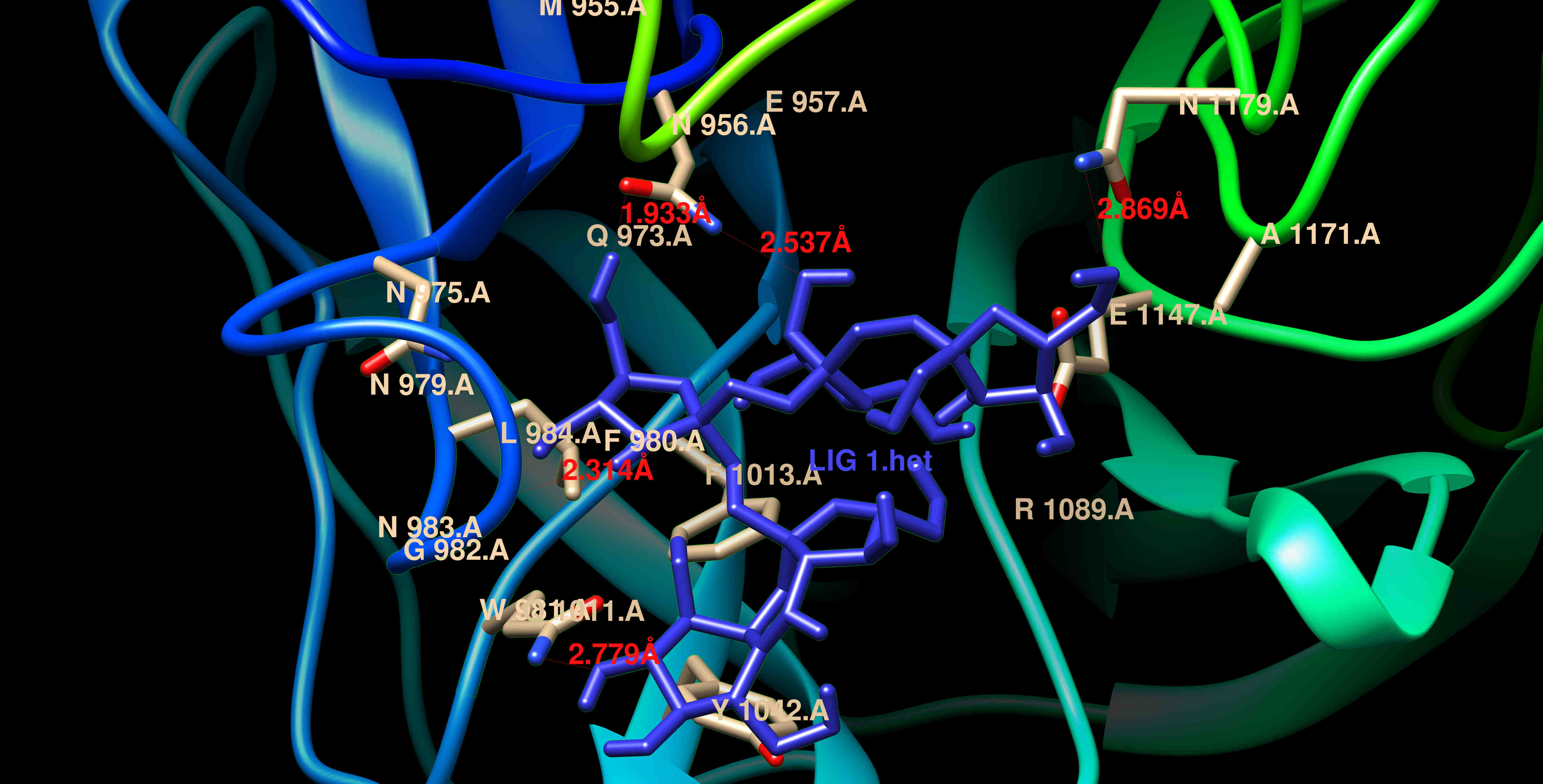

Supplement: S7 Dataset — (ZIP) [file pone.0200607.s007.zip › Docking_Images/SCP3_Docked.jpg]

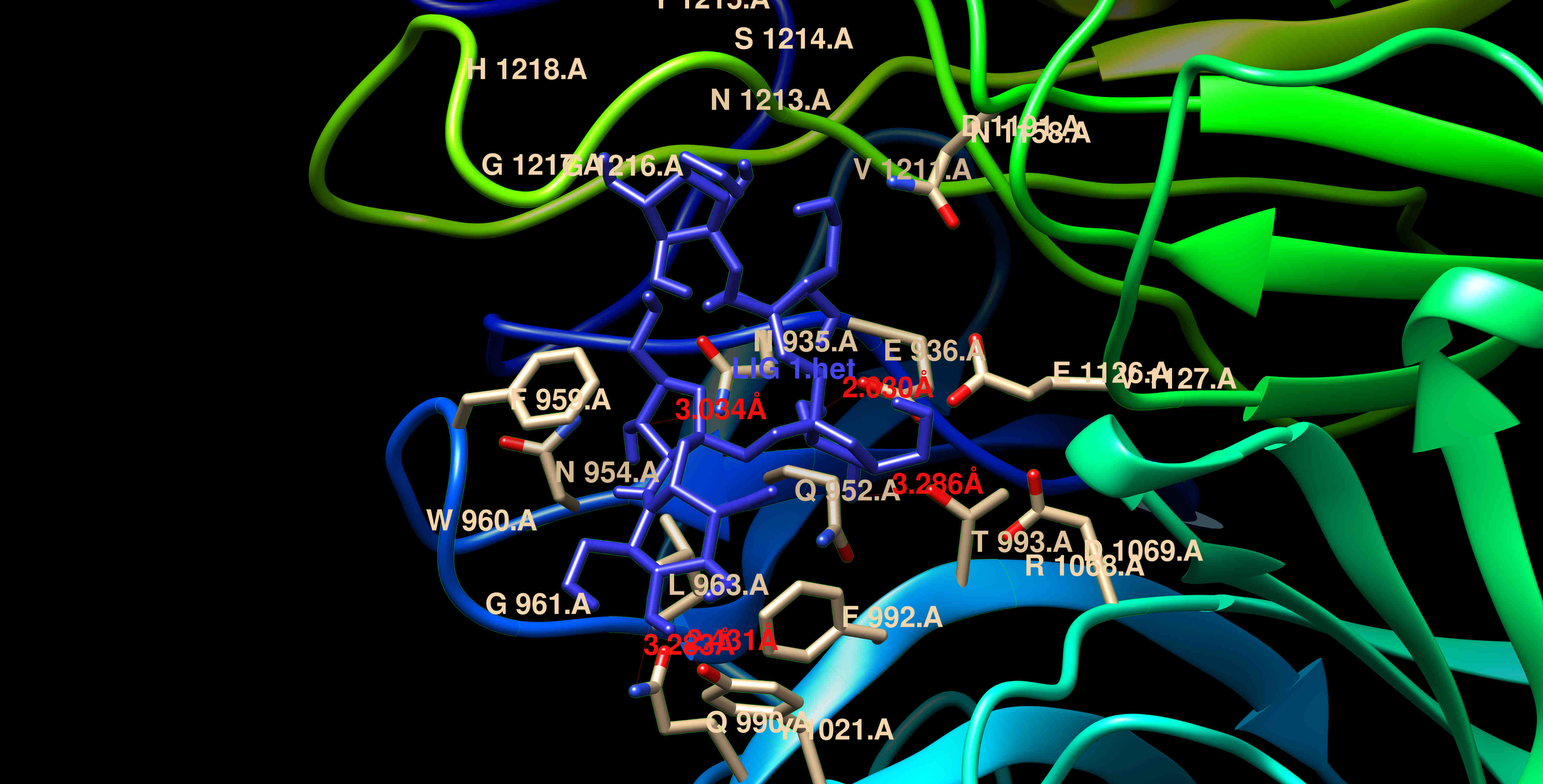

Supplement: S7 Dataset — (ZIP) [file pone.0200607.s007.zip › Docking_Images/SCP4_Docked.jpg]

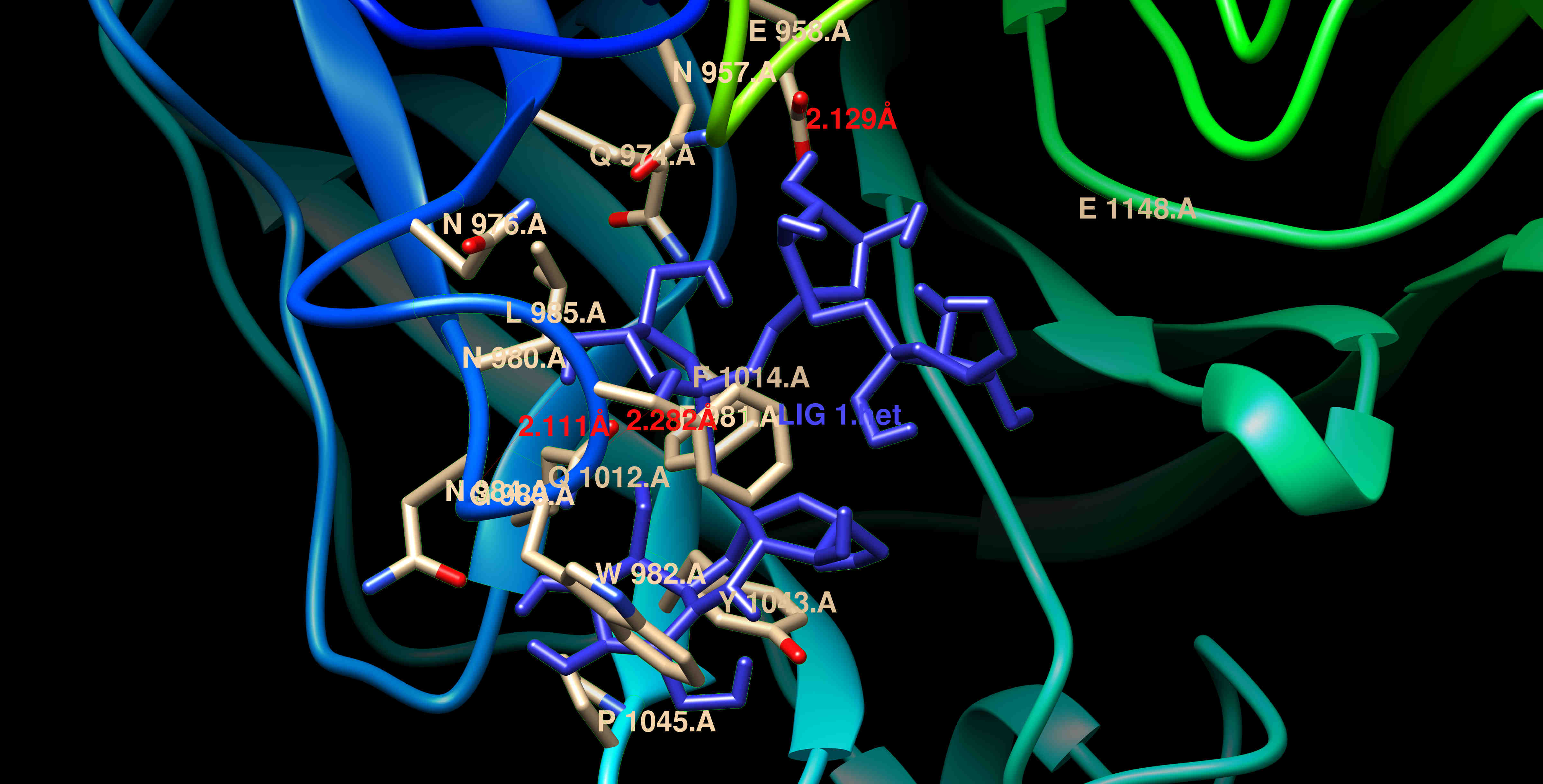

Supplement: S7 Dataset — (ZIP) [file pone.0200607.s007.zip › Docking_Images/SCP5_Docked.jpg]

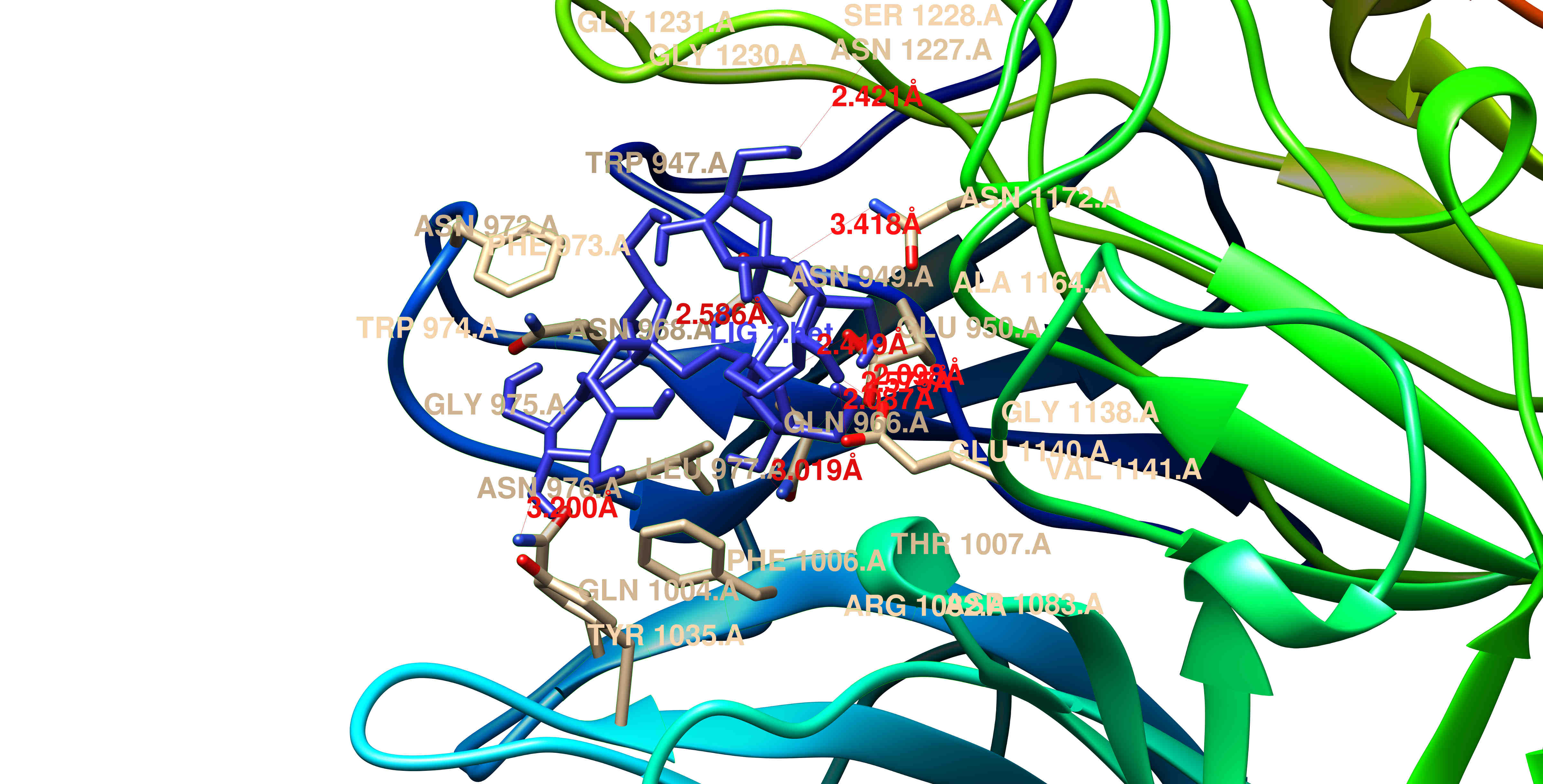

Supplement: S7 Dataset — (ZIP) [file pone.0200607.s007.zip › Docking_Images/SCP6_Docked.jpg]

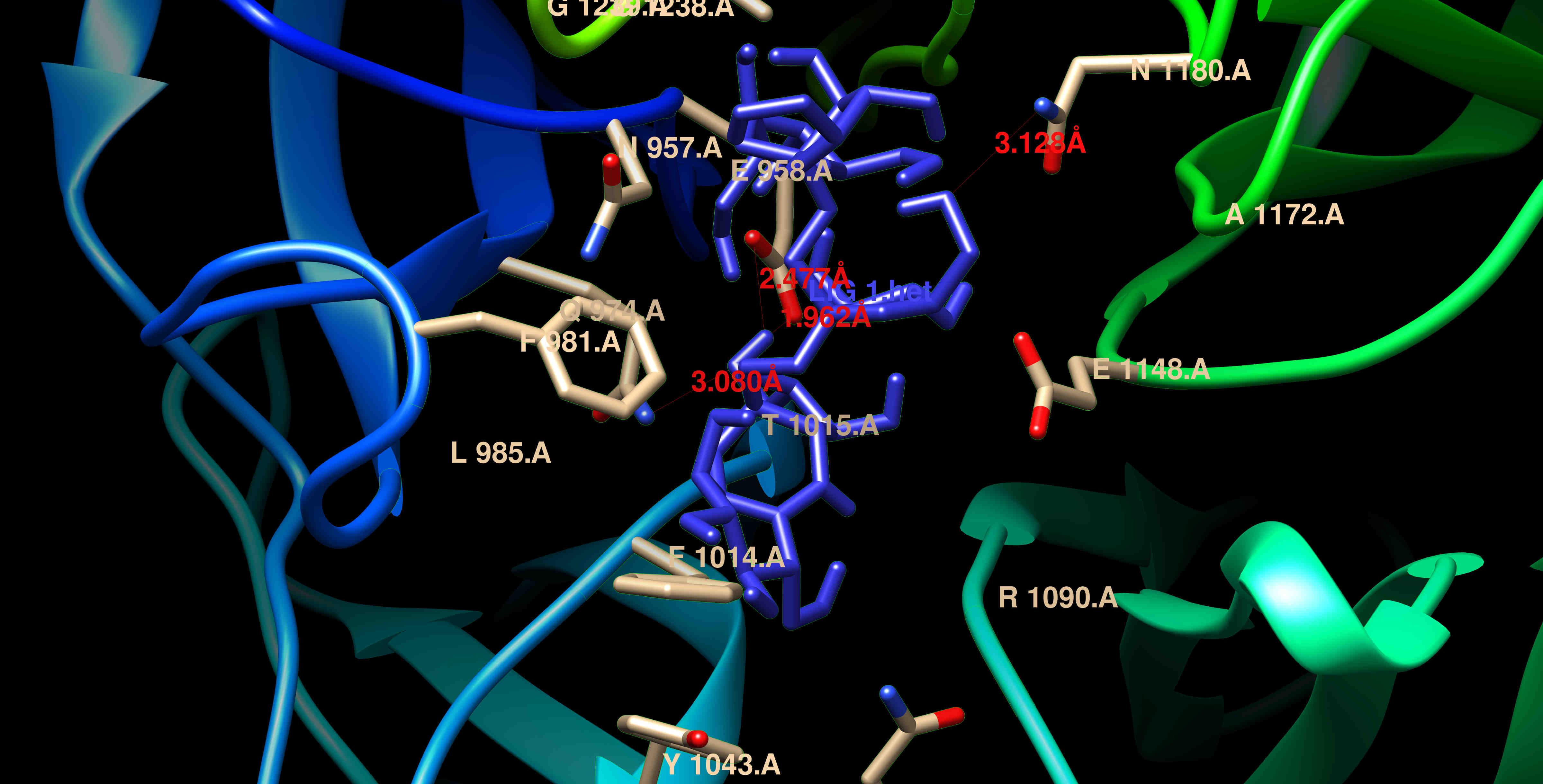

Supplement: S7 Dataset — (ZIP) [file pone.0200607.s007.zip › Docking_Images/SCP7_Docked.jpg]

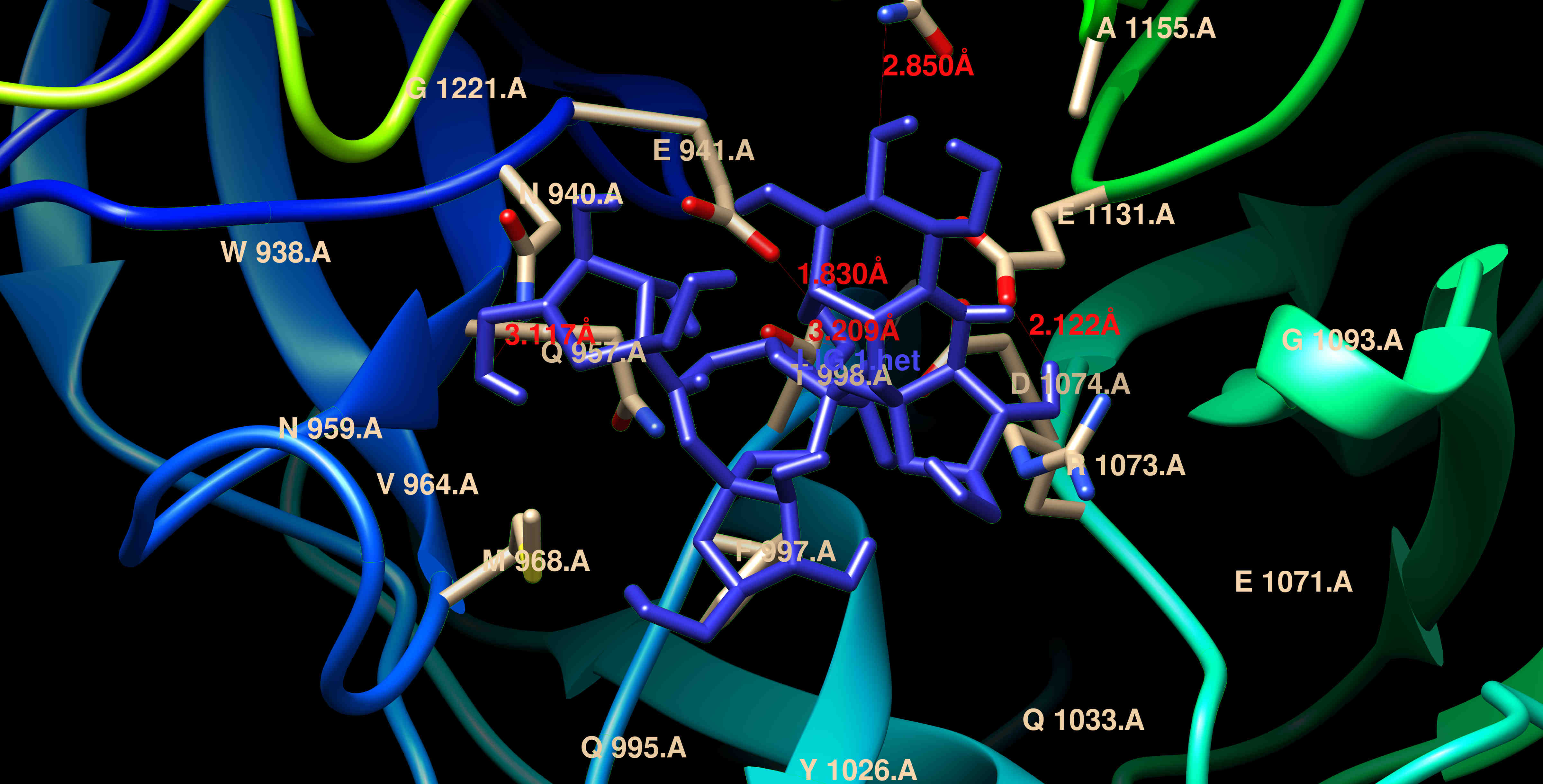

Supplement: S7 Dataset — (ZIP) [file pone.0200607.s007.zip › Docking_Images/TCP1_Docked.jpg]

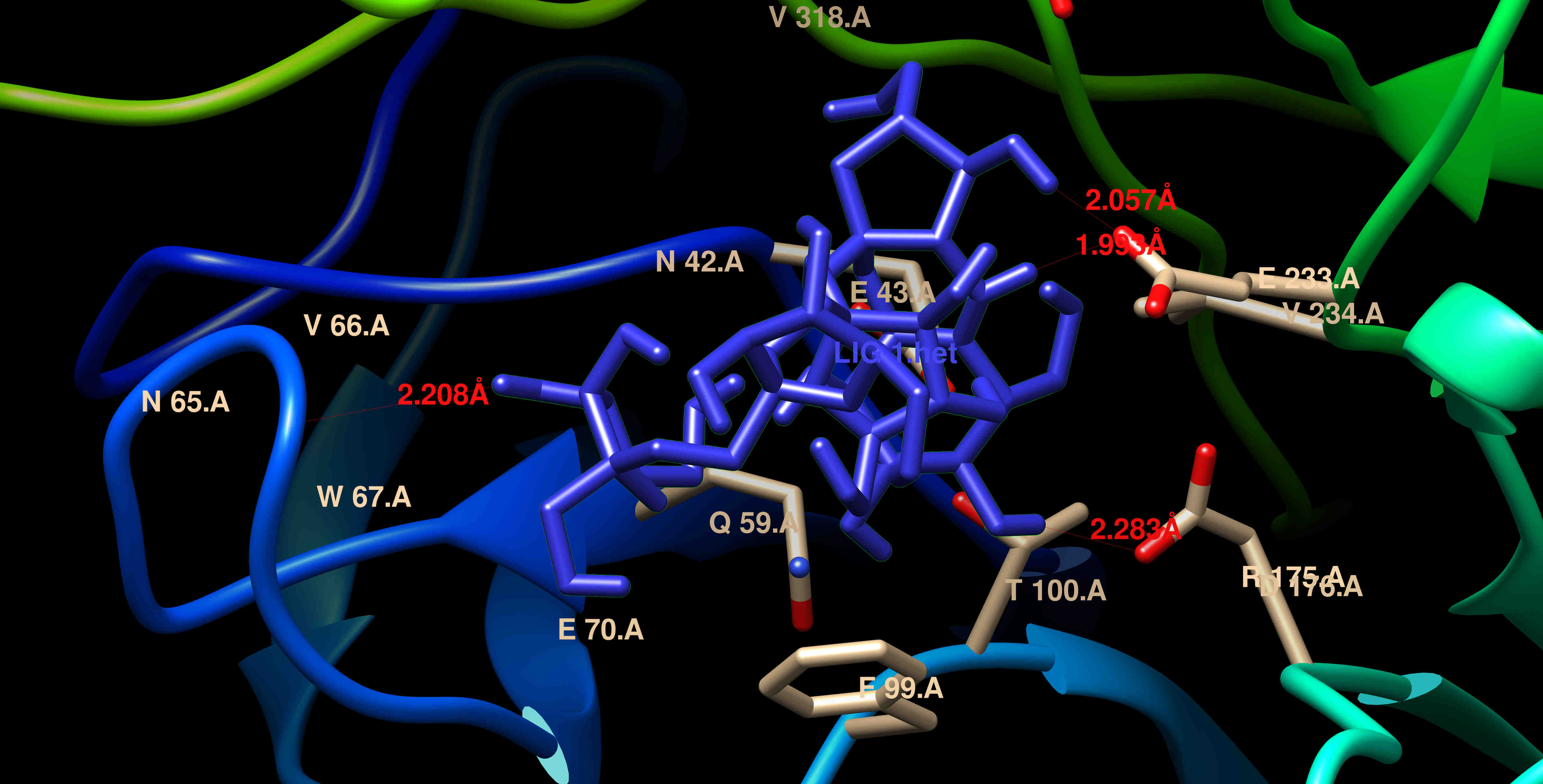

Supplement: S7 Dataset — (ZIP) [file pone.0200607.s007.zip › Docking_Images/TCP2_Docked.jpg]

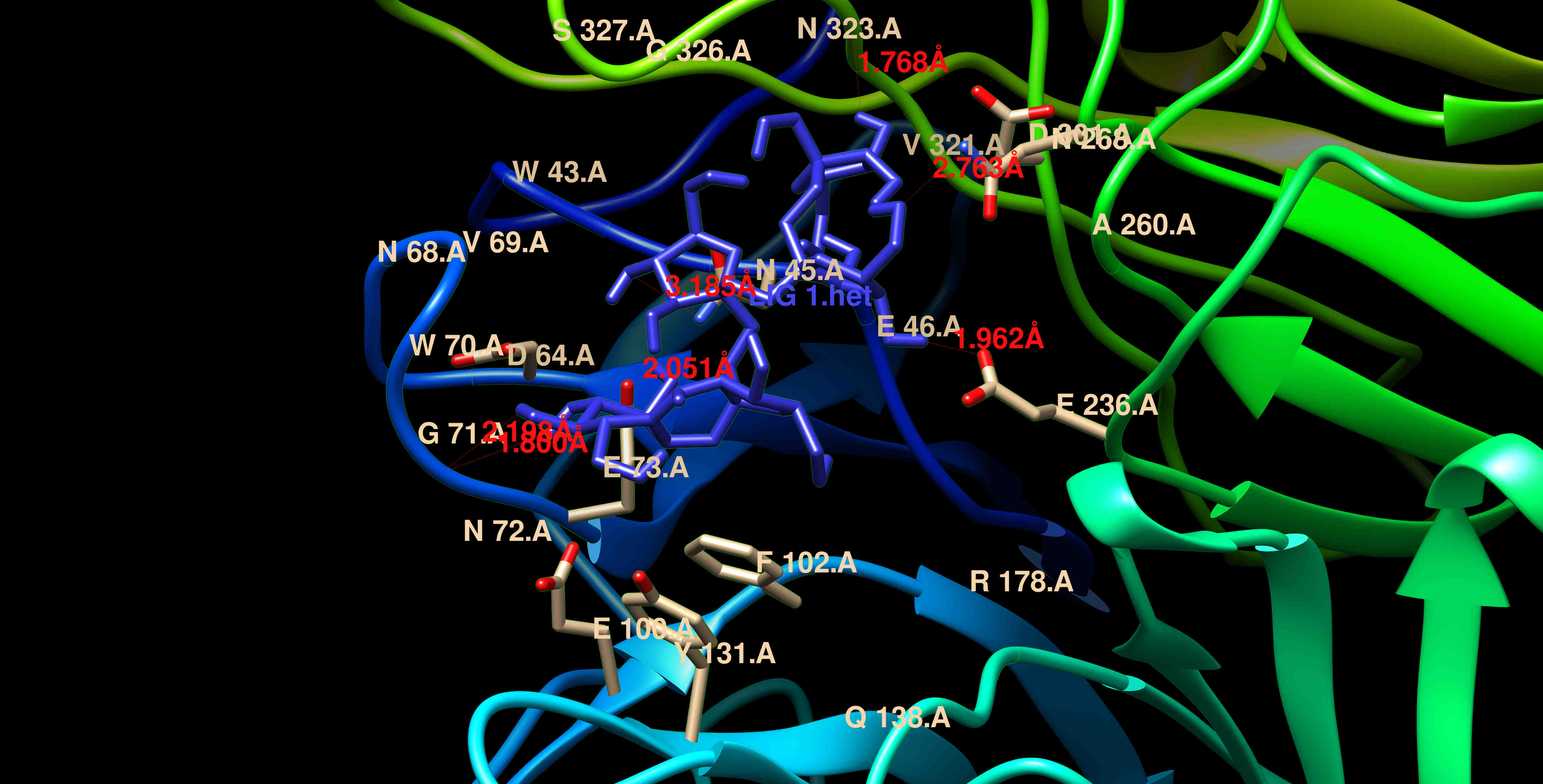

Supplement: S7 Dataset — (ZIP) [file pone.0200607.s007.zip › Docking_Images/TIP1_Docked.jpg]

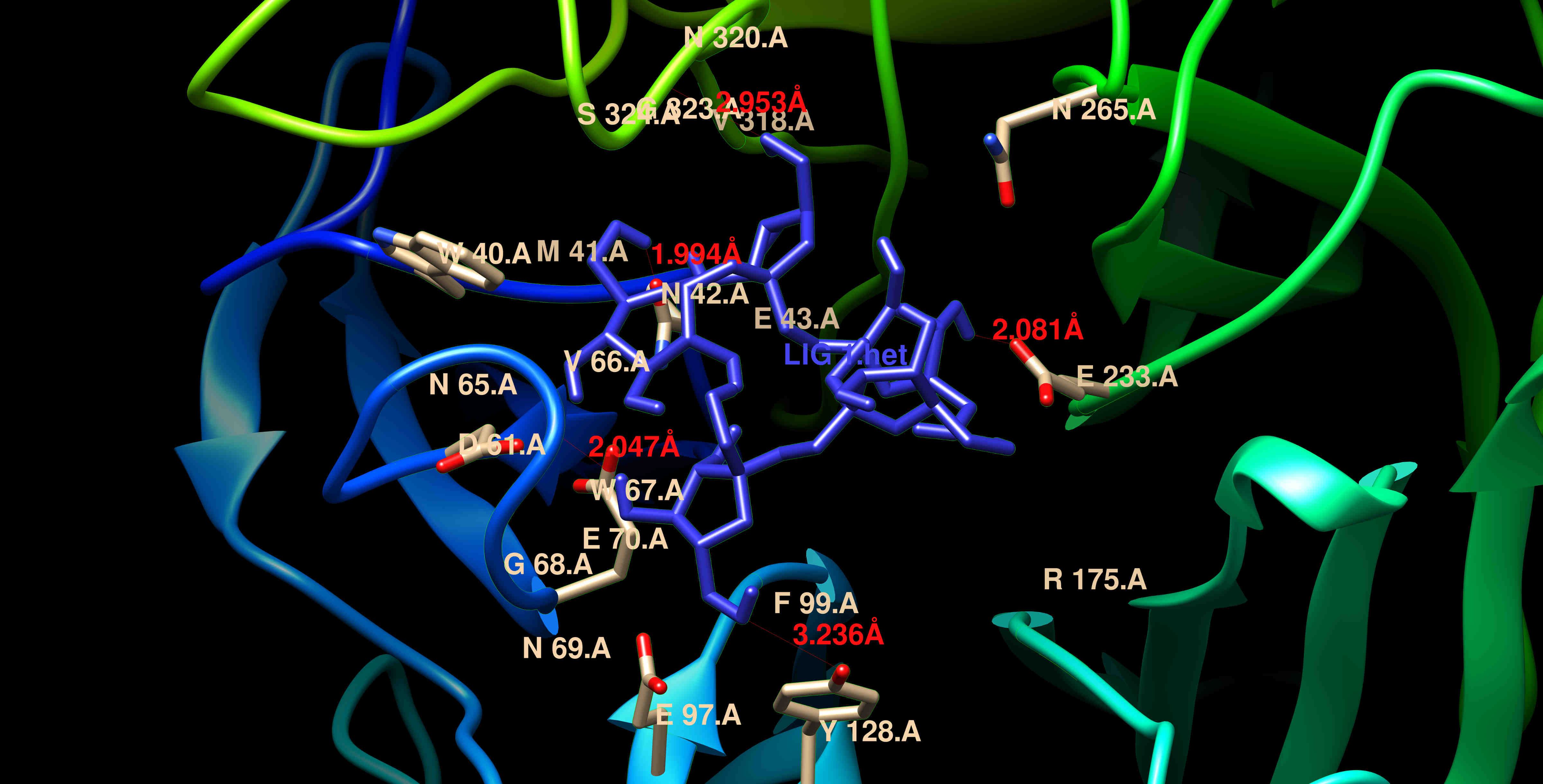

Supplement: S7 Dataset — (ZIP) [file pone.0200607.s007.zip › Docking_Images/TPP1_Docked.jpg]

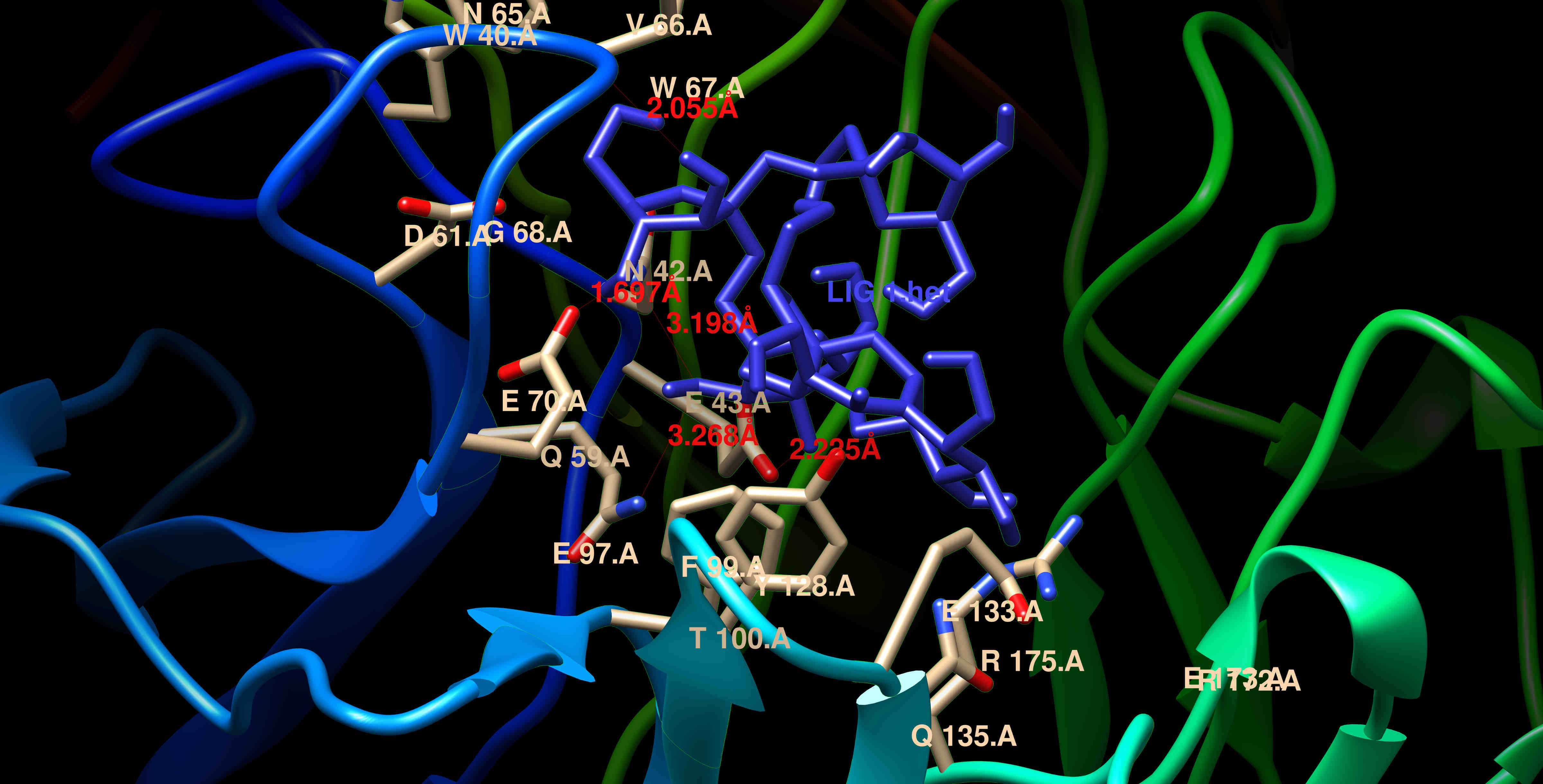

Supplement: S7 Dataset — (ZIP) [file pone.0200607.s007.zip › Docking_Images/TSP1_Docked.jpg]

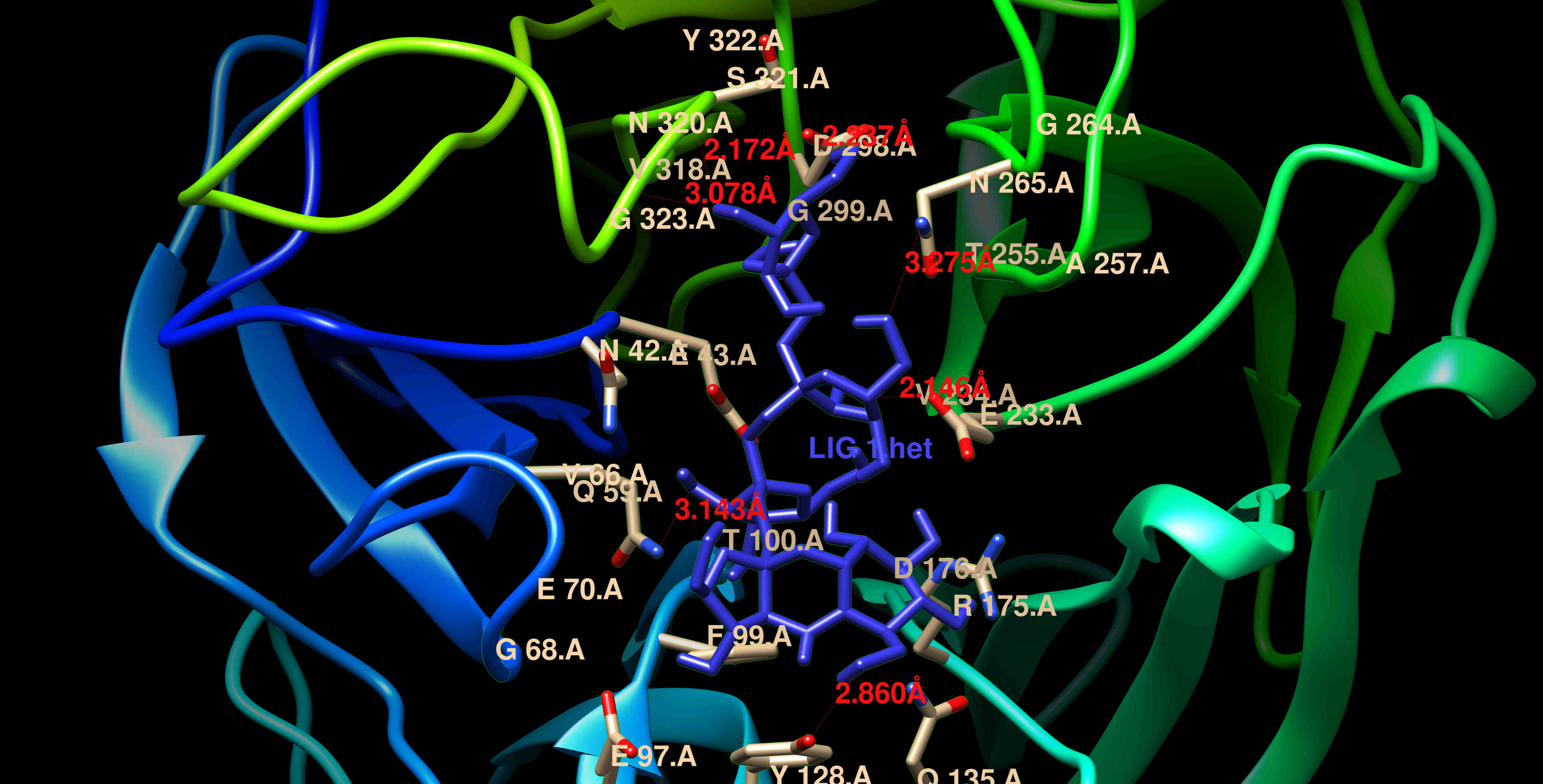

Supplement: S7 Dataset — (ZIP) [file pone.0200607.s007.zip › Docking_Images/TVP1_docked.jpg]

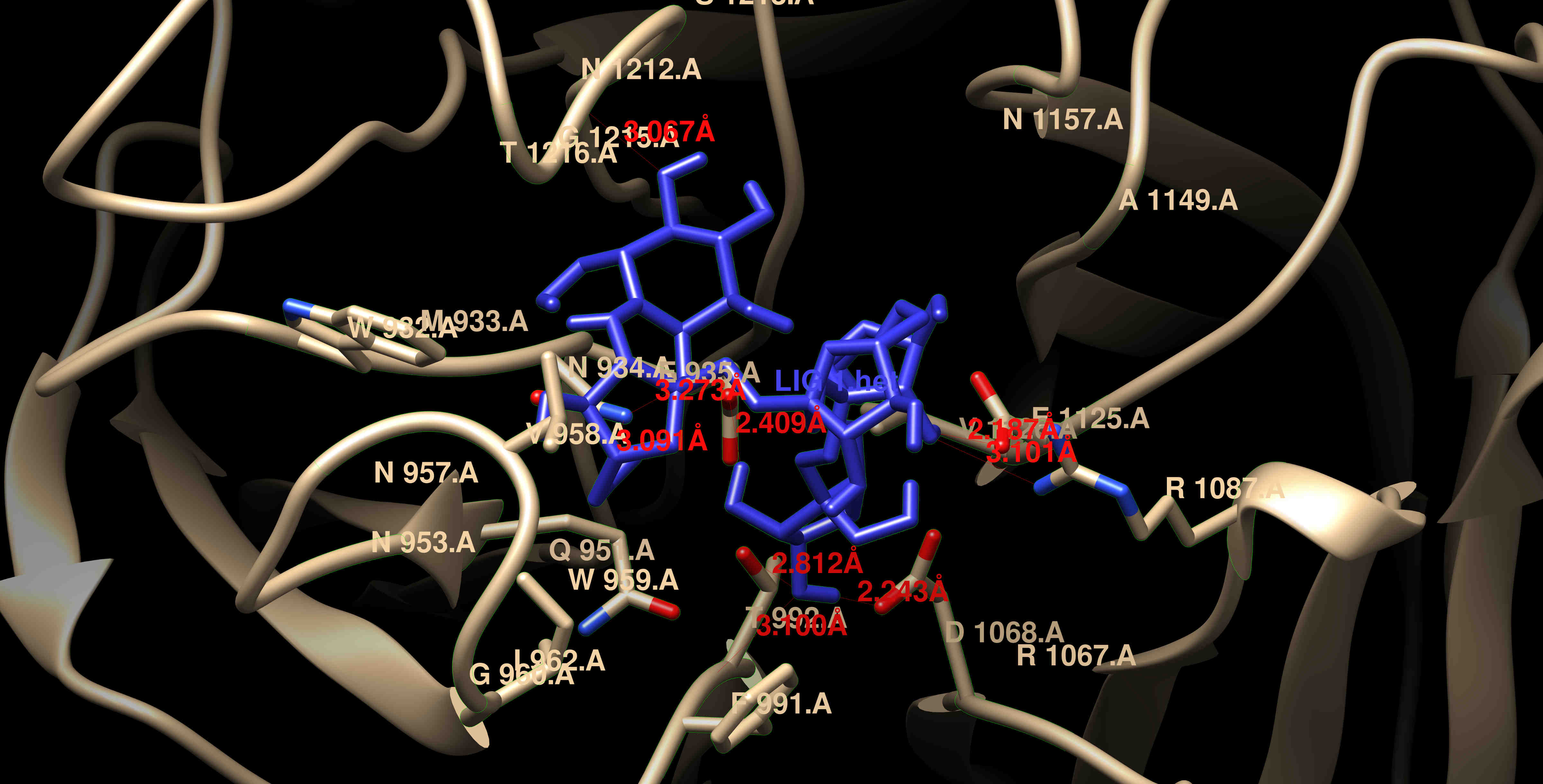

Supplement: S7 Dataset — (ZIP) [file pone.0200607.s007.zip › Docking_Images/TVP2_Docked.jpg]
